# Open-source satellite image pre-processing and annotation workflows: Stranded whale and dolphin case study

**DOI:** 10.1016/j.mex.2026.103949

**Published:** 2026-05-14

**Authors:** Penny J. Clarke, Hannah C. Cubaynes, Karen A. Stockin, Ellen Bowler, Kris Carlyon, Marie R.G. Attard, Peter T. Fretwell, Aliaksandra Skachkova, Asha de Vos, Kaitlin M. McConnell, Encarni Medina-Lopez, Sergio Lopez Dubon, Jennifer A. Jackson

**Affiliations:** a British Antarctic Survey, High Cross, Madingley Road, Cambridge, CB3 0ET, United Kingdom; b University of Edinburgh, King’s Buildings, Thomas Bayes Road, Edinburgh, EH9 3FG, United Kingdom; c Cetacean Ecology Research Group, School of Natural Sciences, Massey University, Auckland, New Zealand; d Animal Welfare Science and Bioethics Centre, School of Veterinary Science, Massey University, Palmerston North, New Zealand; e Marine Conservation Program, Department of Natural Resources and Environment Tasmania (NRE Tas), Tasmanian Government, 134 Macquarie St, Hobart TAS 7000, Australia; f Oceanswell, Colombo, Sri Lanka; g UWA Oceans Institute, University of Western Australia, Crawley, WA, Australia; h Estación Científica Fundación San Ignacio del Huinay, Fiordo Comau, Región de Los Lagos, Chile

**Keywords:** Cetacean stranding, Colonial science, Machine learning, Remote sensing, Standardised training data, VHR optical satellite imagery, Wildlife

## Abstract

Very high-resolution (VHR) satellite imagery can increase the temporal frequency and spatial scale of wildlife studies and complement conventional monitoring methods, to address data gaps globally. However, most gaps in wildlife monitoring, particularly the study of stranded cetaceans, coincide with less-resourced countries, and commercially operated VHR satellite imagery are costly. Increasing access to imagery, expertise, resources, and open access tools is therefore, essential to achieving a globally scalable solution for wildlife monitoring using VHR satellite imagery. This study provides open access workflows to pre-process and annotate satellite imagery by publishing:

• a step-by-step VHR satellite image pre-processing and annotation workflow for detecting features of interest (geolocated point, bounding box and image chip outputs), developed using QGIS 3.28.
• a replicable step-by-step VHR satellite image pre-processing and annotation workflow in Python 3.10.
• a data standard for the ‘satellites to study stranded cetaceans’ community, including a template for recording annotation metadata and accompanying training document, to enable data sharing internationally.

In the long term, greater equity of access to satellite imagery and workflows like those shared here, will innovate applications and methodological developments to improve environmental management outcomes and empower local communities to lead in this space.

## Graphical abstract

**Figure:**
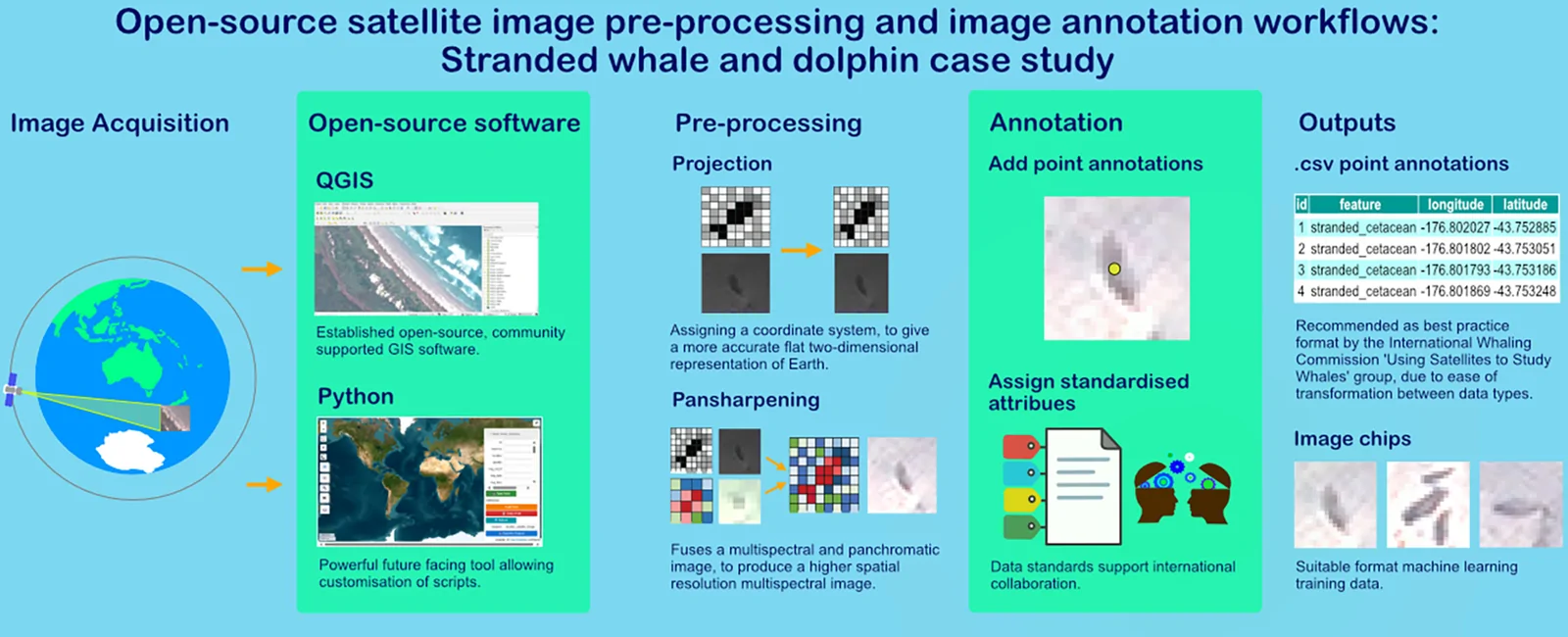

Specifications table

**Table:** 

Subject area	Earth and Planetary Sciences
More specific subject area	Earth Observation
Name of your method	Open-source satellite image pre-processing (projection and pansharpening) and annotation (point, bounding box, and image chip datasets to train machine learning models).
Name and reference of original method	Adapted methodology from: H.C. Cubaynes, P.J. Clarke, K.T. Goetz, T. Aldrich, P.T. Fretwell, K.E. Leonard, C.B. Khan, Annotating very high-resolution satellite imagery: A whale case study, MethodsX. 10 (2023) 102040, https://doi.org/10.1016/j.mex.2023.102040. Validation of methodology published in: P.J. Clarke, H.C. Cubaynes, K.A. Stockin, E. Bowler, K. Carlyon, M.R.G. Attard, P.T. Fretwell, A. Skachkova, A. de Vos, K.M. McConnell, E. Medina-Lopez, S. Lopez Dubon, J.A. Jackson, Odontocete strandings from space: Accurately counting individuals with very-high resolution optical and SAR satellite imagery, Ecol. Inform. (2026) 103924, https://doi.org/10.1016/j.ecoinf.2026.103924. Dataset demonstrating method validity: P.J. Clarke, H.C. Cubaynes, E. Bowler, J.A. Jackson, M.R.G. Attard, K.A. Stockin, K. Carlyon, Point annotation dataset of stranded whale and dolphin species identified in very high-resolution optical and SAR satellite imagery along offshore islands of New Zealand and Tasmania between 2018-2023 (Version 1.0) [Data set], NERC EDS UK Polar Data Centre, (2025) https://doi.org/10.5285/b26c3a3d-73c8-4500–9a23–696011c20a45.
Resource availability	Software: QGIS 3.28 (https://www.qgis.org/download/ (historic versions can be downloaded from: https://download.qgis.org/downloads/), Python 3.10 (full details on how to create an environment and run the code are, available at: https://doi.org/10.5281/zenodo.20547057, for the most up-to-date version see https://github.com/PennyJClarke/strandings_from_space).

## Background

Very high-resolution (VHR; see glossary in supplemental S1) satellite imagery has proven useful for monitoring marine and terrestrial wildlife [[1], [2], [3], [4], [5], [6], [7], [8], [9], [10], [11], [12], [13], [14], [15], [16], [17], [18], [19], [20], [21], [22], [23], [24]]. Owing to their global coverage, the greatest strength of satellites is in extending surveillance to remote locations, increasing the frequency of surveys, and complementing conventional monitoring methods to fill data gaps.

It is evident through research that locally driven efforts in biodiversity and wildlife conservation are most effective [[25], [26], [27], [28], [29], [30]]. However, VHR satellites are commercially operated and the current pricing structure risks facilitating parachute science, by preferentially enabling use of the tool by high income nations [[31], [32], [33]]. ‘Parachuting’ in this manner could lead to externally driven efforts, power imbalances, and dependency on external resources and expertise [29,34]. This is concerning as local knowledge is essential for accurate validation of remotely sensed data. When using satellites to study stranded cetaceans, Clarke et al. [35] identified that people living local to a coastline are best placed to evaluate local research needs, to rapidly coordinate tasking (see glossary in supplemental S1 and Khan et al. [33]) with satellite image providers, and are best aware of realities on the ground. Furthermore, in the case of studying cetacean strandings, the largest monitoring gaps coincide not only with remote and complex coastlines or those deemed unsafe due to conflict, but largely with less resourced coastlines. The same correlation between low to mid-economic activity and gaps in data can be found more broadly in the wider study of biodiversity and wildlife monitoring [[36], [37], [38]].

Increasing accessibility is a key consideration for scaling VHR satellite imagery to monitor stranded cetaceans and wildlife more broadly. This includes access to imagery, knowledge, sharing expertise, infrastructure and open access tools and protocols [39,40]. To date studies using satellites to monitor wildlife have confirmed the value of the tool but rarely disclosed their methodology. While, some studies have adopted open-source software, detailed protocols to replicate such work are not yet available [15,41]. Cubaynes et al. [42] was the first study to provide a full satellite image pre-processing and annotation workflow, but this work uses proprietary software, ArcGIS. Acknowledging the need for greater equality in the field, Clarke et al. [32] recommended the development of publicly available protocols to pre-process and analyse VHR satellite imagery using open-source software, such as QGIS [43], to further support the accessibility of this technology. In addition, the development of best practice open-source standardised workflows and training is also recognised and recommended by the International Whaling Commission, as important for the future use of VHR satellite imagery to study cetaceans [44].

This study aims to build on the work of Cubaynes et al. [42] to share (1) robust standardised open-source protocols for pre-processing and analysing VHR satellite imagery, using stranded cetaceans as a case study, and (2) a data standard/template and descriptive and visual training guide, aimed at the strandings community, to support high quality satellite-based detections. The workflows are also applicable beyond the strandings and wildlife from space community, as it forms a framework supporting remote sensing applications that require image annotation, such as automated detection. The customisable templates/scripts provide users’ flexibility to create a new annotation template or adapt the existing annotation template for other use cases. Uses include the detection of illegal, unreported and unregulated fishing activity, marine plastics, terrestrial applications and non-environmental/commercial sectors.

## Method details

Here, we share two workflows to pre-process and annotate satellite imagery using open-source software, QGIS 3.28 (supplemental S1 and S2) and Python 3.10 (https://doi.org/10.5281/zenodo.20547057 [32], for the most up-to-date version see https://github.com/PennyJClarke/strandings_from_space), to work towards a globally scalable solution for analysing satellite imagery. These workflows are the first of its kind application in using these tools to understand cetacean strandings, the tools are designed for ecologists and conservation managers, and are novel methods for this ecological domain. In addition, we co-developed an annotation template for recording metadata (supplemental S4, alongside .csv files that list the standardised values per attribute, for manually loading the interactive annotation template) and an accompanying descriptive and visual training guide (supplemental S3) for the strandings community, to ensure standardisation across the field of satellites to study stranded cetaceans, and to enable data comparison and reproducibility. By publishing a step-by-step guide that is openly accessible, standardised, and draws upon expert recommendations, these workflows will help foster internationally collaborative efforts to monitor cetacean strandings, and enable the generation of large datasets required for automated detection to make this tool scalable.

**Fig. 1:**
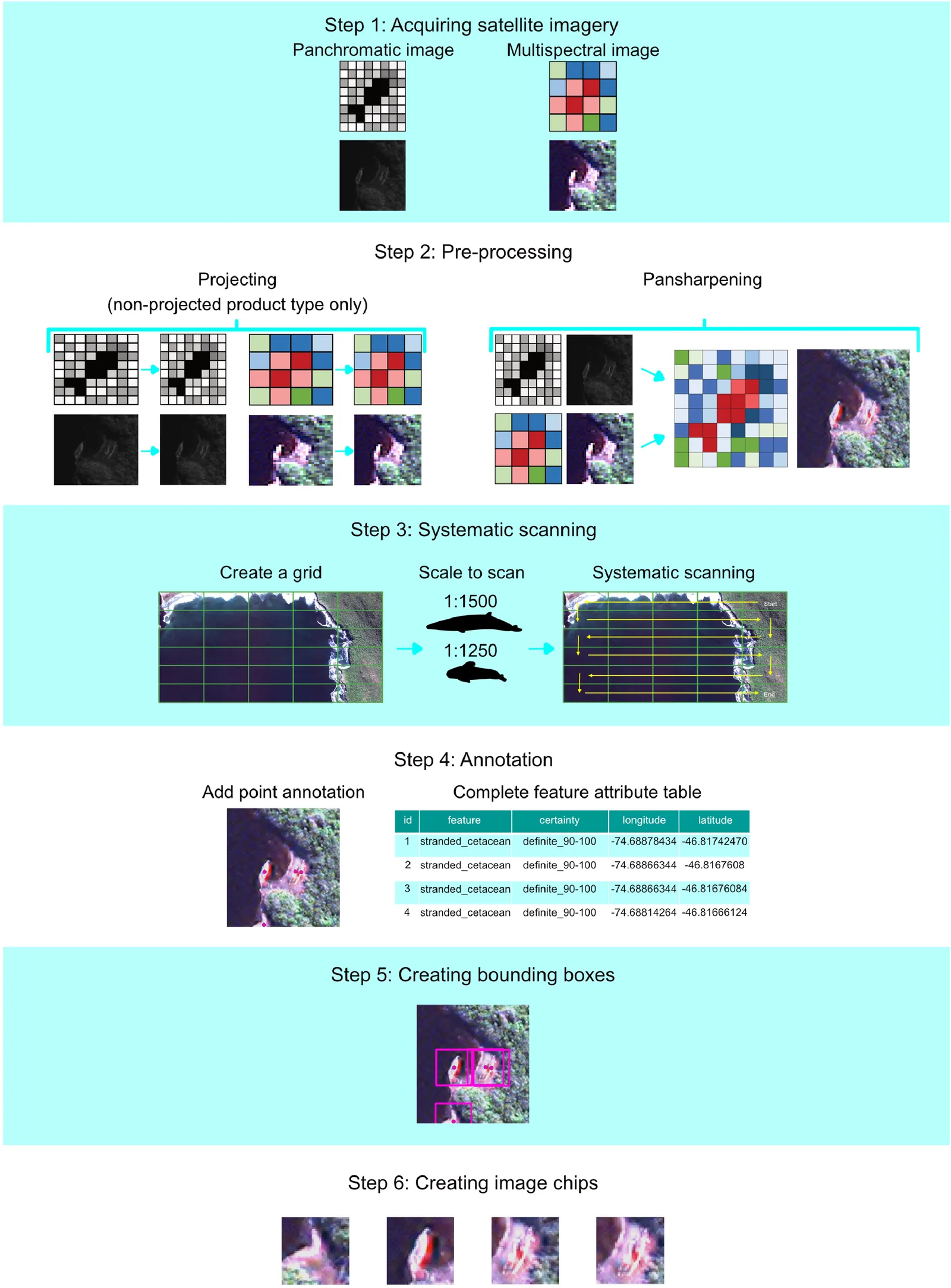
A visual representation of the QGIS and Python workflows, depicting the steps required to build a dataset of annotated cetacean strandings in very high-resolution satellite imagery (an adaption of Fig. 1 in Cubaynes et al. [42]). Satellite imagery © 2026 Vantor.

Both the QGIS workflow and Python pipeline presented here, replicate the steps outlined in Cubaynes et al. [42] ArcGIS workflow, in an open-source format. Full details of the methodology are available in Cubaynes et al. [42], and here in Fig. 1, supplemental S1 (QGIS), and available at: https://doi.org/10.5281/zenodo.20547057 [32] (Python). This methodology was generated for use with VHR optical satellite imagery; however, the workflows can also be used with other imagery such as synthetic aperture radar satellite imagery (SAR), as demonstrated by Clarke et al. [35]. Herein we expand on the methodology from Cubaynes et al. [42], detail any advances or differences in the methodology and outline the data standard developed for the strandings community.

### Software(s)

**Fig. 2:**
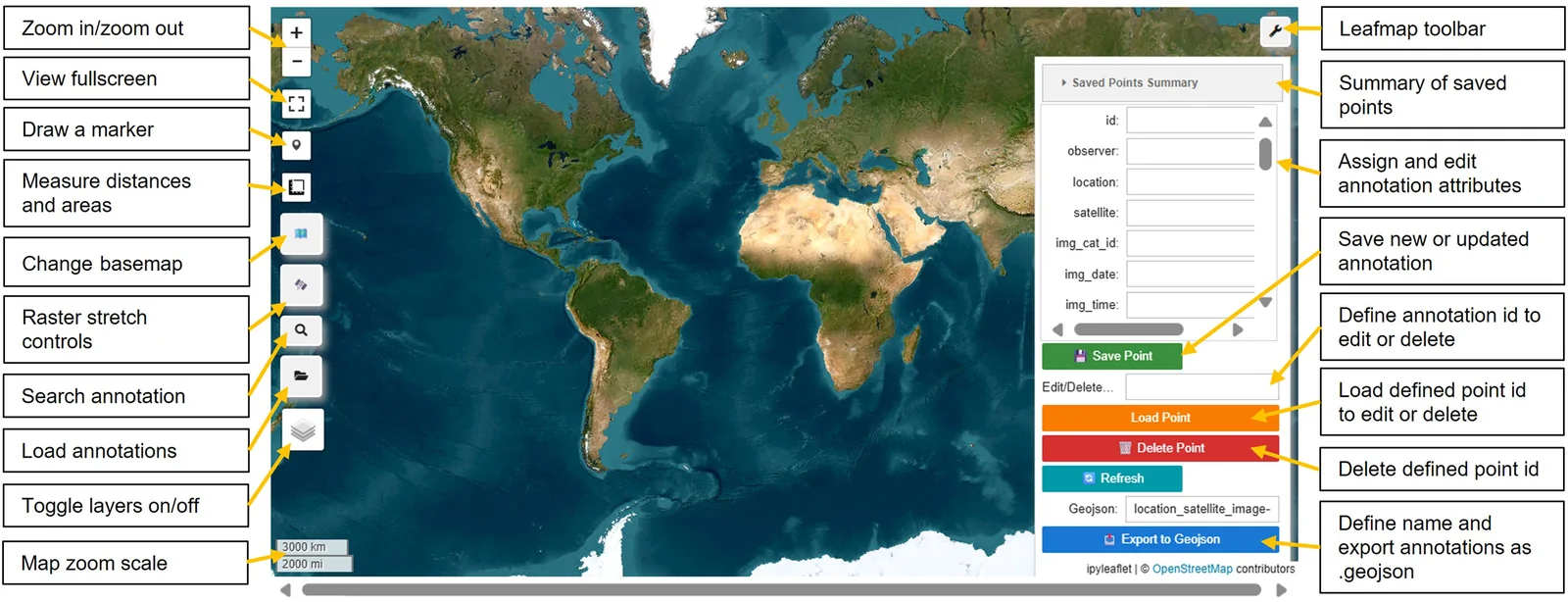
Exemplar of the custom Python satellite image interactive mapping and annotation tool, including the ability to define custom drop-down values (amendable for unique projects) for annotation metadata.

QGIS software was chosen here as it is a reputable open-source geospatial information system (GIS) tool that is well-maintained and supported by a global community [43]. The tool is well suited to the intended audience of our workflow (*i.e.*, ecologists and conservation managers), owing to its user-friendly geographical interface that enables use of powerful analytical tools at the click of a button. The step-by-step workflow shared here uses QGIS 3.28. The importance of open-source software was emphasised, to improve access and uptake in under-resourced regions. Therefore, as well as a user-interface centred pipeline in QGIS, a replicable version of the workflow was developed using Python 3.10 (Fig. 2), available at: https://doi.org/10.5281/zenodo.20547057 [32]. Python is an increasingly popular programming language utilised in data science and machine learning that is widely recognised for its readability and accessibility to beginners [45]. Like QGIS, Python is progressive and its development is driven by an active international network of developers and volunteers. It has a large number of existing libraries for geospatial data manipulation and analysis and machine learning, making it a versatile tool. The Python workflow here, specifically offers a powerful future-facing tool given its ability to allow customisable scripts, which can be adapted to different user’s needs. In addition, the Python workflow streamlines the application of satellites to study wildlife, by enabling users to perform imagery pre-processing, generation of robust standardised training datasets, machine learning, and downstream analytics, in a single software pipeline. The Python workflow was developed by ecologists and remote sensing experts using Jupyter Notebook [46], a single document that combines code, explanations, and outputs, making it understandable for non-coding specialists.

### Step-by-step guidance

#### Step 1: Acquiring satellite imagery

VHR satellite imagery can be ordered through a number of commercially operated satellite imagery providers, most notably Airbus (orders contact^1^ and search image library^2^), Planet (Planet Community^3^, a forum monitored daily, can be used to begin an order enquiry, otherwise contact Planet Support^4^), and Vantor (formerly Maxar Technologies Limited; orders contact^5^ and search image library^6^). Imagery can be ordered through sales representatives, through approved resellers, or more recently through online platforms. For example, Airbus OneAtlas^7^ and the Vantor Hub^8^ (formerly Maxar technologies Ltd MGP Pro), both operate on cloud-based subscription models that include credits for previewing and purchasing imagery. A key advantage of these platforms is the ability to inspect imagery at high resolution prior to purchase, facilitating the selection of suitable scenes. In contrast, offline ordering may offer research and educational discounts not available through subscription, and pricing between offline and subscription-based platforms can vary. While research and education discounts reduce costs, they can come with trade-offs of decreased tasking prioritisation compared to paying customers, and increased latency time between tasking imagery and receiving a successful collection. This is an important consideration for use cases like cetacean strandings, where rapid information provision is critical for both timely detection and on-ground response (*e.g.,* challenges to detection from tides carrying carcasses away, decomposition, and scavengers). Additionally, a number of initiatives exist that may provide a project free access to a quota of imagery, for example, the European Space Agency’s Third-Party missions.

Unlike coarser spatial resolution satellite sensors that collect imagery continuously (see glossary in supplemental S1 and Clarke et al. [32]), VHR satellite sensors capture imagery upon request. To order imagery, a provider will require details including, the area of interest (for example, a .shp or .kmz file), the desired spatial and spectral resolution (see glossary in supplemental S1 and Clarke et al. [32]), the level of pre-processing (see section 2.2 on pre-processing) and the file format. New image collections can be tasked. In addition, after an elapsed period of time, providers store historic imagery in an archival library, which can be accessed and ordered, typically at a reduced cost. When enquiring about tasked imagery a provider can utilise the product specification provided by the user, to return a feasibility window (image collection time that fulfils a user’s product specification criteria) for achieving successful collections. For both archival and tasked imagery, orders may be subject to a minimum order size (km^2^).

When ordering archival or tasked imagery, the data received will likely include a panchromatic and multispectral image file (see glossary in supplemental S1 and Khan et al.[33]). Exceptions would be images ordered, for example, from Vantor’s WorldView-1, a panchromatic only sensor. Panchromatic and multispectral imagery can be identified through the image file naming convention or the image metadata (see supplemental S1). Panchromatic images have a single band, whereas multispectral images can have for example, four, six, or eight bands. The band combinations to visualise multispectral imagery can be ascertained by reviewing the satellite sensor specification, either online from the satellite image provider or within the image metadata file (see supplemental S1).

Depending on the image provider, images may arrive in different formats. For example, Vantor provides images in a .tif format, whereas Airbus imagery may be supplied in .jp2 format. In the case of Vantor imagery, large image files may be delivered as multiple .tif files, which are compiled into one .til file. Details on how to load imagery within QGIS are available in supplemental S1, for adding Vantor imagery, best practice is to select the .til format. Alternatively, when using Python, the workflow loads imagery using Rasterio [47] (a Python package designed for working with raster data), and for instances where large image files are supplied over multiple .tif files, the code enables mosaicking (joining multiple .tif files) to create a single .tif image, rather than loading the .til file.

#### Step 2: Pre-processing

Satellite image companies can provide images at differing levels of pre-processing, which are defined by the user at the time of ordering imagery. Pre-processing can include, projection, orthorectification, pansharpening, and atmospheric correction of imagery (see Khan et al. [33] for more details). For the purpose of annotation, the pre-processing steps of importance for detection of stranded cetaceans, are geolocation correction (projection and orthorectification of basic products like Vantor’s System-Ready 1B data) and pansharpening. Atmospheric correction is the process of removing the effect of the atmosphere, clouds, and aerosols on reflectivity, to get a representative spectral signal from the target feature, otherwise known as surface reflectance (British Antarctic Survey, 2023). Atmospheric correction is important for work conducting spectral analysis and not applicable for annotation.  

*Projecting satellite imagery*

When a satellite image is collected, the image is a flat representation of a spherical Earth, and the image may therefore appear distorted. By assigning a coordinate system that is most relevant to the image area through projection/re-projection, the image representation will show a more accurate flat two-dimensional representation of the Earth. A projection is a mathematical transformation of a coordinate system [48]. A coordinate system defines the position of a feature, for example a point on Earth, in a two or three-dimensional space. Universal Transverse Mercator (UTM), is a type of Projected Coordinate System (PCS), which is a series of grid zones of the world, a plane coordinate grid (more details and a guide to determining an images UTM zone can be found on the following link: https://www.dmap.co.uk/utmworld.htm).

Cross-referencing an image to the product type (Table 1) can help determine if an image requires projection/re-projection. For example, the imagery used to demonstrate the QGIS step-by-step guidelines in supplemental S1, was Vantor’s View-Ready (Standard) 2A data. Vantor’s View Ready (Standard) 2A data is projected, whereas Vantor System Ready 1B data is not and a re-projection is required. In both QGIS and Python projection can be executed using GDAL warp [49] (here the QGIS workflow uses version GDAL 3.6.0 and the Python workflow uses GDAL version 3.10.3). The process of geolocation correction presented here utilises Rational Polynomial Coefficients (RPC). RPC are embedded into VHR satellite imagery metadata, and effectively document the pixel location in relation to geolocated coordinates (longitude and latitude) based on the sensors position in relation to the Earth’s surface.

**Table 1:** List of product types for the main very high-resolution satellite imagery providers adapted from Cubaynes et al. [42].

Satellite operator	Product name	Mapping projection
Airbus	Primary	Coordinate Reference System: WGS84 Map projection: None
	Projected	Coordinate Reference System: WGS84 Map projection: UTM
	Ortho	Coordinate Reference System: WGS84 Map projection: UTM
Albedo	Photogrammetry Ready	New sensor - information currently not available
	View Ready	New sensor - information currently not available
	Analysis Ready	New sensor - information currently not available
Planet	SkySat Basic Scene	Coordinate Reference System: WGS84 Map projection: None
	SkySat Ortho Scene	Coordinate Reference System: WGS84 Map projection: UTM
	SkySat Ortho Collect	Coordinate Reference System: WGS84 Map projection: UTM
Vantor (formerly Maxar Technologies Ltd)	System-Ready (Basic) 1B	Coordinate Reference System: WGS84 Map projection: None
	System-Ready Stereo (Basic) 1B	Coordinate Reference System: WGS84 Map projection: None
	View-Ready (Standard) OR2A	Coordinate Reference System: WGS84 Map projection: UTM
	View-Ready Stereo (Standard) OR2A	Coordinate Reference System: WGS84 Map projection: UTM
	View-Ready (Standard) 2A	Coordinate Reference System: WGS84 Map projection: UTM
	Map-Ready (Ortho) 3D (formerly: Map-Ready (Ortho) 1:12,000)	Coordinate Reference System: WGS84 Map projection: UTM

To perform a geolocation correction, the method requires orthorectification. Orthorectification is the process of removing image distortions caused by the local topography and sensor collection angle, to create an image where features are represented accurately [50]. In the application of image annotation for cetacean strandings and cetaceans at sea, orthorectification could be performed without detailed elevation information, as image elevation is close to sea level. Here we document how to project both with and without inclusion of a Digital Elevation Model (DEM) for both QGIS and Python. Research suggests higher spatial resolution DEMs (0.31 m) are optimal for achieving accurate orthorectification [51]. In absence a freely available coarser (30 m) resolution DEM can be used, like Copernicus or Shuttle Radar Topography Mission (SRTM), however, geo-correction accuracy diminishes (root mean square error of 7.94 m) [51]. Although, not detailed as part of this workflow, the accuracy of a projected image output can be determined by calculating the difference between a known (surveyed) geolocated (longitude, latitude) ground control point(s) (GCP) and the corresponding location in the projected image product.  

*Pansharpening satellite imagery*

A satellite image is made up of lots of pixels, each pixel (square) is the equivalent to a measurable distance on the ground, this is called spatial resolution (see glossary, supplemental S1). Panchromatic images are grayscale images captured across the visible (and possibly near-infrared) wavelengths of the electromagnetic spectrum. Multispectral images‌ cover more bands of the electromagnetic spectrum but each band covers a narrow portion of the visible, near-infrared, and shortwave infrared wavelengths of the spectrum. A panchromatic image will have a higher spatial resolution than a multispectral image as it captures a wider range of light in a single band, allowing it to be significantly sharper and more detailed [52]. For example, Vantor's WorldView-3 sensor, produces a panchromatic image with a spatial resolution of 0.31 m, and a multispectral image with a spatial resolution of 1.24 m.

To analyse a multispectral image, it is useful to enhance its spatial resolution to the higher spatial resolution of the panchromatic image, through the process of pansharpening. Pansharpening takes the panchromatic and multispectral images, and where the two rasters fully overlap, fuses them together to produce a multispectral image with the higher spatial resolution of the panchromatic image [53]. Pansharpening is useful for studying cetacean strandings from space as both spectral and spatial richness is needed to discriminate individual features with confidence. In both QGIS and Python pansharpening can be achieved using Orfeo Toolbox (OTB) [54] (BundleToPerfectSensor) or GDAL [49]. OTB is recommended over GDAL as it preserves the multispectral resolution of the original image. Details on how to execute OTB and GDAL for QGIS can be found in supplemental S1 and for Python available at: https://doi.org/10.5281/zenodo.20547057 [32]. Compared to ArcGIS, which produces a virtual raster, pansharpening with QGIS creates a new image file. This takes a considerable amount more time and so clipping an image to a region of interest prior to pansharpening may be advisable, guidance is provided in supplemental S1.

**Fig. 3:**
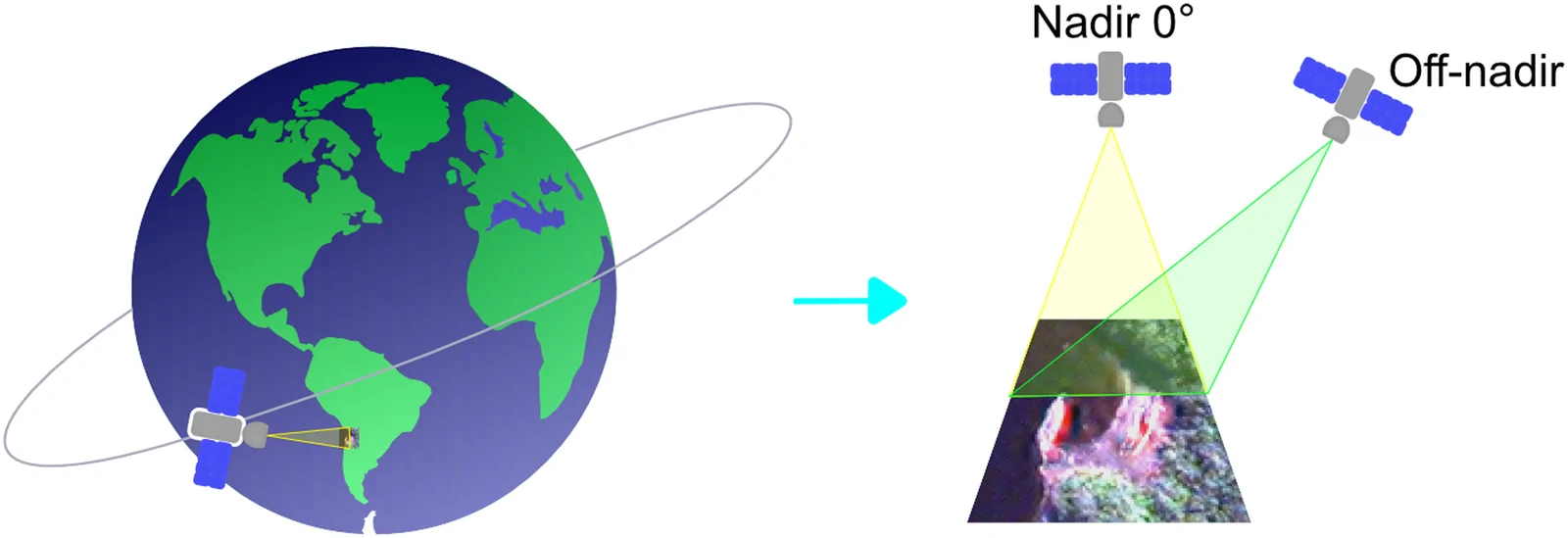
Depiction of nadir (yellow) and off-nadir (green) image collection angles, satellite imagery © 2026 Vantor.

When an image is received the spatial resolution may be less than the advertised sensor spatial resolution, due to variations in nadir angle (see Khan et al. [33]). Nadir angle refers to the angle of the satellite to the target area or feature on Earth, at the time of collection [55]. Nadir is the point on Earth directly below a satellite, off-nadir is a sideways look at Earth (Fig. 3); the greater the angle, the greater pixels in an image are stretched, resulting in a lower spatial resolution and sometimes, distortion. While off-nadir reduces the spatial resolution, it can provide interesting perspectives and insights into features that are otherwise missed where an overhead view may obscure details.

#### Step 3: Systematic scanning

**Fig. 4:**
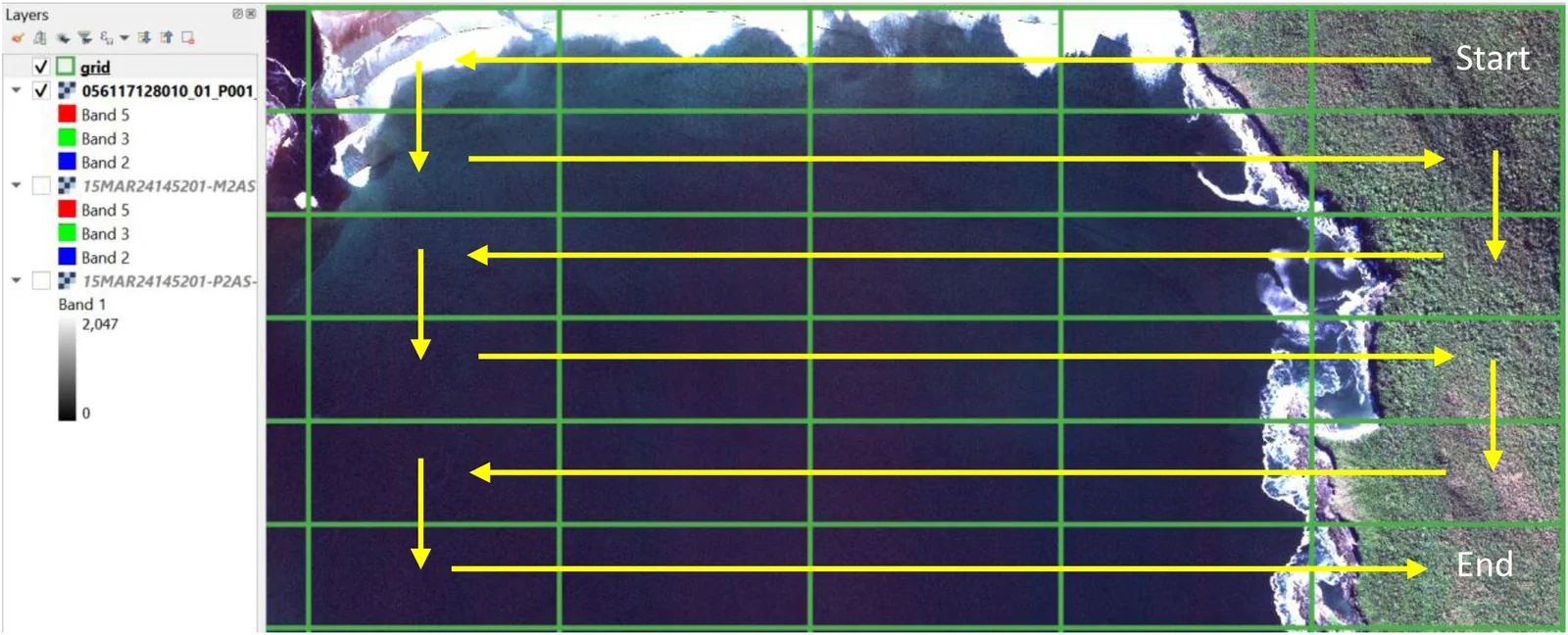
Example of a grid (green boxes) overlaid on a satellite image (satellite imagery © 2026 Vantor) for systematically scanning an image in an s pattern (yellow arrows indicating direction to scan) to ensure no detections are missed.

To ensure no features of interest are missed, it is important to adopt systematic methods of scanning. A good approach is overlaying a grid on an image, as recommended by Cubaynes et al. [42]. The grid allows for marking cells as complete, or an opportunity to note the cell number and direction of movement when pausing or stopping scanning as a reference to return to. Scanning in an ‘s’ type pattern, is a good method for systematically scanning an image using a grid (Fig. 4). For annotating imagery of stranded cetaceans only, an alternative systematic approach could be to restrict the area of interest to follow the coastline instead of an s-shape method. For some projects, monitoring of stranded cetaceans may extend to floating cadavers in near and offshore waters and parallel monitoring of live whales at sea. In this case a systematic ‘s’ shape pattern can focus on the grid cells that encompass both the coastline and open water.

For a guide on suitable zoom scales when scanning for different sized stranded cetaceans (or equivalent sized features), and estimated grid cell height and width for certain computer screen sizes, see supplemental S1, Table S1.1. The reference measurements can be used to create a grid in both QGIS and Python. In QGIS the ‘Create Grid’ tool can be used (see supplemental S1). In Python, the code is designed to extract the satellite image bounds, and using user defined height and width measurements from Table S1.1 (supplemental S1), incrementally create polygons across the image extent to the specified size. The resulting geopandas dataframe of the coordinates for each polygon is converted into a shapefile (grid), to overlay on the respective satellite image.

#### Step 4: Annotation

Point shapefiles are useful for denoting the location of a feature of interest, detailing attributes (storing information/metadata of a feature), and for extracting spectral information of a given pixel below a point in a raster layer. As a minimum, our workflows enable the export of geolocated (longitude and latitude) point data in .csv format. Geolocated point data in .csv format was considered the least heavy data type for data storage and sharing, and also the most flexible for onward users to utilise and transform into other formats and data types. For example, continuation of the workflow enables a user to convert point data to bounding boxes, to create image chips, like those published by Cubaynes and Fretwell [56], which are suitable for training machine learning models for detecting occurrence of a feature.

Along with the open-source workflows we provide a standardised list of attributes (required and desirable) for the satellites to study cetacean strandings community, to enable international data sharing and collaboration (see section 3 ‘Data template and training document’ and supplemental S3).

QGIS operates similarly to ArcGIS with a geographical user interface designed for geospatial data. Python Jupyter Notebook requires the development of a custom tool to interactively map and annotate satellite imagery. The Python pipeline established here draws upon Leafmap [57], a Python package designed to enable interactive mapping and analysis of geospatial imagery and data. Custom ipywidgets have then been designed to enable near-full interaction of the tool using the geographic user interface rather than code. These custom controls include, manipulation of satellite image stretch (adjust image brightness and colour for improved visualization of an image), searching for annotation by ID or attribute, custom drop-down mapping of attributes, viewing a summary of annotations, edit and delete functionality of annotations, and exporting of annotations.

#### Step 5: Creating bounding boxes

For several automation methods, training data is expected in the form of image chips (red, green, blue (RGB) 8-bit .png/.jpeg/.tif format) [56]. However, most models are not designed to receive geolocated multispectral data [58] and need converting to RGB 8-bit, though developments are being made through packages like TorchGeo [50,60]). A point shapefile can be used to create bounding boxes around a feature, to later extract image chips and create training data for deep learning algorithms. The size of the bounding box and resulting image chip (effectively a grid of pixels) are important for certain model architectures. Some machine learning algorithms like Convolutional Neural Networks (which use pooling layers, repeatedly halving the input image size), operate optimally when incoming training data is a power of two (*e.g.,* 64, 128, 256), over several iterations. To create image chips in QGIS of a known dimensionality (number of pixels), which are a power of two, requires defining distance based on metres not pixel number, whereas in Python pixel distance can be used. For achieving equal sized bounding boxes in QGIS, where the feature is centrally located and full pixels preserved, points must initially be placed central to a pixel. In Python the code intuitively refers to the whole pixel containing the point, iteratively counting outward to the specified size of the clipping window, preserving whole pixel alignment. Therefore, in QGIS, as point placement is dependent on annotator precision, placement needs to be corrected, see Cubaynes et al., (2023) guidance [42]. The first step is to rasterise the point shapefile to create a raster pixel (QGIS ‘Rasterization’ tool), re-convert the pixel back into a point shapefile (QGIS ‘Raster pixels to points’ tool), which centralises the point within a pixel. After joining the attribute table from the original shapefile (QGIS ‘Join attributes by nearest’), the centralised point can be used to create a buffer (QGIS ‘Buffer’ tool) of a given distance. Distance in metres equal to the number of pixels can be inferred by using the known pixel size of an image. For example, to create a 128 × 128 pixel output image chip for an image with a 0.5 m spatial resolution or pixel size, the distance would be 128 × 0.5 = 64 m. The QGIS buffer tool calculates distance from the point, and expects half the height of the desired bounding box, not the full height, *e.g.*, 64/2 = 32 m. See supplemental S1, Table S1.2, for example distances for a number of different desired output chip sizes that can be used with the QGIS buffer creation tool. In Python, the desired final chip output size can be defined as n = total pixels, for example n = 128 would result in a 128 × 128 (pixel) image chip. For each point, map coordinates (longitude, latitude) are converted to pixel coordinates (x, y) a resulting clipping window equal to n x n is generated, with the pixel containing the point annotation central to the window. The clipping window is used to clip a raster to create an image chip, which is written out using Rasterio as a .png or .jpeg format.

#### Step 6: Creating image chips

Bounding boxes created using the method detailed in this process flow, can be used to generate image chips for use as training data in machine learning models. Building large enough datasets for machine learning, will require an internationally collaborative effort. Given the nature of VHR satellite data, digital datasets, in principle, can easily be shared, subject to licencing agreements. The open-source database by Cubaynes and Fretwell [56] demonstrates the possibility for data sharing (published image chips, as .png format) and achieving a global dataset. Each end user should check with the satellite image provider and the end user licence agreement before sharing datasets.

### Data template and training document

Data sharing is a crucial step for inclusivity and to develop a global dataset [39]. A global dataset unites researchers, NGOs and governments from well-resourced and less resourced countries, and can diminish the power imbalances of colonial legacy [39]. The sharing of open-source pre-processing and satellite image annotation workflows is integral to develop large open-source standardised training datasets for automation and for semi-automation/data validation [40]. This also enables the community to work in alignment with FAIR principles to make data, Findable, Accessible, Interoperable, and Re-useable [61]. Machine learning outputs are only as good as the data that are used to train them. Therefore, in the early stages of using VHR satellite imagery to study stranded cetaceans, the field is in a unique position, to develop robust and standardised annotation protocols that achieve the highest quality annotations from the outset and enable data sharing internationally. Further to the workflows shared here, we co-developed a template for recording annotation metadata (*e.g.*, confidence scores, satellite metadata, feature information, and environmental conditions) and an accompanying training document, informed by stranding and remote sensing experts (see Table 2, Table 3, supplemental S3 and S4, and the custom ipywidget attribute drop-down mapping tool in the Python code on GitHub at https://doi.org/10.5281/zenodo.20547057 [32]). This approach ensures that future stranding datasets can be collected and formatted using consistent data standards, enabling comparison across studies and reproducibility of experiments. In the future, when developing projects, budgets need to consider costs to achieve high quality annotations.

**Table 2:** List of required attributes associated with each strandings from space annotation. Most attribute values are standardised drop-down menus with exception of ‘id’, ‘observer’, ‘location’, ‘img_cat_id’, ‘img_date’, ‘img_time’, these are user defined, and ‘longitude’ and ‘latitude’, which are calculated post-annotation.

Attribute	Description	Unit	Format	Value
id	A unique numerical value assigned to each annotation (marked point).	None	Integer	*e.g., 0, 1, 2, 3…*
observer	Name of person reviewing the image (user to define the names of each person in the team of observers for a given project. Numerical values can be assigned to preserve observer anonymity).	None	String	*e.g.,* penny_clarke *or 1*
location	Name of the location where the satellite image was captured (user to define, use lower case and ‘_’ instead of space for compatibility with coding languages)	None	String	*e.g.,* golfo_de_penas
satellite	Name of the satellite that captured the image (‘satellite’ is a list of commercial VHR satellites (specifically those, which are internationally accessible), including those decommissioned, currently in orbit, or projected for launch in the near future).	None	String	clarity_1 geoeye_1 pelican pleiades pleiades_neo quickbird skysat worldview_1 worldview_2 worldview_3 worldview_4 worldview_legion
img_cat_id	Unique identification that the satellite imagery provider assigns to each image. With Vantor, this corresponds to the catalog ID.	None	String	*e.g.,* 10400ED2959
img_date	Date the image was captured (UTC).	Year, month, day (ISO8601 format)	String	YYYY-MM-DD
img_time	Time the image was captured (UTC).	Hours and minutes (ISO8601 format)	String	hh:mm:ssZ
gsd_m	The ground sampling distance (is the distance on the ground represented by a single pixel in a satellite image), which in QGIS can be found in the metadata, by right clicking on the panchromatic file and selecting ‘Properties’, then ‘Information’ and ‘Pixel Size’ or else by viewing the .imd or .xml file.	meter	Double	0.1 0.15 0.2 0.3 0.31 0.4 0.46 0.5 0.6 0.65 0.7 0.75 0.8 0.9 1
prod_type	The product type indicates the level of pre-processing applied to an image. ‘prod_type’ is a list of commercial VHR satellites operators, specifically those, which are internationally accessible, and their available product types.	None	String	analysis_ready map-ready_ortho_1_12,000 map-ready_ortho_3d ortho photogrammetry_ready primary projected skysat_basic_scene skysat_ortho_collect skysat_ortho_scene system-ready_basic_1b system- stereo_basic_1b view_ready view-ready_standard_2a view-ready_standard_or2a view-ready_stereo_standard_or2a
projection	Projection applied to the image to remove distortion (Universal Transverse Mercator (UTM), is a series of grid zones of the world, a plane coordinate grid and can be determined for an image using https://www.dmap.co.uk/utmworld.htm.	None	String	*e.g.*, epsg_32,718_wgs84_utm_zone_18s
pansharpen	The algorithm used to perform pansharpening of an image (format includes available algorithms in QGIS OrfeoToolbox (OTB) and GDAL. For example, OTB algorithms are either ‘rcs’, ‘lmvm’, or ‘bayes’, and interpolation is either ‘bco’, ‘nn’ or ‘linear’) (user defined alternatives can be added to value mapping where necessary).	None	String	gdal_average gdal_bilinear gdal_cubic gdal_cubic_spline gdal_lanczos_windowed_sinc gdal_nearest_neighbour otb_bayes_bco otb_bayes_linear otb_bayes_nn otb_lmvm_bco otb_lmvm_linear otb_lmvm_nn otb_rcs_bco otb_rcs_linear otb_rcs_nn
bands	The combination of bands used to visualise an image (spectral bands are the ranges of wavelengths of light that are captured by a sensor, assigned to bands). Combinations of bands can alter image visualisation. To visualise as the eye would expect, typical band combinations for Vantor imagery are R = 3, G = 2, B = 1 (four band image) and R = 5, G = 3, B = 2 (eight band image). The band order in the ‘Value’ column is RGB. RGB refers to the QGIS display settings, while the number refers to the specific band of the multispectral sensor.	None	String	1,2,3 3,2,1 4,3,1 4,3,2 5,3,2 6,4,3
feature	The type of feature a detection is. This includes the ability to annotate confounding features, which can be useful in creating multi-class training datasets (see definitions below).	None	String	stranded_cetacean floating_carcass live_cetacean boat marine_litter organic_beach_debris rock
certainty	Certainty of an observer that a detection is the assigned feature (species or higher taxonomic level) Definite: 90–100% confidence a detection is the defined feature (almost no doubt) Likely: 70–89% confidence a detection is the defined feature (high likelihood but not sure, for example, when in a mass it is challenging to determine individuals, however based on morphology and size you assign as two features, but you are not sure) Possible: 50–69% confidence a detection is the defined feature (hard to tell, perhaps because there is marine debris on the beach with a similar appearance or an individual is far from the mass but has a similar appearance).	None	String	definite_90–100 likely_70–89 possible_50–69
longitude	Longitude of the ‘stranded_cetacean’ or ‘floating_carcass’.	Decimal degree	Double	*e.g.,* 12.70
latitude	Latitude of the ‘stranded_cetacean’ or ‘floating_carcass’.	Decimal degree	Double	*e.g.,* 67.50

**Table 3:** List of optional attributes associated with each strandings from space annotation. Most attribute values are standardised drop-down menus with exception of ‘length_m’, ‘width_m’, and ‘comments’, these are user defined. Details of environmental variables represent the conditions at the point feature/annotation specifically, and not the whole image.

Attribute	Description	Unit	Format	Value
deco_phase	The level of decomposition of a ‘stranded_cetacean’ or ‘floating_carcass’ (see definitions supplemental S3).	None	String	fresh_alive fresh_dead bloating_and_floating active_decomposition passive_decomposition dry_remains unknown
species	The species or higher taxonomic level of the feature detection (*e.g.,* ‘sei_whale’), else when unknown assign higher taxonomic level (*e.g.,* ‘large_whale’ or ‘unidentified_whale’). The list of species uses the Society of Marine Mammalogy (SMM) [62] naming conventions, to ensure consistent terminology throughout the marine mammal community, with addition of instances where species is unknown.	None	String	*e.g.,* unidentified_dolphin unidentified_large_baleen_whale unidentified_large_whale unidentified_porpoise unidentified_small_whale unidentified_whale (… remaining list is the SMM naming conventions for all species *e.g.,* bowhead_whale)
colour	The most dominant colour of the detected cetacean. Colour should be assigned when the feature is viewed in true colour (red, green, blue bands). During decomposition the ‘stranded_cetacean’ or ‘floating_carcass’ colour may be a mixture of colours, assign the most dominant by percentage cover.	None	String	black brown cream dark_grey light_grey orange pink red white
body_shape	Overall shape of the body excluding fluke and flippers. Select the most suited or ‘undefinable’, especially in late stages of decomposition.	None	String	ellipsoid streamline irregular_ellipsoid irregular_streamline undefinable
length_m	Maximum visible length between the tip of the head and the fluke with values ranging from calf size to maximum adult length.	Meters	Double	*e.g.,* 12.4
width_m	Maximum visible width measured at the widest part of the body and perpendicular to the body length.	Meters	Double	*e.g.,* 2.0
flippers	Are the flippers (forelimb used to turn, break and stabilise a cetacean while swimming) visible?	None	String	yes no maybe
fluke	Is the fluke (tail used to propel a cetacean forward) visible?	None	String	yes no maybe
slick_bld	Is a slick or blood visible? A slick is a waxy grey liquid released when a cetacean decomposes (the oil contained within cetacean blubber gets converted to adiopocere, a wax like substance formed when body lipid-tissues break down). It can cover wide areas around the remains. This can temporarily prohibit the growth of nearby plants, so reduced or dead vegetation nearby and grey colouration surrounding a carcass may be seen from space.	None	String	yes no maybe
group	The number of ‘stranded_cetacean’ or ‘floating_carcass’ within 1 km of a detected feature. This should be per annotation and can be an estimate, especially when in a mass.	None	String	individual 2_to_5 6_to_10 11_to_50 51_to_100 101_to_200 201_to_500 501+
cloud_cov	Cloud cover for the area within the vicinity of the detection, using the aviation system: ‘skc_sky_clear’ = 0/8th ‘few_traces’ = 1–2/8th ‘sct_scattered’ = 3–4/8th ‘bkn_broken’ = 5–7/8th ‘ovc_overcast’ = 8/8th	Okta	String	skc_sky_clear few_traces sct_scattered bkn_broken ovc_overcast
cloud_thic	Cloud thickness for the clouds present within the vicinity of the detection: Thin (can see fairly well through the clouds) Medium thin (can see through but no clear view of the sea) Thick (cannot see through)	None	String	none thin medium_thin thick
env	The abiotic environment that a detection is found in (nearshore waters are < 1 km and offshore waters are > 1 km from shore).	None	String	bedrock cobble_beach estuary_mud_flat gravel_beach man_made_structure mangrove marsh_brackish marsh_freshwater nearshore_waters offshore_waters sand_beach_dark sand_beach_light shell_beach vegetated_trees vegetated_wetland
img_bright	The brightness of the image within the vicinity of the detection.	None	String	bright moderate dark
shade	The level of shade from the sun, full, partial or none at the point of the detection.	None	String	full_shade partial_shade no_shade
ice_cover	Relevant to ice covered regions only, the level of ice cover at the point of the detection.	None	String	none low medium dense very-dense
comments	Any other comment the observer would like to make about the specific detection.	None	String	*e.g.,* Could be dry remains of carcass or wood from a tree

## Method validation

The standardised protocol for QGIS and Python presented here, was refined based on feedback from remote sensing and cetacean experts. The template for recording annotation metadata and an accompanying training document was reviewed by stranding experts and the international ‘Using Satellites to Study Whales’ community during a best practice workshop at the Society of Marine Mammalogy Conference 2024 (‘Satellites to Study Live and Stranded Cetaceans: Establishing best practices’ workshop, 10 November 2024).

The QGIS workflow was used by Clarke et al. [35] in a multi-observer experiment, to detail the smallest stranded cetacean detected and quantified in VHR optical and SAR satellite imagery, and the minimum spatial resolution optical imagery needed to count with confidence. The associated data validating the workflows application is available to download at the British Antarctic Survey Polar Data Centre (PDC) https://doi.org/10.5285/b26c3a3d-73c8-4500-9a23-696011c20a45 [65]. The Python pipeline is a replicable version of the QGIS workflow and Cubaynes et al., [42] methodology, which demonstrate its validity.

## Limitations

The workflows presented here form a valuable contribution towards increasing accessibility in the field of using satellites to study stranded cetaceans, and more broadly, the remote sensing community. However, several challenges remain to achieving equality of access and use, such as the cost of access to commercial satellite imagery and the resources required to process, annotate, and store VHR image data.

### Pre-processing

The latest releases of Orfeo Toolbox (OTB) for Python are not compatible with Anaconda installation for Microsoft, so a work around is offered here using a historic version of OTB. In addition, we provide alternative tools to pansharpen for example, GDAL. With newer releases of OTB, this challenge may be resolved.

### Annotation

Here we recommend a data standard as best practice to facilitate international collaboration and reproducibility, however, we recognise this may be restrictive for the needs and priorities of local communities. Given the intent of the workflows presented here are to empower locally driven monitoring efforts and to combat parachute science, the annotation template has been designed to allow customisation and enable users to remove and add/propose new standards.

The option to convert point data to bounding box and image chips of a known dimensionality, presented here, provide the simplest form of training data for machine learning inputs, and they do not provide exact outlines of a stranded cetacean/feature of interest. By introducing large areas of environment around a feature of interest within a bounding box, machine learning models can perform with inaccuracies. To answer more advanced questions, particularly for whale and dolphin features, where often features will appear on diagonal lines, unsuitable for rectangular/square bounding boxes, and to gain individual mask outputs for downstream analytics, polygons are a better approach. Zheng et al. [63] have demonstrated how point data can be successfully converted to precise bounding box/polygons using Segment Anything. Combining our image annotation workflow to produce geolocated point data with Zheng et al. [63] workflow to convert point data to precise bounding box/polygons, could form a robust dual workflow to generate precise training data for machine learning.

The custom Python annotation tool shared here is dependent upon external packages, Leafmap and Ipyleaflet. This raises several common concerns in Python software development such as, package dependencies and version compatibility, and package deprecation [64]. Python packages are vulnerable to poor maintenance due to, predominantly volunteer led efforts, changes in personnel, diminishing personal interest, and time constraints [64]. To mitigate these risks to the functionality of our workflow, we selected packages that are well-established, widely used, and actively managed. We list existing known package versions for environment creation that assure a reliable and operable pipeline. In addition, we will review and adapt to recommended solutions raised by deprecation mechanisms, and be proactive at troubleshooting issues through the GitHub repository. There is a known bug with Leafmap, where using and exiting full screen mode using the ‘View Fullscreen’ button works only once. Once the full screen mode is exited after first use, the user will not be able to re-enter full screen mode without re-running the cell to create the map from fresh. To overcome this challenge, all custom buttons and tools needed to annotate have been designed to fit into the window extent of the map when not in full extent mode. The map can be interactively zoomed using a mouse scroll or the zoom control buttons, which should ensure functionality without using full screen mode.

## CRediT author statement

Conceptualisation: P.J.C. Funding acquisition: P.J.C., J.A.J. Methodology: P.J.C., J.A.J., H.C.C., E.B., K.A.S., A.S., K.M.M. Project administration: P.J.C. Software: P.J.C., J.A.J., H.C.C., E.B., M.R.G.A., A.S., E.M-L., S.L.D. Supervision: J.A.J., H.C.C., K.A.S., P.T.F., A.d.V., E.M-L. Writing – original draft: P.J.C. Writing – review and editing: P.J.C., J.A.J., H.C.C., K.A.S., E.B., K.C., M.R.G.A., P.T.F., A.S., A.d.V., K.M.M., E.M-L., S.L.D.

## Ethics statements

The method presented here does not involve work with human subjects, animal experiments, or data collected from social media platforms.

## Declaration of competing interest

The authors declare that they have no known competing financial interests or personal relationships that could have appeared to influence the work reported in this paper.

## Data Availability

A link to the code and dataset demonstrating the methods validity are available in the manuscript.

## Appendix Supplementary materials

Image, application 1

Image, application 2

Image, application 3

Image, application 4

Supplemental S1 provides a step-by-step guide for satellite image pre-processing and annotation using QGIS 3.28.

Supplemental S2 is a short Python script for outputting image chips as .png/.jpeg format, intended for use with the PyQGIS console.

Supplemental S3 is a training guide (descriptive and visual) to the standardised annotation attributes for identifying stranded cetaceans in satellite imagery.

Supplemental S4, is a template shapefile containing the standardised annotation attributes for identifying stranded cetaceans in satellite imagery. The most up to date version of all supplemental material is available at: https://github.com/PennyJClarke/strandings_from_space.

## Footnotes

1 ukintelligence-imagerysupport@airbus.com

2 https://oneatlas.airbus.com/archive-search-tool/description

3 https://community.planet.com/

4 support@planet.com

5 orders@vantor.com

6 https://xpress.maxar.com/

7 https://space-solutions.airbus.com/imagery/how-to-order-imagery-and-data/

8 https://pro.maxar.com/
