# Supplementary material for: Open-source satellite image pre-processing and annotation workflows: Stranded whale and dolphin case study
Source: MethodsX. 2026 May 14;17:103949. doi: 10.1016/j.mex.2026.103949 (PMC13586820; doi:10.1016/j.mex.2026.103949)
Supplement: Supplementary file 1 [file mmc1.pdf]

S1: QGIS STRANDINGS FROM SPACE PIPELINE

CONTENTS

Introduction 1

Glossary 2

Step-by-step workflow QGIS 3.28.1 3

Setting up 3

Software installation 3

Opening and saving a QGIS project 3

Importing images 6

Pre-processing 11

Projecting non-projected images 11

Setting up Orfeo Toolbox and Pansharpening 18

Systematic scanning 45

Grid scale and Scale to scan 45

Creating a grid 49

Systematically scan imagery 54

Annotating imagery with point shapefiles 62

Creating bounding boxes 81

Creating image chips 93

Appendix 100

appendix 1: Metadata: Access and finding information 100

appendix 2: creating a Template Shapefile 104

INTRODUCTION

The Strandings from Space pipeline is an open-source workflow, to manually annotate stranded cetaceans in very high-resolution (VHR) satellite imagery (see glossary). The workflow draws upon recommendations from experts in the field of wildlife from space. The step-by-step guide builds upon the work of Cubaynes *et al.* (2023), to form the first published guidance for image annotation of VHR satellite imagery for stranded cetaceans, that uses openly accessible software. The workflow includes the first attempt at standardising data annotation methods and terminology for cetacean strandings monitoring using satellite imagery.

By publishing a step-by-step guide that is openly accessible, standardised, and draws upon expert recommendations, this pipeline will help foster internationally collaborative efforts to monitor cetacean strandings, and enable the generation of large datasets required for automated detection to make this tool scalable. The workflow aims to help stranding networks worldwide to work towards achieving their long-term monitoring and conservation goals. The workflow and customisable annotation template is applicable beyond the strandings community, as it forms a framework supporting remote sensing

applications, which require image annotation. Particularly for projects requiring training data for automated detection processes using machine learning (see glossary), regardless of the target feature.

GLOSSARY

The following glossary is reproduced from (Clarke *et al.*, 2021) for further useful terminology see (Khan *et al.*, 2023):

Stranding Terms:

Mass Mortality Event (MME) – is when cetaceans strand either deceased or dying, over extended temporal scales (days to weeks) (Moore, Simeone and Brownell Jr., 2018).

Mass Stranding Event (MSE) – is when two or more individuals of the same species, not including mother calf pairs, simultaneously strand alive in the same location (Geraci and Loundsbury, 2005; Moore, Simeone and Brownell Jr., 2018).

Stranding – is a cetacean that falters ashore, debilitated, or is in an environment incompatible with its natural survival; if the cessation of life has occurred prior to deposition, the cetacean is considered beachcast (Geraci and Loundsbury, 2005).

Unusual Mortality Event (UME) – is a stranding event that is considered unexpected for; a given area or time of year for the species in question; a different age or sex assemblage for an area or time of year; the size of an event; an increase in the frequency of an event when compared with previous years; cetaceans exhibiting unusual pathology or behaviour or for the stranding of critically endangered cetacean species. An UME event comprises a substantial number of dead individuals of any cetacean population and necessitates immediate action (Geraci and Loundsbury, 2005; Mazzariol *et al.*, 2020).

Remote Sensing Terms:

Machine Learning – is the use of computational power to apply a diversity of data analysis approaches and algorithms to existing data/information, to learn from, and then accurately predict (Humphries, Magness and Huettmann, 2018).

Panchromatic and Multispectral imagery – is electromagnetic radiation with emission properties in the visible electromagnetic spectrum that produces greyscale imagery (panchromatic) and colour imagery (multispectral) (Dowman *et al.*, 2012; Rees, 2013).

Remote sensing – also known as “Earth Observation,” is the principle of deriving information about the earth’s surface using a remote device, acquired using a number of electromagnetic radiation sensors such as: microwave, radar, thermal, infrared, ultraviolet and multispectral (Campbell and Wynne, 2011; Rees, 2013).

Spatial Resolution/Ground Sampling Distance – the length of ground represented by a single pixel, which in turn determines the level of detail visible (Liang and Wang, 2019).

Spectral Resolution – is the number of radiometric sensors aboard a satellite, that are receptive to different ranges of wavelengths (spectral bands) of incoming electromagnetic radiation. These include the panchromatic band, the visible light spectrum, and infrared bands (Rees, 2013; Liang and Wang, 2019).

Swath Width – is the maximum width of the earth’s surface acquired in an image in one acquisition (Rees, 2013).

Tasking – the ability of a satellite image provider to directly send information to a satellite, requesting when and where imagery be collected, and the satellites return transmission of imagery (Bayir, 2003).

Temporal Resolution – also known as the revisit rate, is the time between the acquisition of two subsequent images in the same location on the earth's surface (Liang and Wang, 2019).

Very High Resolution (VHR) – is the capability of satellite imagery to collect sub-metre spatial resolution images of the earth's surface (LaRue, Stapleton and Anderson, 2017; Cubaynes *et al.*, 2019).

STEP-BY-STEP WORKFLOW QGIS 3.28.1

SETTING UP

SOFTWARE INSTALLATION

The below guidance uses QGIS 3.28.1 on a Windows 10 operating system and draws upon a Vantor (formerly Maxar Technologies Ltd) WorldView-2 image (image catalogue ID 103001003F7A9900, satellite imagery © 2026 Vantor) to demonstrate the pre-processing and annotation steps. Please note, differences to the process flow may occur if using a different version of QGIS, a different operating system or satellite images from a different satellite sensor or image provider.

1. Downloading QGIS
    a. download QGIS, appropriate to your operating system from: https://www.qgis.org/download/ (historic versions can be downloaded from: https://download.qgis.org/downloads/)
    b. in your 'Downloads' folder, locate and double click the installer package to run

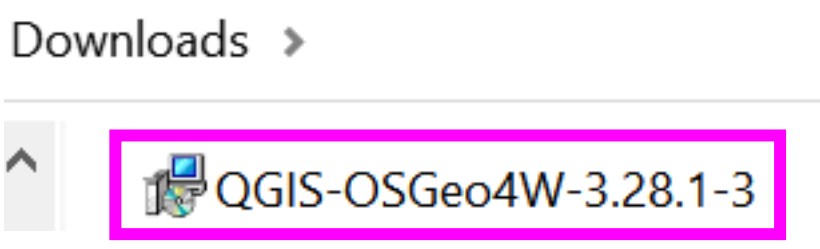

    c. Run the installation
        i. select 'Next'
        ii. tick checkbox to accept the terms of the Licence Agreement, 'Next'
        iii. select a destination folder where your download will install, 'Next'
        iv. run the installation by selecting 'Install'
        v. complete the installation by selecting 'Finish'

OPENING AND SAVING A QGIS PROJECT

2. Open QGIS (search QGIS from the start menu or double click the QGIS 3.28.1 icon to open)

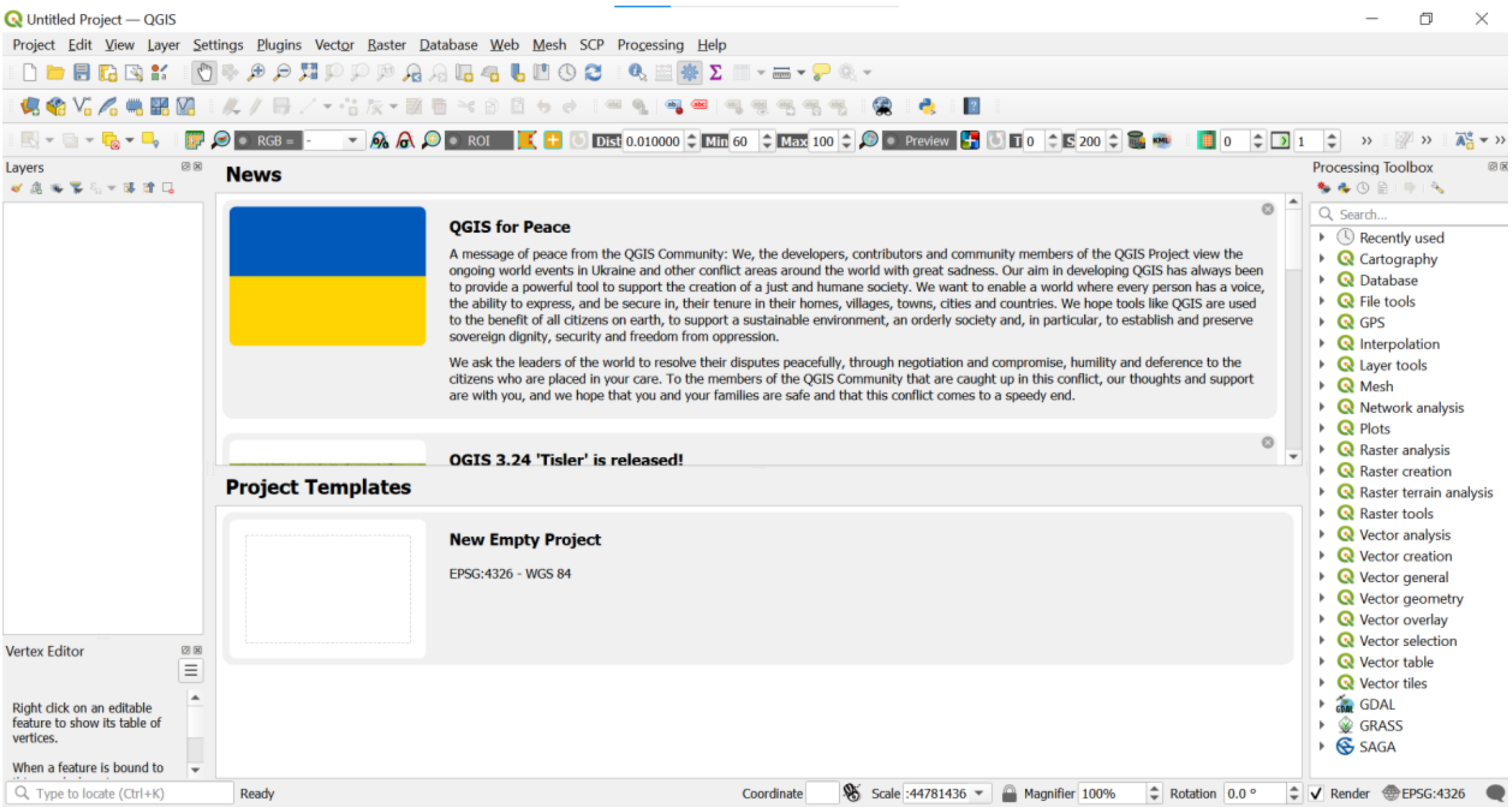

3. Saving your project
    a. from the ‘Project’ menu on the main toolbar, select ‘Save As’

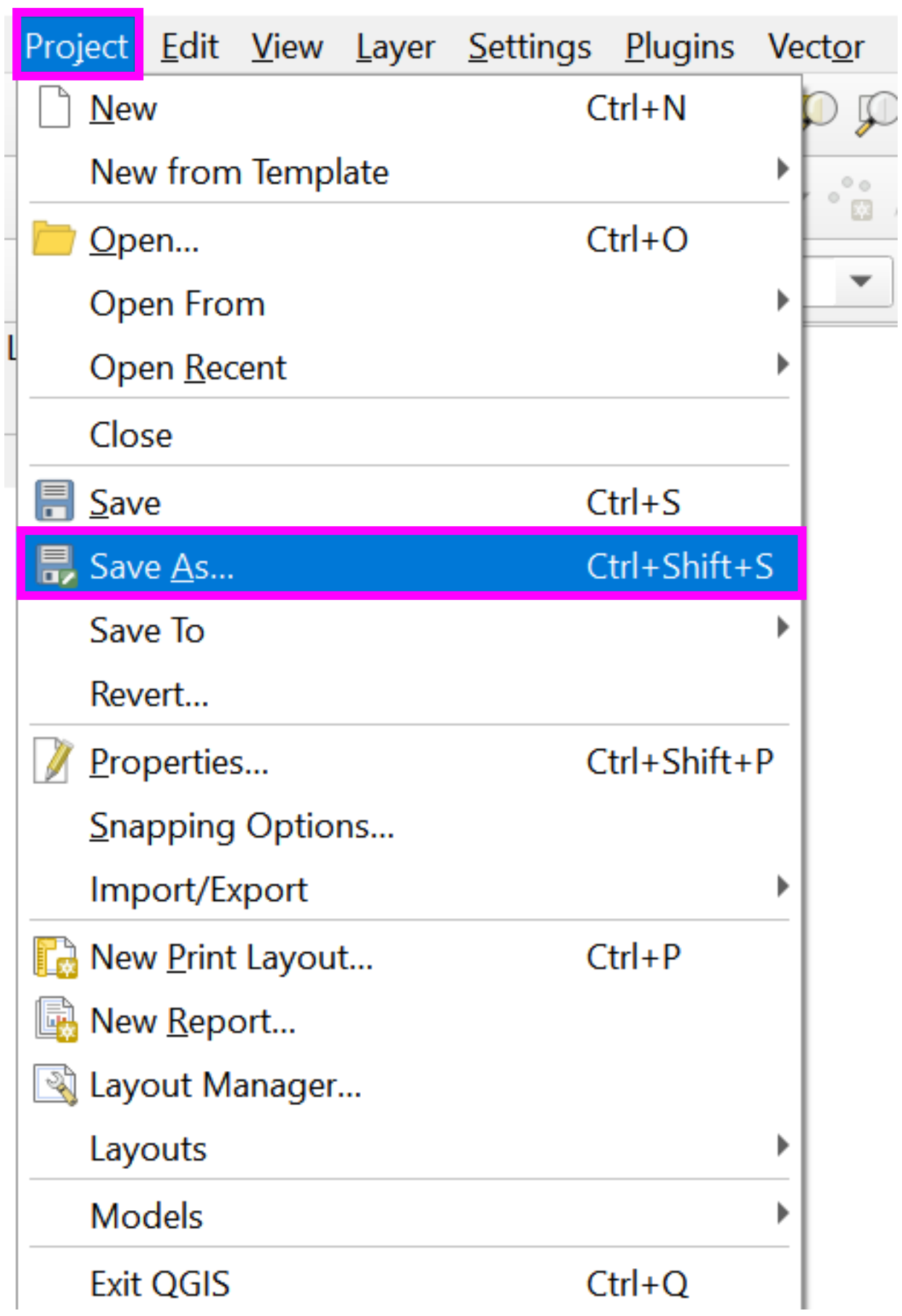

    b. locate the directory you wish to save your QGIS project to
    c. assign your project a name. **For the cetacean strandings community only, see the recommended naming convention, step 3.d, otherwise skip to step 3.e.**
    d. Recommended naming convention (cetacean strandings community only):

We encourage the cetacean strandings from space community use the following recommended naming convention for standardisation, data sharing and collaborative working.

i. location_satellite_image-catalog-id_image-date(YYYYMMDD)_image-time(HHMMSS)_spatial-resolution_observer-id
ii. example naming convention for the image used throughout this pipeline: 'golfo-de-penas-chile_wv2_103001003F7A9900_20240315_145159_50_1'

Use lower case only, where spaces are required use hyphens between words meant together (e.g., a location 'golfo-de-penas-chile' (if the image catalogue ID, assigned by the satellite company, contains underscores, and the underscores are amended to hyphens in the naming convention, state explicitly the correct image catalogue ID in any associated manuscript or metadata and in the attribute table), do add underscores between fields that are separate (e.g., image date and image time, '150324_145201'). This will allow greater compatibility in coding interfaces like Python.

iii. Locating information for the naming convention
- see Appendix 1 for information on how to access and locate information in the image metadata for the following parts of the naming convention: satellite, image-catalog-id, image-date, image-time, and spatial-resolution
- location (as descriptive a location as possible) and observer-id (name *e.g.*, penny_clarke or a number for anonymity *e.g.*, 1, is relevant to include if you wish to compare multiple observer observations) are both defined by the user

e. select 'Save'

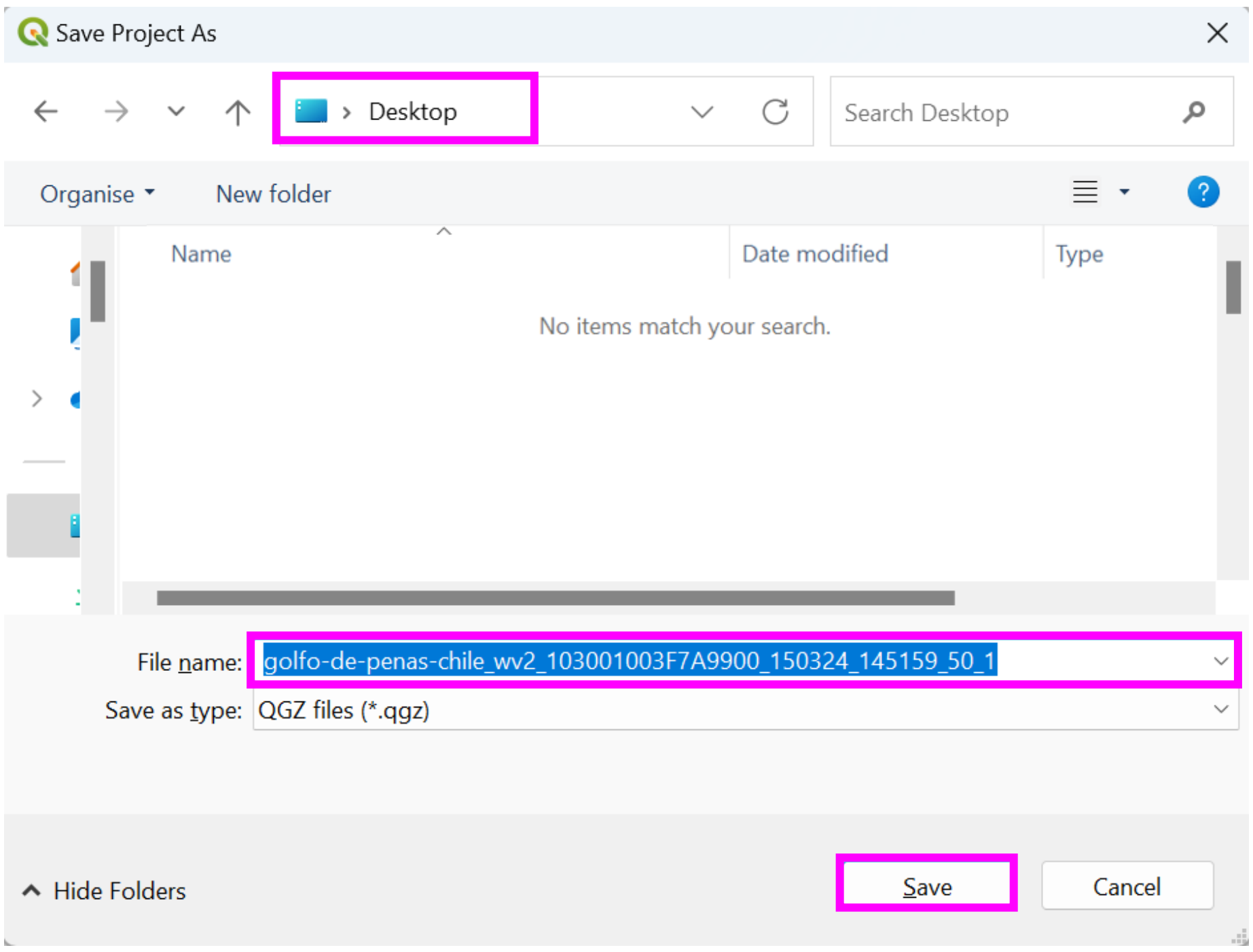

f. thereafter, the project can be saved using the save icon on the main toolbar. It is good practice to save the project regularly and after any edits are made

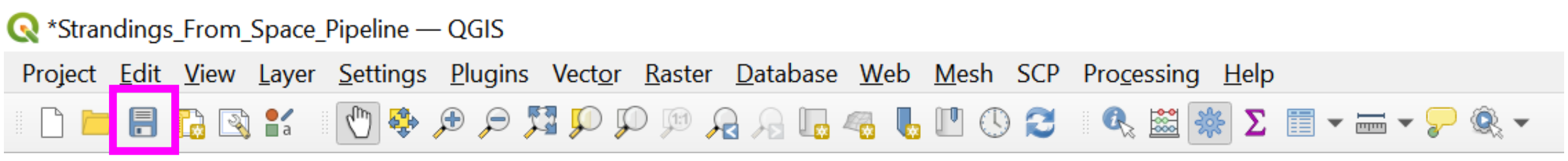

IMPORTING IMAGES

4. Identifying multispectral and panchromatic (see glossary) images (two methods: a, b)
    a. Naming conventions (multispectral ‘MUL’ or ‘M’ and panchromatic ‘PAN’ or ‘P’)

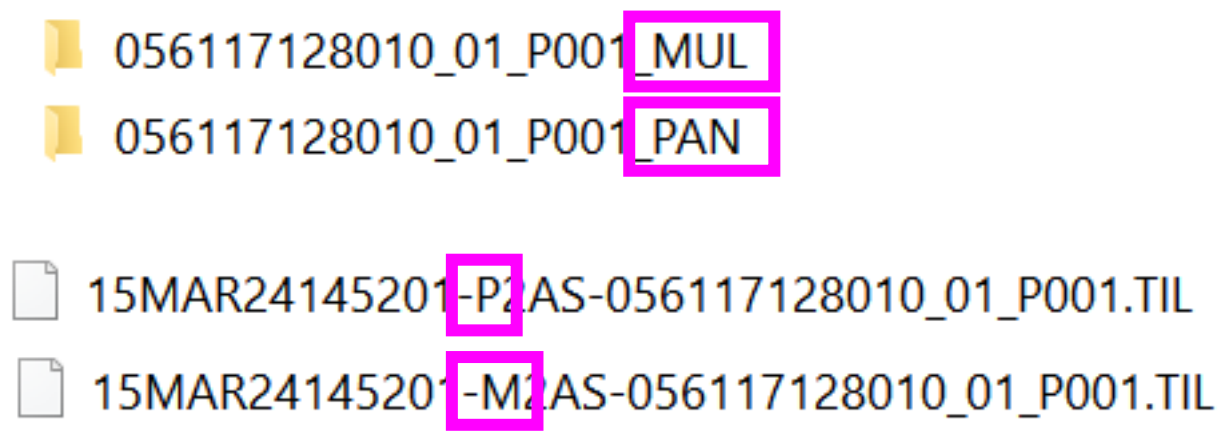

    b. File metadata
        i. see Appendix 1 for how to access and locate information in the image metadata
            - identify the image type from the BANDID or similar term, depending on the image provider (*e.g.,* Vantor BANDID is ‘Multi’ for multispectral and ‘P’ for panchromatic imagery)
            - further to image type, file metadata can help precise the number of bands of a multispectral image (spectral bands are the ranges of wavelengths of light that are captured by a sensor, assigned to bands). A panchromatic image is a single band *e.g.,* BAND_P. A multispectral image will have multiple bands. For example, an eight-band image will have eight bands listed (for example, Vantor’s WorldView-2 imagery: BAND_C, BAND_ B, BAND_G, BAND_Y, BAND_R, BAND_RE, BAND_N, BAND_N2), and a four-band image will have four bands listed (for example, Vantor’s WorldView-2 imagery: BAND_ B, BAND_G, BAND_R, BAND_N). Band numbers are in order the bands appear in the metadata file, *e.g.,* BAND_ B (blue) = 1, BAND_G (green) = 2, BAND_R (red) = 3, BAND_N (near-infrared) = 4, therefore, a true colour composite (an image visualised as the human eye expects), red, green, blue (RGB) = 3, 2, 1. This is important to assign bands for visualising a multispectral image, see section ‘Importing a multispectral image’.

5. Importing a panchromatic image

Panchromatic and multispectral image files must be imported separately into a QGIS project.

    a. from your local drive directory, identify, select, and drag and drop your panchromatic image (selecting the .TIL file for Vantor imagery) into the ‘Layers’ pane on the left of the QGIS window

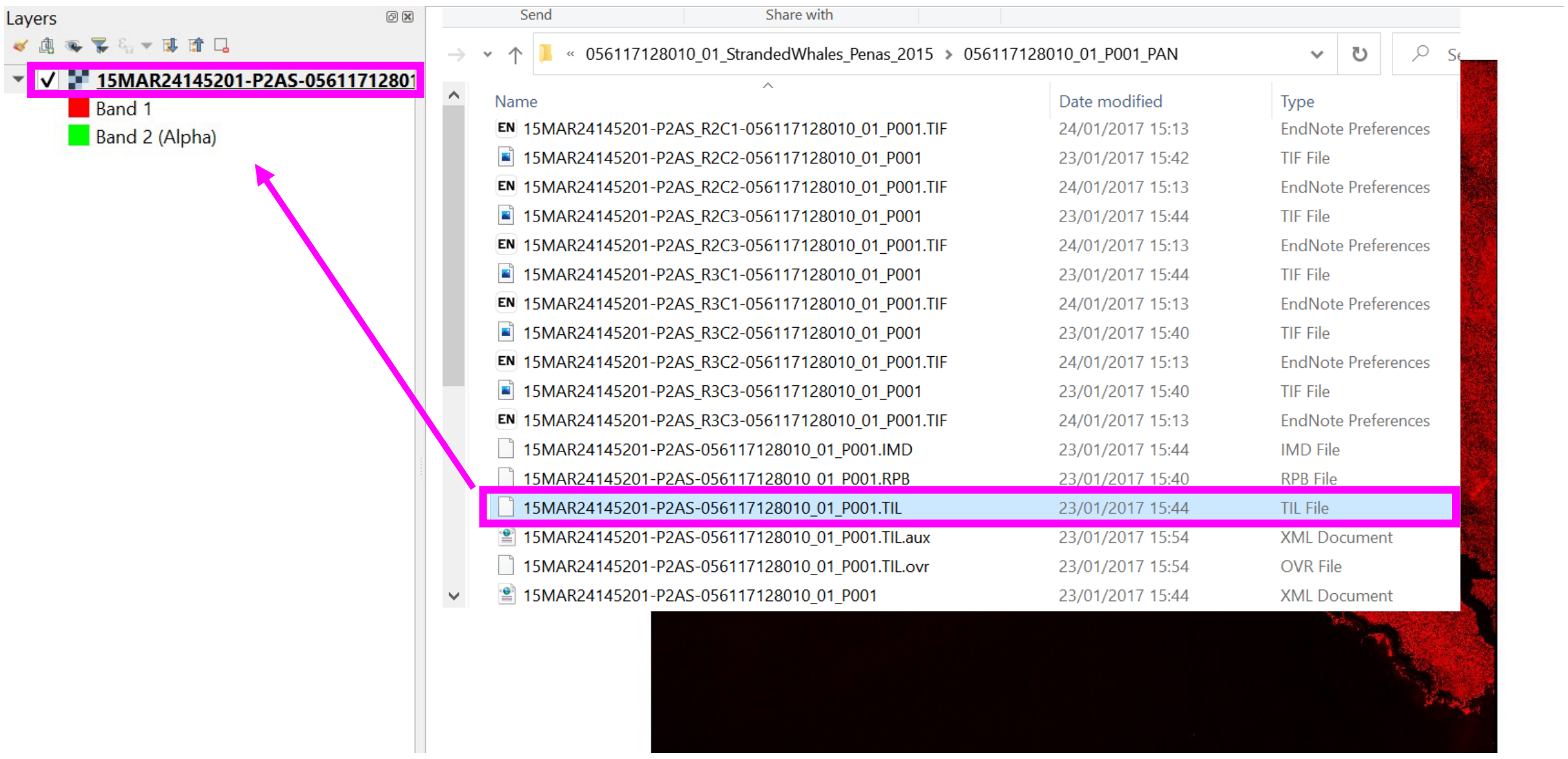

b. the imported image may be received as a multiband colour image (appear red and black), to amend the image to grey scale, right click the panchromatic image in the 'Layers' pane and select 'Properties'

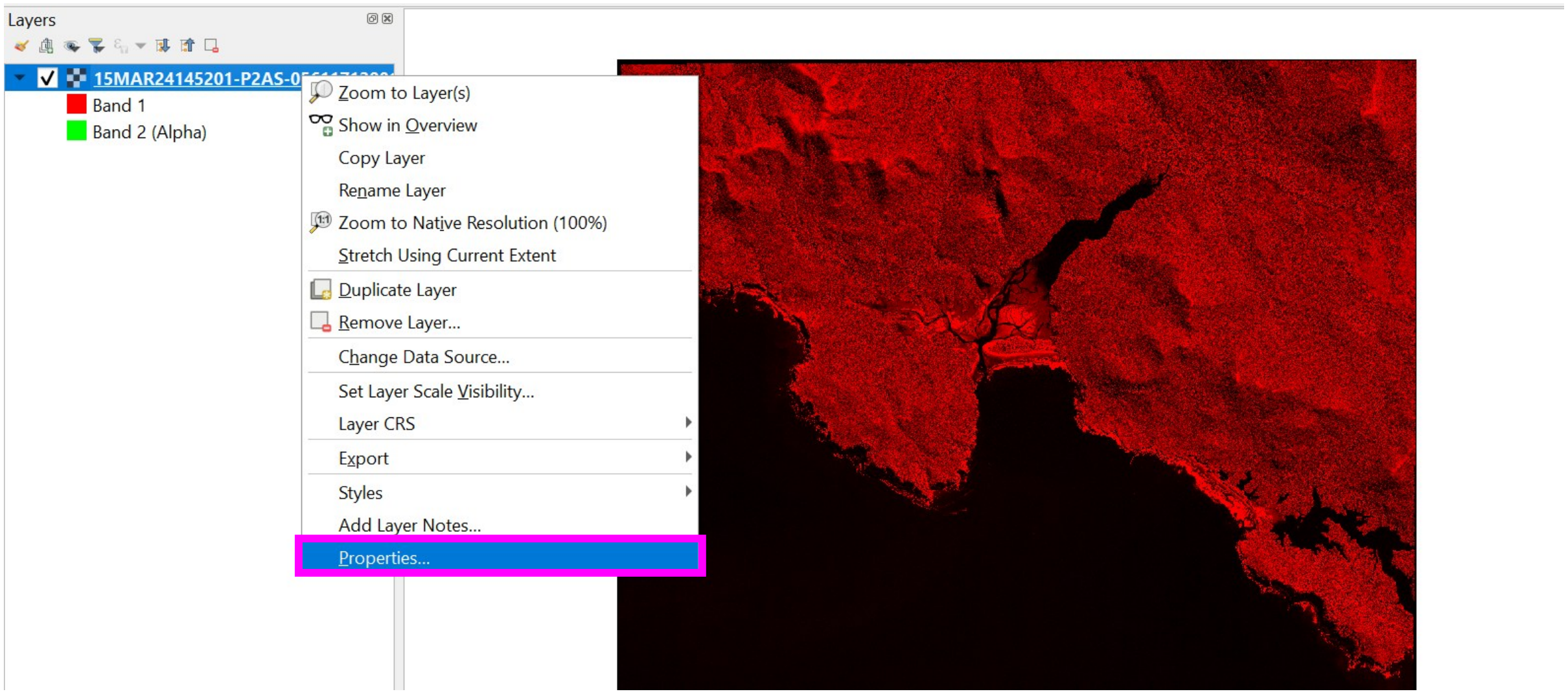

c. select the 'Symbology' tab
d. amend the 'Render type' to 'Singleband gray'
e. select 'Apply, and 'Ok'

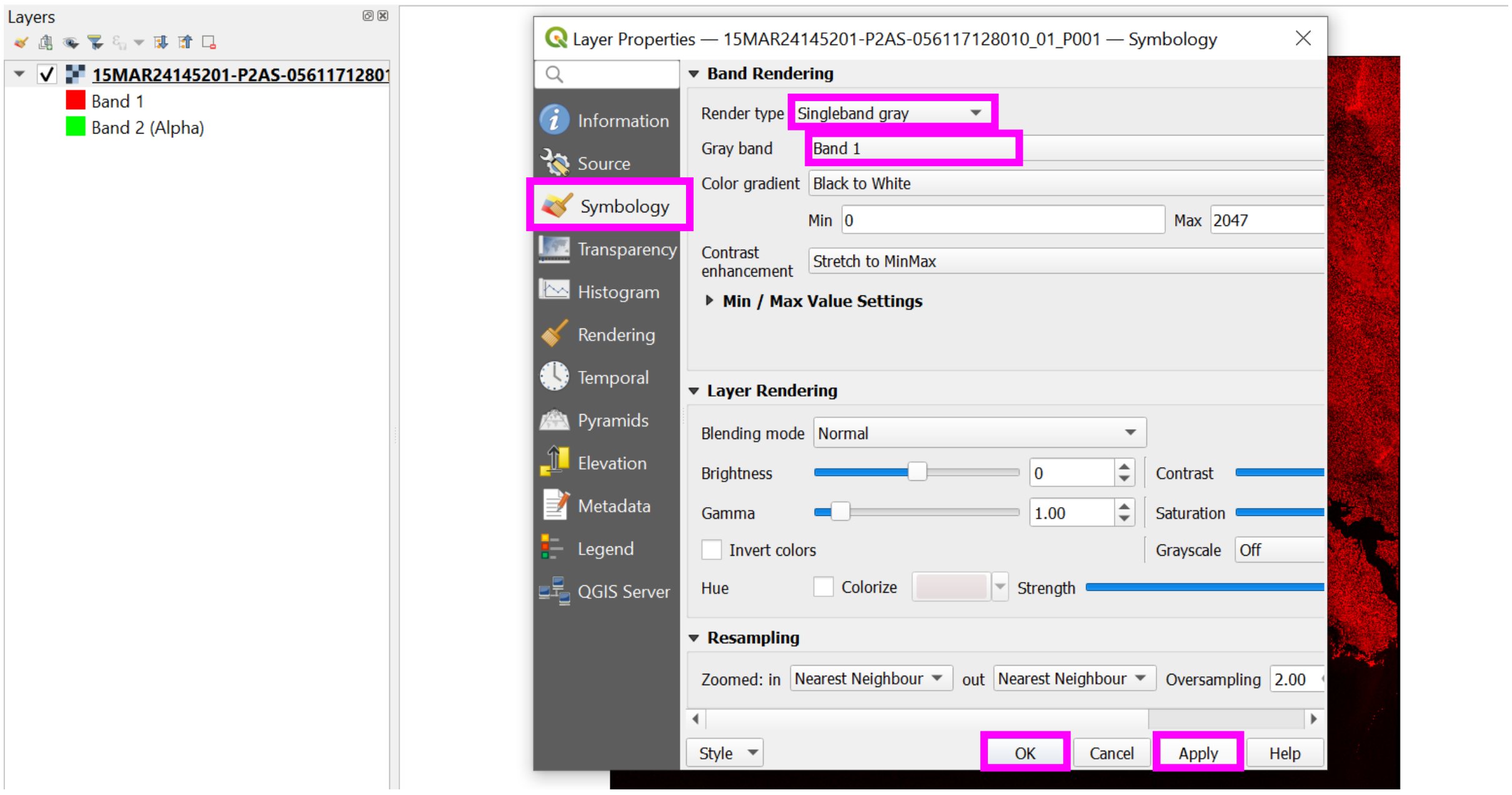

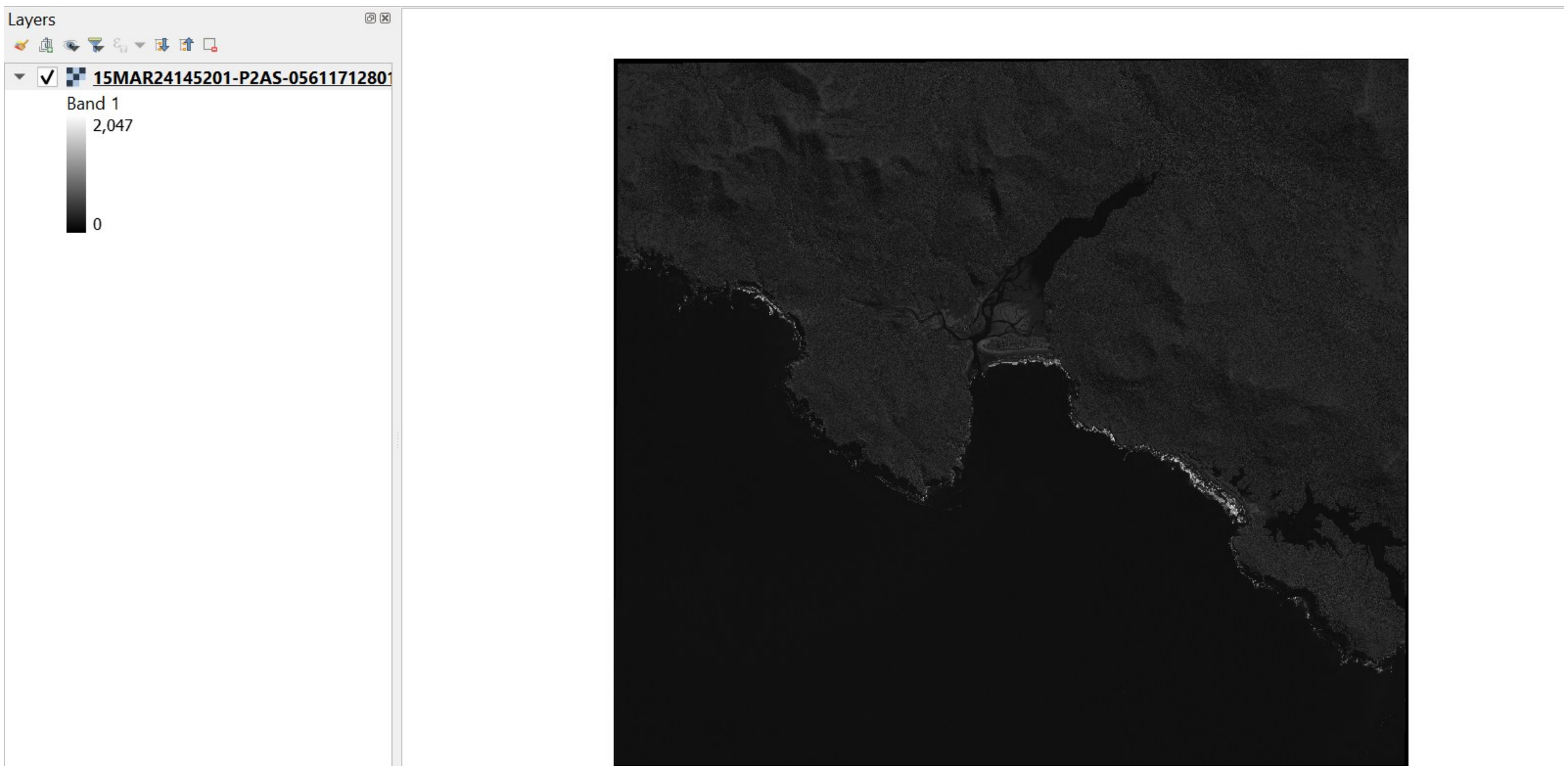

6. Importing a multispectral image
   a. from your local drive directory, identify, select, and drag and drop your multispectral image (selecting the .TIL file for Vantor imagery) into the ‘Layers’ pane on the left of the QGIS window, as per the panchromatic image
   b. amend the multispectral image symbology to visualise the image as per the natural environment (true colour). Right click the multispectral image in the ‘Layers’ pane and select ‘Properties’

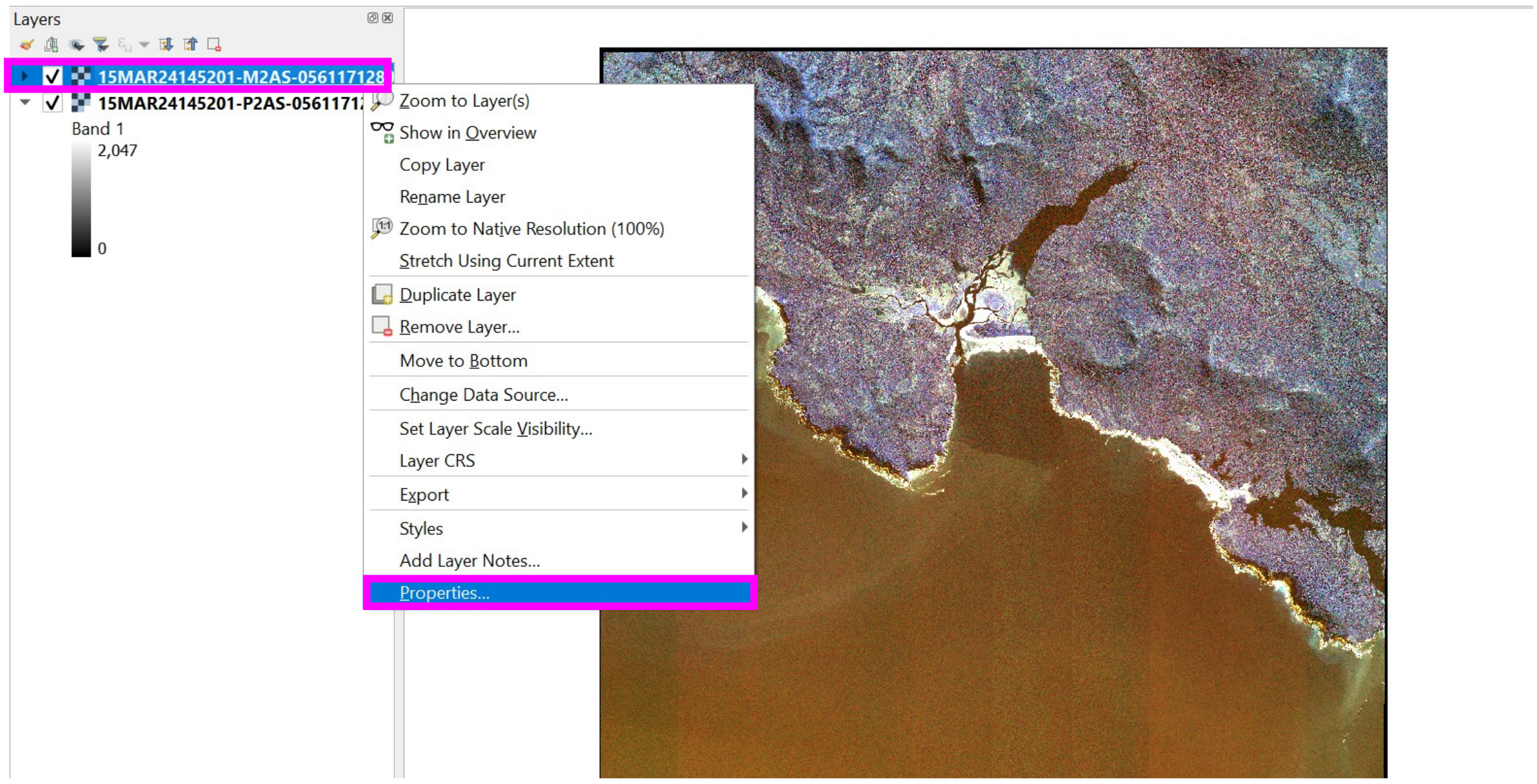

c. change the bands. Combinations of bands can alter an image visualisation. For example, to visualise as the human eye would see (true colour: red, green, blue), typical band combinations for Vantor's WorldView-2 four band imagery are: Red = 3, Green = 2 and Blue = 1 and eight band imagery: Red = 5, Green = 3 and Blue = 2. Here, RGB refers to the QGIS display settings, while the number refers to the specific band of the multispectral sensor. The bands to visualise multispectral imagery (which numeric band relates to each colour band for your satellite image sensor, see (Khan *et al.*, 2023), can be ascertained by reviewing the satellite sensor specification, either online from the satellite image provider or within the image metadata file (see Appendix 1, 'Metadata: Access and finding information'). This may be important to review if using a different satellite sensor or satellite image provider from the example used here.

   i. A false colour composite can help visualise the red-edge and near infrared ranges of the electromagnetic spectrum (band combinations R = 4, G = 3, B = 1 in four band imagery and R = 6, G = 4, B = 3 in eight band imagery, RGB refers to the QGIS display settings, while the number refers to the specific band of the multispectral sensor). These band combinations can be powerful for discriminating stranded cetaceans from confounding features (Figure S1.1), as stranded cetaceans appear spectrally unique (e.g., black colour) compared to the red colour of vegetation (Fretwell *et al.*, 2019).

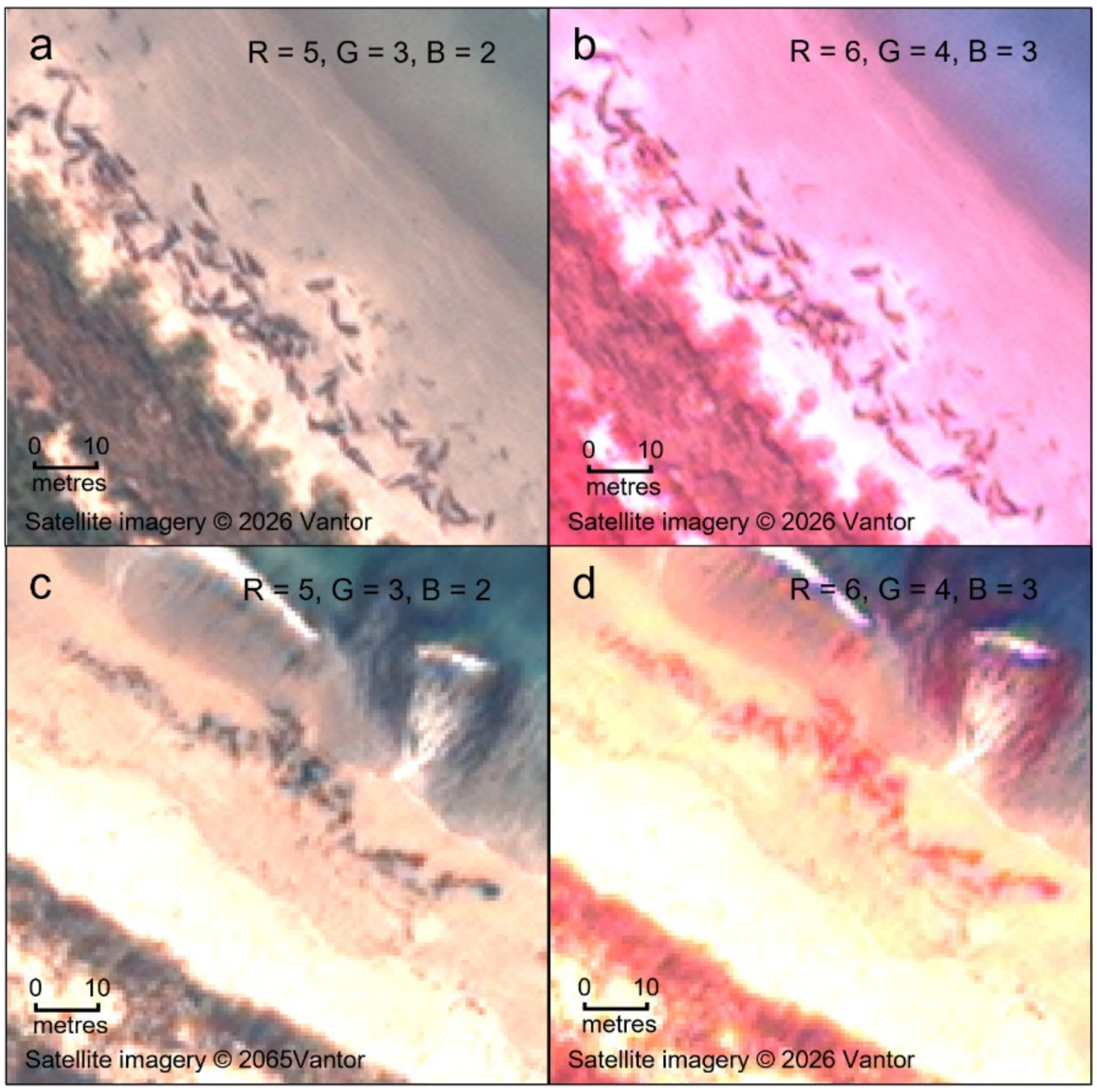

Figure S1.1: 0.5 m eight band VHR optical satellite image collected 19th October 2022 by WorldView-2, (image catalogue ID: 10300100DC306300, satellite imagery © 2026 Vantor), of a long-finned pilot whale (LFPW) mass stranding event on Chatham Island, demonstrating the power of band combination manipulation to differentiate stranded cetacean from confounding features, such as marine debris, in satellite imagery. (a) Band combination R = 5, G = 3, B = 2, showing stranded LFPW carcasses, (b) the same image as (a), only this time displayed in band combination R = 6, G = 4, B = 3. Using this band combination vegetation appears red and stranded cetaceans remain as spectrally unique dark bodies. (c) Band combination R = 5, G = 3, B = 2, showing marine debris, (d) the same image as (c), only this time displayed in band combination R = 6, G = 4, B = 3. Using this band combination vegetation appears red, including marine debris such as algae, allowing differentiation from stranded cetaceans. RGB refers to the QGIS display settings, while the numbers refer to the specific bands of the multispectral sensor.

d. consider adjusting the ‘Contrast enhancement’, ‘Min’ and ‘Max’ values for each band, ‘Brightness’, ‘Contrast’ ‘Saturation’ and ‘Gamma, to improve the images visual quality. These settings are post-processing filters that do not amend the raw image, rather they adjust the pixel values on-the-fly to aid visualisation only. These settings should be adjusted per image by the user when viewing known features in red, green and blue bands (true colour), to achieve a scene representative of reality on the ground. See QGIS documentation for further guidance on these settings.
    i. ‘Contrast enhancement’ manipulates pixel values (*e.g.,* ‘Stetch to MinMax’: stretching pixel values between the minimum and maximum across the full display range) or clips pixel values by a bounded numerical range, to improve image contrast and visualisation. This can be important to create a larger colour gradient and for removing outlying pixel values.
    ii. ‘Brightness’, ‘Contrast’ ‘Saturation’, and ‘Gamma adjust pixel values to make an image more or less bright or to alter the colour intensity, to improve image visualisation.
e. select ‘Apply’ and ‘Ok’

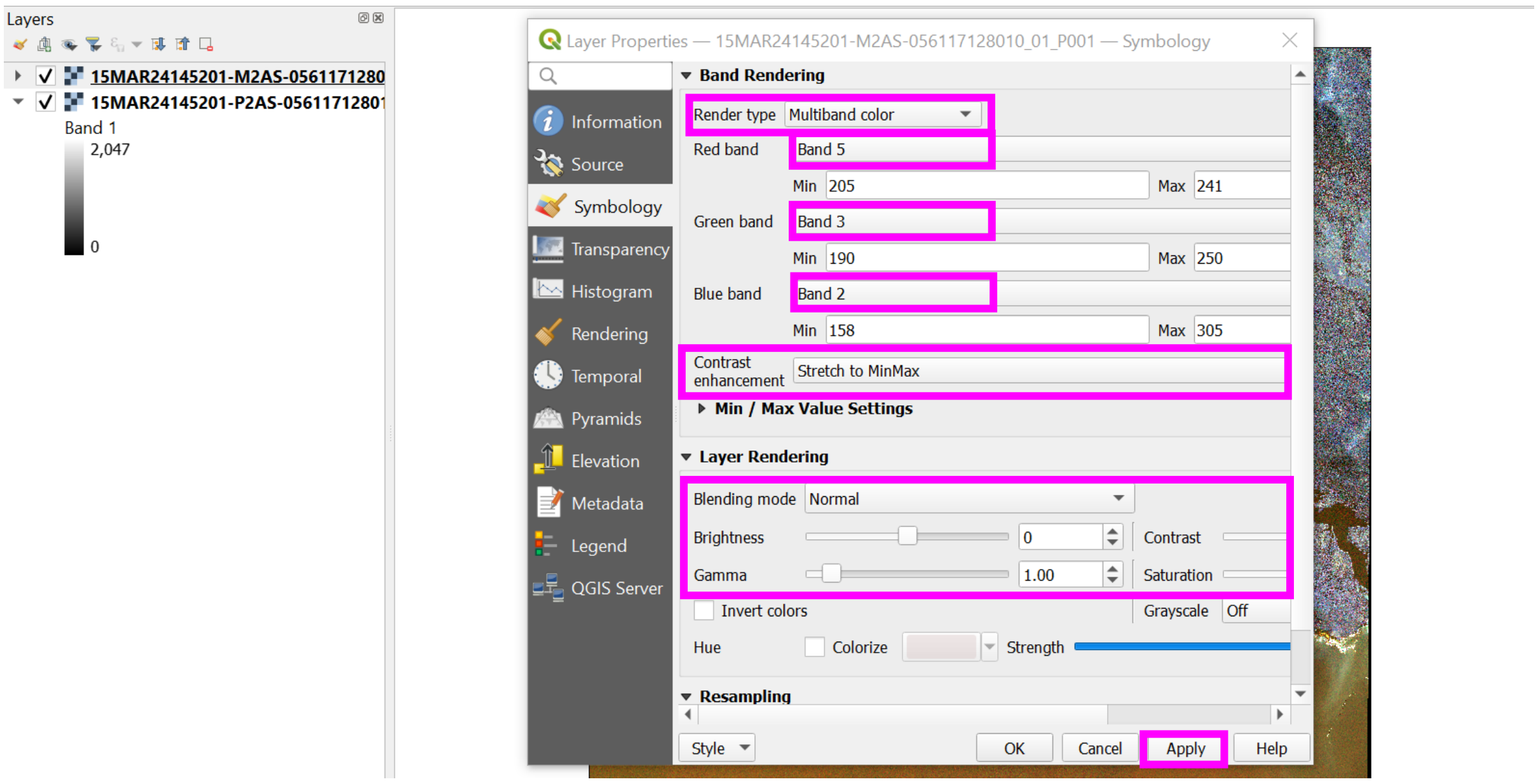

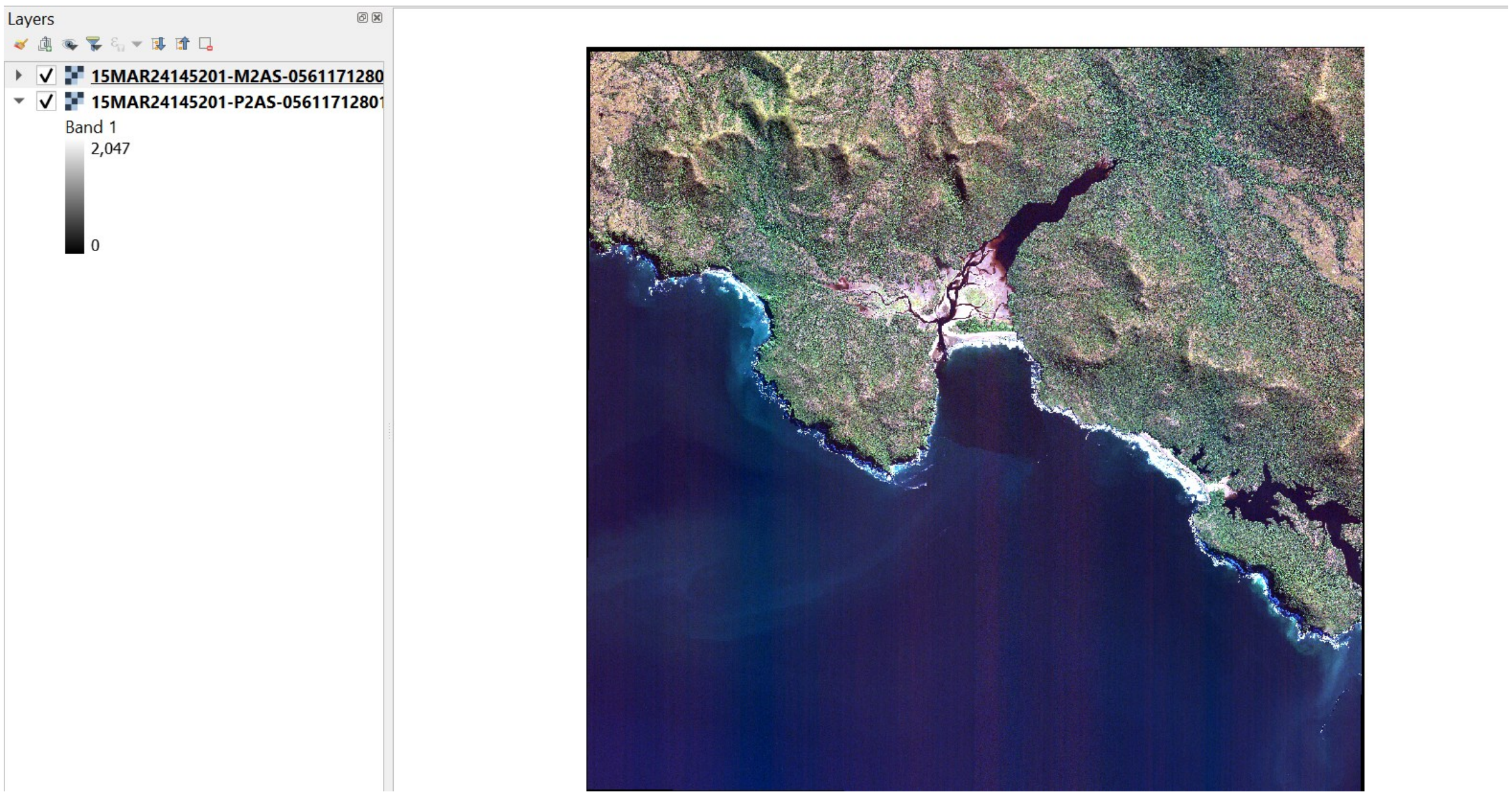

PRE-PROCESSING

PROJECTING NON-PROJECTED IMAGES

To demonstrate the following geolocation correction process, the guidance draws upon sample system ready 1b data, downloaded from Maxar (now Vantor, since the migration from Maxar to Vantor, this resource is no longer freely available), which is compared with the projected Vantor WorldView-2 View Ready OR2A data used throughout this workflow:

7. Identifying whether an image is projected (3 methods: a, b, and c)
    a. image product type:
        i. see Appendix 1 for how to access and locate information in the image metadata. For Vantor imagery, the product type can be identified by the 'IMAGEDESCRIPTOR' or 'PRODUCTTYPE' and 'PRODUCTLEVEL'. The product type can be then cross-referenced with Table 1 in the associated manuscript to inform whether an image is projected

b. image projection:

   i. see Appendix 1 for how to access and locate information in the image metadata. If the product type is projected, for example, like Vantor WorldView-2 View Ready OR2A data, the metadata file will contain a section 'MAP_PROJECTED_PRODUCT' (other satellite sensors or providers may use different terminology). This includes the 'MAPPROJNAME' and 'MAPZONE', which in this example is 'UTM' and '18' respectively. For the System Ready sample data, the '<MAP_PROJETED_PRODUCT>' section is absent, indicating the data is not projected

c. QGIS assigned Coordinate Reference System (CRS): once the image is imported to QGIS, from the 'Layers' pane, right click the image layer, select 'Properties' > 'Source', and the 'Assigned Coordinate Reference System' should reflect the information in the corresponding metadata file

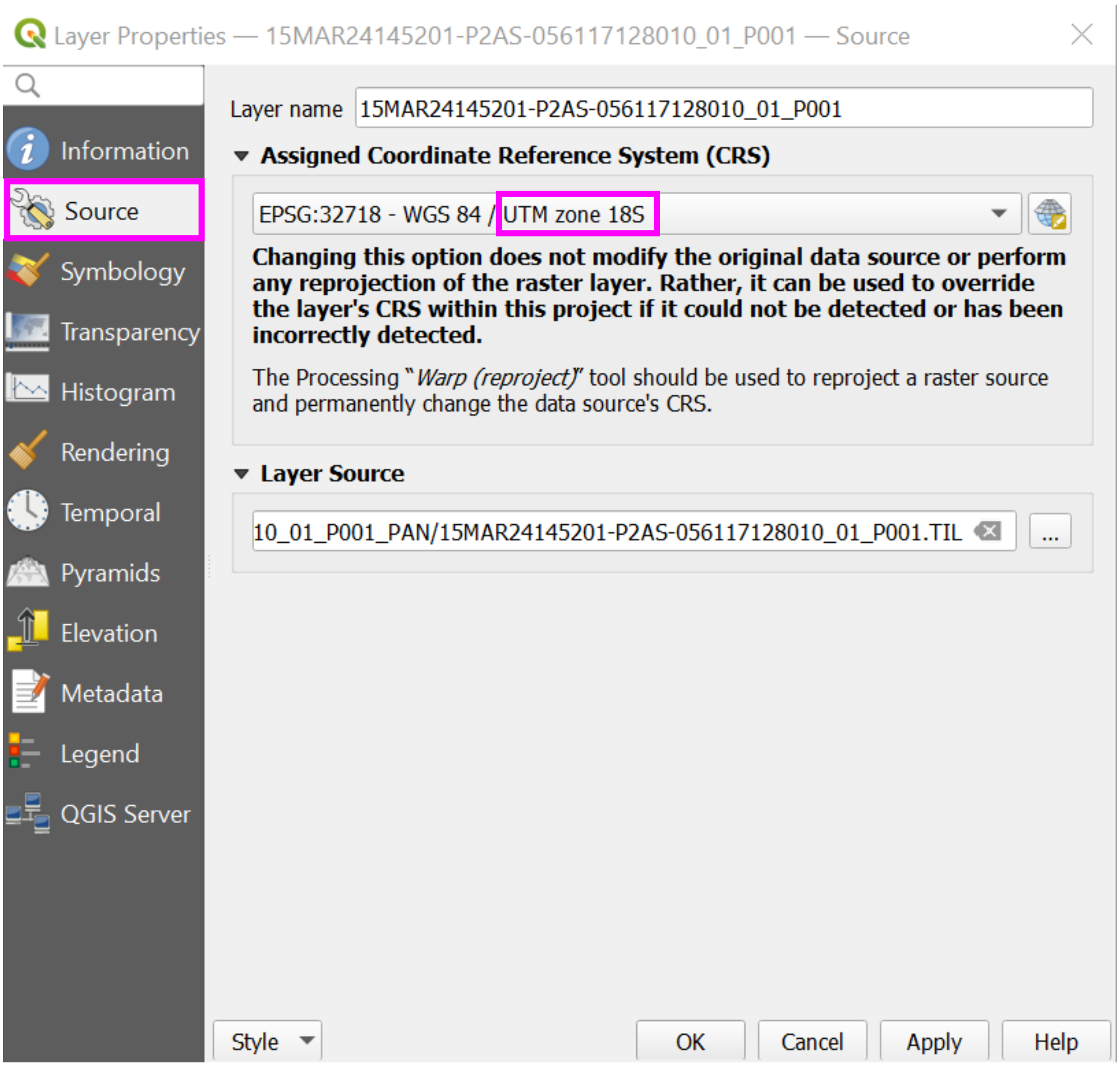

For the System Ready sample data that is not projected, a projection will be absent (assigned as default 'EPSG: 4326 – WGS 84') in the 'Assigned Coordinate Reference System (CRS) within the QGIS image 'Properties' > 'Source' tab

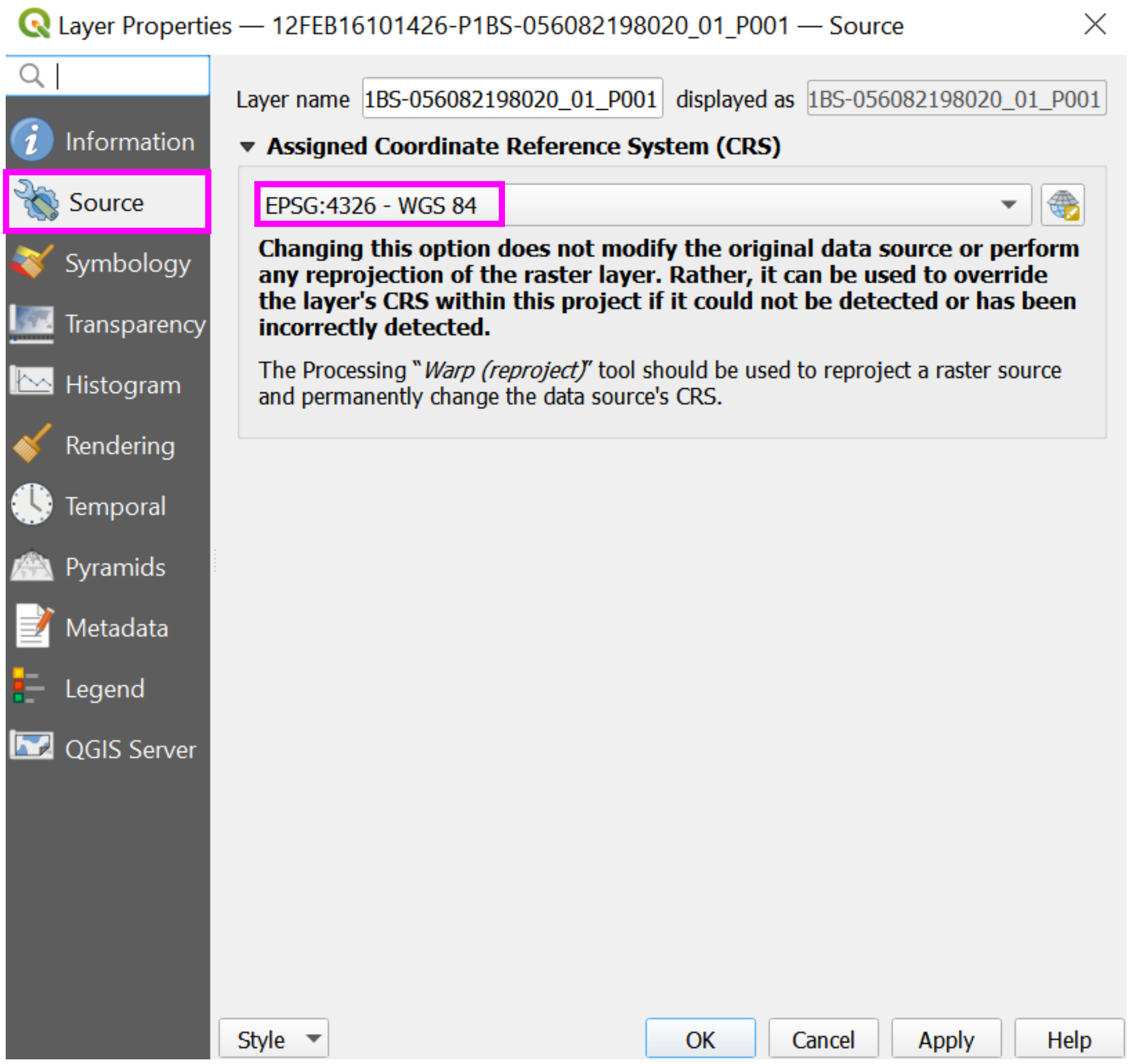

8. Assigning a projection

For data already projected, skip to section '<u>Setting up Orfeo Toolbox and Pansharpening</u>'.

a. view the UTM projection guide <u>https://www.dmap.co.uk/utmworld.htm</u> to identify the correct projection (the sample System Ready image falls directly between UTM zone 33V or 34V. In this example, either zone could be chosen, though distortion may occur in the part of the image zone that is not chosen. Where images overlap zones, best practice would be to choose the UTM zone containing the majority of image or area of interest)
b. verify a selected UTM zone before re-projecting an image, within QGIS
    i. Import the image into QGIS as per '<u>Importing images</u>', select 'Project' from the main toolbar, followed by 'Properties'

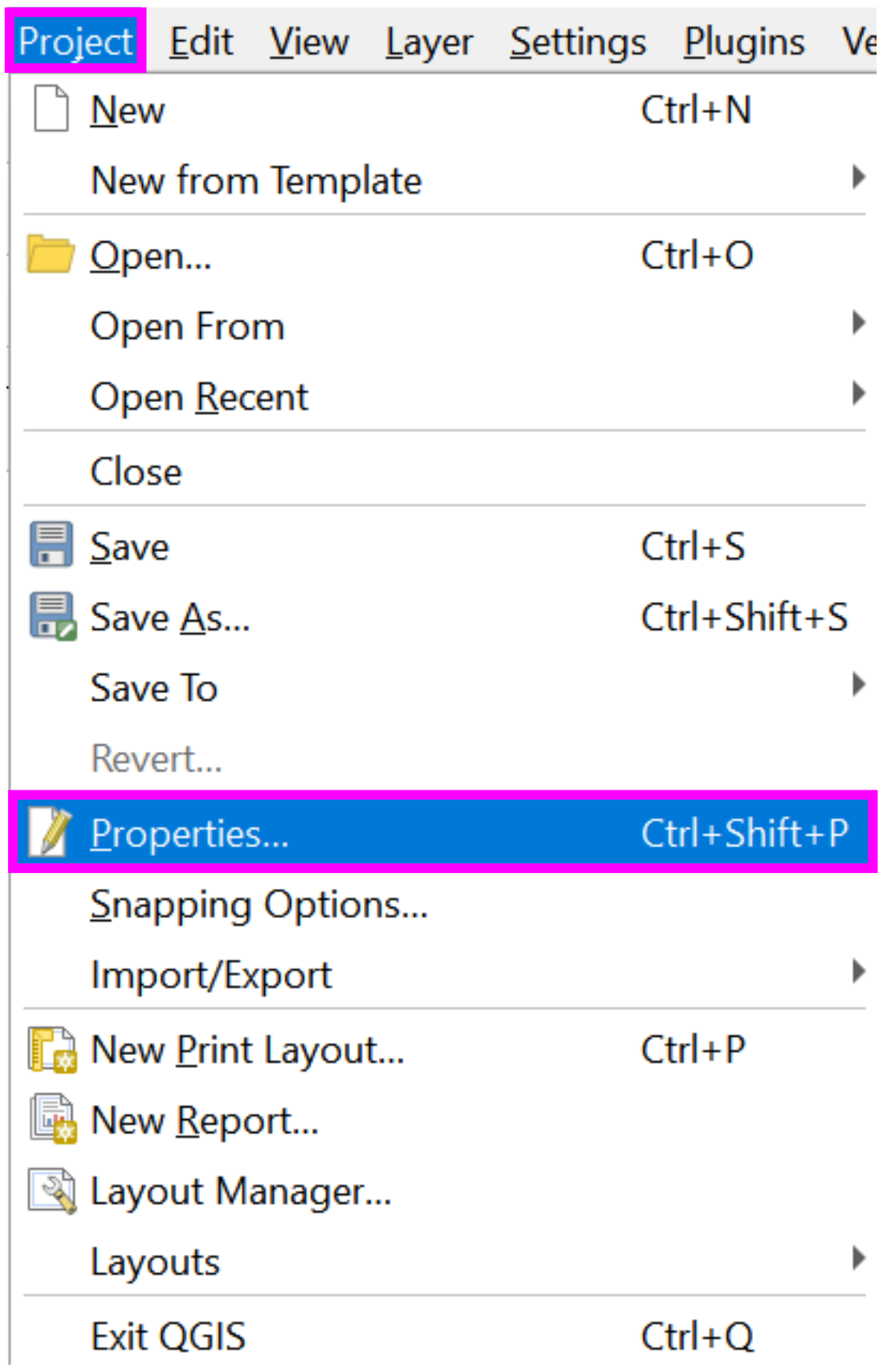

ii. Under the 'CRS' tab, search the expected UTM zone in the 'Filter' box, the purple cross in the pane displaying the globe refers to the location of your image, and the red rectangle indicates the UTM zone. If the purple cross is in the expected location and the red UTM zone aligns, then the planned projection is correct. Once the planned re-projection is verified, do not press 'Apply' or 'Ok', instead click the 'x' to exit the 'Properties' window, as this exercise was for verification purposes only. The image can now be re-projected using the GDAL 'Warp (reproject)' tool.

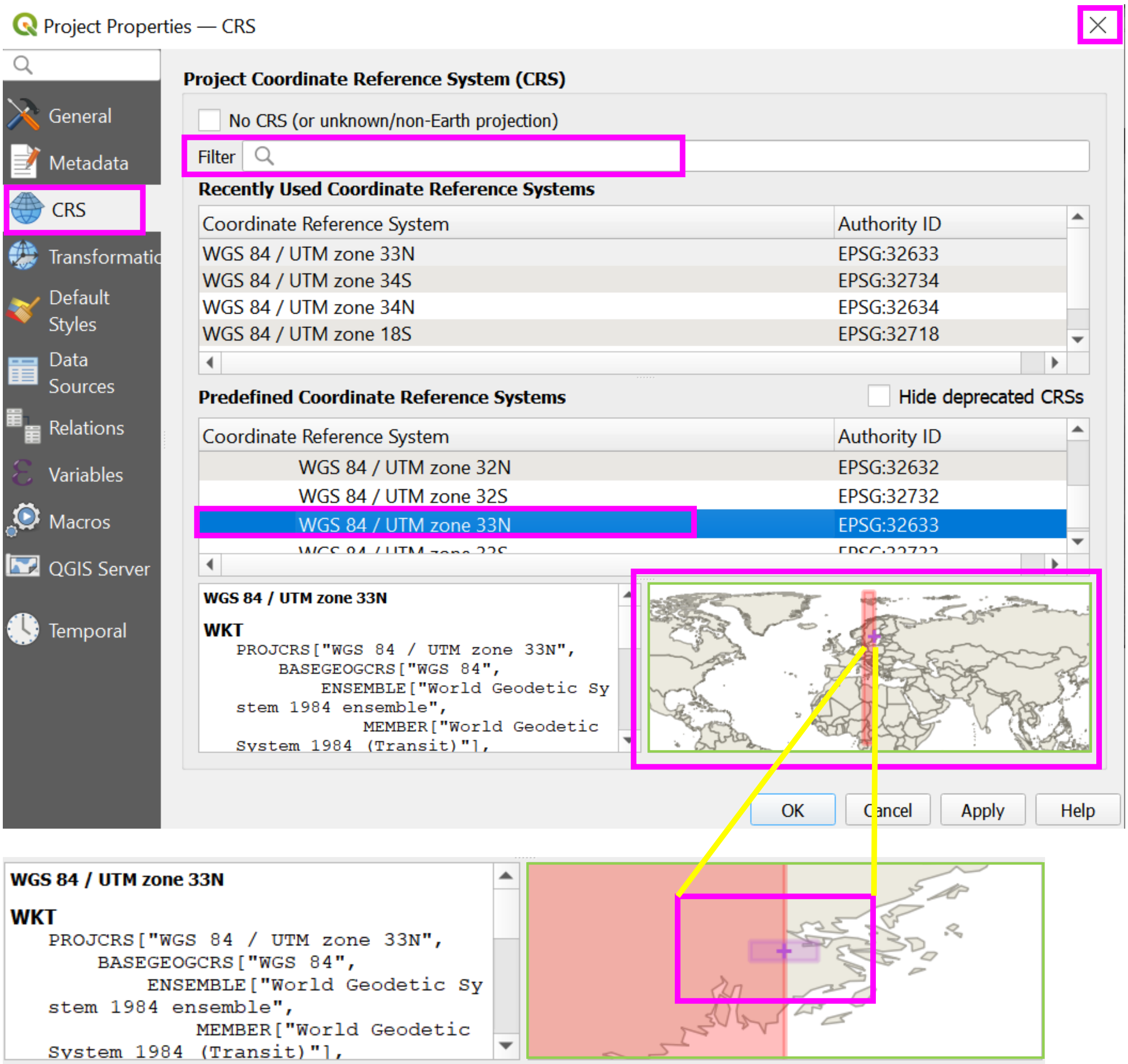

9. Re-projecting an image
    a. Open the ‘Processing Toolbox’ using the cog icon on the main toolbar

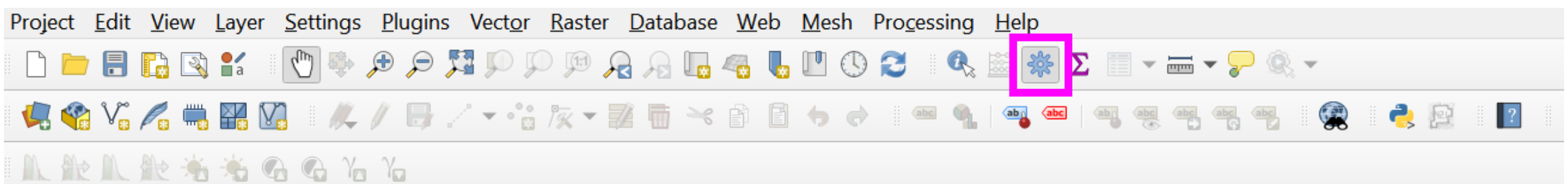

    b. In the ‘Processing Toolbox’ on the right of the QGIS window select ‘GDAL’ > ‘Raster projections’ > ‘Warp (reproject)’

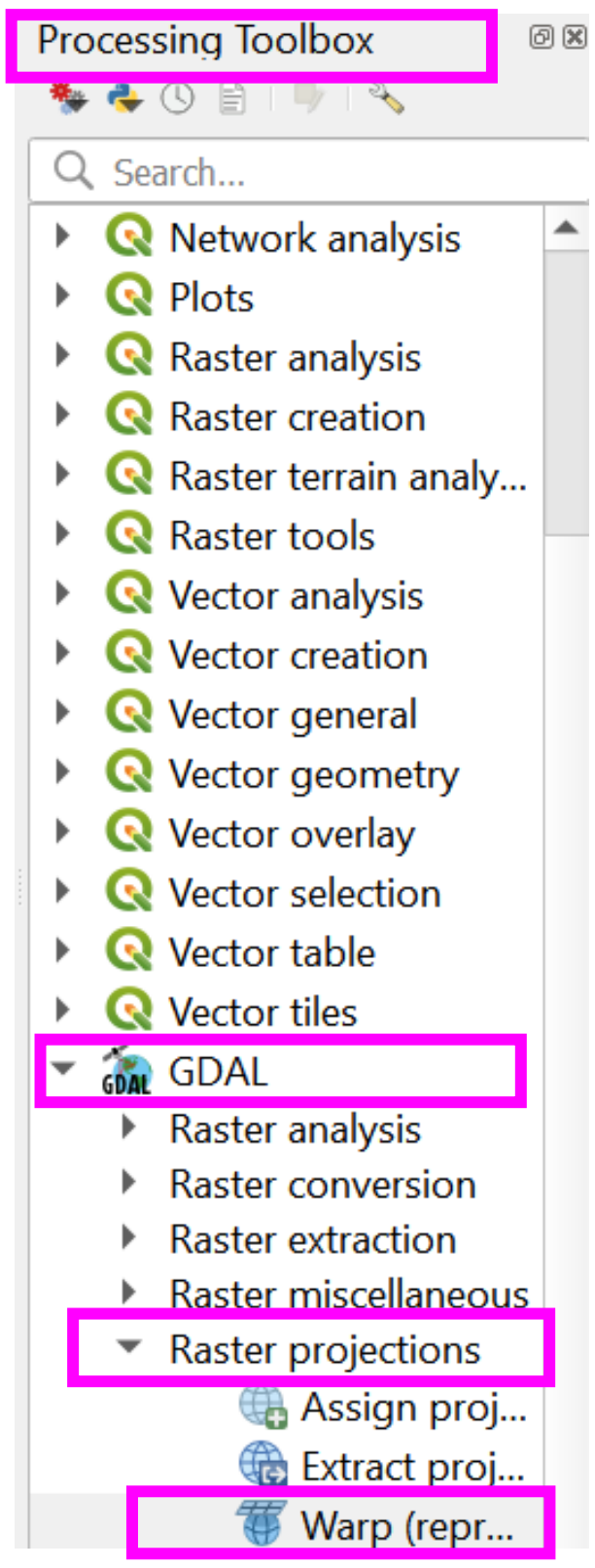

c. select the System Ready data layer as the 'Input Image' (if you have a panchromatic and multispectral image, you will need to repeat this process for both)
d. set the 'Source CRS' as the current projection assigned by QGIS (identified earlier when viewing the image source in the image properties, section 7.c, *e.g.,* 'EPSG:4326 – WGS 84' or leave blank
e. set the 'Target CRS' as the UTM zone identified using the projection guide: https://www.dmap.co.uk/utmworld.htm, *e.g.,* WGS 84/UTM zone 33N.
f. in the 'Advanced Parameters' > 'Additional command-line parameters' section, for correction without detailed elevation (setting to a constant height of 0), add the following statement: -rpc -to "RPC_HEIGHT=0"
g. if correcting using a DEM, add the following statement, ensuring to amend the '/path/to/dem.tif' to the correct path of the DEM local source file: -rpc -to "RPC_DEM=/path/to/dem.tif"
h. define a save location and file name within 'Reprojected'
i. select 'Run

**The same method can be used to orthorectify projected View Ready OR2A data with a digital elevation model, for projects that require a height correction. Ensure to select the 'Target CRS' to the same as the input image 'Source CRS', and set the 'Output file resolution in target georeferenced units [optional]' to the input image spatial resolution. See Appendix 1 for information on how to access and locate the spatial resolution in the image metadata.

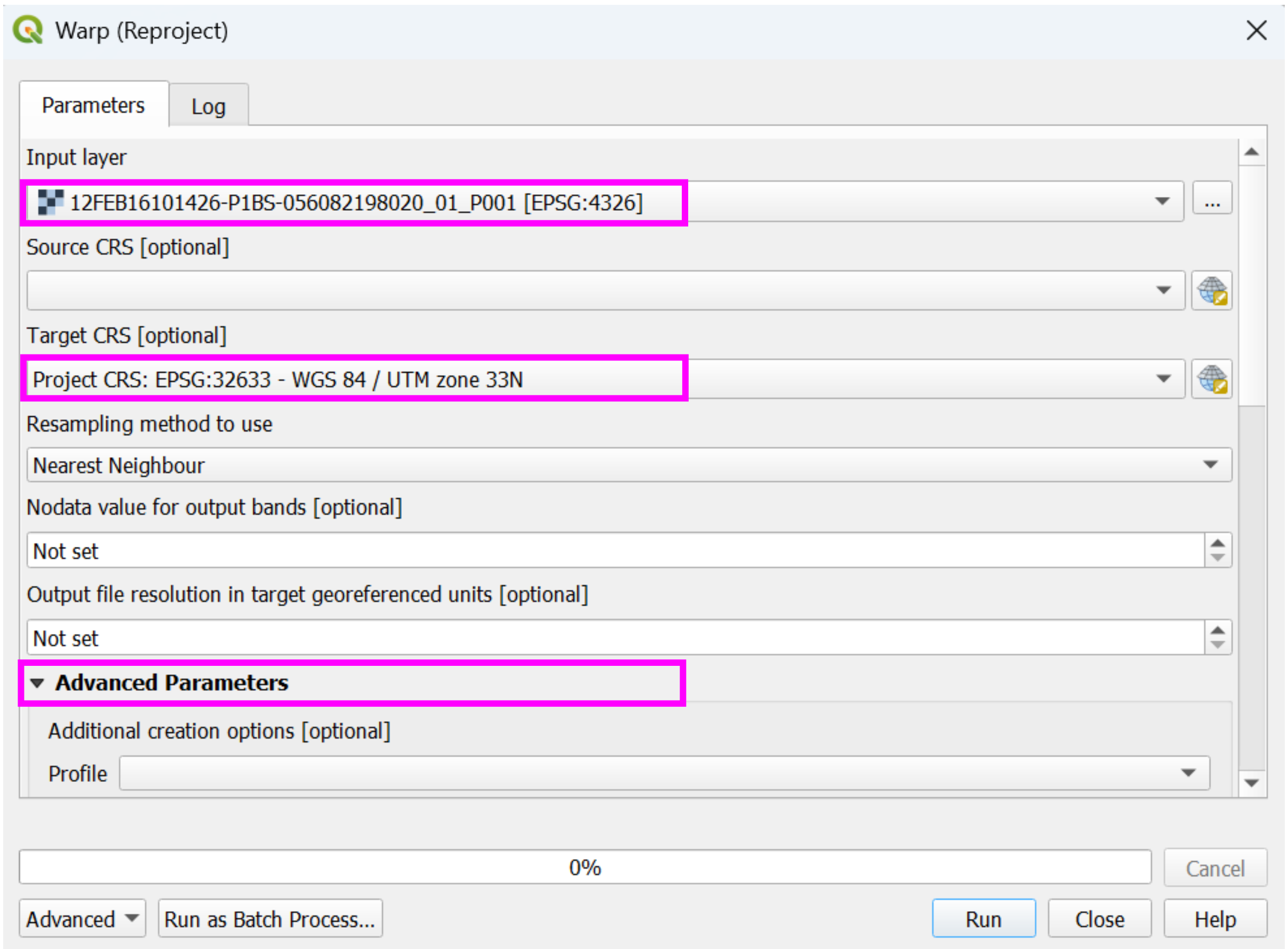
Warp (Reproject)
Parameters
Log
Input layer
12FEB16101426-P1BS-056082198020_01_P001 [EPSG:4326]
Source CRS [optional]
Target CRS [optional]
Project CRS: EPSG:32633 - WGS 84 / UTM zone 33N
Resampling method to use
Nearest Neighbour
Nodata value for output bands [optional]
Not set
Output file resolution in target georeferenced units [optional]
Not set
Advanced Parameters
Additional creation options [optional]
Profile
0%
Cancel
Advanced
Run as Batch Process...
Run
Close
Help

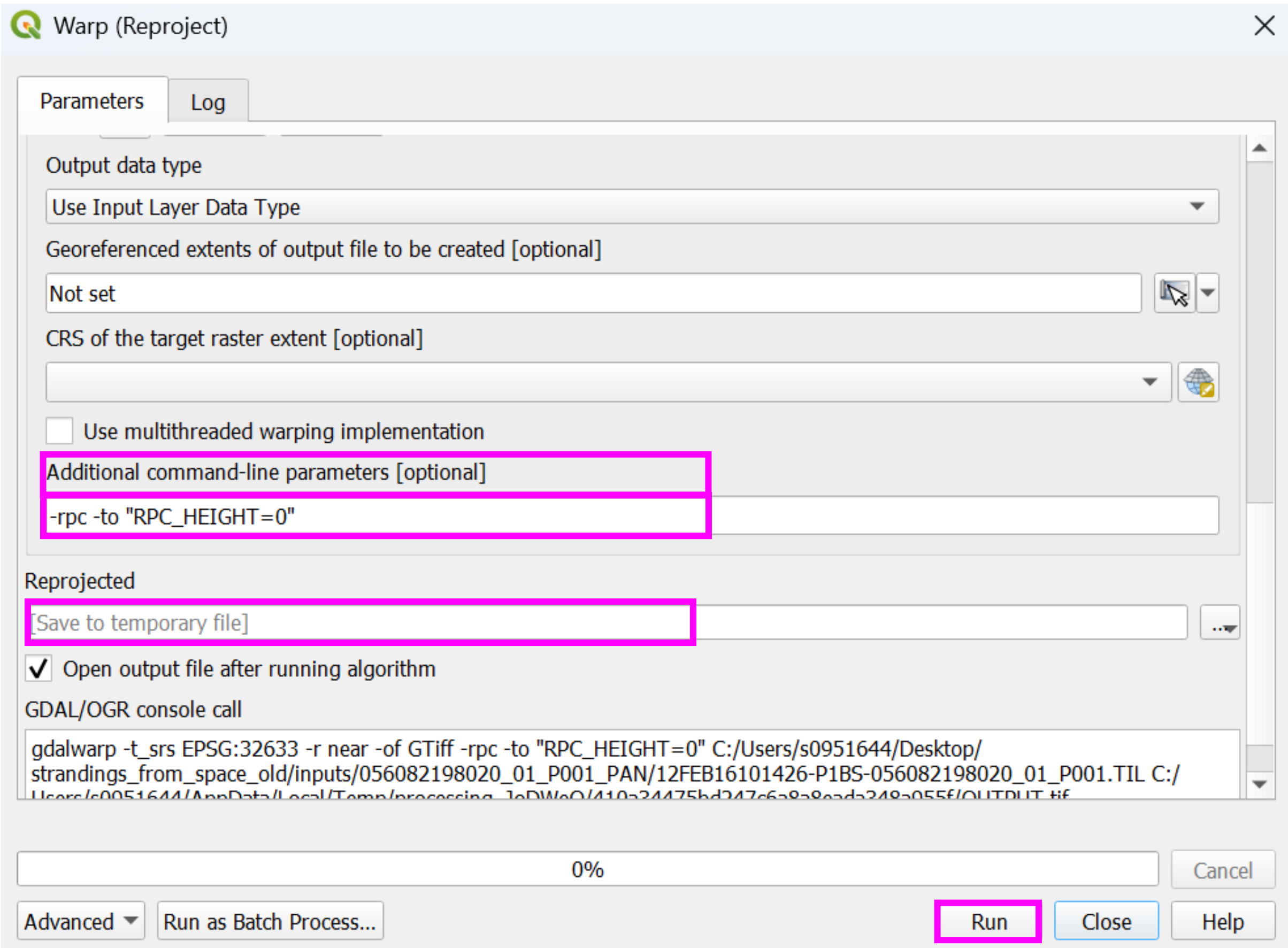
Warp (Reproject)
Parameters
Log
Output data type
Use Input Layer Data Type
Georeferenced extents of output file to be created [optional]
Not set
CRS of the target raster extent [optional]
Use multithreaded warping implementation
Additional command-line parameters [optional]
-rpc -to "RPC_HEIGHT=0"
Reprojected
[Save to temporary file]
Open output file after running algorithm
GDAL/OGR console call
gdalwarp -t_srs EPSG:32633 -r near -of GTiff -rpc -to "RPC_HEIGHT=0" C:/Users/s0951644/Desktop/
strandings_from_space_old/inputs/056082198020_01_P001_PAN/12FEB16101426-P1BS-056082198020_01_P001.TIL C:/
0%
Cancel
Advanced
Run as Batch Process...
Run
Close
Help

10. Setup Orfeo Toolbox plugin (in this workflow we used version 'OTB 8.1.1 Win64'
    a. download Orfeo Toolbox (operating system specific): https://www.orfeo-toolbox.org/download/ (for 8.1.1: https://www.orfeo-toolbox.org/otb-8-1-1-release/)

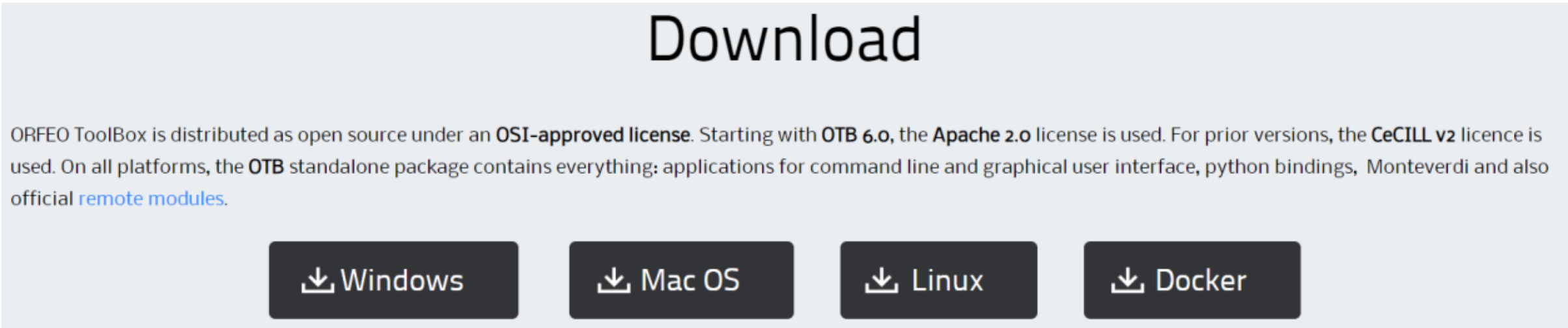

    b. right click the downloaded zip 'OTB-8.1.1-Win64' folder > 'Extract All…' > select a suitable location to store the OTB library and 'Extract'

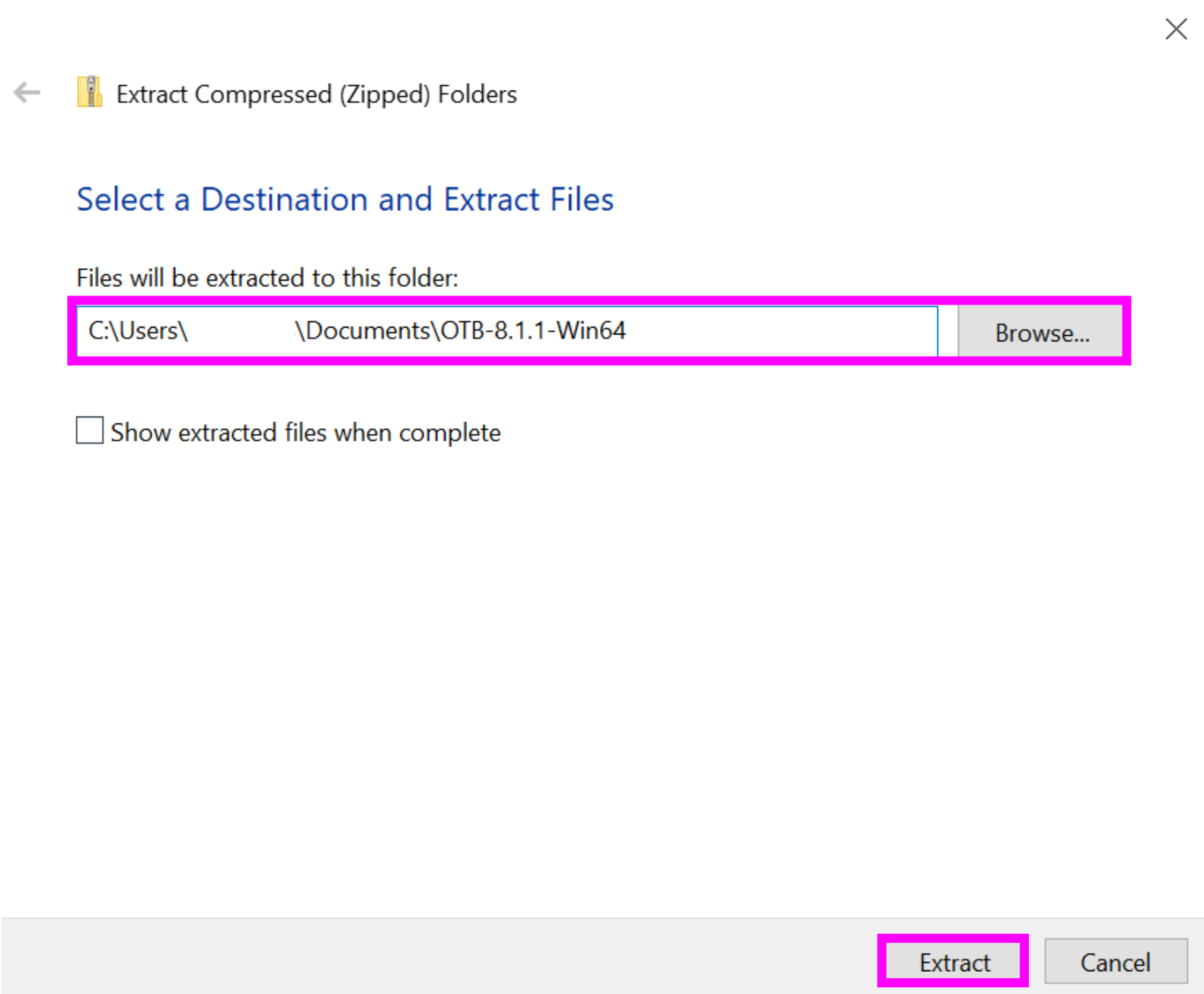

    c. in QGIS, select 'Processing' from the main toolbar
    d. select 'Toolbox' from the dropdown menu

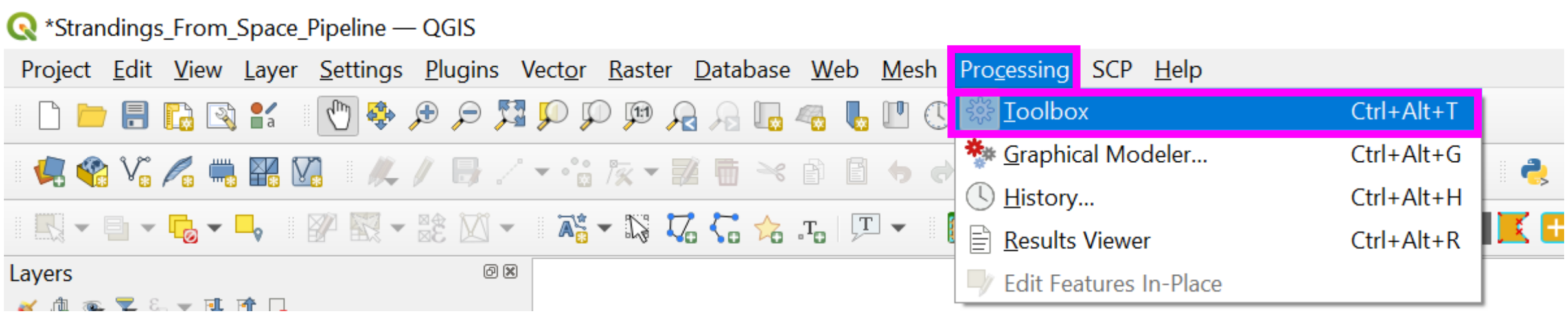

    e. the 'Processing Toolbox' will appear at the right of the QGIS window (OTB is automatically installed with QGIS 3.28.1, however, it may need enabling. If already enabled, 'OTB' will appear as an option in the 'Processing Toolbox', if enabled, continue to section 10.j)

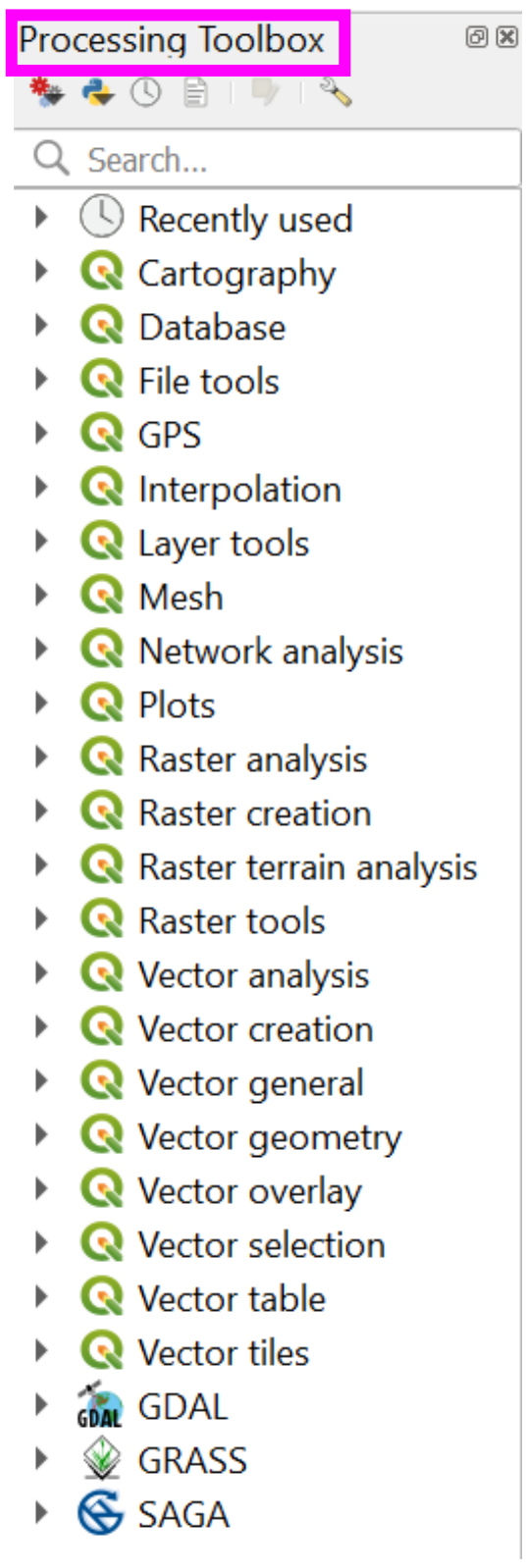

f. to enable OTB, select ‘Plugins’ from the main menu, then ‘Manage and Install Plugins…’

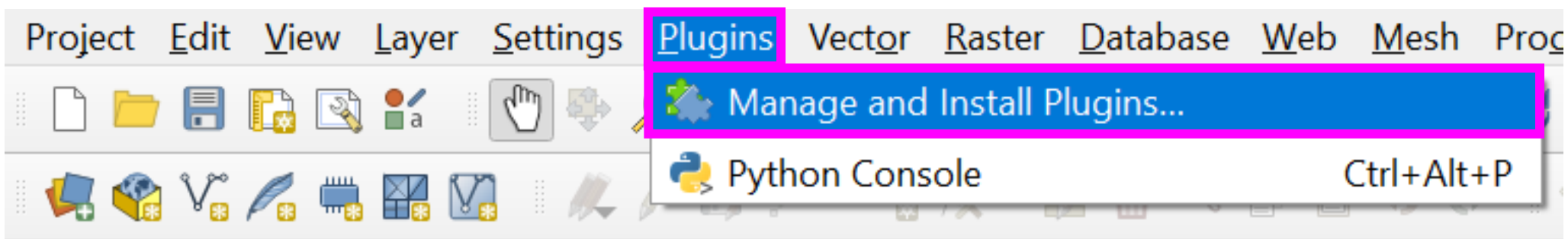

g. under the ‘Installed’ tab, begin to type ‘orfeo’ in the search bar, ‘OrfeoToolbox provider’ should appear (please note: for QGIS version >3.36 OTB must be installed first, so type ‘orfeo’ in the ‘not-installed’ tab).

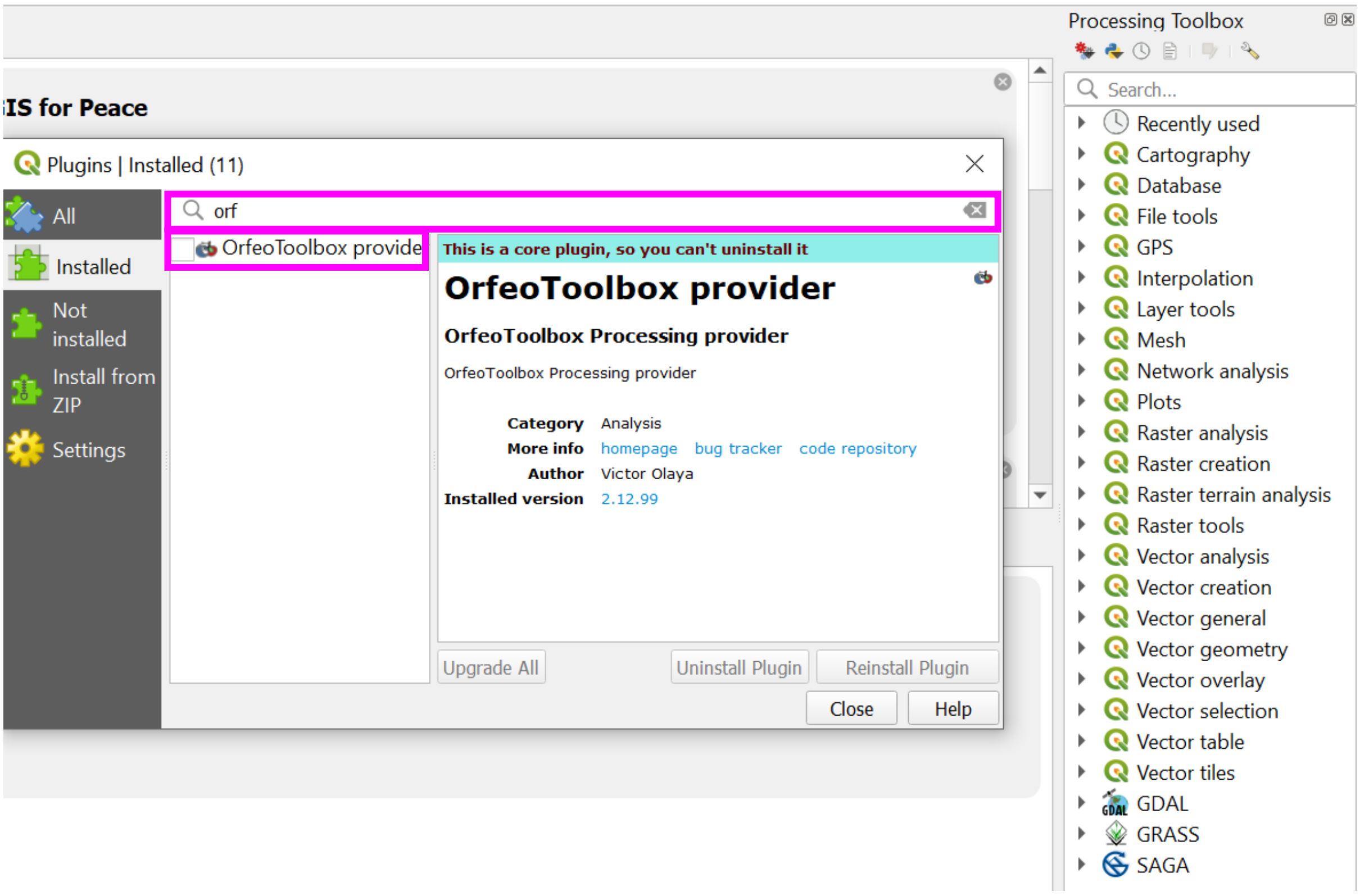

h. check the box next to 'OrfeoToolbox provider' to enable and 'Close', 'OTB' should appear in the 'Processing Toolbox'

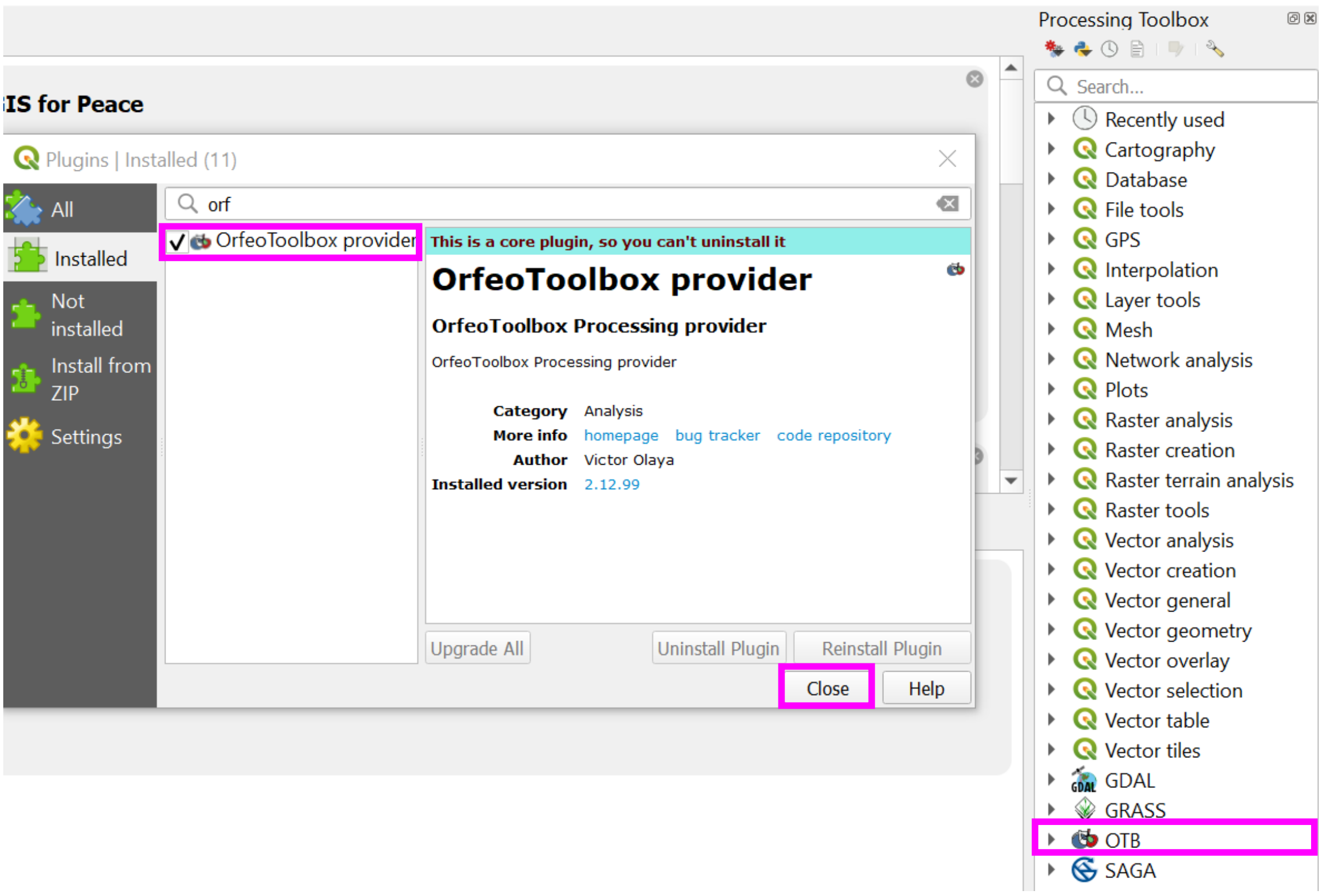

i. once 'OTB' is available in the 'Processing Toolbox', select 'Options' (the spanner icon in the 'Processing Toolbox' toolbar)

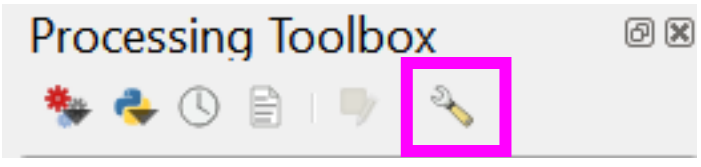

j. under ‘Setting’ > ‘Providers’ > ‘OTB’ select the three dots next to the following options, and navigate to your downloaded and extracted ‘OTB-8.1.1-Win64’ folder, providing the location/relevant links below:
    i. ‘OTB application folder’: C:/‘user specific information’/OTB-8.0.1-Win64/OTB-8.0.1-Win64/lib/otb/applications
    ii. ‘OTB folder’: C:/ ‘user specific information’/OTB-8.0.1-Win64/OTB-8.0.1-Win64
k. once the links are set > ‘OK’

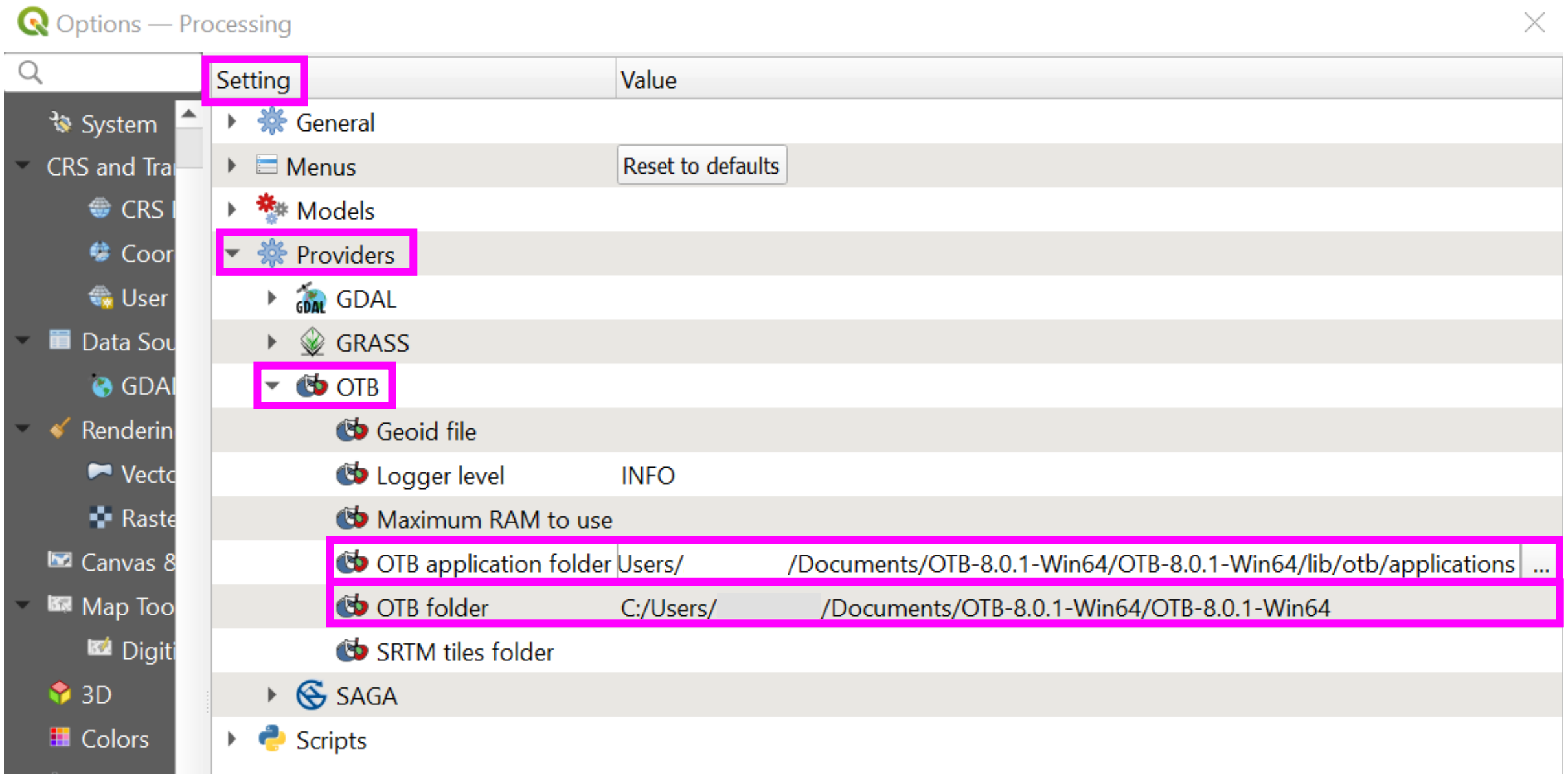

11. Pansharpening using OTB
    a. in the ‘Processing Toolbox’ select ‘OTB’ > ‘Geometry’ > ‘BundleToPerfectRaster’

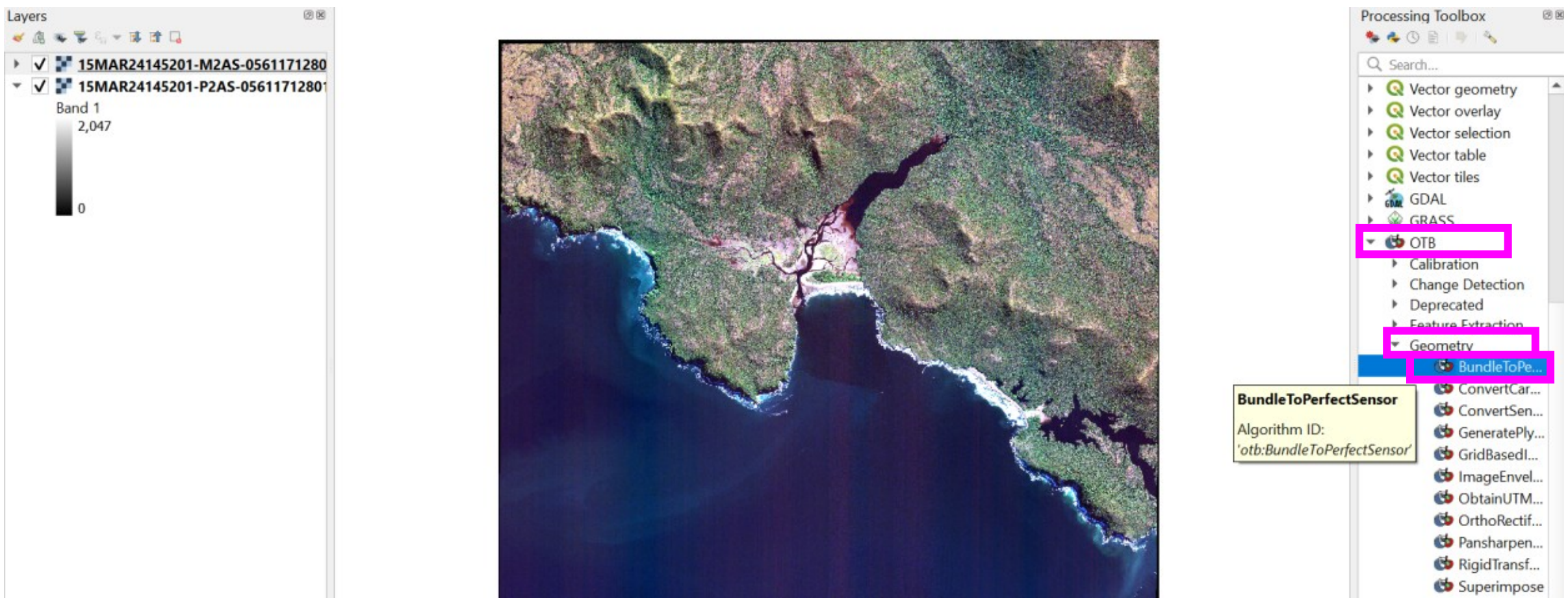

    b. from the dropdown menu, set the ‘Input PAN Image’ as your panchromatic image (‘P’ indicates panchromatic), and set the ‘Input XS Image’ as your multispectral image (‘M’ indicates multispectral)

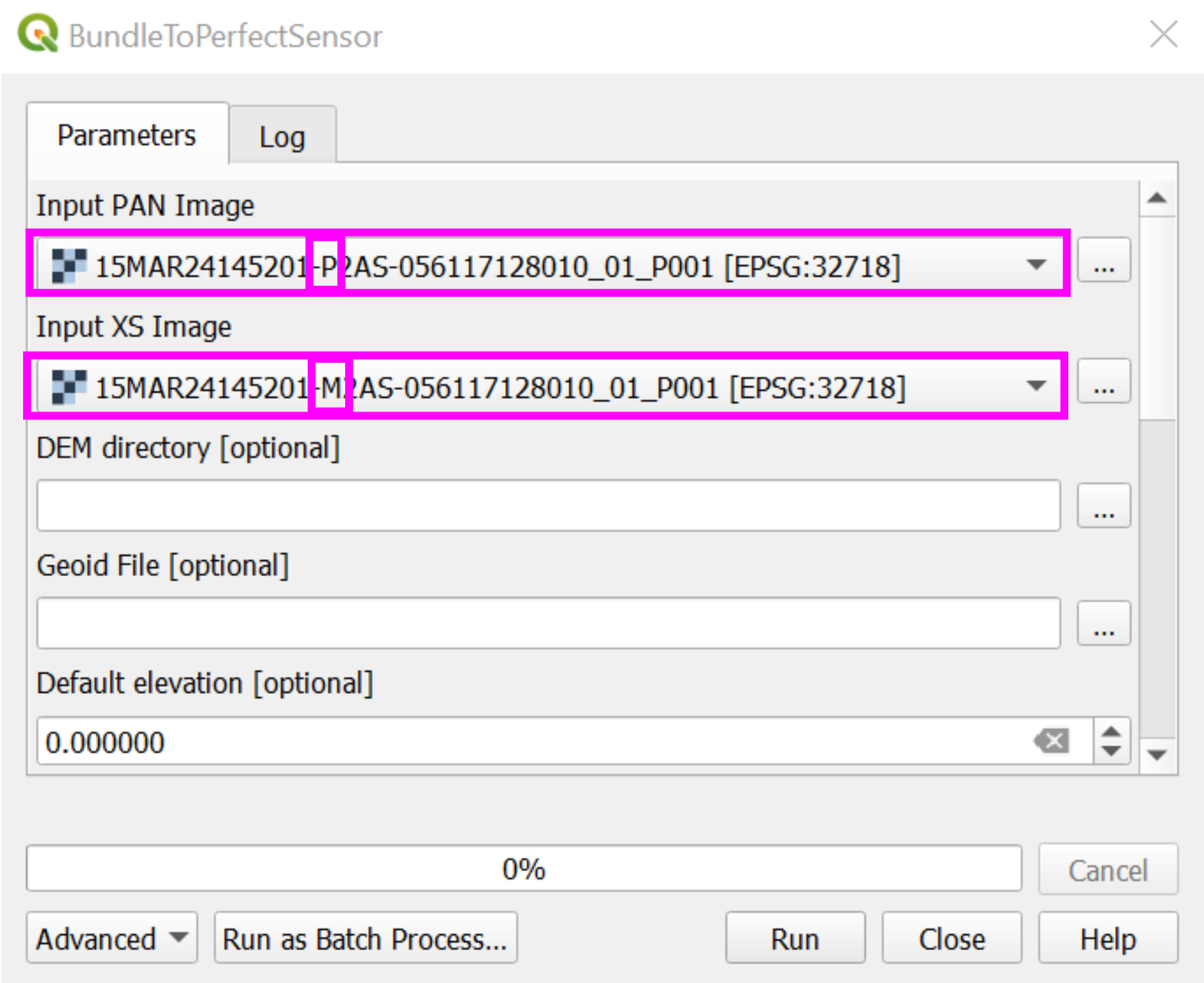

c. the 'Mode' as default, uses a panchromatic and multispectral sensor model to estimate the transformation, superimposing the multispectral image over the panchromatic image before the fusion. If using Pleiades imagery this 'Mode' should be set to 'PHR'. This should be automatically detected when inputting your image, though make sure to check
d. amend the 'Algorithm' your pansharpening will use, from the options 'RCS', 'Bayes' and 'LMVM'. RCS', 'Bayes' and 'LMVM' come with default settings (such as the 'Spacing of the deformation field'), you can tweak these to improve your output. 'Bayes' provides the highest quality output, though takes the longest time to execute
e. set the 'Interpolation'. BCO, otherwise known as Bicubic Interpolation, offers the highest quality output, but again comes at a trade-off, of increased time to process

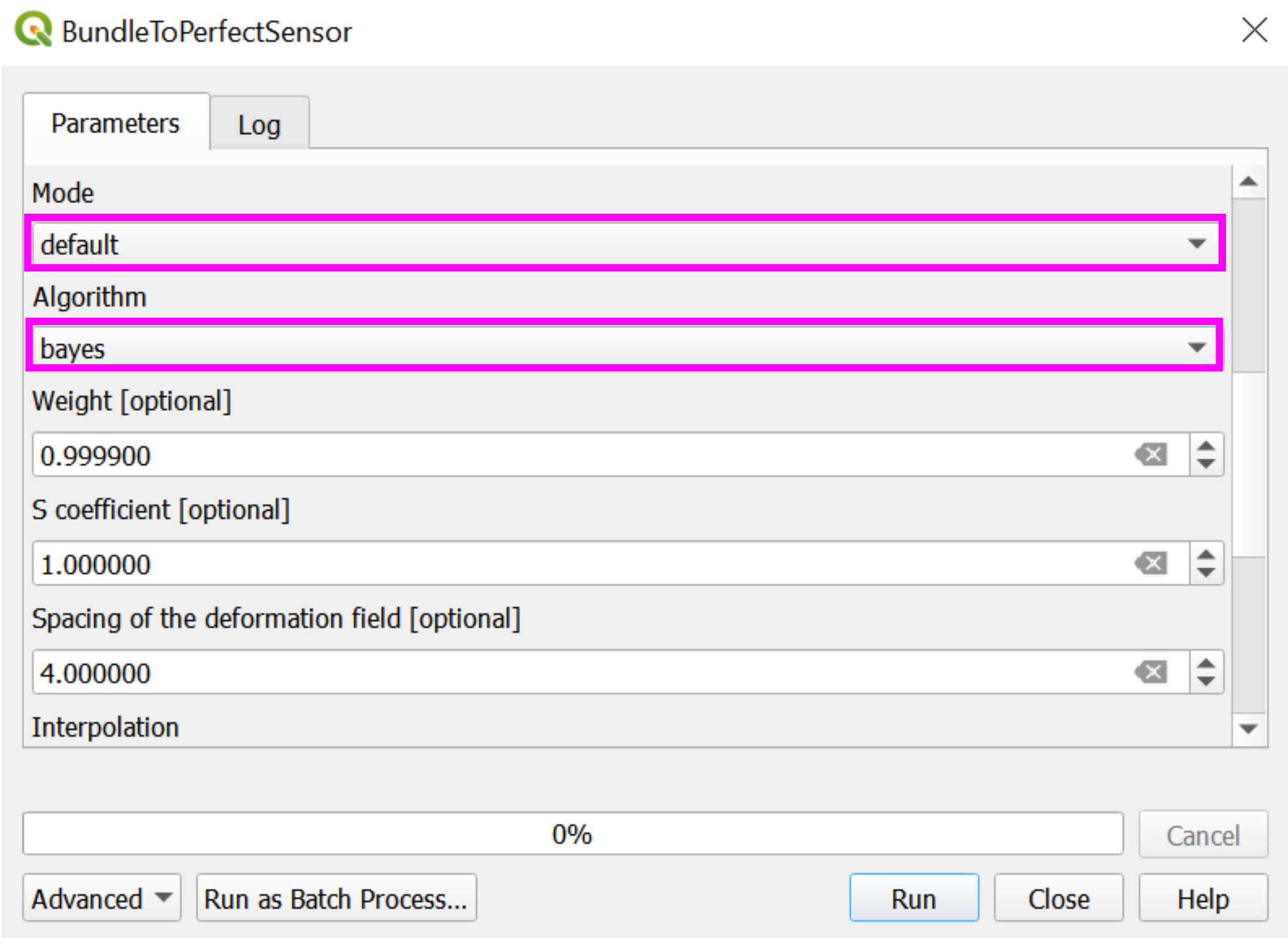

f. before processing, set your 'Output image' as 'Save To File…', identifying a save location and file name

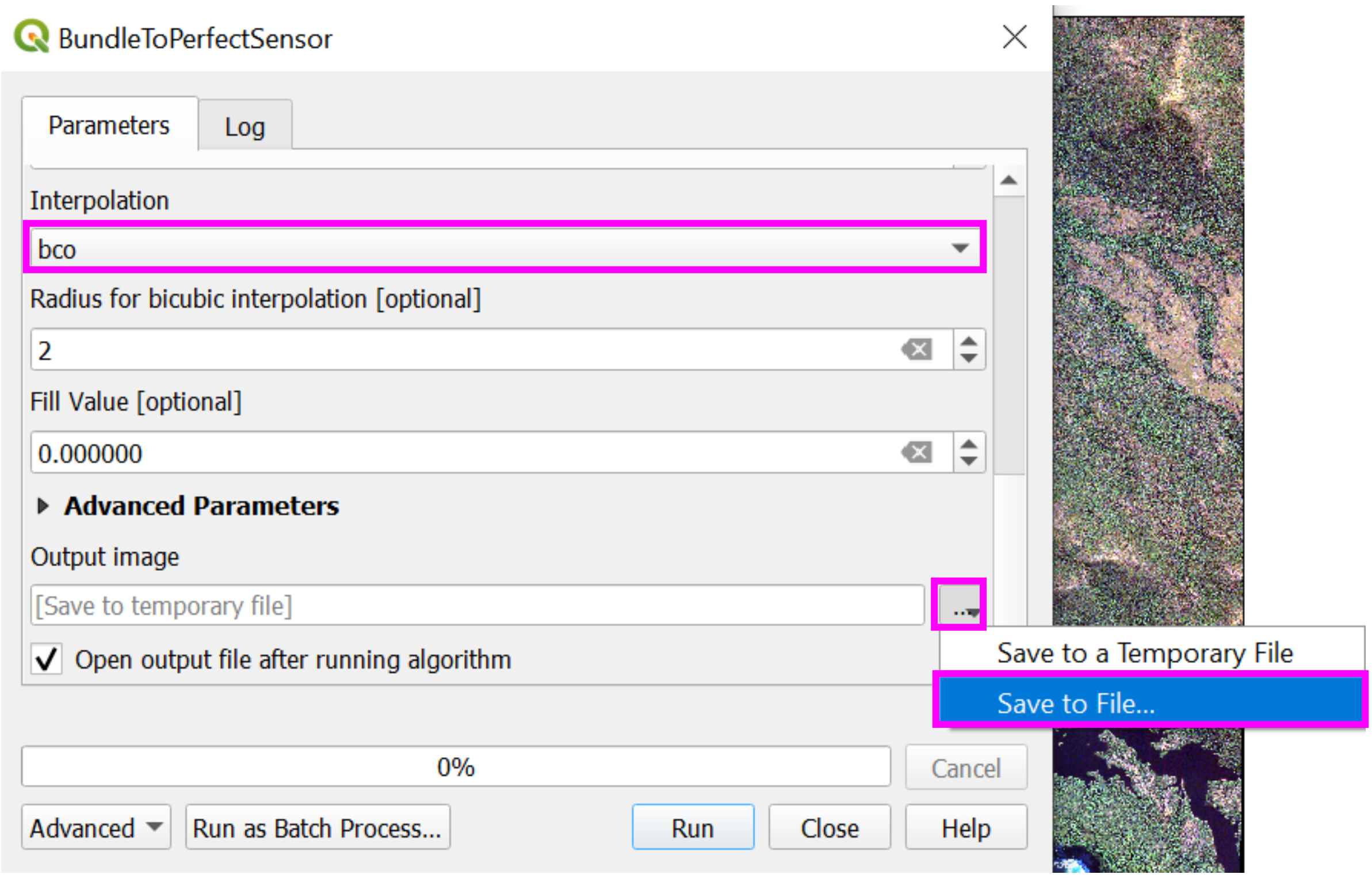

g. run the pansharpening algorithm by selecting 'Run'

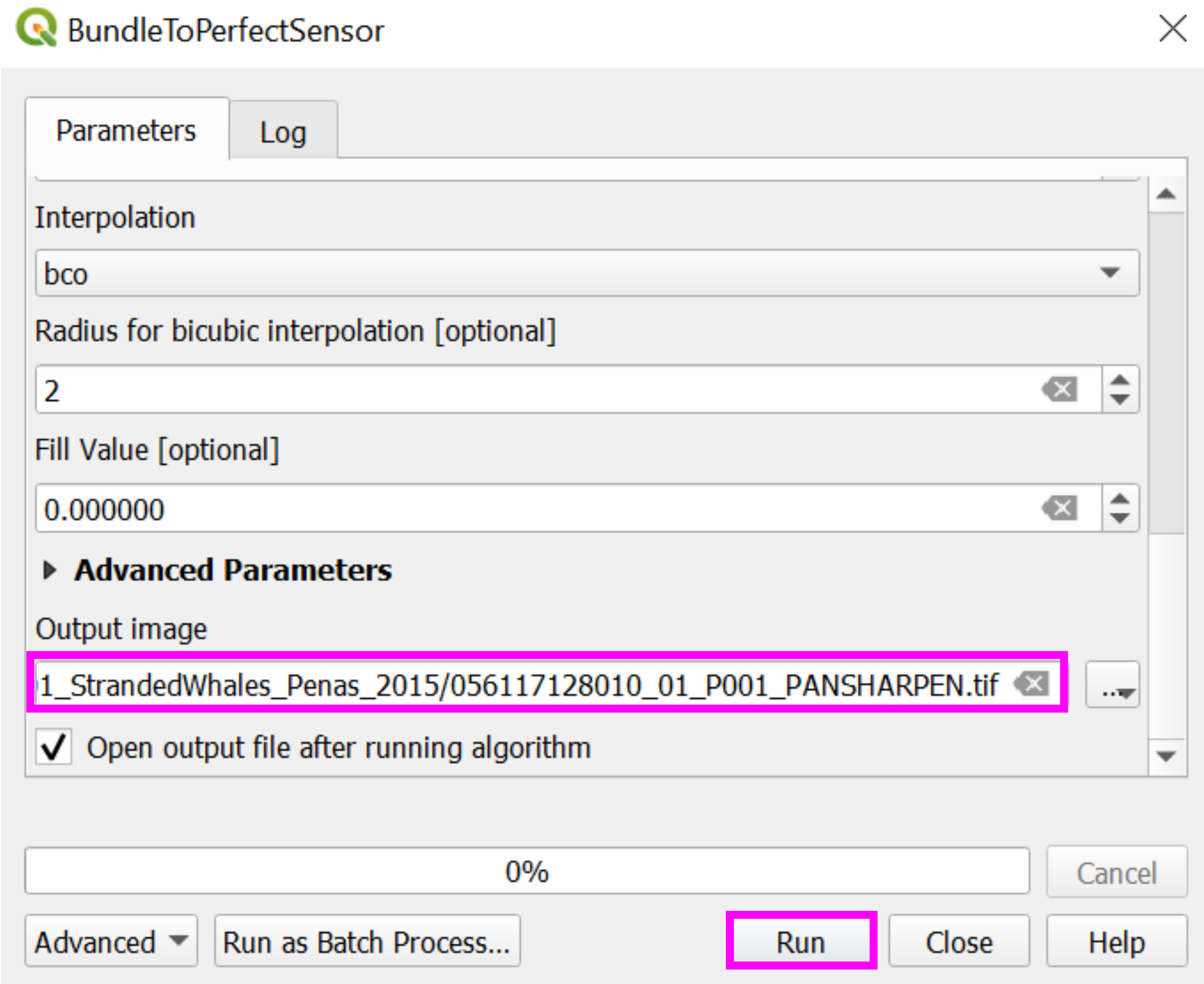

h. the output pansharpened image will appear in the 'Layers' pane. Depending on your file size, this process could take hours. This is because QGIS creates a new file, unlike ArcGIS, which creates a virtual raster (VRT). For a reference of time taken, the image file used in this example (multispectral file 168.86MB and a panchromatic file 387.67MB, with an approximate area of 321km^2), under 'Algorithm' 'RCS' and 'Interpolation' 'bco', execution completed in 2910.63 seconds (48 minutes 31 seconds) and under 'Algorithm' 'Bayes' and 'Interpolation' 'bco', execution completed in 4829.19 seconds (1 hour 20 minutes 29 seconds). As a cost benefit analysis examples of each output are shown in section 11.j)

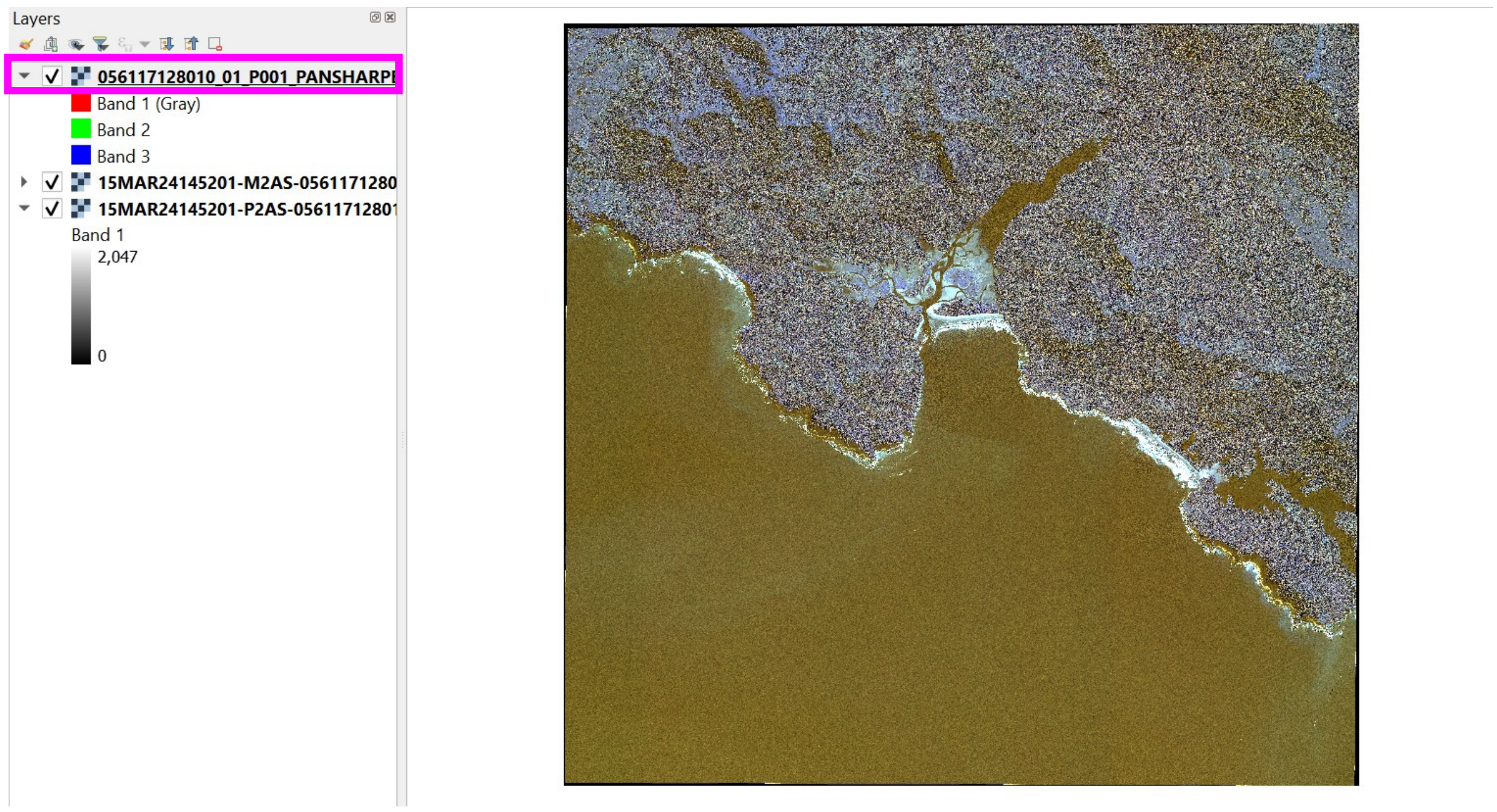

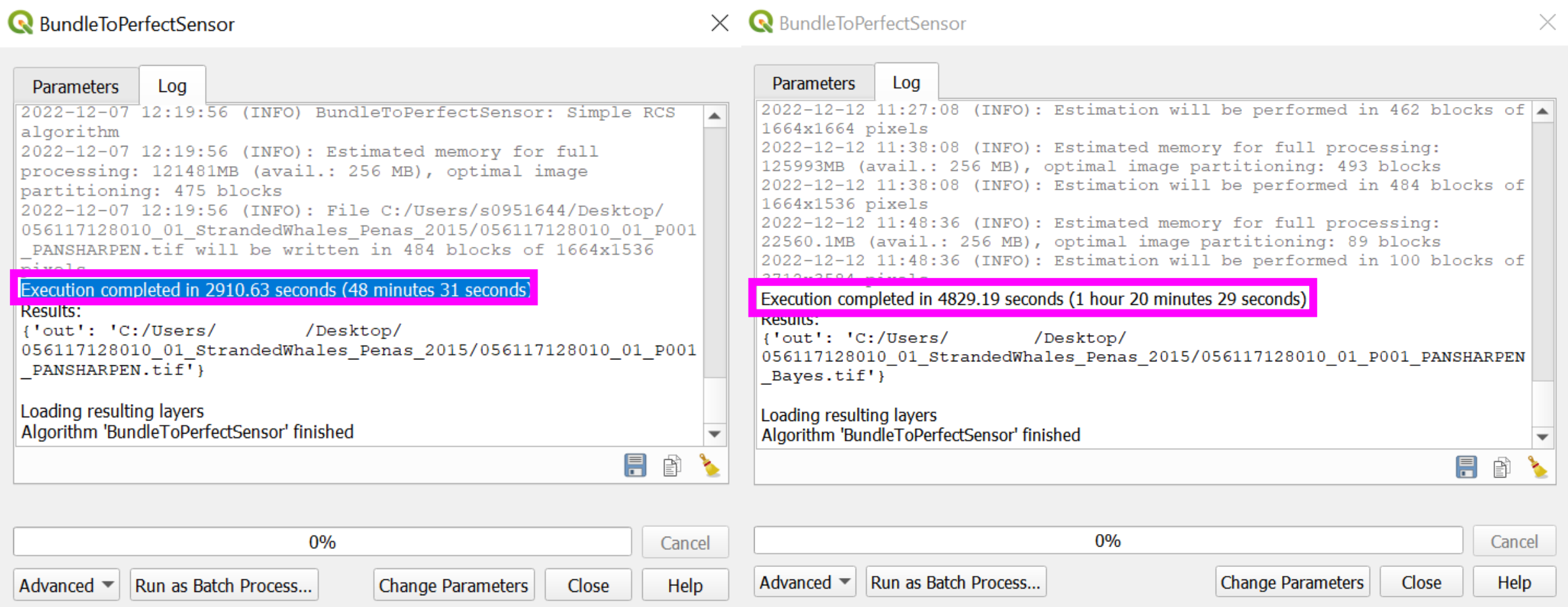

i. as in section 'Importing a multispectral image', amend the image bands as required to correctly visualise the image (*e.g.*, for WorldView-2, R = 5, G = 3 and B = 2)

j. the below image examples, demonstrate the improved image resolution resulting from the process of pansharpening (visualising different layers, can be achieved by unchecking or reordering layers in the 'Layers' pane). Image 1 = the original multispectral image, image 2 = 'RCS' pansharpened image and image 3 = 'Bayes' pansharpened image, both image 2 and 3 have much greater visible details than image 1

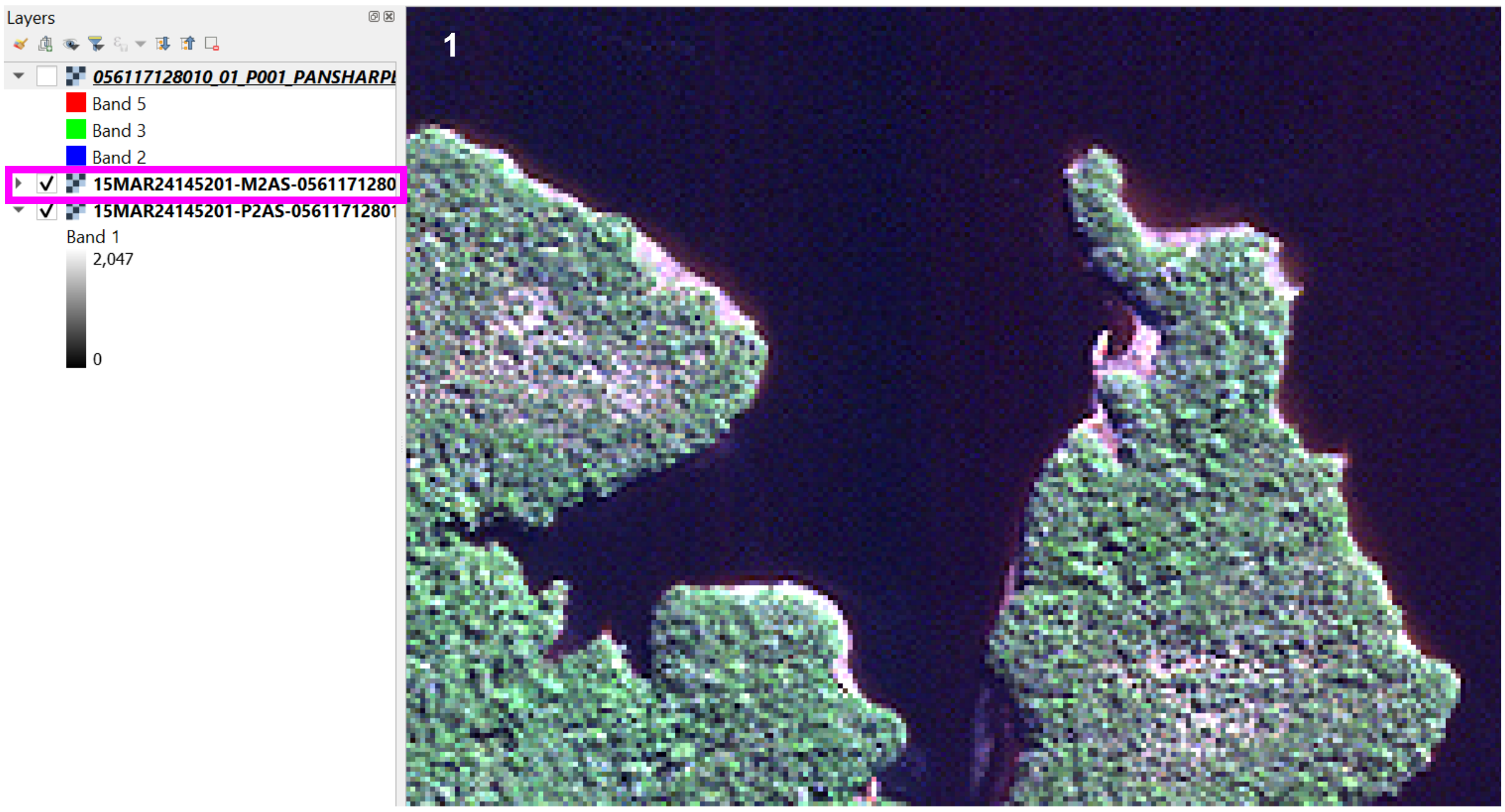

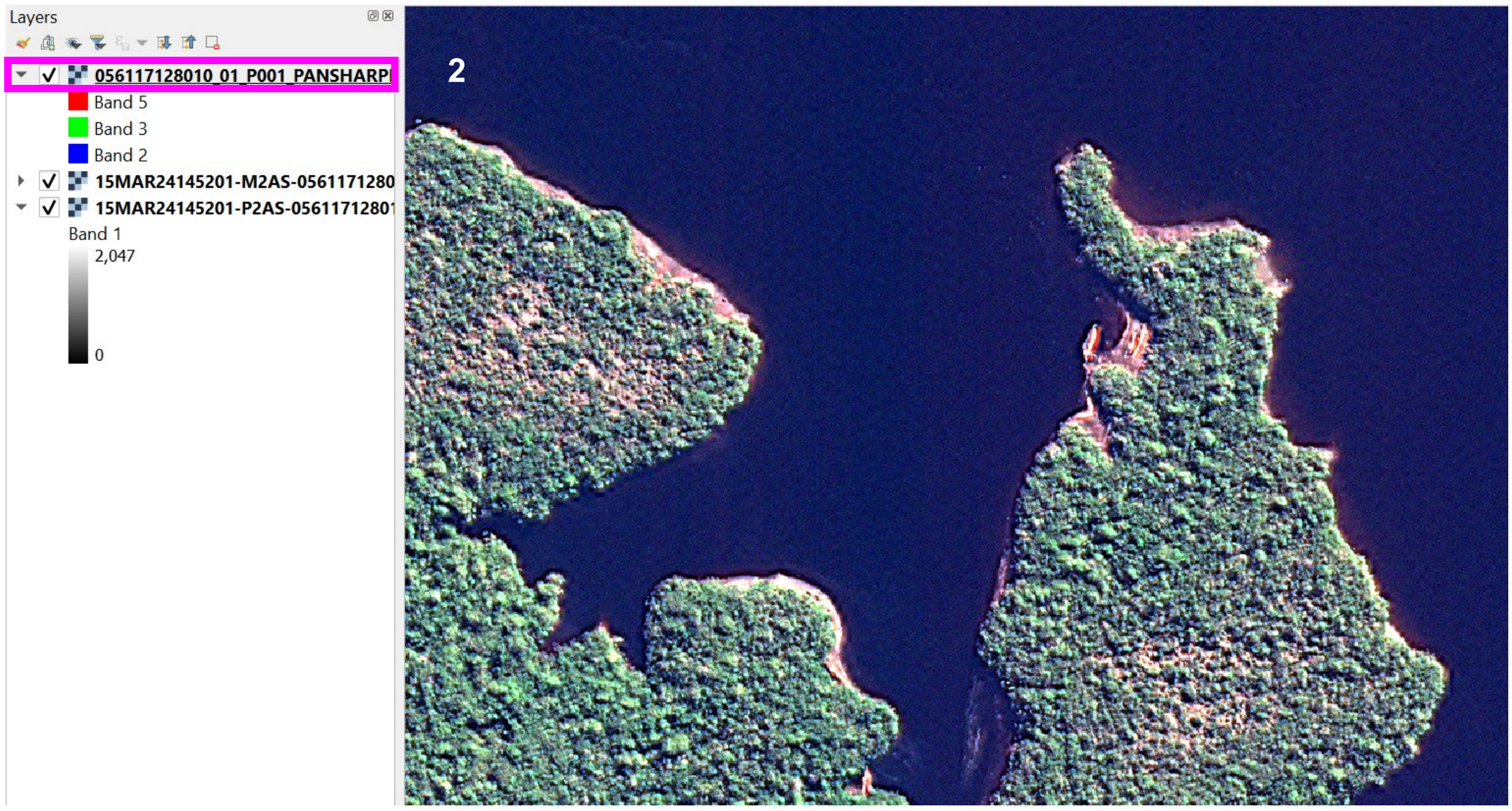

Layers
056117128010_01_P001_PANSHARP
Band 5
Band 3
Band 2
15MAR24145201-M2AS-0561171280
15MAR24145201-P2AS-0561171280
Band 1
2,047
0
2

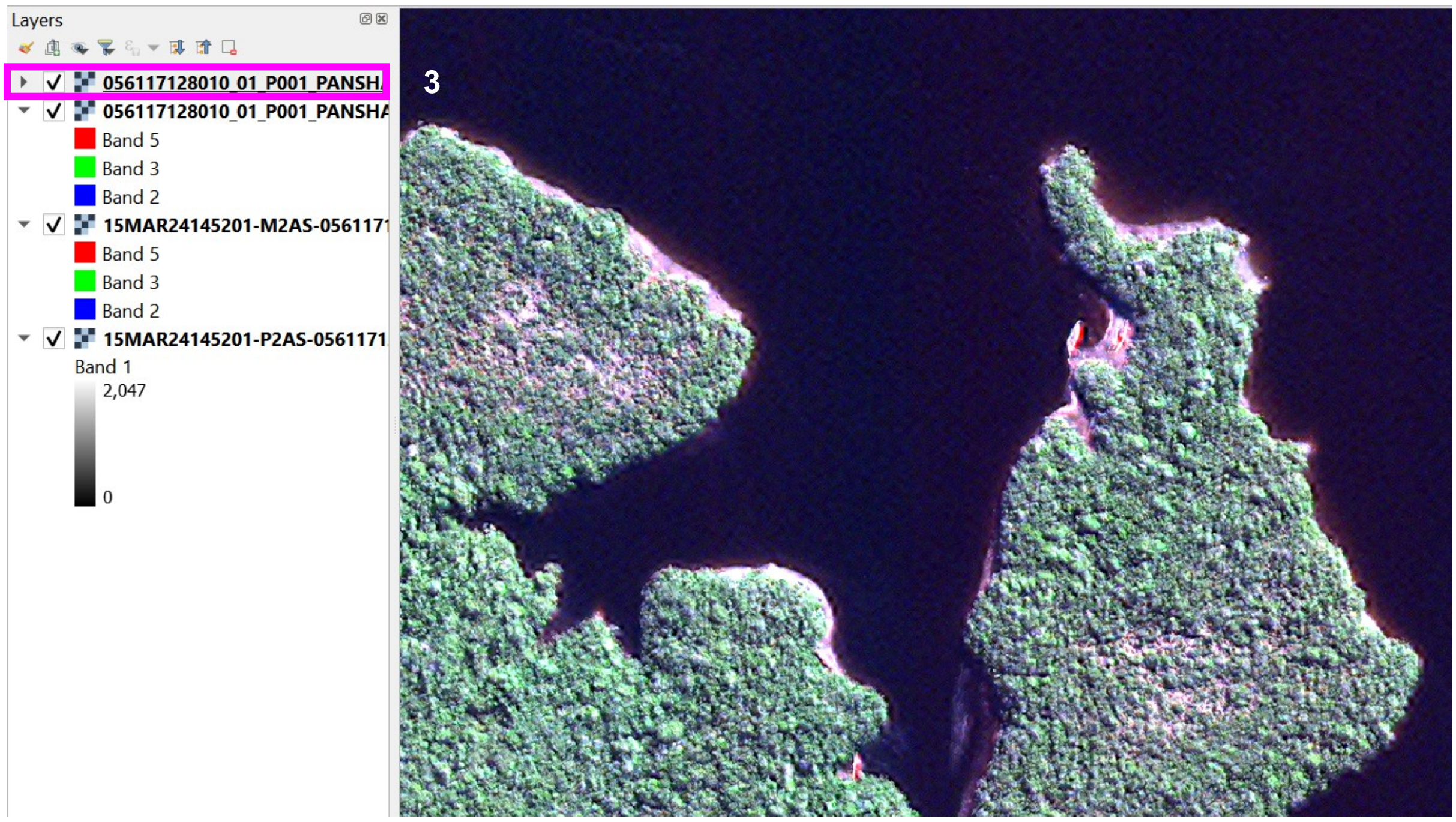

Layers
056117128010_01_P001_PANSH
056117128010_01_P001_PANSHA
Band 5
Band 3
Band 2
15MAR24145201-M2AS-0561171
Band 5
Band 3
Band 2
15MAR24145201-P2AS-0561171
Band 1
2,047
0
3

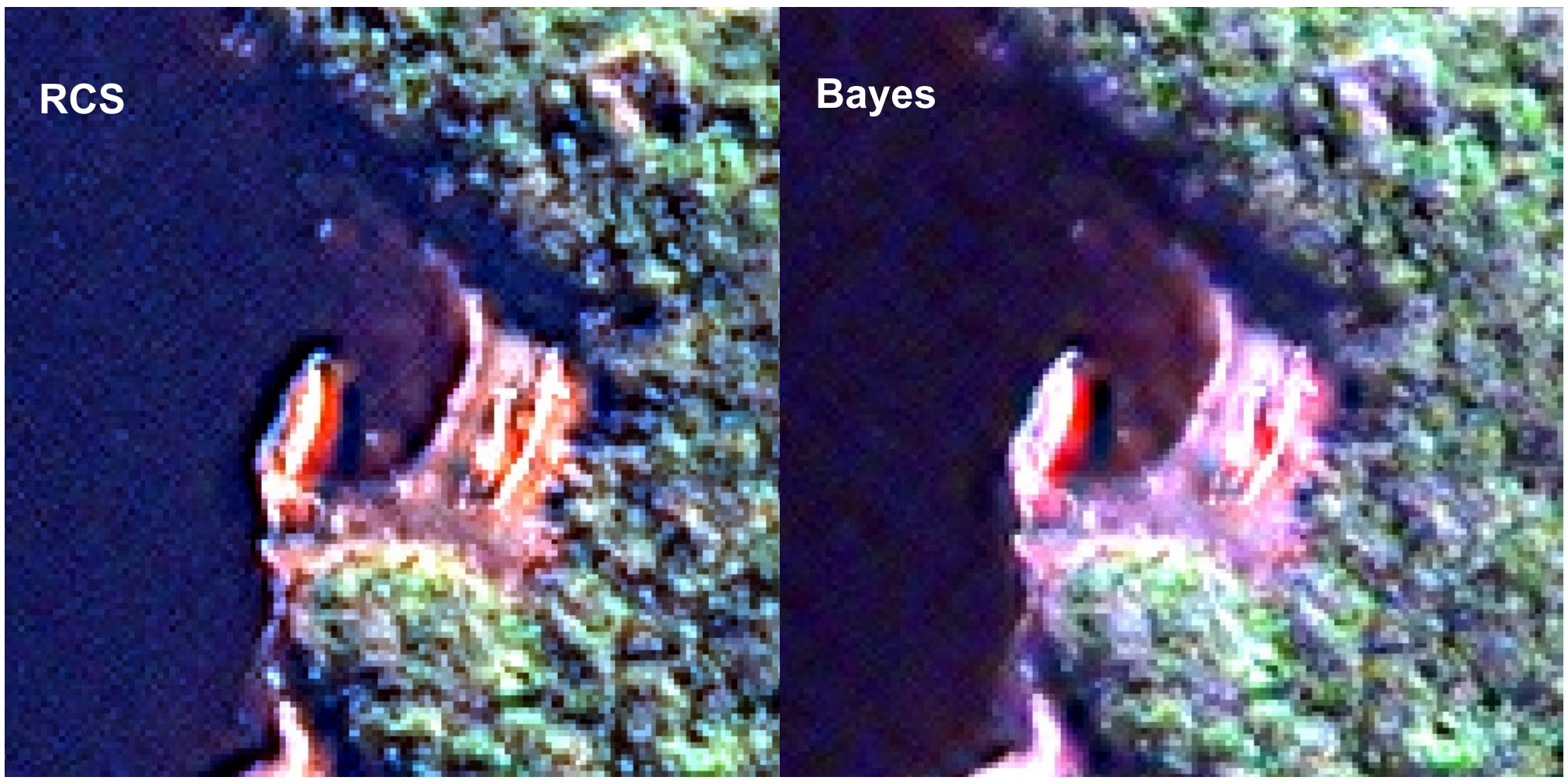

12. Increasing the speed of pansharpening using OTB

Methods can be adopted to speed up the timely pansharpening process (**if not required, continue to section 'Systematic scanning'**), these include:

a. Increase 'Max Threads'

This increases the number of tasks your computer can process at once (it will depend on the computer, though generally this value should be close to the number of cores your computer has).

i. open the 'Processing Toolbox', using the spanner icon ('Options'):

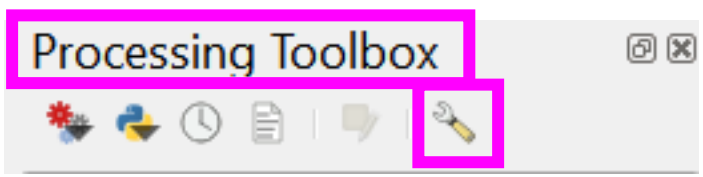

ii. select 'Processing' on the left-hand menu and then 'General' in the sub-menu
iii. manipulate/increase 'Max Threads'

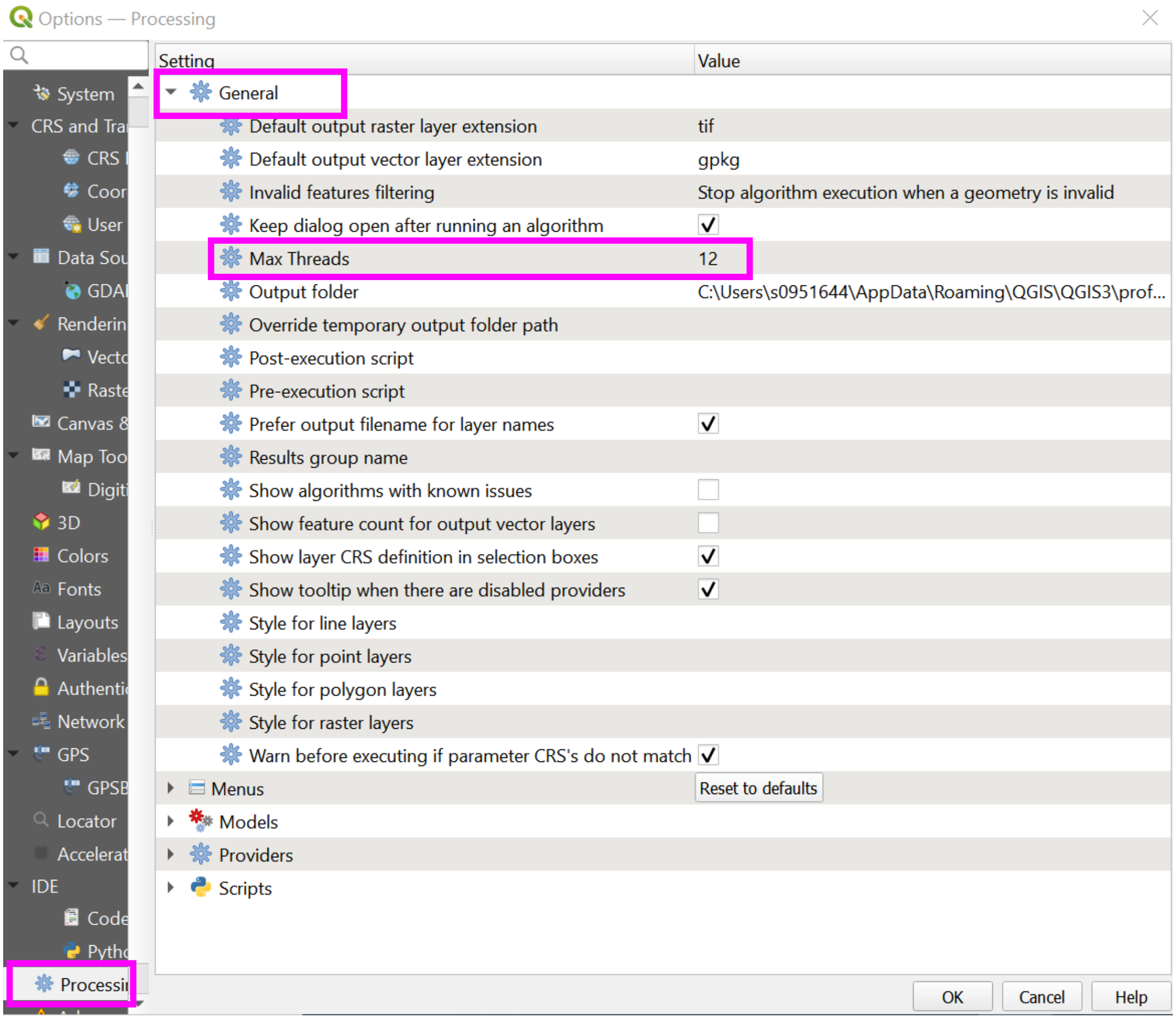

b. Clip raster by extent

GDAL 'Clip raster by extent' is powerful when only wishing to use a certain area within a large image.

i. from the main toolbar select 'Processing', open the 'Processing Toolbox' > 'GDAL' > 'Raster extraction' > 'Clip raster by extent'

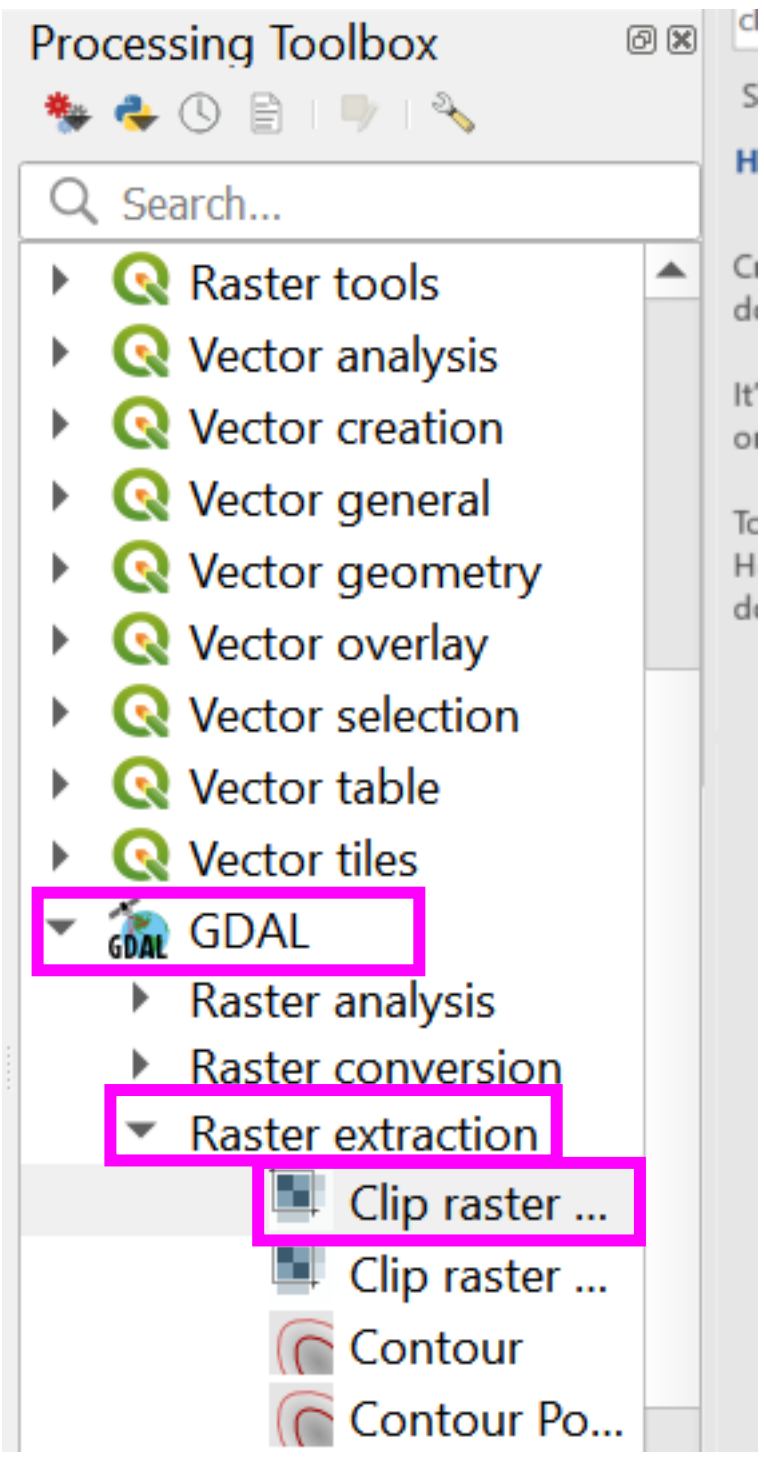

ii. select the multispectral image as the ‘Input layer’, define the ‘Clipping extent’ by clicking the arrow to the right ‘Clipping extent’ and select ‘Draw on Map Canvas’. The ‘Clip by Raster Extent’ window will temporarily close, allowing an area to be defined. Click and drag the extent to form a square/rectangle area, once an area is defined, release the mouse and the ‘Clip by Raster Extent’ window will reappear with the bounding box coordinates of the defined area

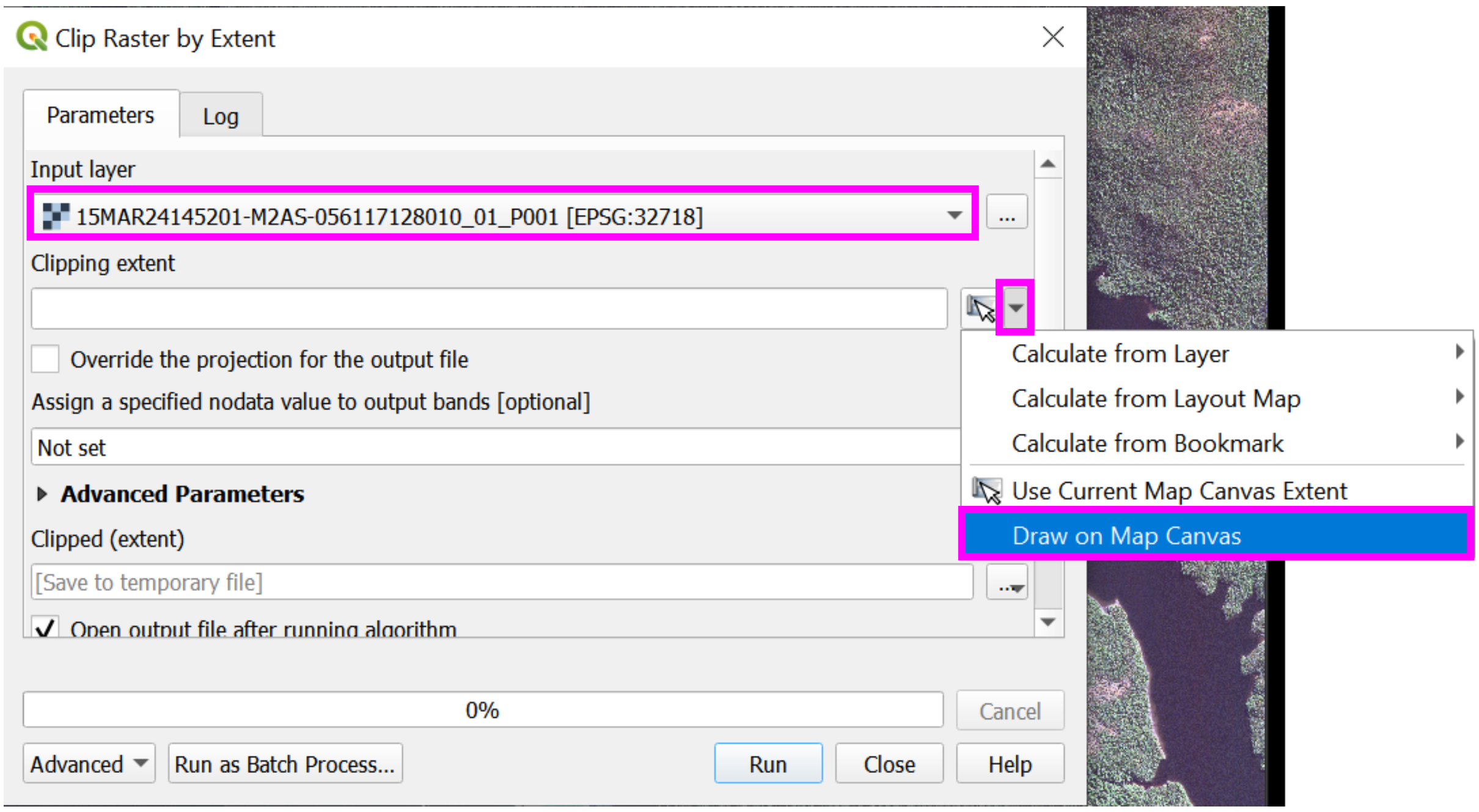

iii. for the ‘Clipped (extent)’ select a location and file name to save the output and ‘Run’

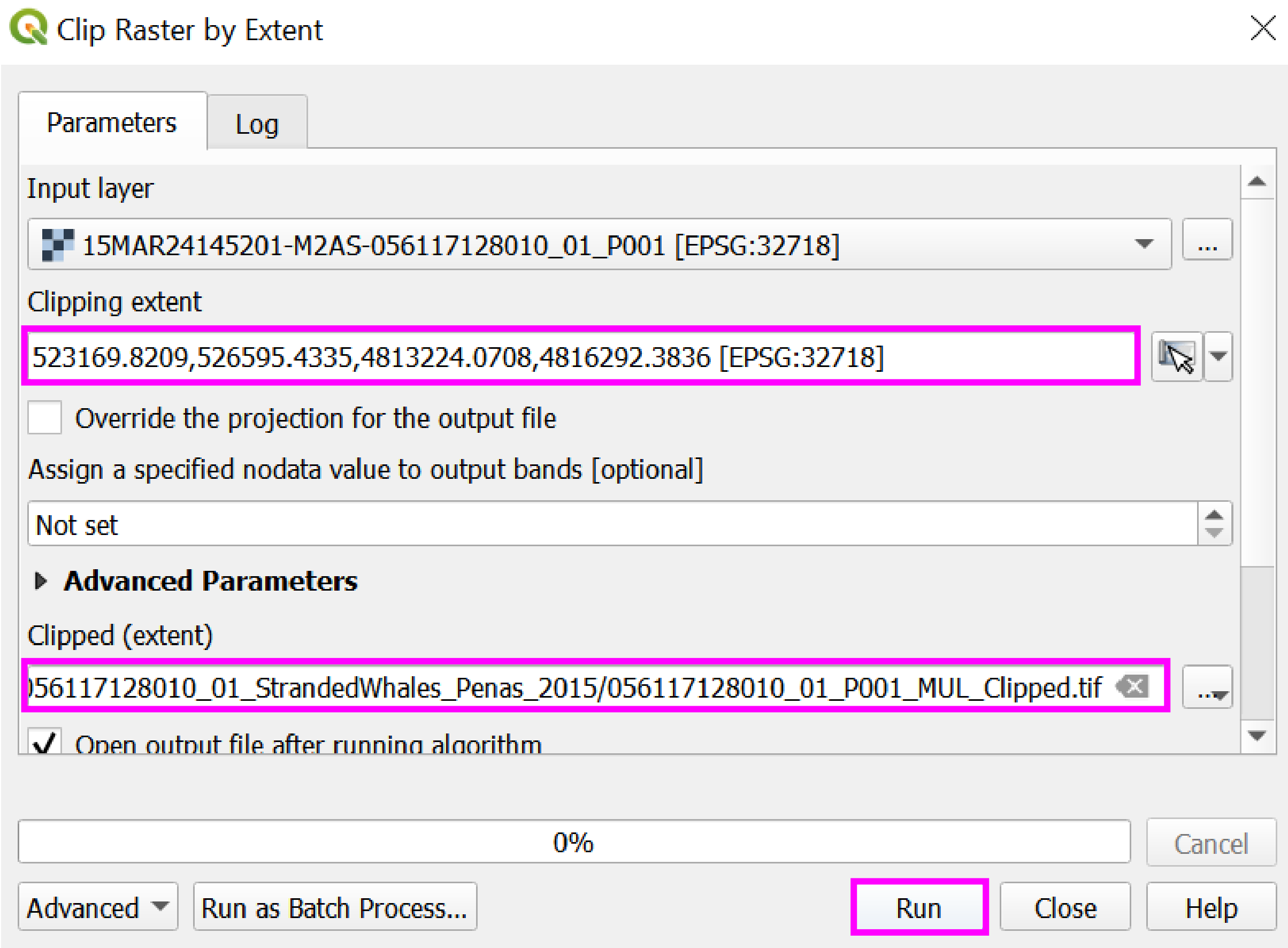

iv. once the clip is complete, reselect the ‘Parameters’ tab, amend the ‘Input layer’ to the panchromatic image, and repeat (ensure to change the file name in the ‘Clipped (extent)’ output)

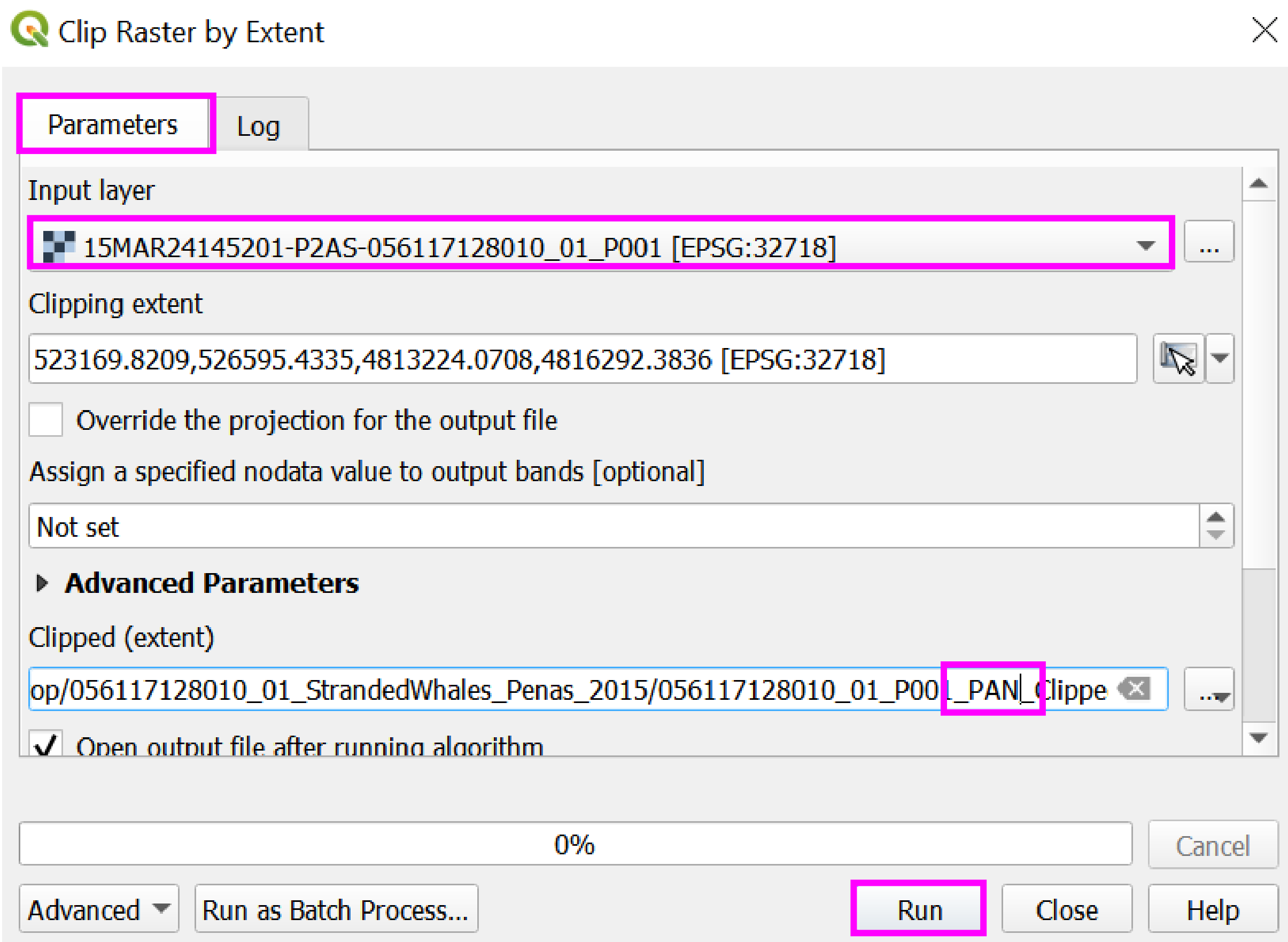

v. the clipped extents can then be used to perform pansharpening, following the steps in section 'Pansharpening using OTB'

vi. 'Bayes' algorithm previously took 4829.19 seconds (1 hour 20 minutes 29 seconds), the new clipped extents include a multispectral file 40.11MB and a panchromatic file 80.23MB, with an approximate area of 10km^2, the algorithm ran in 90.11 seconds (1 minute 30 seconds)

c. Clip raster by mask - Coast shapefile (two methods)

A coast shapefile can be useful in the case of strandings detection, to clip an image to the area of most concern, the coast. The coast can either be defined manually, by creating a line shapefile and digitising the coast by hand, or using freely available online global coastal shapefiles, such as those available from https://osmdata.openstreetmap.de/data/coastlines.html

i. Method 1: Creating a manually digitised coastline

- create a new shapefile by selecting 'Layer' > 'Create Layer' > 'New Shapefile Layer'

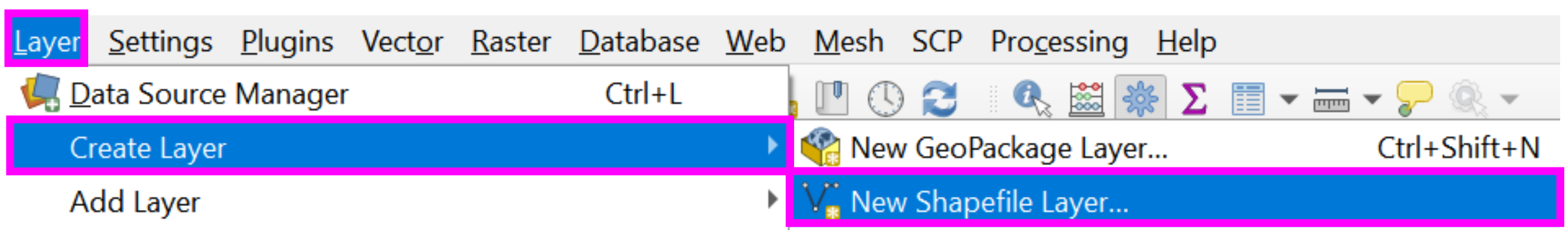

- define the 'File name', set the 'Geometry type' to 'LineString' and set the projection under 'Additional dimensions' to the CRS for the project, then select 'OK'

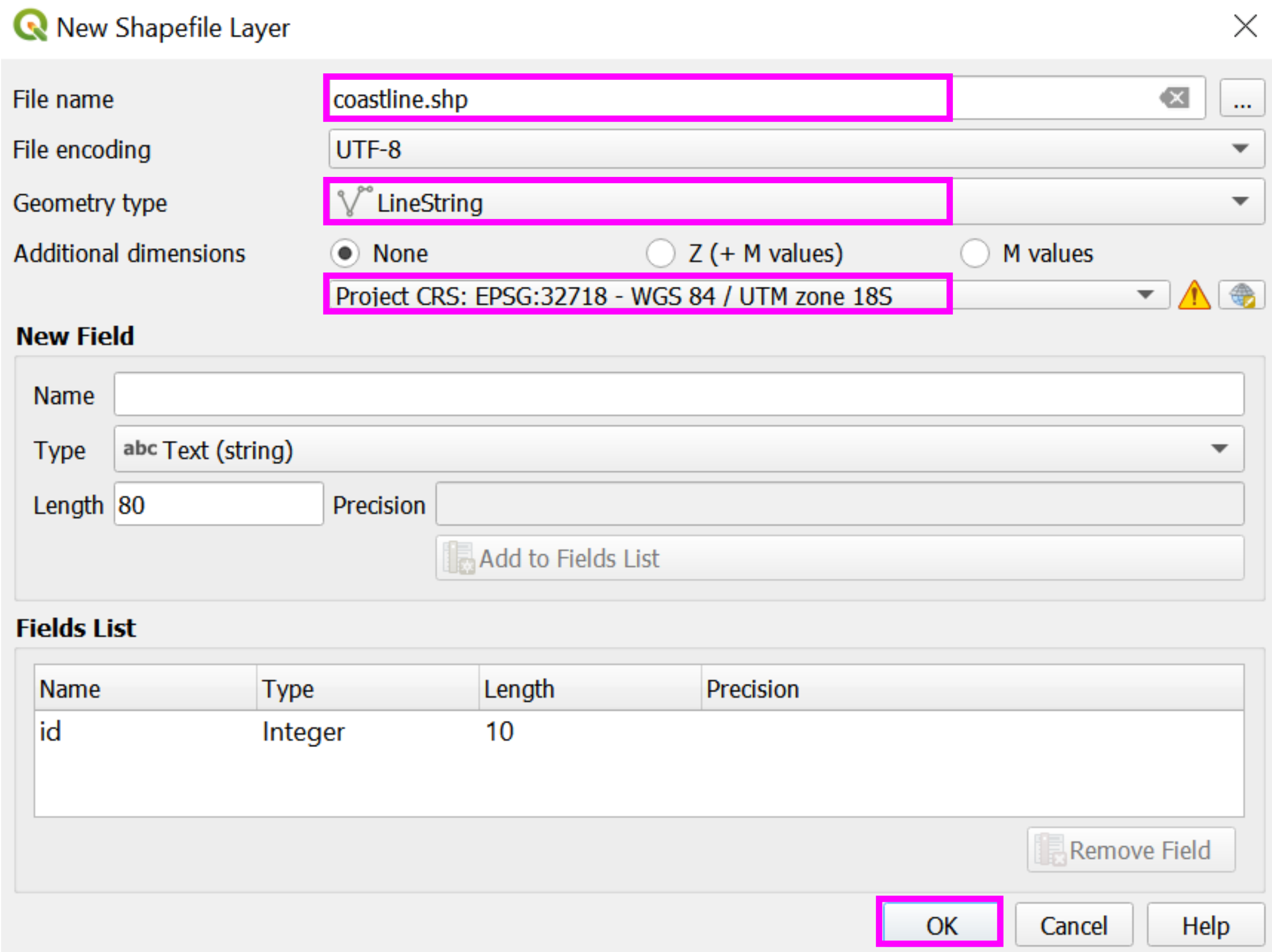

- the line colour can be amended similar to the band combinations in raster layer, by right clicking the shapefile layer in the 'Layers' pane > 'Properties' > 'Symbology', amend the 'Color' and 'Width', then select 'OK'

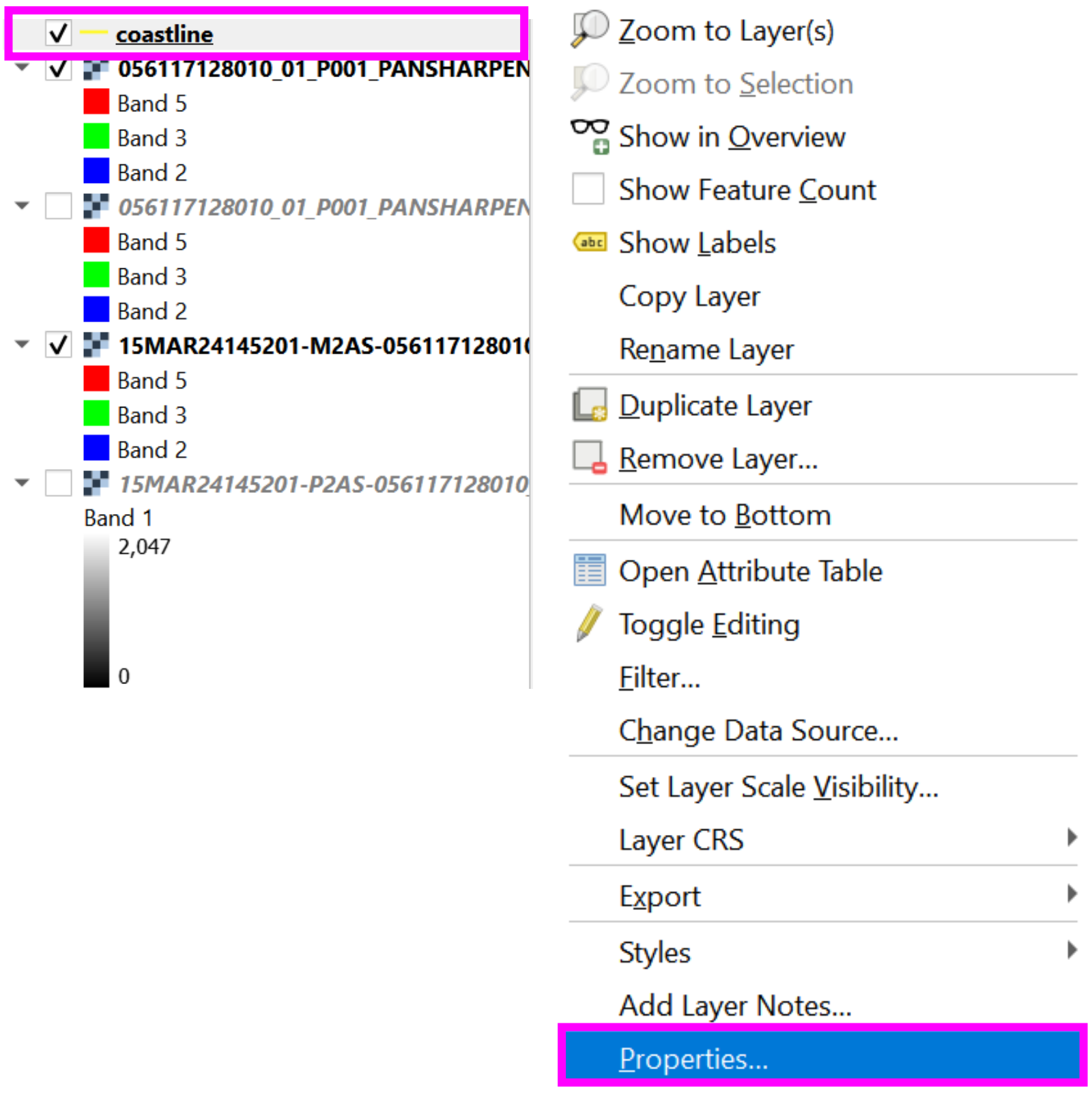

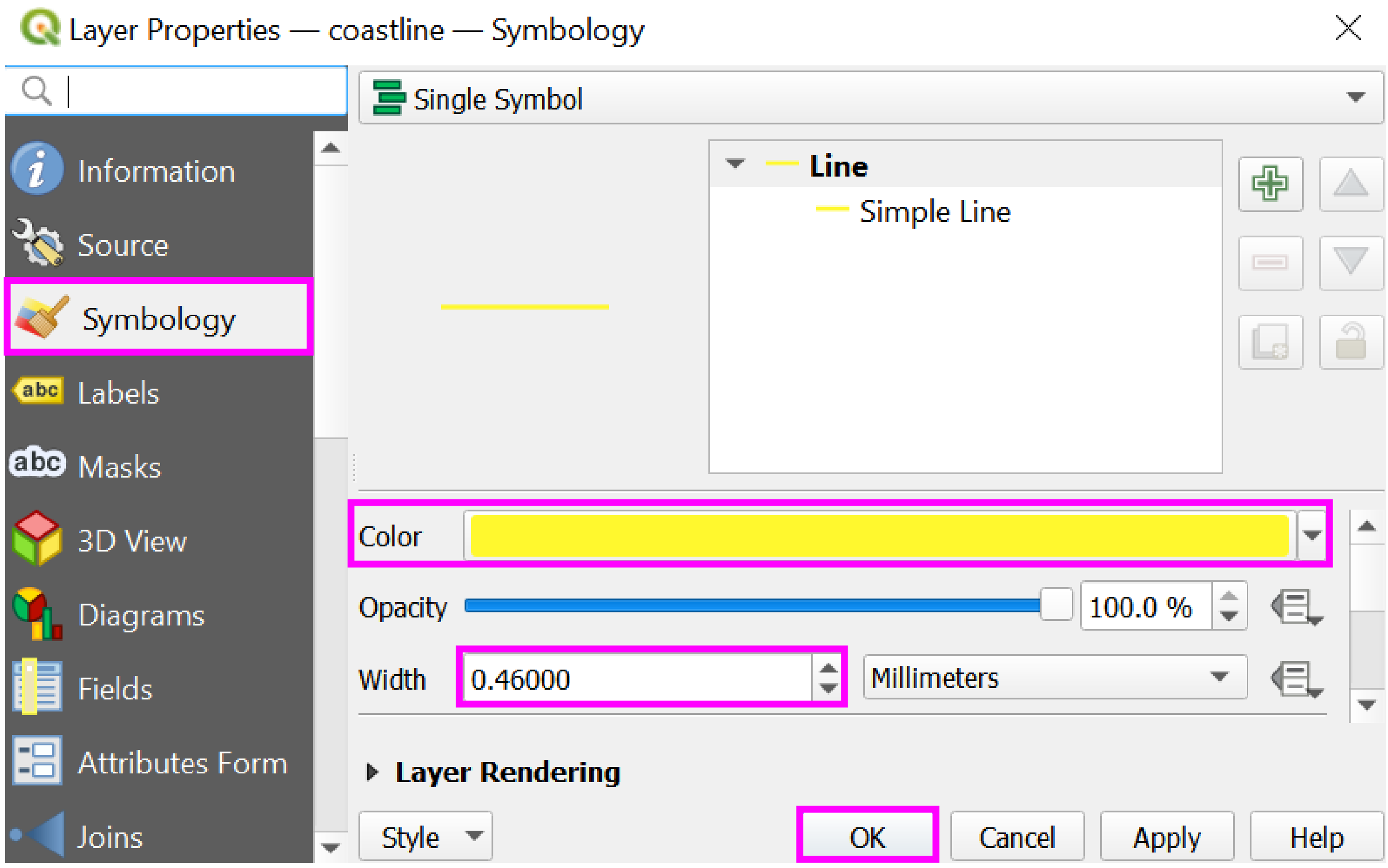

- begin drawing a line feature, by selecting the 'Toggle Editing' icon (yellow pencil), followed by the 'Add Line Feature' icon (green line with vertices)

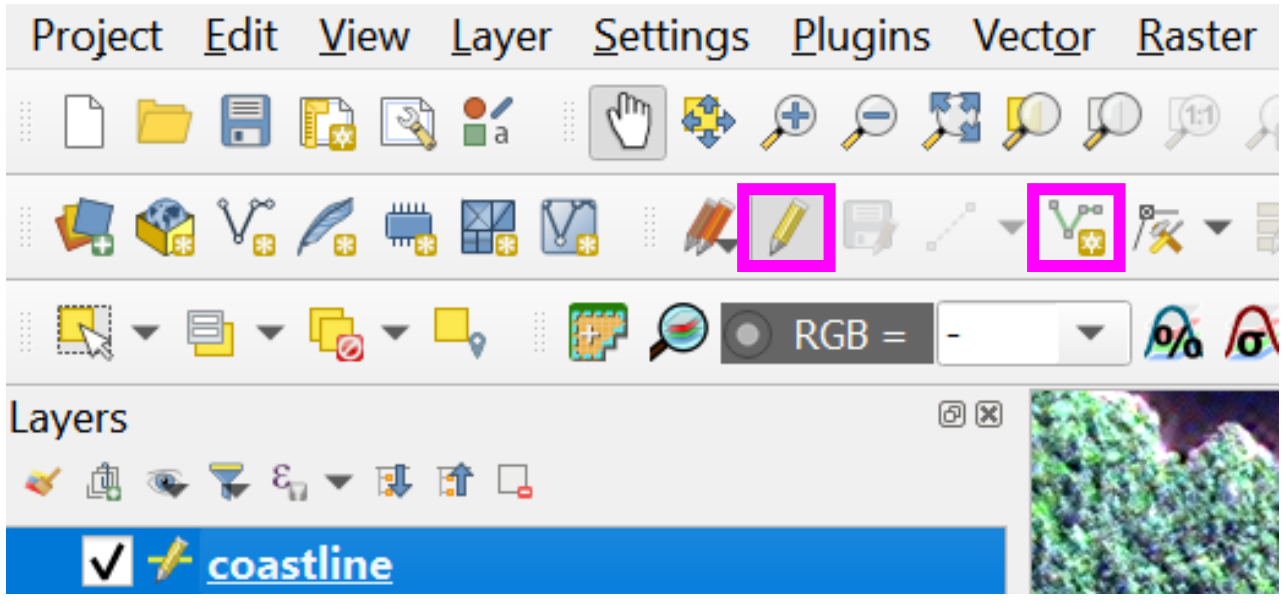

- click in the main QGIS window to begin drawing the line, once all vertices are complete, right click to finish. Add a numeric 'id' for the newly added feature and select 'OK'

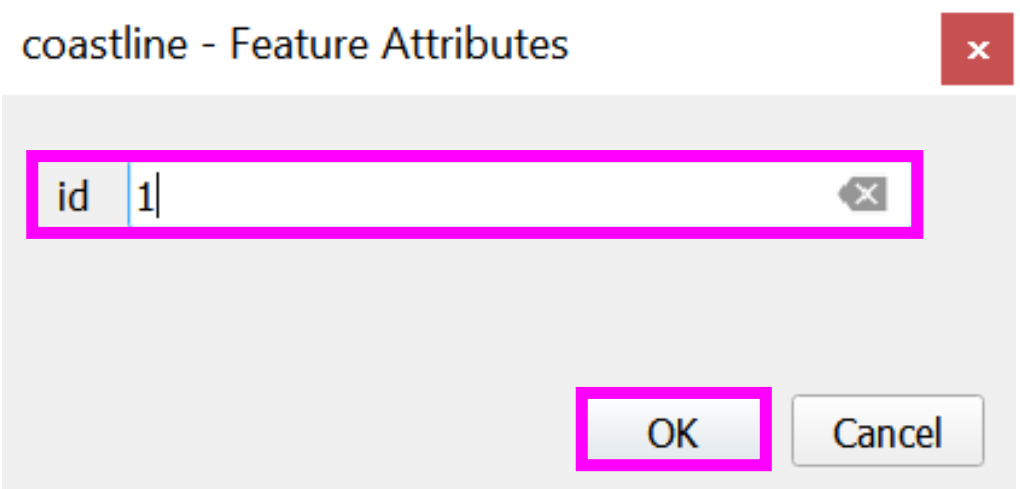

- the line feature will appear in the main window, click 'Save Layer Edits' on the 'Toogle Editing' toolbar and reselect the 'Toogle Editing' icon to end an editing session

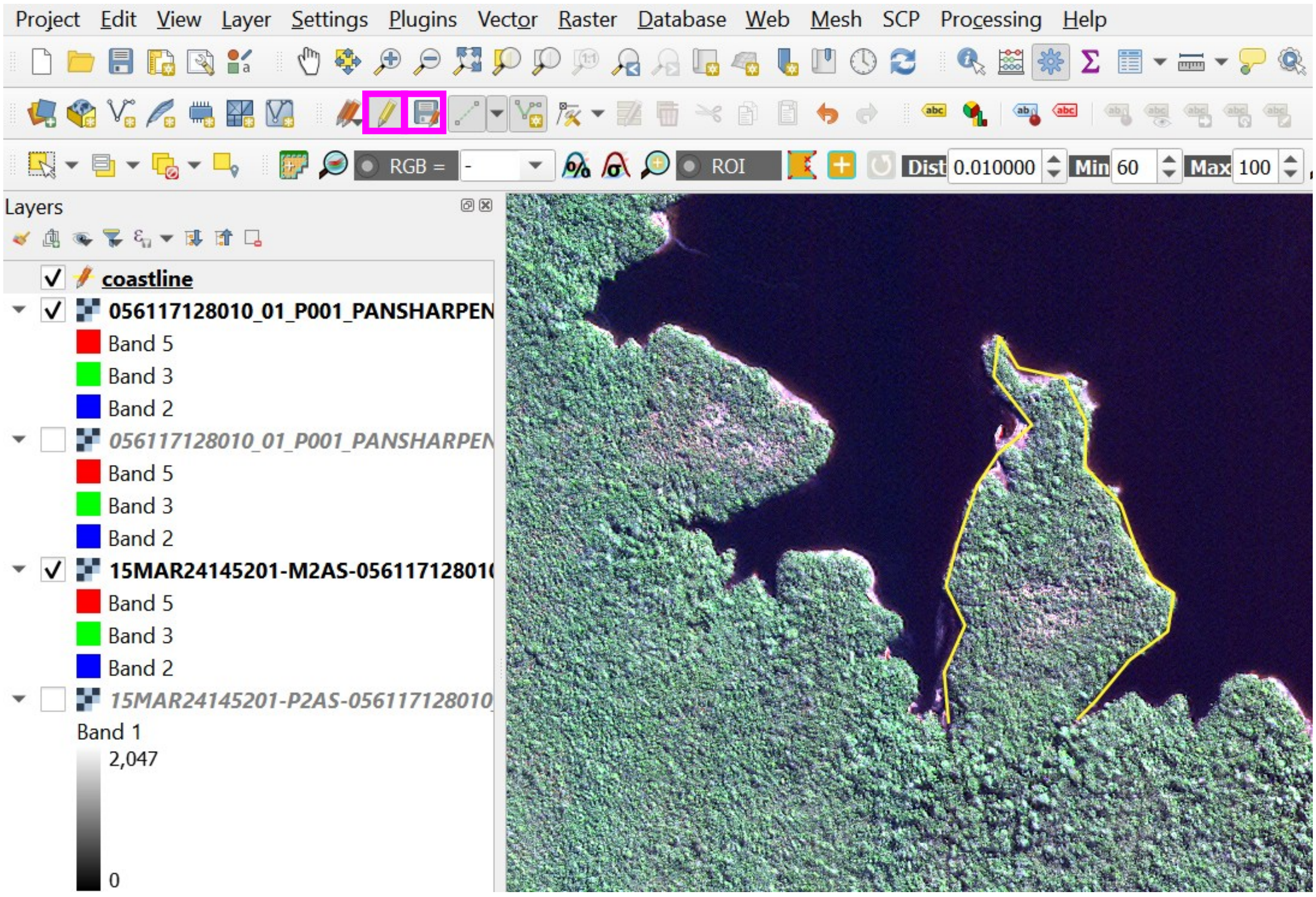

- continue to section 'creating a mask', to create a buffer around the 'coastline.shp', and clip raster by mask. Else, if using the freely available online coastline shapefile follow the below guidance before

moving to section ‘creating a mask’. If you wish to amend your line shapefile, view the how to in section ‘editing a line shapefile’

ii. Method 2: Create a coastline using a freely available online coastline shapefile

Manually digitising a coastline is timely, freely available online coastline shapefiles offer a solution to digitising the coast by hand. These files need to be used with caution, as they may be too coarse to provide an accurate outline of your coast.

- visit https://osmdata.openstreetmap.de/data/coastlines.html and download the coastline shapefile in the relevant projection
- right click the downloaded zip file and ‘Extract All…’, save the extracted folder to a suitable location

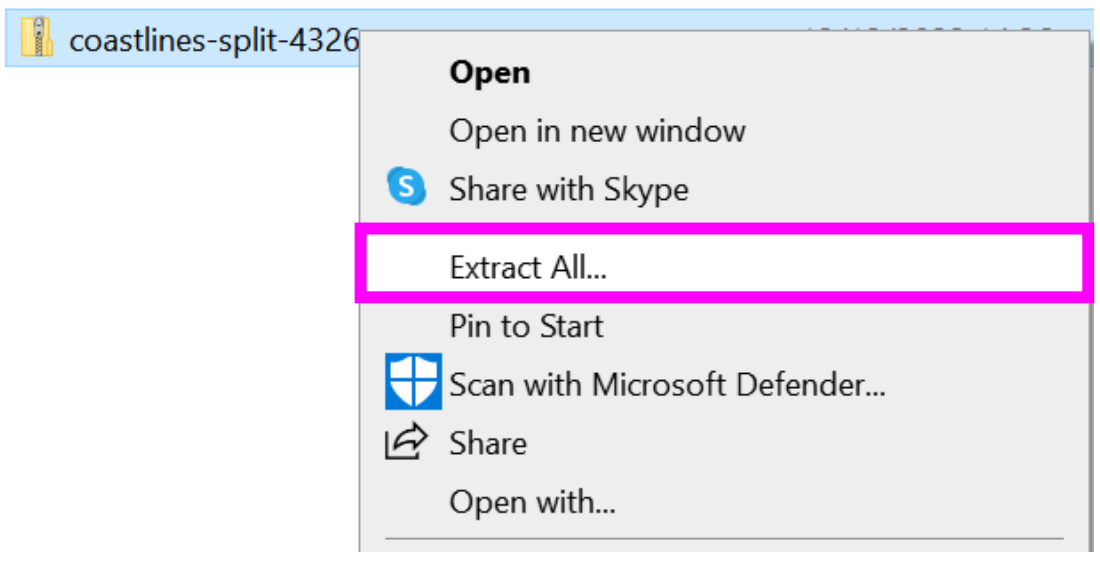

- from the newly extracted folder, drag and drop the ‘lines.shp’ shapefile into the QGIS ‘Layers’ pane

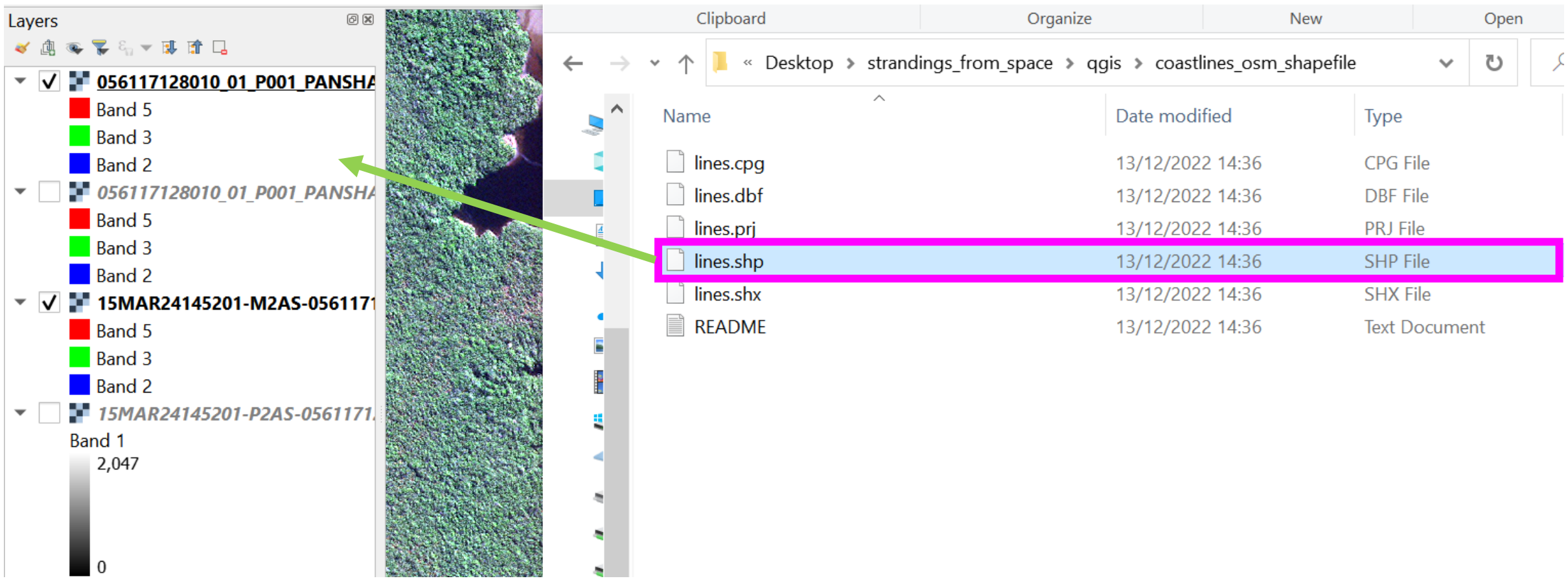

- to amend the line colour, right click the ‘lines’ layer in the ‘Layers’ pane > ‘Properties’ > ‘Symbology’ > ‘Color’, like section ‘Method 1: Creating a manually digitised coastline’.

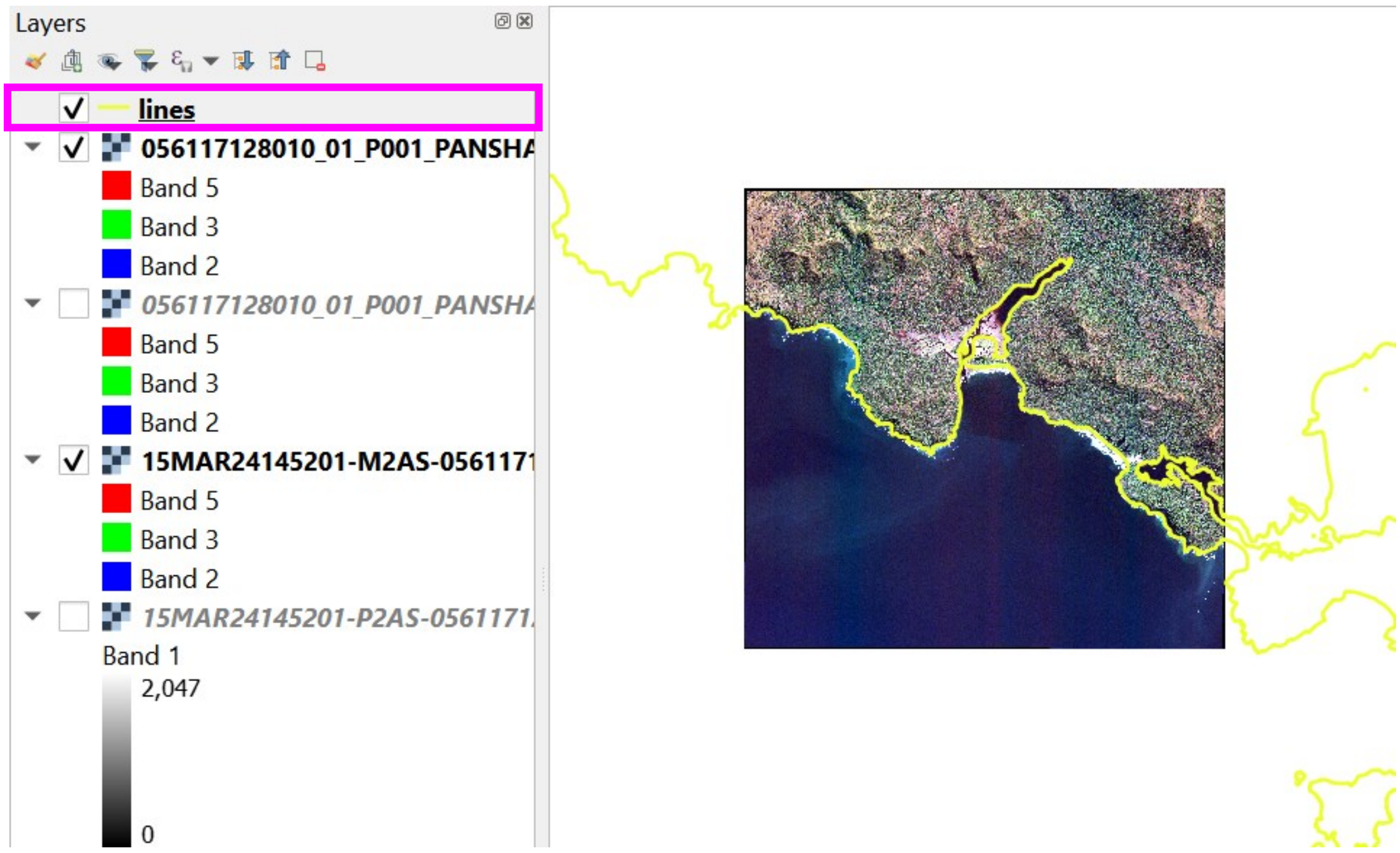

- the ‘lines.shp’ shapefile is the global coastline, to create a mask for your image location, best practice would be to clip the ‘lines.shp’ shapefile to your location only. On the main toolbar, open the ‘Processing Toolbox’ > ‘GDAL’ > ‘Vector geoprocessing’ > ‘Clip vector by extent’

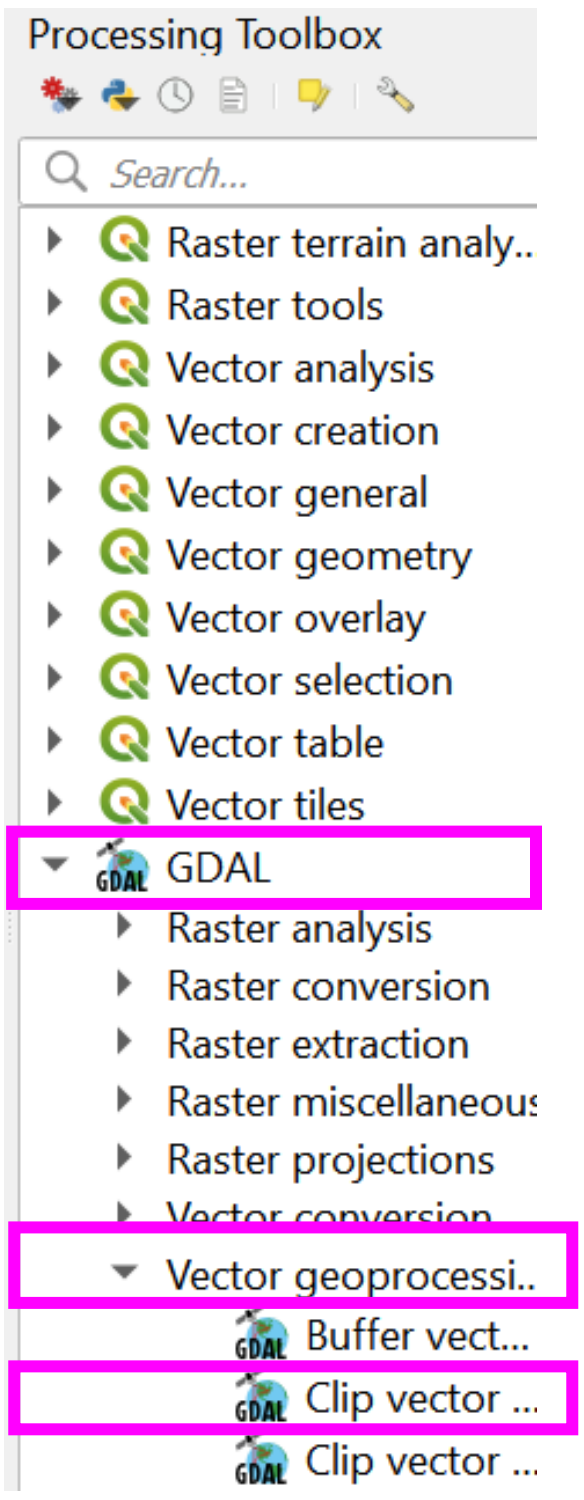

- select the ‘Input layer’ as the ‘lines’ shapefile, define the ‘Clipping extent’ by selecting the drop-down arrow > ‘Draw on Map Canvas. Limit the coastline shapefile to the image extent by clicking on the project window and dragging until the image is enclosed or select ‘Calculate from layer’, selecting the image of interest.

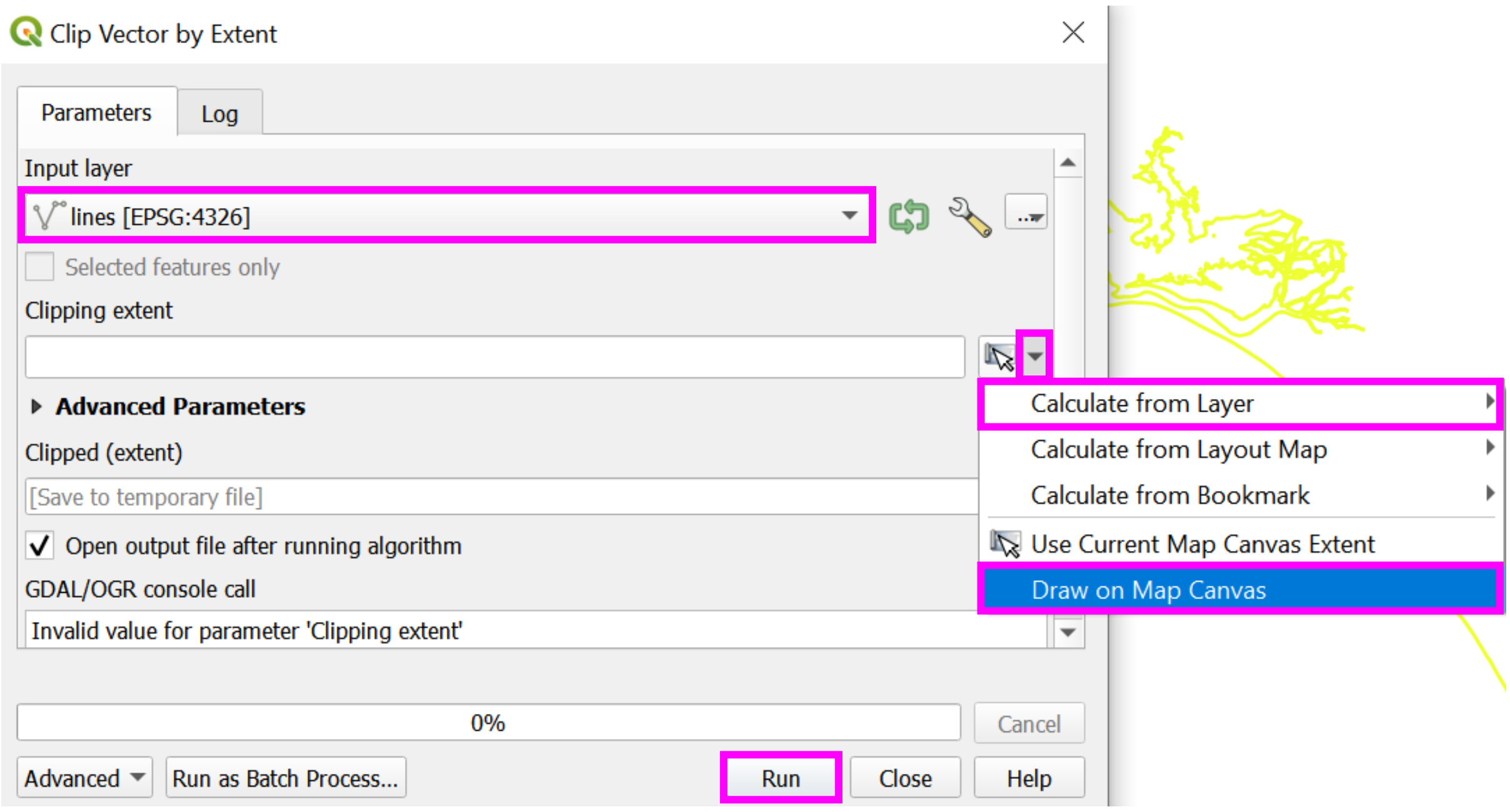

- select a suitable location and file name to save and 'Run'

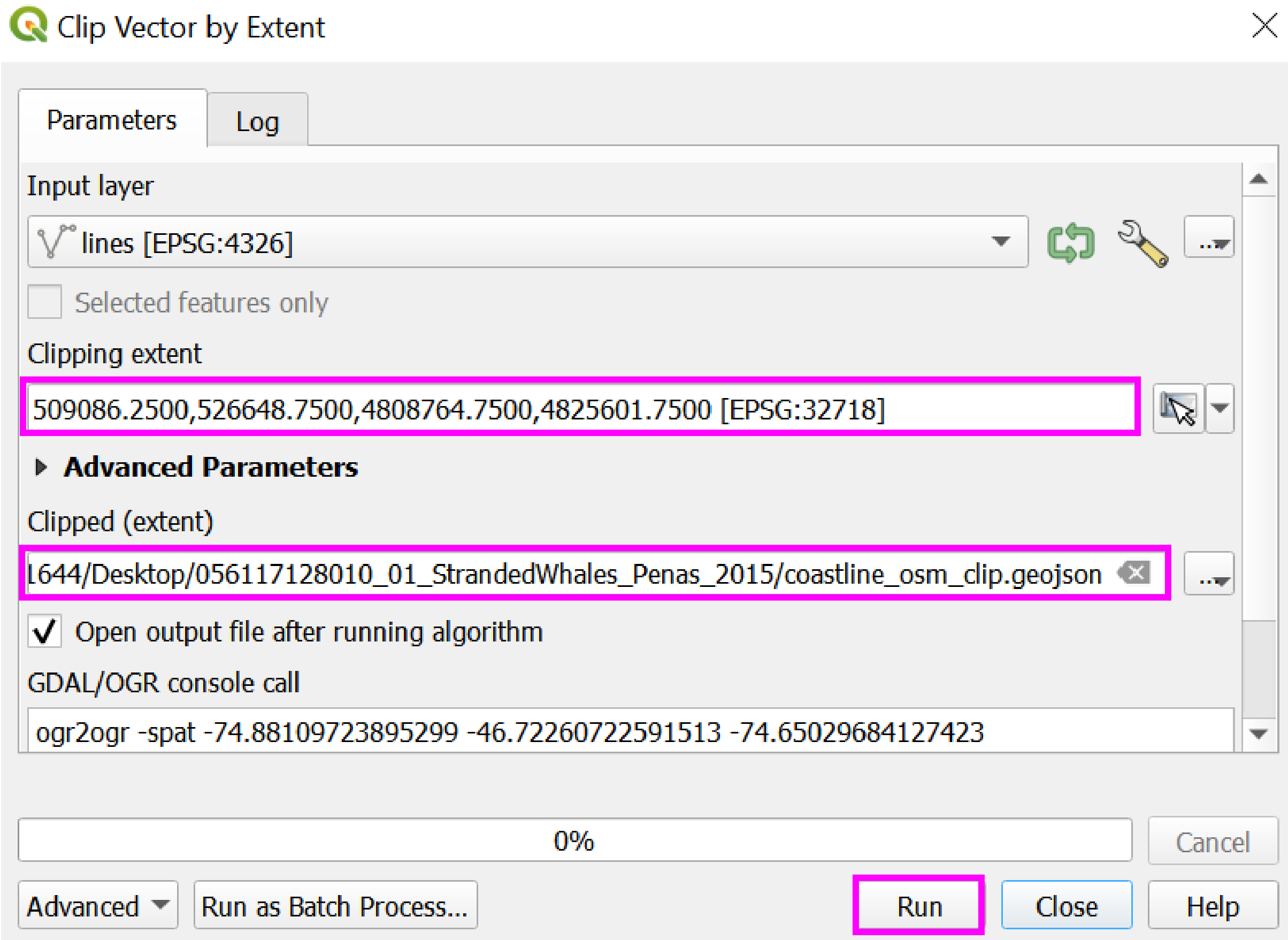

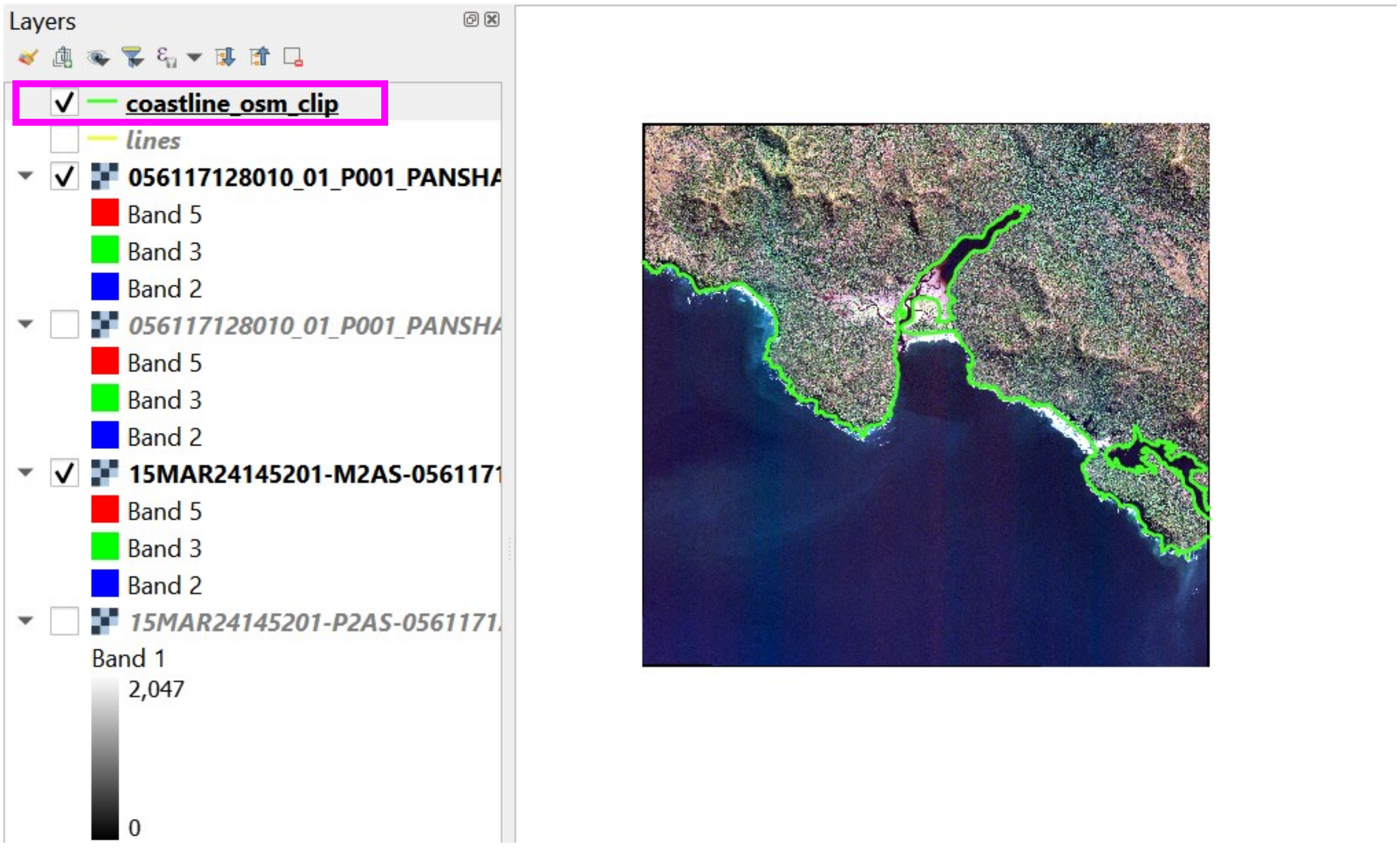

iii. editing a line shapefile

- to adjust your line shapefile (whether manually digitised or from online), while in 'Toggle Editing' mode, select from the toolbar the 'Vertex Tool'. Once selected the vertex (red circles in the image below) should appear on your line when hovering over. To move an individual vertex, click the vertex once and move your mouse to the new location and click again once. To delete a vertex, click the vertex once and press the delete button on your keyboard. Ensure to save after each edit

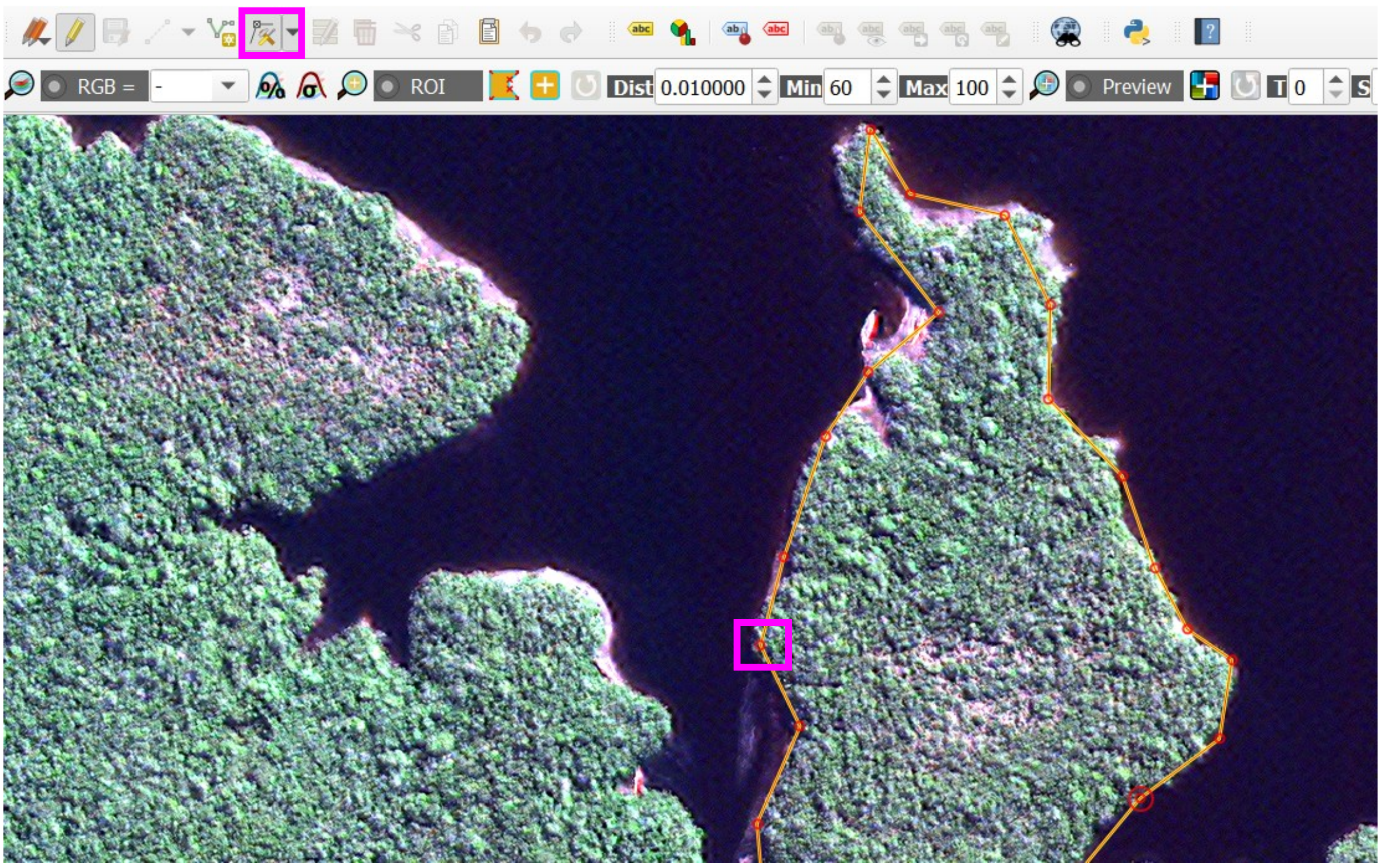

iv. creating a mask

A buffer is required to extract an area around the line shapefile, to form a mask. Therefore, either the manually digitised line shapefile or the imported coastline shapefile will need buffering before clipping the raster.

- press Ctrl + K or in the bottom left of the screen in the search bar, type 'Buffer'. Select the first processing algorithm called 'Buffer'. Alternatively, open the 'Processing Toolbox' > 'Vector geometry' > 'Buffer'

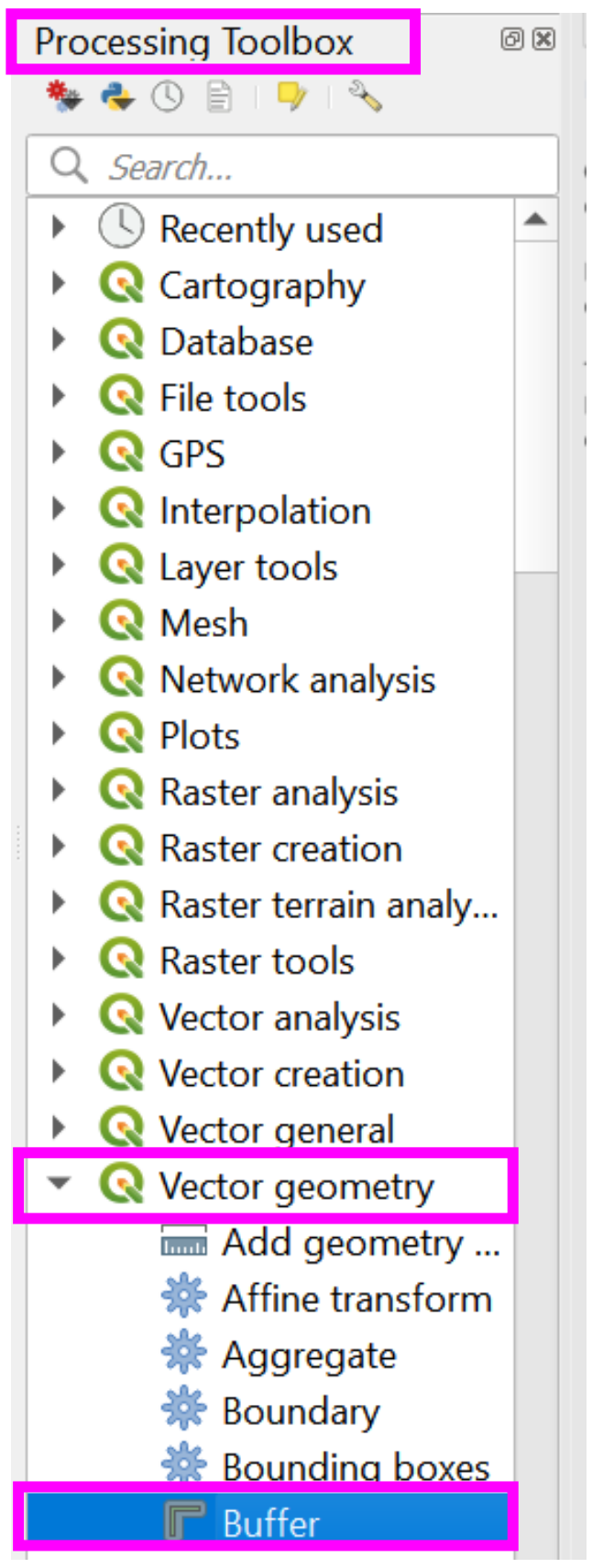

- in the Buffer window, set the 'Input Layer' as your coastline shapefile (manually digitised) or coastline_osm_clip (clipped imported coastline shapefile) you just created. Set the appropriate distance for your buffer around your line feature, in degrees, 0.005 is a good distance to encompass the coast fully

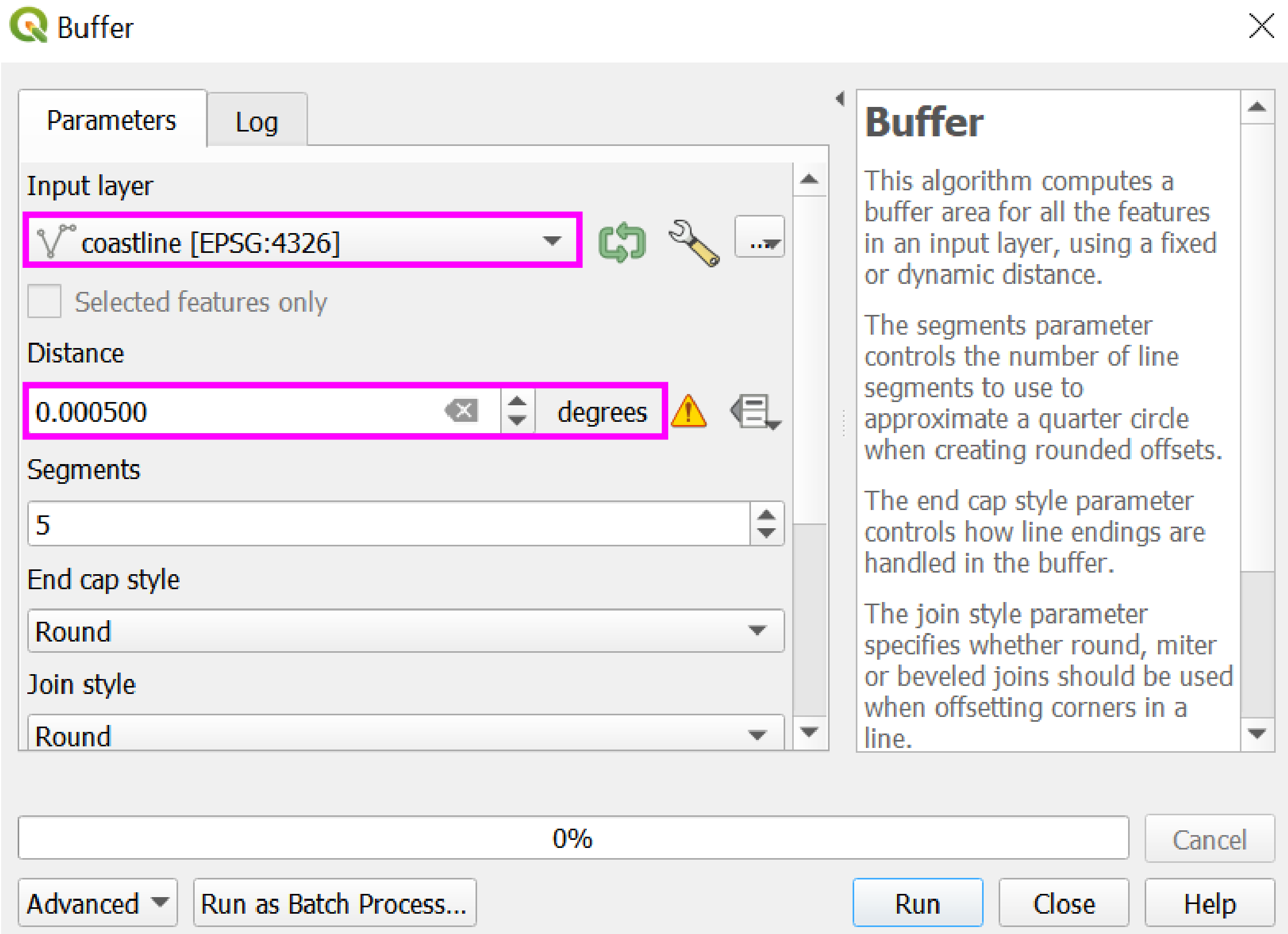

- select a suitable location and file name to save your buffered file and then 'Run'

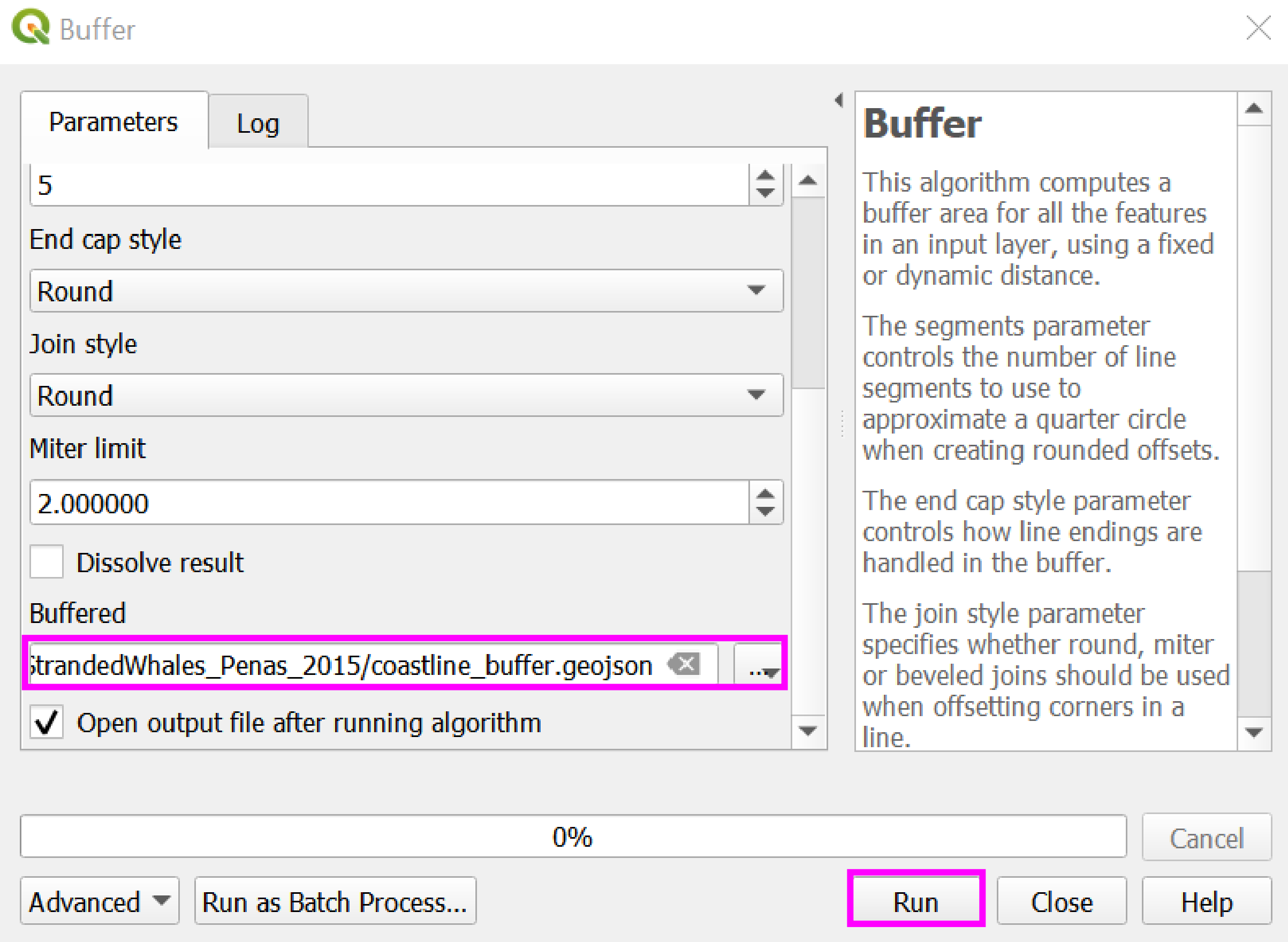

The output will look like the below, where the yellow line has increased in size, creating a new buffer layer (example using the manually digitised coastline shapefile):

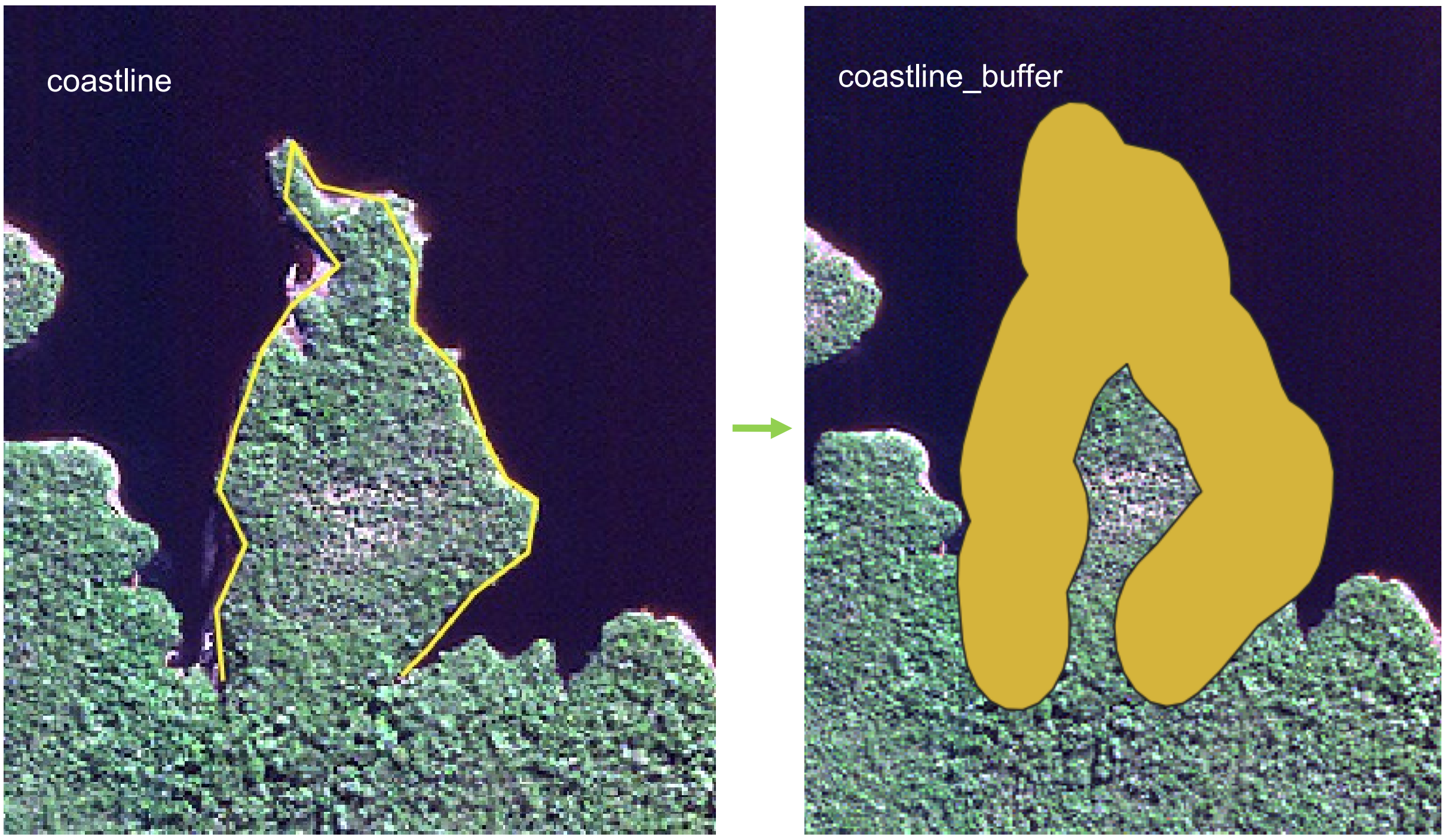

- if after the buffer is created the distance for the buffer is not large enough to encompass the coastline, remove the layer (right click the

layer in the 'Layers' pane and select 'Remove Layer…'), and re-execute the 'creating a mask' steps, adjusting the 'Distance'

v. clipping raster by mask layer

Once the buffer layer is generated, the next step is to complete a raster extraction, to 'Clip raster by mask layer'.

- open the 'Processing Toolbox' > 'GDAL' > 'Raster extraction' > select 'Clip raster by mask layer'
- in the 'Clip raster by mask layer ' window, select the 'Input Layer' as your panchromatic image, the 'Mask Layer' as your buffer layer (*e.g.*, coastline_buffer (manually digitised buffer) or coastline_osm_buffer (downloaded online coastline buffer)), and the 'Source CRS' and 'Target CRS' as the project CRS

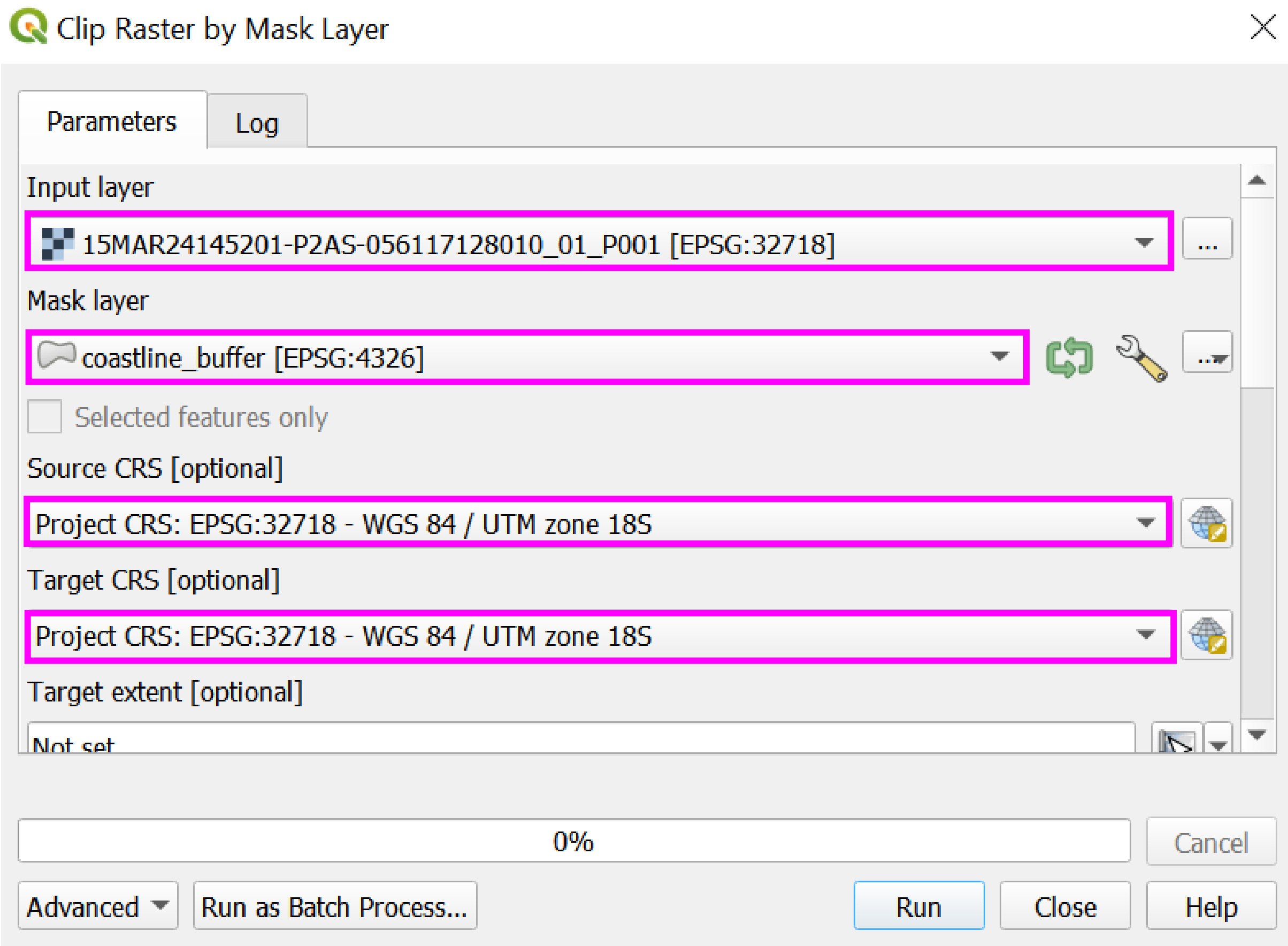

- set 'Assign a specified nodata value to output band [optional]' to for example, '0' or '-999999999.000000' (this enables QGIS to ignore these pixels in continued analysis and the user to easily mask no data values). Click in the cell and scroll upwards using a mouse or click the up arrow to the right of the cell once, to set this value. Check the 'Keep resolution of input raster' box, and keep the 'Match the extent of the clipped raster to the extent of the mask layer' box checked

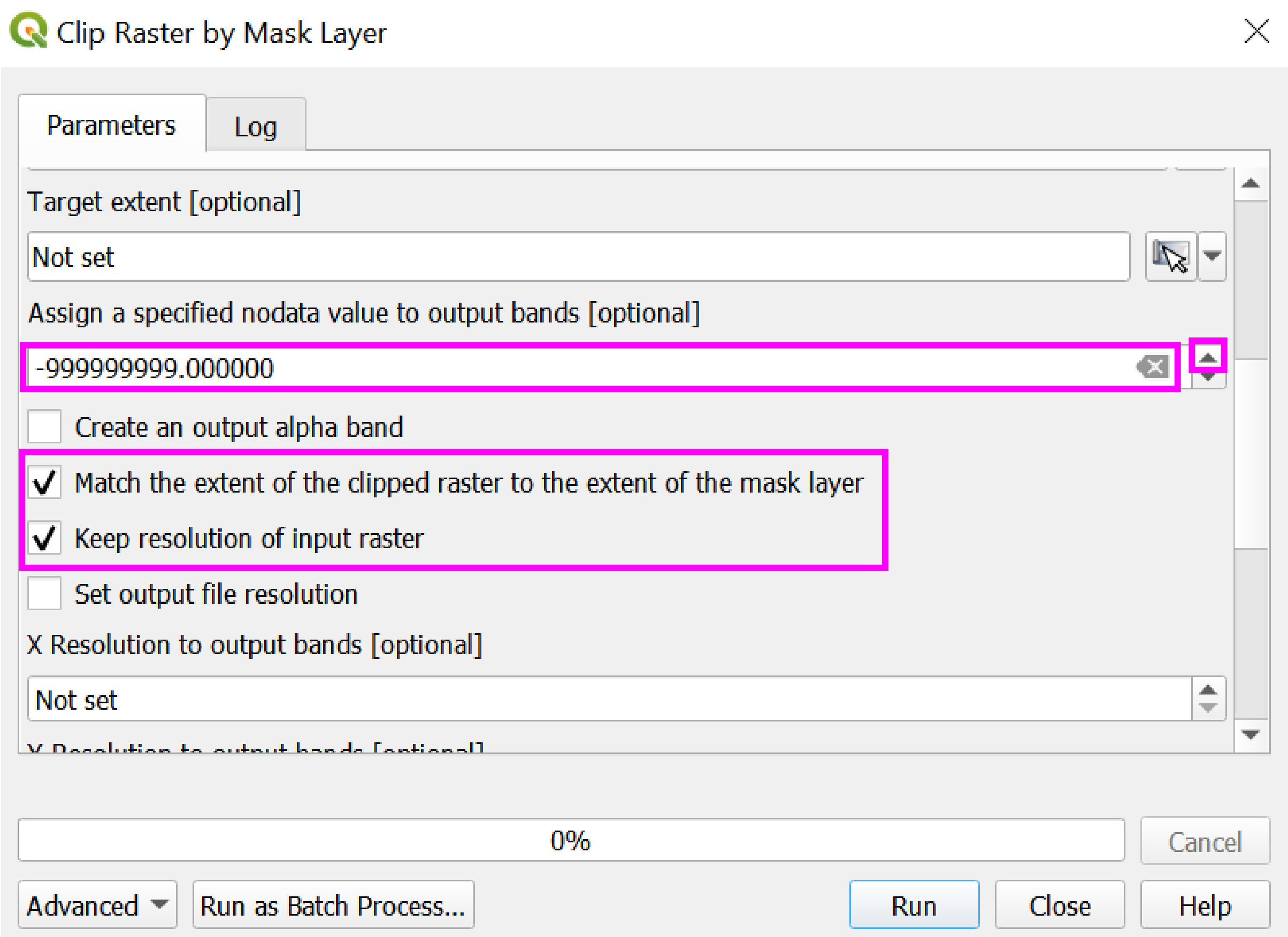

- select a location and suitable file name in the ‘Clipped (extent)’ and ‘Run’

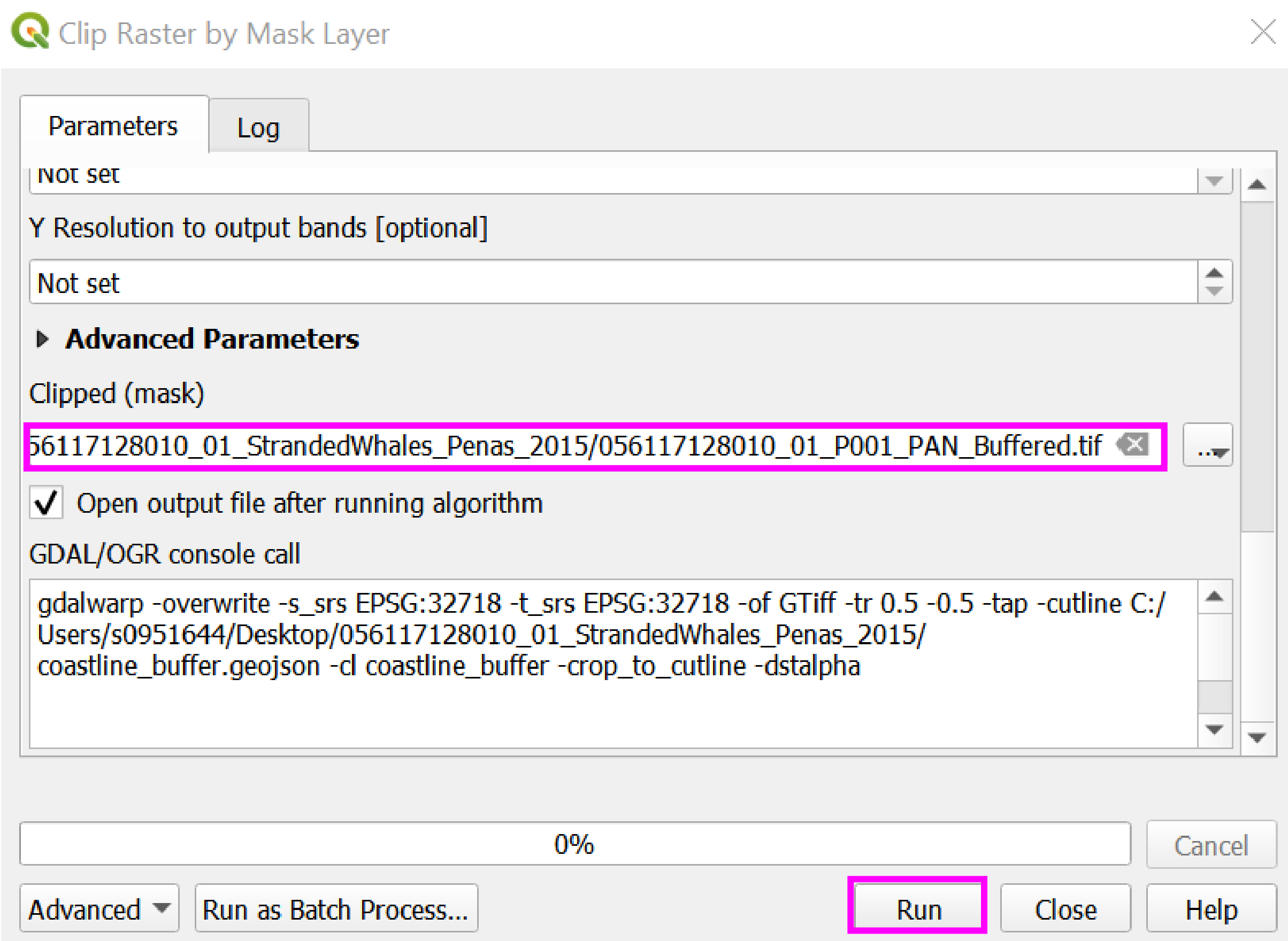

- select the 'Parameter' tab and repeat the 'Clip raster by mask layer' for your multispectral image (ensure to change the file name in the 'Clipped (extent)' output)

The outputs will look similar to the below:

Clipped multispectral image:
manually digitised shapefile

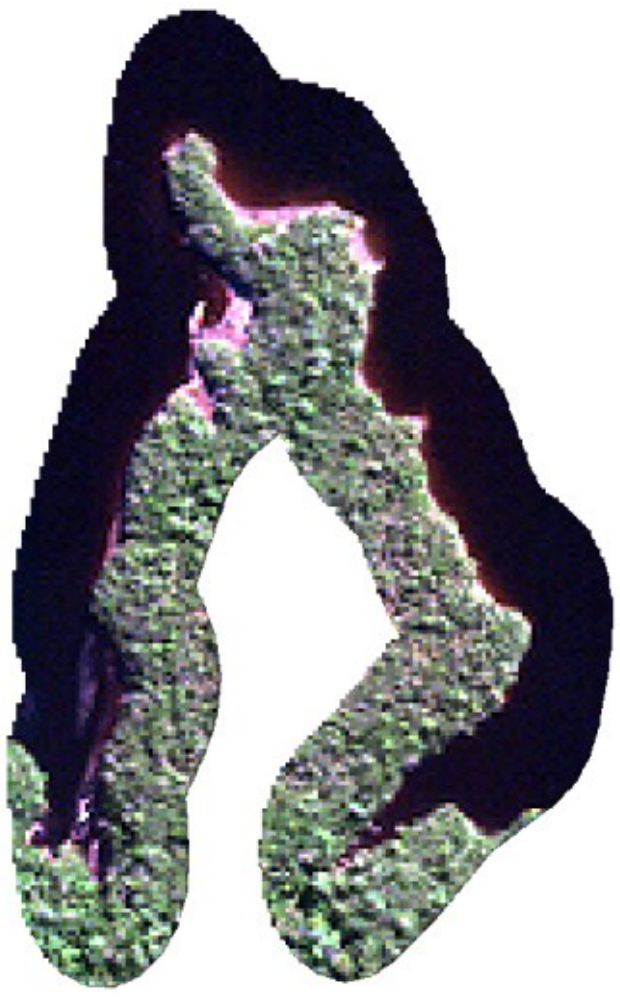

Clipped multispectral image:
Downloaded online shapefile

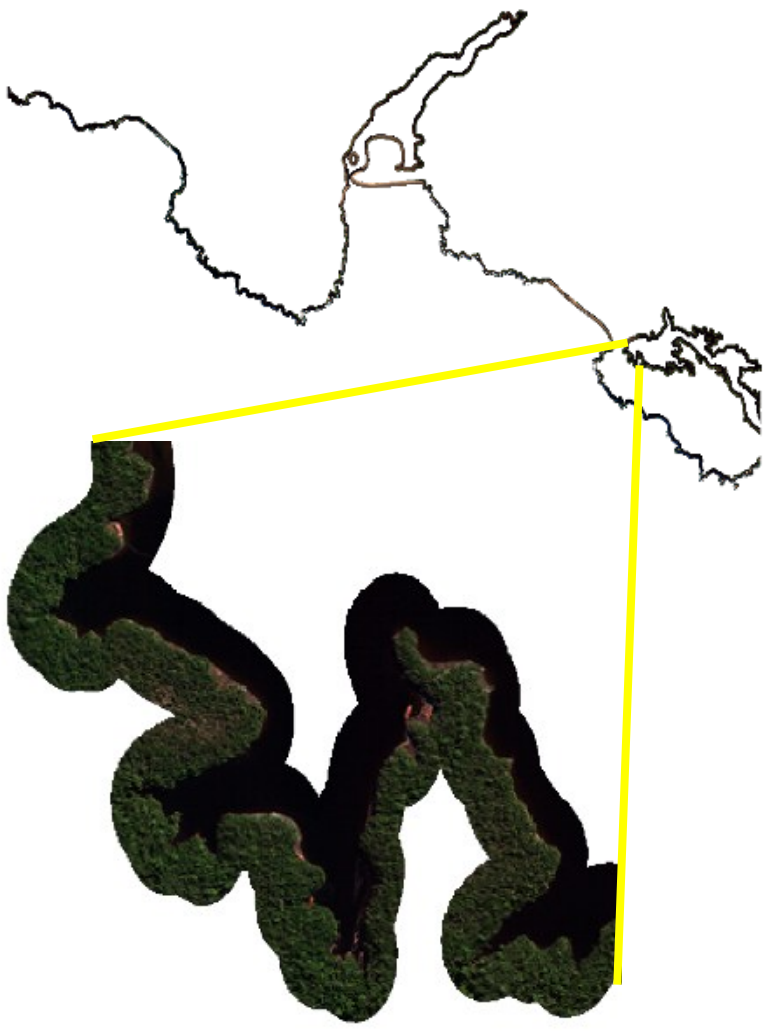

- the clipped extents can be used to perform pansharpening, following the steps in section 'Pansharpening using OTB'

For comparison, the original image when pansharpened using 'Bayes' algorithm, took 4829.19 seconds (1 hour 20 minutes 29 seconds), the new clipped extents took:

Manually digitised shapefile: (includes a multispectral file 457KB and a panchromatic file 893KB) algorithm ran in 1.73 seconds.

Downloaded online shapefile: (includes a multispectral file 747.17MB and a panchromatic file 1.46GB) algorithm ran in 1774.72 seconds (29 minutes 35 seconds).

- when performing pansharpening, the output may not appear as expected, even after altering the band combination. This can be resolved by amending further settings, for example, if viewing the band properties of the newly created pansharpened image, compared to the properties of original multispectral image, the 'Min' and 'Max' values per band may differ

Newly pansharpened image band combination:

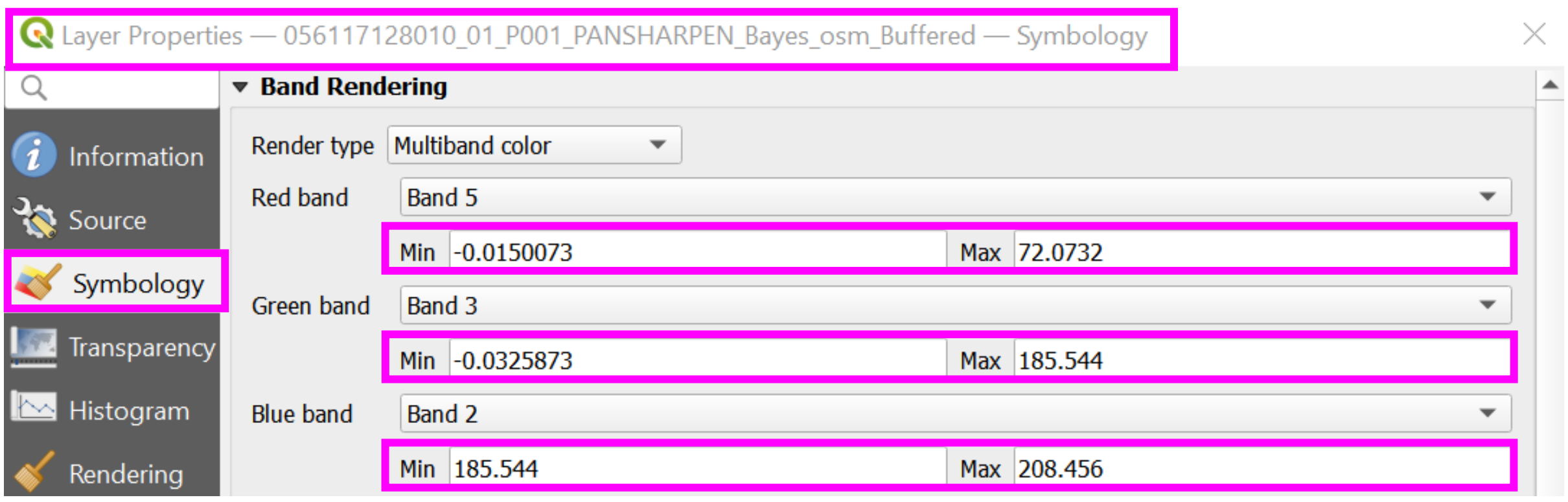

Original multispectral image band combination:

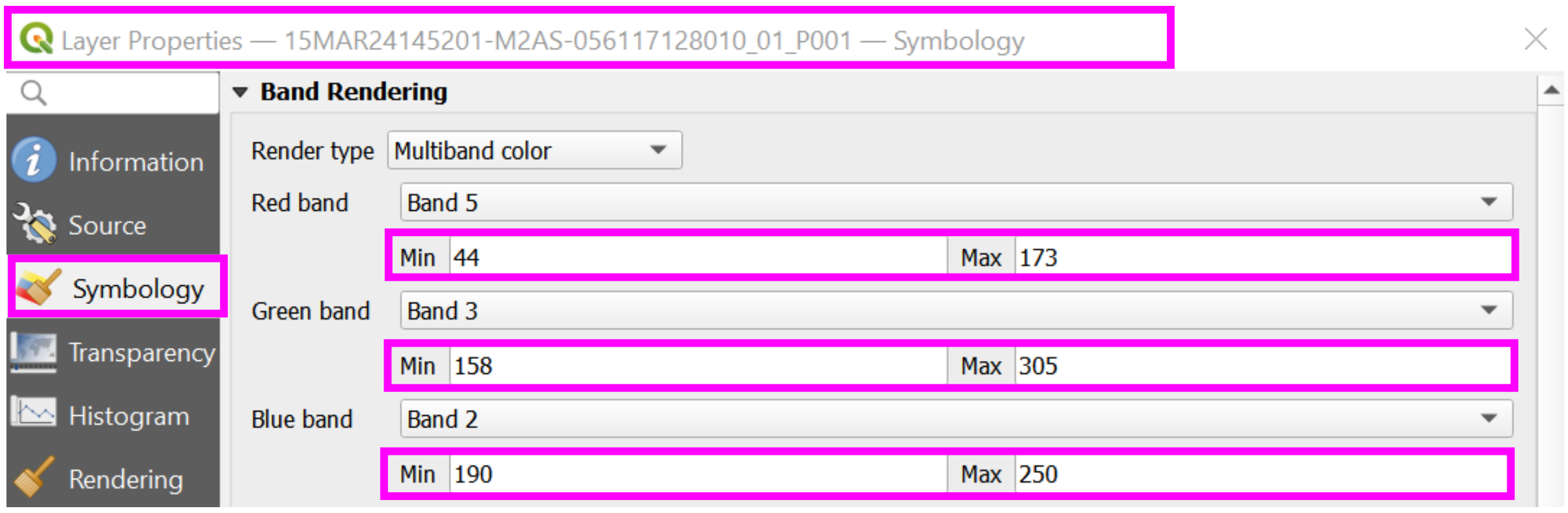

- using the values of the ‘Min’ and ‘Max’ band combinations from the original multispectral image, manually amend those of the newly pansharpened image. Please note, copying and pasting the style directly in the ‘Layers’ pane > ‘Styles’ > ‘Copy Style’ and ‘Styles’ > ‘Paste Style’ will not work as the image formats are different.

SYSTEMATIC SCANNING

GRID SCALE AND SCALE TO SCAN

13. Selecting a grid size for systematic scanning

The following guide details recommended scanning scales for detecting stranded or live cetaceans (or a similar sized target feature). A suitable zoom scale when scanning for medium sized whales like, right whales (*Eubalaena*) and sperm whales (*Physeter macrocephalus*), is 1:1,500. For larger species like fin (*Balaenoptera physalus*) and blue whales (*Balaenoptera musculus*), it may be more efficient to scan at 1:2,000. For smaller whale and dolphin species, it may be more appropriate to scan at 1:1250. These zoom scales are recommended for scanning to ensure features are not missed, however, once a feature is located, users can zoom to finer scales to investigate the feature, such as 1:600. These scanning zoom levels are guides only, and reference checks should be made at the beginning of each project relevant to the subject feature size.

For the aforementioned zoom levels, Table S1.1 details an estimated guide of grid cell height and width for certain computer screen sizes (the measurements were taken in QGIS with all visible layer panels removed, to give a full height and width view of the satellite image).

Table S1.1: Recommended scales to scan for specific computer screen sizes, for large, medium and small-sized stranded cetaceans

Screen Size (inch)	Zoom Scale (ratio scale – m on map : is equal to m in real life)	Feature Size (metre(s) - m)	Cell Height (metre(s) - m)	Cell Width (metre(s) - m)
14	1:1,250	<9	175	420
	1:1,500	9-18	210	510
	1:2,000	18-30	275	675
24	1:1,250	<9	280	640
	1:1,500	9-18	335	765
	1:2,000	18-30	450	1020
27	1:1,250	<9	390	845

	1:1,500	9-18	470	1015
	1:2,000	18-30	630	1350

To reference check these scales for your project or to define your own scale to scan for other target features, follow the below guidance:

a. grid cells need to be a similar size to the size of the main image panel when at a selected base zoom level (minimum zoom level to spot the target feature). In the main image panel of your screen, zoom to an appropriate scale that you would anticipate is needed to see the target feature. For example, 1:1,500 is suitable for a 15 m whale. If unsure of a suitable zoom scale, find another feature present in the image of a similar size to the target feature, or simply measure the screen (anywhere) to the length you would expect of your feature, and use this as a reference whether the current zoom level is suitable for spotting the target feature

b. to measure features, select the ‘Measure Line’ tool on the main toolbar, click on the screen to start measuring and click again to end measuring. The distance measured will appear in the ‘Measure’ window. The zoom scale can be viewed on the bottom of the QGIS window within the ‘Scale’ box

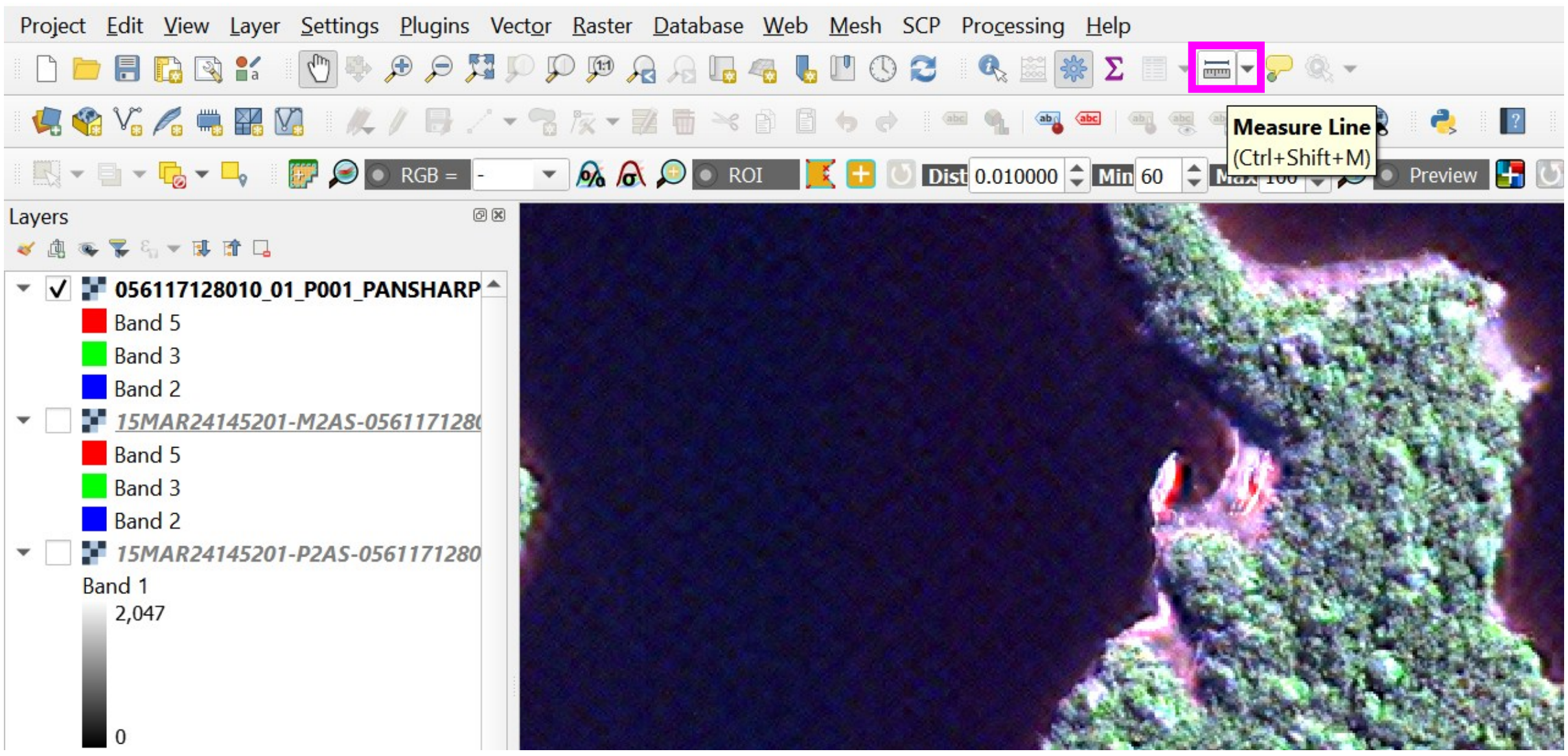

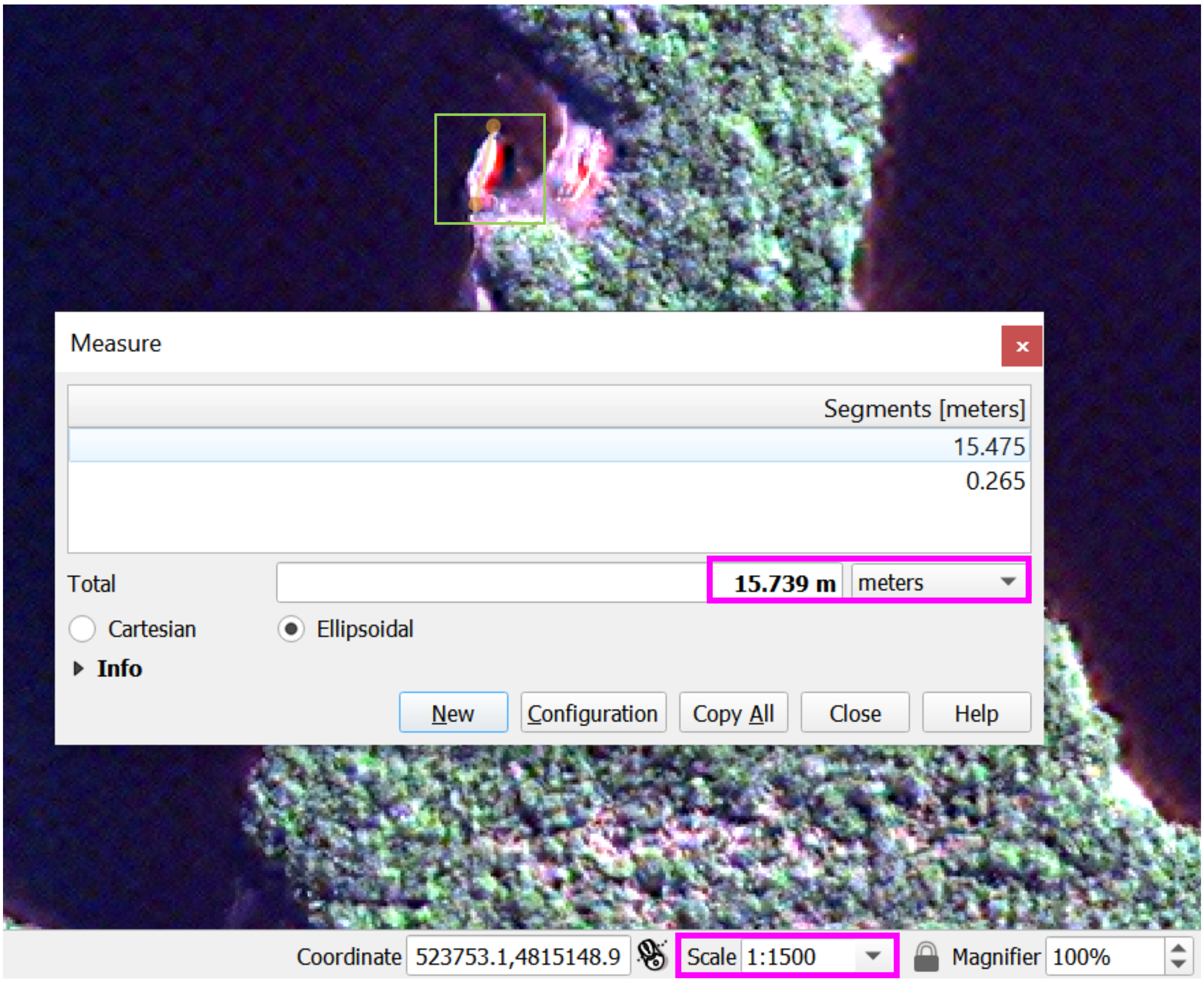

c. once a suitable scale has been found, ensure to record the zoom level ('Scale'), found on the bottom of the main image panel. This will become the base zoom level for reviewing imagery. By scanning at this level, hopefully no features should be missed.

d. at the identified base zoom level, the grid cell size can be informed by measuring both the horizontal and vertical extent of the main image panel

e. switch off all visible panels from the 'View' menu on the main toolbar > 'Panels', and uncheck all the checked panels

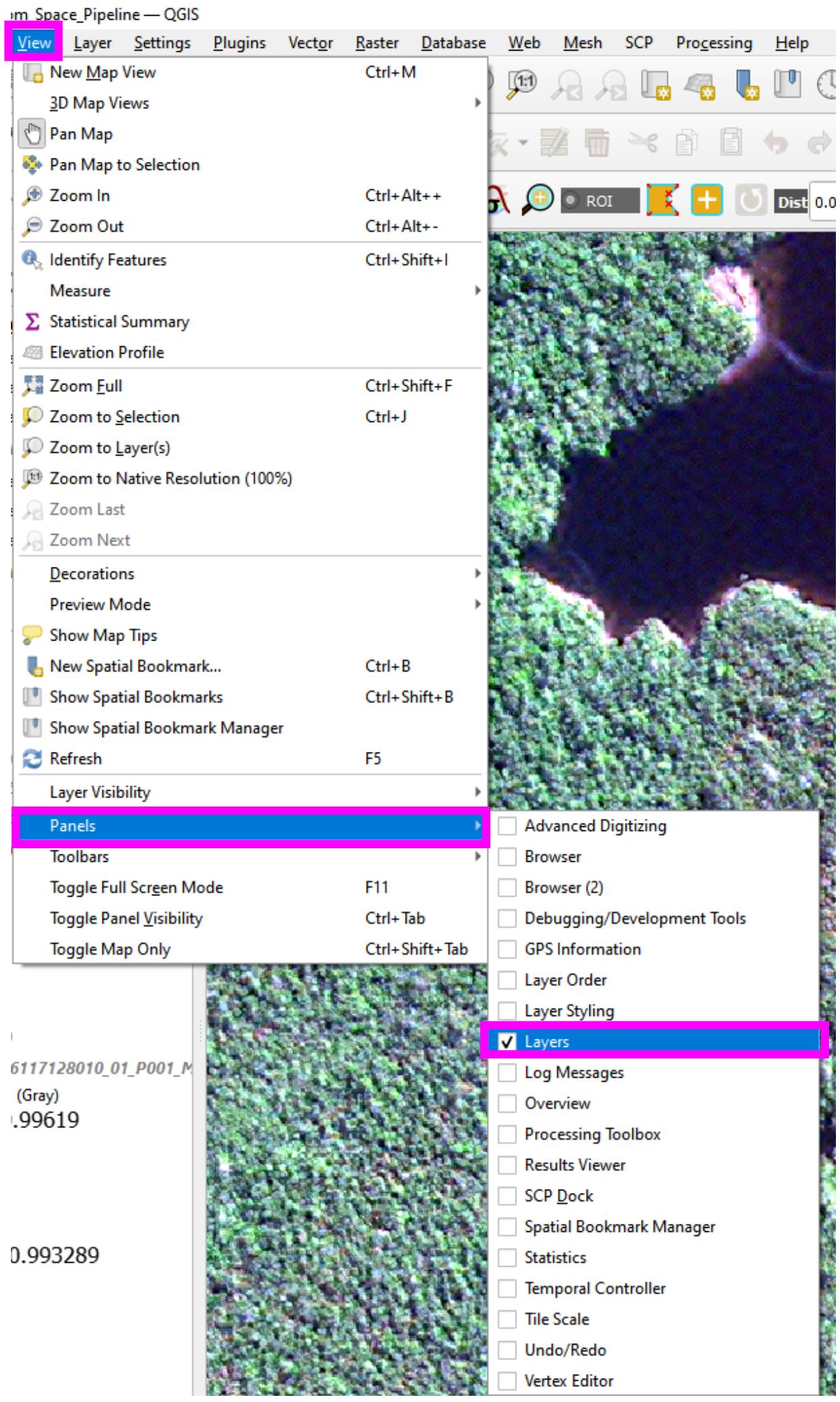

f. using the ‘Measure Line’ tool, measure the screens height and width

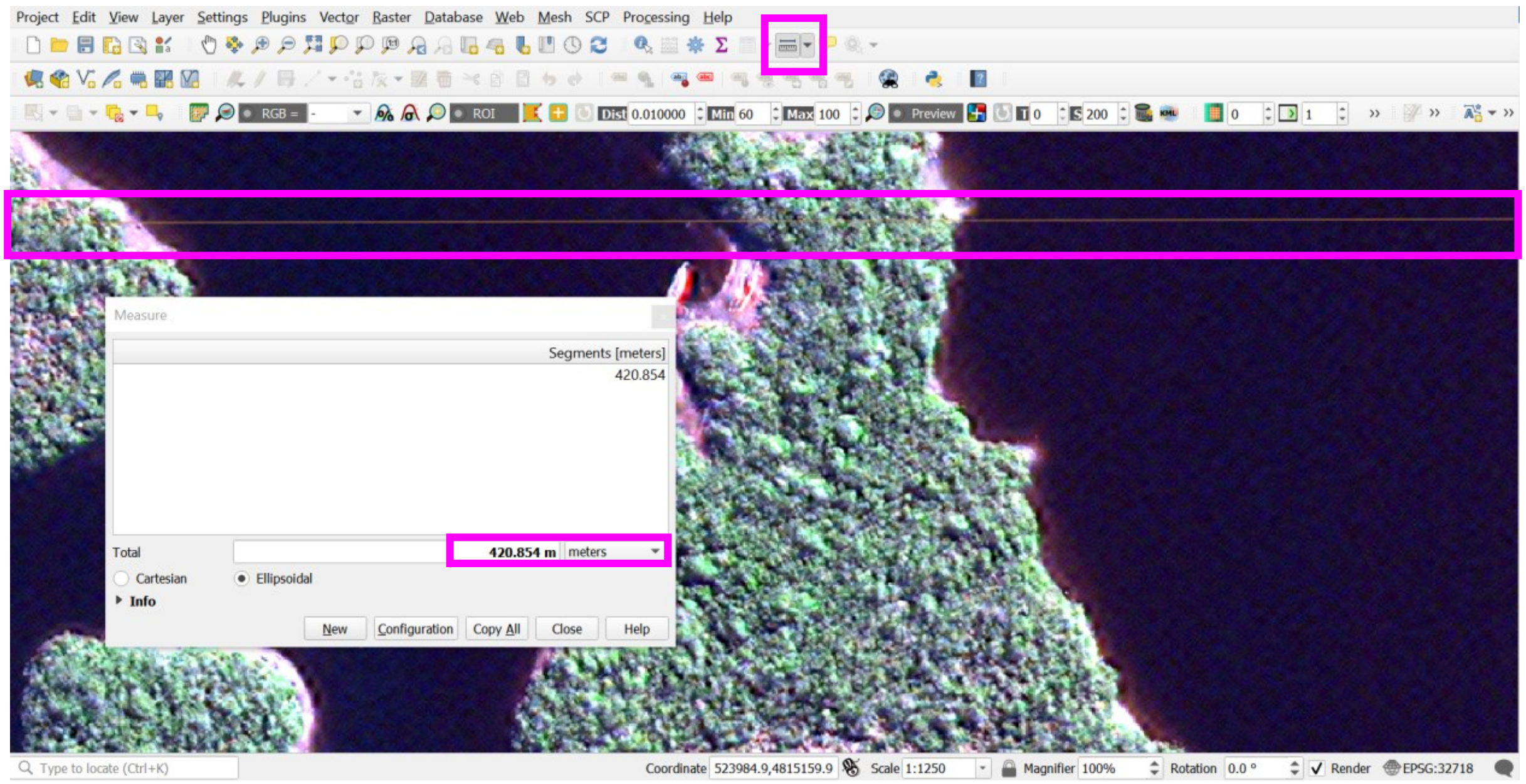

CREATING A GRID

14. Creating a grid for systematic scanning

Use the measured screen height and width or reference measurements from Table S1.1 to create a grid.

a. open the 'Processing Toolbox' > 'Vector Creation' > 'Create Grid'

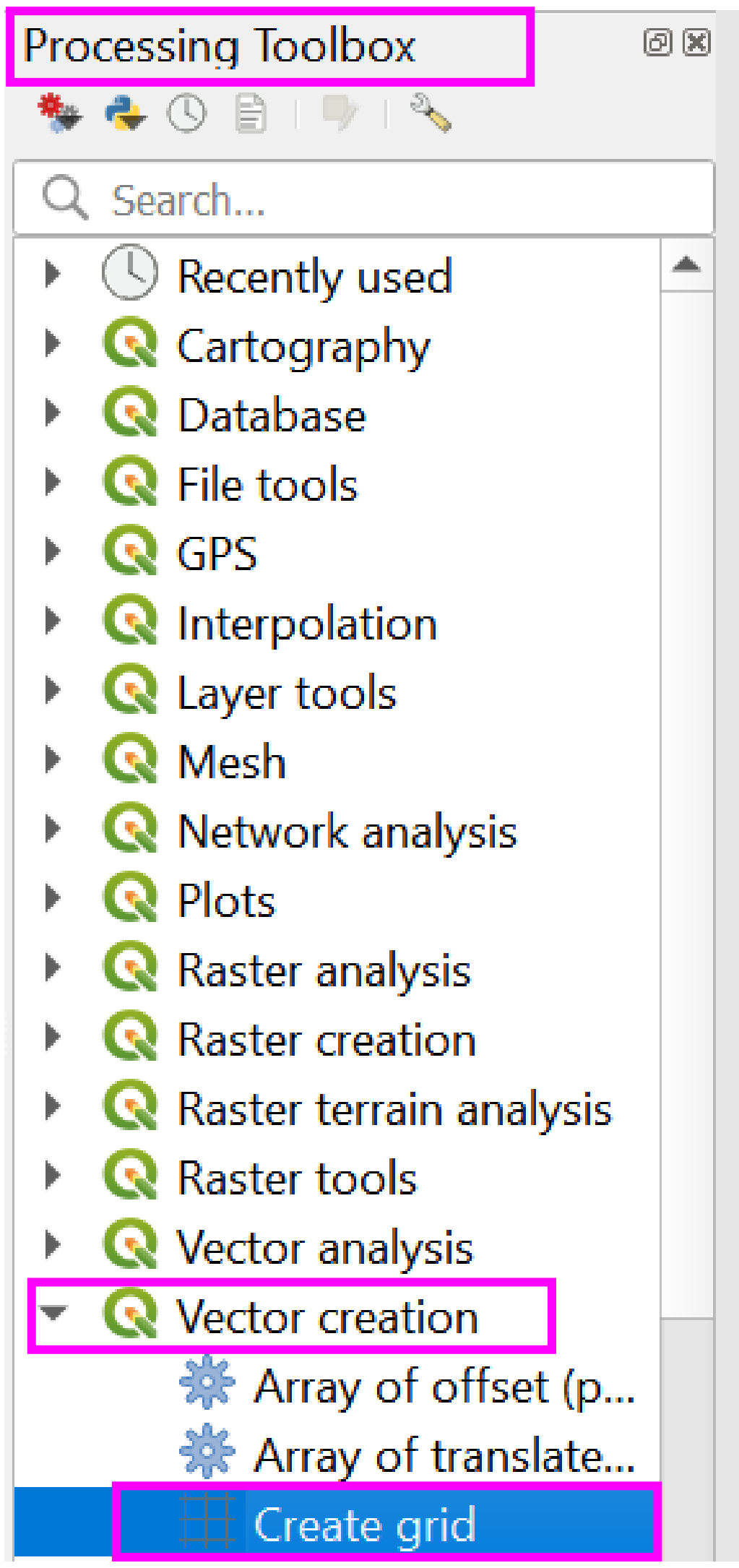

b. select the grid type as 'Rectangle (Polygon)'. To the right of grid extent, click the drop-down arrow and select 'Calculate from Layer'. From the list of layers choose the pansharpened image, and the extent will be mapped as bounding box coordinates of the image.

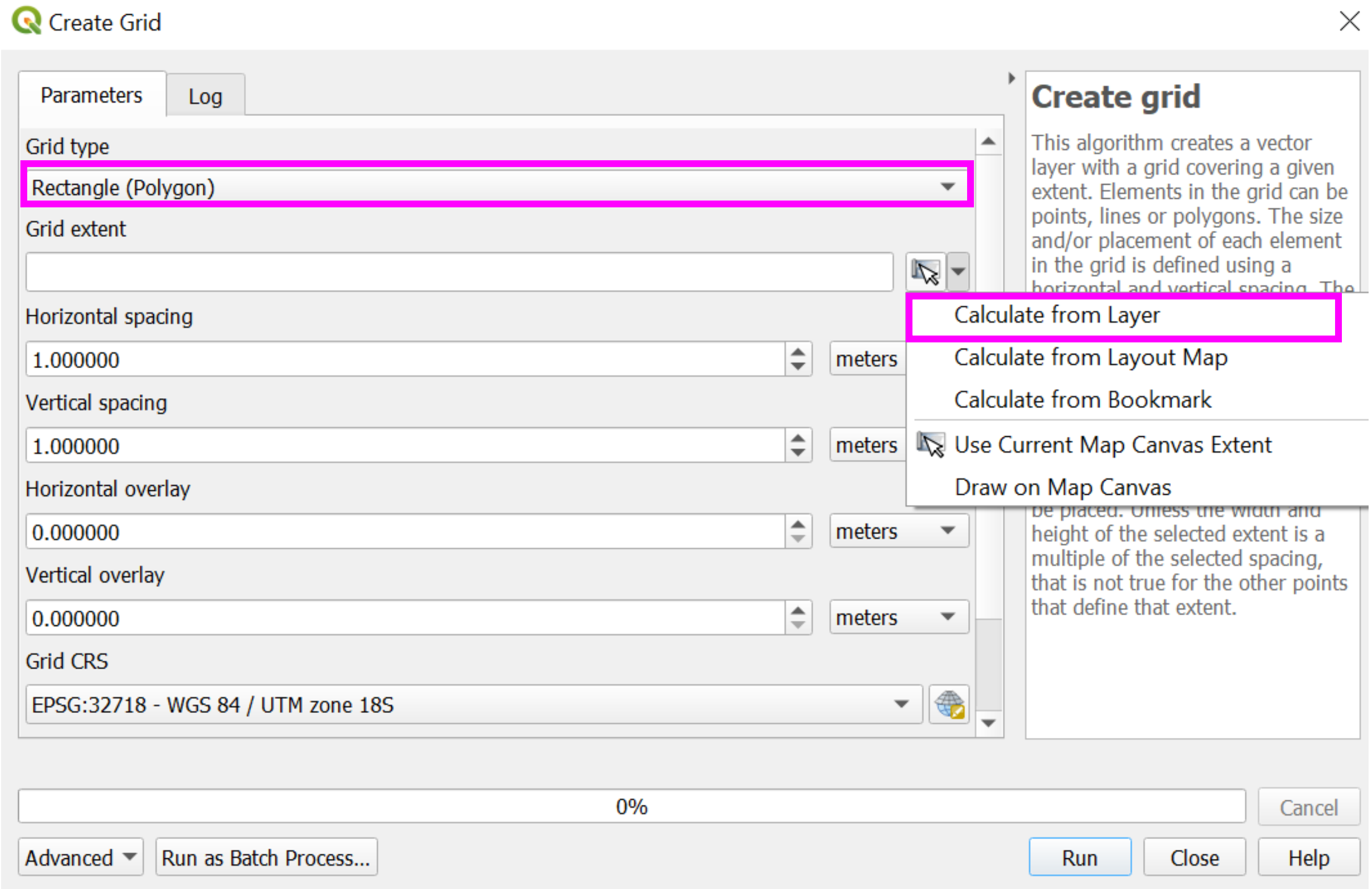

c. in the 'Horizontal Spacing' and 'Vertical Spacing' cells, enter the values of the horizontal and vertical extents measured or taken from the reference table (Table S1.1). The units of measure, for example 'metres', can be changed in the drop-down. Check the projection matches the image and dataframe in the 'Grid CRS', in the 'Grid' cell

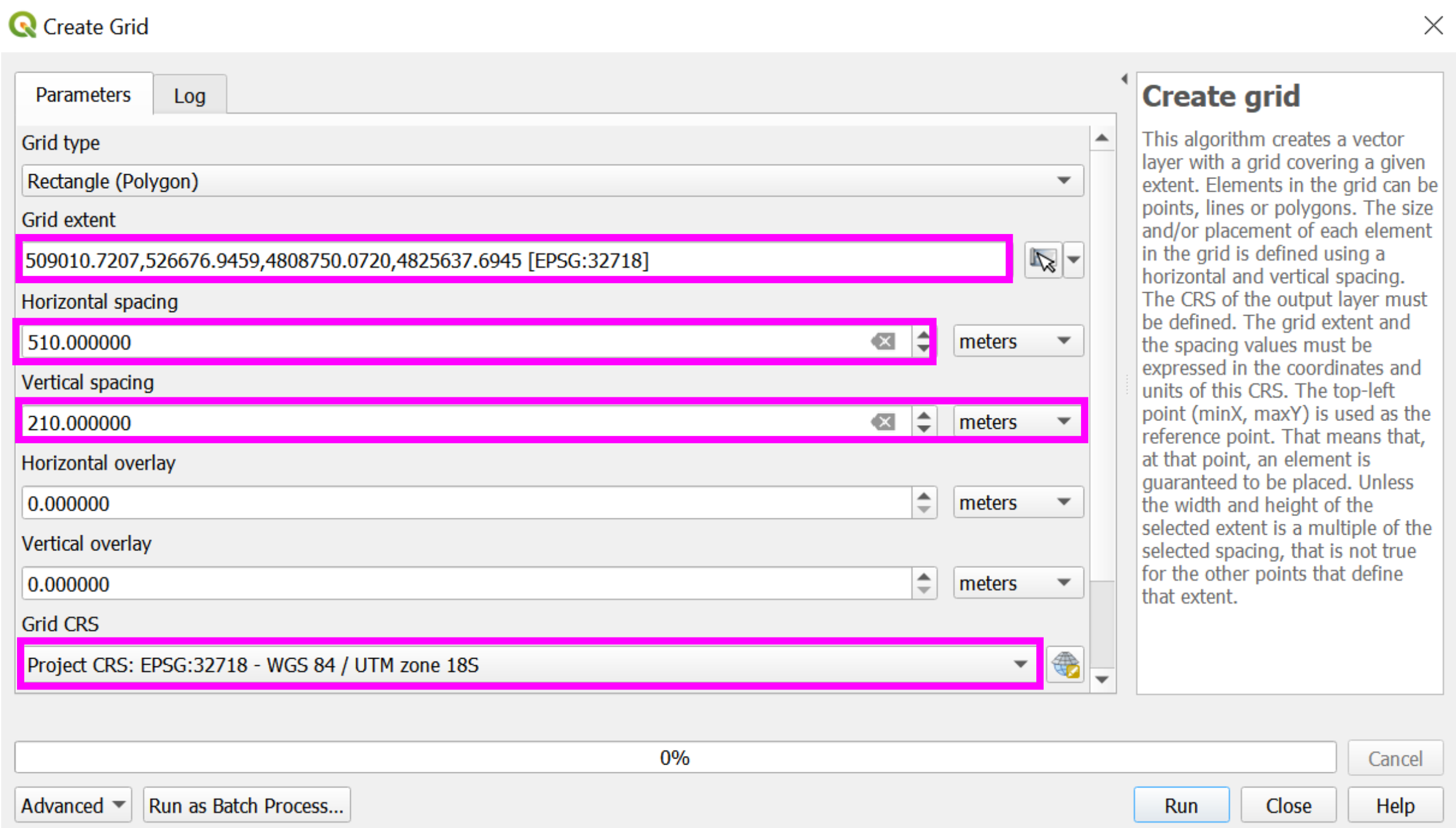

d. select a location and file name to save, and 'Run'

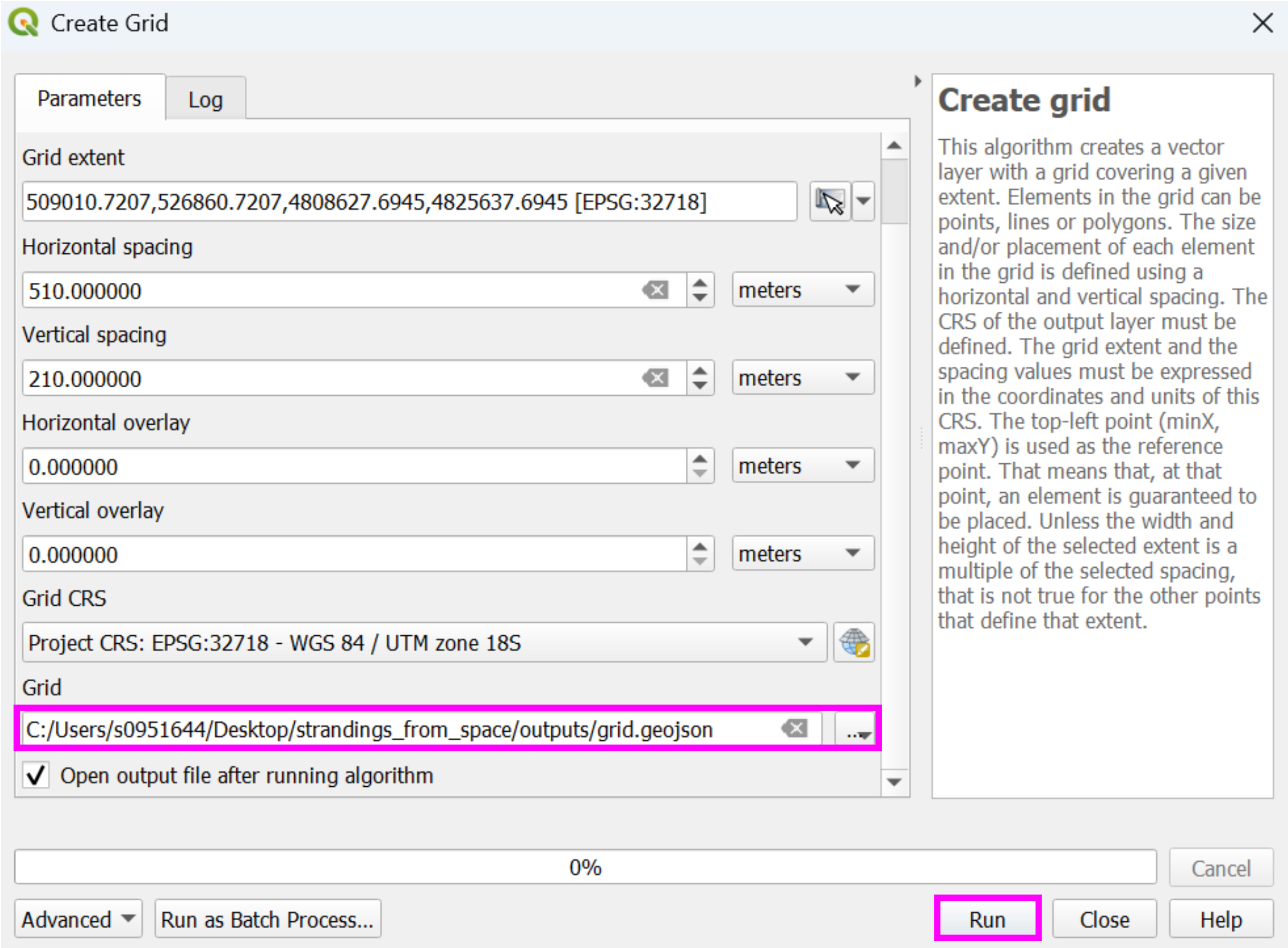

e. the grid will appear in the 'Layers' pane on the left of the screen and a grid will be deposited over the image

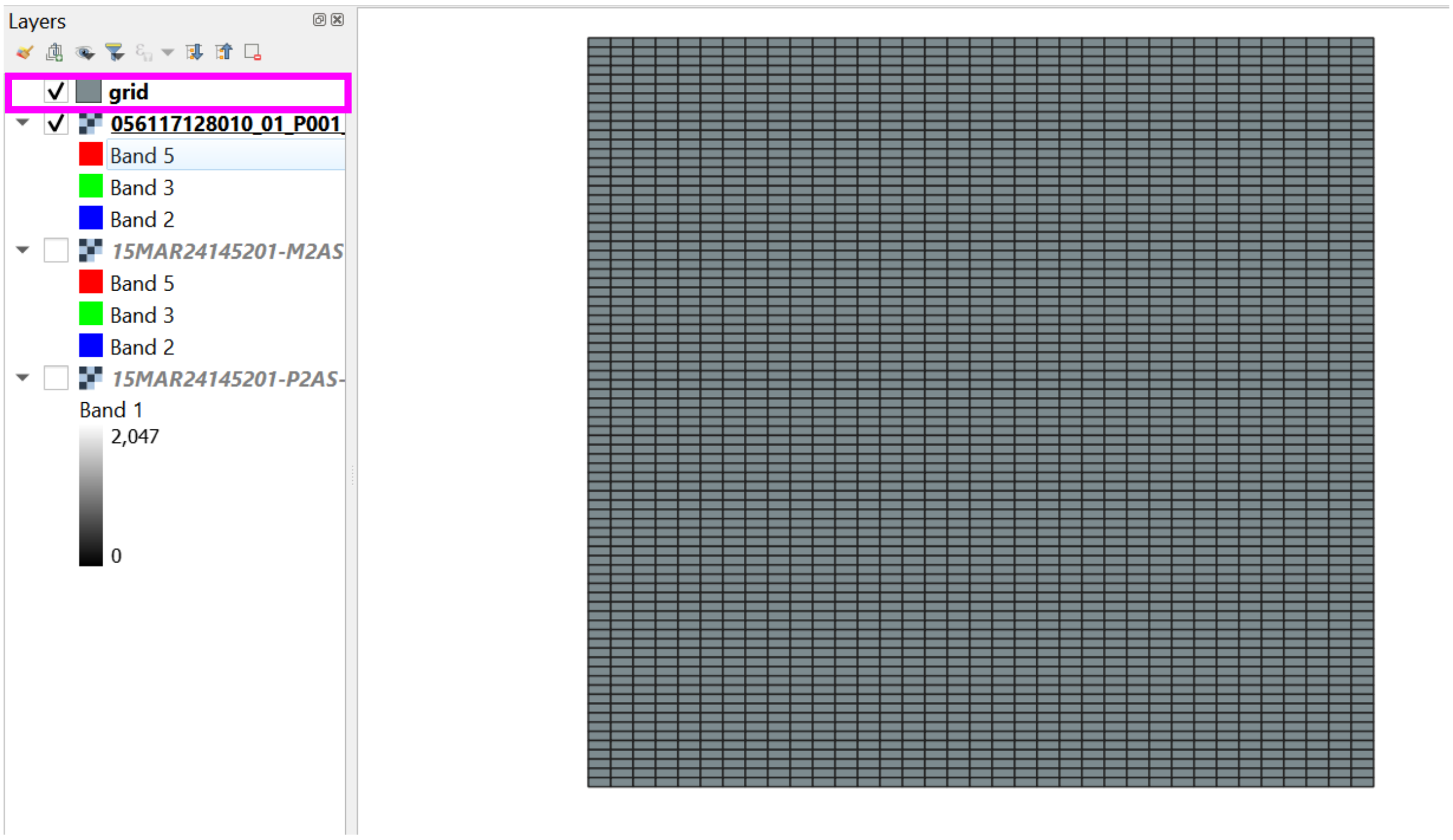

f. the output is typically solid fill, to alter the grid to line fill only, right click the layer in the 'Layers' pane > select 'Properties' > 'Symbology', select the example 'outline green' and amend the line colour accordingly

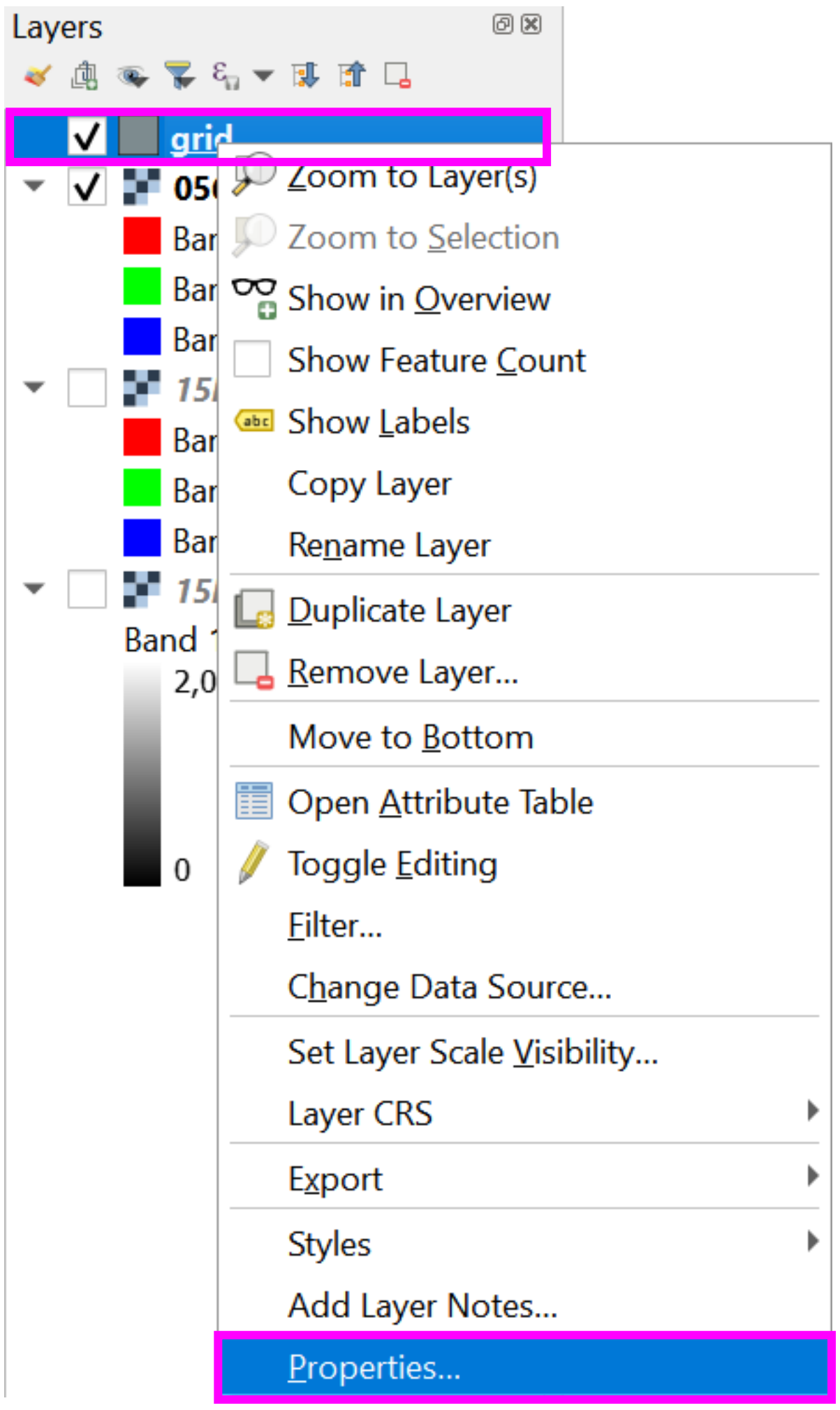

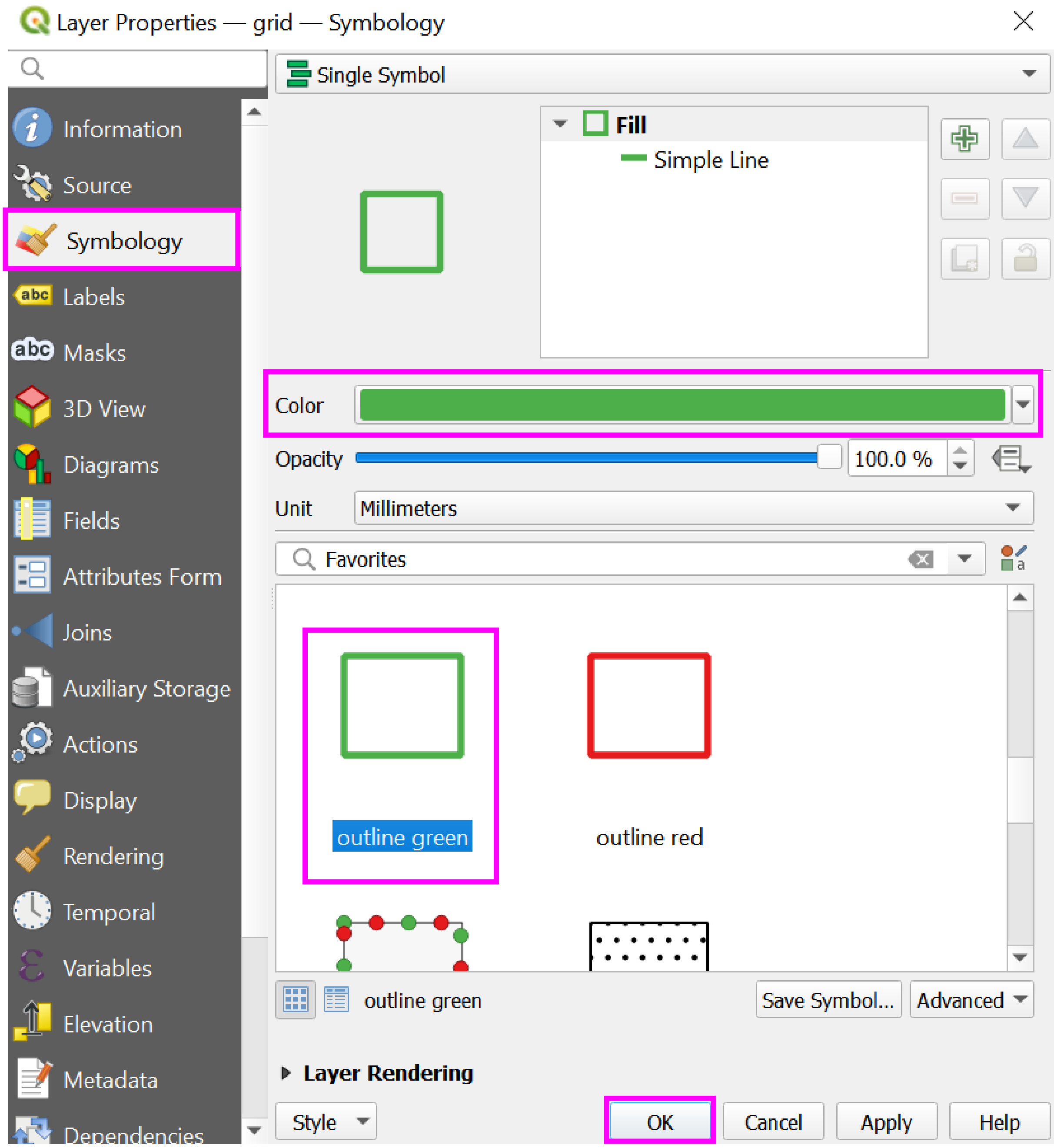

g. the grid will appear as line fill, which is better for visualising and searching imagery. When zooming to the base zoom scale, for example, 1:1,500 (in the example screenshot here, 1:1590, to demonstrate and visualise the grid line edge), each grid cell is approximately the height and width of the main image pane, enabling consistent scanning at the desired zoom

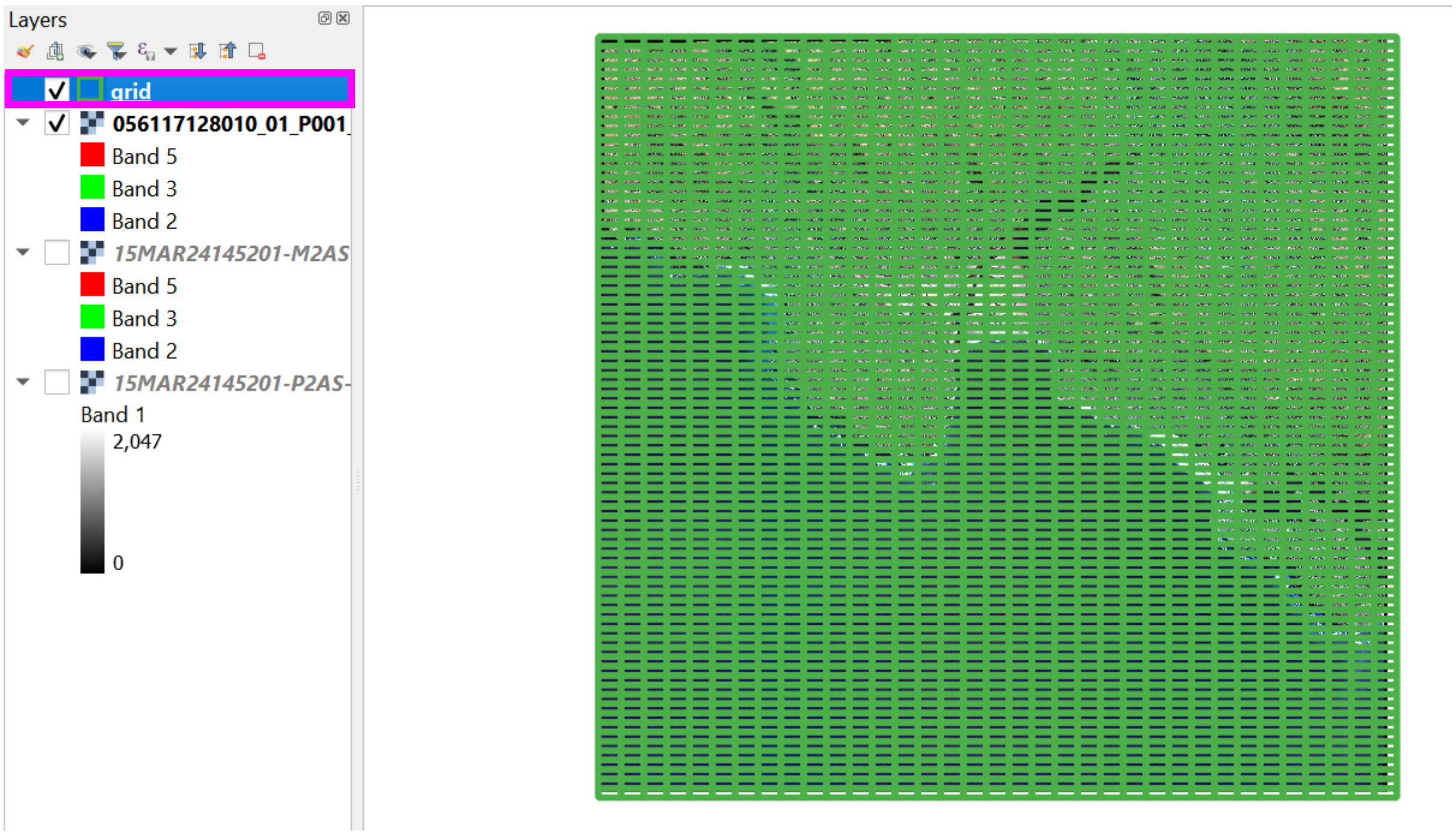

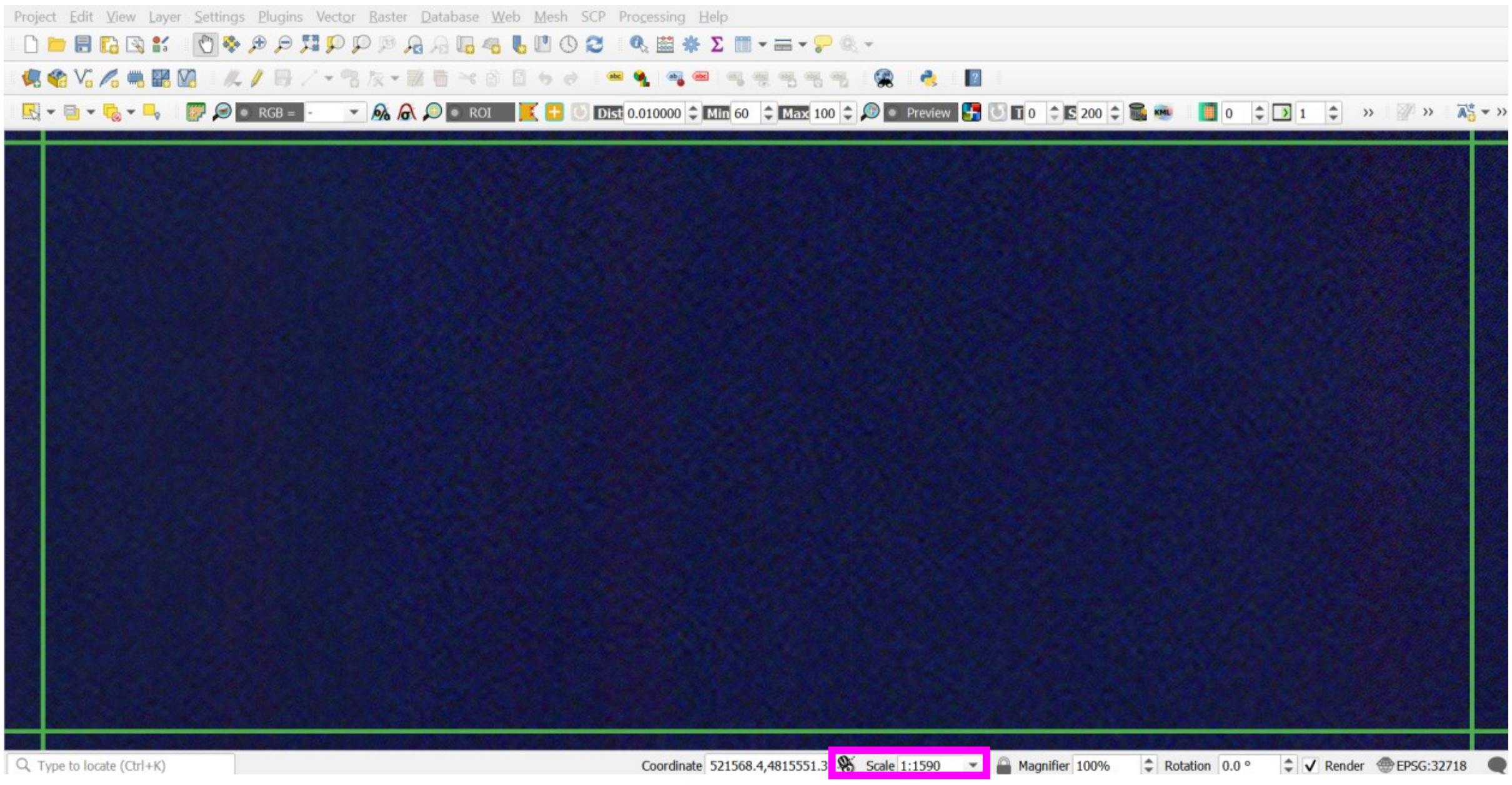

SYSTEMATICALLY SCAN IMAGERY

15. Methods to systematically scan an image

There are two methods to know where to return if pausing or stopping scanning, either ‘add yes markers/coloured cells’ or ‘add id labels’. The method selected is preference of the observer.

a. method one: add yes markers/coloured cells
   i. in the ‘Layers’ pane right click the ‘grid’ layer > ‘Open Attribute Table’

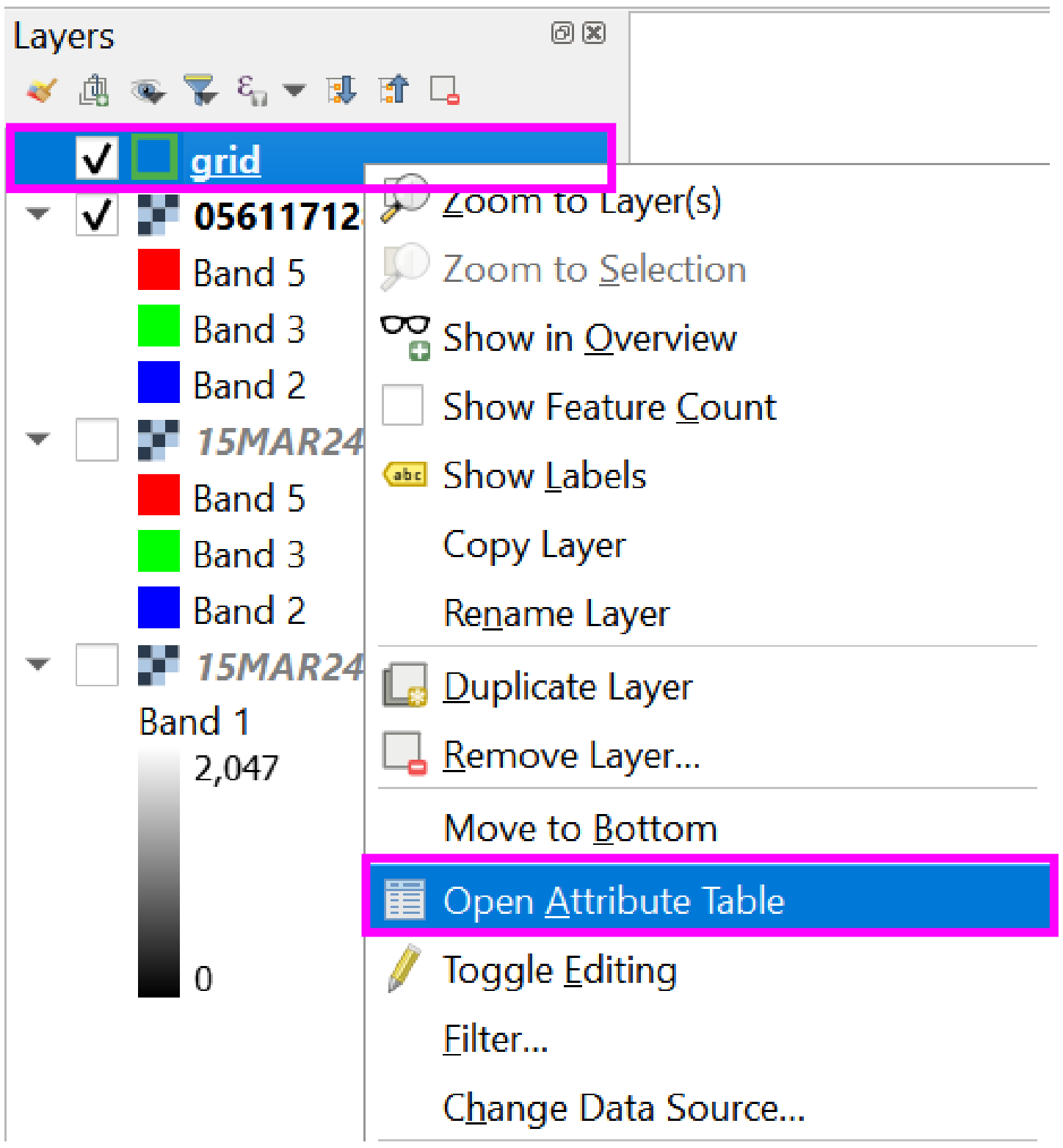

ii. create a new column to store details of the checked cells. First select 'Toggle editing mode' (yellow pencil icon), followed by the 'New field' icon (table with yellow pencil icon)

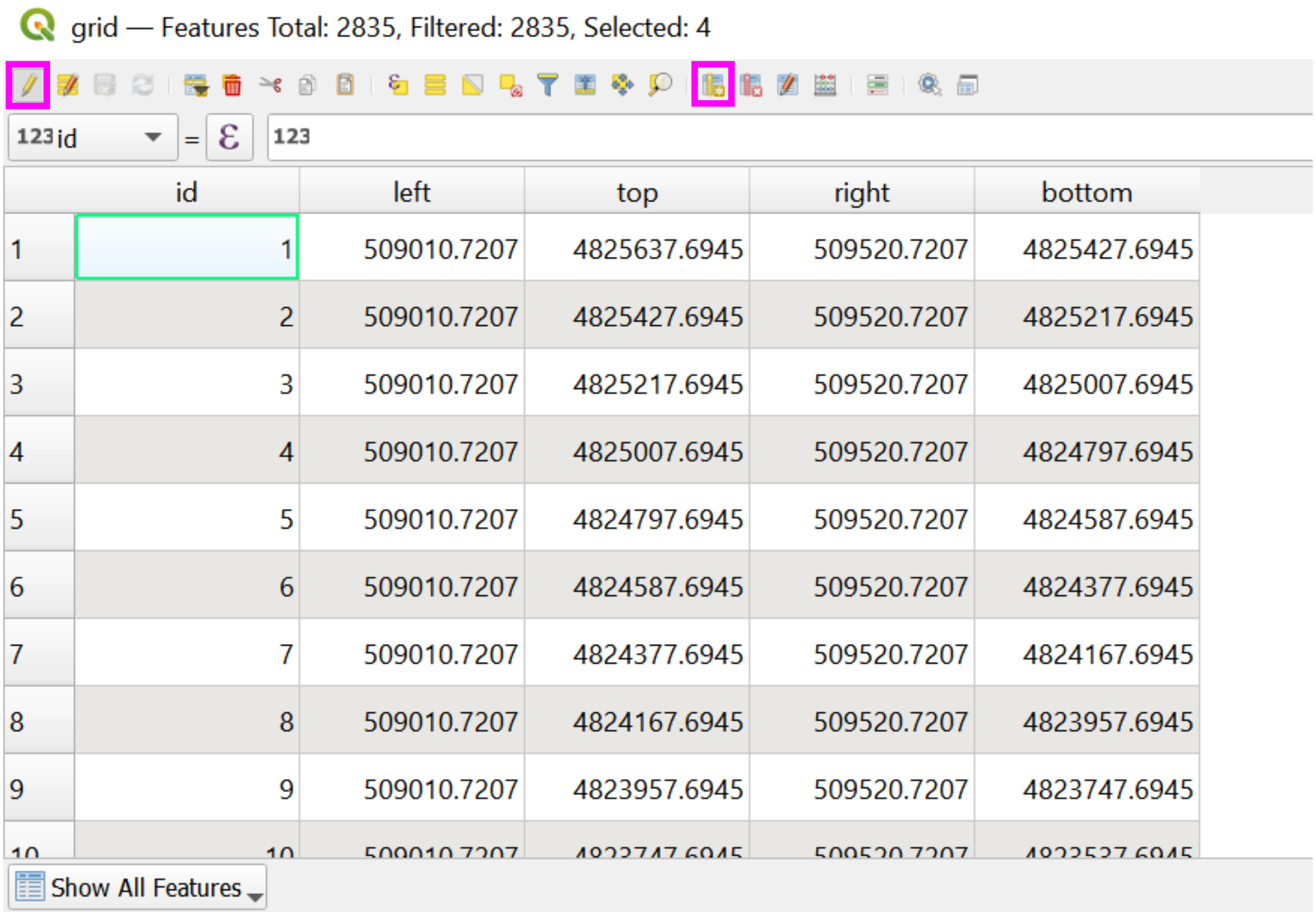

	id	left	top	right	bottom
1	1	509010.7207	4825637.6945	509520.7207	4825427.6945
2	2	509010.7207	4825427.6945	509520.7207	4825217.6945
3	3	509010.7207	4825217.6945	509520.7207	4825007.6945
4	4	509010.7207	4825007.6945	509520.7207	4824797.6945
5	5	509010.7207	4824797.6945	509520.7207	4824587.6945
6	6	509010.7207	4824587.6945	509520.7207	4824377.6945
7	7	509010.7207	4824377.6945	509520.7207	4824167.6945
8	8	509010.7207	4824167.6945	509520.7207	4823957.6945
9	9	509010.7207	4823957.6945	509520.7207	4823747.6945
10	10	509010.7207	4823747.6945	509520.7207	4823537.6945

iii. in the ‘Add Field’ window, create a new column called ‘complete’, select the ‘Type’ as ‘Text (string)’, and ‘OK’ (name columns using lower case only and where spaces are required, use underscores ‘for_example’, for compatibility in coding interfaces like Python)

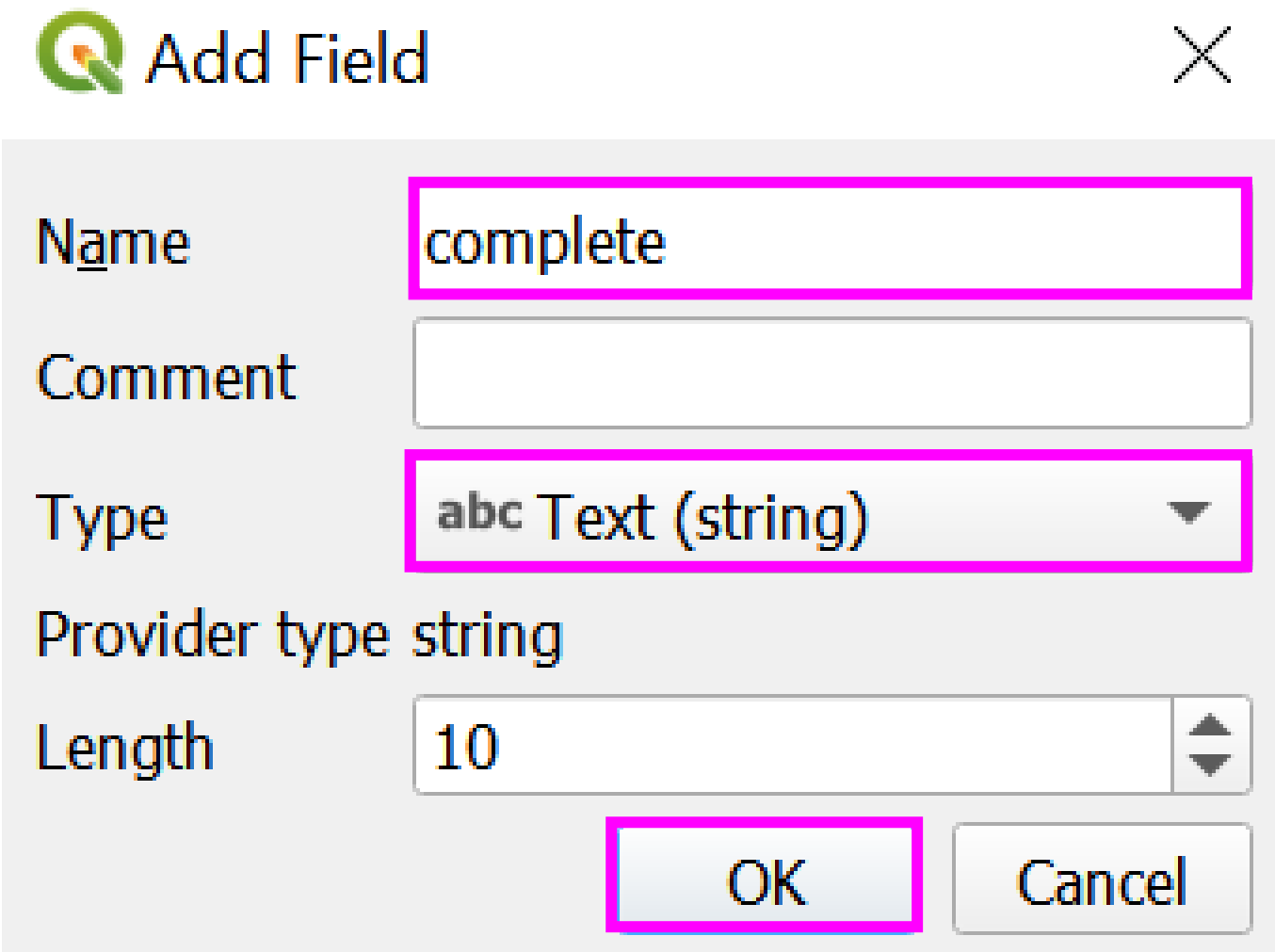

iv. the ‘complete’ column will appear in the attribute table

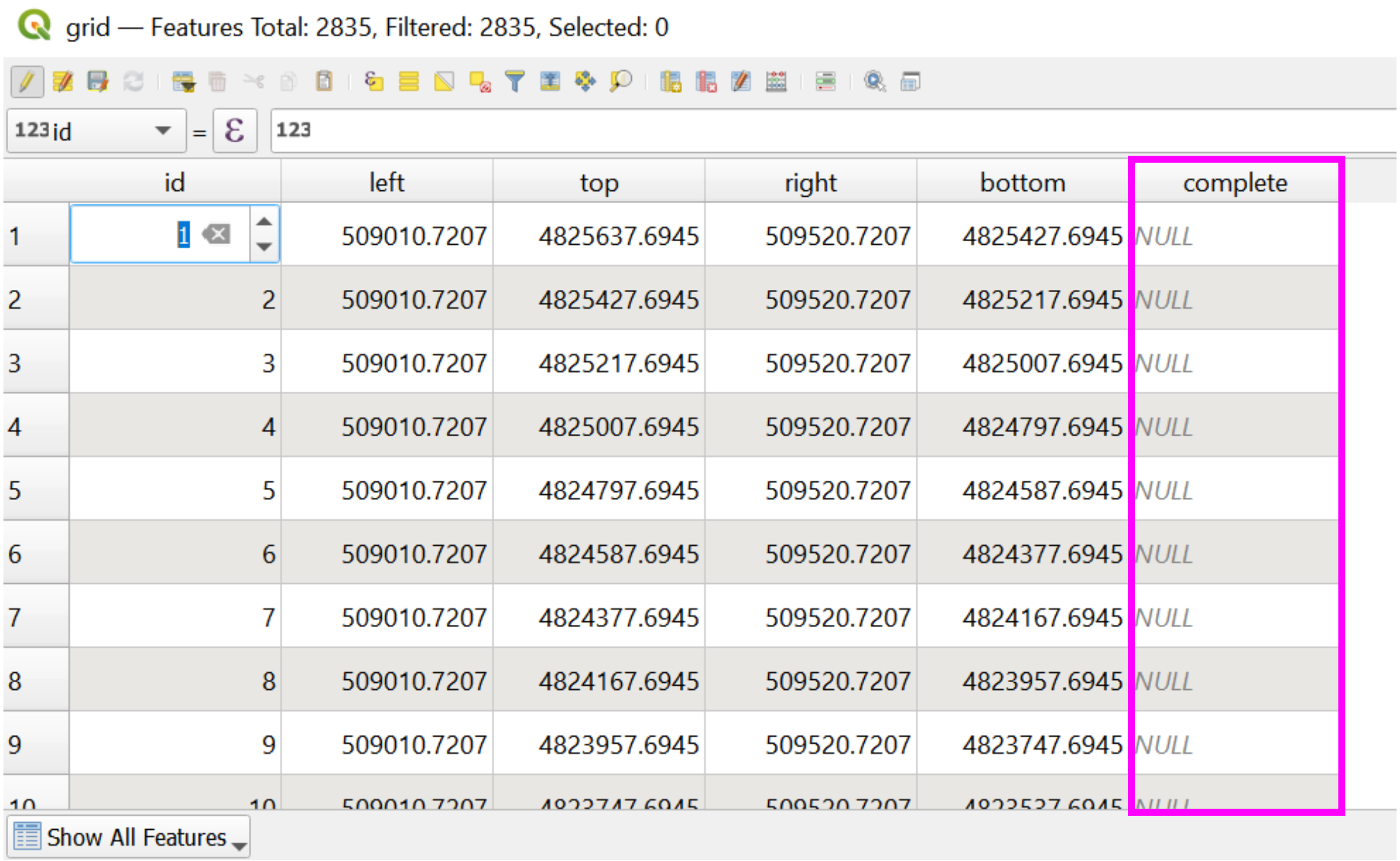
grid — Features Total: 2835, Filtered: 2835, Selected: 0

	id	left	top	right	bottom	complete
1	1	509010.7207	4825637.6945	509520.7207	4825427.6945	NULL
2	2	509010.7207	4825427.6945	509520.7207	4825217.6945	NULL
3	3	509010.7207	4825217.6945	509520.7207	4825007.6945	NULL
4	4	509010.7207	4825007.6945	509520.7207	4824797.6945	NULL
5	5	509010.7207	4824797.6945	509520.7207	4824587.6945	NULL
6	6	509010.7207	4824587.6945	509520.7207	4824377.6945	NULL
7	7	509010.7207	4824377.6945	509520.7207	4824167.6945	NULL
8	8	509010.7207	4824167.6945	509520.7207	4823957.6945	NULL
9	9	509010.7207	4823957.6945	509520.7207	4823747.6945	NULL

Show All Features

v. the next step is to mark the completed cells. Using the ‘Select Features by Area or Single Click’ icon on the main toolbar (yellow square with arrow), click and drag on first searched grid cell and release the click once all searched cells are highlighted (yellow highlight below)

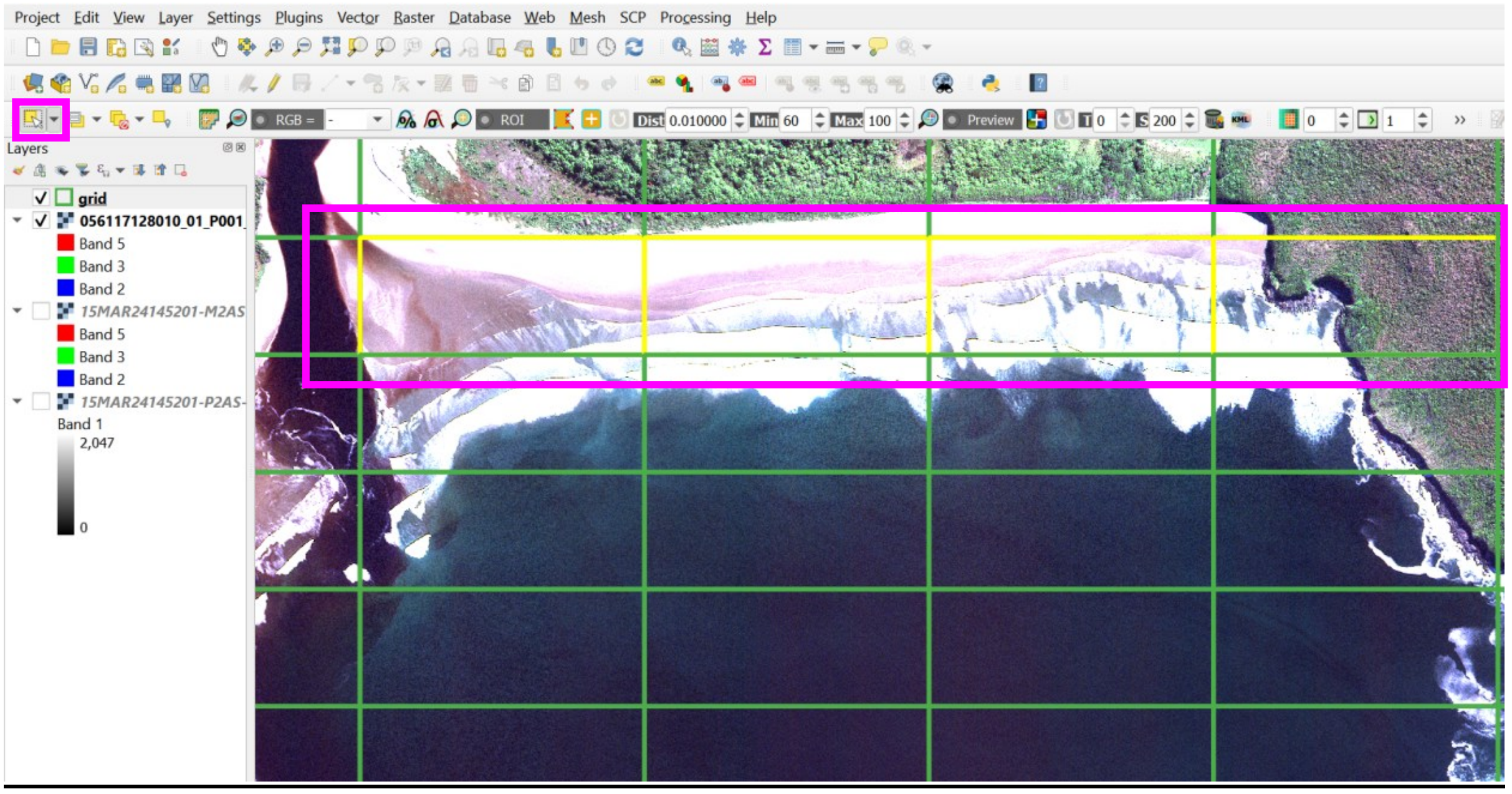

vi. re-open the attribute table from the 'Layers' pane, in the bottom left of the open attribute table, amend the 'Show All Features' drop-down to 'Show Selected Features'

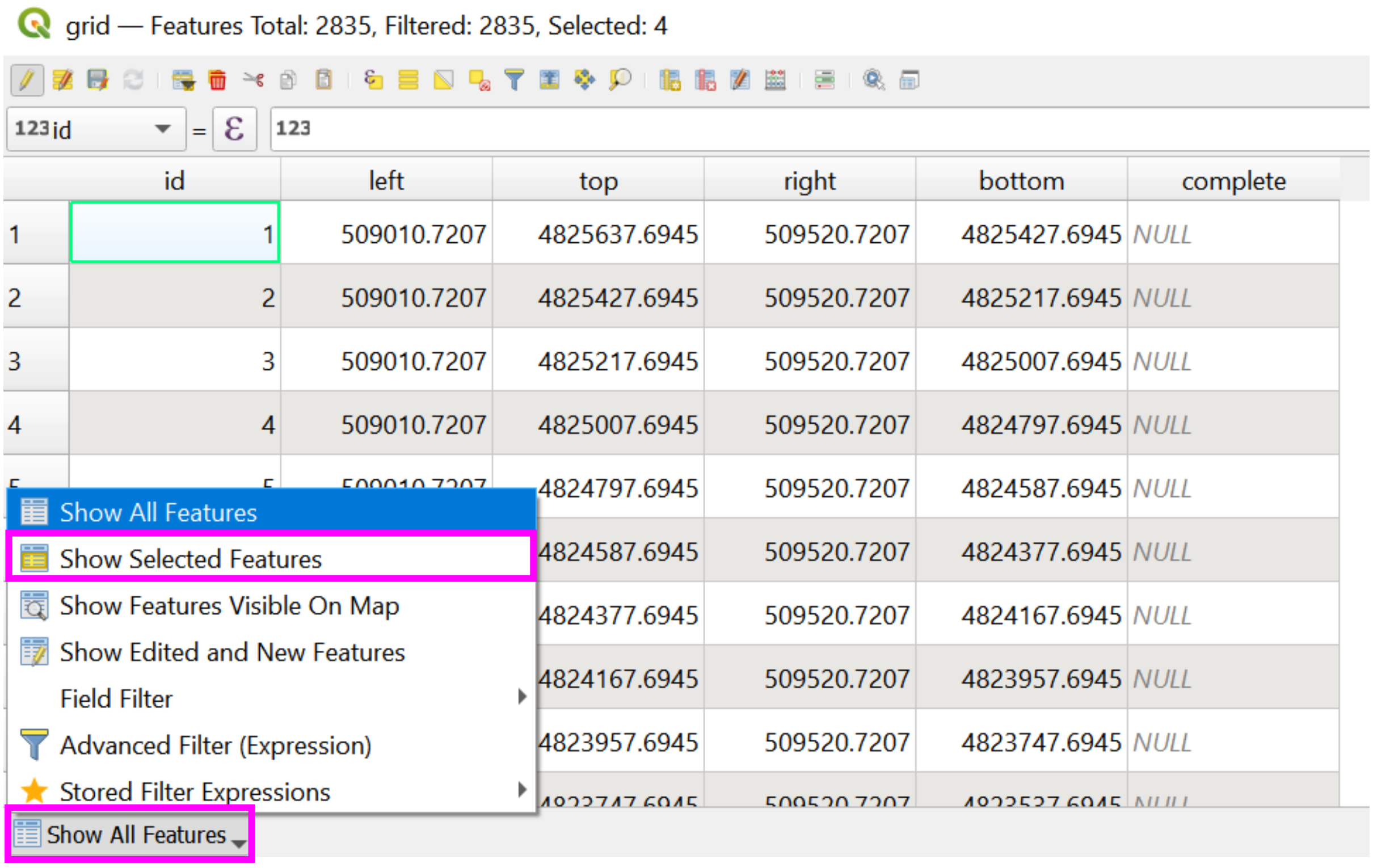

vii. only the cells selected using the 'Select Features by Area or Single Click' will now be visible in the attribute table

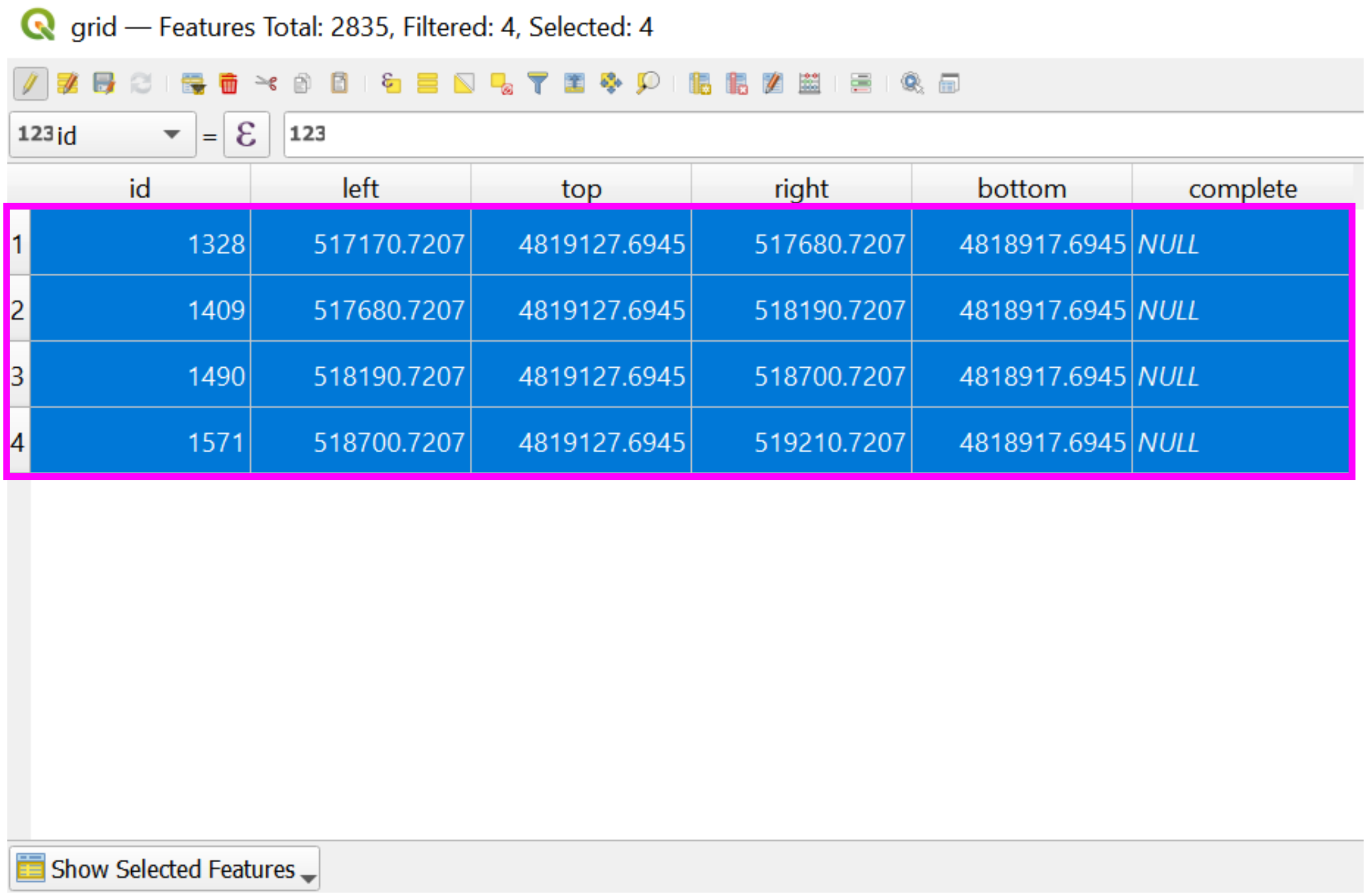

viii. Using the expression toolbar at the top of the attribute table mark the complete columns with 'yes'. Click the black drop arrow, and select the 'complete' column. In the 'Ɛ' entry, add the value to populate the 'complete' column, for example, 'yes' (ensure words are put in inverted commas for QGIS to know this is a string format (text type)). Select 'Update Filtered' and yes will appear in all selected cells of the 'complete' column

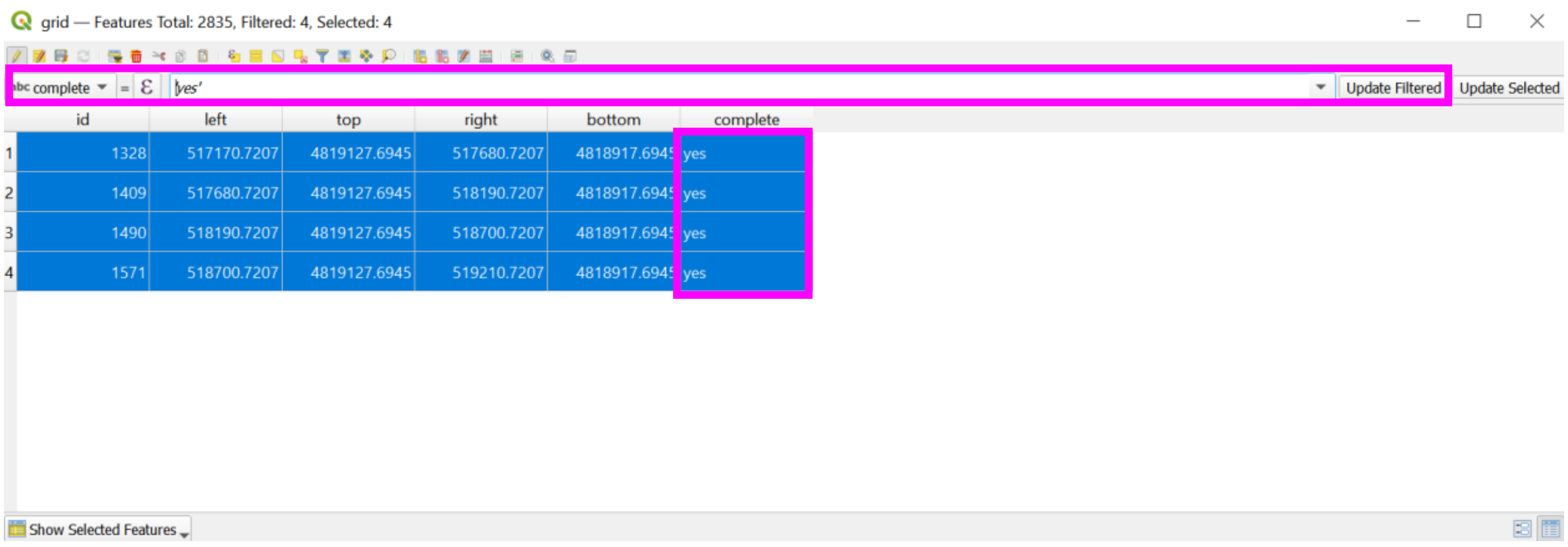

ix. as a reference, 'Labels' can be turned on to display 'yes' in all the complete cells, to appear visible when scanning. Right click the 'grid' layer in the 'Layers' pane > 'Properties' > 'Labels'. In the first drop-down, select 'Single Labels', set the 'Value' as 'complete' and amend the 'Color' to match the grid colour, and 'Apply then 'OK'

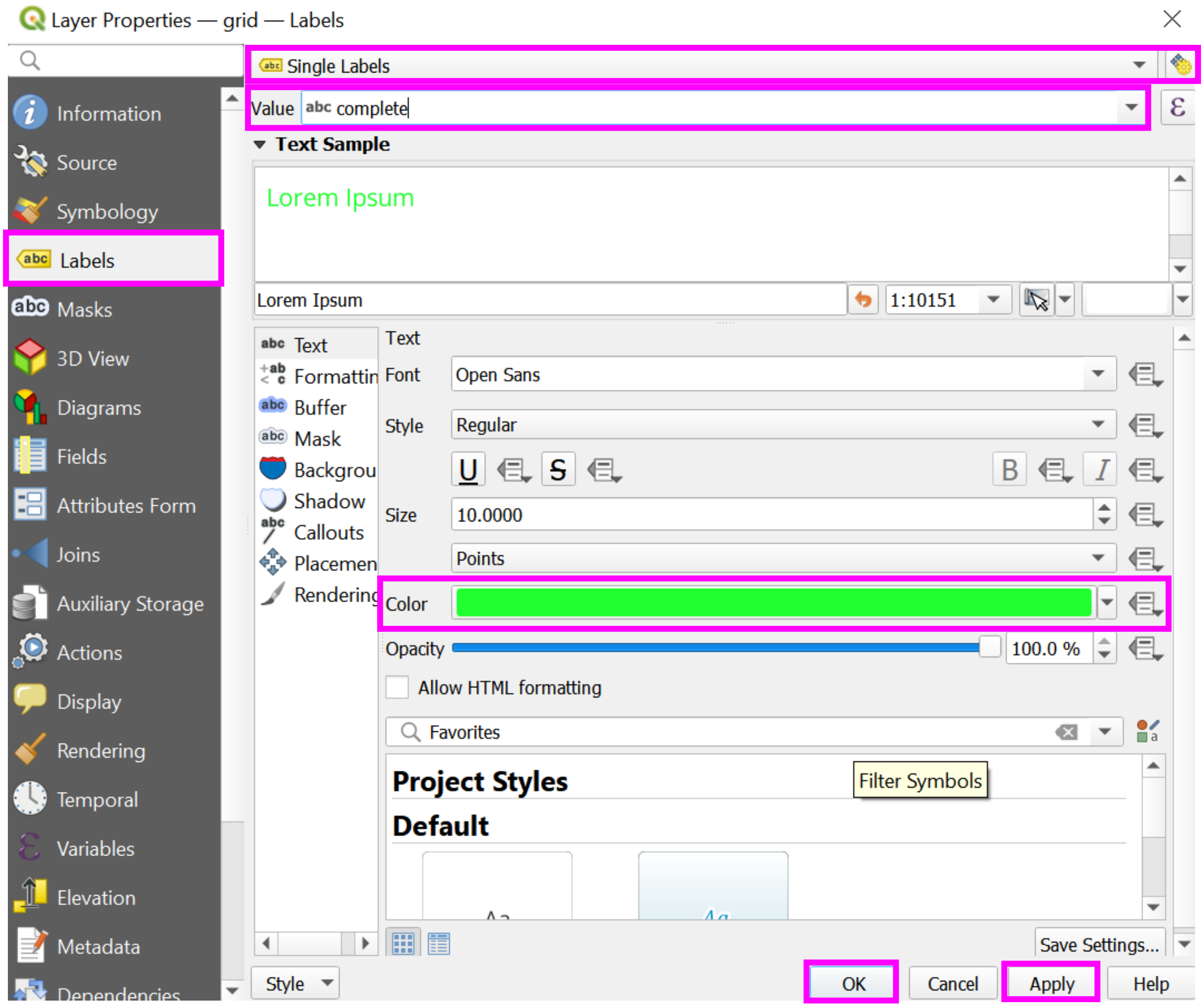

x. ‘yes’ will now populate in the ‘complete’ cells in the main image window

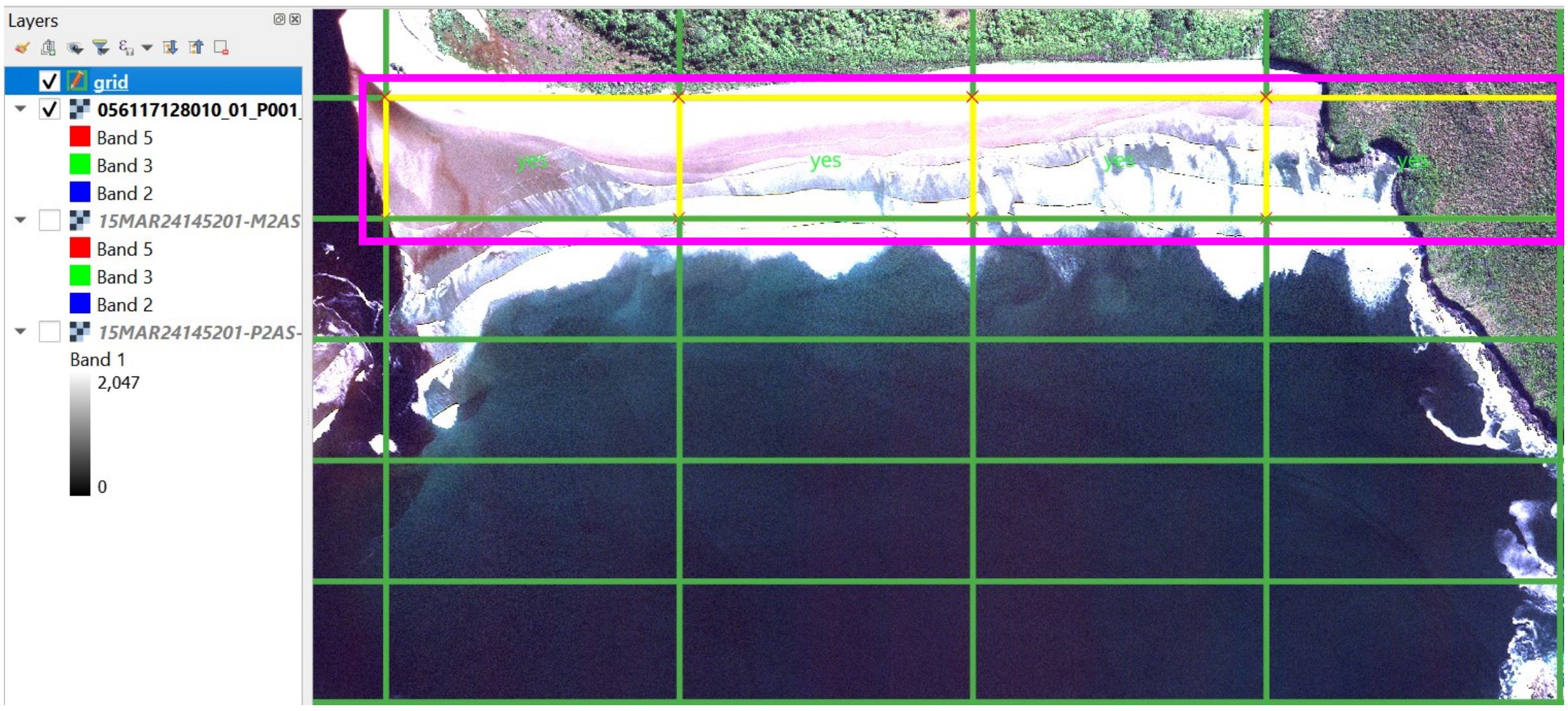

xi. alternative to labelling cells with ‘yes’, is to solid fill complete cells. Right click the ‘grid’ layer in the ‘Layers’ pane > ‘Properties’ > ‘Symbology’. Set the ‘Value’ as ‘complete’, select ‘Classify’, to define the ‘yes’ and ‘all other values’ classes.

Highlight each class in turn and amend the ‘Symbol’ colour. Assign ‘yes’ a solid fill and ‘all other values’ line fill. Click ‘ Apply’ and ‘OK’

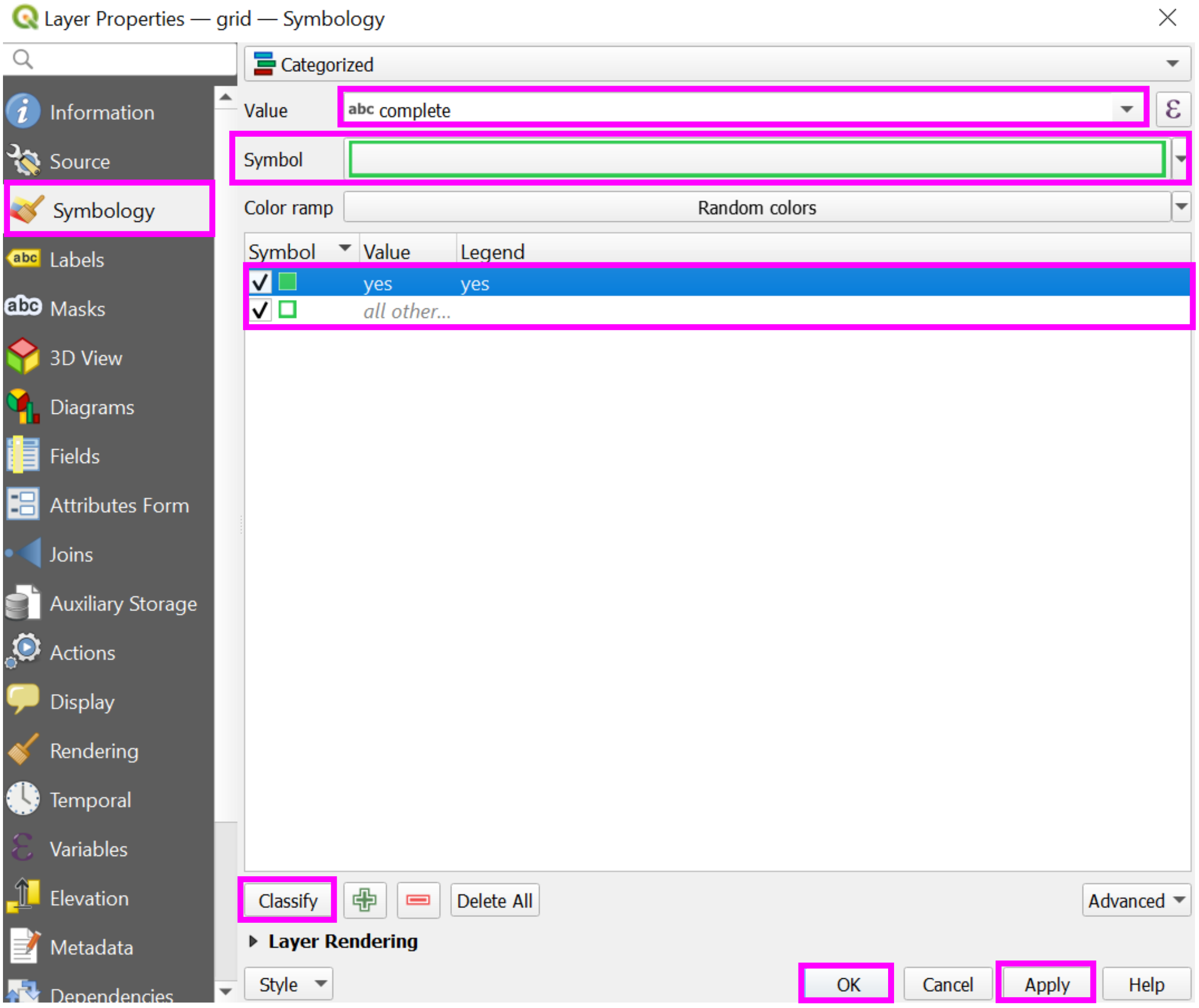

xii. ‘complete’ cells will now appear solid fill in the main image window

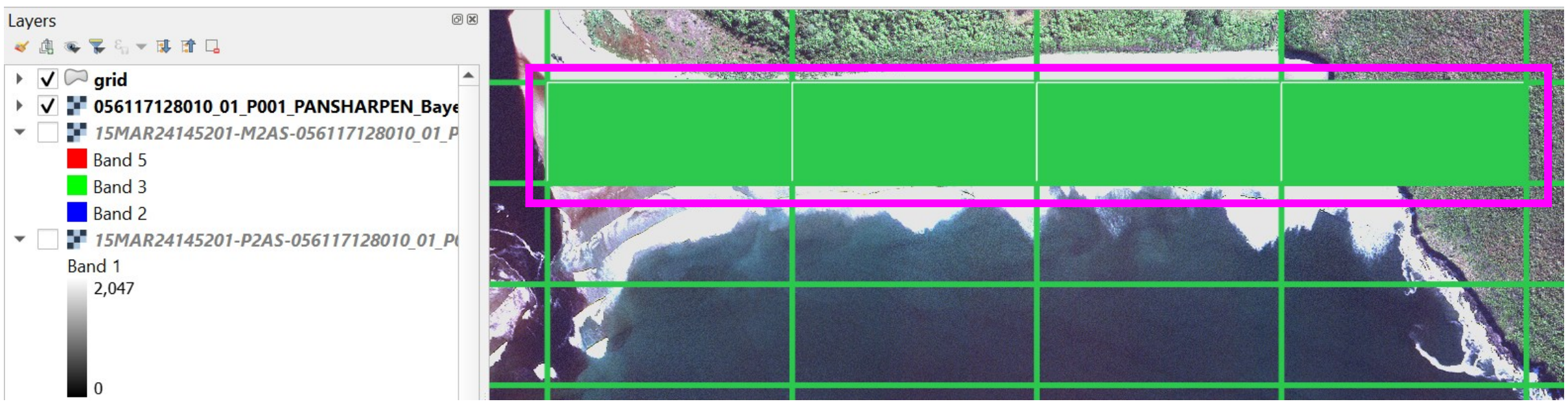

xiii. Each time scanning is paused or stopped, populate the complete column for the searched cells in the attribute table and the ‘yes’ labels or solid colour, and this will automatically update in the main grid

b. Method two: add id labels

i. right click the ‘grid’ layer in the ‘Layers’ pane > select ‘Properties’ > ‘Labels’. In the first drop-down select ‘Single Labels’, set the ‘Value’ to ‘id’ and amend the ‘Color’ to match the grid colour, select ‘OK’

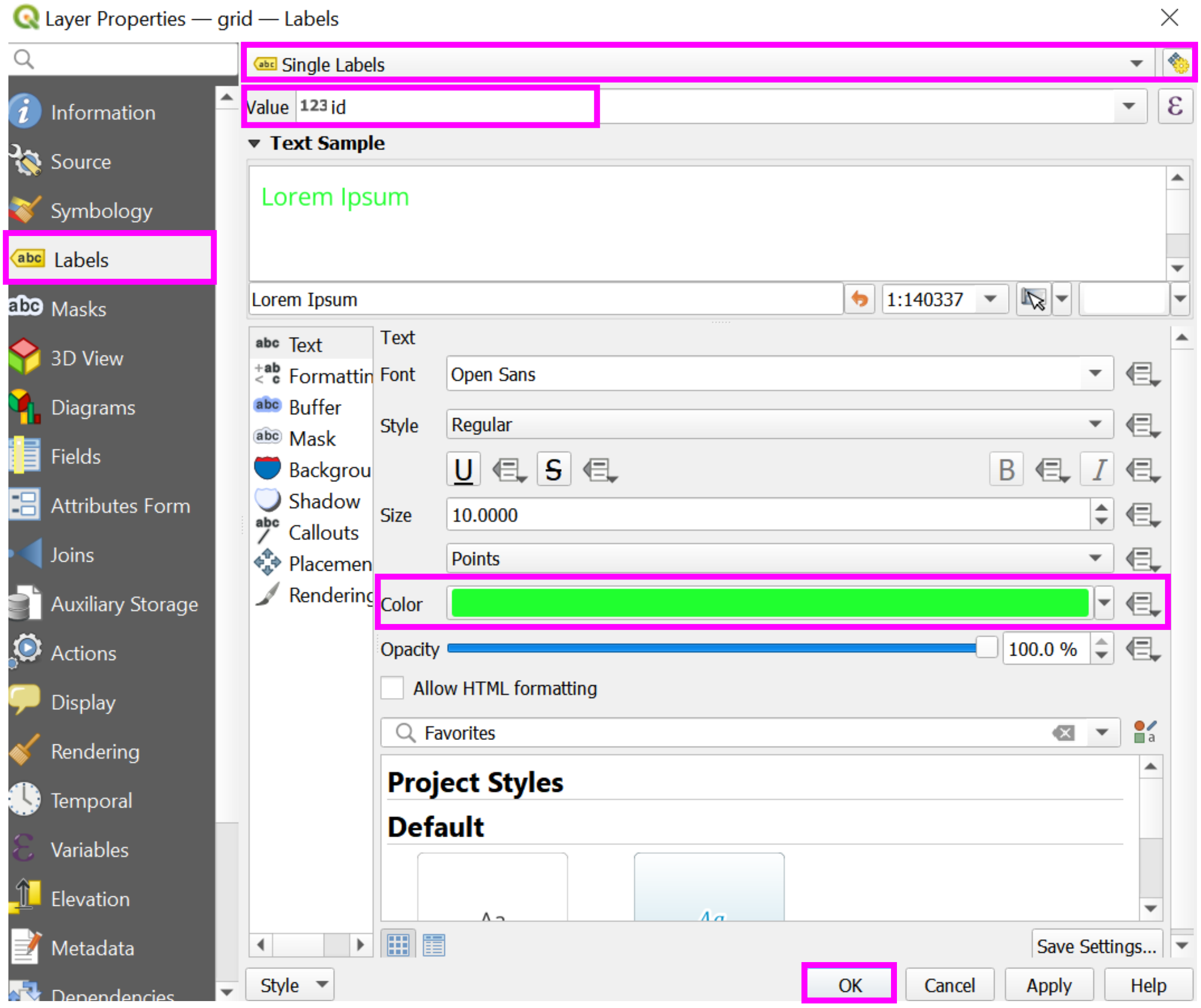

ii. the cell ‘id’ will appear in each cell

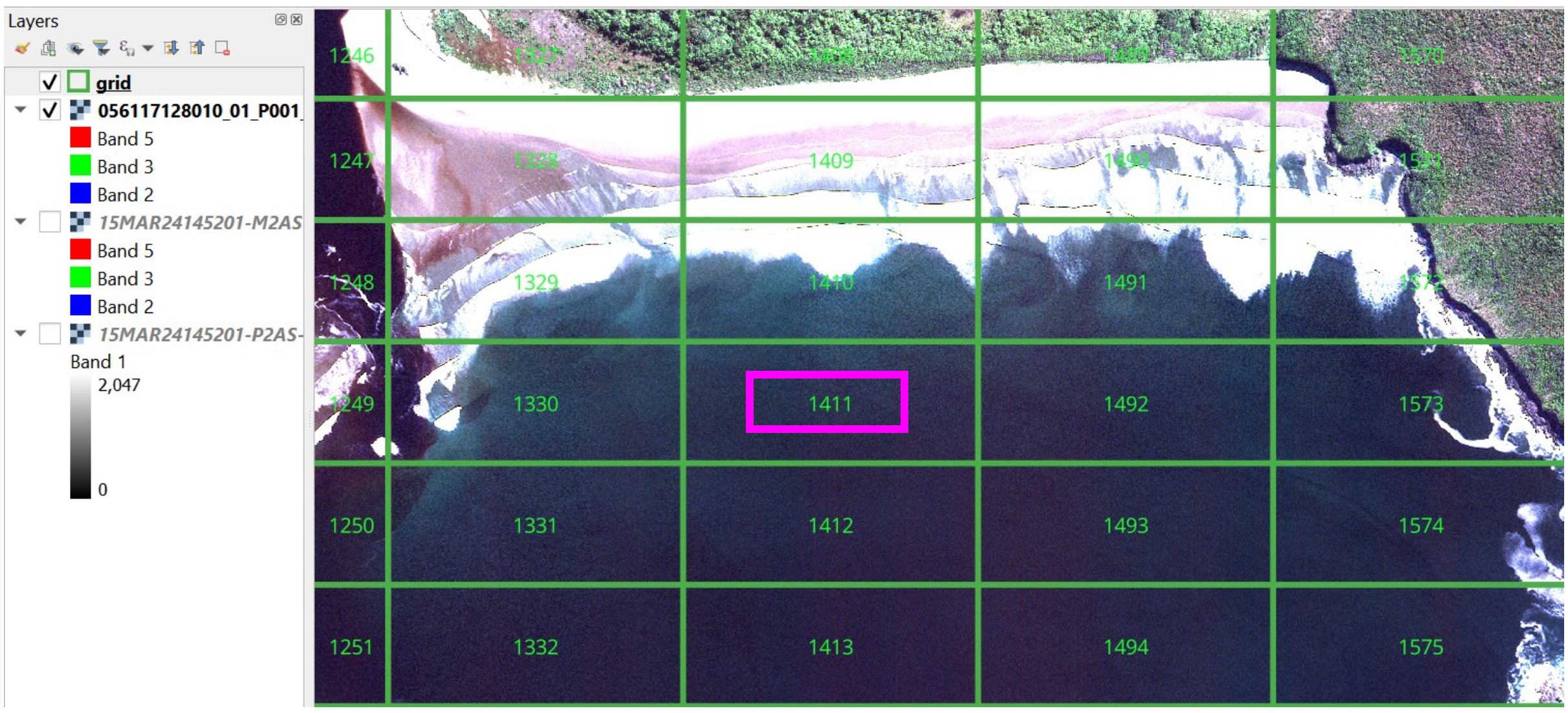

iii. each cell 'id' can be used as a reference to return to when pausing or stopping scanning, note the cell 'id' and direction of scanning

iv. this method is most simple to set up and an easy reference tool, however, when zoomed to the base zoom scale the 'id' number remains visible, which could conceal a feature. To overcome this, would require intermittently turning the grid layer on and off when scanning, using the checkbox next to the layer in the 'Layers' pane. Alternatively, by toggling 'No Labels' and 'Single Labels' in the 'Layer Properties' window, adding only when needing to check the cell number and then removing. Ensure if using this method, to have a systematic and safe method of logging grid cell number and direction of scanning to return to

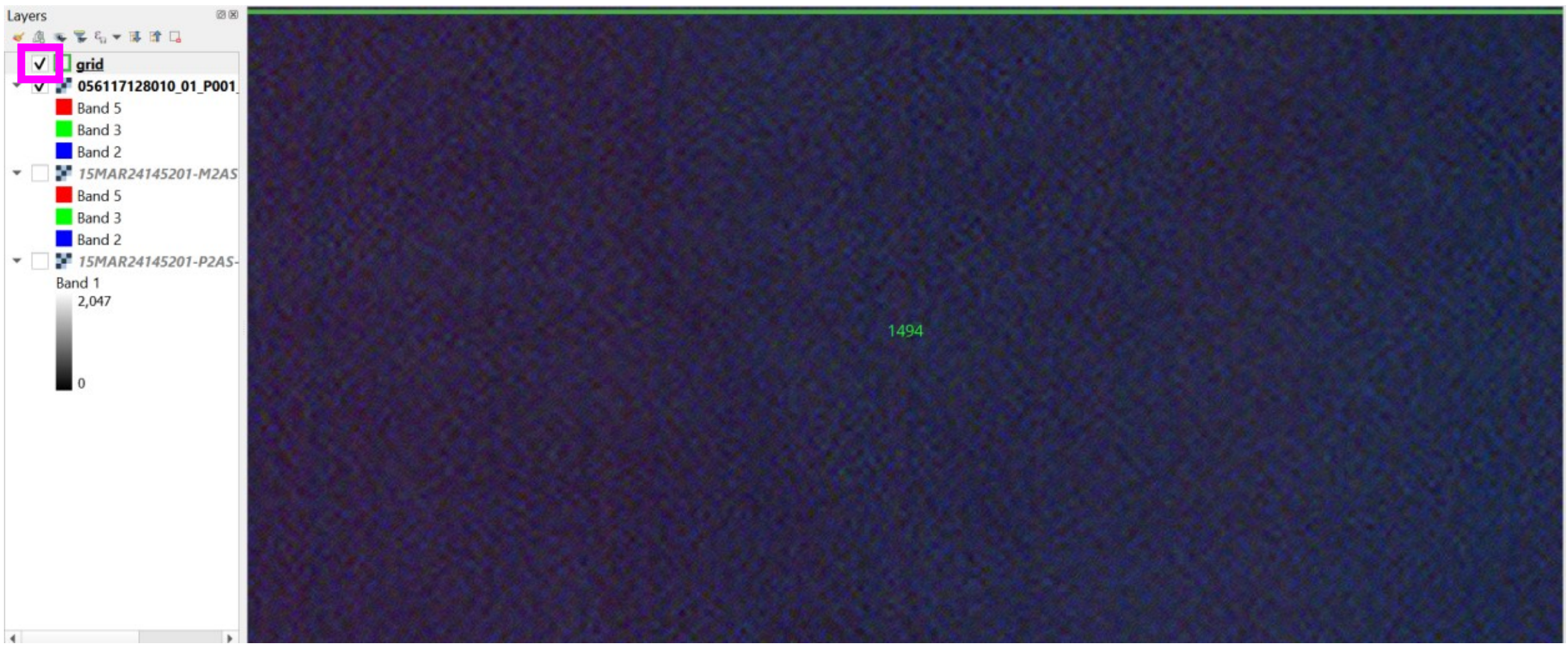

ANNOTATING IMAGERY WITH POINT SHAPEFILES

This section demonstrates how to manually annotate features in satellite imagery with a point shapefile. For the strandings community, a template shapefile is available in supplemental material S4_template_shapefile (access the most up to date version at, https://github.com/PennyJClarke/strandings_from_space). By providing pre-set drop-down values in the attribute table, this template shapefile aims to ensure standardised terminology is used throughout the community, for data sharing and collaborative working practices. However, we do recognise this may be restrictive for the needs and priorities of local communities. Given the intent of the workflow is to empower locally driven monitoring efforts and to combat parachute science, the annotation template has been designed to allow customisation and enable users to remove standards and add/propose new standards.

In case of errors encountered importing the template, as guidance for adding new standards, or for developing new templates for non-strandings projects, instructions on how to create a point shapefile and template attribute table with drop-down options is available in, appendix 2: creating a Template Shapefile. For guidance on removing attributes within the template not required by a project, see section 18.

Before conducting any annotations, observers should train their eye to spy on strandings from space, view the descriptions and visual guides of the template shapefile fields and standardised values, in supplemental material S3 (access the most up to date version at, https://github.com/PennyJClarke/strandings_from_space). A single shapefile is provided for annotation of all features (including target and confounding features, for example, whale and organic beach debris, such as algae), as QGIS allows for filtering data by attribute ('Selected features only') when working with or extracting data ('Selected features only', see image below), as would extracting the data using a coding software like Python.

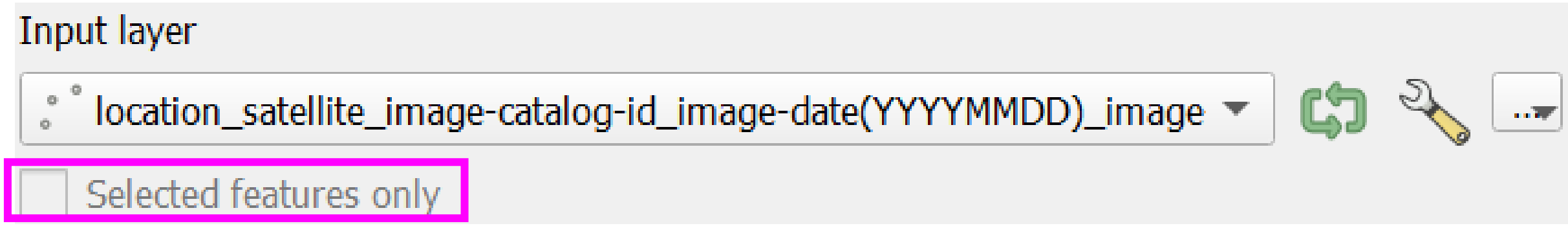

16. Importing template point shapefile
    a. from the downloaded supplemental material S4_template_shapefile, (or for the most up to date version visit, https://github.com/PennyJClarke/strandings_from_space, and locate the 'qgis' folder > 'template_shapefile'), drag and drop the file with a .shp extension, into the 'Layers' pane of the QGIS project

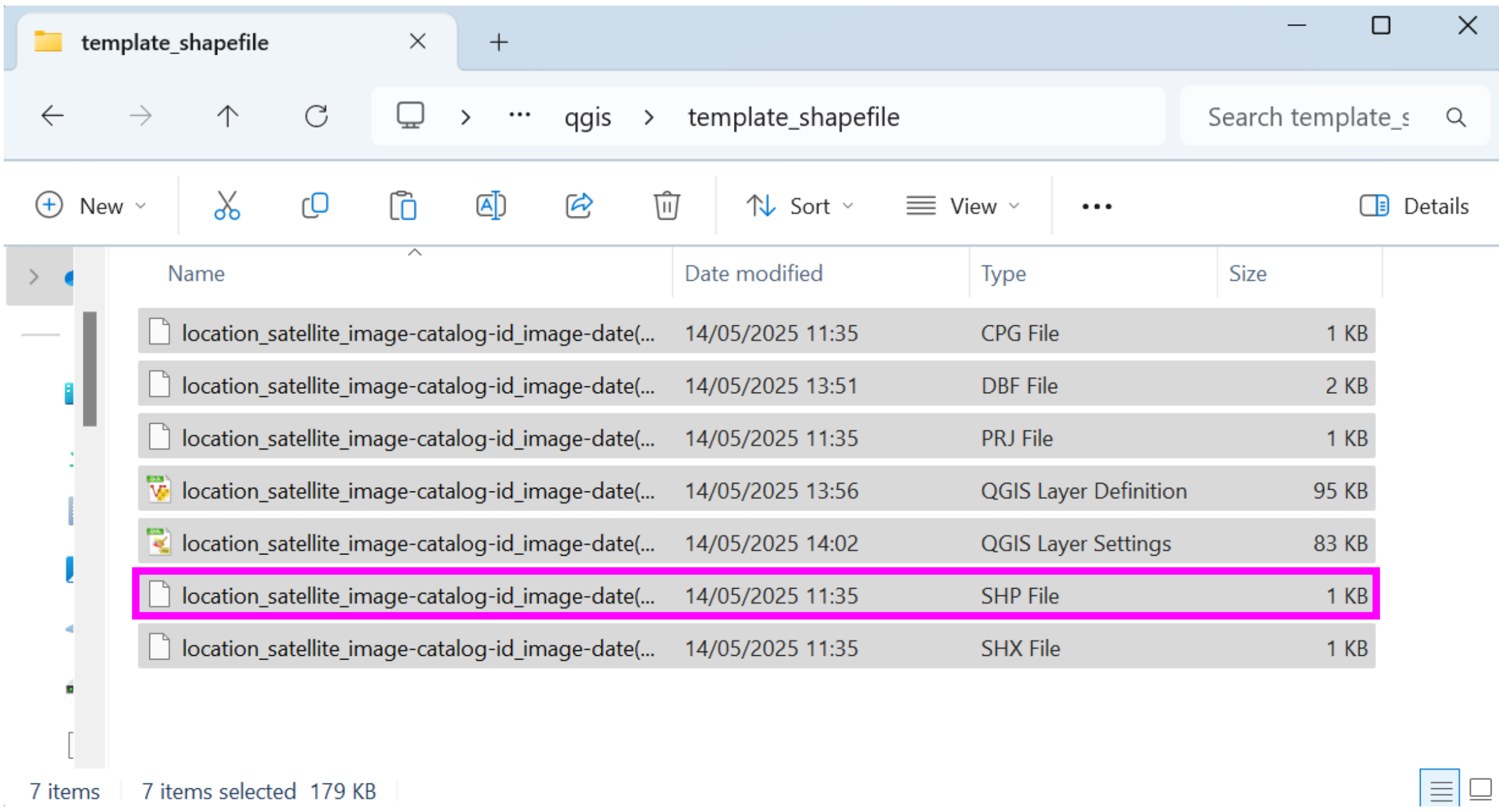

    b. check the shapefile loaded correctly by right clicking the shapefile layer in the 'Layers' pane > 'Properties' > 'Attributes Form'. If loaded correctly, 31 fields (0-30) will already exist under the 'Fields' menu. Under the 'Attributes Form', when selecting a field for example, 'feature', the 'Widget Type' will be set to 'Value Map' with a list of 'Values' and 'Description' already pre-set

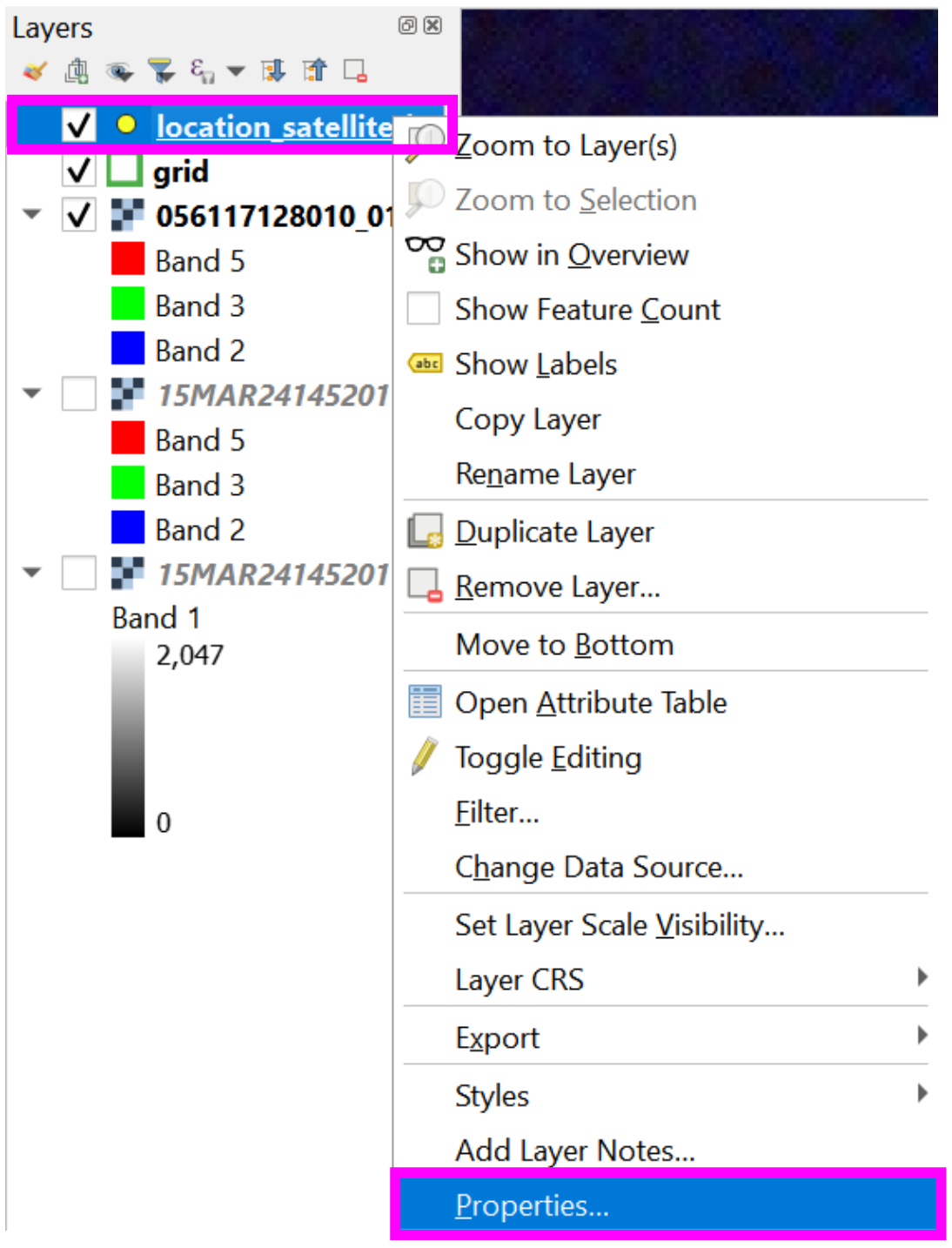

Layers
location_satellite
grid
056117128010_01
Band 5
Band 3
Band 2
15MAR24145201
Band 5
Band 3
Band 2
15MAR24145201
Band 1
2,047
0
Zoom to Layer(s)
Zoom to Selection
Show in Overview
Show Feature Count
Show Labels
Copy Layer
Rename Layer
Duplicate Layer
Remove Layer...
Move to Bottom
Open Attribute Table
Toggle Editing
Filter...
Change Data Source...
Set Layer Scale Visibility...
Layer CRS
Export
Styles
Add Layer Notes...
Properties...

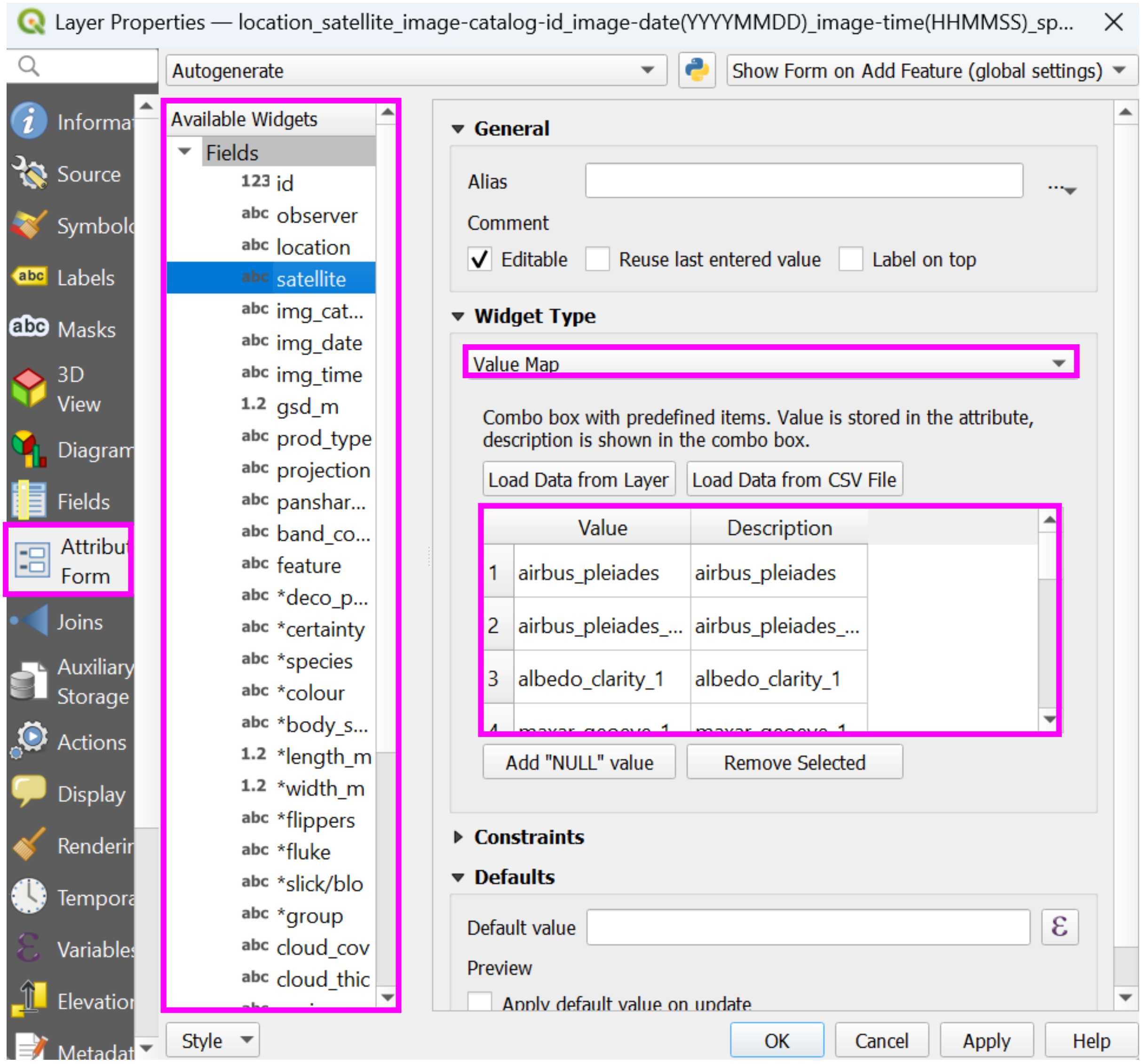

Layer Properties — location_satellite_image-catalog-id_image-date(YYYYMMDD)_image-time(HHMMSS)_sp...
Autogenerate
Show Form on Add Feature (global settings)
Information
Source
Symbology
Labels
Masks
3D View
Diagrams
Fields
Attributes Form
Joins
Auxiliary Storage
Actions
Display
Rendering
Temporal
Variables
Elevation
Metadata
Available Widgets
Fields
id
observer
location
satellite
img_cat...
img_date
img_time
gsd_m
prod_type
projection
panshar...
band_co...
feature
*deco_p...
*certainty
*species
*colour
*body_s...
*length_m
*width_m
*flippers
*fluke
*slick/blo
*group
cloud_cov
cloud_thic
General
Alias
Comment
Editable
Reuse last entered value
Label on top
Widget Type
Value Map
Combo box with predefined items. Value is stored in the attribute, description is shown in the combo box.
Load Data from Layer
Load Data from CSV File
Value
Description
1 airbus_pleiades airbus_pleiades
2 airbus_pleiades_... airbus_pleiades_...
3 albedo_clarity_1 albedo_clarity_1
Add "NULL" value
Remove Selected
Constraints
Defaults
Default value
Preview
Apply default value on update
Style
OK
Cancel
Apply
Help

*Please note: If the template entries in the 'Value Map' do not contain options needed for your project, add to the list by typing a 'Value' and 'Description' to the next line in the entries for a given field. Ensure any new entries are lower case and any spaces between words are replaced with a hyphen (_). ***As this template is a base list of entries for the strandings community and can be expanded upon, if you do add new entries, please inform the corresponding author, for these to be added to the core (most up to date) template for download at https://github.com/PennyJClarke/strandings_from_space***.*

It is likely the 31 column names ('Fields') will appear on importing the template, however, the 'Widget Type' / 'Value Map' entries are absent and return to the default 'Text Edit' format. There are two options to resolve this:

c. in the 'Properties' window (already open), in the drop-down in the bottom left corner 'Style', click and select 'Load Style…'

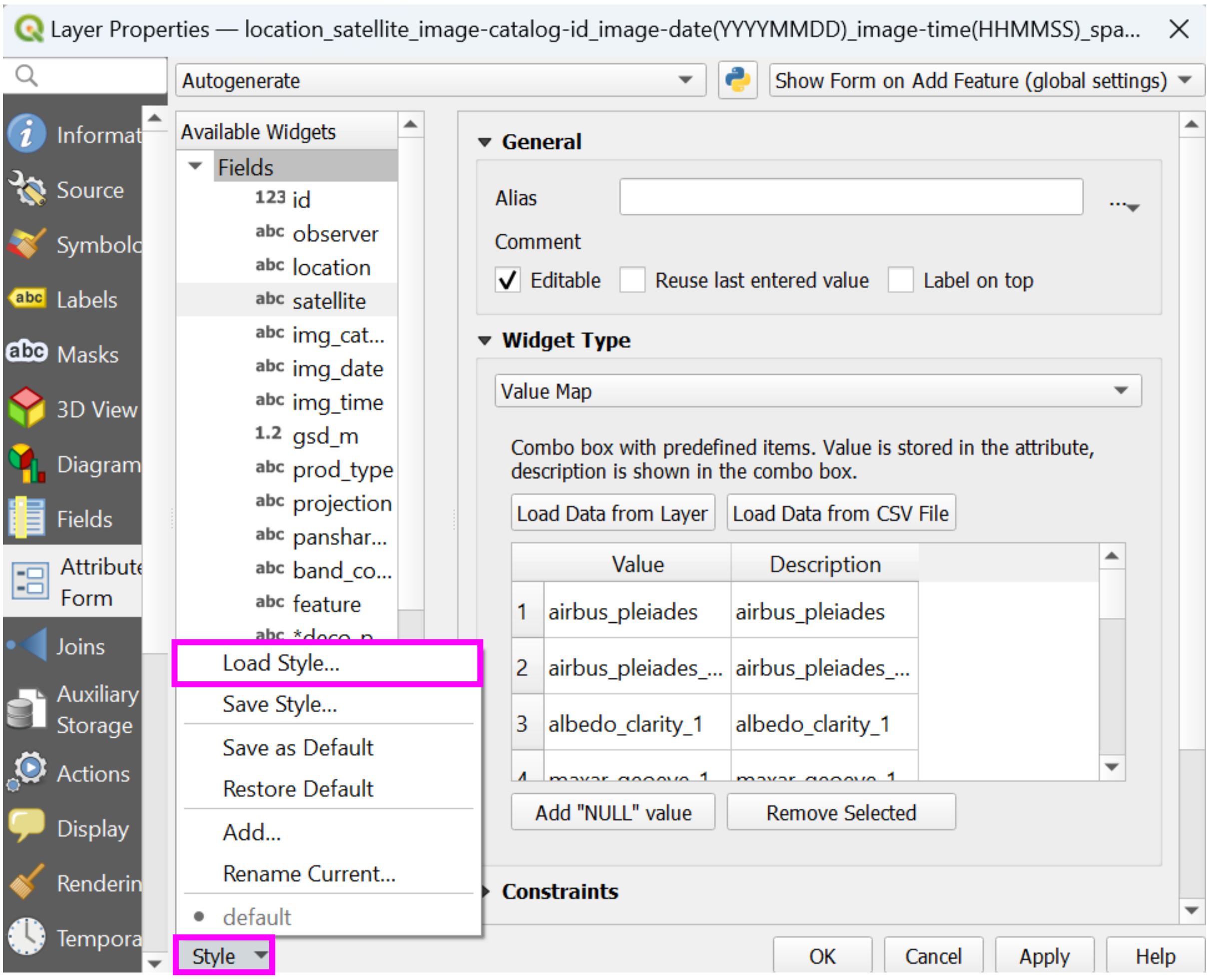

d. in the 'Database Styles Manager' set the 'Load style' to 'From File', click the browse icon (3 dots) next to 'File' and select the 'QGIS Layer Settings' file from within supplemental material S4_template_shapefile (or for the most up to date version visit, https://github.com/PennyJClarke/strandings_from_space, and extract from the 'strandings_from_space' > 'qgis' > 'template_shapefile' folder). Ensure all 'Categories are checked in the 'Database Styles Manager', select 'Load Style'

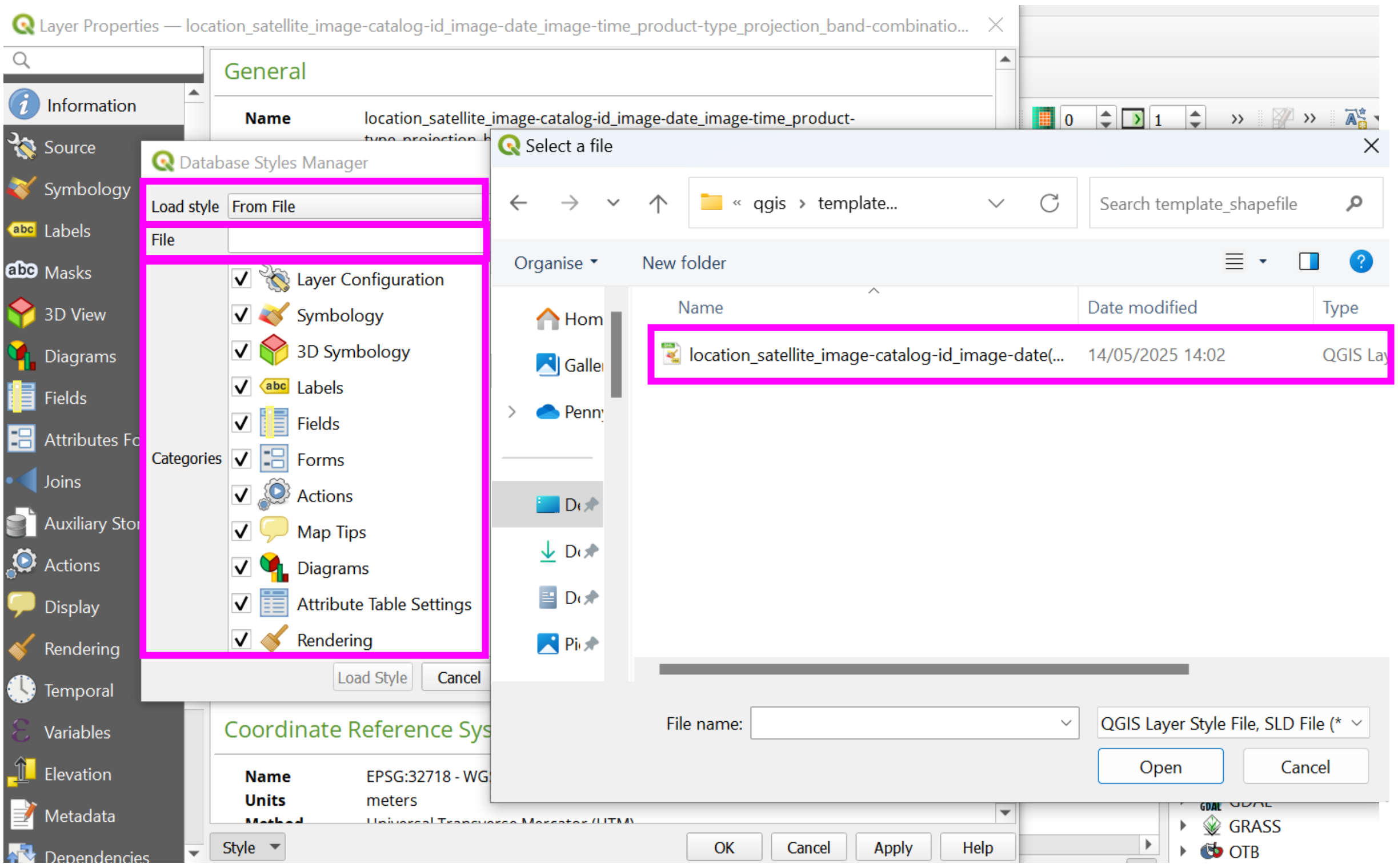

This should resolve the missing 'Value Map' entries, however, if loading the layer style settings does not solve the errors, guidance in appendix 2: creating a Template Shapefile can be followed to import the value map entries manually.

17. Re-projecting the template point shapefile
    a. the template shapefile has been generated with geographic coordinate system (EPSG: 4326 – WGS 84). Therefore, the shapefile will need re-projecting before use to the projected coordinate system of the image for a given project. In the 'Processing Toolbox', select 'Vector general' > 'Reproject layer'

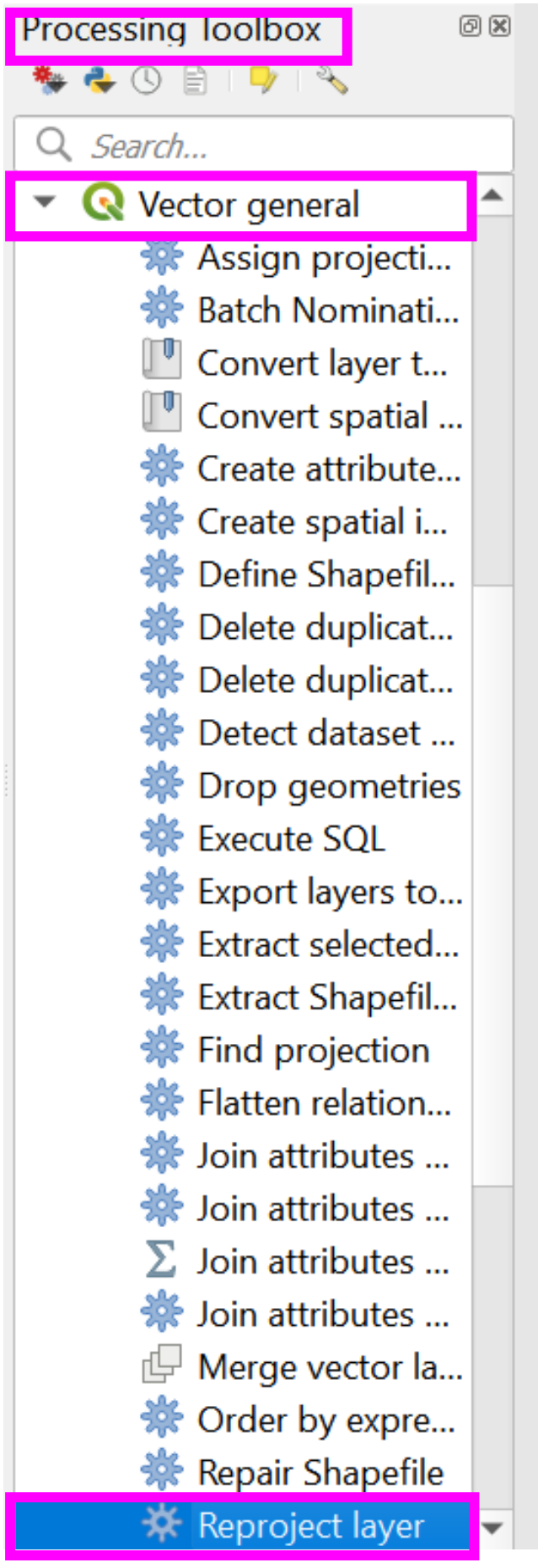

b. in the 'Reproject layer' window, select the template shapefile as the 'Input layer', in the 'Target CRS', using the 'Select CRS' icon (globe), search for the relevant CRS that matches your image (this should be available as 'Project CRS' in the dropdown options). In the 'Reprojected' cell, save the template shapefile to a suitable location, with the name of your relevant project details (use the standardised naming convention section '**Error! Reference source not found.**') and 'Run'

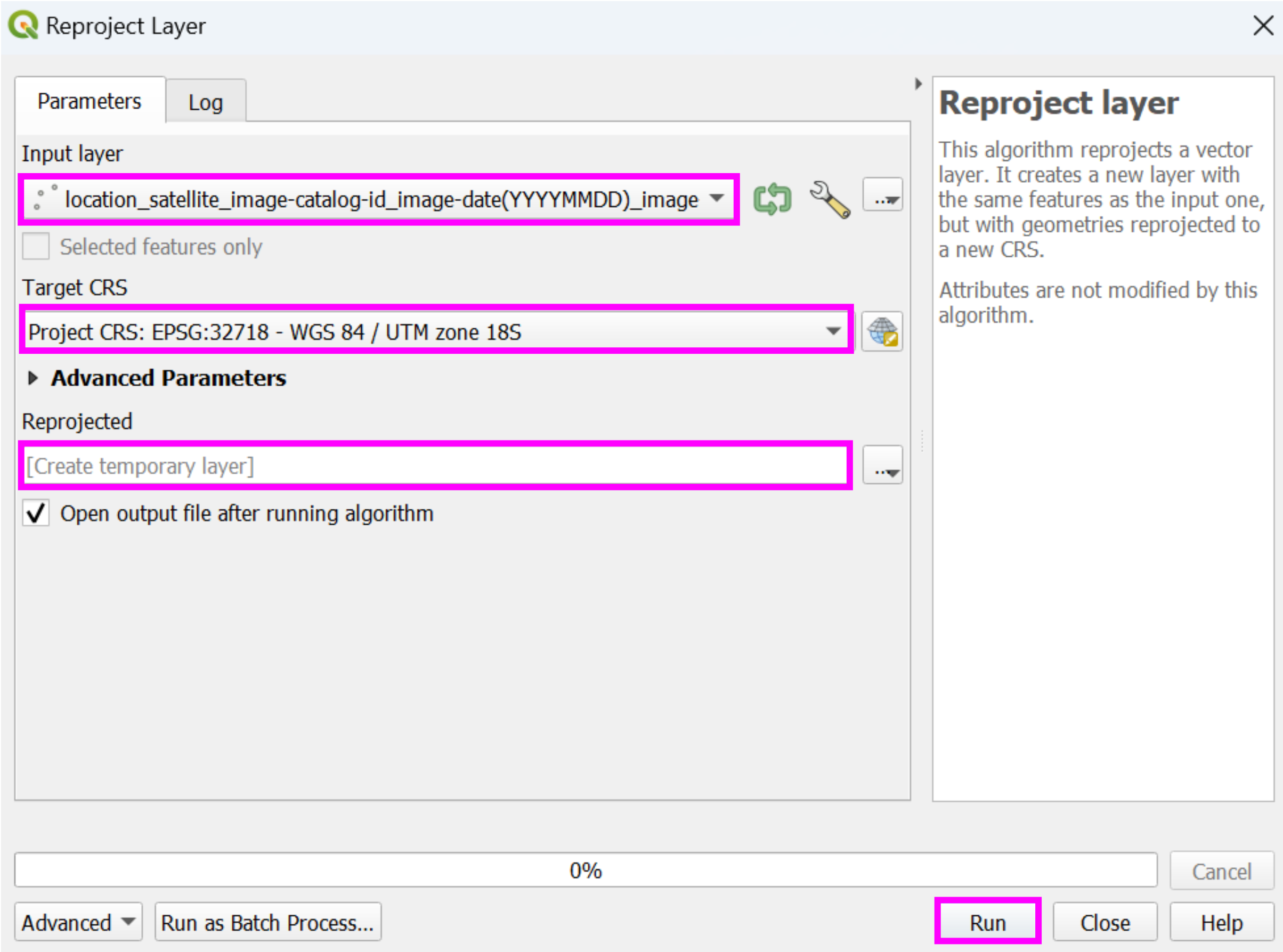

c. Once the re-projection is complete, you may be required to reload the template style as per section 16.c andd.

d. the new shapefile will appear in the 'Layers' menu. The appearance (shape and colour) of annotations can be amended by, right clicking the shapefile in the 'Layers' pane, 'Properties' > 'Symbology' (high contrasting colours to your raster are recommended, for projects using multiple shapefiles for annotation, consider using different shapes and colour-blind friendly colour palettes)

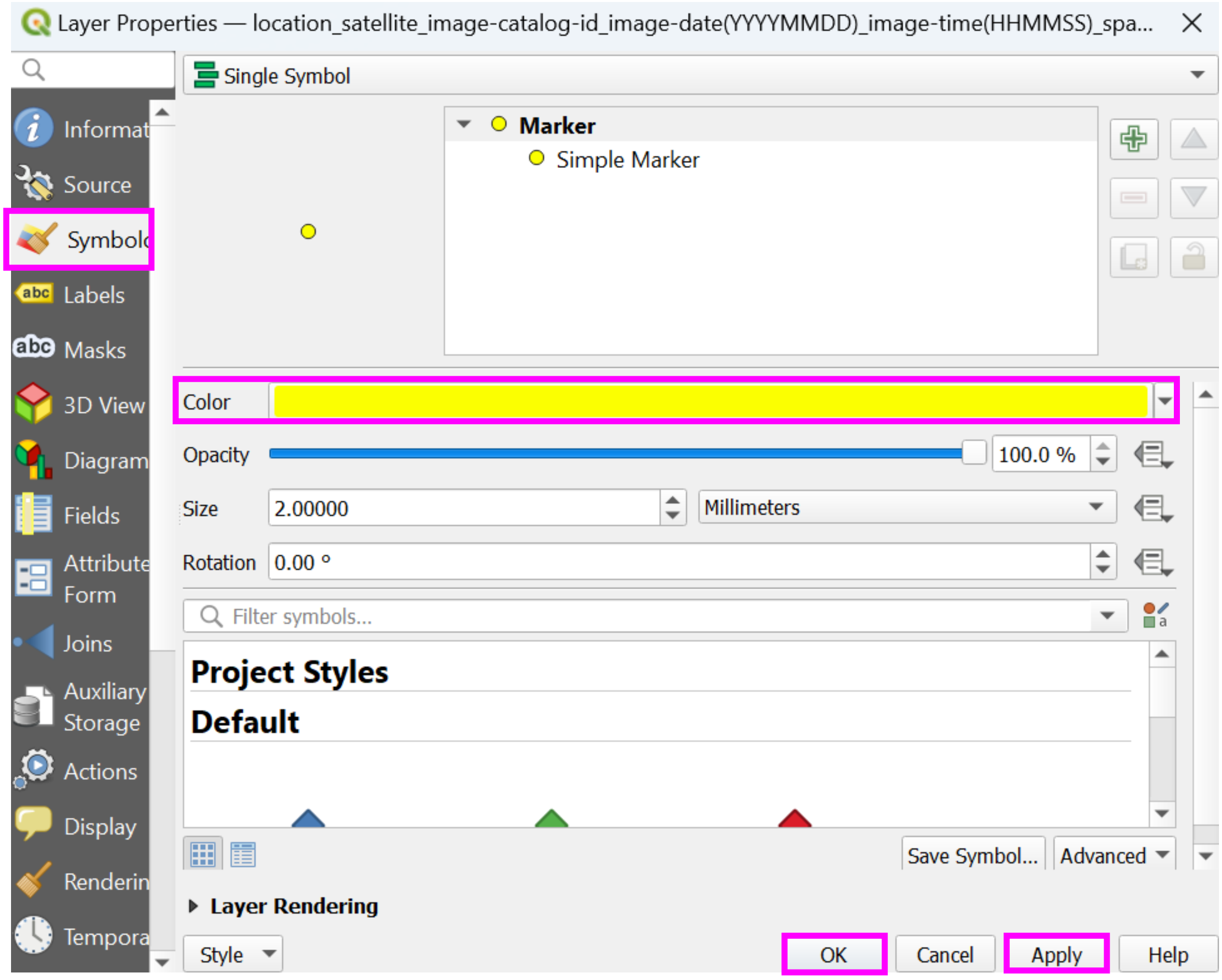

18. Annotating features using a point shapefile

Once the shapefile is formatted correctly, any attributes not required by a user/project (*e.g.,* optional attributes) can be removed from the shapefile. Right click the shapefile layer in the 'Layers' pane > 'Properties', to open the 'Layer Properties' if not already open. Select the 'Fields' tab, to delete a field switch on 'Toggle editing mode' (yellow pencil icon), click the attribute/column to delete (hold 'Shift' or 'Ctrl' while clicking columns to delete, to select more than one column) and select the 'Delete Field' icon (table with red cross). The chosen attribute/column(s) will be removed, select 'Apply' and 'OK'.

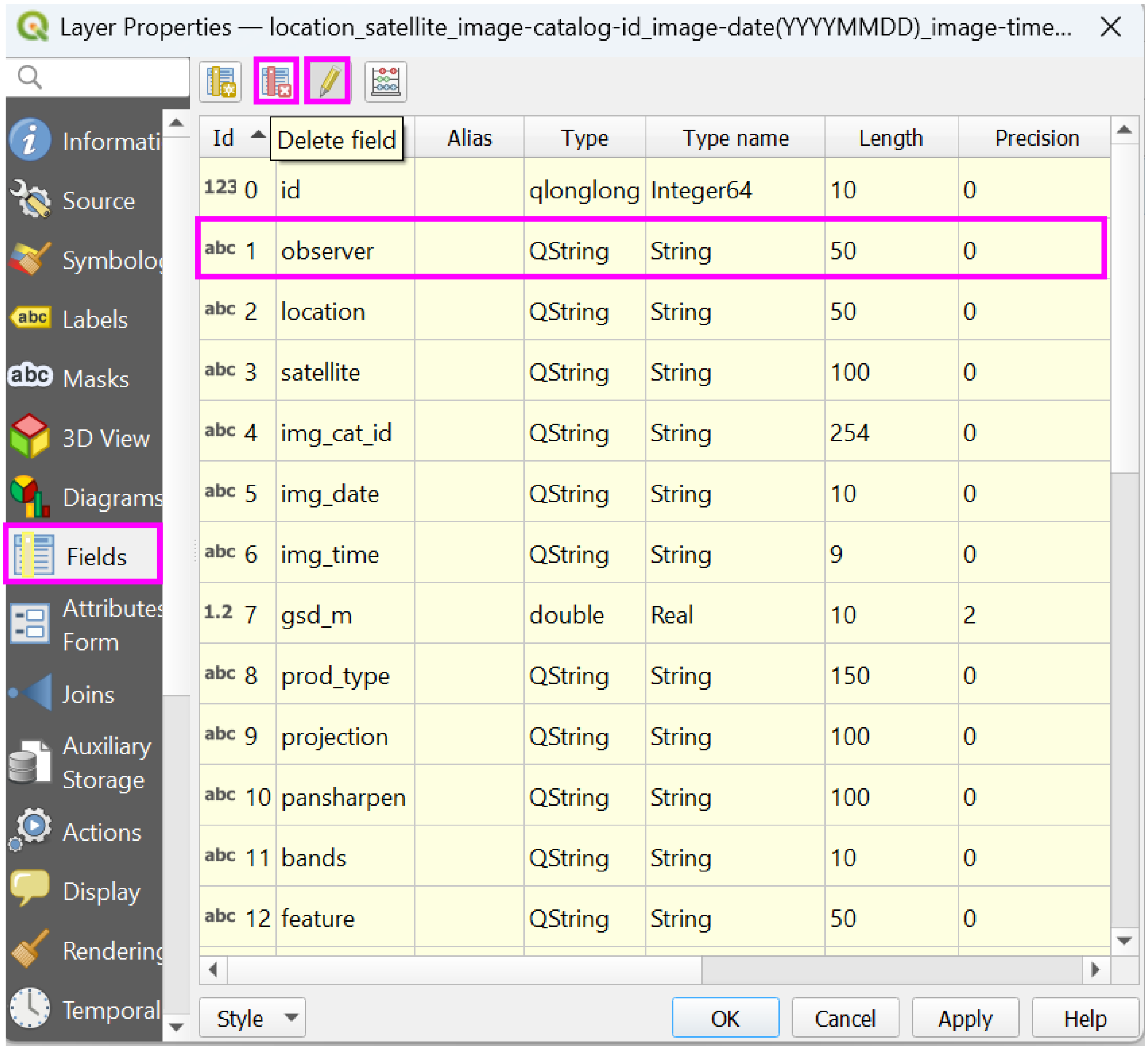

Features can now be annotated:

a. to add a point to a feature, select the point shapefile in the ‘Layers’ pane and click the ‘Toggle Editing’ icon (yellow pencil) on the main toolbar or right click the point shapefile layer and select ‘Toggle Editing’

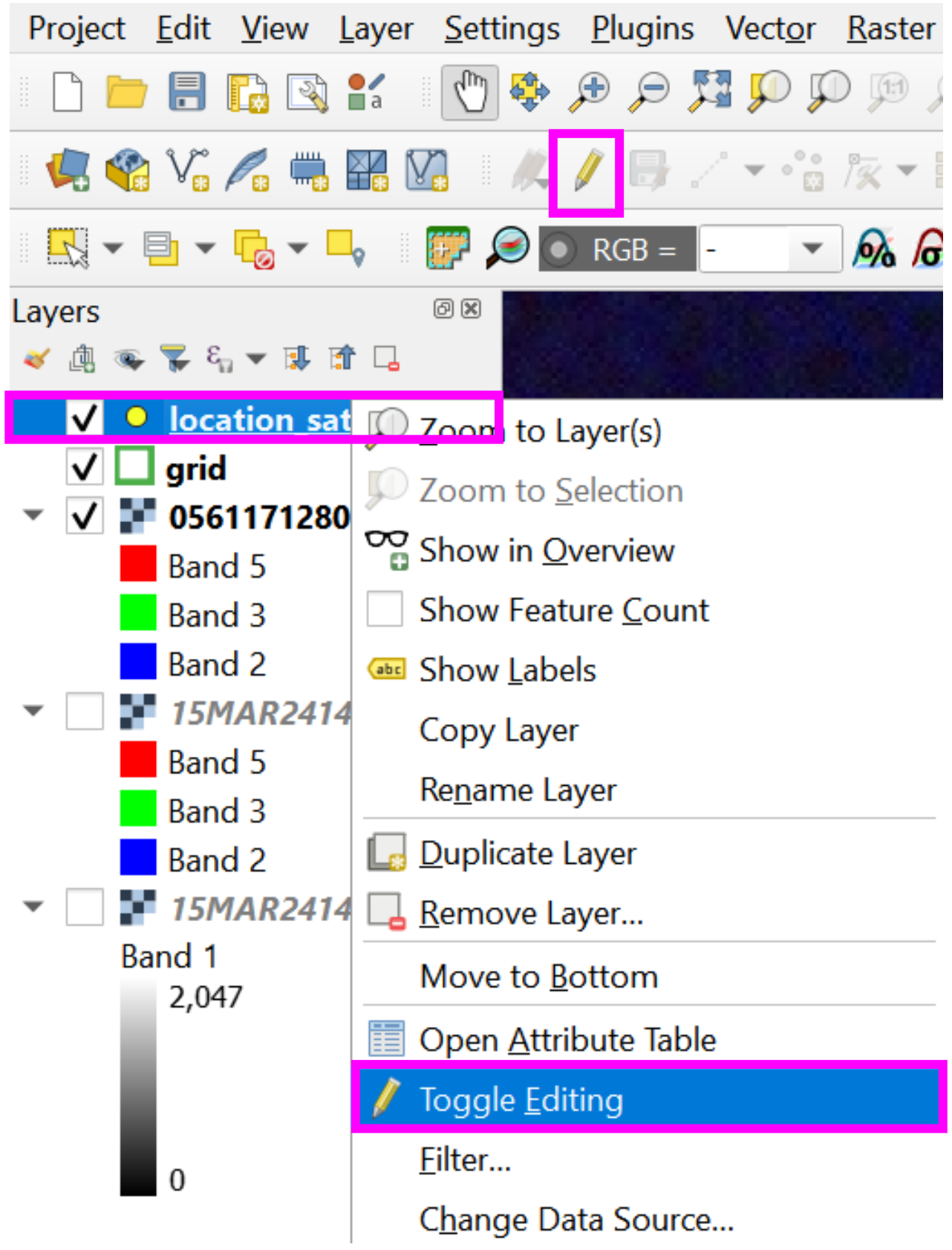

b. once ‘Toggle Editing’ is on, select the ‘Add Point Feature’ icon (3 green points and a yellow square) from the ‘Toggle Editing’ toolbar. When moving over the image, the cursor will appear like a target, place **centrally** (central placement is important for the later conversion of data into other formats for machine learning and for spectral analysis) over the feature of interest, and left click to deposit a point

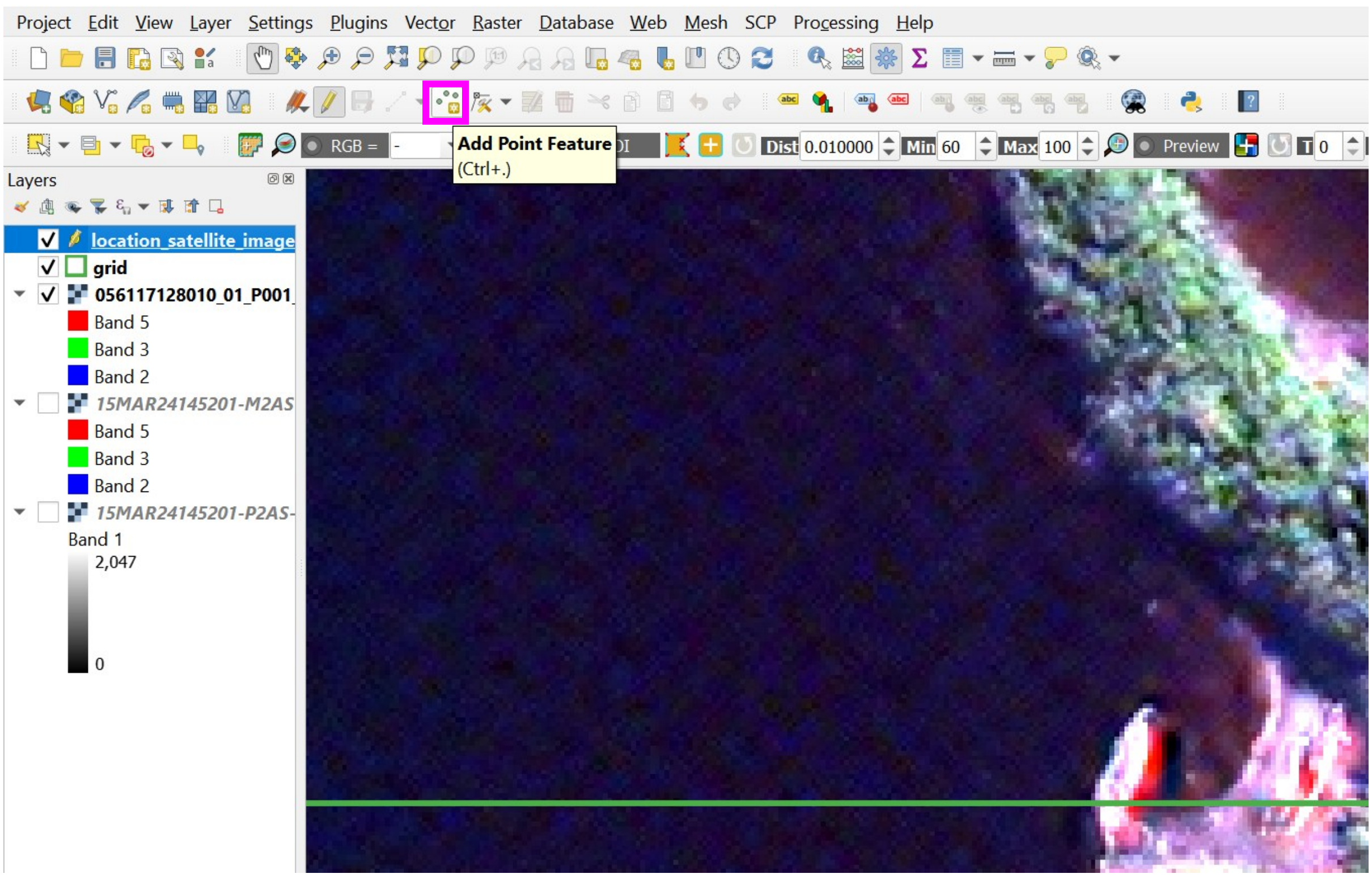

c. a feature attribute pop-up will appear, the feature 'id' will need to be manually populated, as a numerical value, beginning at zero and increase incrementally for each new point. The remaining entries can be completed using the available drop-down options from the inserted template or for entries requiring manual input, as per guidance in supplemental material S3.

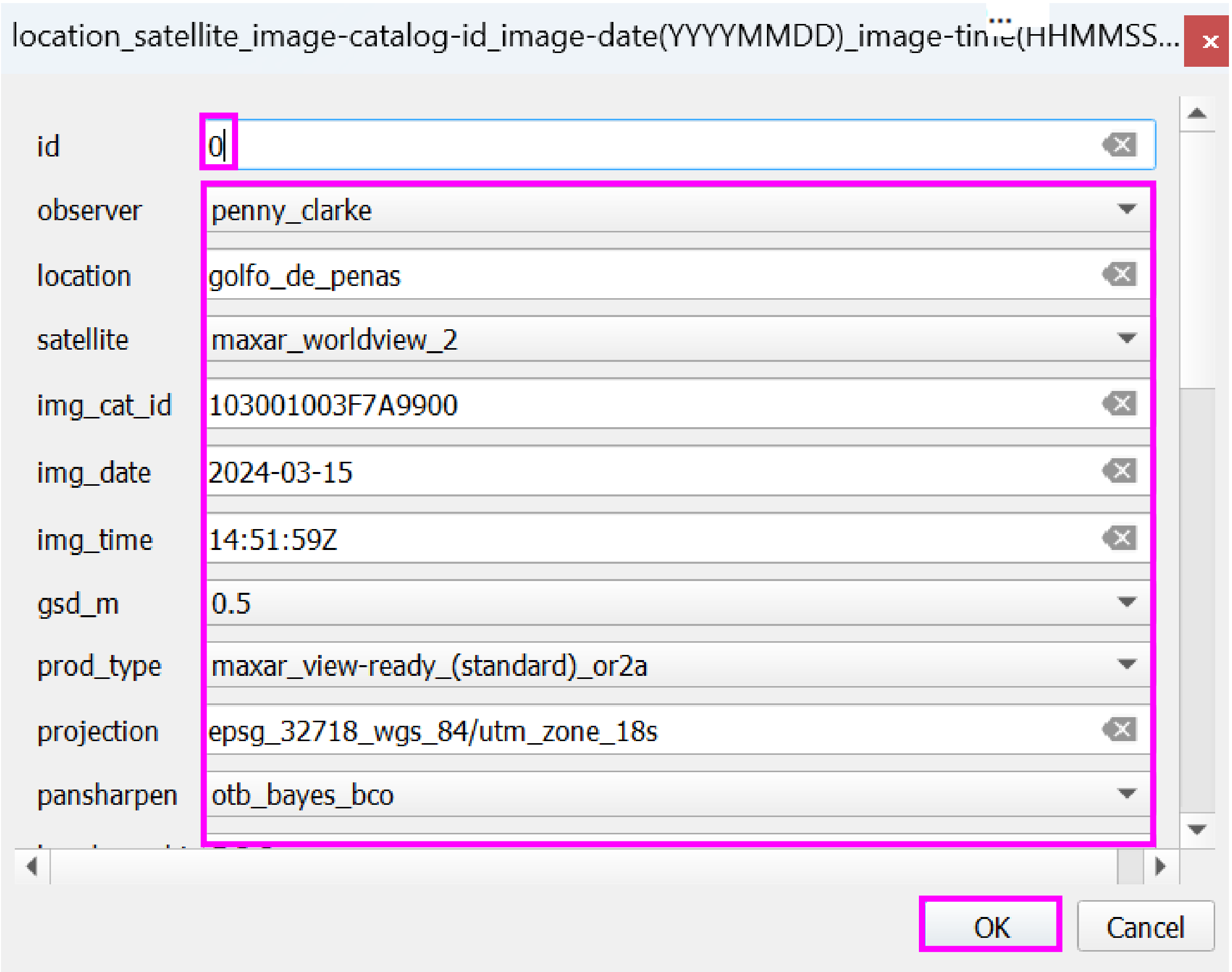

d. for the '*length_m' and '*width_m', measure the feature, this can be done while the feature attribute pop-up remains open. On the main toolbar, select the 'Measure Line' tool, measure the feature length, select 'New' and repeat for the feature width, 'Close' the measure tool and enter the values in the '*length' and '*width' fields. Continue populating the remaining fields and click 'OK' once complete

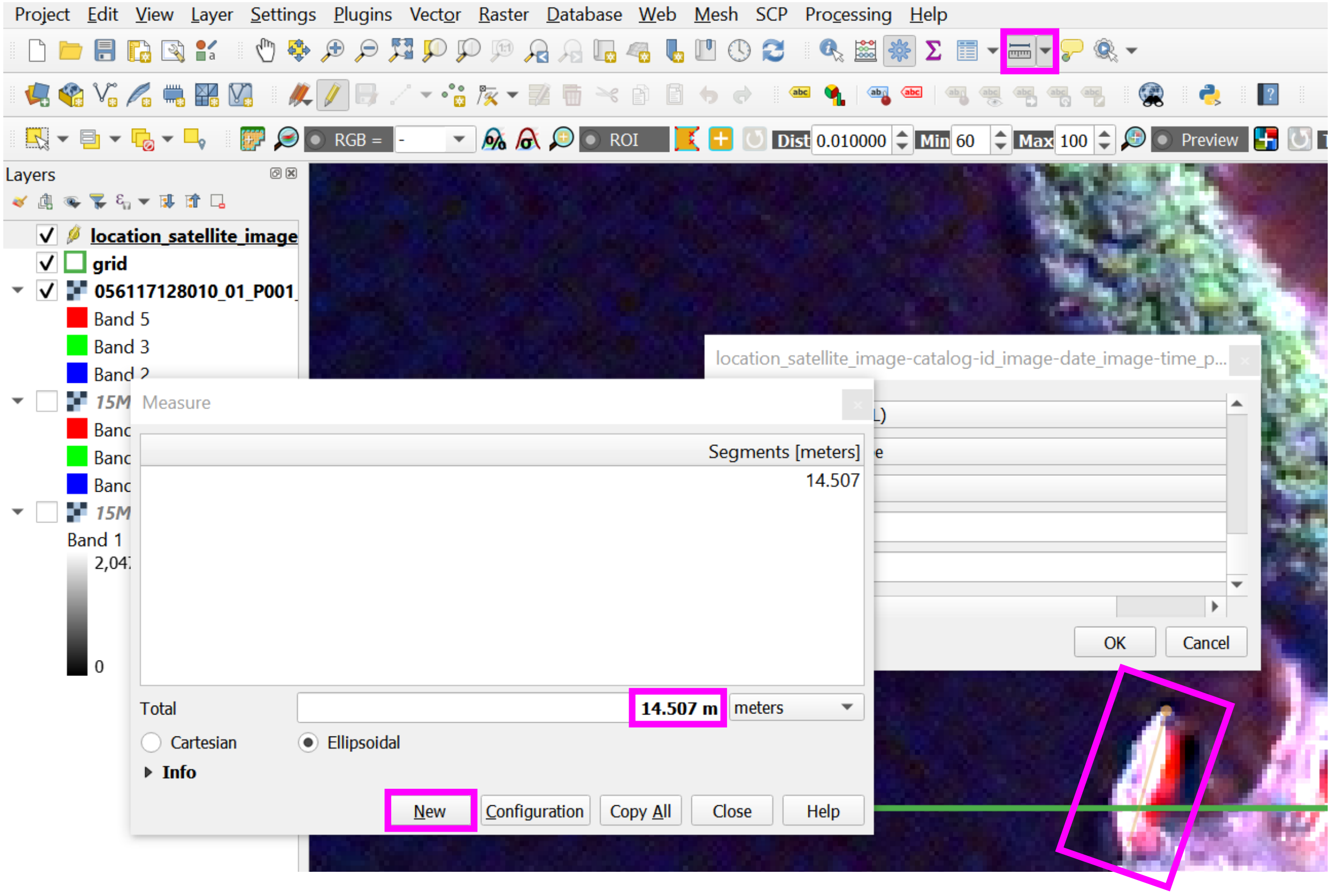
Project Edit View Layer Settings Plugins Vector Raster Database Web Mesh SCP Processing Help
RGB =
ROI
Dist 0.010000
Min 60
Max 100
Preview
Layers
location_satellite_image
grid
056117128010_01_P001
Band 5
Band 3
Band 2
Band 1
location_satellite_image-catalog-id_image-date_image-time_p...
Measure
Segments [meters]
14.507
Total
14.507 m
meters
Cartesian
Ellipsoidal
Info
New
Configuration
Copy All
Close
Help
OK
Cancel

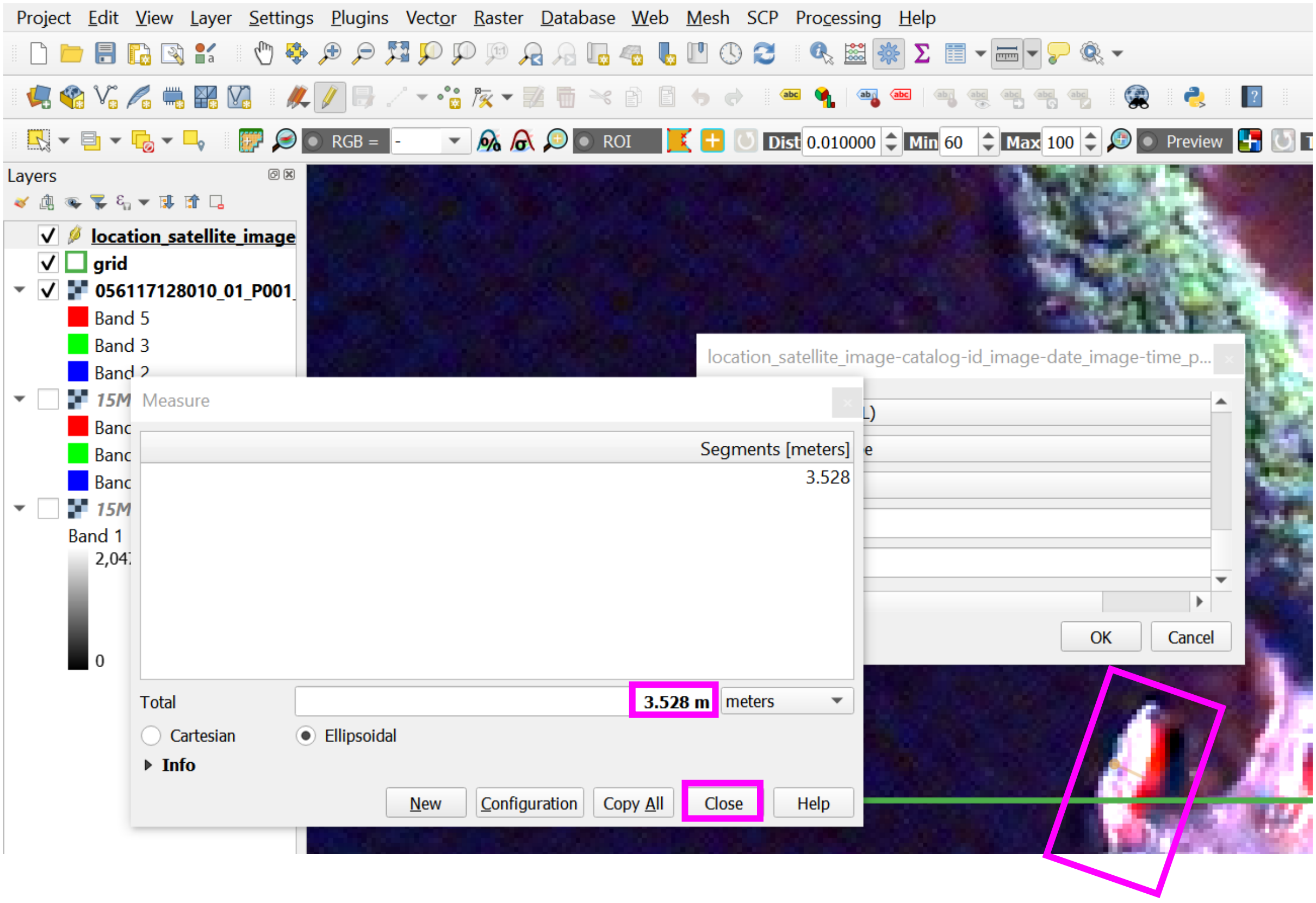
Project Edit View Layer Settings Plugins Vector Raster Database Web Mesh SCP Processing Help
RGB =
ROI
Dist 0.010000
Min 60
Max 100
Preview
Layers
location_satellite_image
grid
056117128010_01_P001
Band 5
Band 3
Band 2
Band 1
location_satellite_image-catalog-id_image-date_image-time_p...
Measure
Segments [meters]
3.528
Total
3.528 m
meters
Cartesian
Ellipsoidal
Info
New
Configuration
Copy All
Close
Help
OK
Cancel

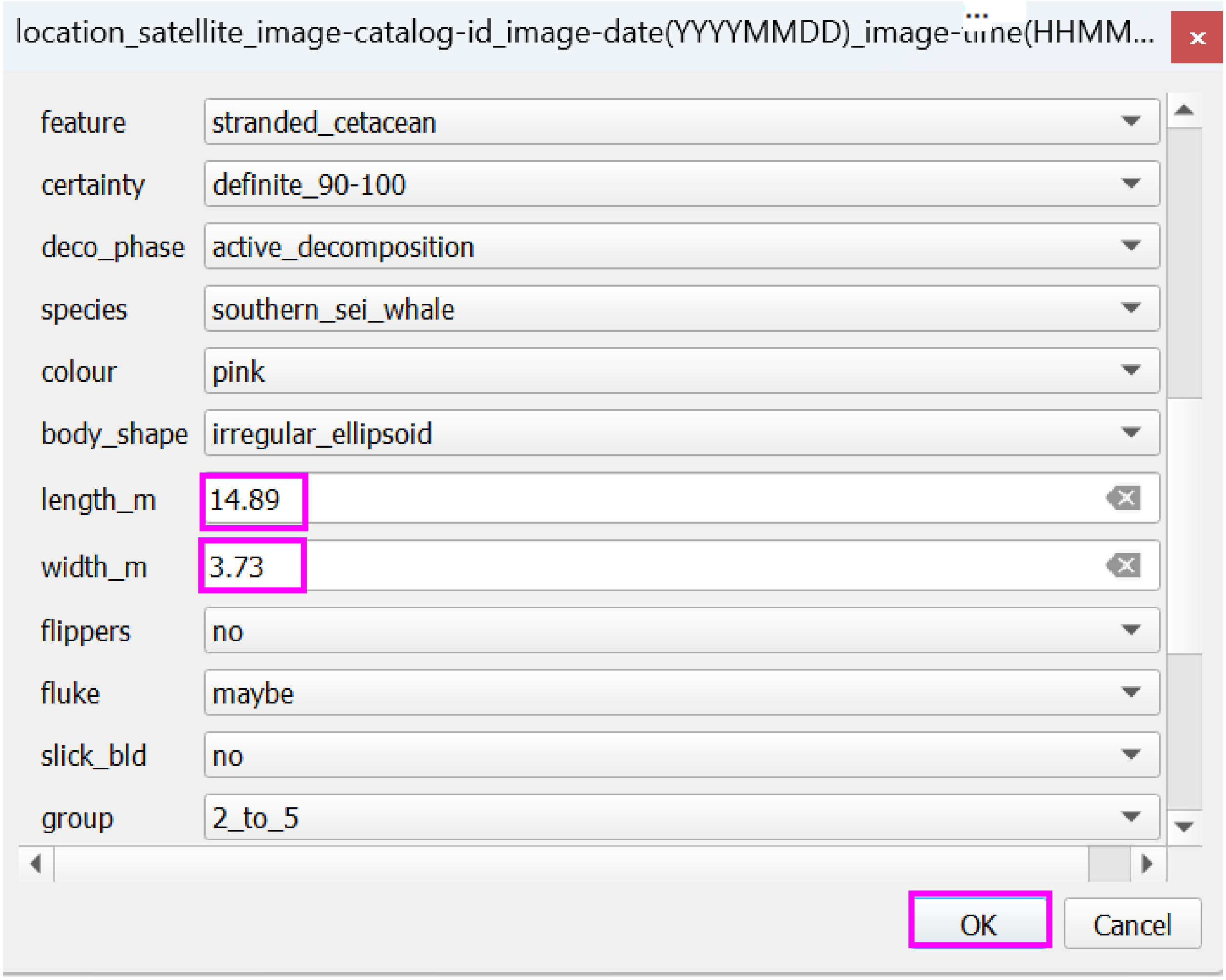

e. after each annotation, save, firstly using the save in the 'Toggle Editing' toolbar, followed by the project save. The features annotated and associated data, will be stored in the attribute table of the shapefile layer. To review the entry, right click the layer in the 'Layers' pane and chose 'Open Attribute Table'. The attribute table can be docked to the main page so it is visible while working, using the 'Dock Attribute Table' icon

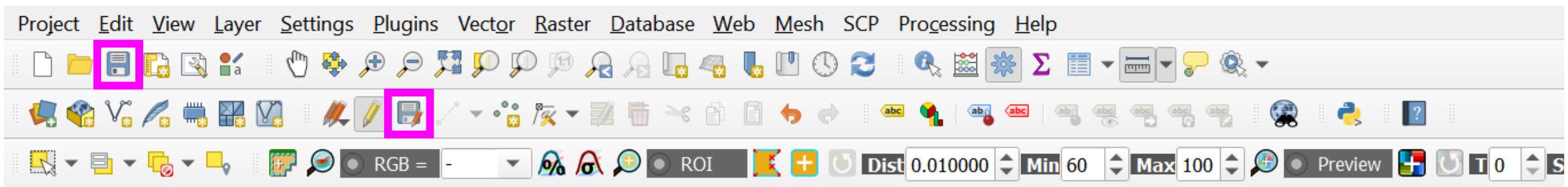

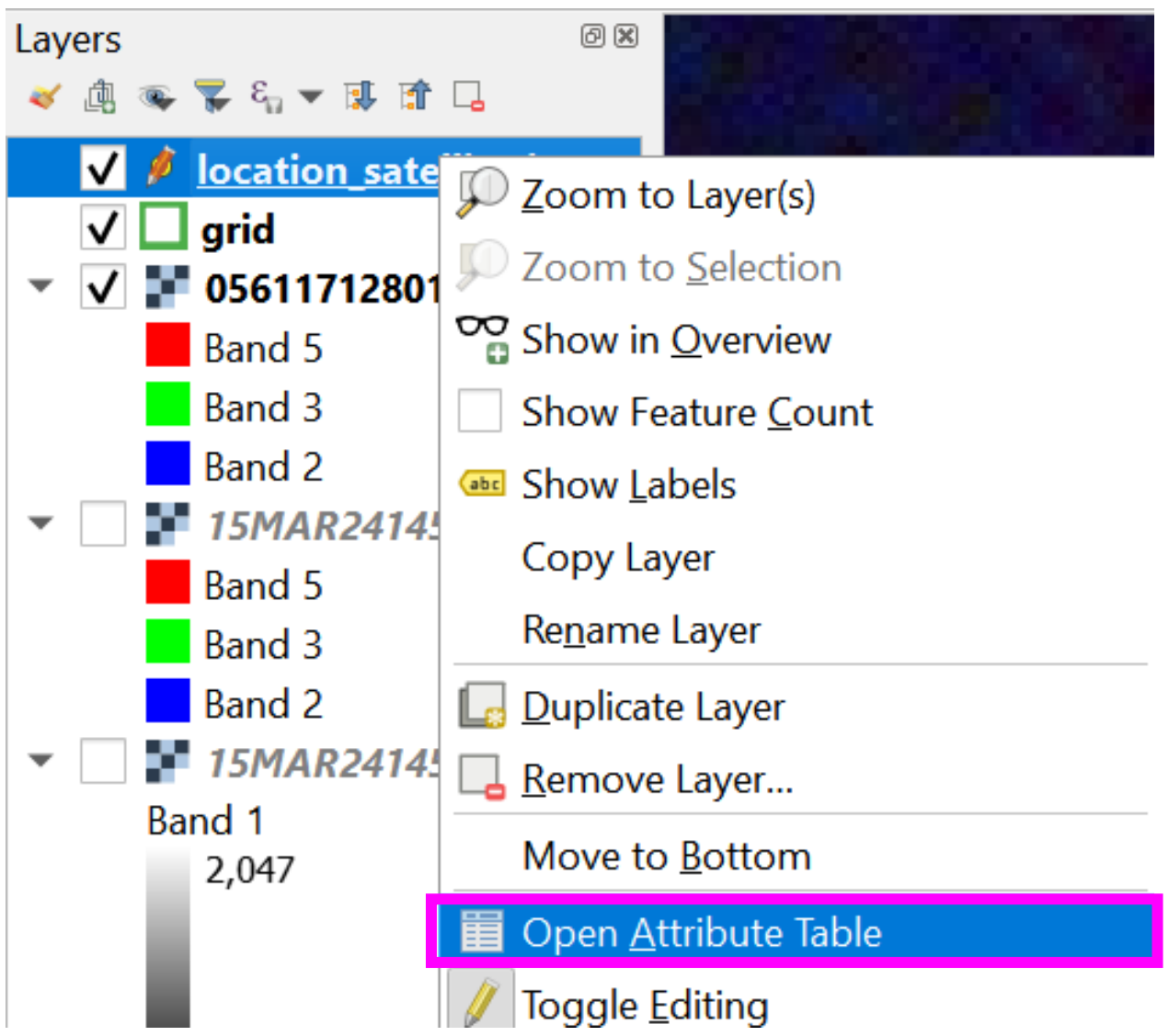

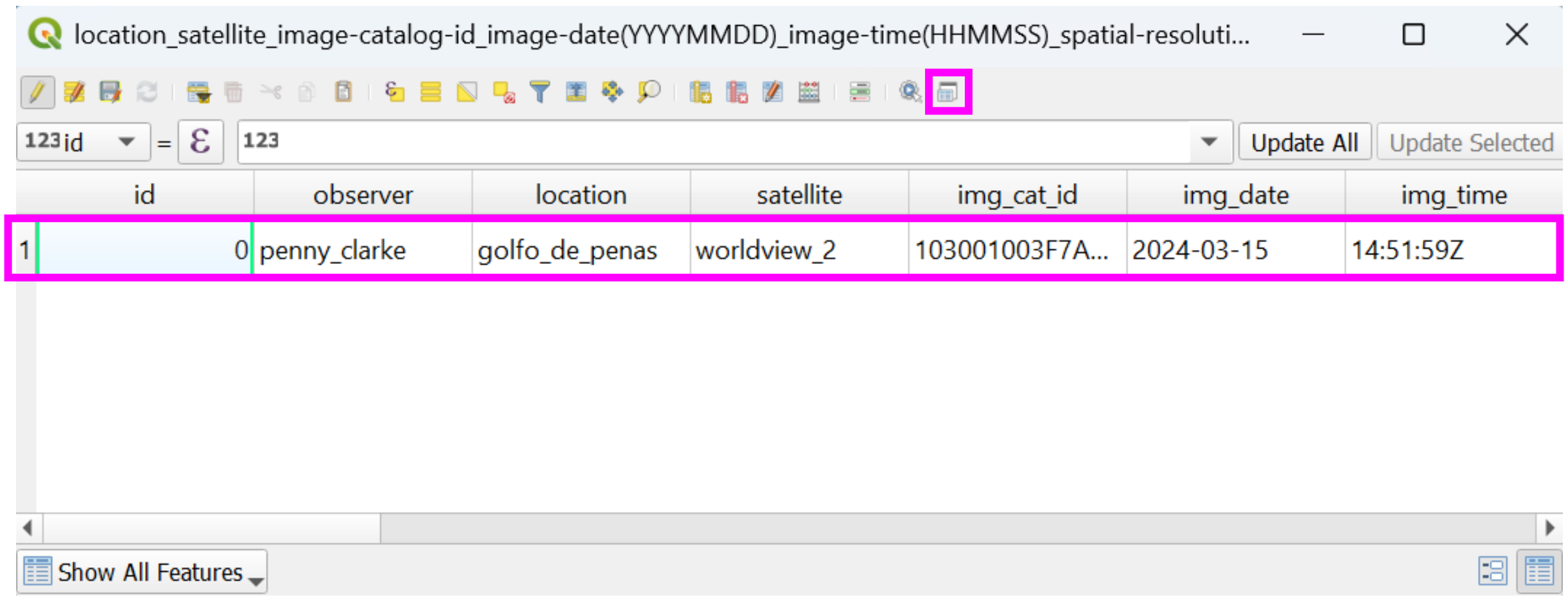

f. for each subsequent annotation, the fields should automatically populate with the last entries. This is helpful to reduce time repeating consistent entries, but observers need to be careful to ensure attributes are amended where necessary. If the fields do not populate automatically for new annotations, in the point shapefile ‘Layer Properties’ > ‘Attributes Form’, the attribute can be formatted to ‘Reuse last value entered’, by checking the box.

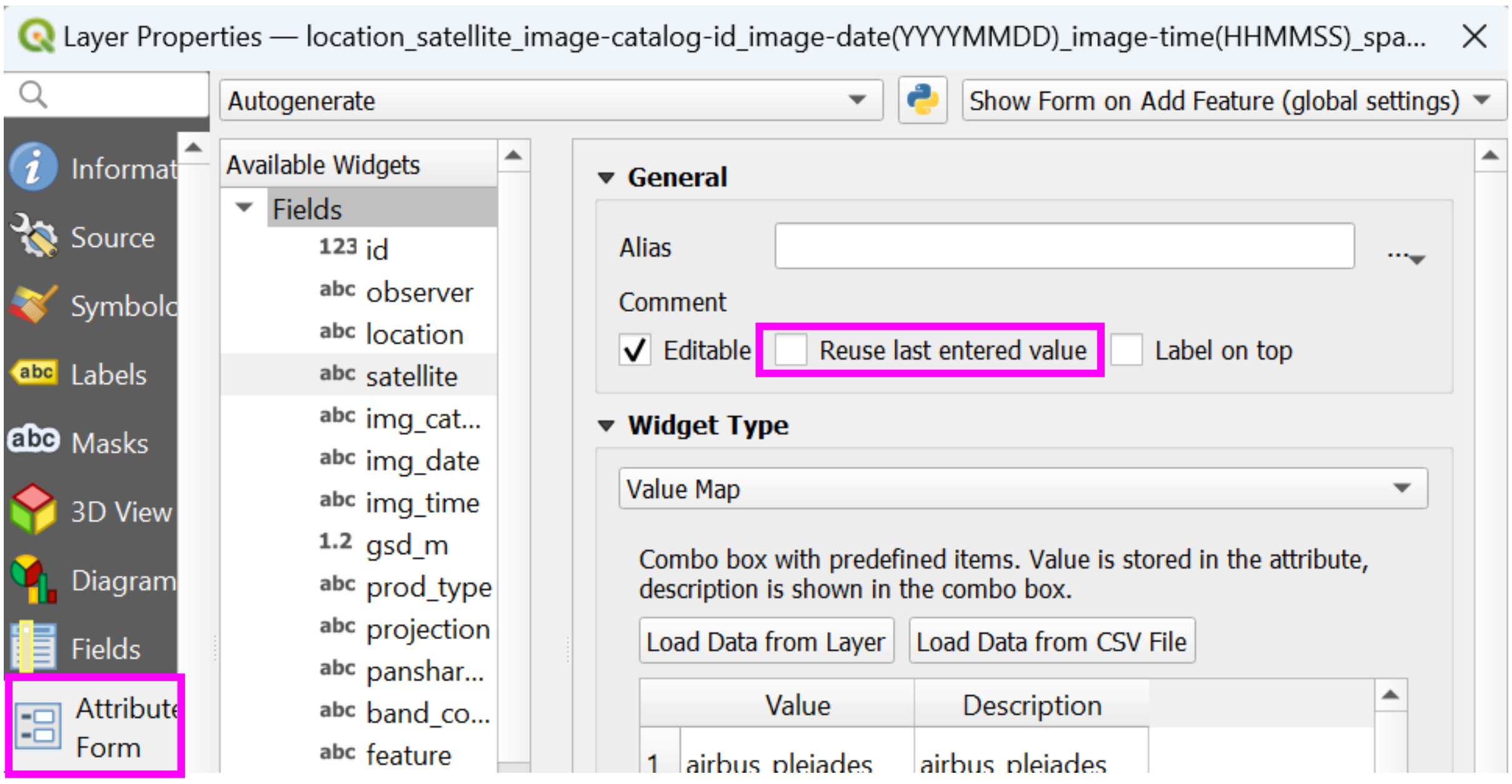

g. to edit or delete annotations, in 'Toggle Editing' mode, click the 'Select Features by Area or Single Click' icon (yellow square with arrow) from the main toolbar

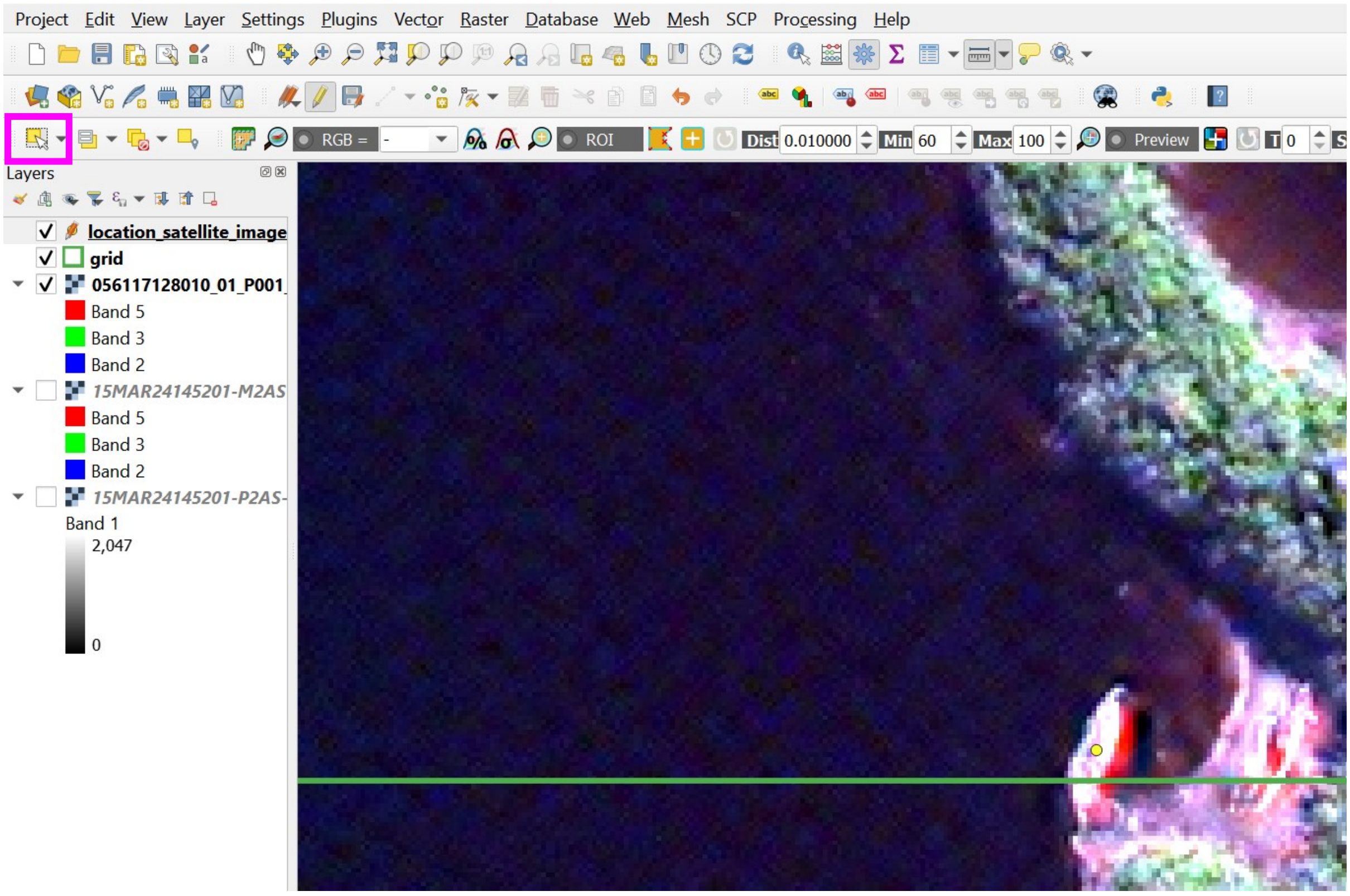

h. click on the feature to edit or delete and use the delete button on your keyboard to delete an annotation. To complete the deletion, select 'Delete 1 Feature(s)'

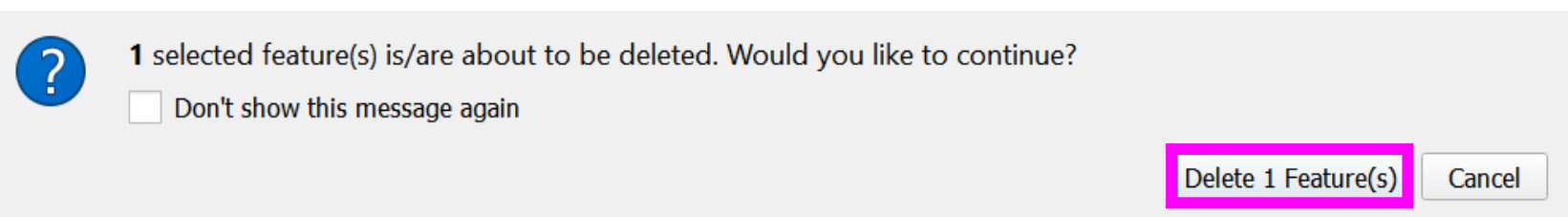

i. to edit or move a point, select the 'Vertex Tool – Current Layer' (line with a vertex and hammer icon) in the 'Toggle Editing' toolbar, double click and drag the point to a new location

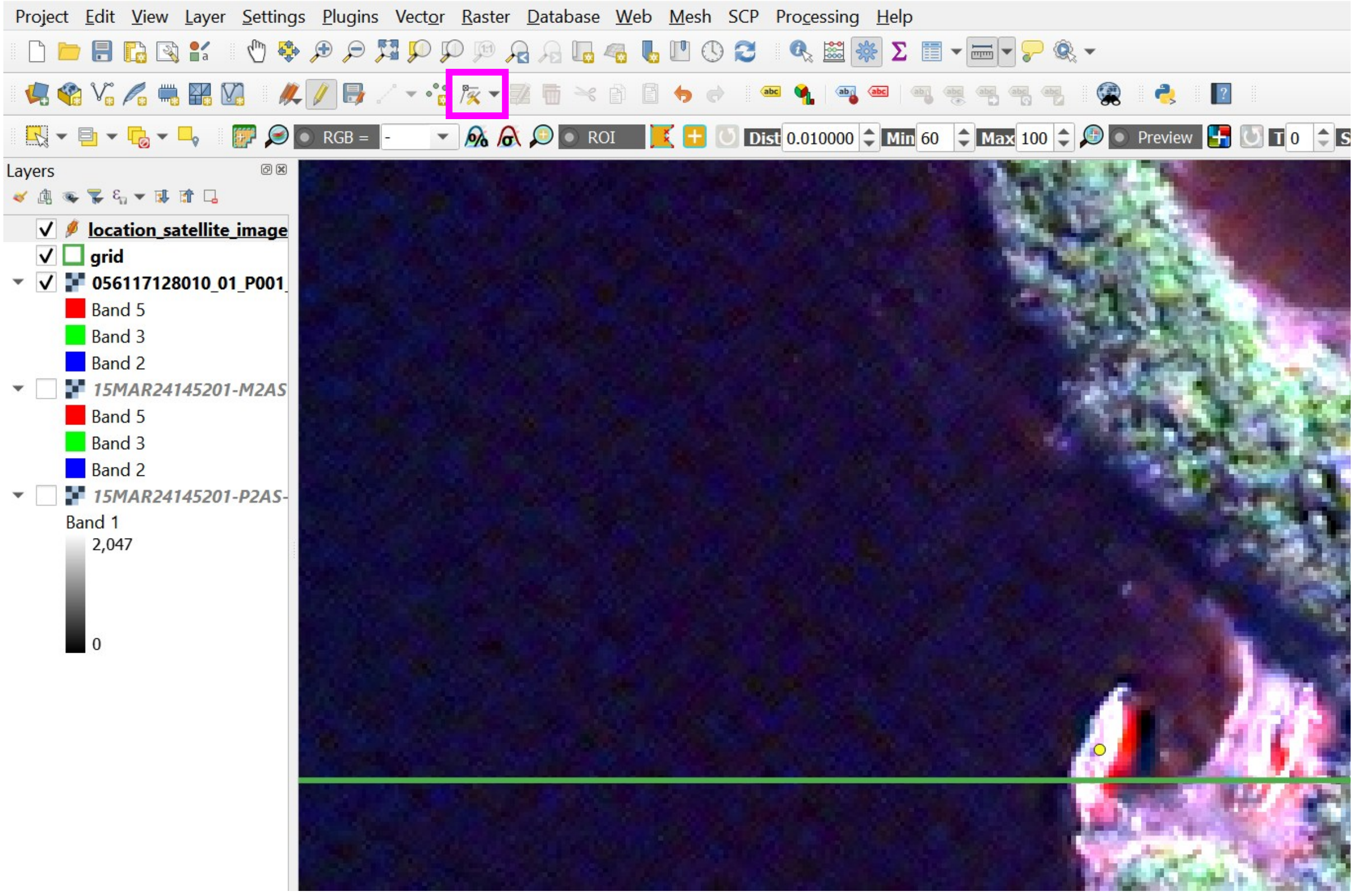

j. ensure to save after any deletion/edits, firstly using the save in the 'Toggle Editing' toolbar, followed by the project save

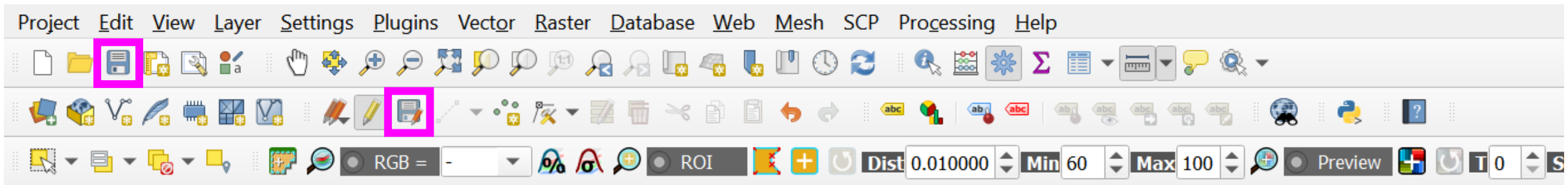

19. Adding XY (Longitude and Latitude) values to annotated features

Skip this step if creating image chips and complete instead when directed to during buffer creation.

When all features are annotated, the latitude and longitude can be extracted. This information is valuable for ground truthing observations and for data sharing. For consistency and ease of comparison the recommended format is decimal degrees. Projected coordinate systems units, with a known zone and datum, are typically linear measurements, for example, metres, and will need to be transformed. Geographic coordinate systems units are typically in decimal degrees and will not require transforming.

Add latitude and longitude values for point features using the field calculator:

a. from the attribute table, select the 'Open field calculator' icon (abacus icon)

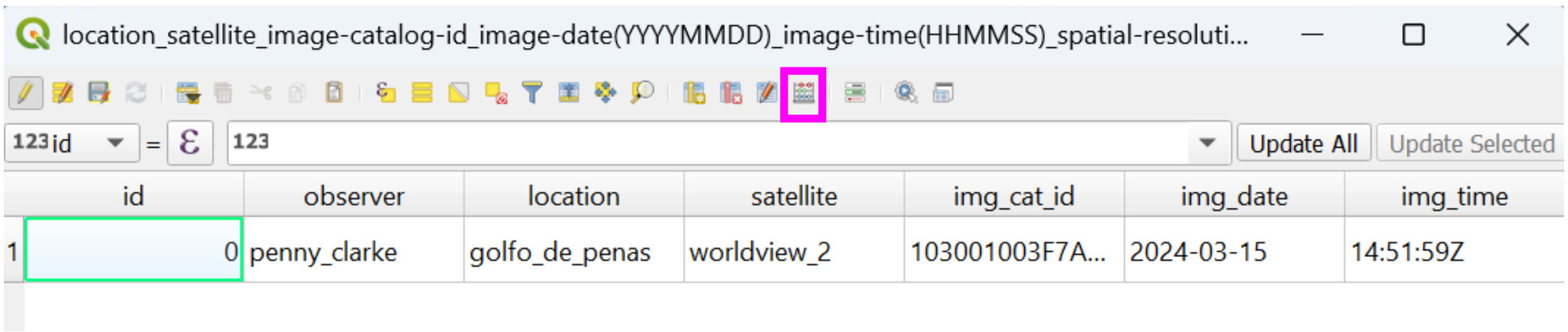

b. check the ‘Create a new field’ option, provide the ‘Output field name’ as ‘longitude’, the ‘Output field type’ as ‘Decimal number (real)’ the ‘Precision’ as 6, and in the ‘Expression’ box: if using a projected coordinate system, type **x(transform($geometry, layer_property(@layer, 'crs'),'EPSG:4326'))**, and if already using a geographic coordinate system type **$x**

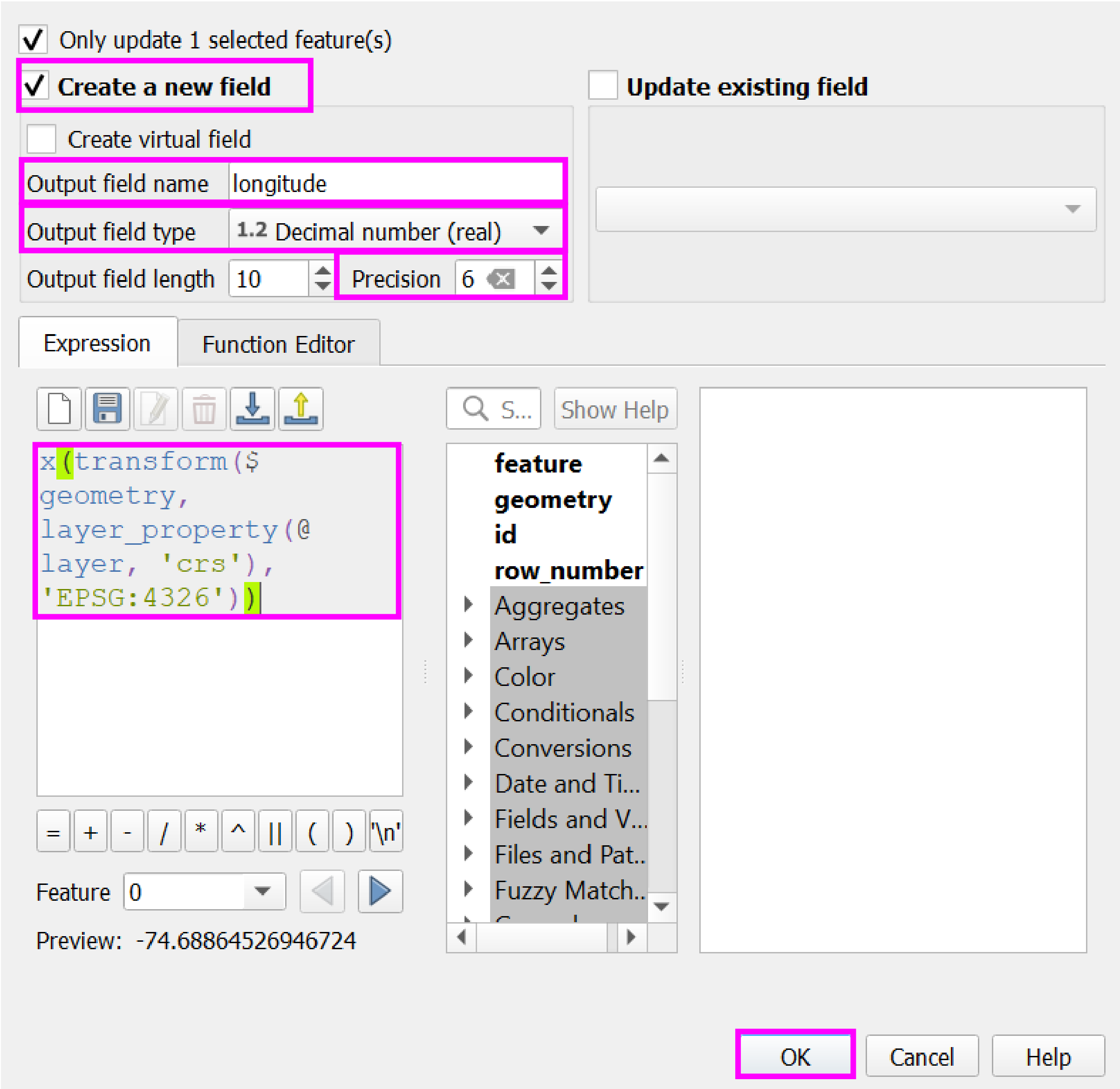

c. Repeat the same process, amending the ‘Output field name’ to ‘latitude’, and in the ‘Expression’ box: if using a projected coordinate system, type **y(transform($geometry,**

layer_property(@layer, 'crs'),'EPSG:4326')), and if already using a geographic coordinate system type **$y**

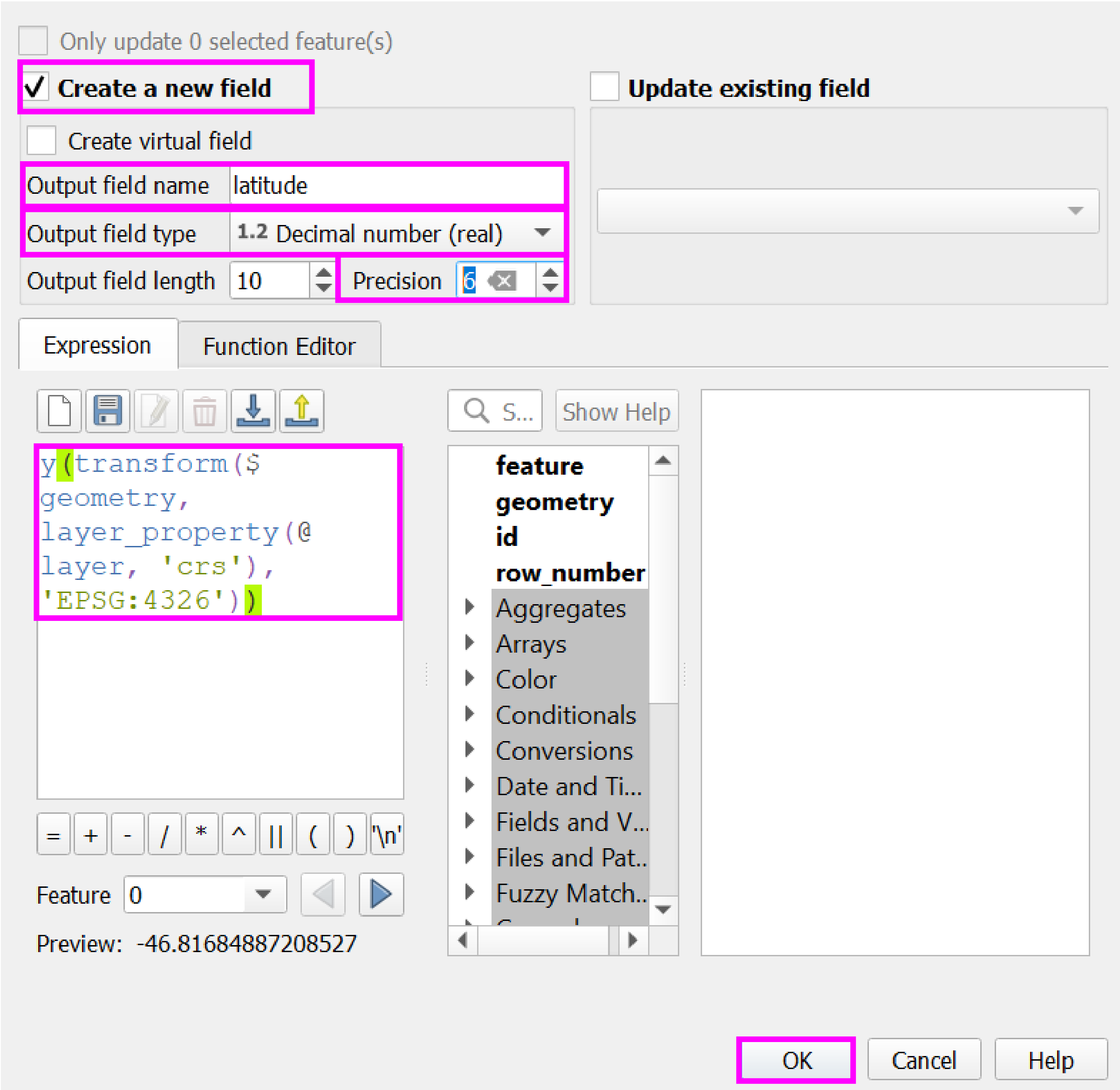

d. the new columns ‘longitude’ and ‘latitude’ will now appear in the attribute table

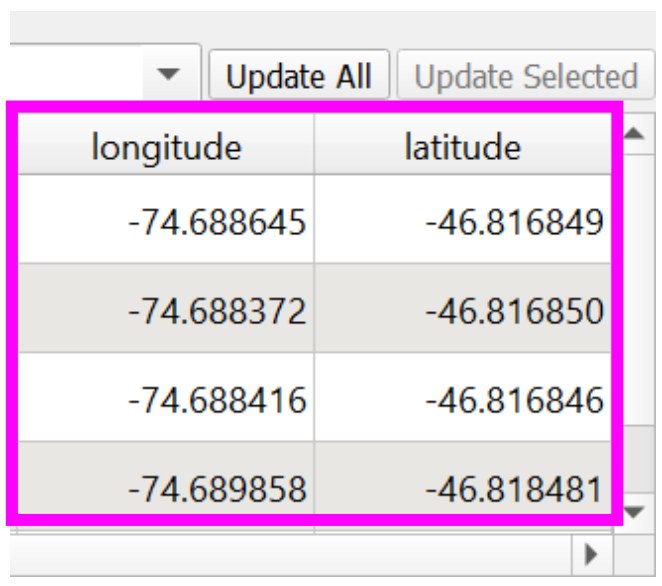

e. Ensure to save the edits and main project once complete

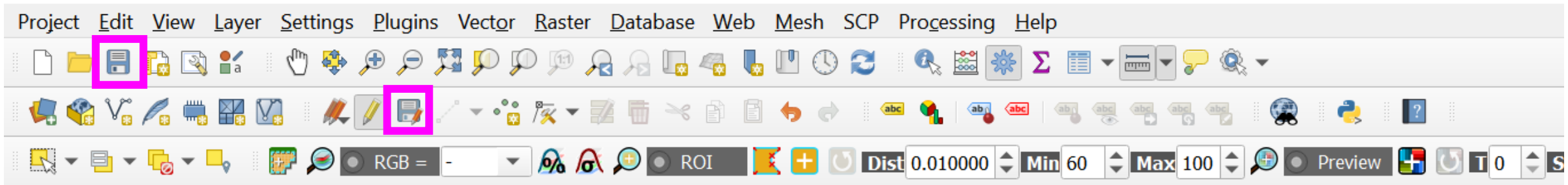

f. Applying something to all cells in a column of the attribute table

While the template shapefile is designed to include dropdown options and replicate fields for each point where possible; you may wish to amend a value for a whole column quickly.

i. in the attribute table, enter 'Toggle editing mode' (yellow pencil icon), using the sum toolbar below the main attribute table toolbar, from the dropdown, select the column to amend, and in the cell next to the Ɛ icon enter the new value (in 'quote marks' for string/text), and select 'Update All'.

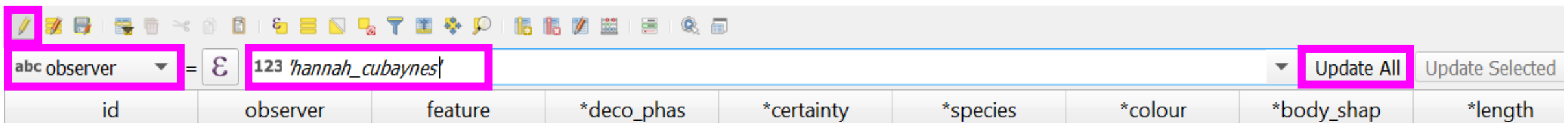

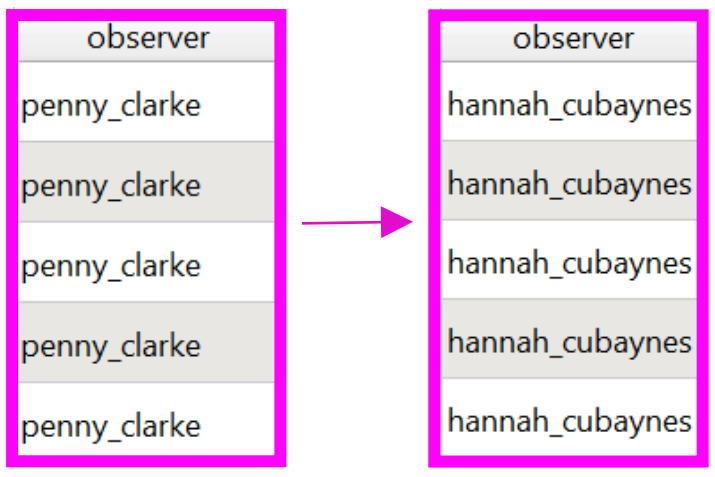

g. extracting an attribute table:

i. to work with data in a coding environment, you may wish to export your attribute table as a .csv format. Right click the shapefile in the 'Layers' pane > 'Export' > 'Save Feature As…' and set the format to 'Comma Separated Value [CVS]'

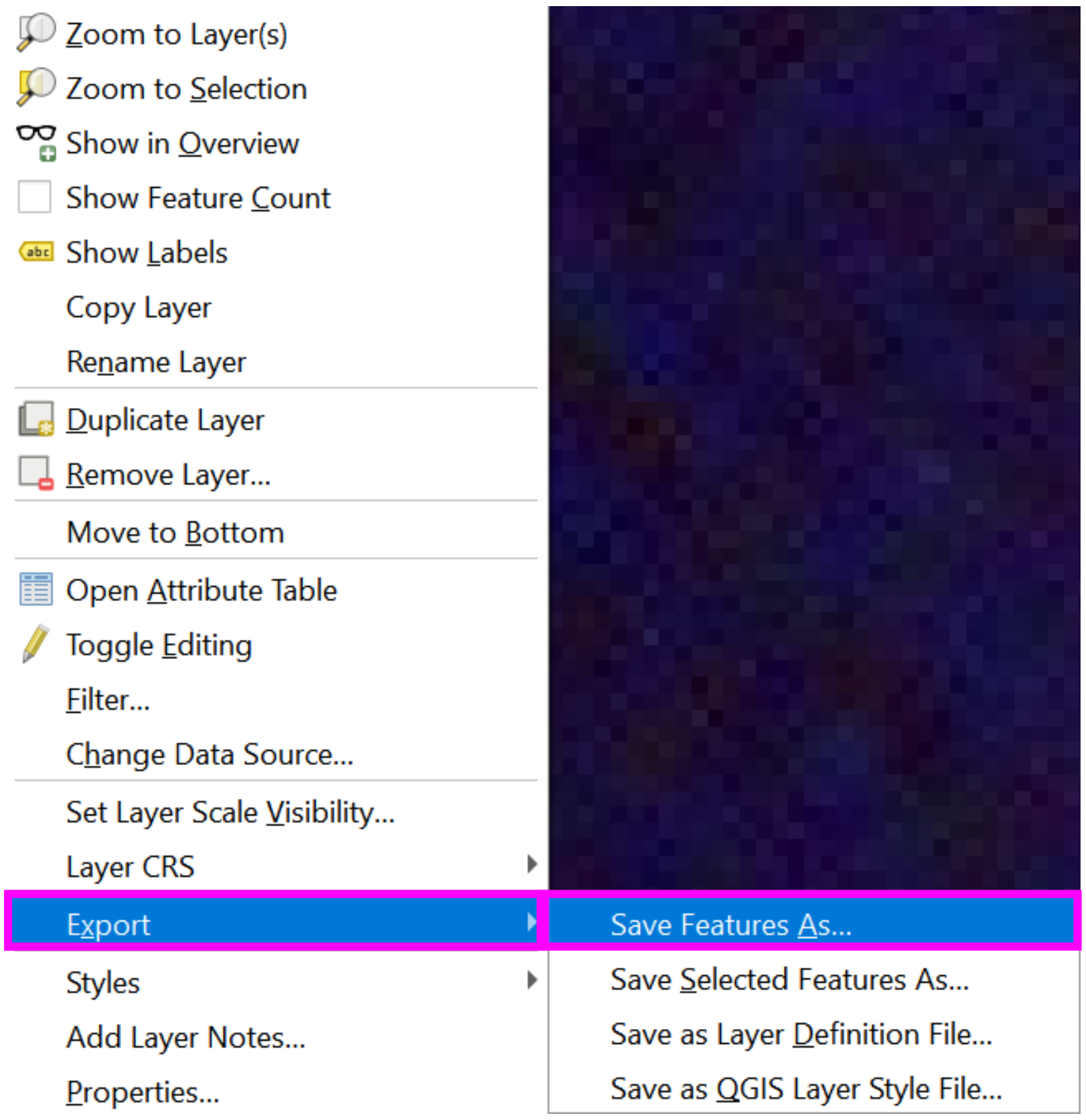

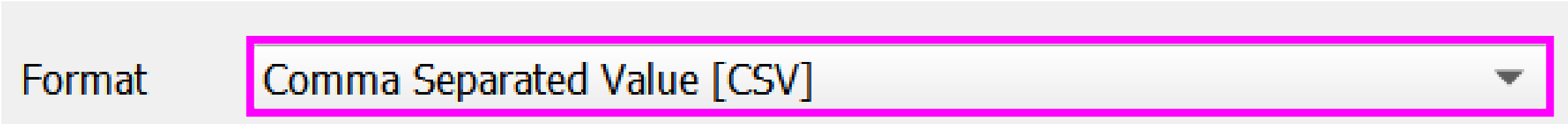

CREATING BOUNDING BOXES

20. Centralising point to pixel using 'Rasterization'
    a. In the 'Processing Toolbox' select 'OTB' > 'Vector Data Manipulation' > Rasterization

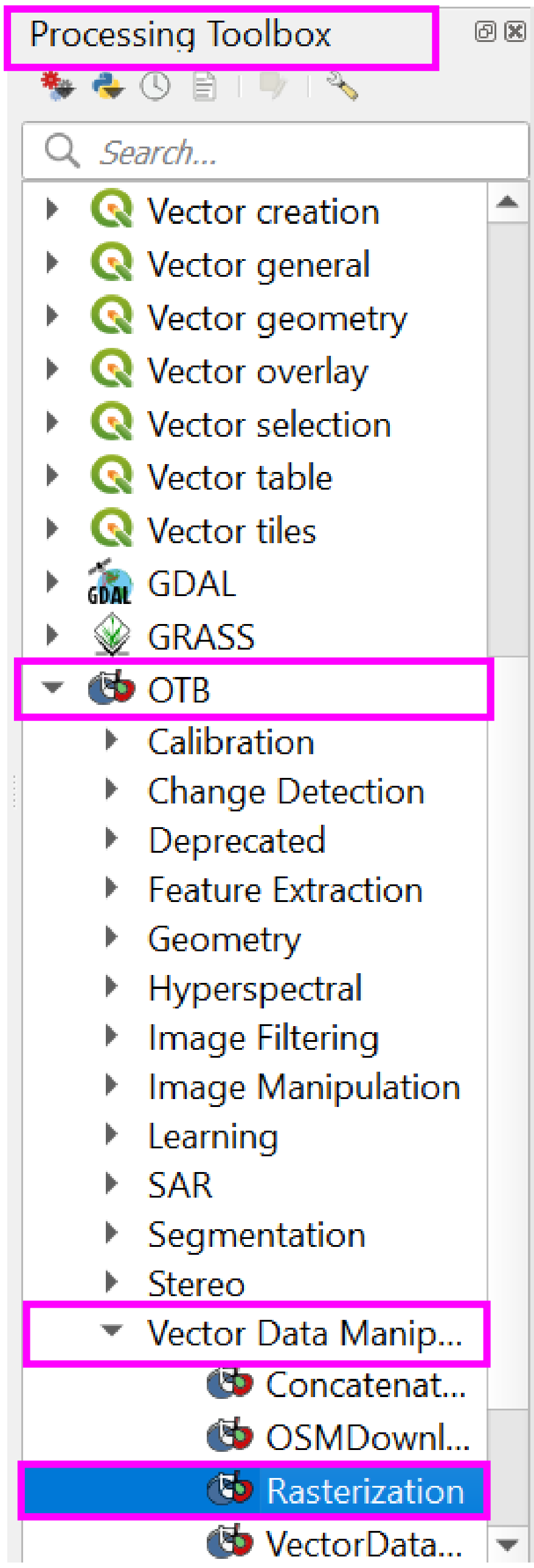

    b. set the 'Input vector dataset' as the template/point shapefile and the 'Input reference image' as the pansharpened image. Set the 'Output EPSG code' as your project crs if not automatically detected. Set the 'Output image' to save to a suitable directory and assign a file name, for example 'point_to_pixel' and 'Run'

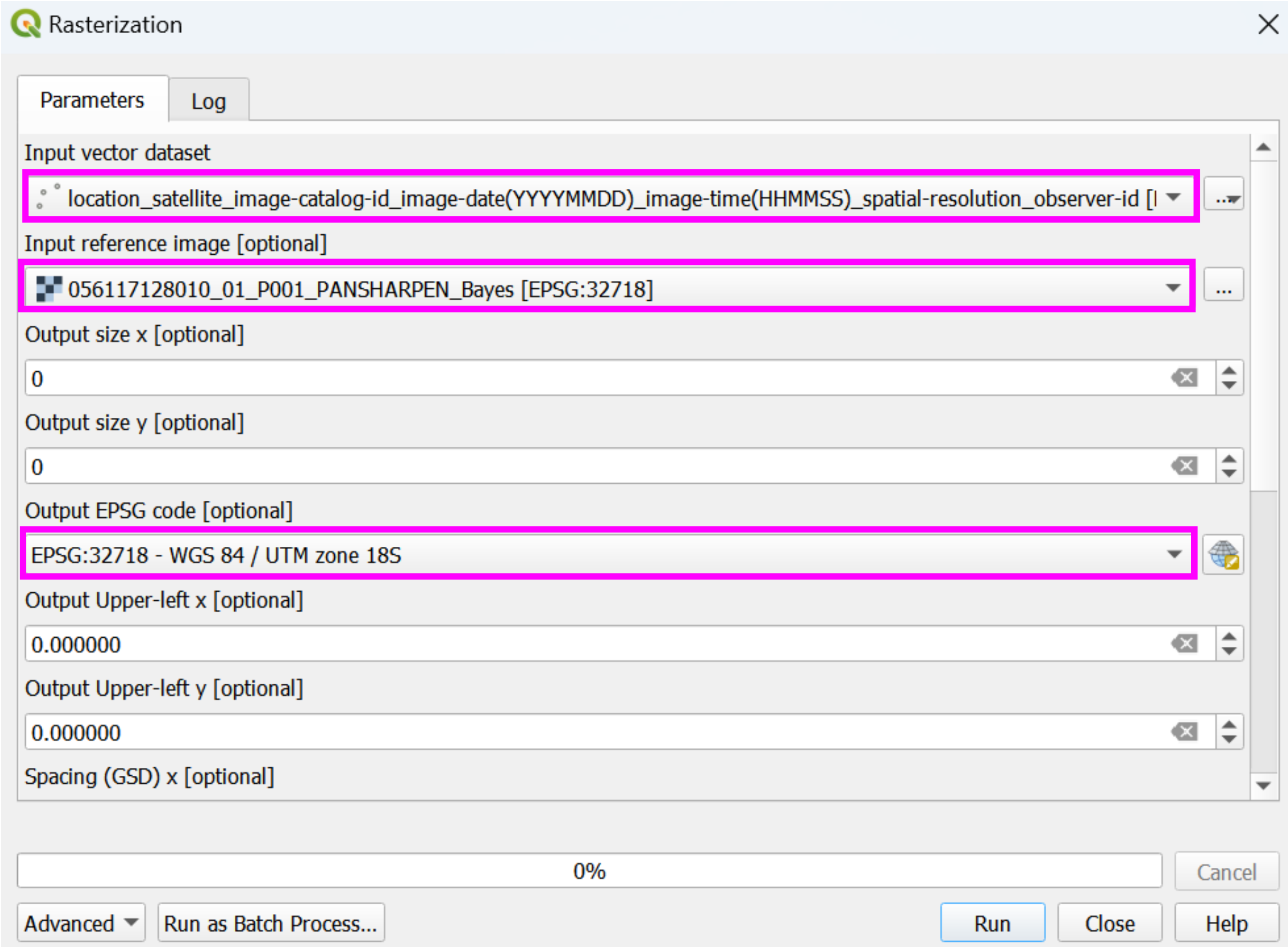
Rasterization
Parameters
Log
Input vector dataset
location_satellite_image-catalog-id_image-date(YYYYMMDD)_image-time(HHMMSS)_spatial-resolution_observer-id [I
Input reference image [optional]
056117128010_01_P001_PANSHARPEN_Bayes [EPSG:32718]
Output size x [optional]
0
Output size y [optional]
0
Output EPSG code [optional]
EPSG:32718 - WGS 84 / UTM zone 18S
Output Upper-left x [optional]
0.000000
Output Upper-left y [optional]
0.000000
Spacing (GSD) x [optional]
0%
Cancel
Advanced
Run as Batch Process...
Run
Close
Help

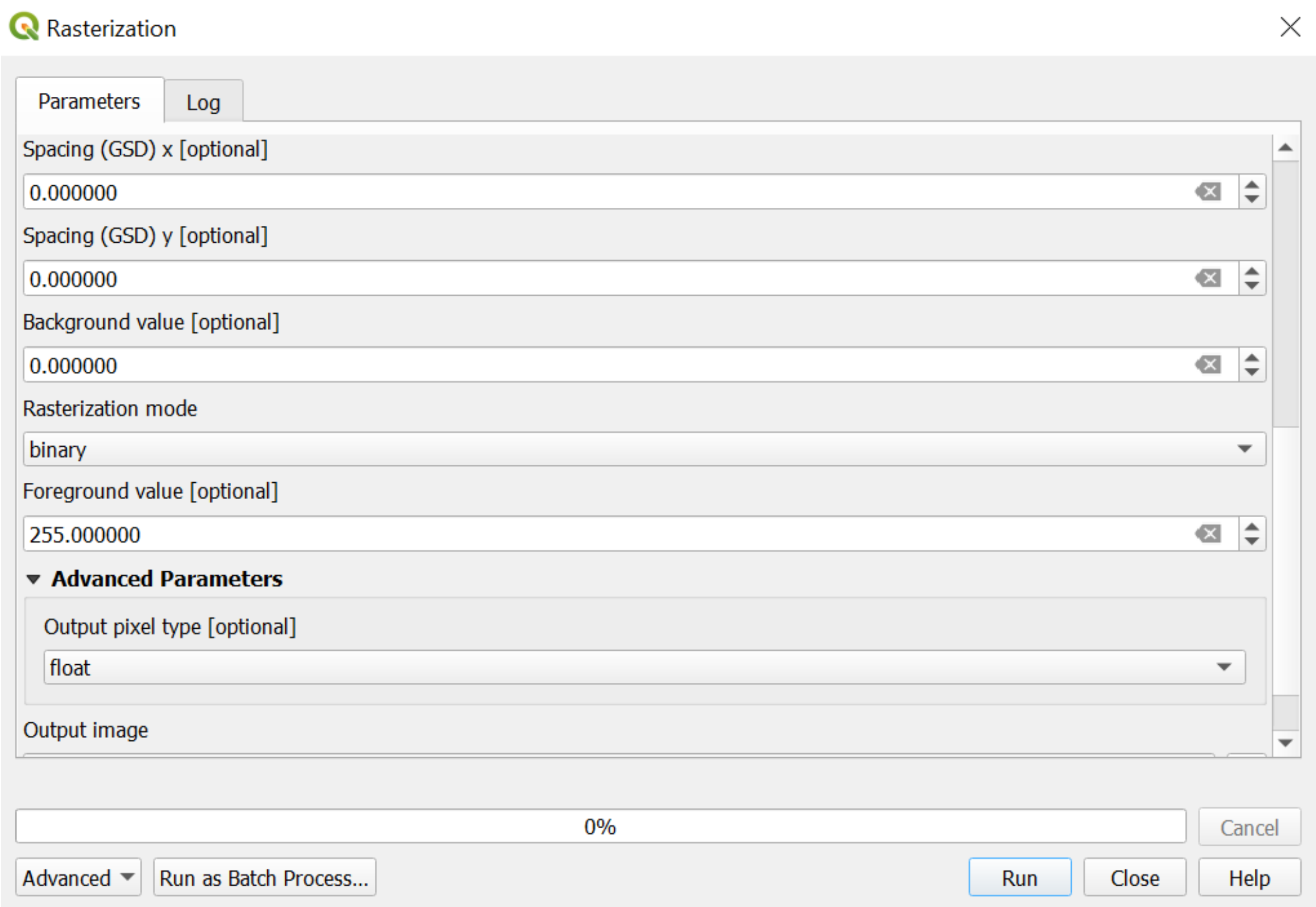
Rasterization
Parameters
Log
Spacing (GSD) x [optional]
0.000000
Spacing (GSD) y [optional]
0.000000
Background value [optional]
0.000000
Rasterization mode
binary
Foreground value [optional]
255.000000
Advanced Parameters
Output pixel type [optional]
float
Output image
0%
Cancel
Advanced
Run as Batch Process...
Run
Close
Help

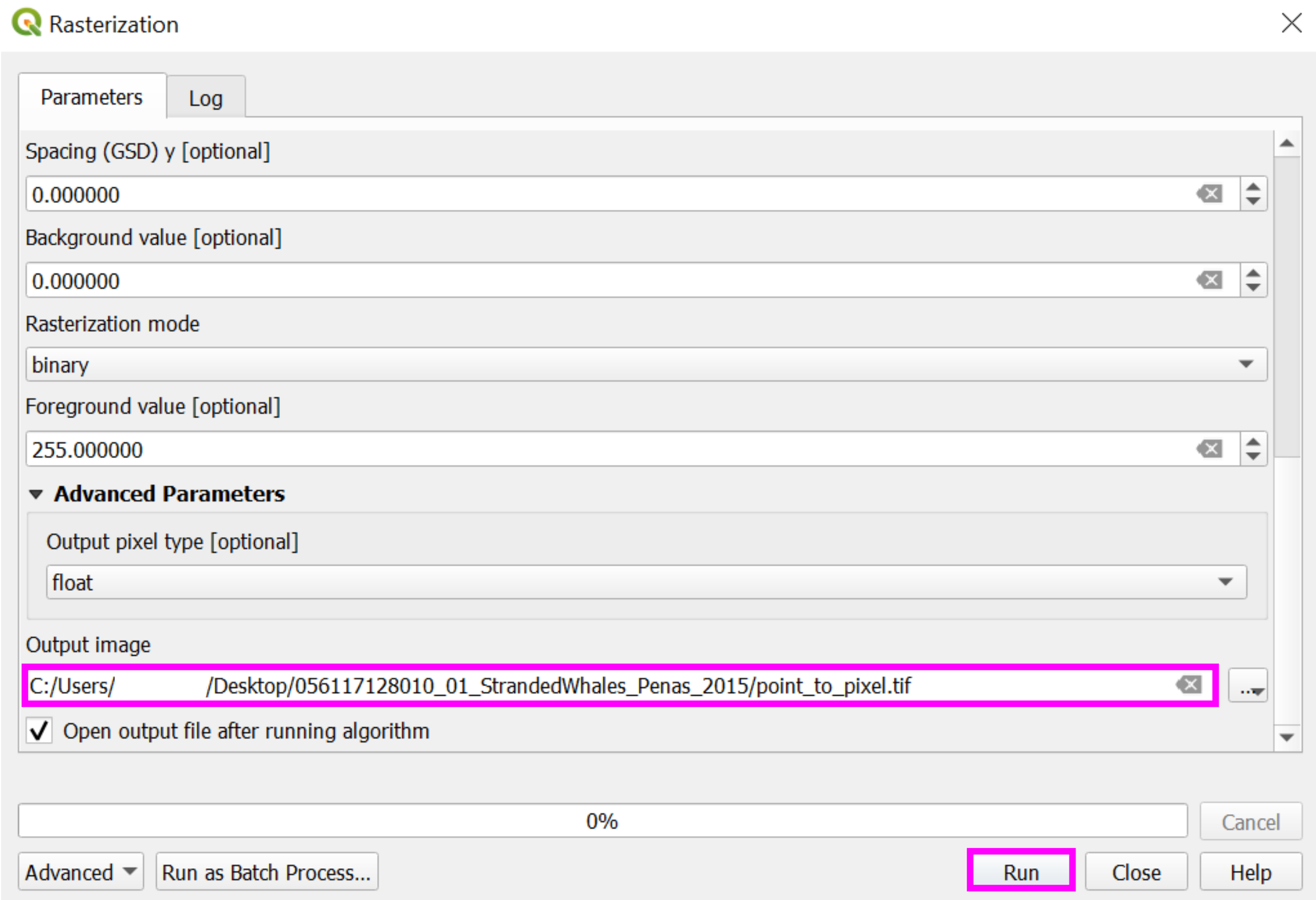

c. the 'point_to_pixel' layer will appear in the 'Layers' pane and a single band grey scale pixel will appear atop of each annotated point

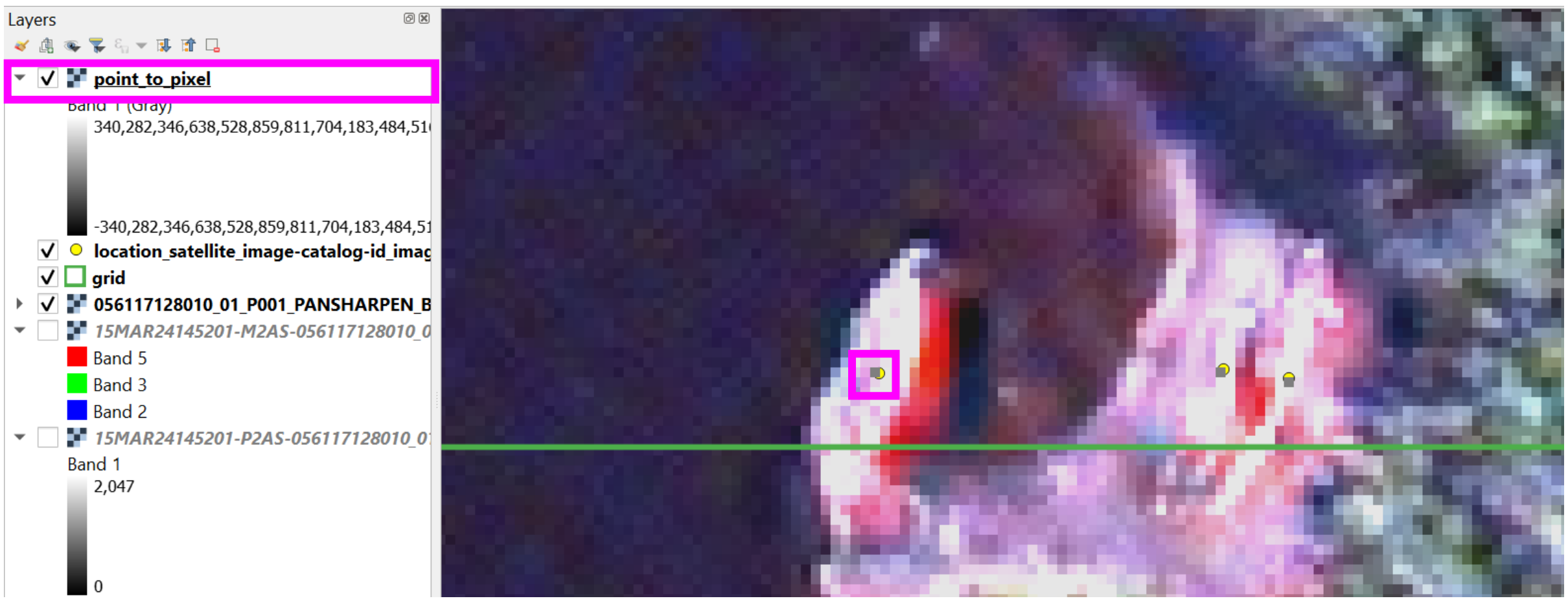

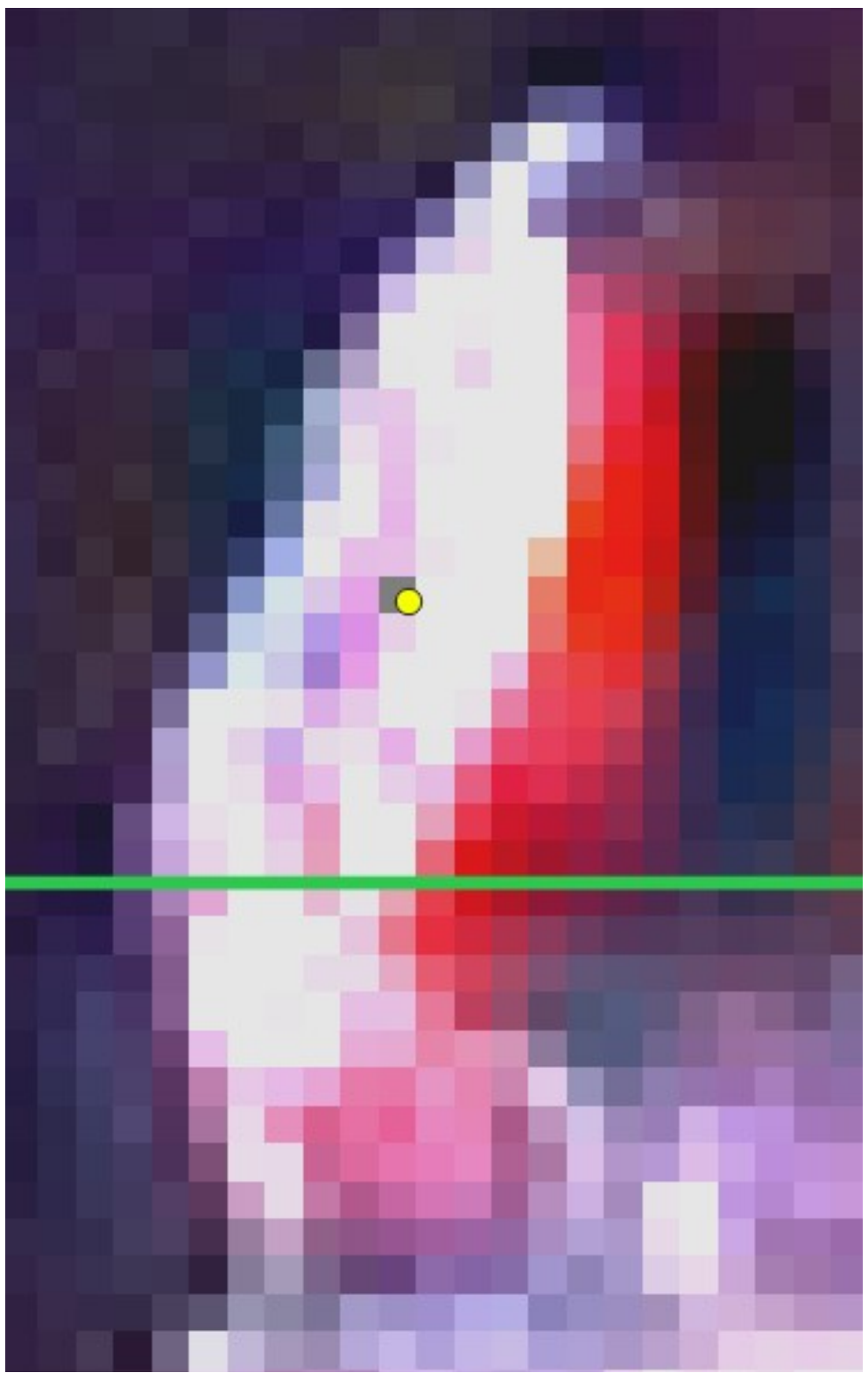

21. Creating centralised points using ‘Raster pixels to points’
    a. In the ‘Processing Toolbox’ select ‘Vector creation’ > ‘Raster pixels to points’

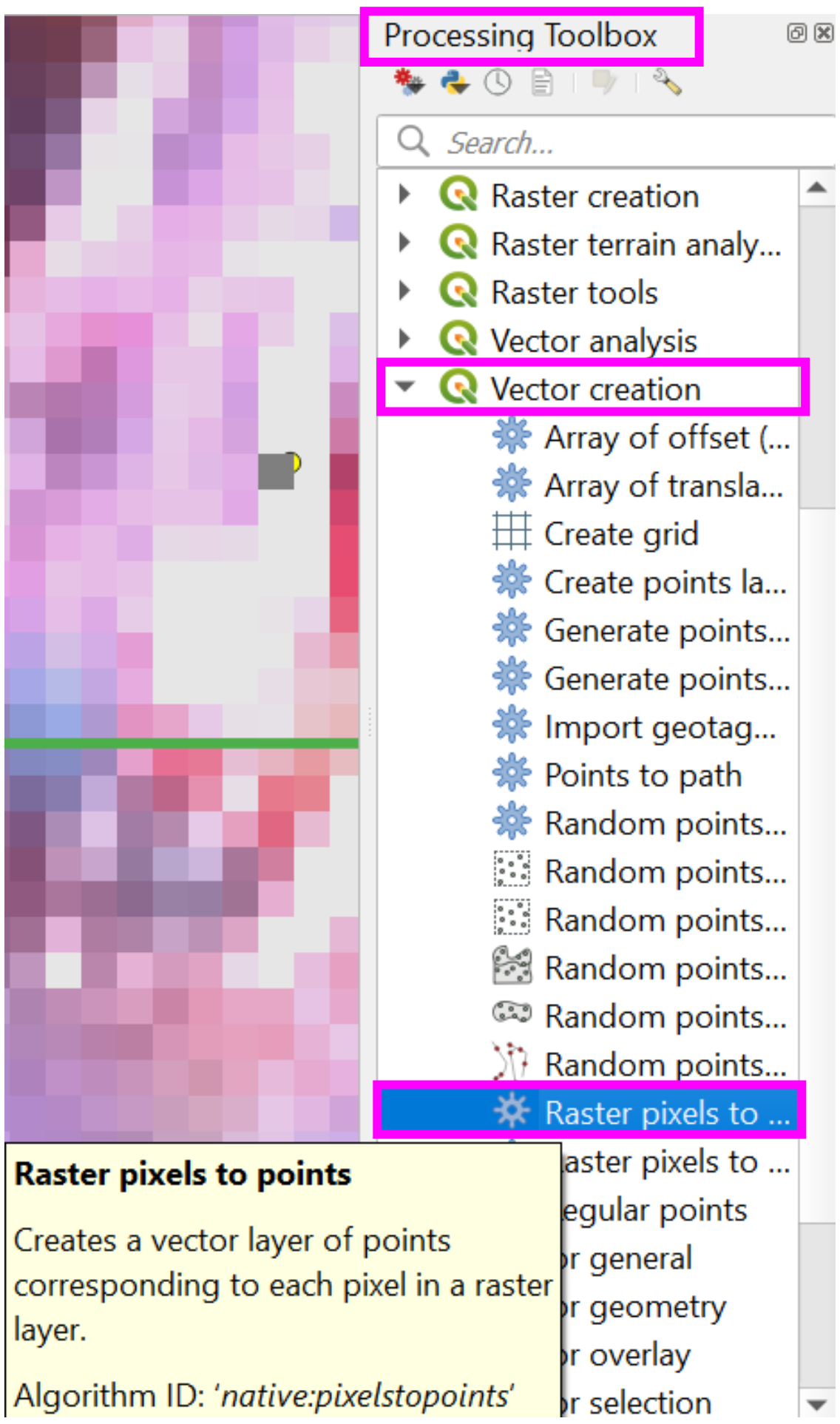

b. set the 'Raster layer' as the point_to_pixel created in section '20', 'Band number' as 'Band 1 (Gray), 'Field name' as 'VALUE'. For 'Vector point' provide a suitable file path and name to save, for example 'centred_point' and 'Run'

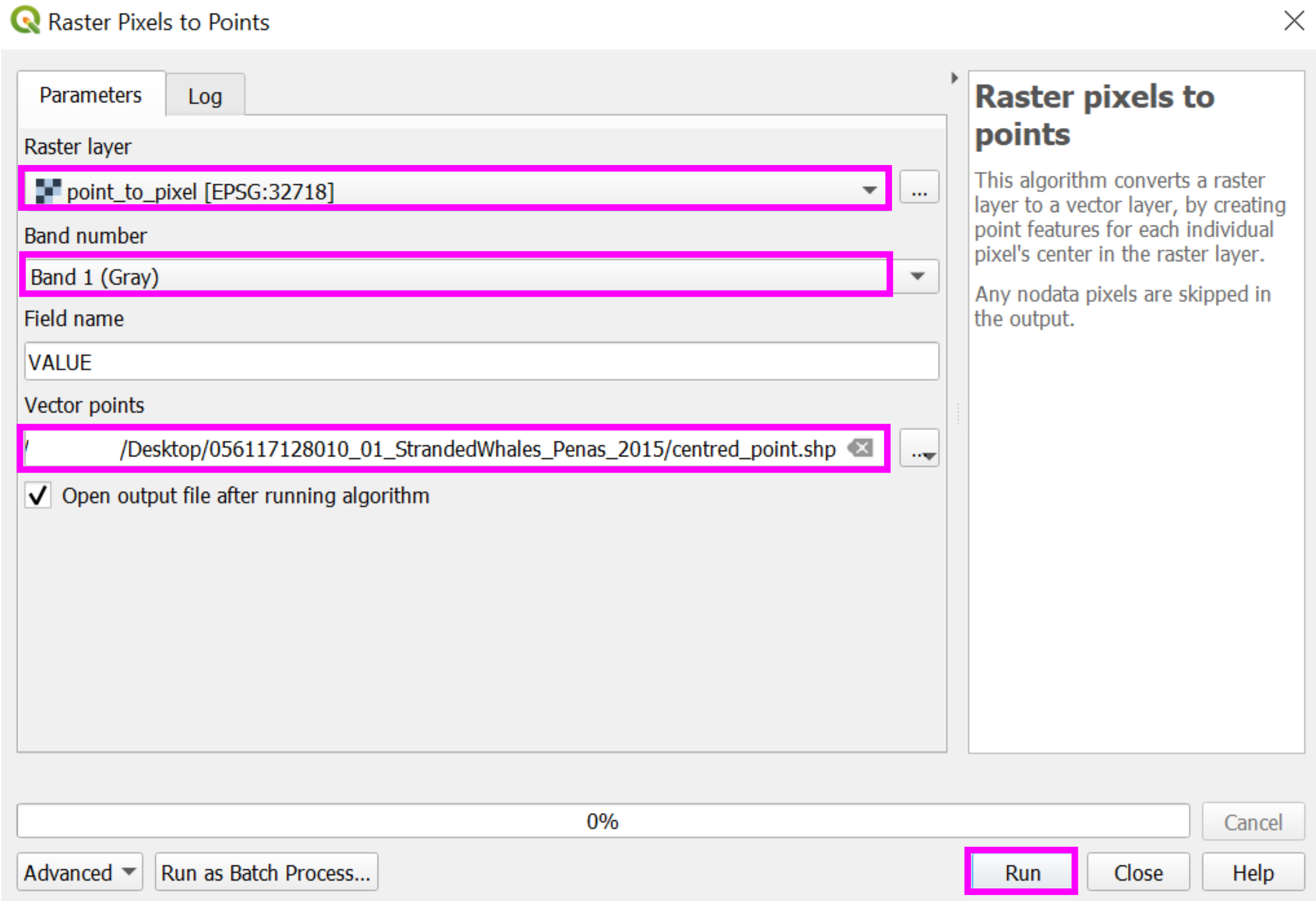

c. 'centred_point' layer will appear in the 'Layers' pane and a new point shapefile will appear in the main project pane atop of each original annotated point and central to the underlying pixel

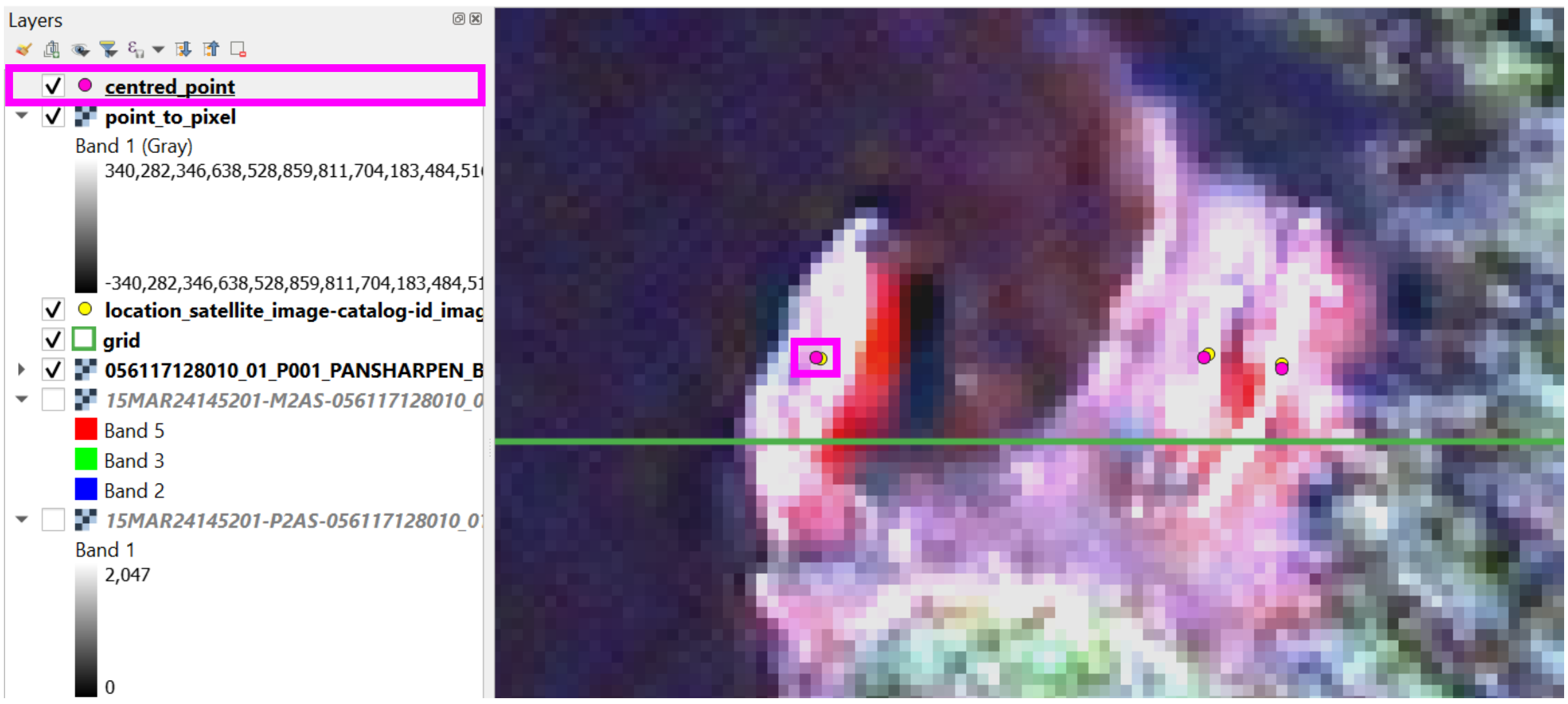

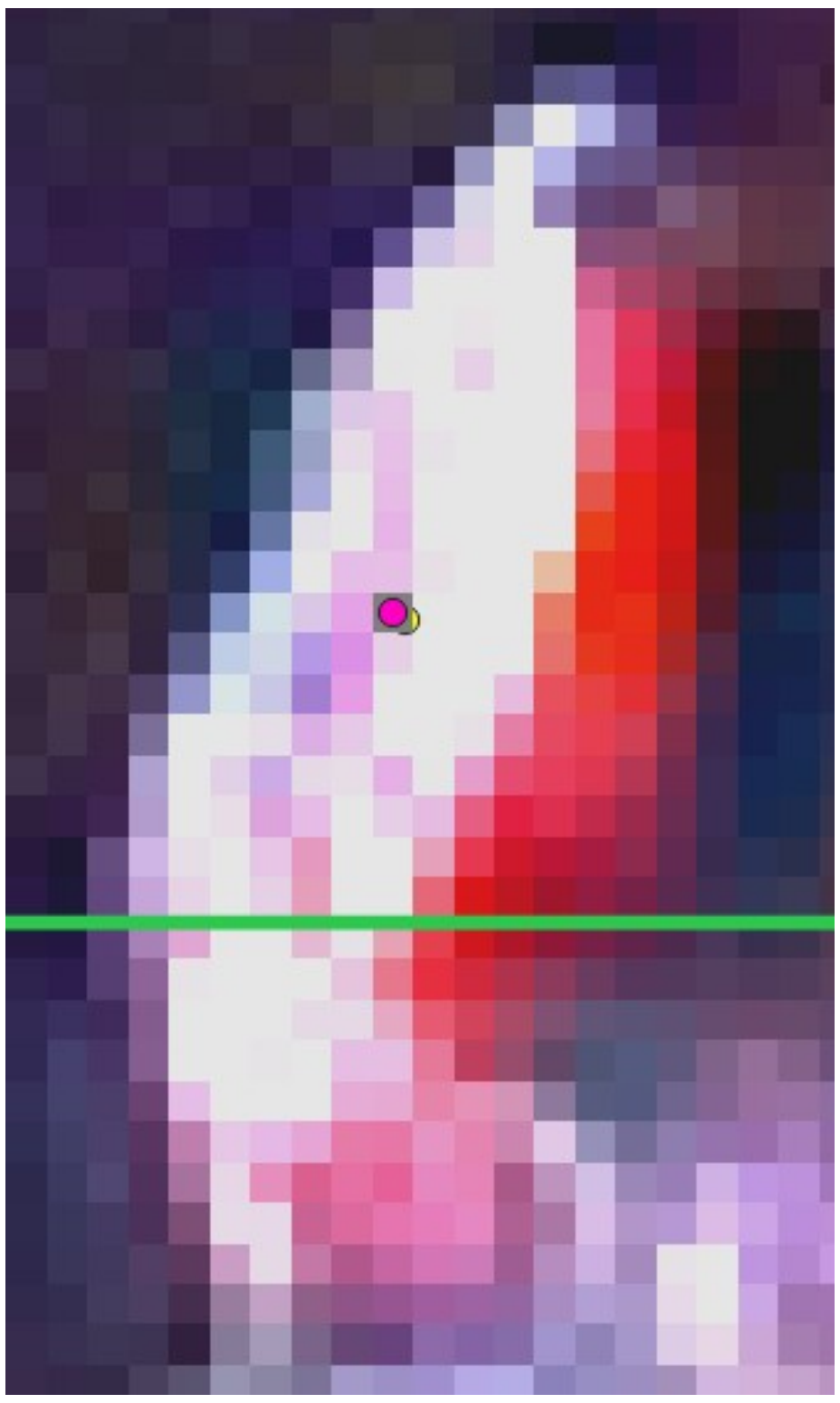

22. Adding attribute table to centred point shapefile using ‘Join attributes by nearest’
    a. in the ‘Processing Toolbox’ select ‘Vector general’ > ‘Join attributes by nearest’

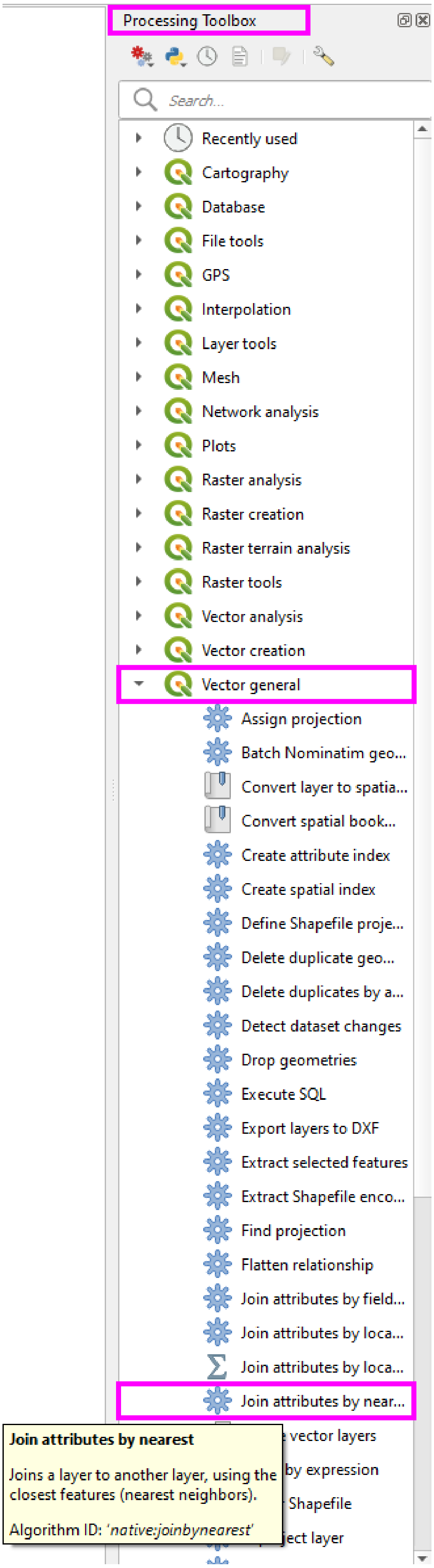
Processing Toolbox
Search...
Recently used
Cartography
Database
File tools
GPS
Interpolation
Layer tools
Mesh
Network analysis
Plots
Raster analysis
Raster creation
Raster terrain analysis
Raster tools
Vector analysis
Vector creation
Vector general
Assign projection
Batch Nominatim geo...
Convert layer to spatia...
Convert spatial book...
Create attribute index
Create spatial index
Define Shapefile proje...
Delete duplicate geo...
Delete duplicates by a...
Detect dataset changes
Drop geometries
Execute SQL
Export layers to DXF
Extract selected features
Extract Shapefile enco...
Find projection
Flatten relationship
Join attributes by field...
Join attributes by loca...
Join attributes by loca...
Join attributes by near...
Join attributes by nearest
Joins a layer to another layer, using the closest features (nearest neighbors).
Algorithm ID: 'native:joinbynearest'

b. set the 'Input layer' as 'centred_points' created in section '21', the 'Input layer 2' as the original shapefile layer containing the completed attribute table. Set the 'Maximum nearest neighbors' to '1' and the 'Maximum distance' to your pixel size. In this example, 0.5m

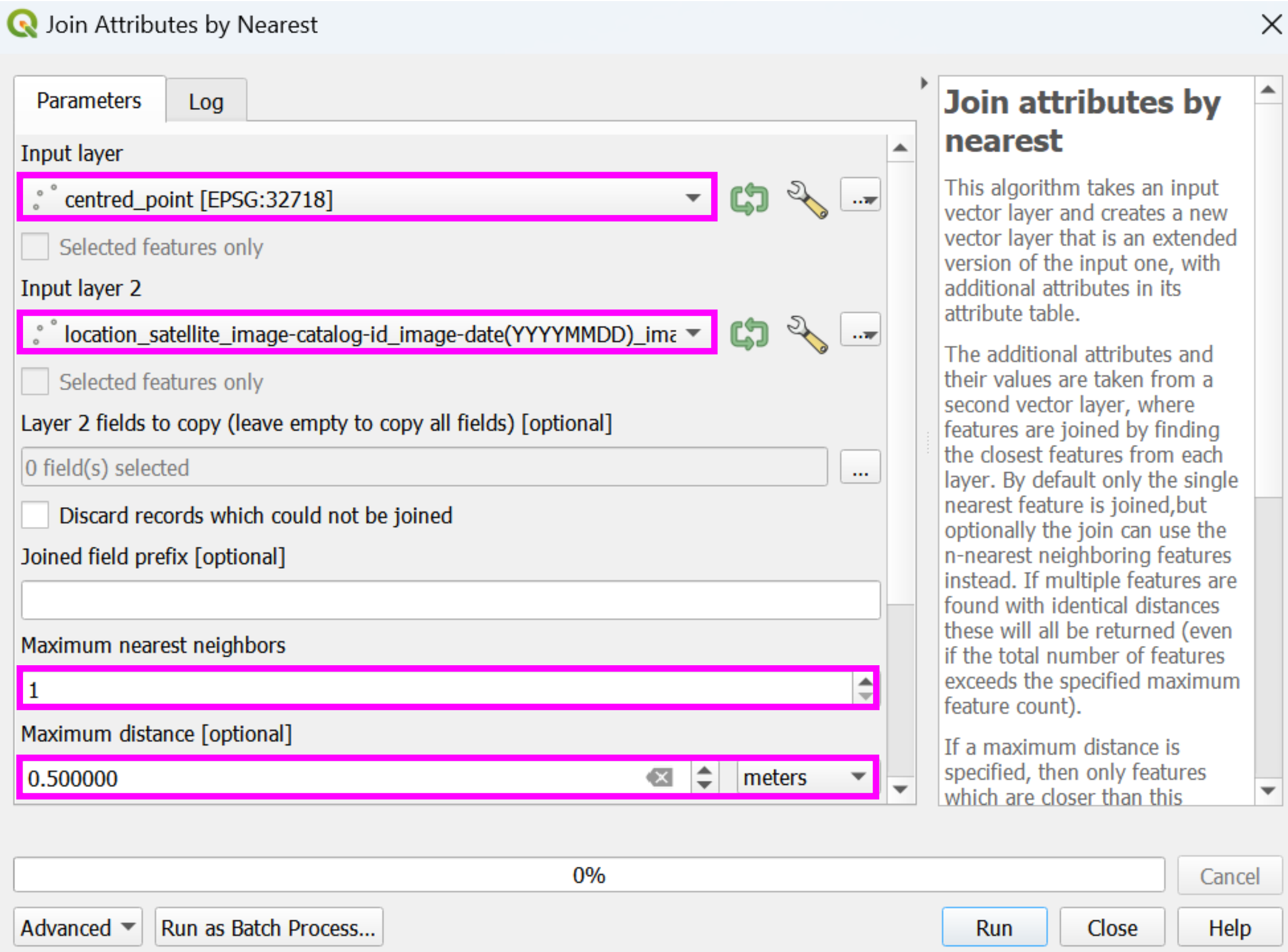

c. as this is the final version of the shapefile/attribute table, in 'Joined layer' define a suitable location and rename the file as your project naming convention, in this example, 'golfo-de-penas-chile_wv2_103001003F7A9900_20240315_145159_50_1'. Select 'Run'

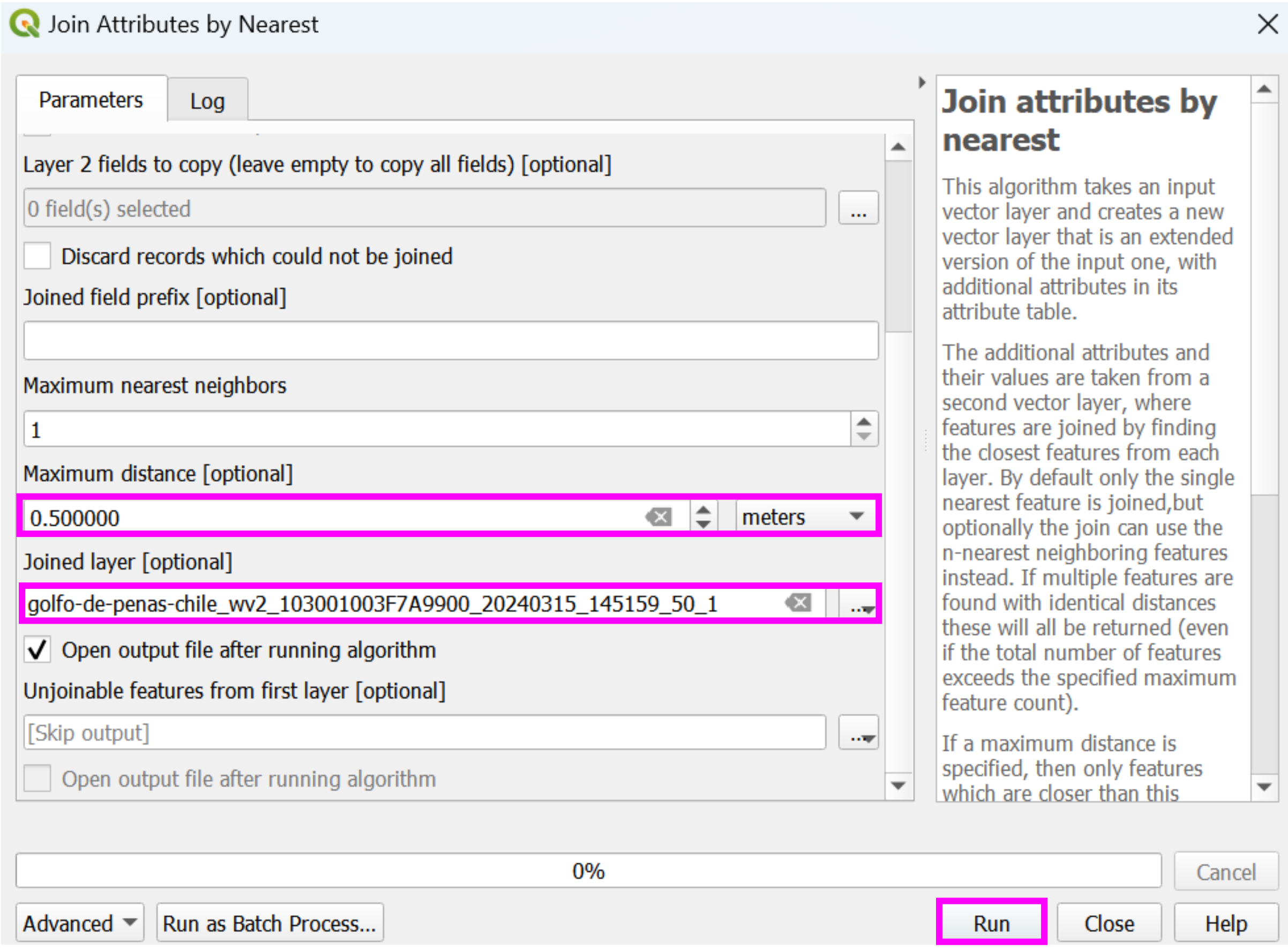

d. the newly created shapefile with project name will appear in the 'Layers' pane, and when right clicking the layer and 'Open Attribute Table', the table will now be populated with all the annotations of the original shapefile

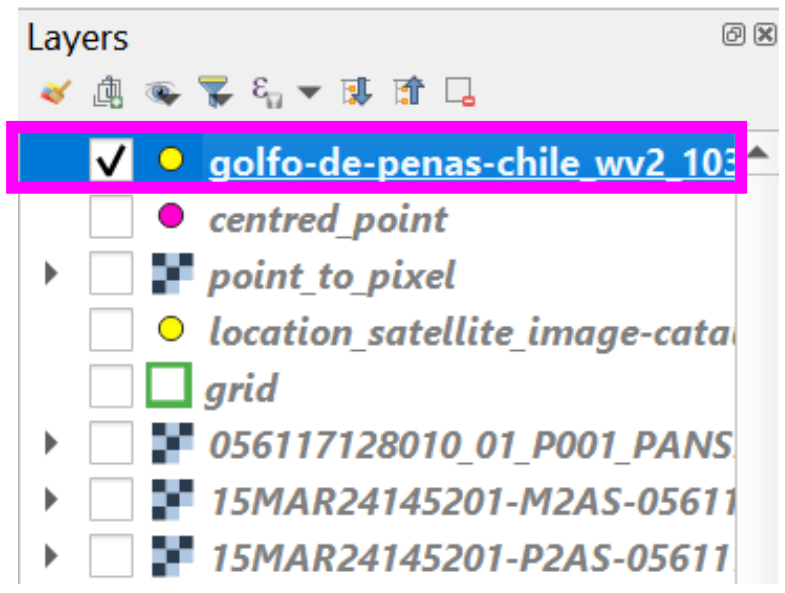

e. the shapefile by default will have 7 new columns, 'Value', 'n', 'distance', 'feature_x', 'feature_y', 'nearest_x' and 'nearest_y'. These columns are not required and can be removed. Select 'Toggle editing mode' icon (yellow pencil) followed by 'Delete field' icon (table with red pencil). In the 'Delete Fields' window that opens, highlight columns not required, by pressing the 'ctrl' key while clicking on column names, and select 'OK'

VALUE	n	distance	feature_x	feature_y	nearest_x	nearest_y
255.00000000	1	0.19650023878...	523751.500000...	4815141.50000...	523751.682015...	4815141.42595...
255.00000000	1	0.18355040226...	523772.500000...	4815141.00000...	523772.500441...	4815141.18354...
255.00000000	1	0.22801995437...	523769.000000...	4815141.50000...	523769.180907...	4815141.63880...
255.00000000	1	0.07285656149...	523658.500000...	4814960.50000...	523658.479189...	4814960.43017...
255.00000000	1	0.24882872941...	523474.500000...	4815317.50000...	523474.489746...	4815317.74861...

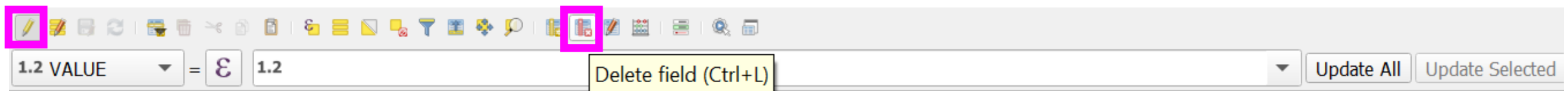

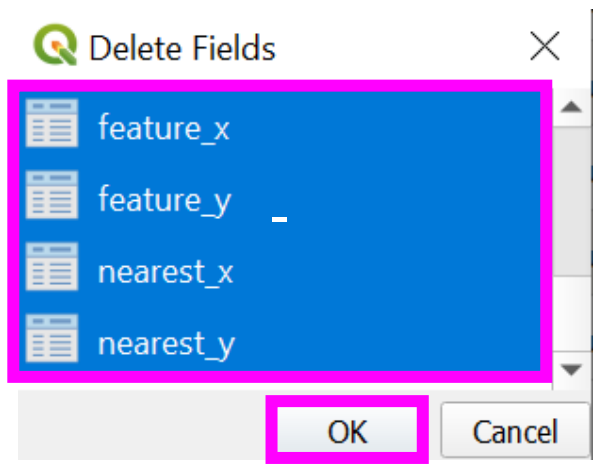

f. the table will now contain all the required data, however, 'Value Map' dropdown options will be absent. These can be restored using the 'Load Style' option discussed in section 'Importing template point shapefile'

g. add XY data to the shapefile using guidance in section 'Adding XY (Longitude and Latitude) values to annotated features'

23. Creating a buffer around centred point

   a. in the 'Processing Toolbox', select 'Vector geometry' > 'Buffer'

   b. select the point shapefile as the 'Input layer' (selecting the green arrow next to the vector input iterates through every point in the shapefile. However, this creates an output for every point separately, if this is left unchecked then the output is a buffer for ever point stored under one shapefile, which is most appropriate and manageable). Set the 'Distance' of the buffer to half of the vertical distance you would like for the whole box, see section 'Step 5: Creating bounding boxes' in the associated manuscript for guidance on setting distance and Table S1.2, for example distances for a number of different desired output chip sizes. Set the 'End cap style' to square to create square buffers, chose a suitable name and location to save the buffer and 'Run'

Table S1.2: Recommended bounding box distances in metres (m) for the main commercially available VHR spatial resolution imagery, based on the desired image chip output size in pixels.

**Pixel size (metres)**	**Desired output chip size (no. of pixels)**	**Bounding Box Distance (m)**
0.50	256 x 256	128
	128 x 128	32
	64 x 64	16
	32 x 32	8
0.30	256 x 256	38.4
	128 x 128	19.2
	64 x 64	9.6
	32 x 32	4.8
0.15	256 x 256	19.2
	128 x 128	9.6
	64 x 64	4.8
	32 x 32	2.4

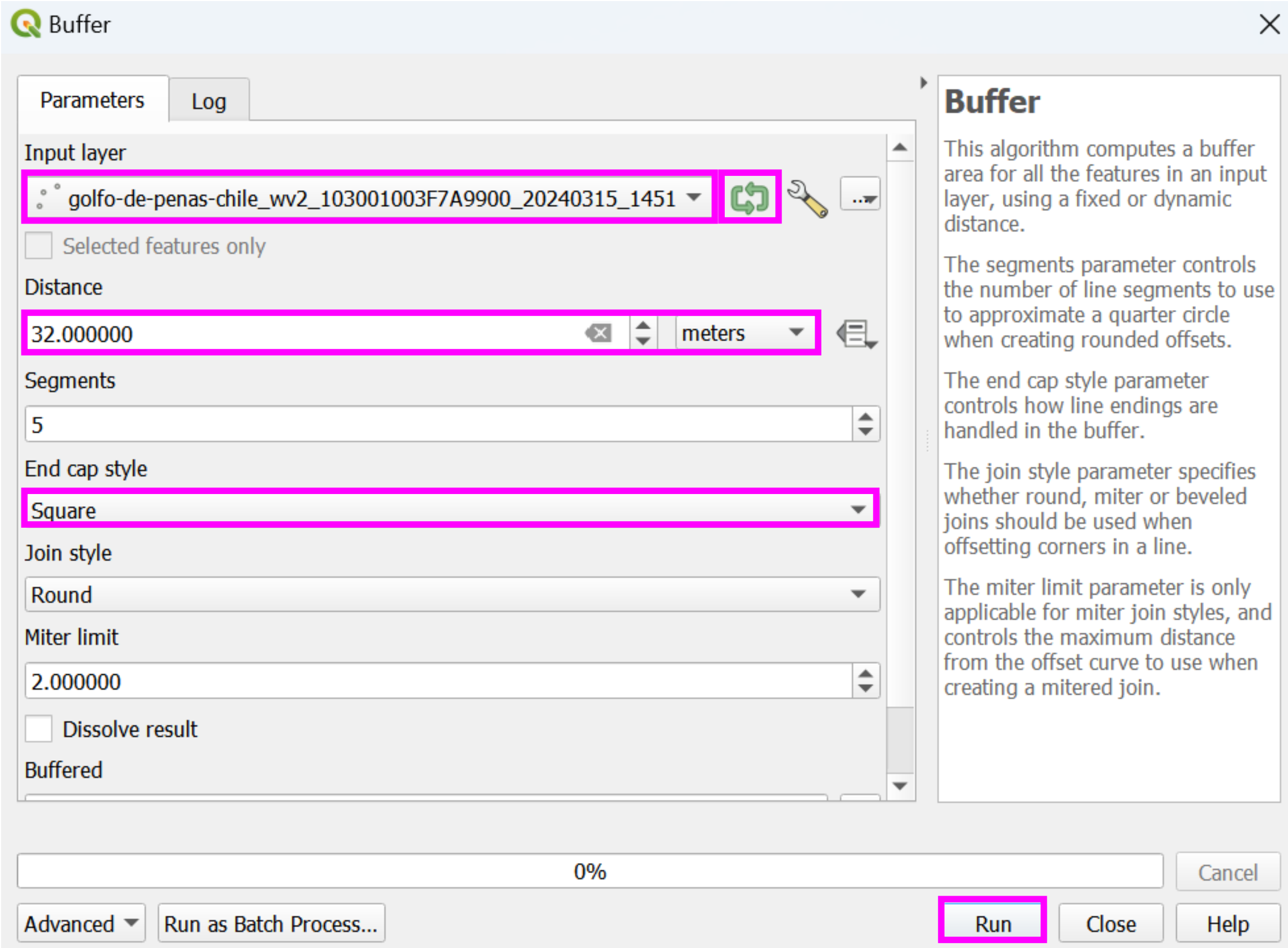

c. the layer will appear in the 'Layers' pane and the box outputs will appear atop of your image. The boxes will appear as hollow fill, right click the buffer layer in the 'Layers' pane > select 'Properties' > 'Symbology'. Choose 'outline green' from the favourites and amend to an appropriate colour, select 'Apply' and 'OK'

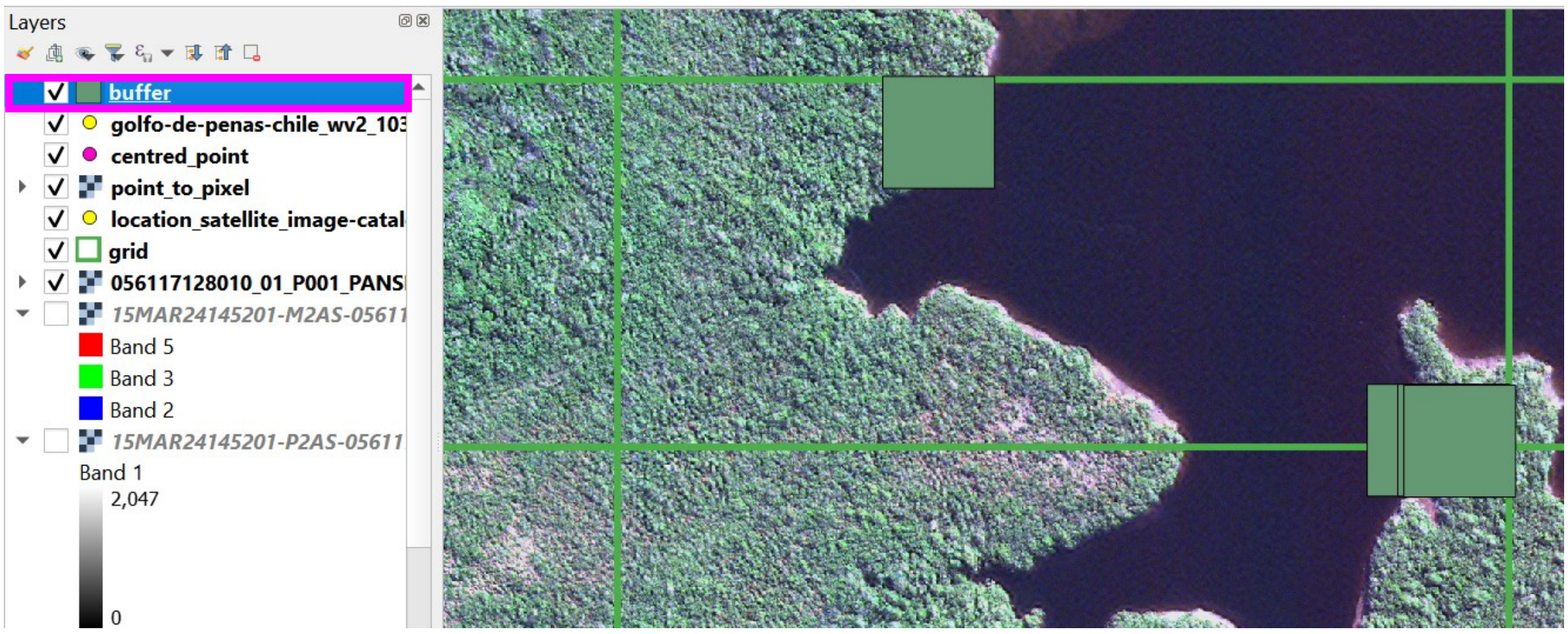

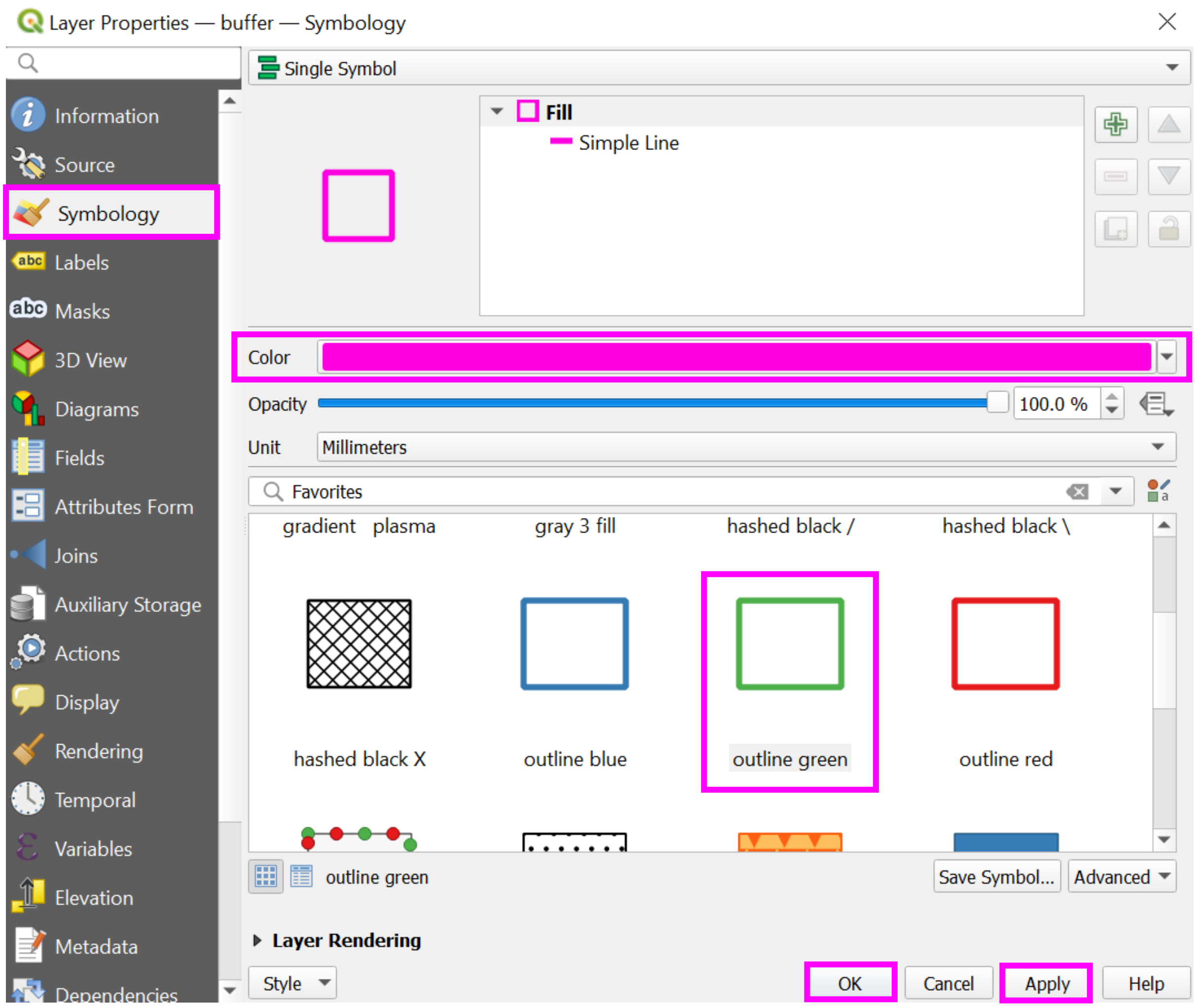

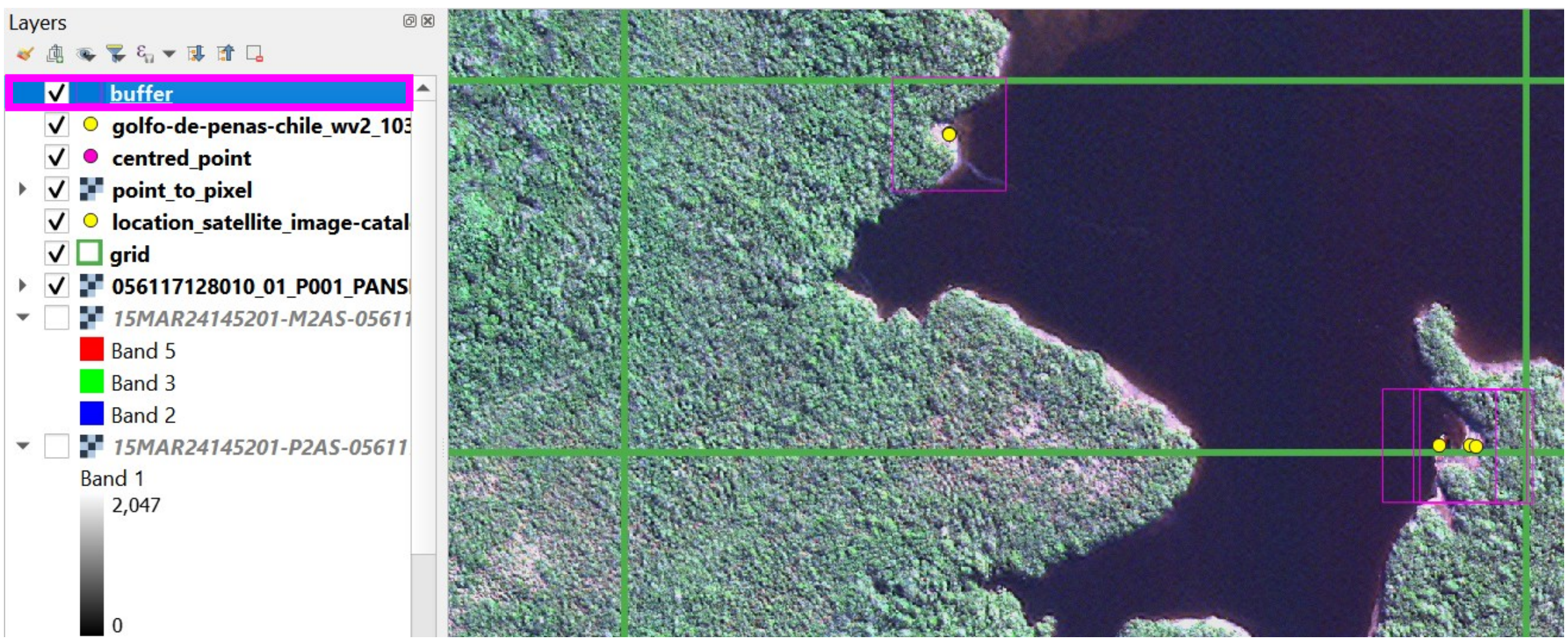

d. zoom into view the boxes to verify the boxes encompass the entirety of the features. If the buffer is too small or too large, right click the 'buffer' layer in the 'Layers' pane, 'Remove Layer…', 'OK'. Repeat section '23' again

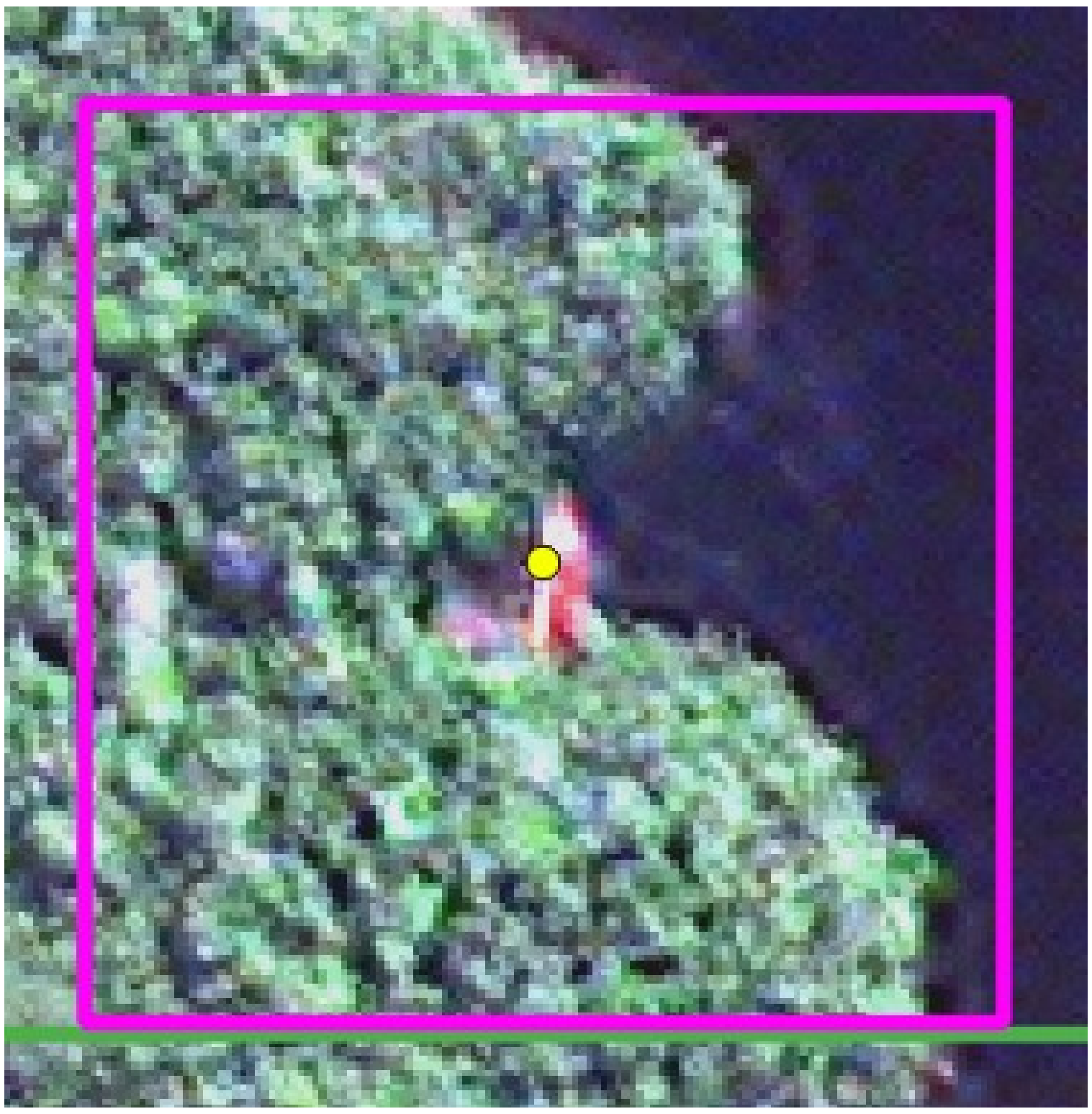

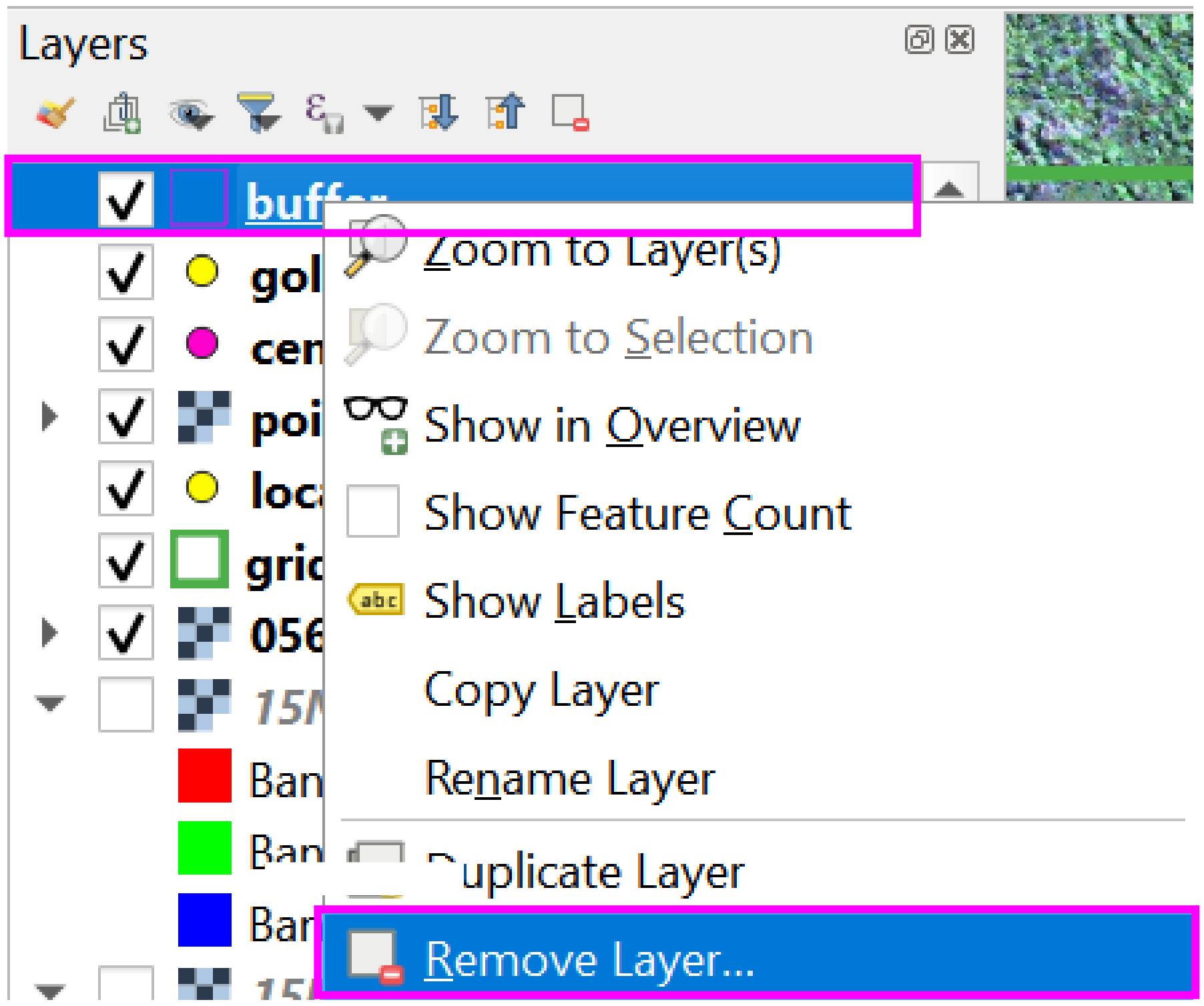

CREATING IMAGE CHIPS

24. Clipping raster by bounding boxes

Once the buffer layer is accurate, complete a raster extraction to clip raster by mask layer. This will clip the raster layer to create a new layer containing only the masked raster, the image chips.

a. in the 'Processing Toolbox' > GDAL > Raster Extraction > select 'Clip raster by mask layer'

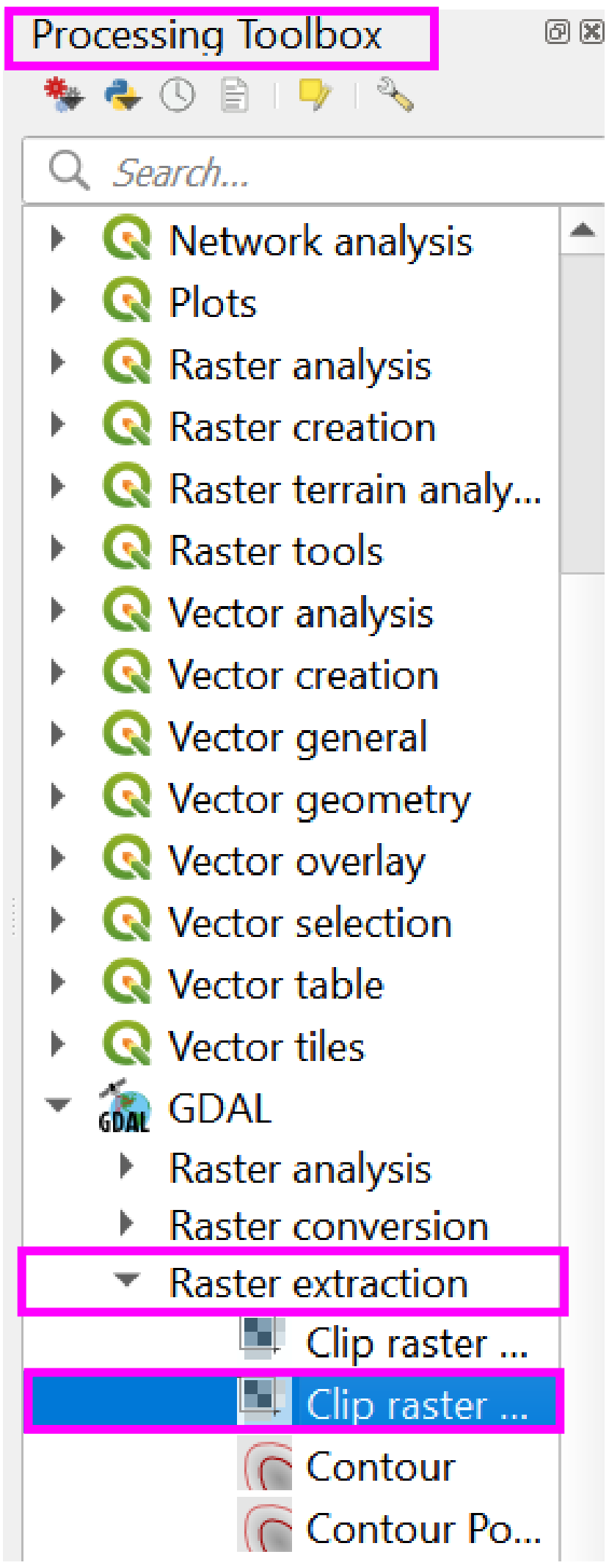

b. select the 'Input layer' as your pansharpened image, the 'Mask layer' as your 'buffer' layer (the newly created bounding box buffers). Next to the 'Mask layer', click the circular green arrow icon, unlike the buffer creation, we now want to create a separate image chip for every feature/buffer/bounding box. Set the 'Source' and 'Target CRS' as the 'Project CRS', select the up arrow in the 'Assign a specified no data value to output bands' from 'not set' to -999999999.000000. Check the box next to 'Create an output alpha band', 'Match the extent of the clipped raster to the extent of the mask' and 'Keep resolution of the input raster'. In the 'Clipped (extent)', select a location to save the file and use the same name given to the project and feature shapefile (each chip created will be assigned a value that will be added to the end of the name, _0, _1, _2 etc.), and 'Run'

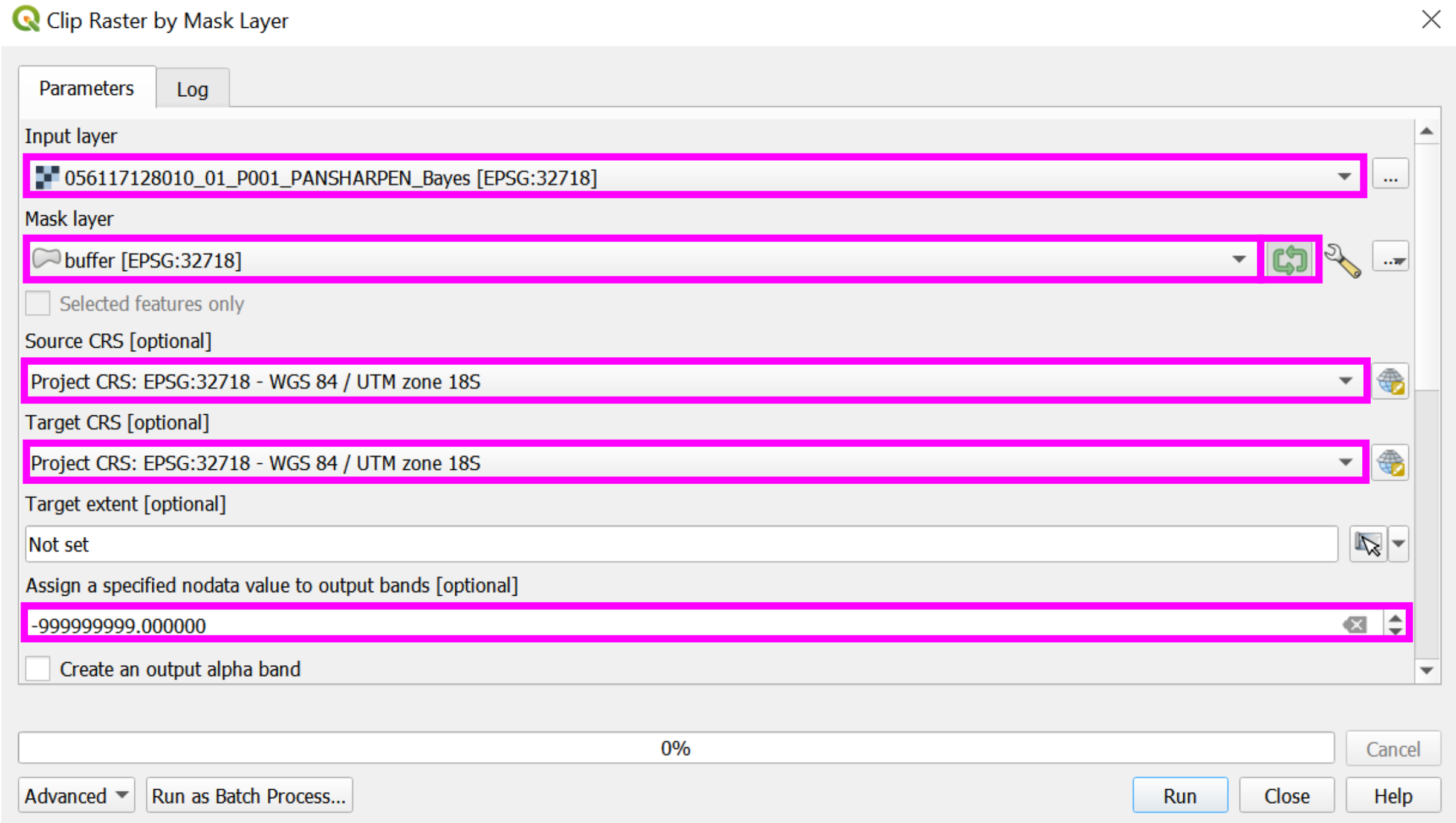

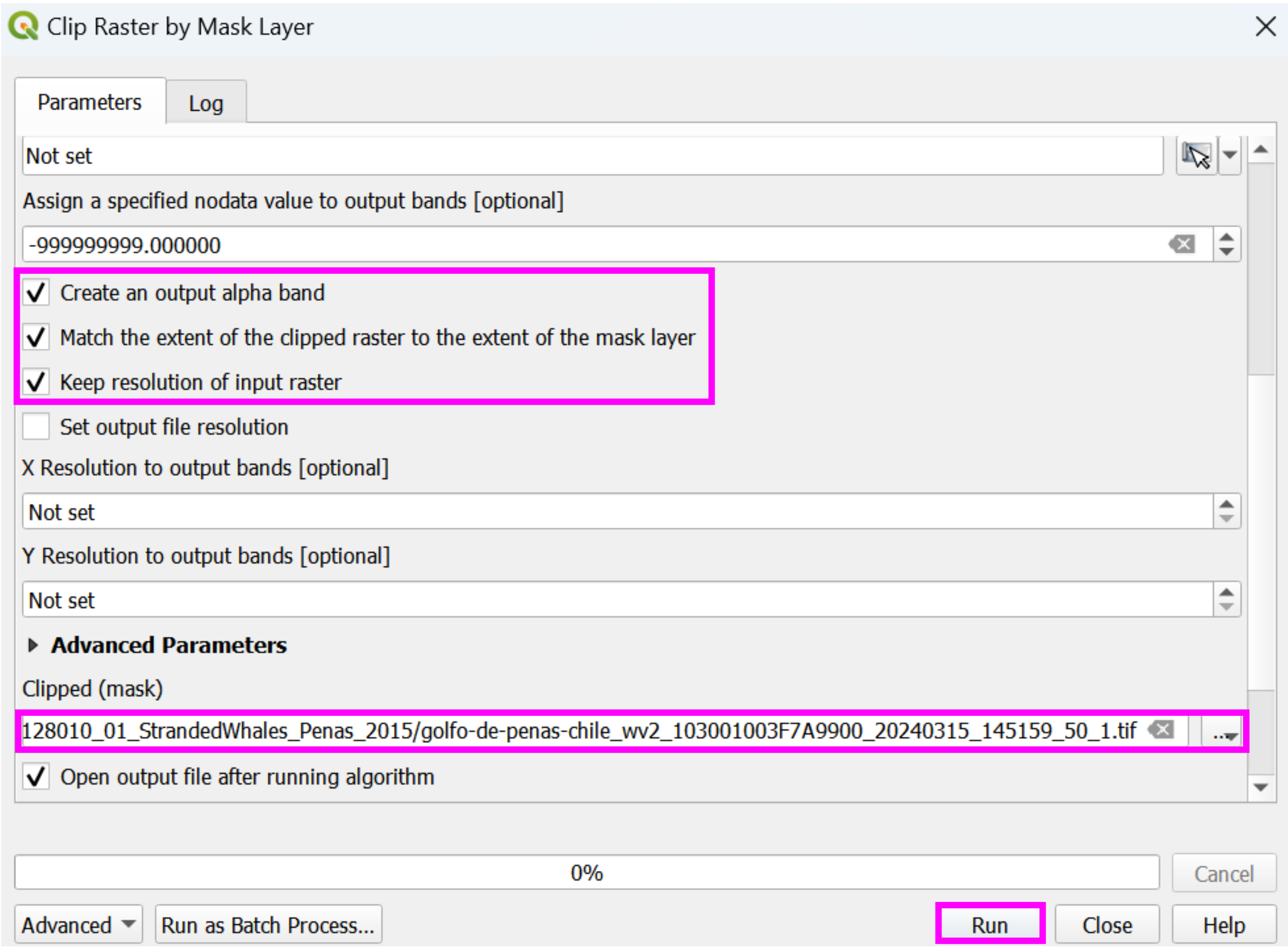

c. the output image chips will appear in the 'Layers' pane and will be visible in the main project window. The images will also appear in the directory they were saved to with the name convention assigned along with the newly assigned image 'id'. This 'id' should correspond to the original point feature 'id' if originally the 'id' assigned began at zero

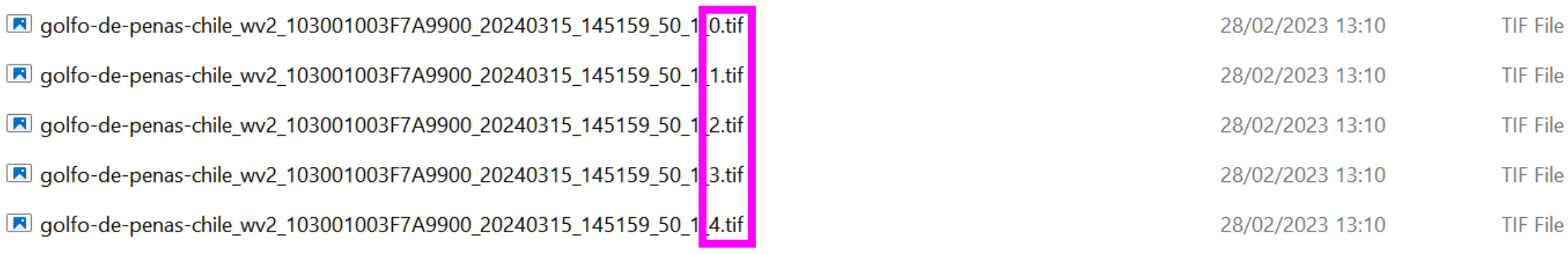

d. to amend the band combination for each image chip manually would be timely, an alternative is to 'Copy Style' of the pansharpened image and 'Paste Style' to each image chip. Right click the pansharpened image layer > 'Styles' > 'Copy Style'

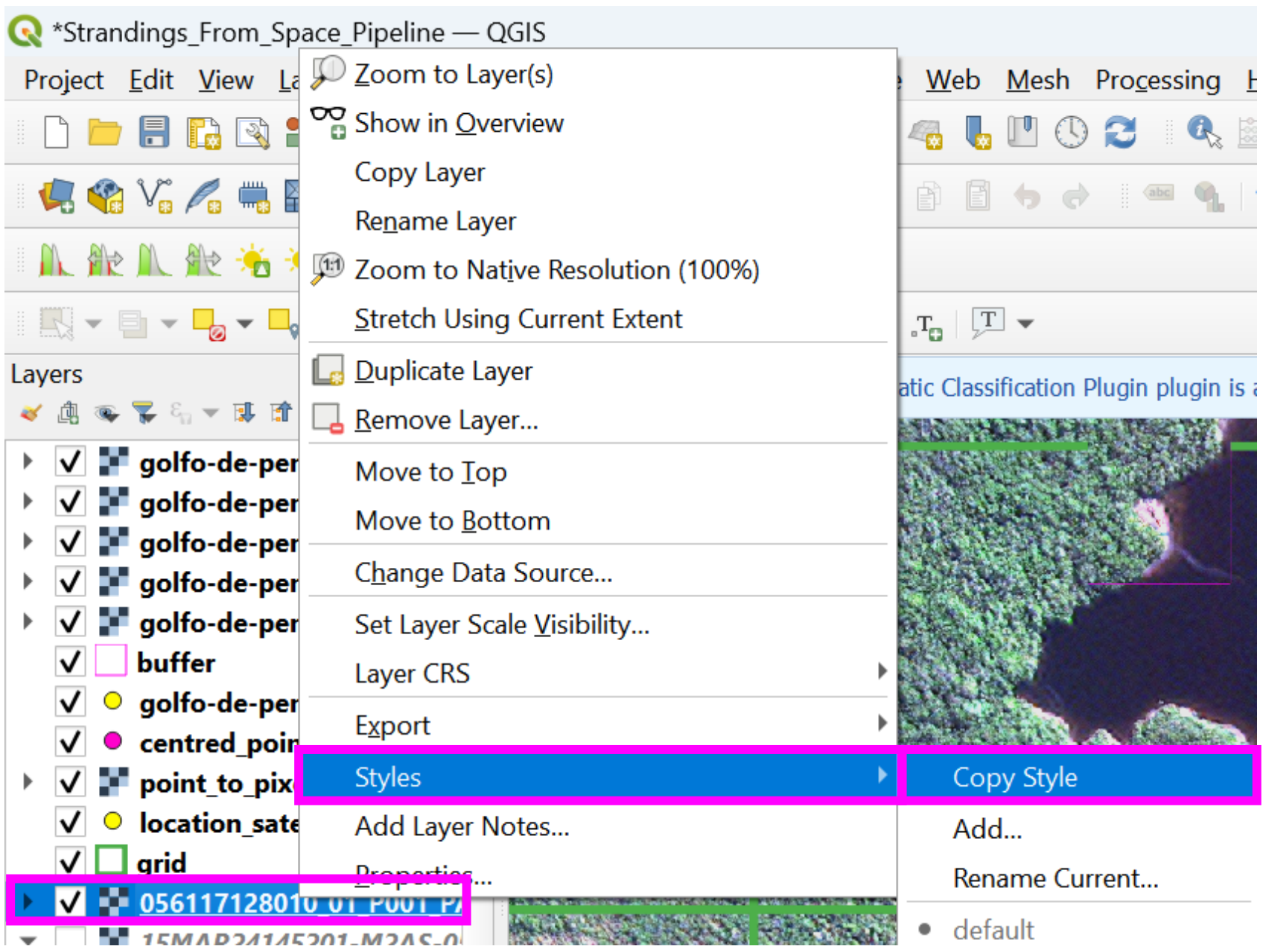

e. Click the first image chip, then hold 'shift' and click the last image chip, to select (highlight) all image chips. Right click the highlighted layers > 'Paste Style'

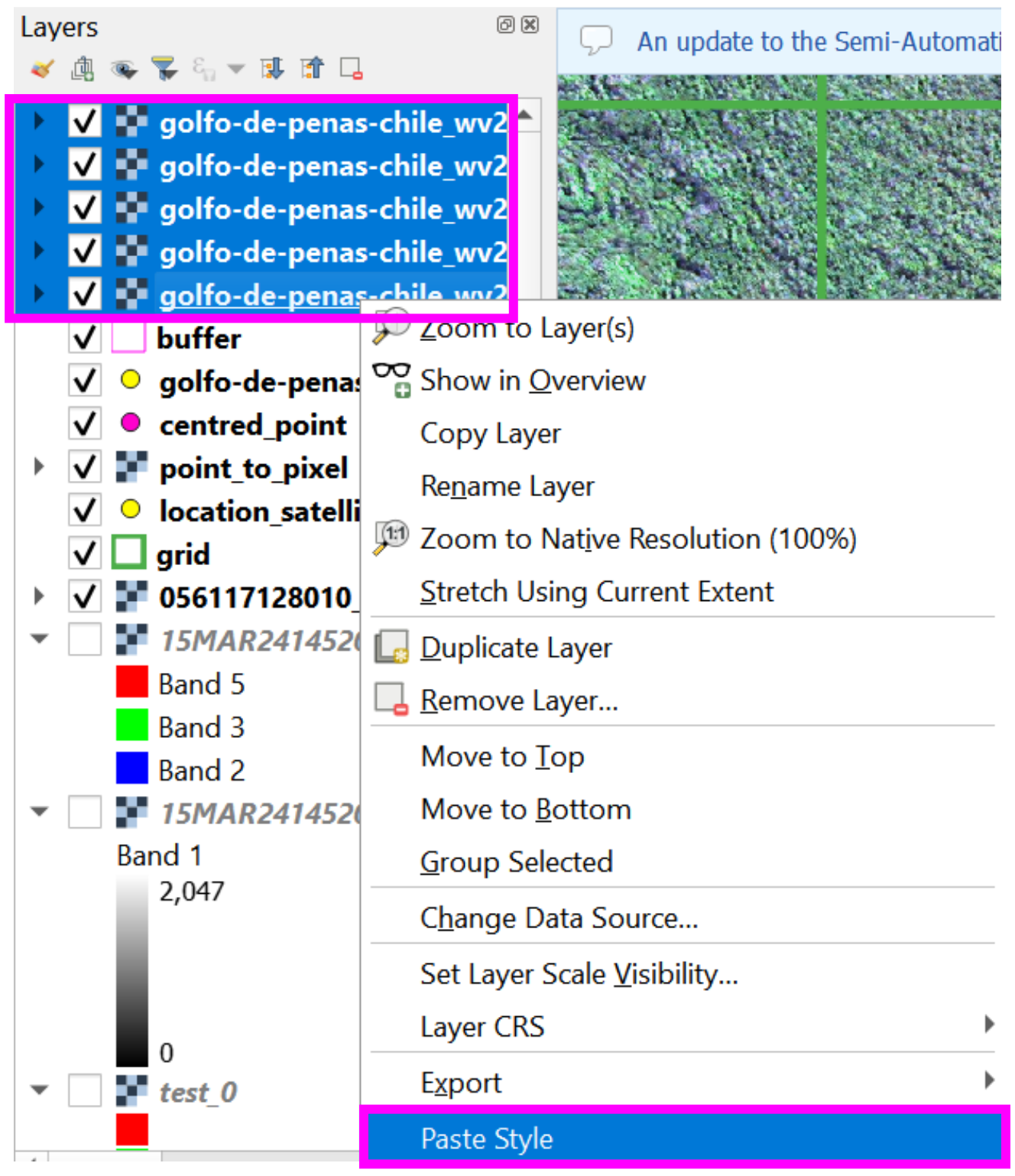

f. layers can be switched off or reordered in the 'Layers' pane using the check boxes to turn off/on and click drag and drop to reorder, to help visualise the new image chips

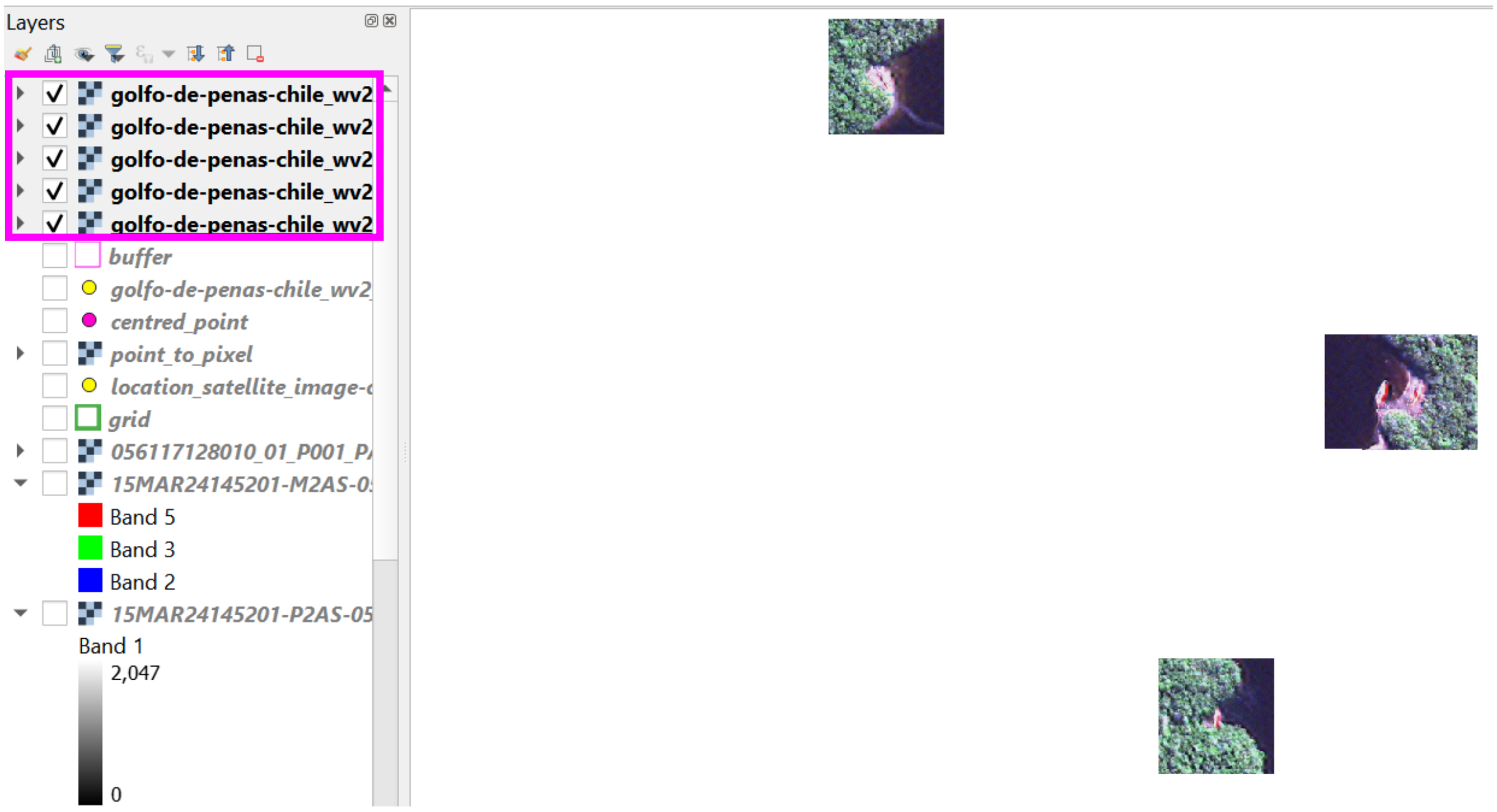

g. the tile may not align exactly with the buffer, this is because the tool has compensated to preserve the pixels over exact distance. To check the output is 128 x 128 pixels, right click one of the image chips in the 'Layers' pane > 'Properties' > 'Information' > the 'Width' and 'Height' should be equal to 128

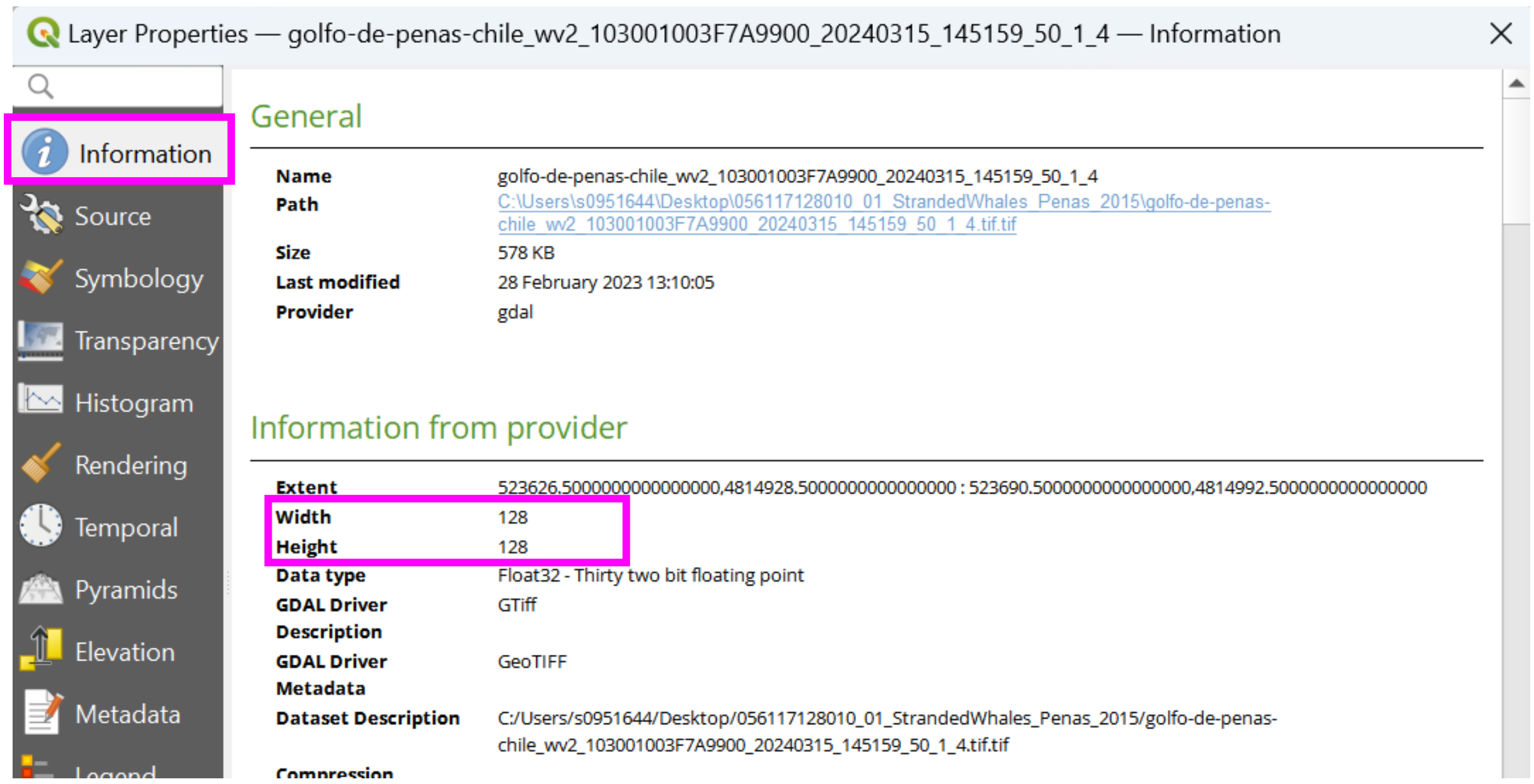

25. Extract image chips as .png format

   a. in the 'Layers' pane uncheck all layers other than the image chips to export

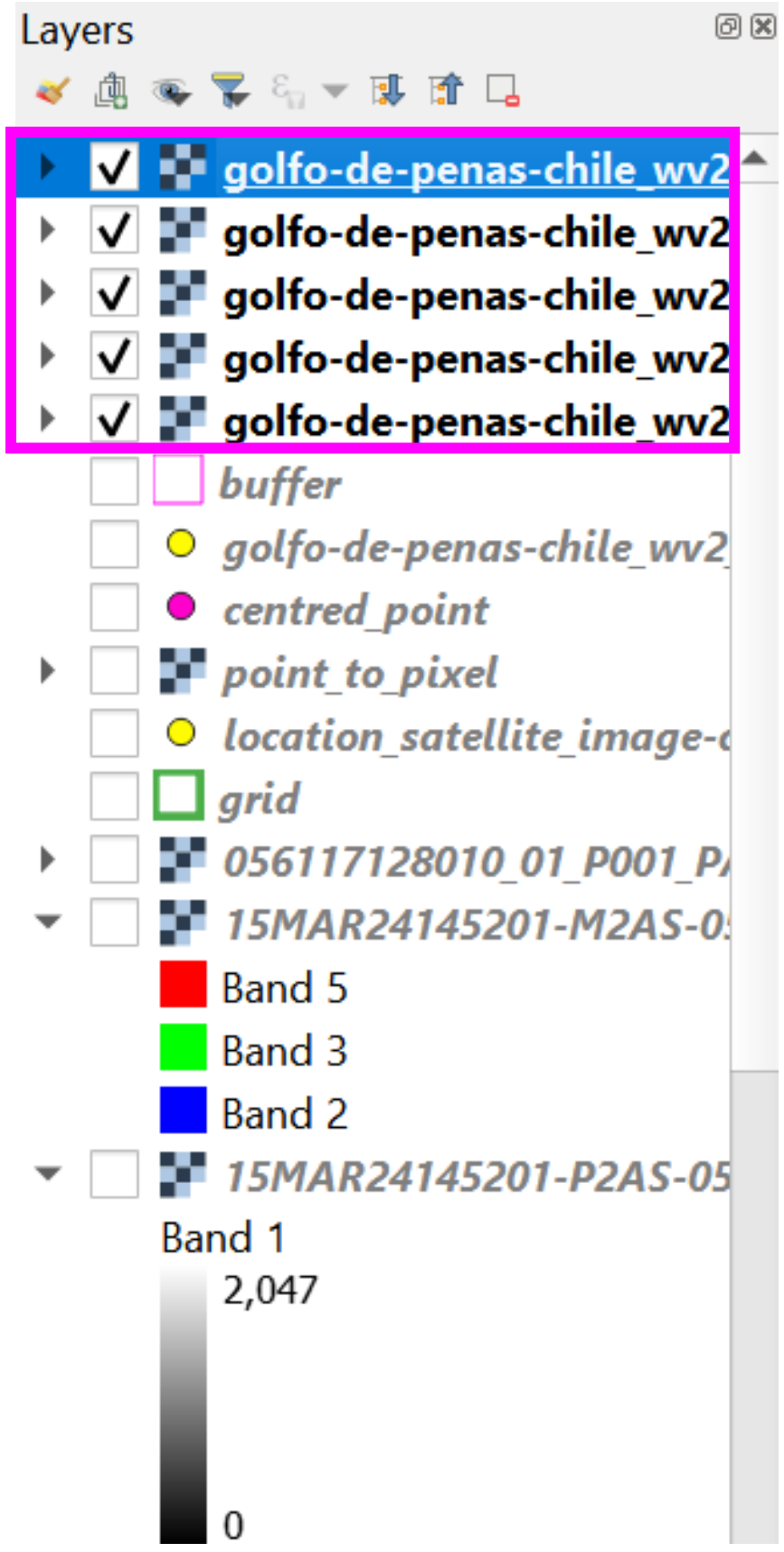

b. in the main QGIS toolbar select 'Plugins' > 'Python Console'

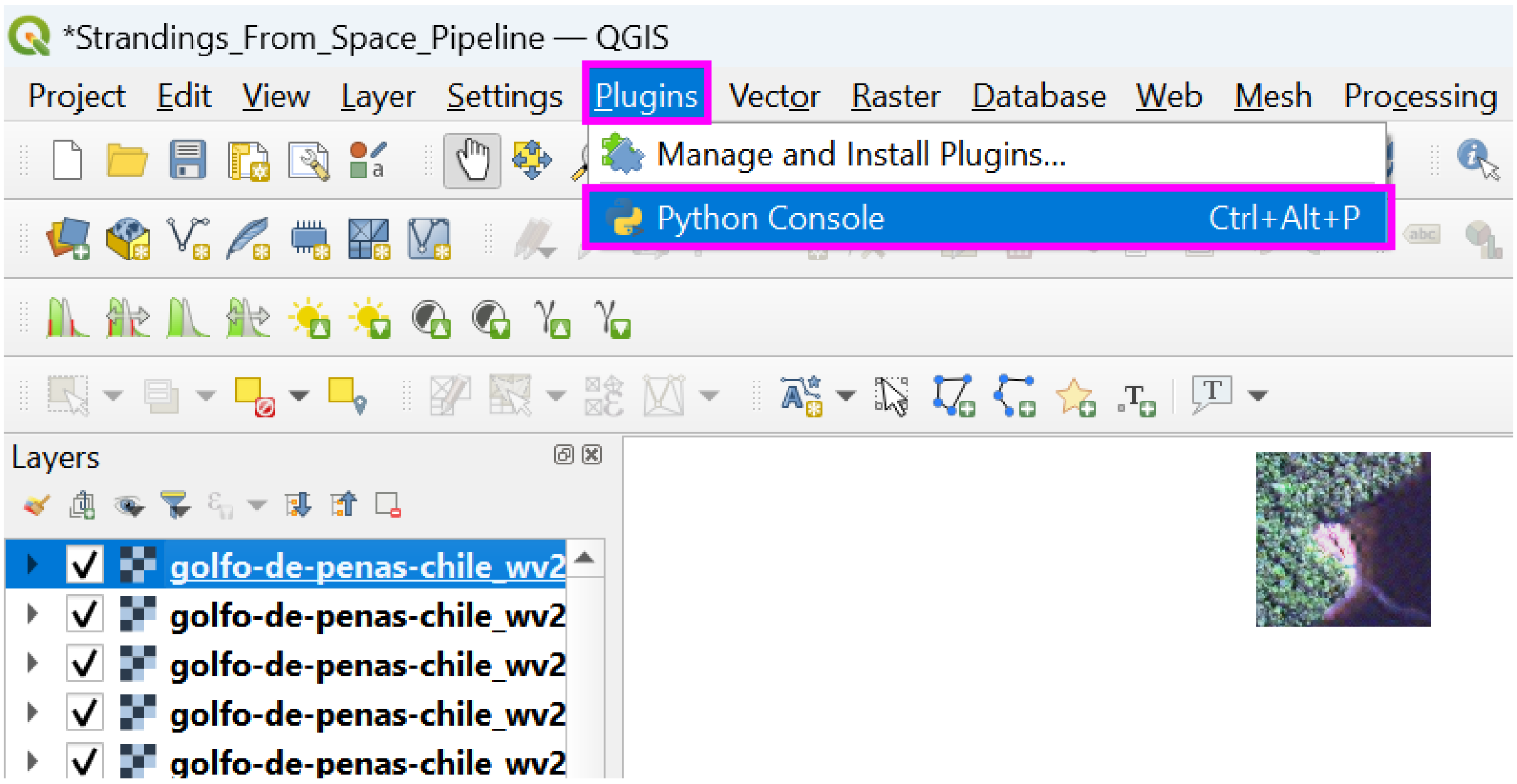

c. in the 'Python Console' select the 'Show Editor' icon (page with red pencil)

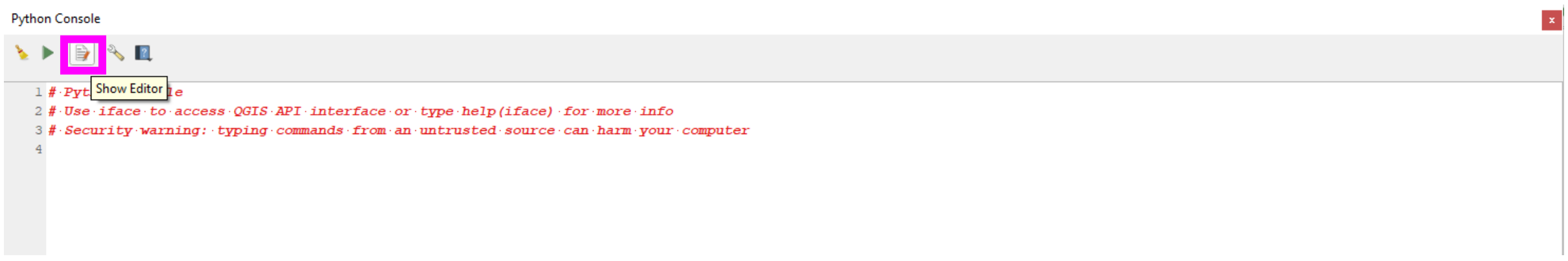

d. select the ‘Open Script’ icon (yellow folder)

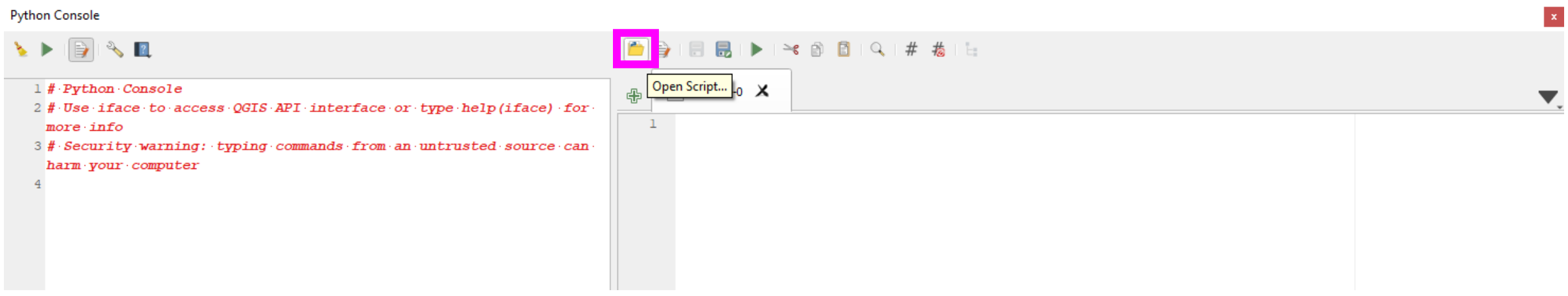

e. navigate to the downloaded supplemental material ‘S2_png_creation.py’ (or for the most up to date version visit https://github.com/PennyJClarke/strandings_from_space, and extract the ‘S3_png_creation.py’ script file), and ‘Open’

f. in the .py file that opens, ensure to amend the out_path to a suitable location on your directory and select the ‘Run Script’ icon (green triangle)

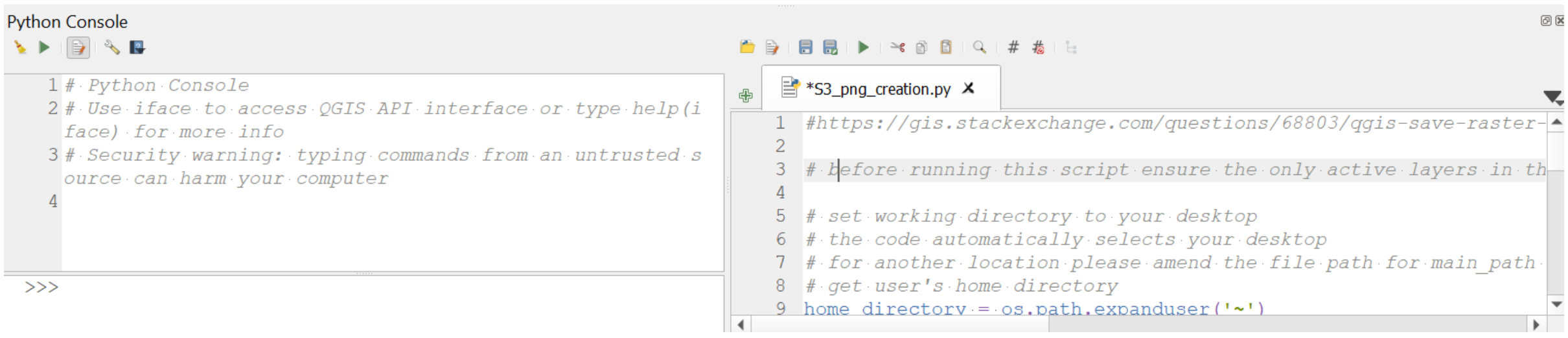

g. if the code runs successfully, the outputs will appear as a list of files created in the left of ‘Python Console’, and when navigating to your directory, the image chips should be available in the folder, named with the original buffer name, which corresponds to the ‘id’ of the original feature

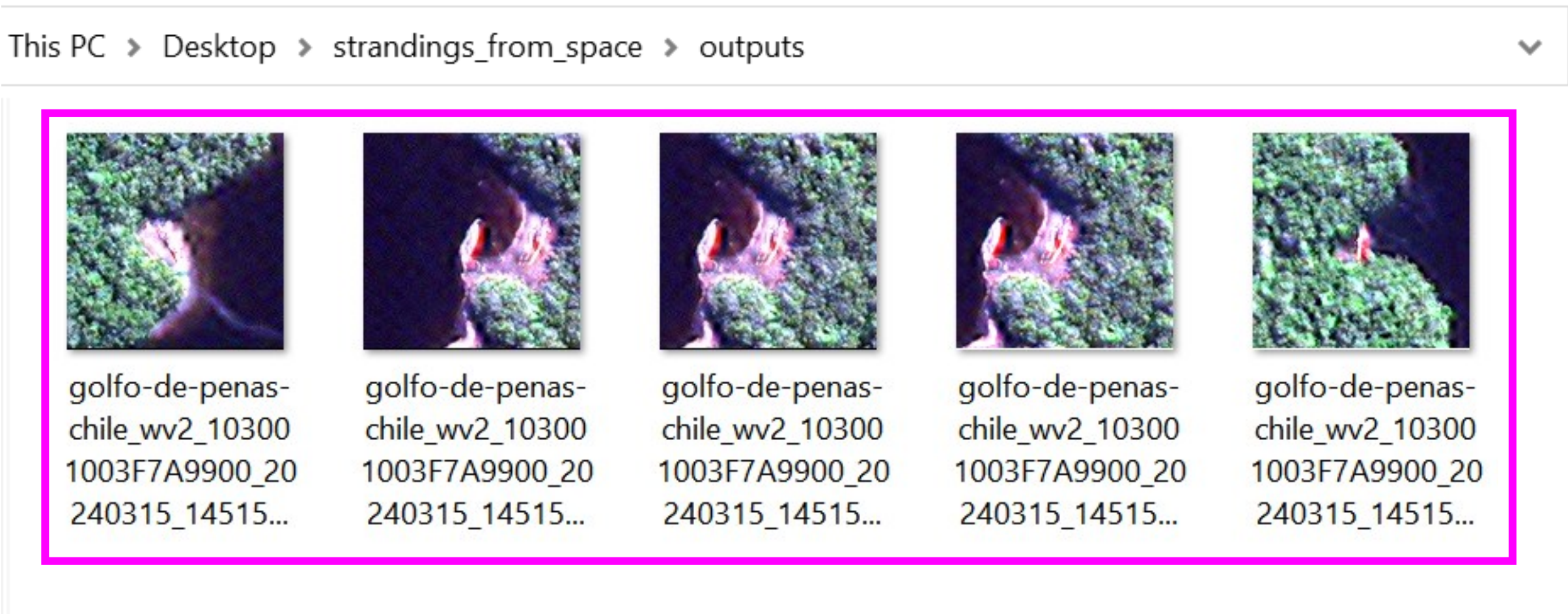

APPENDIX

APPENDIX 1: METADATA: ACCESS AND FINDING INFORMATION

1. Accessing satellite imagery metadata
    a. navigate to the panchromatic image folder in your directory (unless if the aim is to confirm the number of multispectral bands, then open the multispectral image metadata file), double click the last .XML file in the folder to open the image metadata in an internet browser, or right click the .XML file, and select ‘Open with’ > ‘Notepad’ or ‘Notepad++’. For Airbus imagery, the metadata can be located in a html file with the name beginning ‘DIM_…’

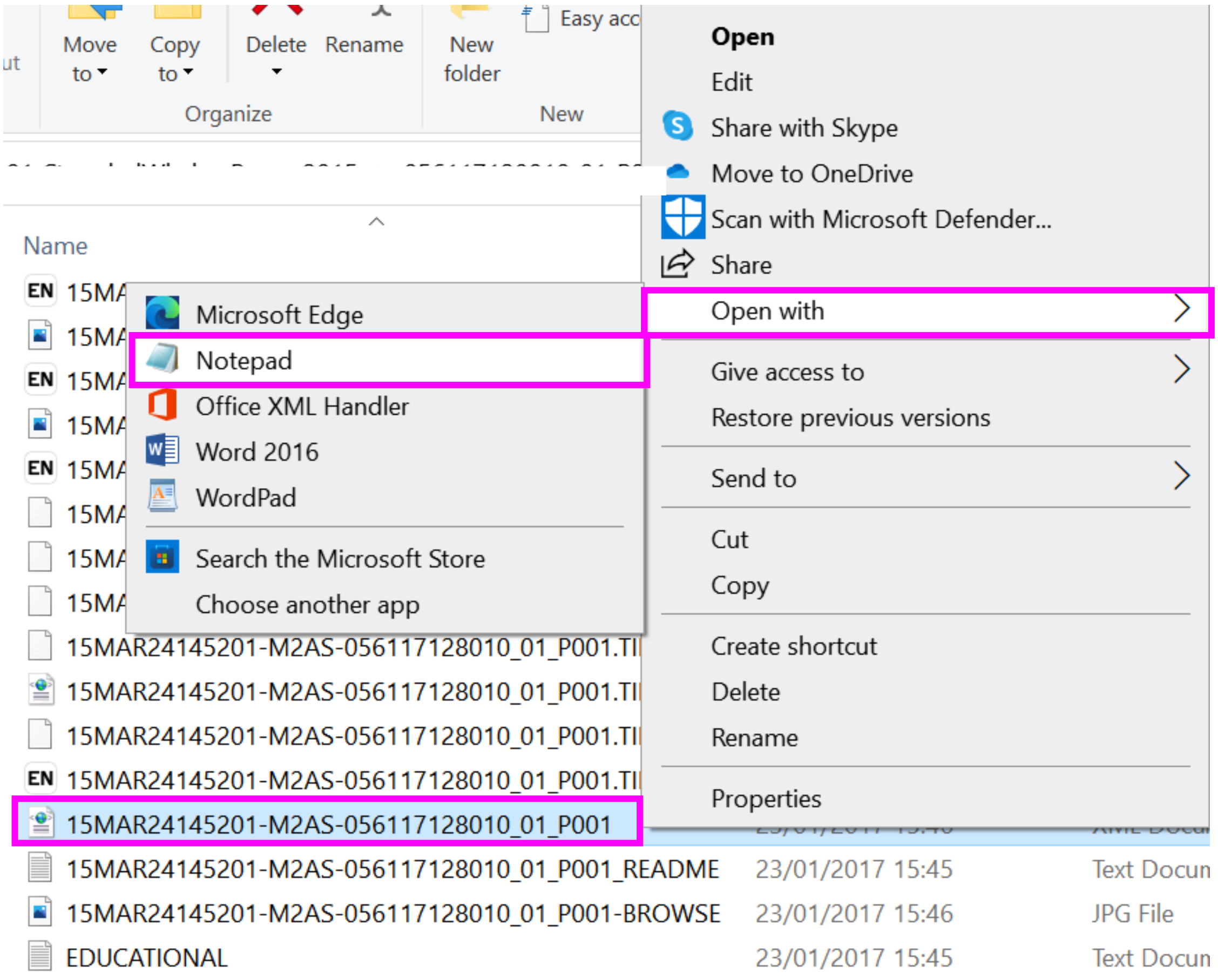

2. Identifying details within the metadata file
    a. using the ‘ctrl’ and ‘f’ keys, search the image metadata
        - see Table S1.A1.1 for key metadata attributes to aid the location of information within a satellite imagery metadata file. Using either terms from the ‘**Vantor metadata attribute’** or **‘Airbus metadata attribute’** columns, appropriate to your satellite imagery provider, ‘ctrl’ and ‘f’ the term of interest in the image metadata (*e.g.,* to locate the satellite sensor in Vantor metadata, ‘ctrl’ and ‘f’ search ‘SATID’, the corresponding value, ‘WV02’, indicates the name of the sensor that collected the VHR satellite image. For example, ‘WV02’ is equal to WorldView-2).

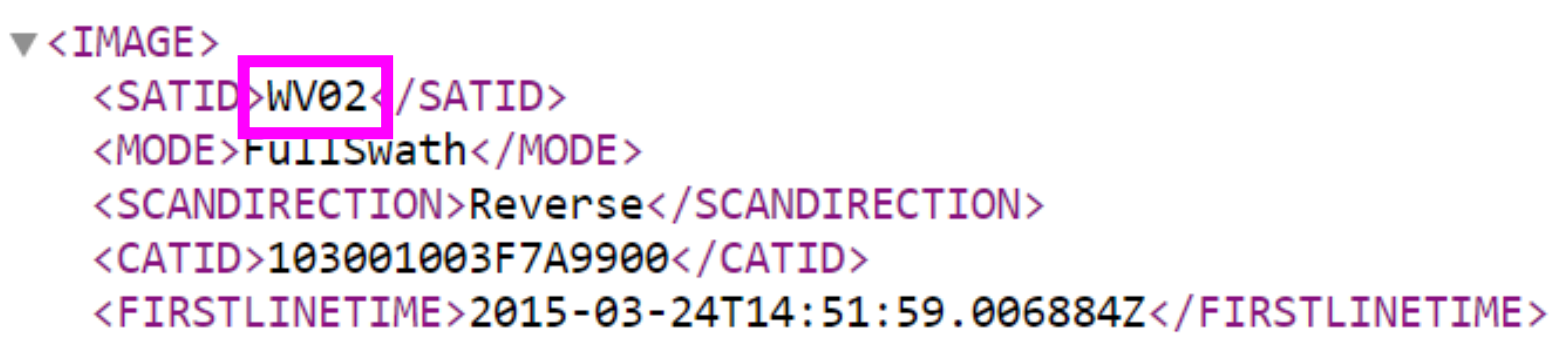

Table S1.A1.1: Key metadata terminology and example values for both Airbus and Vantor satellite imagery

Variable of interest	Description	Vantor metadata attribute	Example Vantor metadata value	Airbus metadata attribute	Example Airbus metadata value
Satellite sensor	Name of the sensor, which collected the satellite image	SATID	WV02	MISSION<br>MISSION_INDEX	PHR<br>1B
Image catalogue ID	Unique image ID assigned by the satellite image provider	CATID	103001003F7A9900	SOURCE_ID	DS_PHR1B_201811282258003_FR1_PX_E167S47_1106_03071
Image collection date	Date of image collection YYYYMMDD, ISO8601 format	FIRSTLINETIME	2015-03-24	IMAGING_DATE	2018-11-28
Image collection time	Time of image collection HHMMSS, ISO8601 format	FIRSTLINETIME	T14:51:59	IMAGING_TIME	22:58:00.3Z
Spatial resolution/ Ground sampling distance (GSD)	The spatial resolution of the satellite image (distance on the ground represented by a single pixel). The 'MEANCOLLECTEDGSD' and 'Ground_Sample_ Distance' is the raw spatial resolution at the time of image collection, and 'PRODUCTGSD' and 'RESAMPLING_SPACING' is the spatial resolution of the final processed image provided to the user. GSD values for Vantor products are in scientific notation, where 5.000000000000000e-01 is equivalent to 0.5 m, whereas the value for Airbus products is in units' metres (m). Airbus provide GSD for different locations in the image, select the	MEANCOLLECTEDGSD<br><br>PRODUCTGSD	4.900000000000000e-01<br><br>5.000000000000000e-01	Ground_Sample_ Distance<br>GSD_ACROSS_ TRACK<br>GSD_ALONG_ TRACK<br><br>RESAMPLING_SPACING	 <br>0.8736423525639271<br><br>0.8197207698277972<br><br>0.5

Variable of interest	Description	Vantor metadata attribute	Example Vantor metadata value	Airbus metadata attribute	Example Airbus metadata value
	centre value or an average of all values. The centre value can be identified using: LOCATION_TYPE>Center </LOCATION_TYPE.				
Panchromatic or multispectral	Electromagnetic radiation with emission properties in the visible electromagnetic spectrum that produces greyscale imagery (panchromatic) and colour imagery (multispectral) (Dowman *et al.*, 2012; Rees, 2013).	BANDID	'P' or 'Multi'<br><br>For four band multispectral imagery: BAND_B, BAND_G, BAND_R, BAND_N<br><br>For eight band multispectral imagery: BAND_C, BAND_B, BAND_G, BAND_Y, BAND_R, BAND_RE, BAND_N, BAND_N2	NBANDS<br>BAND_ID<br><br>BAND_NAME	1, 4, or 6<br>PAN, or B0, B1, B2, B3, or R, G, B, NIR, RE, DB<br>PANCHROMATIC or Red, Green, Blue, NIR, Red Edge, Deep Blue
Image product type	Level of pre-processing defined by the user at the time of ordering.	IMAGEDESCRIPTOR<br>PRODUCTTYPE<br>PRODUCTLEVEL	Standard2A<br>LV2A<br>Standard	PROCESSING_ LEVEL<br>RADIOMETRIC_ PROCESSING	ORTHO<br><br>REFLECTANCE
Image projection	Assigned Coordinate Reference System	MAPPROJNAME<br>MAPZONE	UTM<br>18	PROJECTED_ CRS_NAME<br>PROJECTED_ CRS_CODE	*e.g.,* WGS 84 / UTM zone 1S<br><br>*e.g.,* crs:EPSG::32701
Nadir angle (°)	The angle of the satellite to the target area or feature on Earth, at the time of collection. Values for Vantor are in scientific notation where 2.670000000000000e+01 is equivalent to 26.7°	MEANOFFNADIRVIEWANGLE	2.670000000000000e+01	VIEWING_ANGLE	24.197206168844456

Variable of interest	Description	Vantor metadata attribute	Example Vantor metadata value	Airbus metadata attribute	Example Airbus metadata value
Sun azimuth angle (°)	The sun's relative direction along the horizon at the time of image collection. Values for Vantor are in scientific notation where 4.130000000000000e+01 is equivalent to 41.3°	MEANSUNAZ	4.130000000000000e+01	SUN_AZIMUTH	46.740780992739836

APPENDIX 2: CREATING A TEMPLATE SHAPEFILE

The following guidance can be used by the strandings from space community to manually create the data standard template. Additionally, this guidance can be used to inform users on how to add new standards as the field evolves, or how to create a new custom template for users applying this workflow to new projects or target features.

1. Creating a new point shapefile
   a. Select 'New Shape Layer…' from the main toolbar

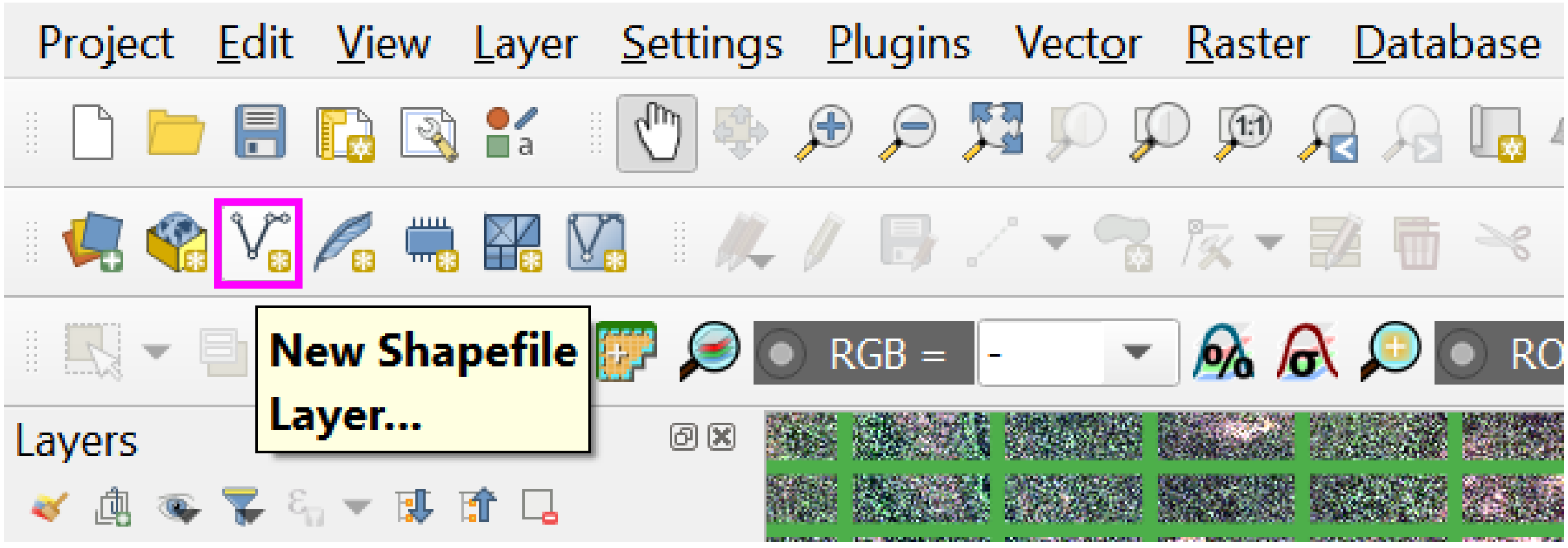

   b. for 'File Name' define a suitable location to save to and name using the recommended naming convention guidance in section 3.3.d. Set the 'Geometry type' to 'Point', ensure the 'Additional dimensions' match those of the project/raster layer (satellite image), and 'OK'

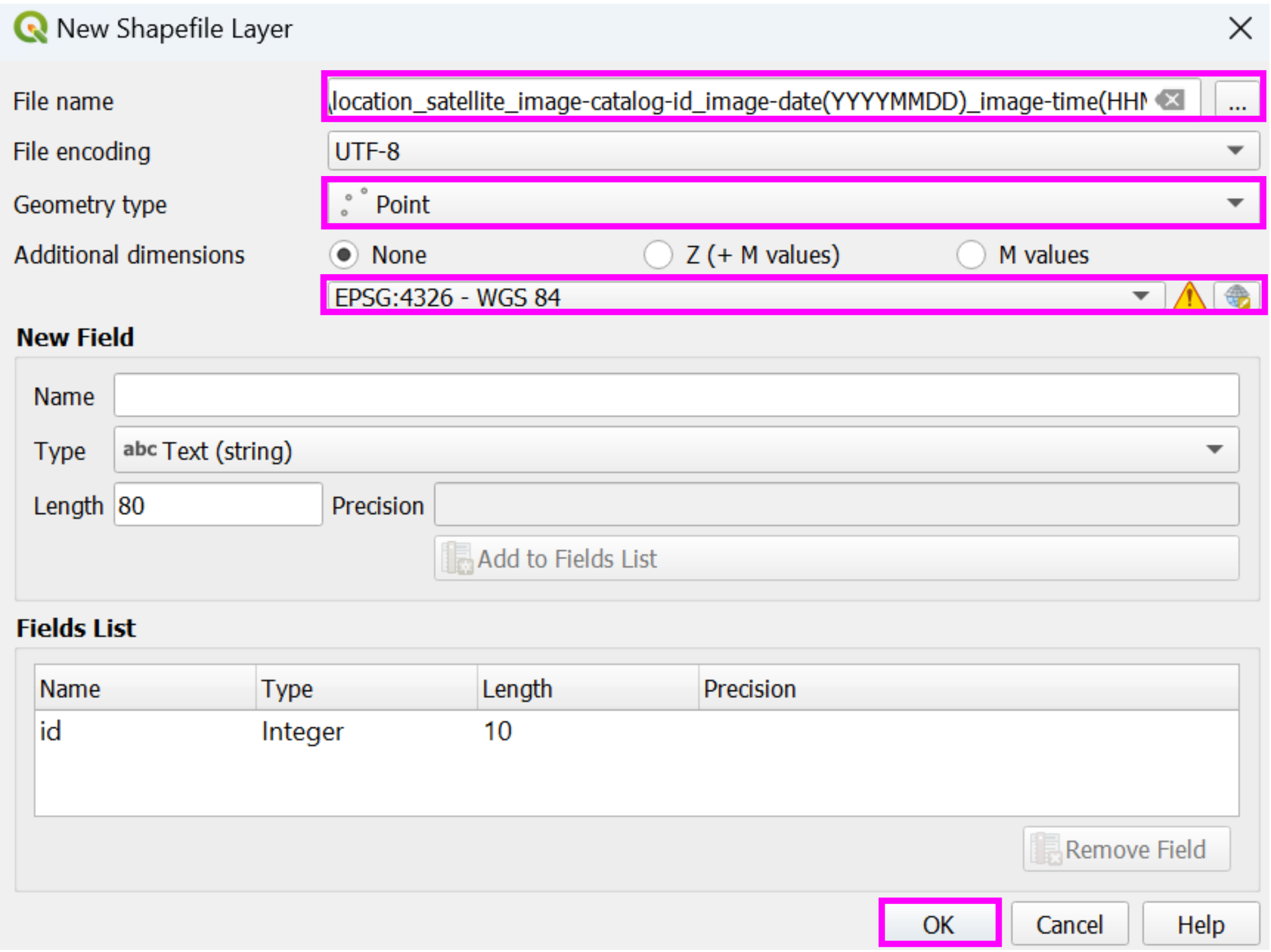

c. the shapefile will appear in the ‘Layers’ pane of the QGIS project and as a .shp file format in the selected directory

2. Defining fields and drop-down options

The attribute table of a point shapefile stores and displays information for a given layer.

a. to create new ‘Fields’ (new columns in the attribute table), right click the shapefile layer in the ‘Layers’ pane > ‘Properties’, select the ‘Fields’ tab. ‘id’ will appear as a default field. To add a new field, switch on ‘Toggle editing mode’ (yellow pencil icon), select the ‘New Field’ icon (table with yellow pencil)

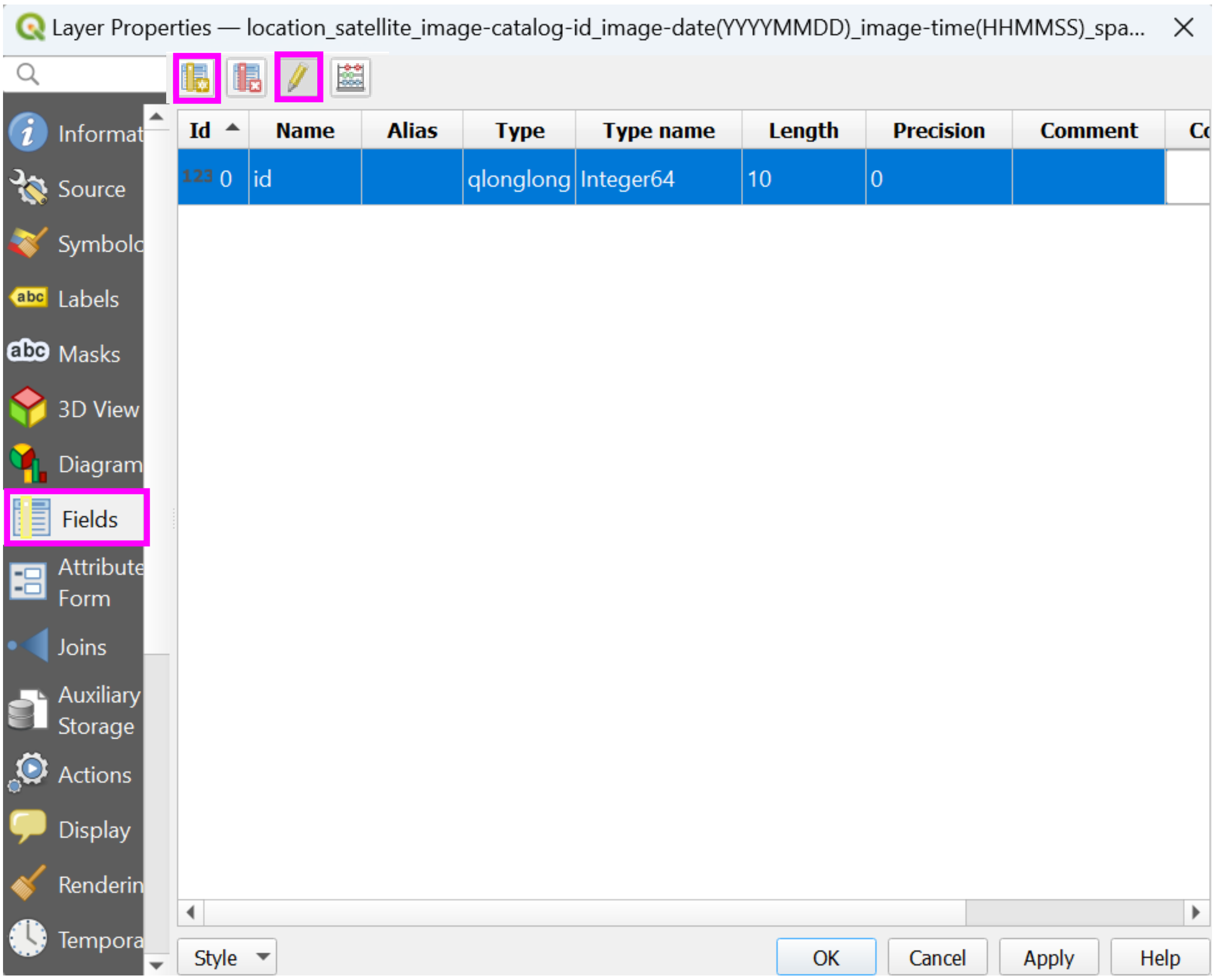

b. in the ‘Add Field’ window, set the field name in ‘Name’, set the ‘Type’ relevant for your column, for example, ‘Text (string)’ for columns expecting words and ‘Integer’ or ‘Decimal number’ for entries expecting a numerical input. The length of the expected entry can also be set, this is useful for example for, ‘Decimal numbers’ to limit the number of decimal point values. Select ‘OK’ and repeat for as many columns/new fields that you wish to enter, once finished, select ‘Apply’ and ‘OK’

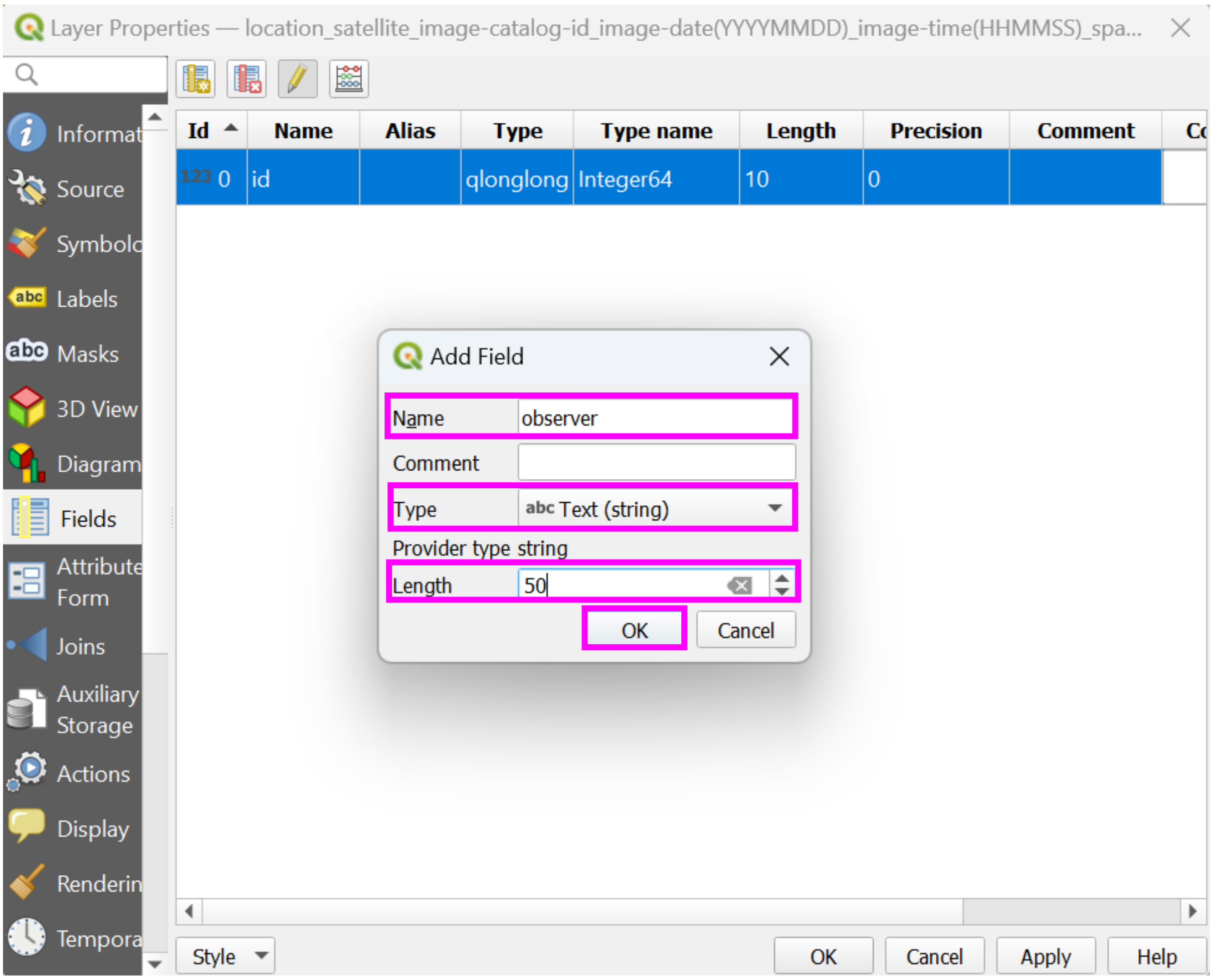
Layer Properties — location_satellite_image-catalog-id_image-date(YYYYMMDD)_image-time(HHMMSS)_spa...
Informat
Source
Symbolo
Labels
Masks
3D View
Diagram
Fields
Attribute Form
Joins
Auxiliary Storage
Actions
Display
Renderin
Tempora
Id Name Alias Type Type name Length Precision Comment
0 id qlonglong Integer64 10 0
Add Field
Name observer
Comment
Type abc Text (string)
Provider type string
Length 50
OK
Cancel
Style
OK
Cancel
Apply
Help

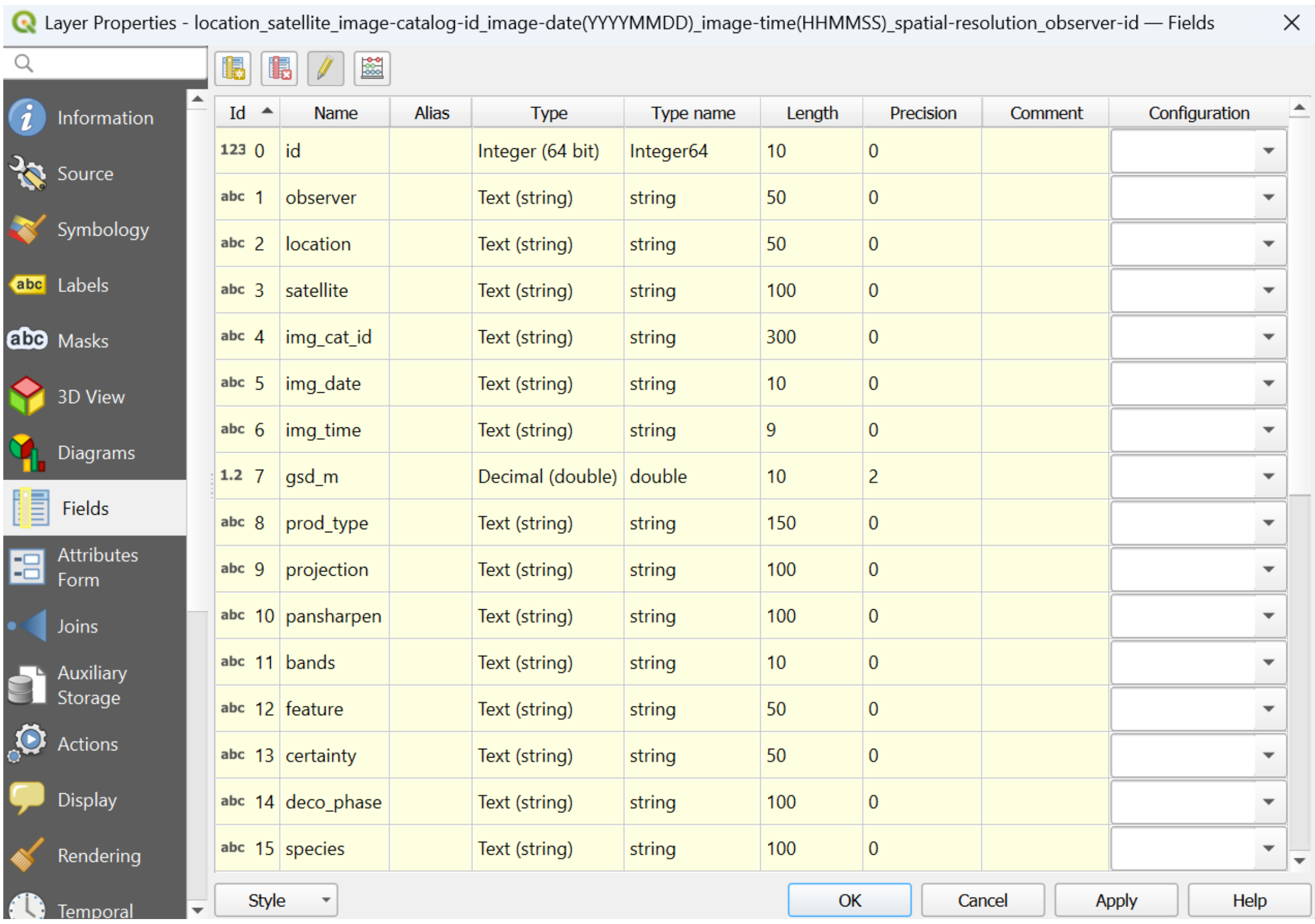
Layer Properties - location_satellite_image-catalog-id_image-date(YYYYMMDD)_image-time(HHMMSS)_spatial-resolution_observer-id — Fields
Information
Source
Symbology
Labels
Masks
3D View
Diagrams
Fields
Attributes Form
Joins
Auxiliary Storage
Actions
Display
Rendering
Temporal
Id Name Alias Type Type name Length Precision Comment Configuration
123 0 id Integer (64 bit) Integer64 10 0
abc 1 observer Text (string) string 50 0
abc 2 location Text (string) string 50 0
abc 3 satellite Text (string) string 100 0
abc 4 img_cat_id Text (string) string 300 0
abc 5 img_date Text (string) string 10 0
abc 6 img_time Text (string) string 9 0
1.2 7 gsd_m Decimal (double) double 10 2
abc 8 prod_type Text (string) string 150 0
abc 9 projection Text (string) string 100 0
abc 10 pansharpen Text (string) string 100 0
abc 11 bands Text (string) string 10 0
abc 12 feature Text (string) string 50 0
abc 13 certainty Text (string) string 50 0
abc 14 deco_phase Text (string) string 100 0
abc 15 species Text (string) string 100 0
Style
OK
Cancel
Apply
Help

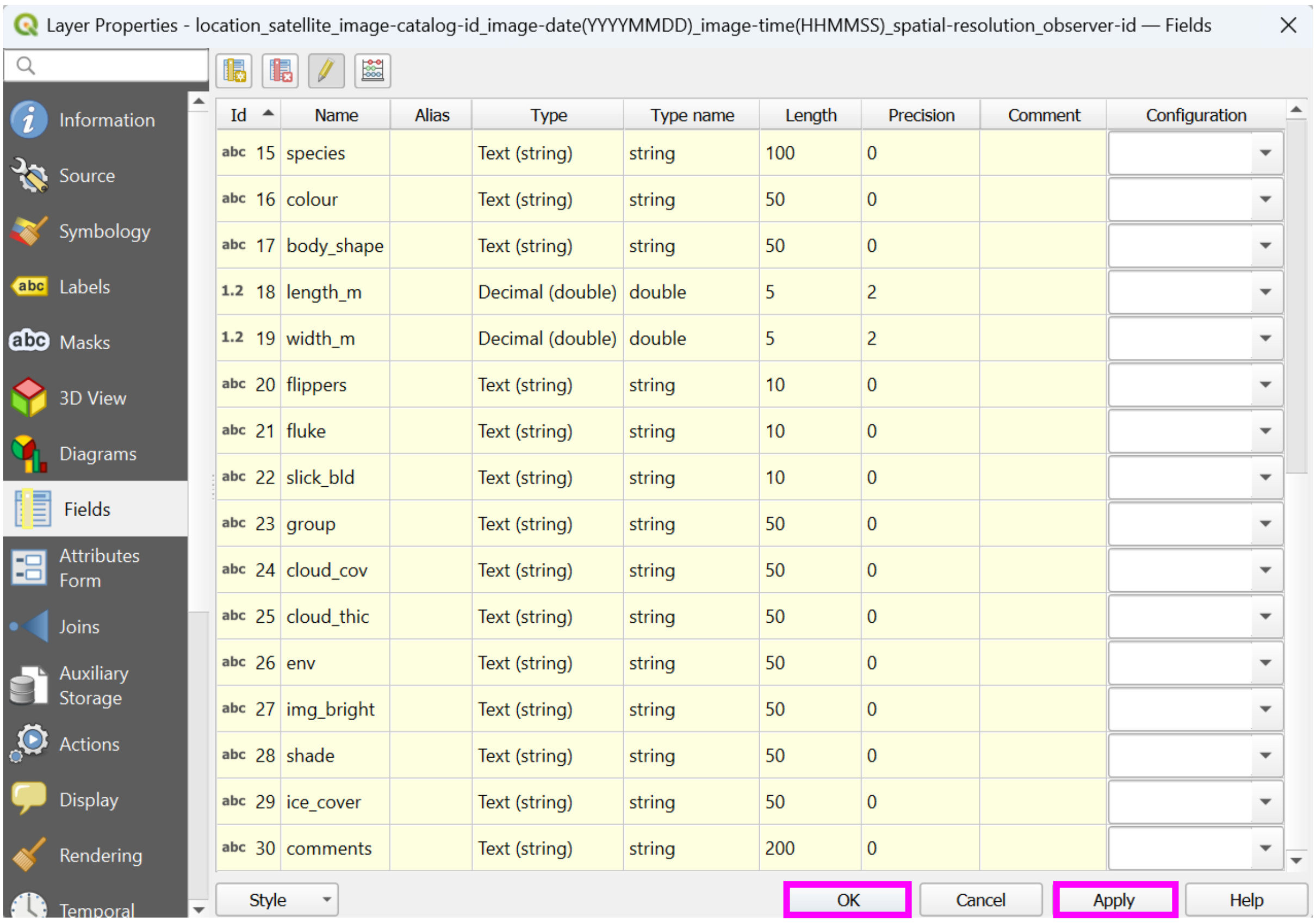

3. Defining drop-down options and 'Value Map' entries for template shapefile
    a. to add the 'Value Map' entries to the 'Fields' manually and set the attribute properties, import the .csv files downloaded in supplemental material 'S4_template_shapefile' (or for the most up to date version the 'template_shapefile > 'value_map' folder available at https://github.com/PennyJClarke/strandings_from_space), containing the entries. In the 'Properties' window, select 'Attributes Form', select each field in turn and add the relevant .csv file and set the attribute properties (see the relevant properties for each field below and in Table S1.A2.1), or manually add new entries.
        i. 'id': it is recommended this value is entered manually for each new feature, starting at 0, assign each new feature an incremental numerical value, for example, 1, 2, 3, 4… (no amendments are required to the 'Attributes Form')

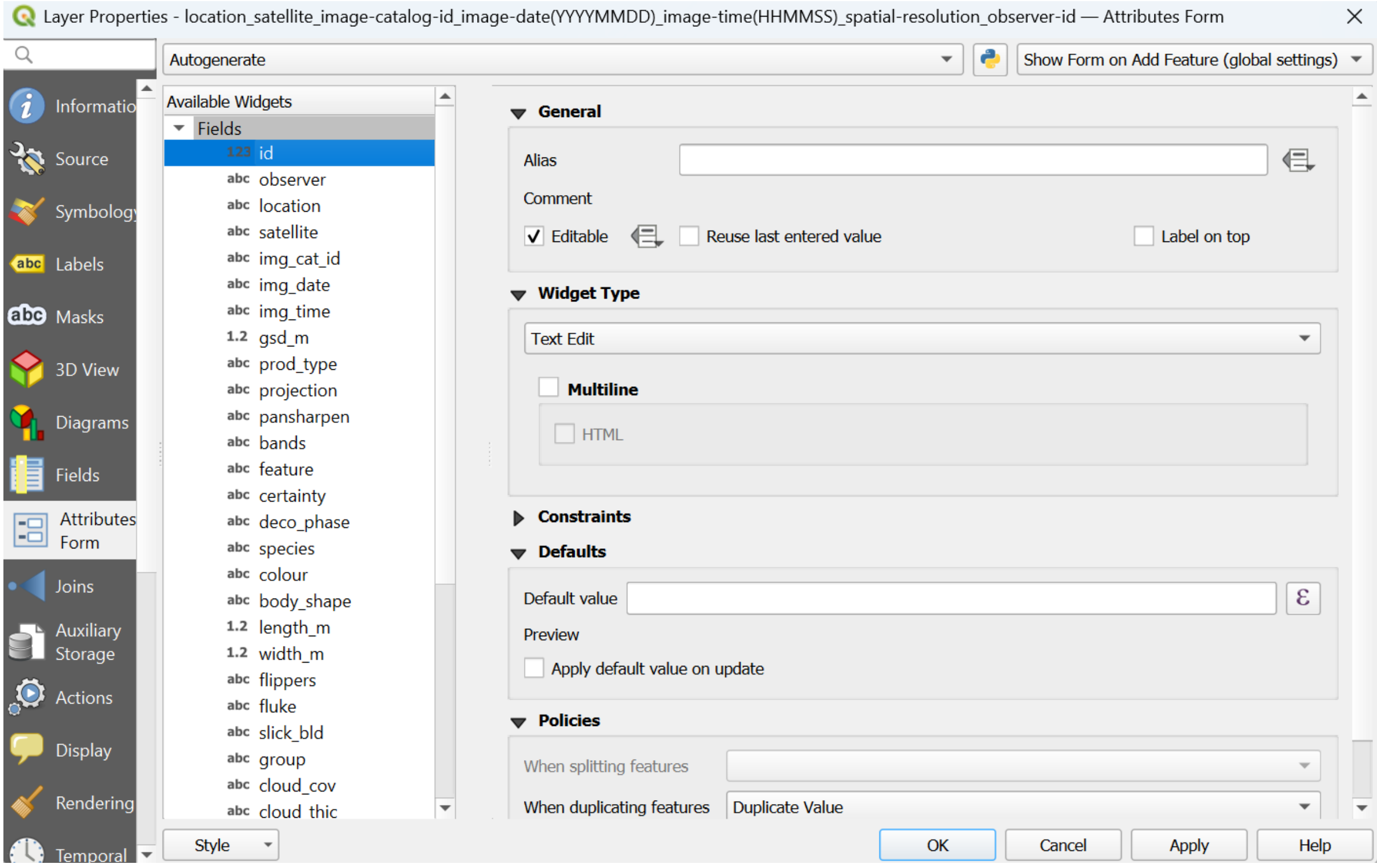

Note: There is an option to make this field populate automatically with the next incremental value, however, this is not recommended. This method requires the ‘Apply default value on update’ to be checked, which results in amendments to the ‘id’ value anytime a change is made. If the more automated method is preferred, uncheck ‘Editable’, add maximum(“id”) + 1 to the ‘Default value’ and check the ‘Apply default value on update’, and ‘Apply’.

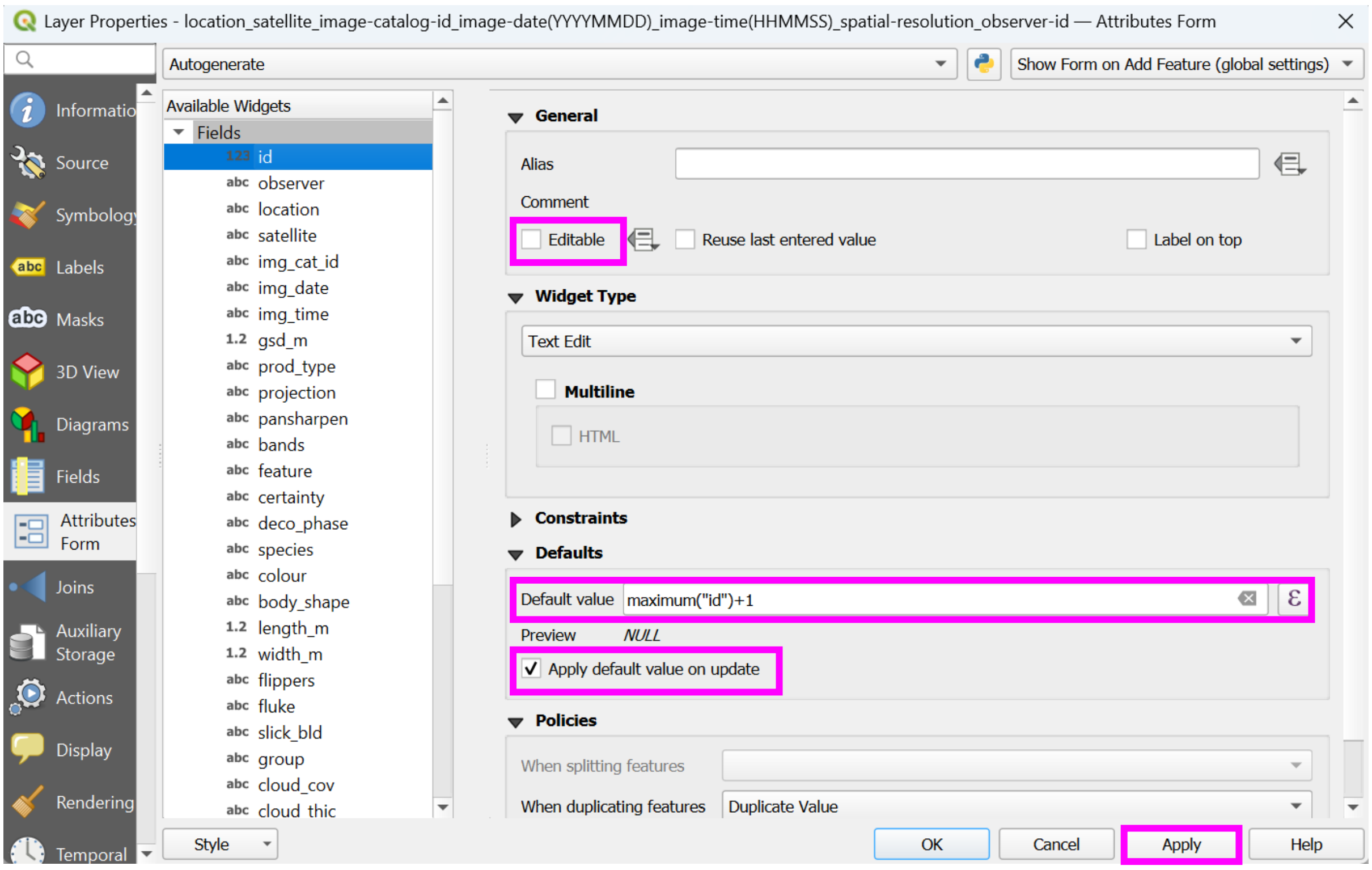

See Table S1.A2.1 for the properties of the remaining attributes. For entries requiring 'Value Map' dop-down entries, for example, 'satellite', select 'Load Data from CSV File' navigate to the supplemental material 'S4_template_shapefile' 'value_map' folder (or for the most up to date version visit https://github.com/PennyJClarke/strandings_from_space), and locate the 'satellite' .csv file, and 'Apply'. For full details on the attributes and values, and for image examples of the data standard for the cetacean strandings community, please see supplemental material S3. To add a new 'Value Map' dop-down entry, either create a .csv file listing your standard values, per attribute and 'Load Data from CSV File', or manually type entries directly into the 'Value' and 'Description' columns in the 'Layer Properties' window.

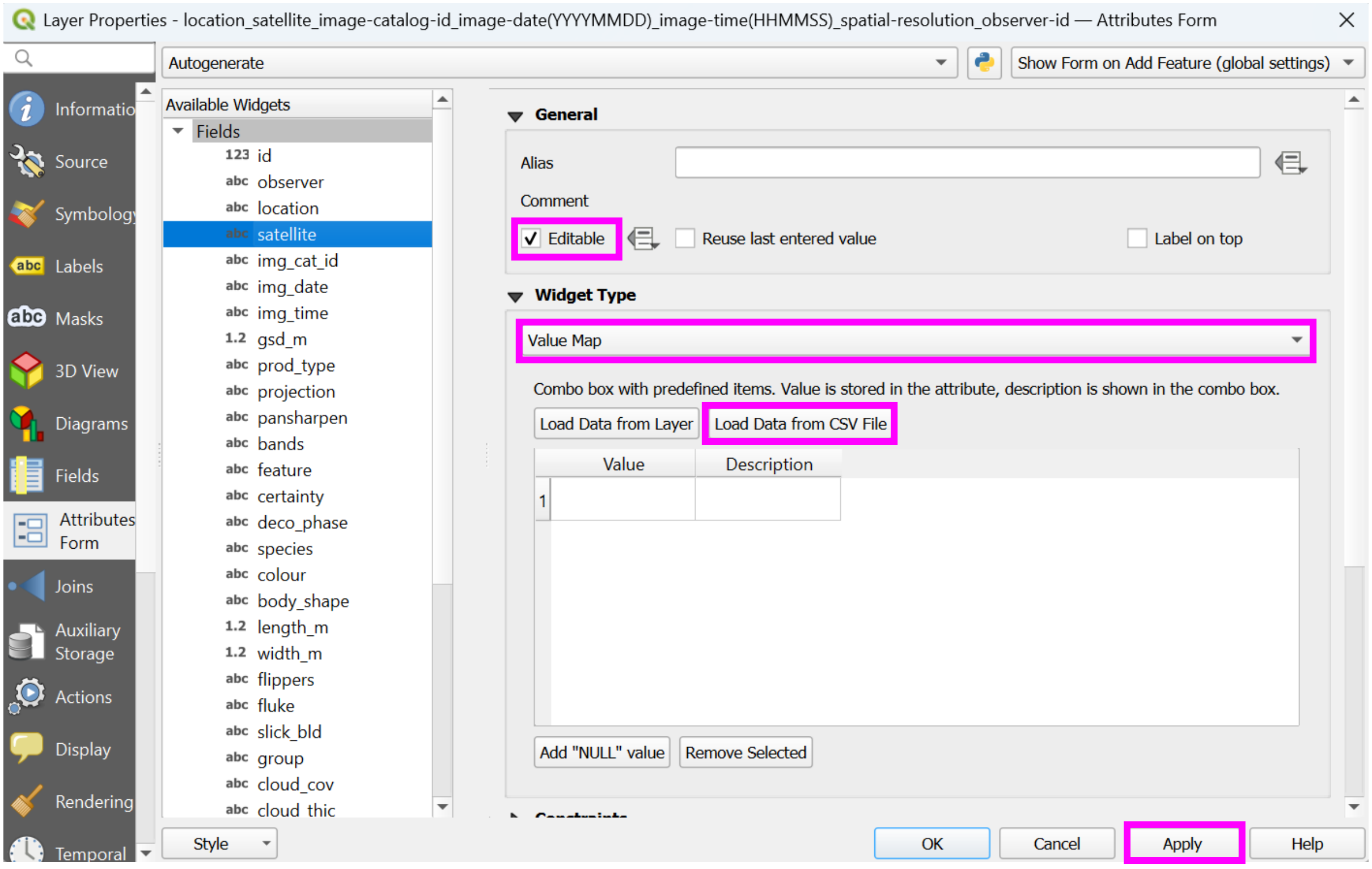

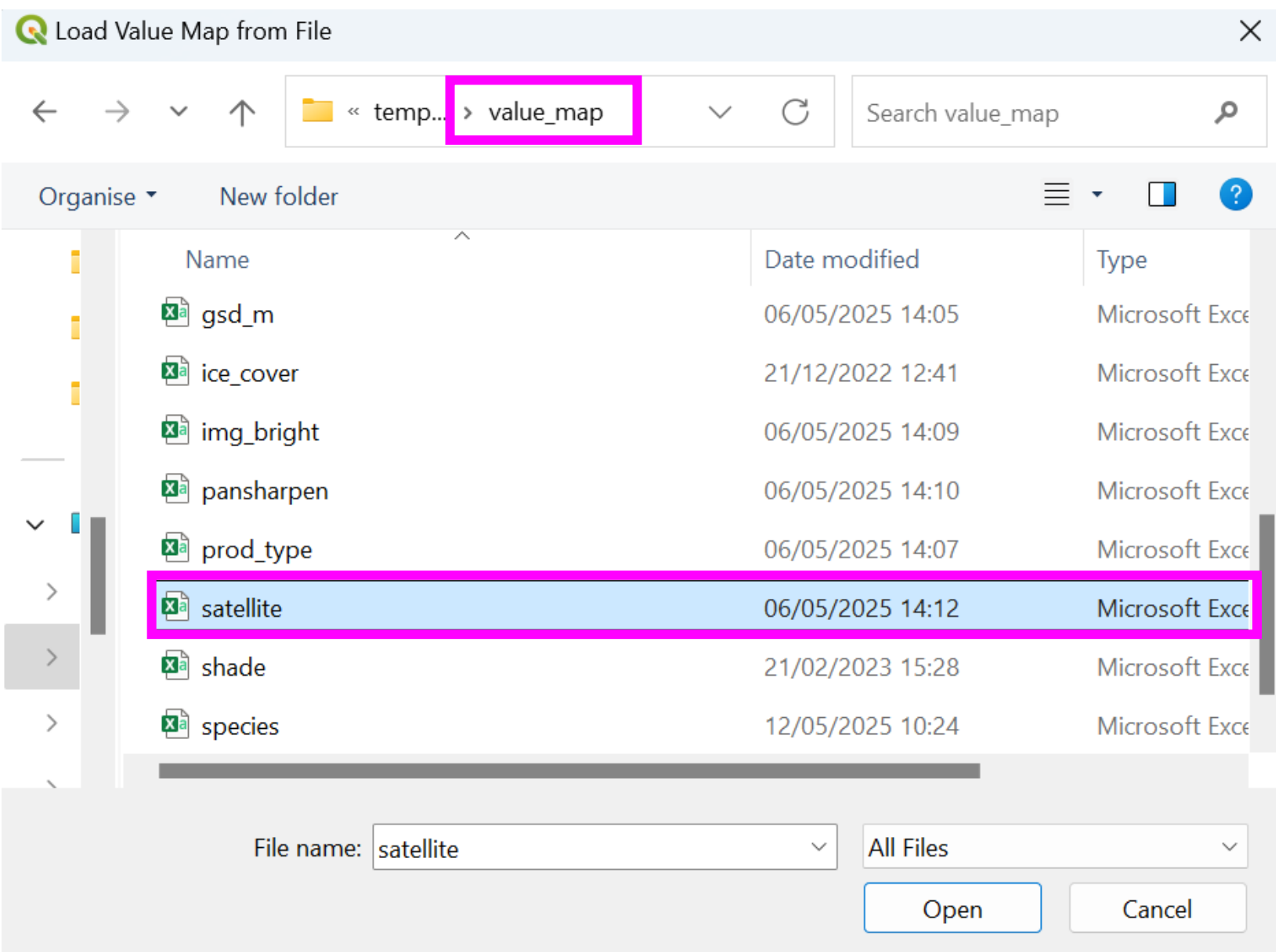

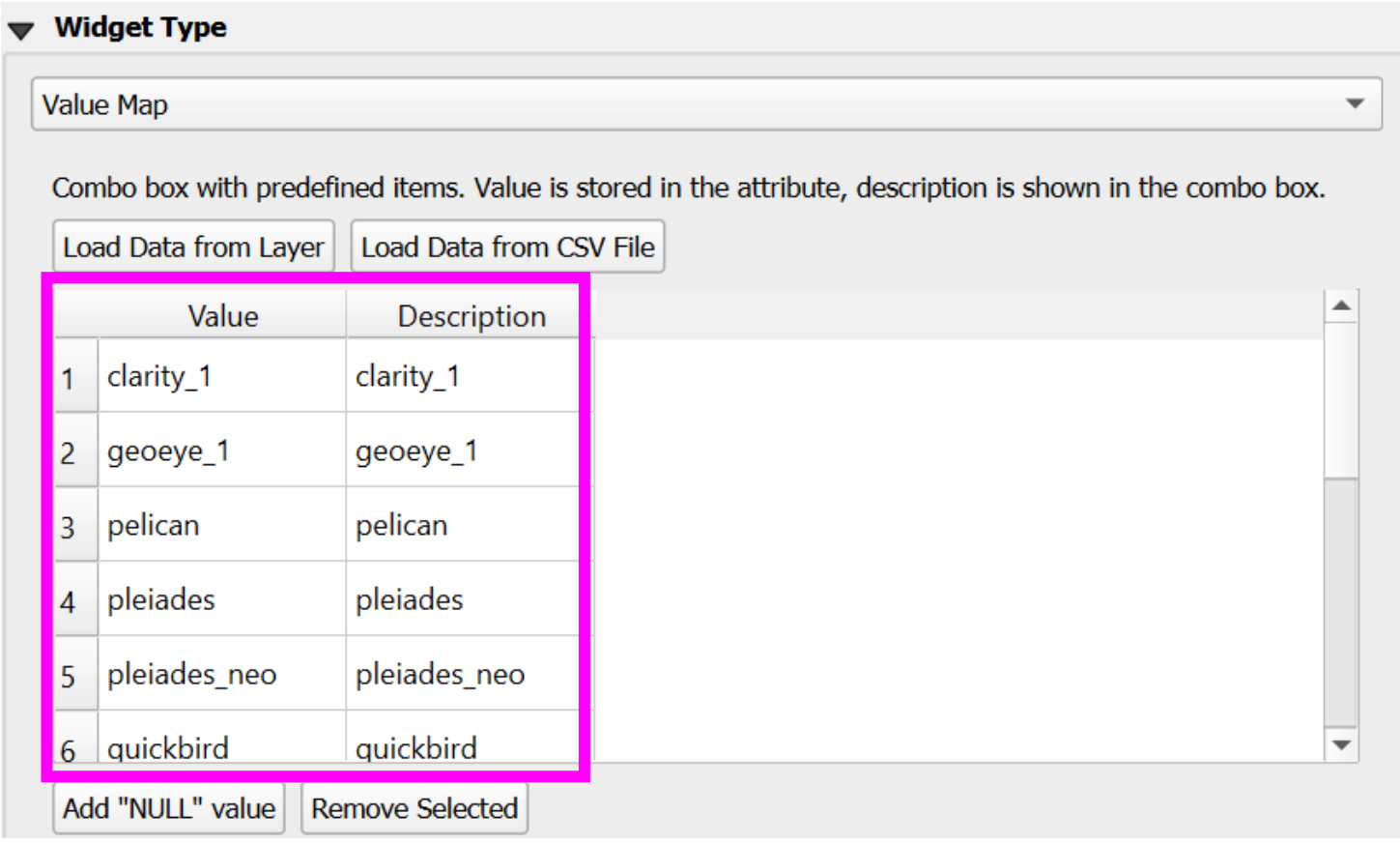

Table S1.A2.1: Properties per attribute of the proposed data standard/template shapefile for the strandings from space community.

Attributes Form Fields	Editable	Reuse last entered value	Widget Type	Load Data from CSV File	Comments
id	✓	-	Text Edit	-	To populate automatically see guidance and cautions detailed above.
observer	✓	✓	Value Map	-	User to manually add drop-down entries (observer name or number) for a given project.
location	✓	✓	Text Edit	-	
satellite	✓	✓	Value Map	✓	
img_cat_id	✓	✓	Text Edit	-	
img_date	✓	✓	Text Edit	-	
img_time	✓	✓	Text Edit	-	
gsd_m	✓	✓	Value Map	✓	
prod_type	✓	✓	Value Map	✓	
projection	✓	✓	Text Edit	-	
pansharpen	✓	✓	Value Map	✓	
bands	✓	✓	Value Map	✓	
feature	✓	-	Value Map	✓	
certainty	✓	-	Value Map	✓	
deco_phase	✓	-	Value Map	✓	
species	✓	-	Value Map	✓	
colour	✓	-	Value Map	✓	
body_shape	✓	-	Value Map	✓	
length_m	✓	-	Text Edit	-	
width_m	✓	-	Text Edit	-	

flippers	✓	-	Value Map	✓	For ‘Value Map’ use ‘flippers_fluke_slick_bld’ .csv
fluke	✓	-	Value Map	✓	For ‘Value Map’ use ‘flippers_fluke_slick_bld’ .csv
sick_bld	✓	-	Value Map	✓	For ‘Value Map’ use ‘flippers_fluke_slick_bld’ .csv
group	✓	-	Value Map	✓	
cloud_cov	✓	-	Value Map	✓	
cloud_thic	✓	-	Value Map	✓	
env	✓	-	Value Map	✓	
img_bright	✓	-	Value Map	✓	
shade	✓	-	Value Map	✓	
ice_cover	✓	-	Value Map	✓	
comments	✓	-	Text Edit	-	A space for any further information, this field can be left blank

References

Bayir, I. 'IKONOS collection capability with the direct tasking from a regional operation center', *International Conference on Recent Advances in Space Technologies, 2003. RAST '03. Proceedings of.* Istanbul, Turkey. pp. 30–33. Available at: https://doi.org/10.1109/RAST.2003.1303386

Campbell, J.B. and Wynne, R.H. (2011) *Introduction to Remote Sensing.* 5th edn. The Guilford Press, p. 669.

Clarke, P.J., Cubaynes, H.C., Stockin, K.A., Olavarría, C., de Vos, A., Fretwell, P.T. and Jackson, J.A. (2021) 'Cetacean Strandings From Space: Challenges and Opportunities of Very High Resolution Satellites for the Remote Monitoring of Cetacean Mass Strandings', *Frontiers in Marine Science,* 8, article 650735. Available at: https://doi.org/10.3389/fmars.2021.650735

Cubaynes, H.C., Clarke, P.J., Goetz, K.T., Aldrich, T., Fretwell, P.T., Leonard, K.E. and Khan, C.B. (2023) 'Annotating very high-resolution satellite imagery: A whale case study', *MethodsX,* 10, article 102040. Available at: https://doi.org/10.1016/j.mex.2023.102040

Cubaynes, H.C., Fretwell, P.F., Bamford, C., Gerrish, L. and Jackson, J.A. (2019) 'Whales from space: Four mysticete species described using new VHR satellite imagery', *Marine Mammal Science,* 35(2), pp. 466-491. Available at: https://doi.org/10.1111/mms.12544

Dowman, I., Jacobsen, K., Konecny, G. and Sandau, R. (2012) *High Resolution Optical Satellite Imagery.* 1st edn. Whittles Publishing, p. 230.

Fretwell, P.T., Jackson, J.A., Ulloa Encina, M.J., Häussermann, V., Perez Alvarez, M.J., Olavarría, C. and Gutstein, C.S. (2019) 'Using remote sensing to detect whale strandings in remote areas: The case of sei whales mass mortality in Chilean Patagonia', *PLoS ONE,* 14(11), article e0225209. Available at: https://doi.org/10.1371/journal.pone.0222498

Geraci, J.R. and Loundsbury, V.J. (2005) *Marine Mammal Ashore: A field guide for strandings.* 2nd edn. National Aquarium in Baltimore, Inc.

Humphries, G.R.W., Magness, D.R. and Huettmann, F. (2018) *Machine Learning for Ecology and Sustainable Natural Resource Managment.* Springer.

Khan, C.B., Goetz, K.T., Cubaynes, H.C., Robinson, C., Murnane, E., Aldrich, T., Sackett, M., Clarke, P.J., LaRue, M.A., White, T., Leonard, K., Ortiz, A. and Lavista Ferres, J.M. (2023) 'A Biologist Guide

to the Galaxy: Leveraging Artificial Intelligence and Very High-Resolution Satellite Imagery to Monitor Marine Mammals from Space', *Journal of Marine Science and Engineering,* 11(3), article 595. Available at: https://doi.org/10.3390/jmse11030595

LaRue, M.A., Stapleton, S. and Anderson, M. (2017) 'Feasibility of using high-resolution satellite imagery to assess vertebrate wildlife populations', *Conservation Biology,* 31(1), pp. 213-220. Available at: https://doi.org/10.1111/cobi.12809

Liang, S. and Wang, J. (2019) *Advanced Remote Sensing: Terrestrial Information Extraction and Applications.* 2nd edn. Academic Press, p. 1010.

Mazzariol, S., Siebert, U., Scheinin, A., Deaville, R., Brownlow, A., Uhart, M., Marcondes, M., Hernandez, G., Stimmelmayr, R., Rowles, T., Moore, K., Gulland, F., Meyer, M., Grover, D., Lindsay, P., Chansue, N. and Stockin, K. (2020) *Summary of Unusual Cetacean Stranding Events worldwide (2018-2020)*. Paper SC/68B/E/09 presented to the IWC Scientific Committee (Unpublished) 10. Available at: https://archive.iwc.int/pages/view.php?search=%21collection29937+&k=&modal=&display=list&order_by=title&offset=0&per_page=240&archive=&sort=DESC&restypes=&recentdaylimit=&foredit=&ref=17280 (Accessed: May 15, 2020).

Moore, K.M., Simeone, C.A. and Brownell Jr., R.L. (2018) 'Strandings', in B. Würsig, Thewissen J. G. M., Kovacs, K. M. (ed.) *Encyclopedia of marine mammals.* 3rd edn. Academic Press, Elsevier Inc, pp. 945-951.

Rees, W.G. (2013) *Physical Principles of Remote Sensing.* 3rd edn. Cambridge University Press, p. 442.
