# Supplementary material for: Open-source satellite image pre-processing and annotation workflows: Stranded whale and dolphin case study
Source: MethodsX. 2026 May 14;17:103949. doi: 10.1016/j.mex.2026.103949 (PMC13586820; doi:10.1016/j.mex.2026.103949)
Supplement: Supplementary file 2 [file mmc3.pdf]

S3: Training guide (descriptive and visual) to the standardised annotation attributes for identifying stranded cetaceans in satellite imagery

The following training guide aims to aid the strandings community to assess the certainty of stranded cetaceans detected in VHR optical satellite imagery. The document provides (1) a descriptive guide and (2) a visual guide for the standardised (required and optional) attributes for data annotation. The guide should be viewed by observers ahead of reviewing VHR satellite imagery for stranded cetaceans, to familiarise themselves with examples of stranded cetaceans and confounding features.

As satellite imagery is a novel tool for the strandings community, the standardised values for annotating features have been developed based on existing knowledge of strandings and the current state of the field. This template is a baseline and intended to develop with the field. Users can add, amend or remove values as needed. New proposed values should use lower case and '_' instead of space for compatibility with coding languages.

Where satellite imagery is used for known stranding events, the correct definition of a stranding event (between, mass stranding, mass mortality event and unusual mortality events) should be used in publication. In cases where satellite imagery cannot be validated with ground data, species are unknown, or it is not possible to ascertain both the temporal and spatial scale of the event, we recommend these events are referred to as 'a spatial cluster of strandings' (pers comms. Dr Andrew Brownlow, Chair of the International Whaling Commission Stranding Expert Panel).

1. Descriptive guide

The descriptive guide provides a definition of each attribute and its associated values in the standardised metadata template, proposed to the strandings community when assessing VHR optical satellite imagery for stranded cetaceans.

Recommended file naming convention for sharing exported annotation metadata .csv files: 'location_satellite_image-catalog-id_image-date_image-time_spatial-resolution_observer.shp'. All entries should use lower case and '_' instead of space for compatibility with coding languages.

a. Required attributes

Table S3.1: List of required attributes associated with each strandings from space annotation. Required attributes are those considered essential to include in published datasets as a minimum, as agreed by 'Satellites to study live and stranded cetaceans: Establishing best practice' workshop, Society of Marine Mammalogy Conference, 10 November 2024. Most attribute values are standardised drop-down menus with exception of 'id', 'observer', 'location', 'img_cat_id', 'img_date', 'img_time', these are user defined, and 'longitude' and 'latitude', which are calculated post-annotation.

Attribute	Description	Unit	Format	Value
**id**	A unique numerical value assigned to each annotation (marked point).	None	Integer	*e.g., 0, 1, 2, 3…*
**observer**	Name of person reviewing the image (user to define the names of each	None	String	*e.g.,* penny_clarke<br>*or*<br>*1*

Attribute	Description	Unit	Format	Value
	person in the team of observers for a given project. Numerical values can be assigned to preserve observer anonymity).			
**location**	Name of the location where the satellite image was captured (user to define, use lower case and '_' instead of space for compatibility with coding languages)	None	String	*e.g.,* golfo_de_penas
**satellite**	Name of the satellite that captured the image ('satellite' is a list of commercial VHR satellites (specifically those, which are internationally accessible), including those decommissioned, currently in orbit, or projected for launch in the near future).	None	String	clarity_1<br>geoeye_1<br>pelican<br>pleiades<br>pleiades_neo<br>quickbird<br>skysat<br>worldview_1<br>worldview_2<br>worldview_3<br>worldview_4<br>worldview_legion
**img_cat_id**	Unique identification that the satellite imagery provider assigns to each image. With Vantor, this corresponds to the catalog ID.	None	String	*e.g.,* 10400ED2959
**img_date**	Date the image was captured (UTC).	Year, month, day	String	YYYY-MM-DD

Attribute	Description	Unit	Format	Value
		(ISO8601 format)		
**img_time**	Time the image was captured (UTC).	Hours and minutes (ISO8601 format)	String	hh:mm:ssZ
**gsd_m**	The ground sampling distance (is the distance on the ground represented by a single pixel in a satellite image), which in QGIS can be found in the metadata, by right clicking on the panchromatic file and selecting ‘Properties’, then ‘Information’ and ‘Pixel Size’ or else by viewing the .imd or .xml file.	meter	Double	0.1<br>0.15<br>0.2<br>0.3<br>0.31<br>0.4<br>0.46<br>0.5<br>0.6<br>0.65<br>0.7<br>0.75<br>0.8<br>0.9<br>1
**prod_type**	The product type indicates the level of pre-processing applied to an image. ‘prod_type’ is a list of commercial VHR satellites operators, specifically those, which are internationally accessible, and their available product types.	None	String	analysis_ready<br>map-ready_ortho_1_12000<br>map-ready_ortho_3d<br>ortho<br>photogrammetry_ready<br>primary<br>projected<br>skysat_basic_scene<br>skysat_ortho_collect<br>skysat_ortho_scene<br>system-ready_basic_1b<br>system- stereo_basic_1b<br>view_ready<br>view-ready_standard_2a<br>view-ready_standard_or2a

Attribute	Description	Unit	Format	Value
				view-ready_stereo_standard_or2a
**projection**	Projection applied to the image to remove distortion (Universal Transverse Mercator (UTM), is a series of grid zones of the world, a plane coordinate grid and can be determined for an image using https://www.dmap.co.uk/utmworld.htm.	None	String	*e.g.,* epsg_32718_wgs84_utm_zone_18s
**pansharpen**	The algorithm used to perform pansharpening of an image (format includes available algorithms in QGIS OrfeoToolbox (OTB) and GDAL. For example, OTB algorithms are either 'rcs', 'lmvm', or 'bayes', and interpolation is either 'bco', 'nn' or 'linear') (user defined alternatives can be added to value mapping where necessary).	None	String	gdal_average<br>gdal_bilinear<br>gdal_cubic<br>gdal_cubic_spline<br>gdal_lanczos_windowed_sinc<br>gdal_nearest_neighbour<br>otb_bayes_bco<br>otb_bayes_linear<br>otb_bayes_nn<br>otb_lmvm_bco<br>otb_lmvm_linear<br>otb_lmvm_nn<br>otb_rcs_bco<br>otb_rcs_linear<br>otb_rcs_nn
**bands**	The combination of bands used to visualise an image (spectral bands are the ranges of wavelengths of light	None	String	1,2,3<br>3,2,1<br>4,3,1<br>4,3,2<br>5,3,2<br>6,4,3

Attribute	Description	Unit	Format	Value
	that are captured by a sensor, assigned to bands). Combinations of bands can alter image visualisation. To visualise as the eye would expect, typical band combinations for Vantor imagery are R = 3, G = 2, B = 1 (four band image) and R = 5, G = 3, B = 2 (eight band image). The band order in the 'Value' column is RGB. RGB refers to the QGIS display settings, while the number refers to the specific band of the multispectral sensor.			
**feature**	The type of feature a detection is. This includes the ability to annotate confounding features, which can be useful in creating multi-class training datasets (see definitions below).	None	String	stranded_cetacean<br>floating_carcass<br>live_cetacean<br>boat<br>marine_litter<br>organic_beach_debris<br>rock
**certainty**	Certainty of an observer that a detection is the assigned feature (species or higher taxonomic level)	None	String	definite_90-100<br>likely_70-89<br>possible_50-69

Attribute	Description	Unit	Format	Value
	**Definite**: 90-100% confidence a detection is the defined feature (almost no doubt) **Likely**: 70-89% confidence a detection is the defined feature (high likelihood but not sure, for example, when in a mass it is challenging to determine individuals, however based on morphology and size you assign as two features, but you are not sure) **Possible**: 50-69% confidence a detection is the defined feature (hard to tell, perhaps because there is marine debris on the beach with a similar appearance or an individual is far from the mass but has a similar appearance).			
**longitude**	Longitude of the 'stranded_cetacean' or 'floating_carcass'.	Decimal degree	Double	*e.g.,* 12.70
**latitude**	Latitude of the 'stranded_cetacean' or 'floating_carcass'.	Decimal degree	Double	*e.g.,* 67.50

Feature ID (id)

The feature ‘id’ is a unique numerical value assigned to each marked point. It is recommended this value is entered manually for each new feature, starting at 0, assign each new feature an incremental numerical value, for example, 1, 2, 3, 4, etc. The tool used to export image chips in the final step of the process, numbers from zero, and therefore, will correspond with the original feature ‘id’ assigned. There is an option to make this field populate automatically with the next incremental value, however, this is not recommended, as anytime a change is made this results in automatic reassignment to a new ‘id’ value (for example moving a point’s position).

Image Observer (observer)

This field is project specific, allowing the user to define the ‘Value’ and ‘Description’ with the names of each person in the team of observers for a given project. Ensure to use lower case and hyphens (_) instead of space, for example, penny_clarke. Alternatively, observers can be assigned a numeric value instead of a name for anonymity. Guidance on setting up dropdown entries with observer’s names or auto filling an attribute table column with a given name can be found in the ‘S1_QGIS_Strandings_from_Space_Pipeline’ document.

Image location (location)

Location is a user defined field to detail the name of the location where the satellite image was captured (lower case and ‘_’ instead of space for compatibility with coding languages). The chosen name should be descriptive so that another user could identify the general location the image was collected.

The following attributes associated with information of the satellite image can be found in the satellite image metadata file. N**avigate to the panchromatic or multispectral image folder in your directory, double click the .XML or .IMD file in the folder to open and view the image metadata. Precise details with how to locate the information within the metadata file can be found in the ‘S1_QGIS_Strandings_from_Space_Pipeline’ document.**

Satellite sensor (satellite)

Satellite is a list of the VHR optical satellites sensors for the main commercial operators, Airbus, Albedo, Vantor (formerly Maxar Technologies Ltd), and Planet. Users should select the satellite sensor used to capture the image being reviewed. The list includes decommissioned sensors, those currently in orbit, or projected for launch in the near future.

Image catalogue ID (img_cat_id)

Image catalogue ID is a user defined field that should correspond to the unique identification number or series of letters and numbers that the satellite imagery provider assigns to each image. Ensure to enter the ID precisely to include any instances where an ID contains text separated by underscores, hyphens, or dashes.

Image collection date (img_date)

The image collection date is a user defined field detailing the date image was captured. The date should be recorded as year, month, day (YYYYMMDD) coordinated universal time (UTC, worldwide standard to ensure timekeeping), in accordance with the ISO8601 format.

Image collection time (img_time)

The image collection time is a user defined field detailing the time image was captured. The time should be recorded as hours, minutes, and seconds (hh:mm:ssZ) UTC, in accordance with the ISO8601 format

('T' is removed from the beginning of Thh:mm:ss as per ISO8601 guidelines, as this is not necessary to include unless for separation of date and time when communicated together).

Ground sampling distance (gsd_m)

The ground sampling distance is the distance on the ground represented by a single pixel in a satellite image. The distance is given in meters ('gsd_m', where 'm' denotes the unit metres) to ensure that users could apply this standard to annotate more coarse resolution imagery exceeding 1 m spatial resolution, using the same unit. Values in the image metadata may be given in scientific notation, where 5.000000000000000e-01 is equivalent to 0.5 m or 50 cm.

Product Type (prod_type)

Product Type is a list of product types for the main VHR satellite imagery providers. Satellite image companies can provide images at differing levels of pre-processing, which are defined by the user at the time of ordering imagery. Pre-processing can include, projecting, pansharpening, orthorectification, and atmospheric correction of imagery. Users should select the product type associated with an image, by the image provider who supply the imagery (see Table 1 in the associated manuscript).

Projection (projection)

Projection is a user defined entry to denote the projection applied to the image to remove distortion. When a satellite image is collected, the image is a flat representation of a spherical Earth, and the image may therefore appear distorted. By assigning a coordinate system that is most relevant to the image area through projection / re-projection, the image representation will show a more accurate flat two-dimensional representation of the Earth. A projection is a mathematical transformation of a coordinate system. If the image is projected it will be detailed within the image metadata, else for images without a projection, users can assign the most appropriate projection. Universal Transverse Mercator (UTM), is a series of grid zones of the world, a plane coordinate grid and can be determined for an image using https://www.dmap.co.uk/utmworld.htm (where images overlap zones, best practice would be to choose the UTM zone containing the majority of image). To comply with coding languages entries should be made as all lowercase with hyphens used instead of space, for example, 'epsg_32718_wgs84_utm_zone_18s'.

Pansharpening algorithm (pansharpen)

Pansharpening algorithm is a list of algorithms available in QGIS that can be applied to pansharpen an image. A panchromatic image will have a higher spatial resolution than a multispectral image, as it captures a wider range of light in a single band, allowing it to be significantly sharper. For example, Vantor's WorldView-3 sensor, produces a panchromatic image with a spatial resolution of 0.3 m, and a multispectral image with a spatial resolution of 1.24 m. To analyse a multispectral image, it can be useful to enhance its spatial resolution to the higher spatial resolution of the panchromatic image, through the process of pansharpening. Pansharpening takes the panchromatic and multispectral images, and where the two rasters fully overlap, fuses them together to produce a multispectral image with the higher spatial resolution of the panchromatic image. Pansharpening is useful to studying cetacean strandings from space as both spectral and spatial richness is needed to discriminate individual features with confidence. The list details possible algorithms in QGIS using a plugin called Orfeo Toolbox (OTB) (BundleToPerfectSensor) and GDAL. Both tools offer a series of algorithms to pansharpen imagery, for example, one of OTB's algorithm options is 'bayes' algorithm with 'bicubic interpolation', if users applied this algorithm, they would select 'otb-bayes-bco'.

Band combination (bands)

Band combination is a series of common bands combinations used to review imagery, *e.g.*, RGB (red, green, and blue, also known as true colour) or false colour composite. The band combinations to

visualise multispectral imagery can be ascertained by reviewing the satellite sensor specification, either online from the satellite image provider, or within the image metadata file. Typically for Vantor imagery, to view the imagery as our eye would naturally see (true colour), four band imagery is R = 3, G = 2, B = 1 and eight band imagery is R = 5, G = 3, B = 2. RGB here is referring to the QGIS display settings and the number refers to the specific bands of the multispectral sensor. For example, the band combination R = 6, G = 4, B = 3 (in eight band imagery) is a false colour composite that helps to visualise the red-edge of the electromagnetic spectrum. This can be a powerful band combination for strandings monitoring, to discriminate stranded whales from confounding features, as stranded whales appear spectrally unique, when compared with the red of vegetation (Fretwell *et al.*, 2019).

Feature (feature)

Feature is a category assigned to each marked point to describe what a feature is, the categories are:

1) stranded_cetacean: a live or dead cetacean located onshore. A stranding is a cetacean that falters ashore, debilitated, or is in an environment incompatible with its natural survival (*e.g.*, washed ashore on a beach); if the cessation of life has occurred prior to deposition, the cetacean is considered beachcast (Clarke *et al.*, 2021). As it is not possible to determine from space whether a feature washed ashore is alive or dead (beachcast) at the time of stranding, all features considered stranded should be assigned stranded_cetacean. Partially beached animals within the surf zone should be defined, stranded_cetacean.

2) floating_carcass: cetaceans that die at sea (or those who have stranded and since been washed offshore) may float due to decomposition gasses keeping the carcass at the surface. In these instances, a dead cetacean located in the near shore zone (<1 km from shore) or offshore (>1 km from shore) in the open ocean zone is considered a ‘floating_carcass’.

3) live_cetacean: a live (free-swimming) cetacean. While reviewing imagery for stranded cetaceans it is possible that open water may be visible within the imagery and live (free-swimming) cetaceans may be detected, these can be annotated as a basic annotation to denote the location, for later incorporation into live cetacean datasets using Cubaynes *et al.* (2023) recommended attributes.

4) boat: small or large boats located on the shore, or in the near shore and offshore zones if identifying ‘floating_carcass’ and ‘live_cetacean’.

5) marine_litter: any item not naturally found in the marine environment, for example, plastic and lost, discarded, abandoned or ghost fishing gear, which can exhibit a similar appearance to a stranded cetacean feature from space.

6) organic_beach_debris: sometimes termed wrack/strandline debris/organic marine debris and includes seaweed, algae, seagrass and woody materials such as tree trunks, branches and bark, which may have a similar appearance to a stranded cetacean feature from space.

8) rock: rock that has a similar appearance to a stranded cetacean feature.

We recommend annotating confounding features, features that are visually like a cetacean, for example, algae, trees, boats, and rocks. Confounding features should only be assigned when there is certainty. By annotating confounding features, these annotations could be incorporated into training datasets for multi-class machine learning models, to better train models at detecting stranded cetaceans correctly.

Certainty (certainty)

Certainty is a percentage confidence assigned based on each individual observer’s interpretation and evaluation of a feature. For example, how confident an individual observer is that a feature is for example, a stranded_cetacean.

Certainty can be defined within three categories:

definite_90-100

likely_70-89

possible_50-69

Assigning a level of certainty is subjective and should be based on the observer's best knowledge. To help, the images in the visual guide provide examples of what a definite cetacean stranding feature and confounding features look like within satellite imagery (including fluke, slick, or group presence, which can increase the confidence in assigning certainty). Examples are provided from multiple satellite sensors with differing spatial resolution, supplemented with aerial or ground imagery, where possible.

In the initial phases of annotating features using this novel method, stranded cetaceans may be assigned a lower certainty, for example, 'possible_50-69', particularly due to the heterogeneity of cetacean strandings as they decompose. With greater examples, our understanding and interpretation of features and confidence in assigning a certainty level, will increase. In these early stages, confidence in assigning certainty can be elevated from possible to probable or definite with ground truth data / images of known events. Hence the importance of testing this technology with pilot studies in known locations of strandings with available data on the ground, before testing in more remote locations without prior knowledge available. For example, information from a stranding network verifying the location of a stranding, can offer an observer greater confidence in assigning a feature.

Figure S3.1, provides an example of evaluating the certainty of a feature. The definite cetacean has been assigned definite, even in absence of ground truth data, owing to the whale like shape, size and visible fluke. The probable whale has no visible cetacean defining features like a fluke or flippers, though is within the expected size range. You may consider it looks similar to the possible feature, however, in this instance the feature was assigned probable as the feature was in the location of a known carcass detailed from ground data. Thus, demonstrating the importance of ground data in these early stages to increase the confidence in assigning a certainty value.

definite_90-100	likely_70-89	possible_50-69

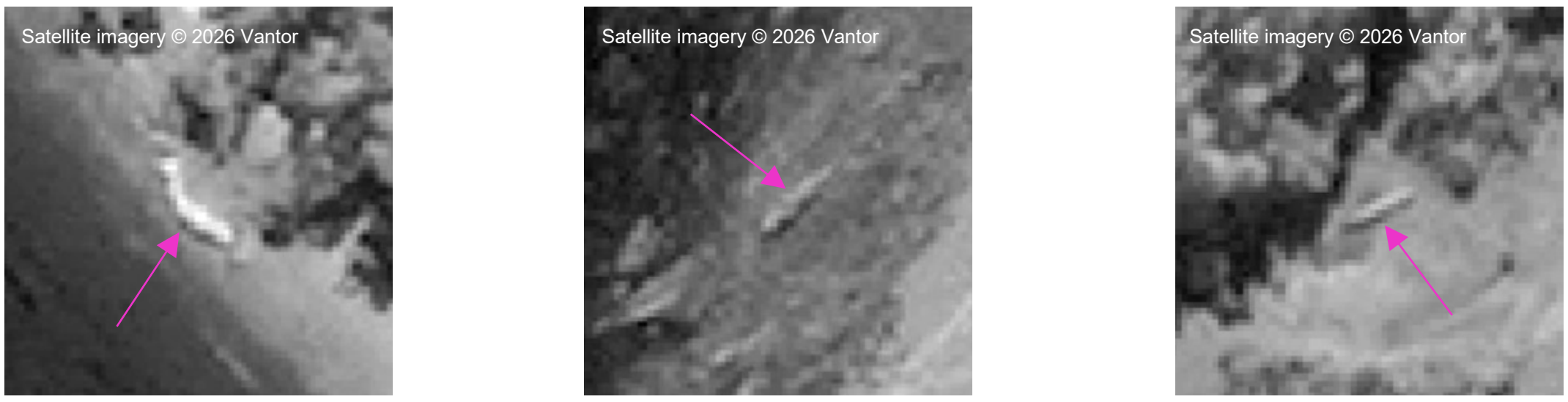

Figure S3.1: An example of a definite_90-100, likely_70-89 and possible_50-69 feature, satellite imagery © 2026 Vantor.

Longitude (longitude)

Once all features are annotated, the longitude of the 'stranded_cetacean' or 'floating_carcass' can be extracted. For consistency and ease of comparison the recommended format is decimal degrees. Projected coordinate systems units, with a known zone and datum, are typically linear measurements, for example, meters, and will need to be transformed. Geographic coordinate systems units are typically in decimal degrees and will not require transforming. See the

‘S1_QGIS_Strandings_from_Space_Pipeline’ document for guidance on how to extract longitude using the attribute table field calculator.

Latitude (latitude)

Once all features are annotated, the latitude of the ‘stranded_cetacean’ or ‘floating_carcass’ can be extracted. For consistency and ease of comparison the recommended format is decimal degrees. Projected coordinate systems units, with a known zone and datum, are typically linear measurements, for example, meters, and will need to be transformed. Geographic coordinate systems units are typically in decimal degrees and will not require transforming. See the ‘S1_QGIS_Strandings_from_Space_Pipeline’ document for guidance on how to extract latitude using the attribute table field calculator.

b. Optional attributes

Table S3.2: List of optional attributes associated with each strandings from space annotation, as agreed by ‘Satellites to study live and stranded cetaceans: Establishing best practice’ workshop, Society of Marine Mammalogy Conference, 10 November 2024. Most attribute values are standardised drop-down menus with exception of ‘length_m’, ‘width_m’, and ‘comments’, these are user defined. Details of environmental variables represent the conditions at the point feature/annotation specifically, and not the whole image.

Attribute	Description	Unit	Format	Value
**deco_phase**	The level of decomposition of a ‘stranded_cetacean’ or ‘floating_carcass’ (see definitions below).	None	String	fresh_alive<br>fresh_dead<br>bloating_and_floating<br>active_decomposition<br>passive_decomposition<br>dry_remains<br>unknown
**species**	The species or higher taxonomic level of the feature detection (*e.g.,* ‘sei_whale’), else when unknown assign higher taxonomic level (*e.g.,* ‘large_whale’ or ‘unidentified_whale’). The list of species uses the Society of Marine Mammalogy (SMM) (Committee on Taxonomy, 2025) naming conventions,	None	String	*e.g.,*<br>unidentified_dolphin<br>unidentified_large_baleen_whale<br>unidentified_large_whale<br>unidentified_porpoise<br>unidentified_small_whale<br>unidentified_whale<br>(… remaining list is the SMM naming conventions for all species *e.g.,* bowhead_whale)

Attribute	Description	Unit	Format	Value
	to ensure consistent terminology throughout the marine mammal community, with addition of instances where species is unknown.			
**colour**	The most dominant colour of the detected cetacean. Colour should be assigned when the feature is viewed in true colour (red, green, blue bands). During decomposition the ‘stranded_cetacean’ or ‘floating_carcass’ colour may be a mixture of colours, assign the most dominant by percentage cover.	None	String	black<br>brown<br>cream<br>dark_grey<br>light_grey<br>orange<br>pink<br>red<br>white
**body_shape**	Overall shape of the body excluding fluke and flippers. Select the most suited or ‘undefinable’, especially in late stages of decomposition.	None	String	ellipsoid<br>streamline<br>irregular_ellipsoid<br>irregular_streamline<br>undefinable
**length_m**	Maximum visible length between the tip of the head and the fluke with values ranging from calf size	Meters	Double	*e.g.,* 12.4

Attribute	Description	Unit	Format	Value
	to maximum adult length.			
**width_m**	Maximum visible width measured at the widest part of the body and perpendicular to the body length.	Meters	Double	*e.g.,* 2.0
**flippers**	Are the flippers (forelimb used to turn, break and stabilise a cetacean while swimming) visible?	None	String	yes<br>no<br>maybe
**fluke**	Is the fluke (tail used to propel a cetacean forward) visible?	None	String	yes<br>no<br>maybe
**slick_bld**	Is a slick or blood visible? A slick is a waxy grey liquid released when a cetacean decomposes (the oil contained within cetacean blubber gets converted to adiopocere, a wax like substance formed when body lipid-tissues break down). It can cover wide areas around the remains. This can temporarily prohibit the growth of nearby plants, so reduced or dead vegetation nearby and grey colouration	None	String	yes<br>no<br>maybe

Attribute	Description	Unit	Format	Value
	surrounding a carcass may be seen from space.			
**group**	The number of 'stranded_cetacean' or 'floating_carcass' within 1 km of a detected feature. This should be per annotation and can be an estimate, especially when in a mass.	None	String	Individual<br>2_to_5<br>6_to_10<br>11_to_50<br>51_to_100<br>101_to_200<br>201_to_500<br>501+
**cloud_cov**	Cloud cover for the area within the vicinity of the detection, using the aviation system:<br>'skc_sky_clear' = 0/8th<br>'few_traces' = 1-2/8th<br>'sct_scattered' = 3-4/8th<br>'bkn_broken' = 5-7/8th<br>'ovc_overcast' = 8/8th	Okta	String	skc_sky_clear<br>few_traces<br>sct_scattered<br>bkn_broken<br>ovc_overcast
**cloud_thic**	Cloud thickness for the clouds present within the vicinity of the detection:<br>Thin (can see fairly well through the clouds)<br>Medium thin (can see through but no clear view of the sea)	None	String	none<br>thin<br>medium_thin<br>thick

Attribute	Description	Unit	Format	Value
	Thick (cannot see through)			
**env**	The abiotic environment that a detection is found in (nearshore waters are < 1 km and offshore waters are > 1 km from shore).	None	String	bedrock<br>cobble_beach<br>estuary_mud_flat<br>gravel_beach<br>man_made_structure<br>mangrove<br>marsh_brackish<br>marsh_freshwater<br>nearshore_waters<br>offshore_waters<br>sand_beach_dark<br>sand_beach_light<br>shell_beach<br>vegetated_trees<br>vegetated_wetland
**img_bright**	The brightness of the image within the vicinity of the detection.	None	String	bright<br>moderate<br>dark
**shade**	The level of shade from the sun, full, partial or none at the point of the detection.	None	String	full_shade<br>partial_shade<br>no_shade
**ice_cover**	Relevant to ice covered regions only, the level of ice cover at the point of the detection.	None	String	none<br>low<br>medium<br>dense<br>very-dense
**comments**	Any other comment the observer would like to make about the specific detection.	None	String	*e.g.,* Could be dry remains of carcass or wood from a tree

Phase of Decomposition (deco_phase)

Upon stranding, a cetacean unless recovered will undergo decomposition, and the level of decomposition can be attributed to a phase as it progresses. Stranding networks working on the ground

have well established decomposition levels definitions used in their response programmes and records. Standardisation in use of terminology between stranding network experts working on the ground and remote sensing experts studying strandings from space, is essential to working collaboratively and cross-disciplinary. Ideally, decomposition level definitions used by stranding networks would be incorporated into space applications. However, stranding network ground-based definitions, for example, for the United Kingdom (UK) criteria (Law *et al.*, 2006), are driven by details that require close observation of an animal, and are more driven by pathology and the internal structure and integrity of the organs. As strandings detection from space uses remote monitoring with limited detail, the definitions for phases of decomposition need to focus more on externa. As such we propose standardised phases of decomposition definitions established through time-lapse imagery as part of the largest mass mortality event of baleen whales in the world, in the Chilean Patagonia, 2015 (pers. comms. McConnell, K., 2025, informed by Payne (1965) and Mądra *et al.* (2015)). For each decomposition phase definition, similarity to stranding response team definitions from the UK are detailed where appropriate, and in addition, comparison and incorporation of the decomposition condition categories (DCC) from the Agreement for the Conservation of Cetaceans of the Baltic Sea, Mediterranean Sea and Contiguous Atlantic Area (ACCOBAMS) and Agreement on the Conservation of Small Cetaceans of the Baltic, North East Atlantic, Irish and North Seas (ASCOBANS) best practice on cetacean post mortem (IJsseldijk, Brownlow and Mazzariol, 2019). Regional differences in timescales for each phase of decomposition may apply, for example, tropical / warmer climates will experience faster rates of decomposition when compared with temperate and again cooler polar climates. In most instances these phases of decomposition may not be attributable without ground truth, knowledge of the timing of the event (stranding network reports) and local environment etc. **In these initial stages of satellites application to study strandings, phases of decomposition should only be assigned where ground truth data is available**. If one of the following phases of decomposition cannot be assigned with confidence, then assign as ‘unknown’. It is unlikely ‘fresh_alive’ will be captured by satellites at present, as current technological advancements do not support emergency response to live mass strandings, due to time delays in image collection (Clarke *et al.*, 2021), though as technology improves this could become possible:

1) fresh_alive: stranded alive (UK: condition code 1, DCC code 1).

2) fresh_dead: Normal appearance, carcass not bloated, tongue and penis not protruded; blubber white and occasionally tinged with blood. Typically, during the first 24-48 hours after death, decomposition processes immediately begin acting on and, in the carcass, (timings may vary across the globe and local knowledge should be applied to a region) (UK: condition code 2a, DCC code 1 & 2, Figure S3.2).

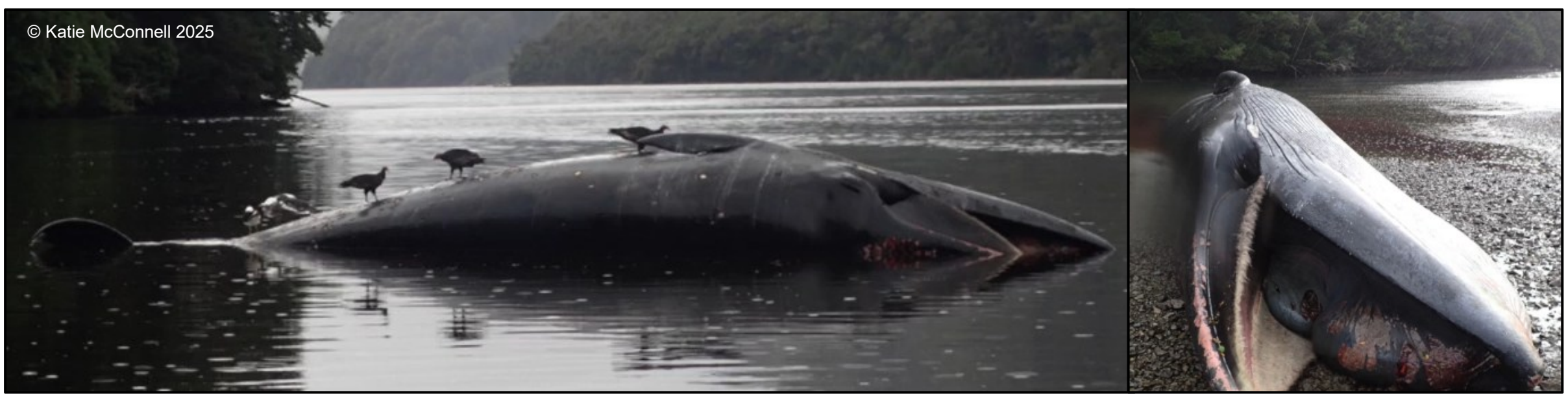

Figure S3.2: ‘fresh_dead’ sei whale (Balaenoptera_borealis), exhibiting normal appearance and no bloating, captured in the Golfo de Penas, Chile, © Katie McConnell 2025

3) bloating_and_floating: Bloating evident, with tongue and penis often distended; skin may be visibly cracked and blubber is blood-tinged and oily. Fermentation of the internal organs causes the whale carcass to swell and float during high tides. Floating carcasses can be transported great distances and so may be found offshore (UK: condition code 2b / 3, DCC code 3, Figure S3.3).

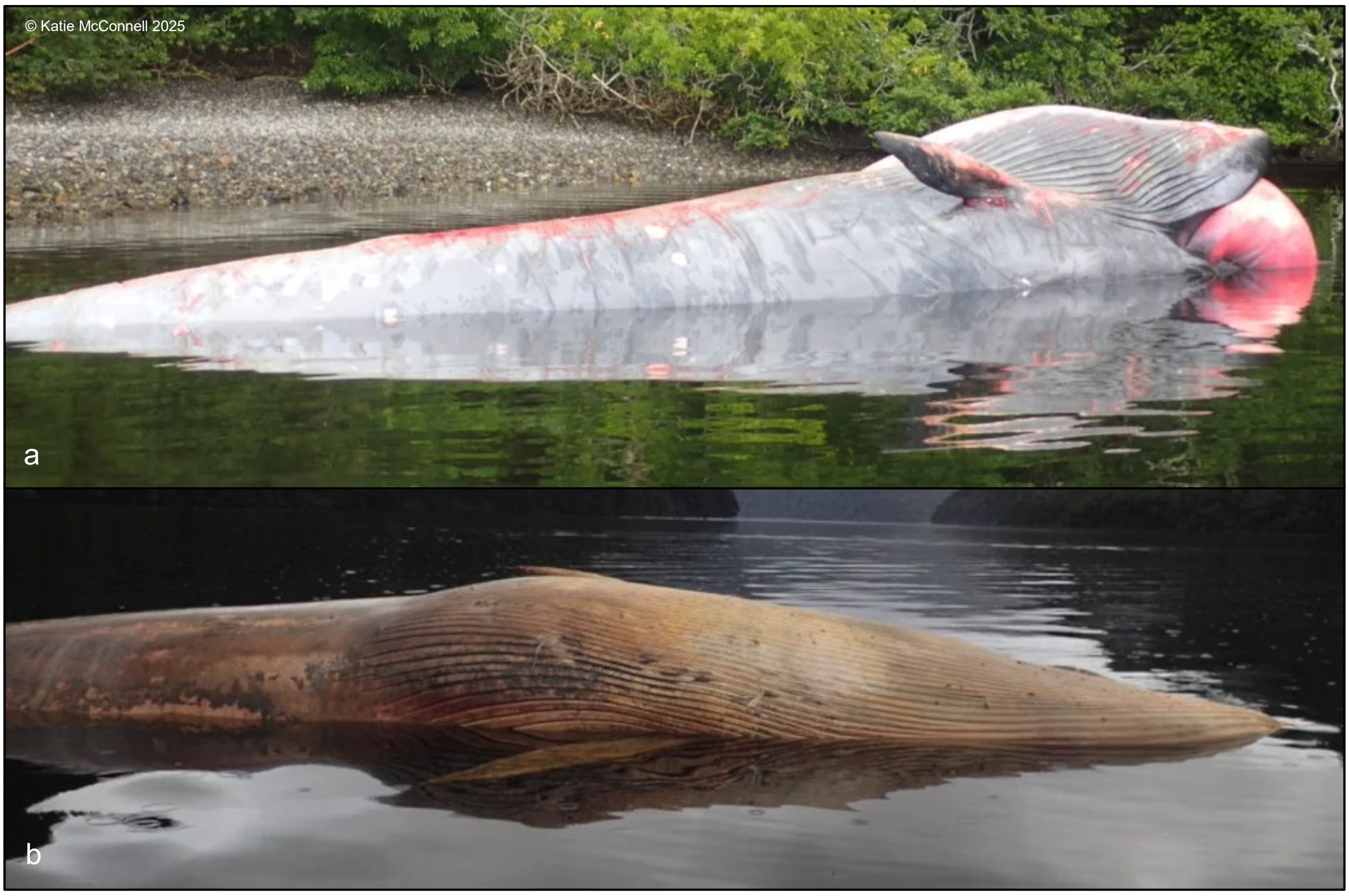

Figure 3.3: 'bloating_and_floating' sei whale with evident bloating (a, b), distended tongue (a) and blood-tinged blubber (a, b), captured in the Golfo de Penas, Chile, © Katie McConnell 2025

4) active_decomposition: 2-3 weeks after death, the carcass may be open, beginning rapid tissue loss via decomposition, microbes, maggots and scavengers, or the carcass may be intact but collapsed, skin sloughing, epidermis may be largely missing, exposing underlying blubber. As lipid-based tissues, like blubber are converted to adiopocere, a waxy grey liquid may be released and can cover wide areas around the remains. This can temporarily prohibit the growth of nearby plants. This may be visible from space, directly as a grey colouration surrounding a carcass during this phase, or indirectly due to reduced vegetation cover/death of vegetation nearby (UK: condition code 4, DCC code 4, Figure S3.4).

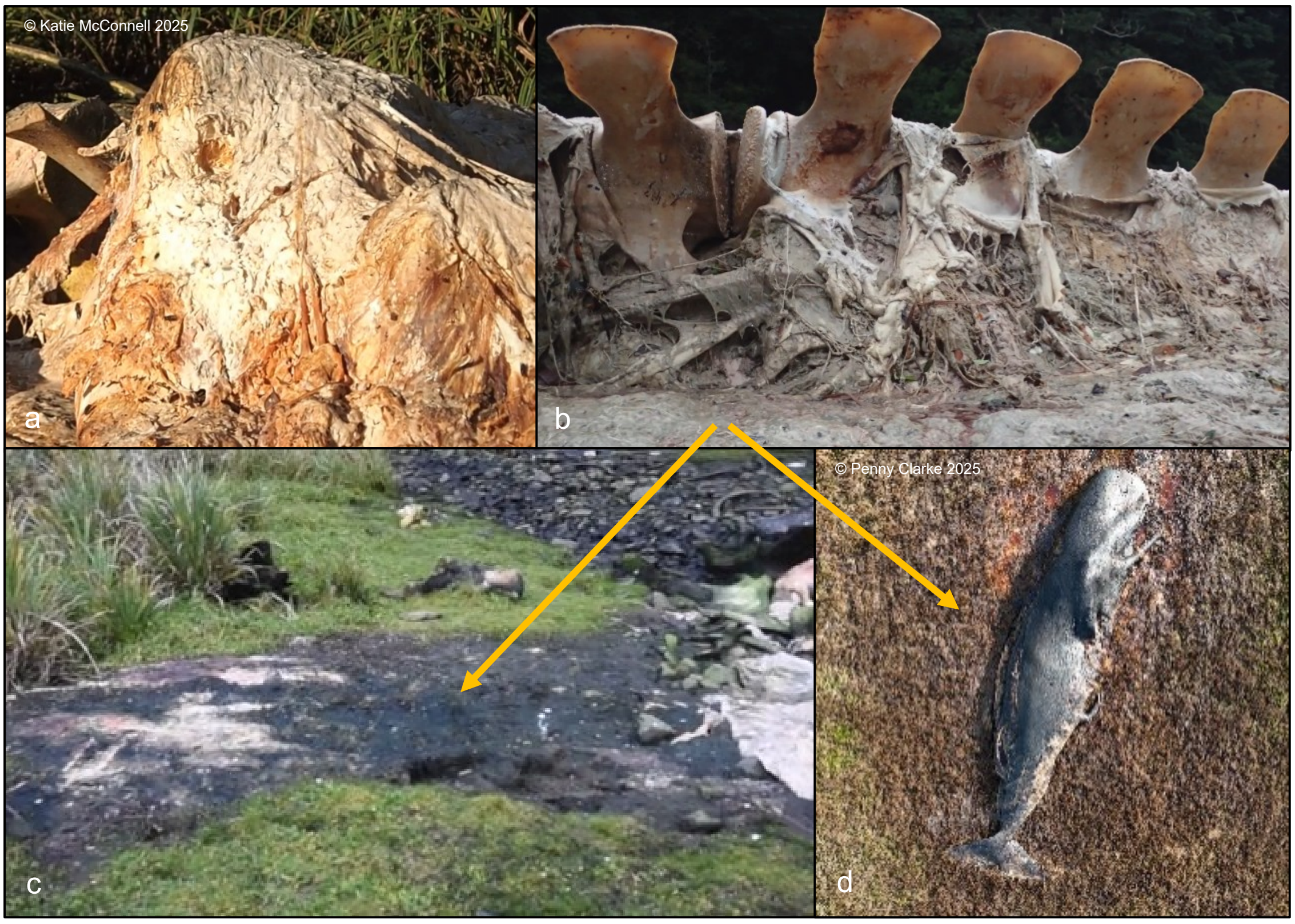

Figure S3.4: (a, b) A sei whale in 'active_decomposition' with rapid tissue loss and visible adipocere (waxy grey liquid), and (c) resulting prohibited growth of vegetation as a result of adipocere, captured in the Golfo de Penas, Chile, © Katie McConnell 2025. (d) Vegetation death associated with the conversion of lipid-based tissues to adipocere from a sperm whale in 'active_decomposition', captured in the Humber estuary, UK © Penny Clarke 2025.

The release of oils associated with this phase of decomposition could result in high reflectivity in satellite imagery, making a feature appear bright, even when visibly on the ground the carcass is predominantly dark in colouration (red/black) (Figure S3.5).

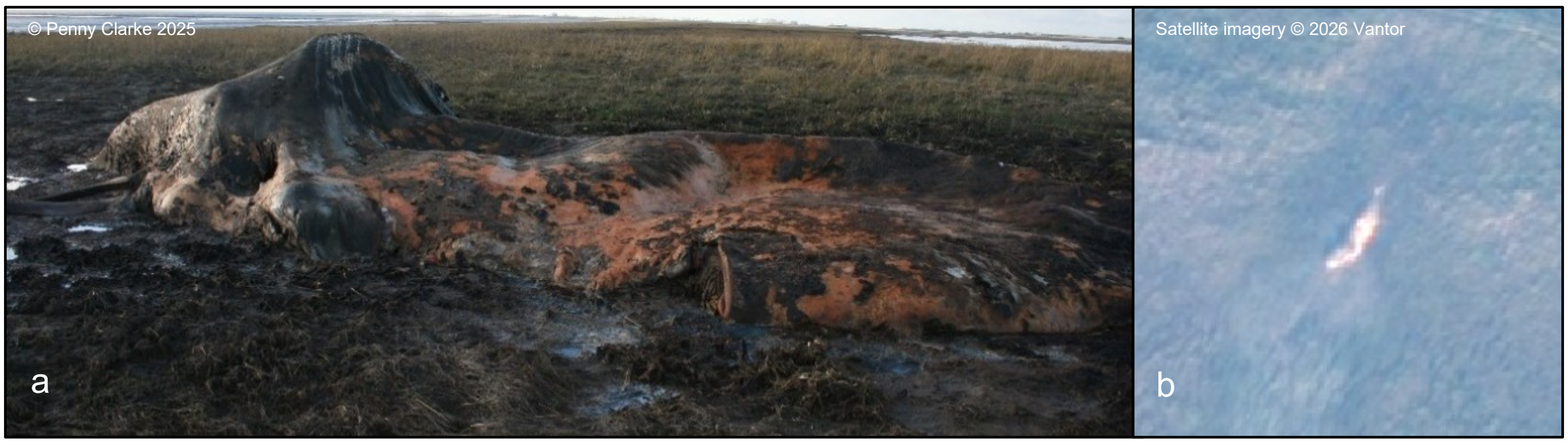

Figure S3.5: (a) A sperm whale in 'active_decomposition' captured from the ground in the Humber estuary, UK © Penny Clarke 2025. The carcass is intact but collapsed, with skin sloughing and the epidermis largely missing exposing the underlying blubber. Lipid-tissue conversion to adipocere is visible by the presence of a waxy grey liquid and death of the surrounding vegetation. From the ground the remaining skin of the carcass appears black and cracked due to sun exposure and decomposition, and the exposed blubber is blood tinged, giving the carcass an overall black and red colouration. (b) The same carcass captured in satellite imagery exhibits a bright white colouration, potentially as a result of the high reflectivity of adipocere, satellite imagery © 2026 Vantor.

5) passive_decomposition: Once the maggots leave the remains, the few remaining tissues are those that are more tough. Skin may be draped over skeletal remains; any remaining tissues are desiccated. Organs will have partially or totally disappeared. During the next year or more, storms and scavengers will gradually clean the remains from exposed skeletons (UK: condition code 5, DCC code 5, Figure S3.6).

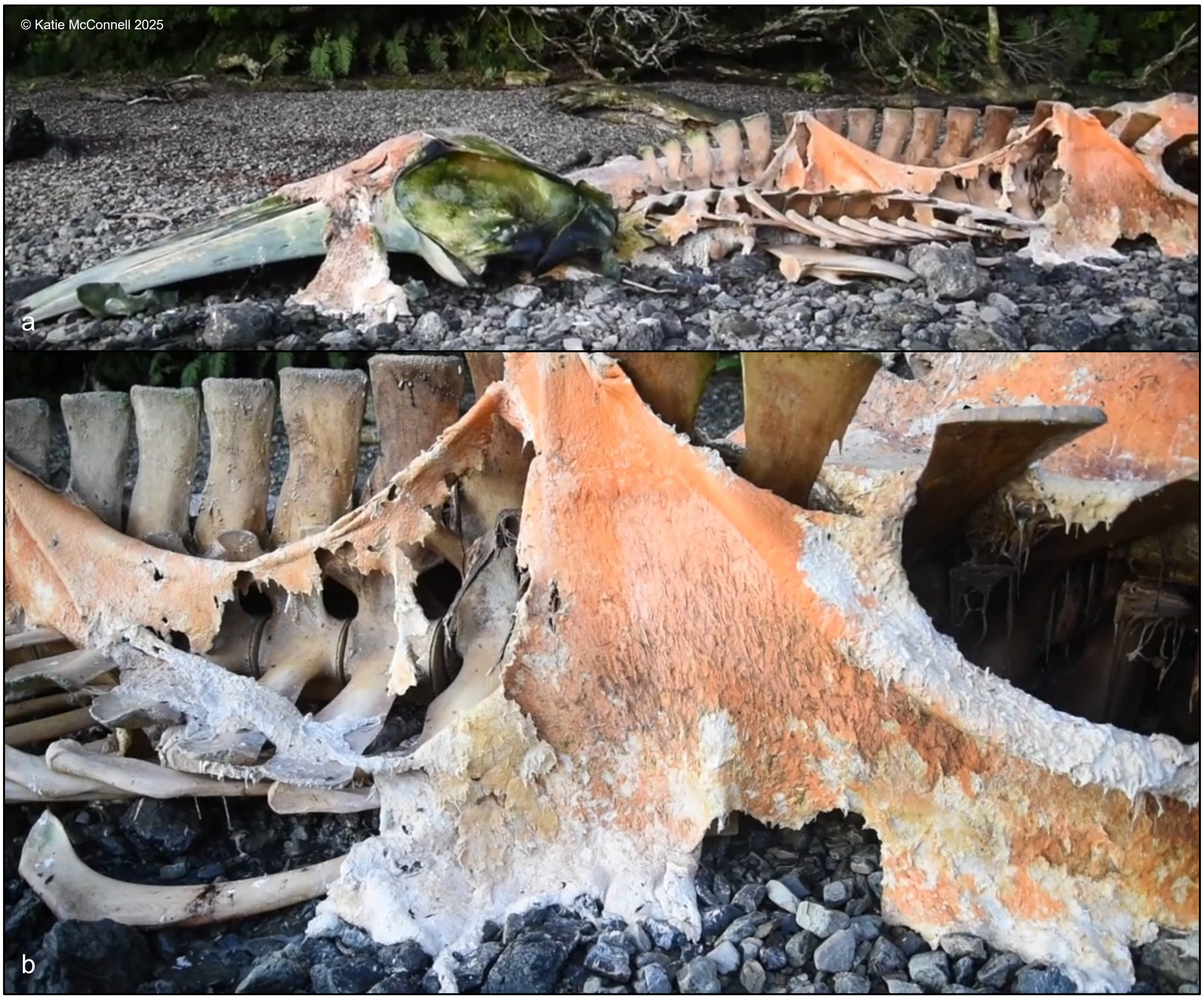

Figure S3.6: (a, b) A sei whale in 'passive_decomposition' with few remaining desiccated *tissues draped over skeletal remains, captured in the Golfo de Penas, Chile, © Katie McConnell 2025.*

6) dry_remains: For many years afterwards the bones from cleaned skeletons are scattered and eventually buried or disintegrated (UK: condition code 5, DCC code 5, Figure S3.7).

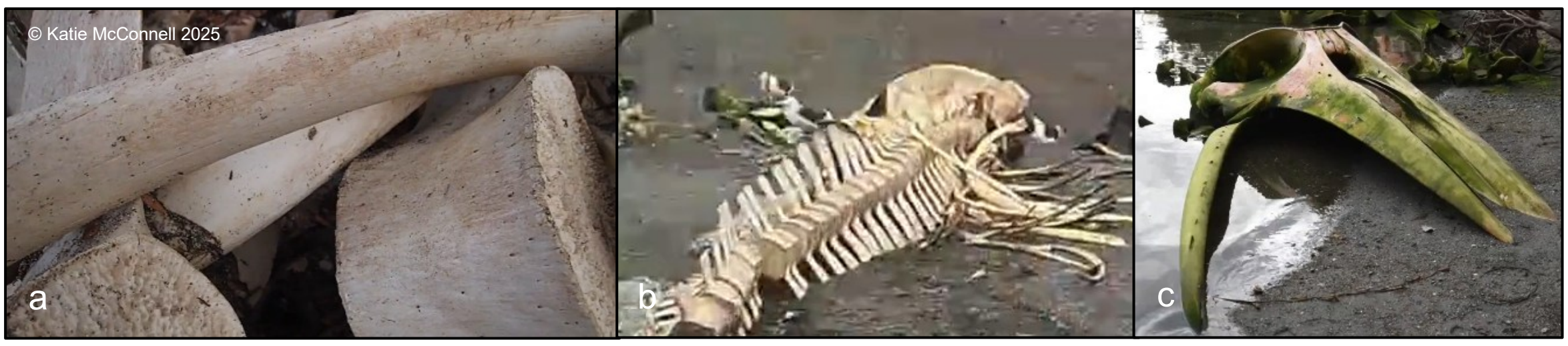

Figure S3.7: (a, b, c) 'dry_remains' of a sei whale, with cleaned skeletons intact and scattered, captured in the Golfo de Penas, Chile, © Katie McConnell 2025.

7) unknown: without prior knowledge or ground truth data, the level of decomposition cannot be confirmed.

species (species)

The list of species draws upon the standardised marine mammal species and subspecies defined by the Society of Marine Mammalogy (Committee on Taxonomy, 2025). The values within the template shapefile provides additional categories for stranded cetaceans identified in satellite imagery for when the species are unknown.

In the early stages of development of this tool, it is recommended the tool is applied to areas of known strandings, with the opportunity to ground truth. This will allow the field to establish examples of species in satellite imagery, providing greater capabilities to discern species in areas without prior knowledge.

Only assign a species for those you are certain of, for example, those you have ground data for, otherwise assign one of the following:

unidentified_dolphin

unidentified_large_baleen_whale

unidentified_large_whale

unidentified_porpoise

unidentified_small_whale

unidentified_whale

Body Colour (colour)

Body colour details a list of colours that may be associated with a live, stranded or decomposing cetacean. Select the most appropriate colour of your feature, by determining the most dominant colour by percentage cover of the 'stranded_cetacean' or 'floating_carcass' body. For smaller cetaceans, mixed pixels containing mixed spectra from cetacean and non-cetacean features may influence the visible colour. Mixed pixels could result in colours atypical for a cetacean or a given species. Colour should be assigned when the feature is viewed in true colour (red, green, blue bands). Contrast enhancement, brightness, contrast, gamma, and saturation settings, can impact colour and these settings should be adjusted per image by the user when viewing known features, to achieve a scene representative of true colour on the ground.

Body shape (body_shape)

Body shape details a list of body shapes that may be associated with a live, stranded or decomposing cetacean. Select the most appropriate body shape for your feature. 'irregular_ellipsoid' and 'irregular_streamline' may be suitable terms to describe carcass where decomposition acting upon the carcass, causes the carcass to collapse causing irregularity in the shape. For decomposing individuals assigning a body shape may become challenging, particularly where appendages break off or in the later stages of decomposition, in these cases body shape can be assigned 'undefinable'.

Body length (length_m)

Body length is a measure of the feature from the tip of the upper jaw to the notch of the tail (this may be across longitudinal (beak/fluke axis) if the cetacean is upright or if on its side across the sagittal plane from the cranial/anterior to caudal/posterior length), or the longest cross section in cases where satellite imagery is too coarse to determine jaw and tail, or where appendages are not visible (Figure

S3.8 and S3.9). For details on how to take a measure, review section 18 in the 'S1_QGIS_Strandings_from_Space_Pipeline' document. Measurement to be entered in meters to nearest 2 decimal points ('length_m', where 'm' denotes the unit metres).

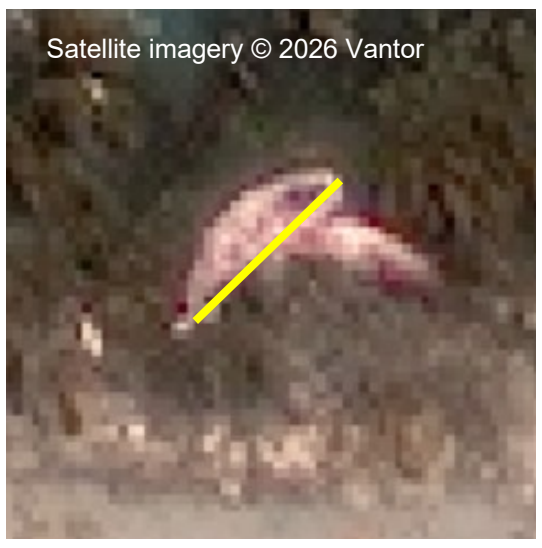

Figure S3.8: An example of measuring body length of a cetacean feature from the tip of the upper jaw to the notch of the tail, satellite imagery © 2026 Vantor.

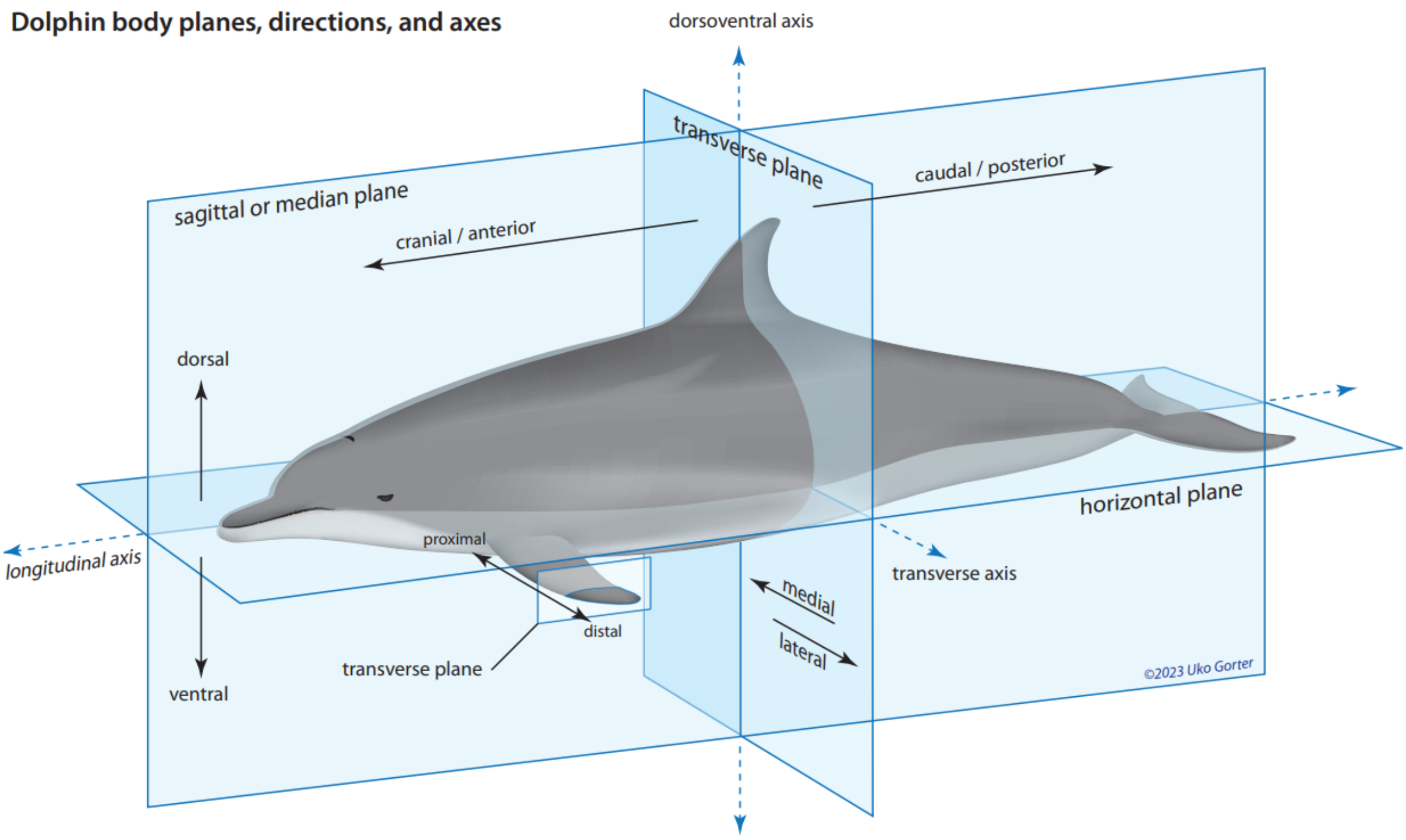

Figure S3.9: Positional veterinary anatomy used to define body morphometrics. For body length measurements, measure the longitudinal (beak/fluke axis) if the cetacean is upright or if on its side across the sagittal plane from the cranial/anterior to caudal/posterior length. For body width measurements, if the 'stranded_cetacean' or 'floating_carcass' is on its side measure across the sagittal plane from dorsal to ventral length or if the 'stranded_cetacean' or 'floating_carcass' is upright the transverse plane across the medial to lateral length, © Uko Gorter 2023.

Body width (width_m)

Body width is a measure of the feature at its widest cross section (if the 'stranded_cetacean' or 'floating_carcass' is on its side this will be across the sagittal plane from dorsal to ventral length or if the 'stranded_cetacean' or 'floating_carcass' is upright the transverse plane across the medial to lateral length) (Figure S3.9 and S3.10). For details on how to take a measure, review section 18 in the 'S1_QGIS_Strandings_from_Space_Pipeline' document. Measurement to be entered in meters to nearest 2 decimal points ('width_m', where 'm' denotes the unit metres).

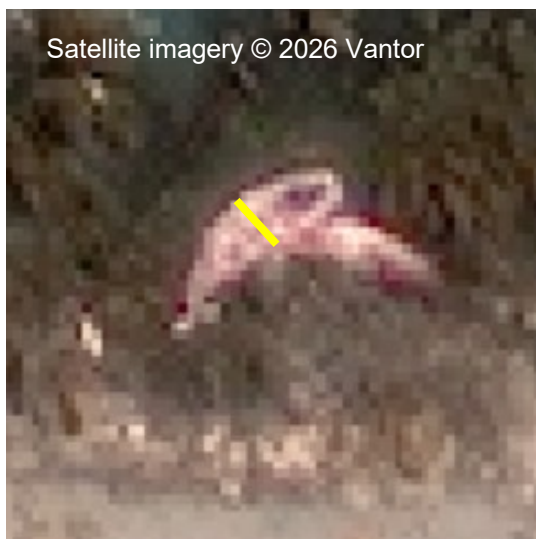

Figure S3.10: An example of measuring body width of a cetacean feature at the widest cross section, satellite imagery © 2026 Vantor.

Flippers (flippers)

Flippers are the forelimbs used to turn, break and stabilise a cetacean while swimming. This value is a presence or absence of flippers, yes, no, or maybe. Flippers are only likely to be visible for larger stranded cetacean species, in fresh strandings of smaller cetacean species, or live cetaceans, as flesh rots and appendages break off during decomposition.

Fluke (fluke)

Fluke is a tail used to propel a cetacean forward. This value is a presence or absence of a fluke, yes, no, or maybe. A fluke is likely only to be visible for larger stranded cetacean species, in fresh strandings of smaller cetacean species, or live cetaceans, as flesh rots and appendages break off during decomposition.

*On sandy beaches tail stocks can become buried, giving the appearance that a fluke is separate from the body (Figure S3.11). Observers should be mindful of this as it could lead to misidentification of flukes as calves.

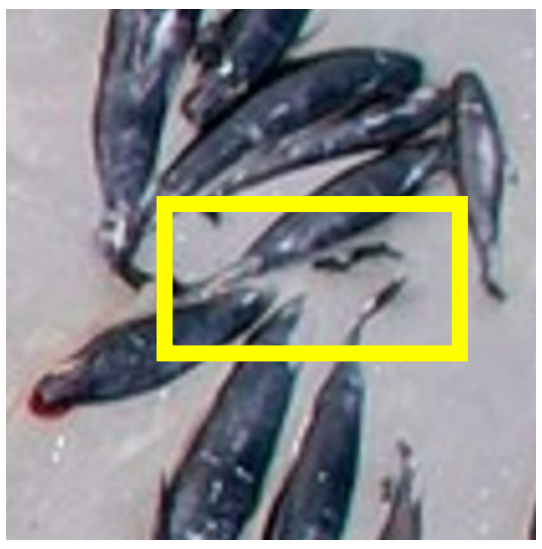

Figure S3.11: An example where a tail stock has become buried by sand, giving the appearance that the fluke is separate to the body, © Tamzin Henderson 2022.

Slick and Blood (slick/bld)

Slick refers to adiopocere, a wax like substance formed when lipid-based tissue breaks down as stranded cetaceans decompose such as, blubber. The waxy grey liquid released can cover wide areas around the remains. Blood can refer to bloody directly or blood-tinged organs or blubber. This value is a presence or absence of a slick or blood, yes, no or maybe. Slicks are only likely to be visible in decomposing strandings, once the blubber is decomposing. Slicks can temporarily prohibit the growth of nearby plants, so reduced vegetation nearby or the death of vegetation and grey colouration surrounding a cetacean may be seen from space during this phase.

Group (group)

Group is the number of individuals identified within a 1km radius of a marked point. The number of individuals does not have to be exact but a best estimate.

Please note: the following attributes are environmental variables and requires the conditions at the point feature / annotation specifically, and not the whole image

The environmental variable attributes, such as cloud cover, coastal type, and the shade conditions can all impact the confidence of an observer in assigning a certainty, hence, why the conditions at the point of the feature are noted, to greater evaluate their impact.

Cloud Cover (cloud_cov)

Cloud cover, using the aviation industry terminology, is reported in terms of 1/8th of sky cover, otherwise known as oktas, the terms are as follows (*e.g.,* 0 oktas represent the complete absence of cloud):

'skc_sky_clear' = 0/8th

'few_traces' = 1-2/8th

'sct_scattered' = 3-4/8th

'bkn_broken' = 5-7/8th

'ovc_overcast' = 8/8th

Cloud Thickness (cloud_thick)

Cloud thickness (as per Cubaynes et al., 2023) should be defined as one of the following:

1) thin: can see fairly well through the cloud

2) medium_thin: can see through but no clear view of the ground

3) thick: can't see through

Environment (env)

Environment is a list of common environments a stranded, floating dead or live cetacean may be found, for example, a light sand beach, <1km nearshore waters, or mangroves. If the environmental context is unknown, to their best knowledge, the observer should assign an interpretation of the environment that is most applicable to the conditions visible in the satellite image at the marked point.

Image brightness (img_bright)

Image brightness can differ due to combination of factors, the suns angle, the nadir angle (the angle of the satellite sensor to the ground, 0° is directly overhead, and the greater the nadir angle, the greater the distortion), and contrast between environments, for example highly reflective ice next to absorbent ocean. Individual observers are to assign image brightness based on their interpretation of the image conditions at the marked point.

Shade (shade)

Shade is an evaluation of the light conditions at a marked point, which is influenced by the incoming UV radiation. For example, a vegetated coastline or local topography relative to the suns angle may result in a feature being in the shade. A feature may be fully or partially shaded or not in the shade.

Ice Cover (ice_cover)

Ice cover is only relevant to regions where ice is present, for example, the Polar Regions. Ice would likely mostly impact the detection of 'floating carcasses' or 'live cetaceans', but in regions of dense ice,

ice may arrive on shore and could impact the detection of stranded cetaceans. Ice cover is an evaluation of the density of ice present in the region at a marked point.

Comments (comments)

Comments is a space for observers to note any other information relating to a feature that may be important for data analysis.

2. Visual guide

The following guide provides visual examples of the standardised attributes and associated values in VHR optical satellite imagery (0.5, 0.3 and 0.15 m spatial resolution), supplemented with aerial or ground imagery to aid the confident detection of stranded cetaceans in VHR satellite imagery. **All image examples included here were validated through use of ground or aerial data**. Some attributes are absent of image examples, as they were not available at the time of creation. Please note, image examples are intended to act as a guide, **users should apply caution in assigning attributes based on these image examples in absence of ground or aerial data for validation, or if there is any uncertainty**.

a. Required attributes

Table S3.3: For each required standardised attribute associated with ‘stranded_cetacean’ and ‘floating_carcass’, image examples are provided for the respective standardised values, where possible from VHR optical satellite imagery across different spatial resolutions (0.5, 0.3 and 0.15 m), and supplemented with aerial and ground image examples.

Attribute	Description	Satellite 0.15 m	Satellite 0.3 m	Satellite 0.5 m	Aerial	Ground
**feature**	stranded_cetacean	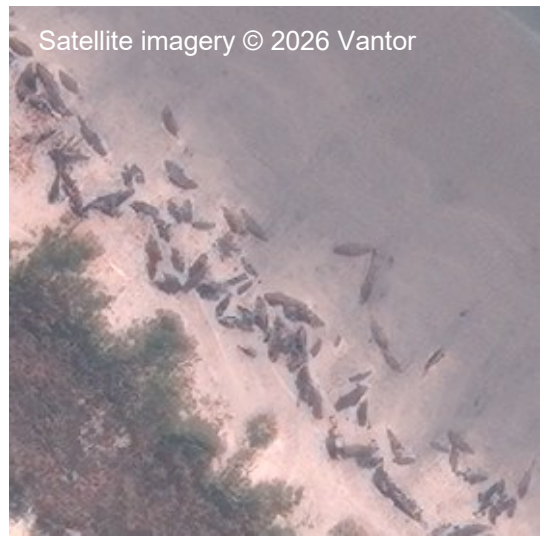	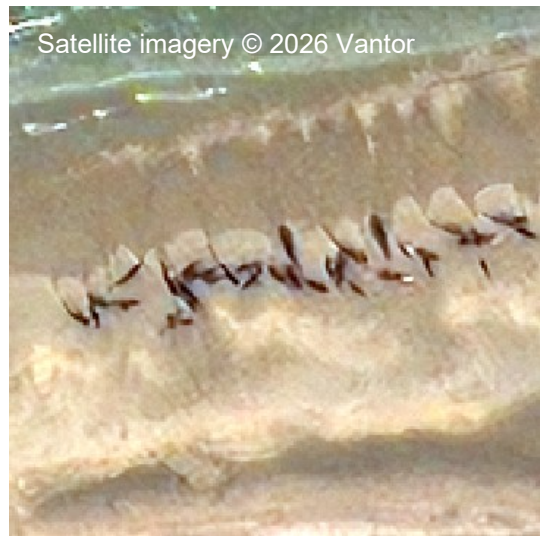	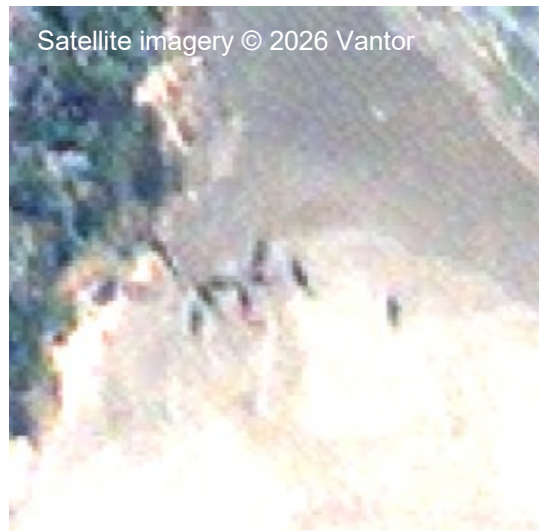	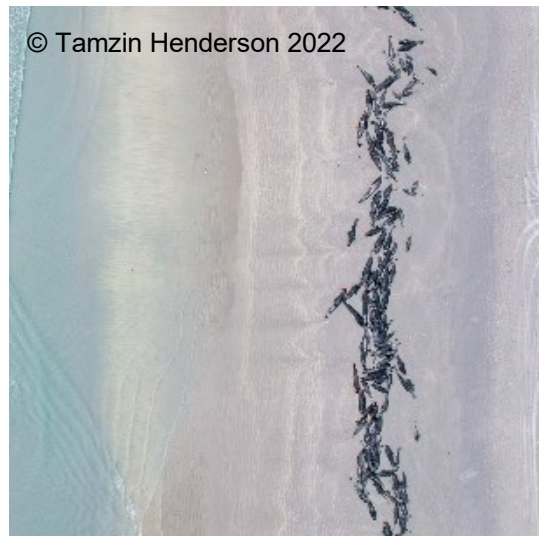	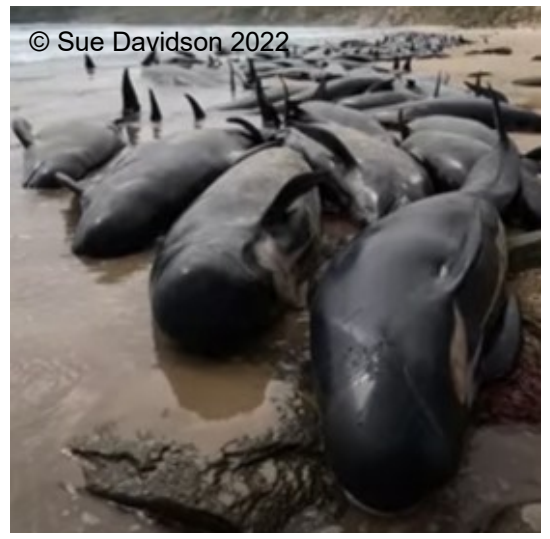
			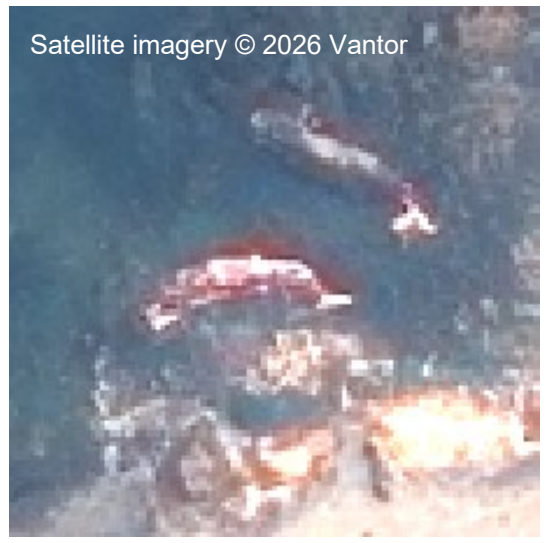	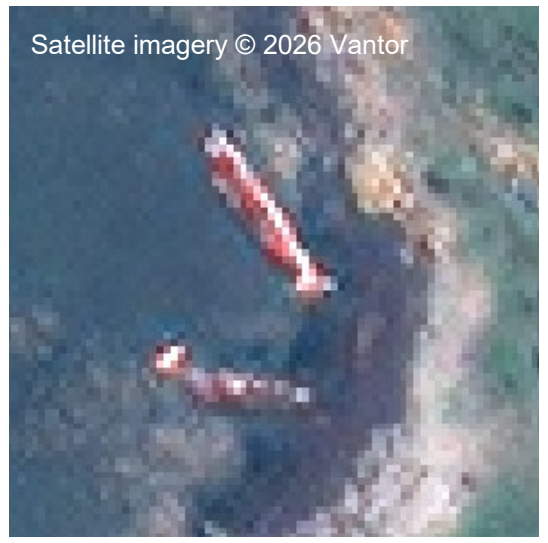	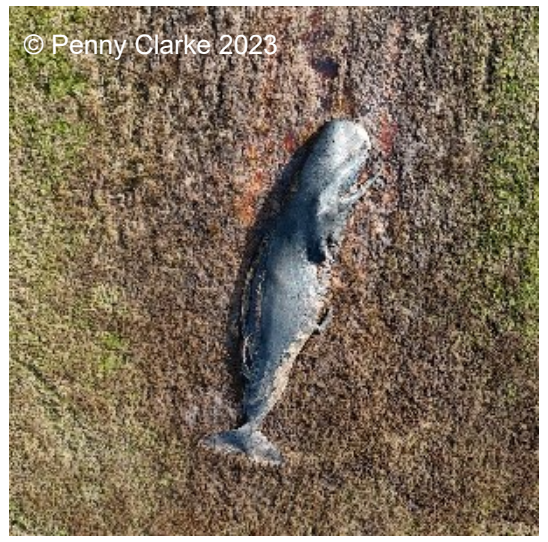	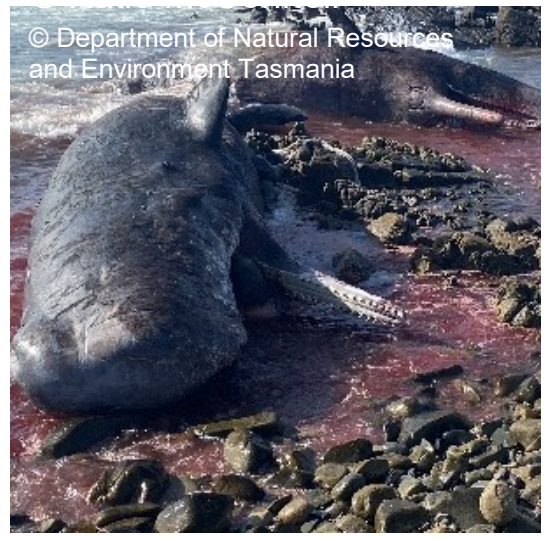
		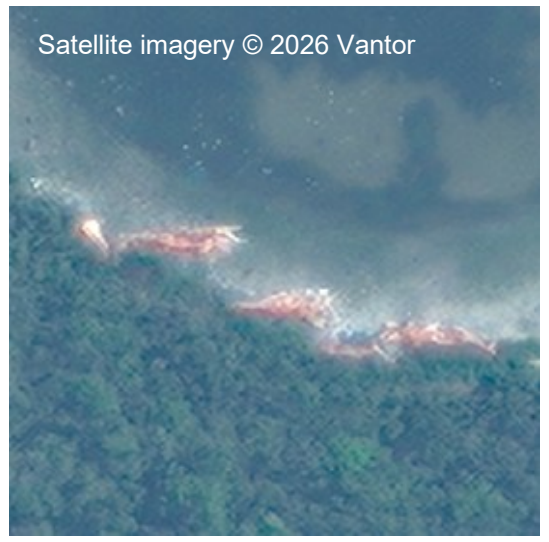	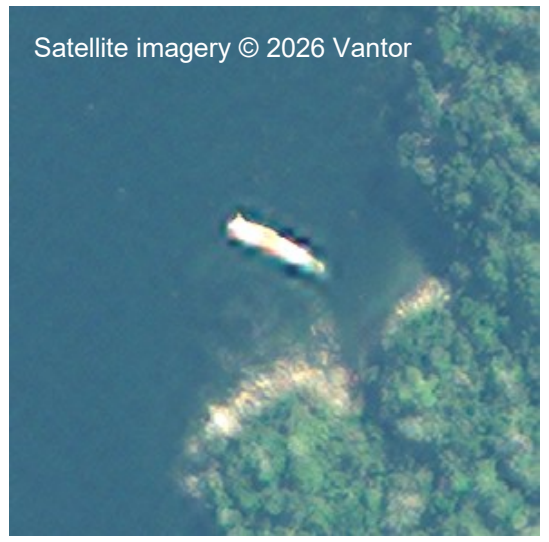	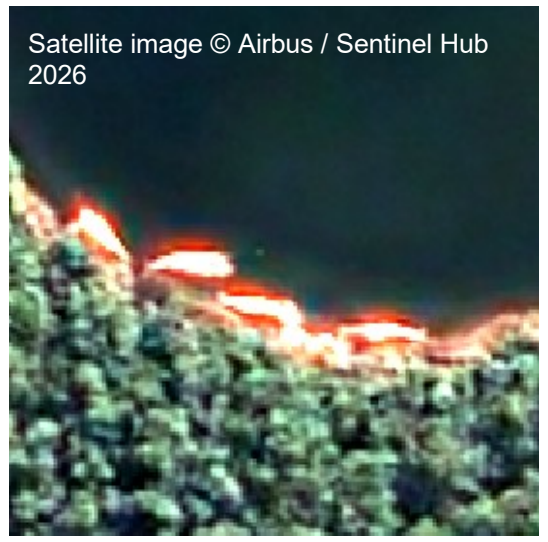	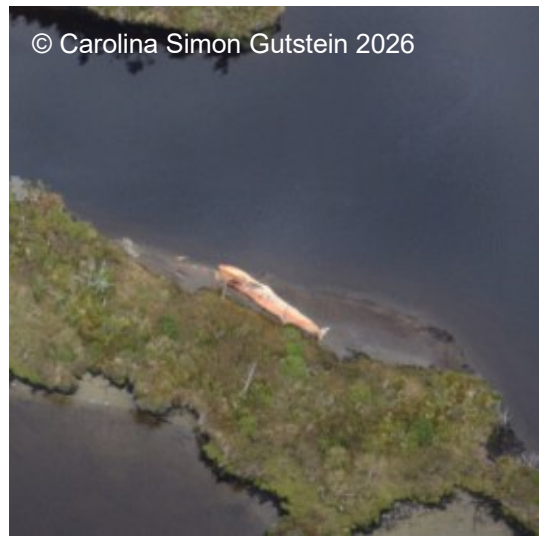	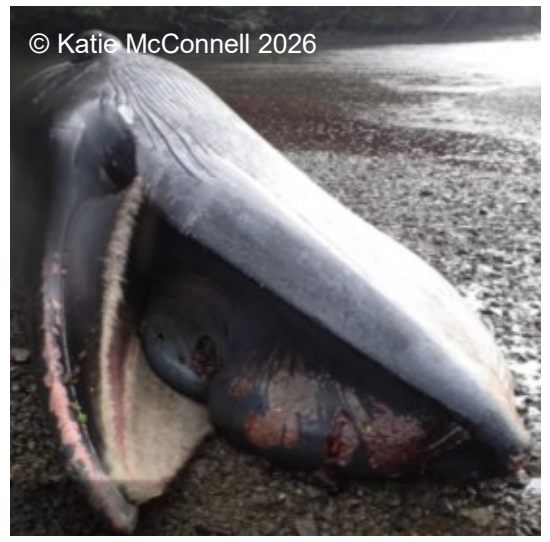

Attribute	Description	Satellite 0.15 m	Satellite 0.3 m	Satellite 0.5 m	Aerial	Ground
	floating_carcass		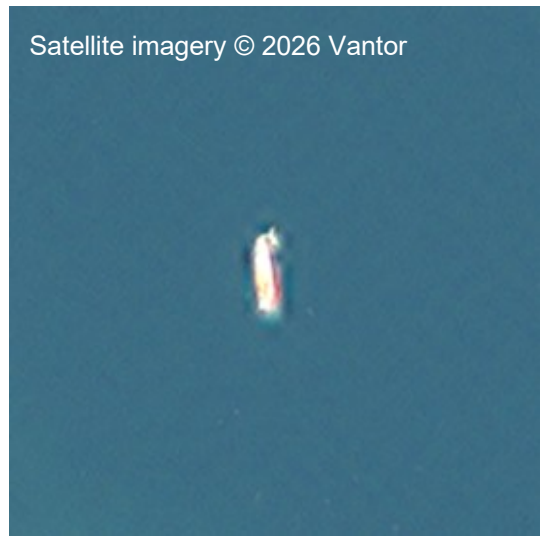	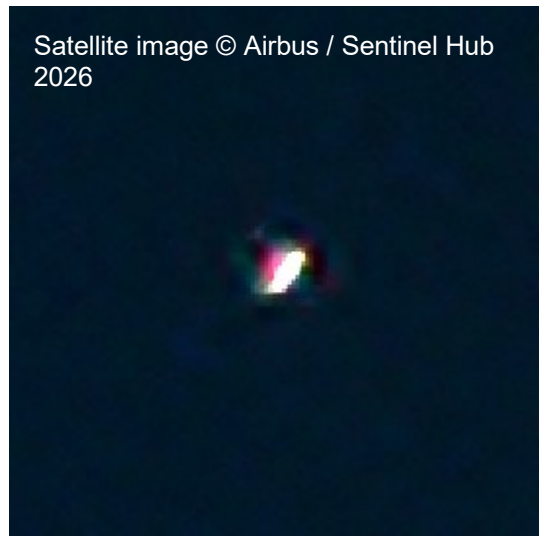		
	live_cetacean		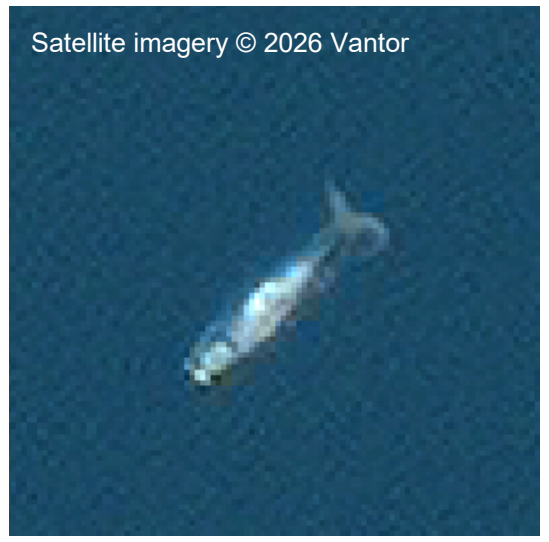	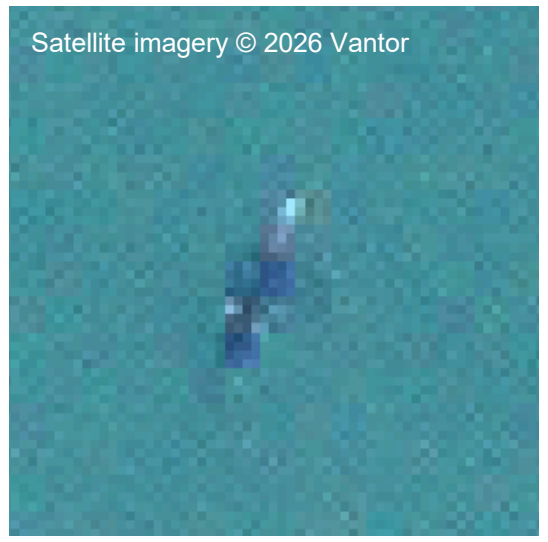	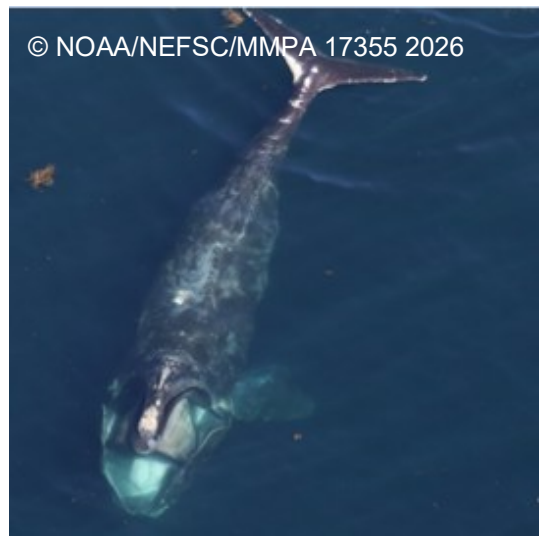	
			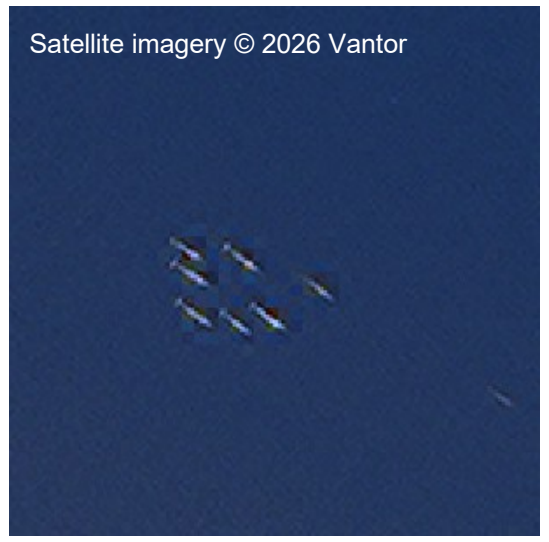	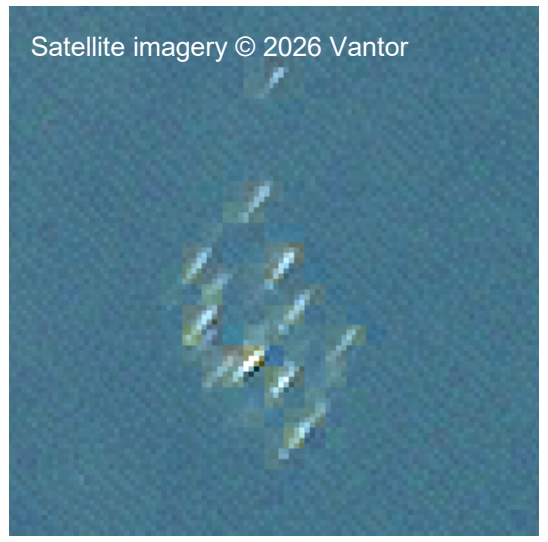	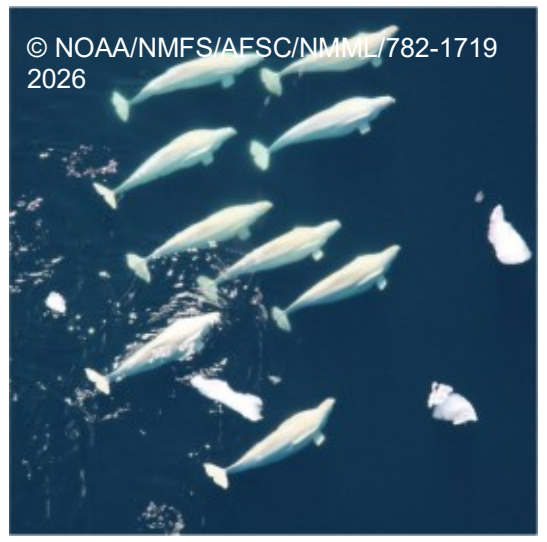	
			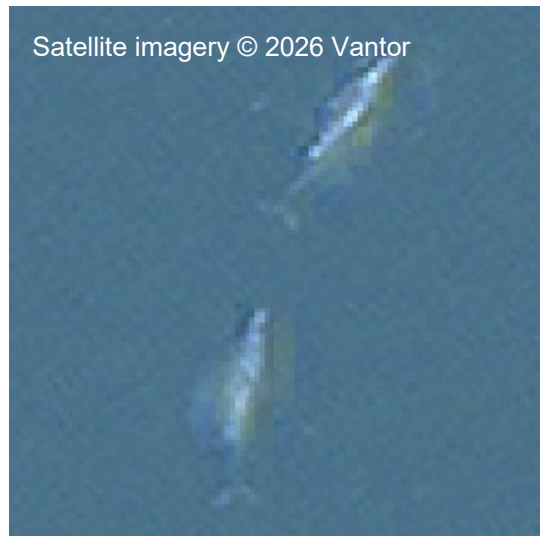	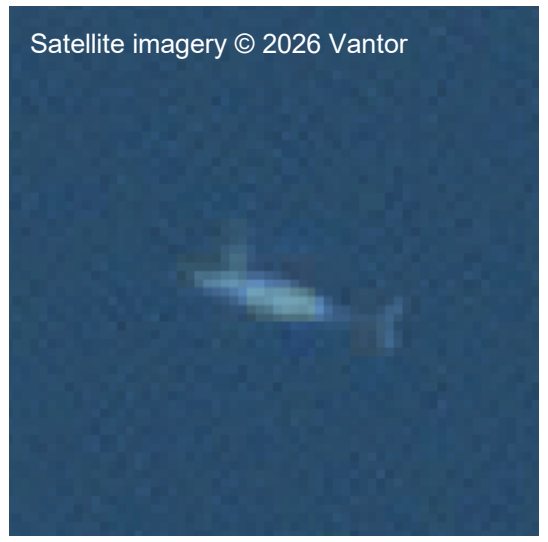	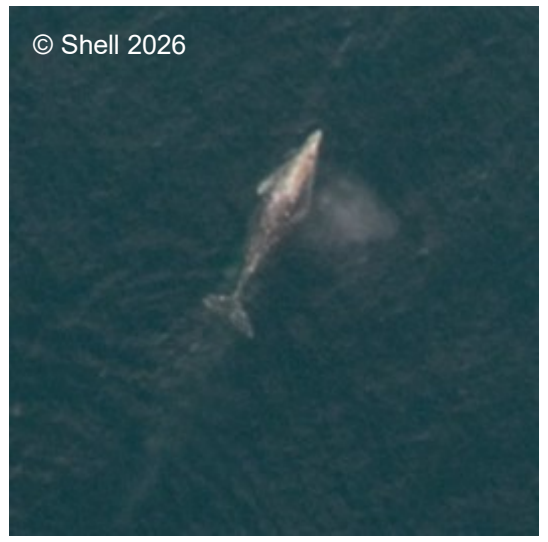	

Attribute	Description	Satellite 0.15 m	Satellite 0.3 m	Satellite 0.5 m	Aerial	Ground
	boat			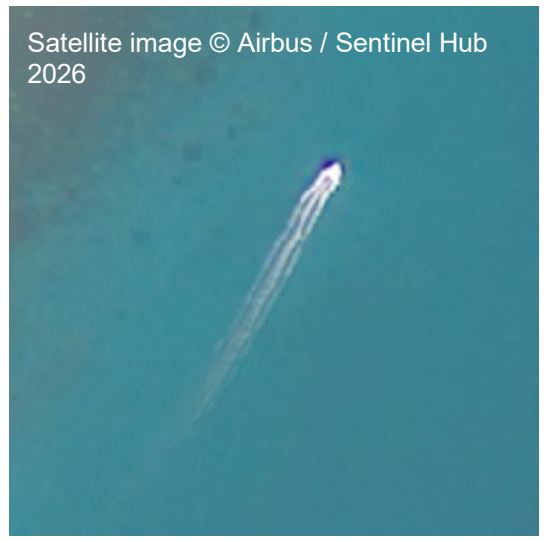 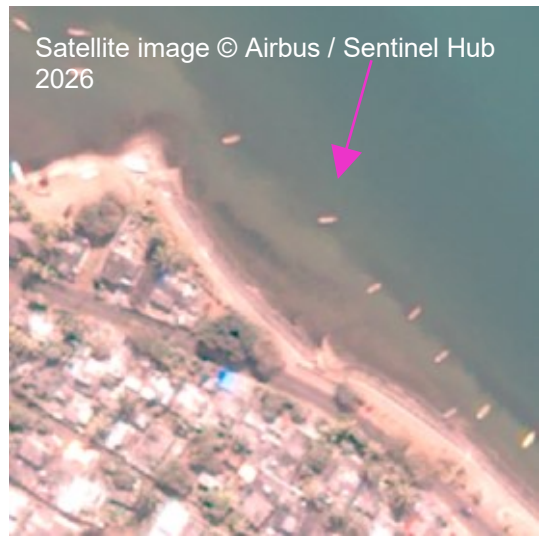 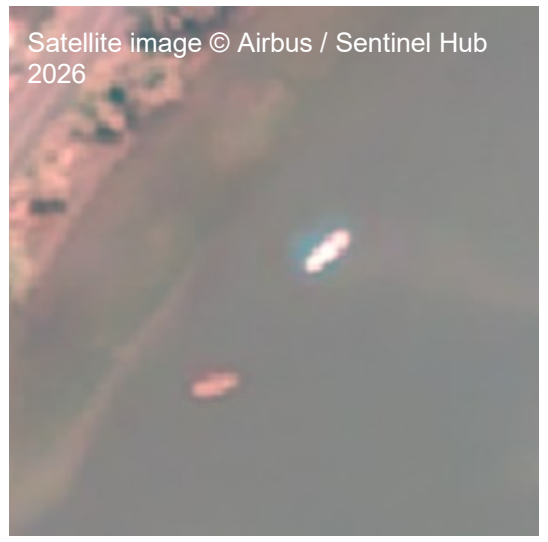		
	marine_litter					

Attribute	Description	Satellite 0.15 m	Satellite 0.3 m	Satellite 0.5 m	Aerial	Ground
	organic_beach_debris			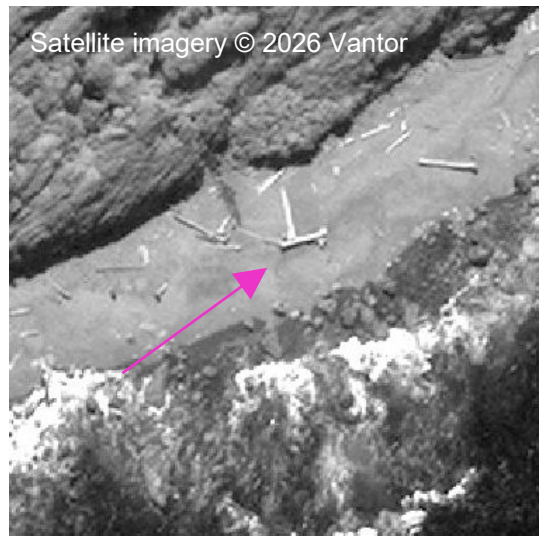<br>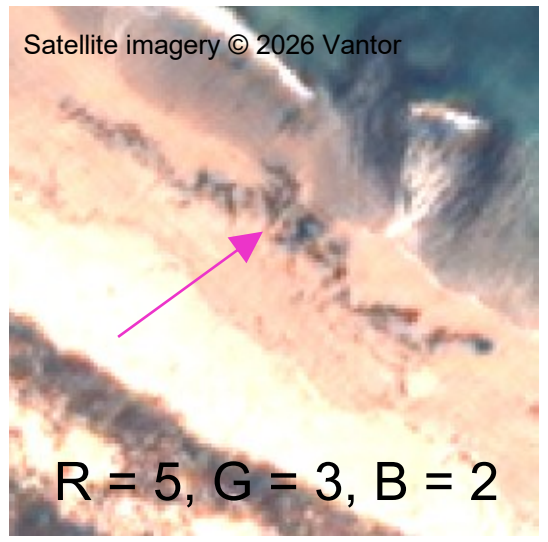<br>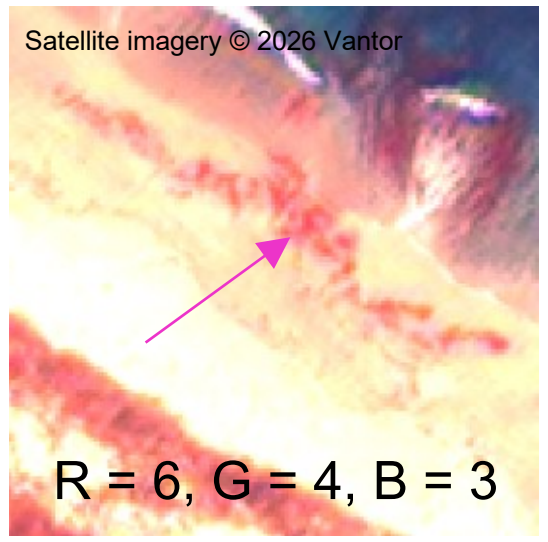		
	rock		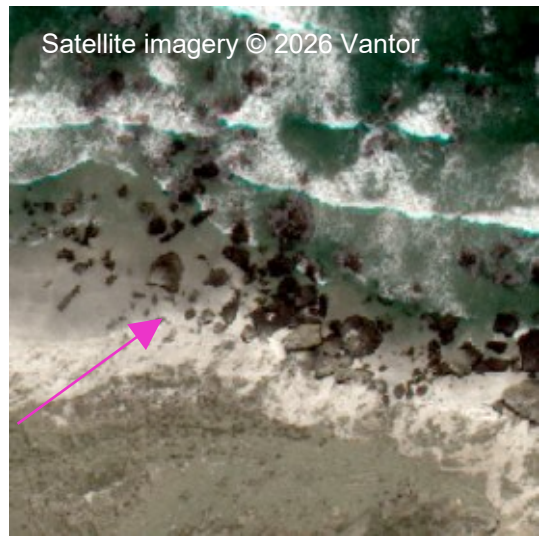	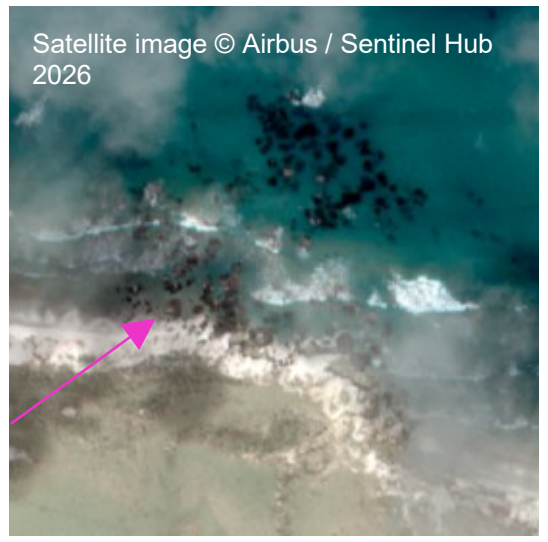		

Attribute	Description	Satellite 0.15 m	Satellite 0.3 m	Satellite 0.5 m	Aerial	Ground
				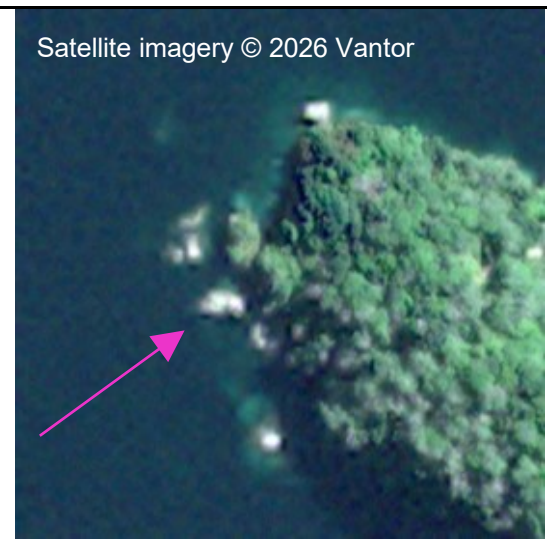		

b. Optional attributes

Table S3.4: For each optional standardised attribute associated with 'stranded_cetacean' and 'floating_carcass', image examples are provided for the respective standardised values, where possible from VHR optical satellite imagery across different spatial resolutions (0.5, 0.3 and 0.15 m), and supplemented with aerial and ground image examples.

Attribute	Description	Satellite 0.15 m	Satellite 0.3 m	Satellite 0.5 m	Aerial	Ground
**deco_ phase**	fresh_alive					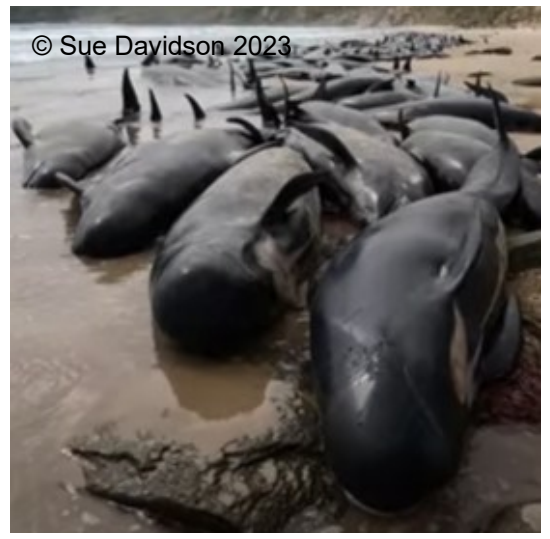
	fresh_dead		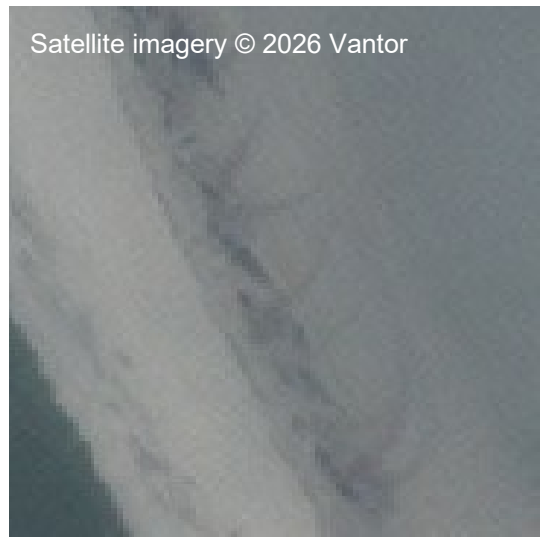		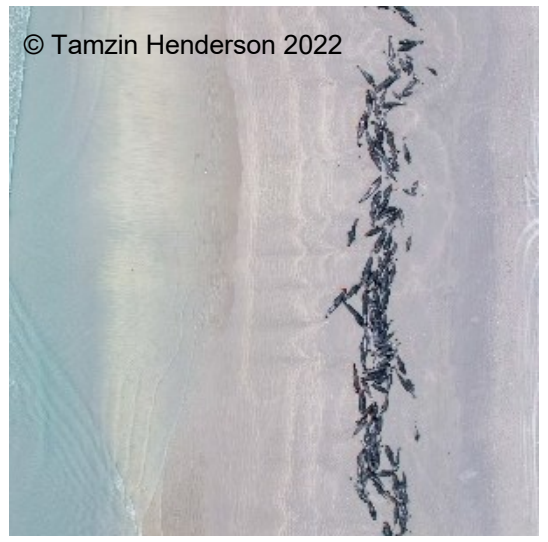	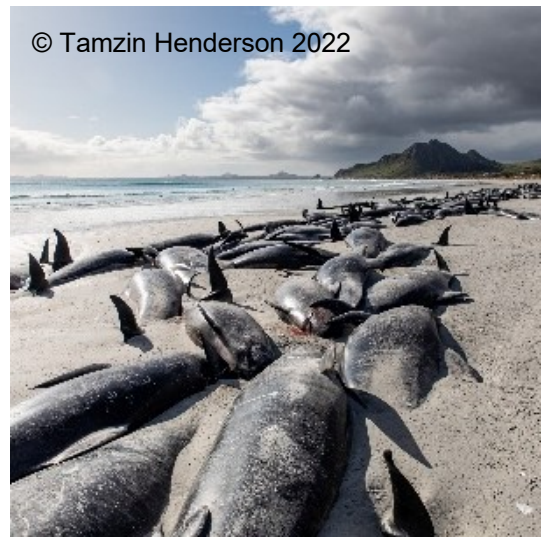

Attribute	Description	Satellite 0.15 m	Satellite 0.3 m	Satellite 0.5 m	Aerial	Ground
						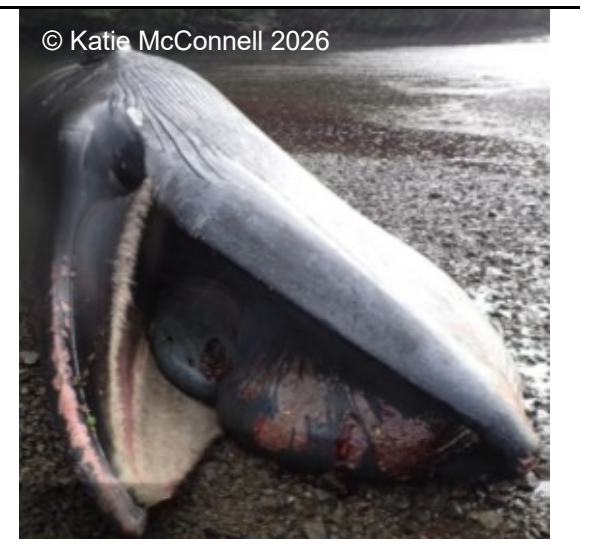
	bloating_and_floating					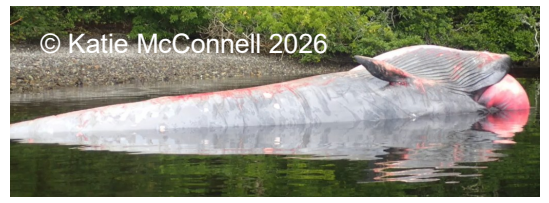 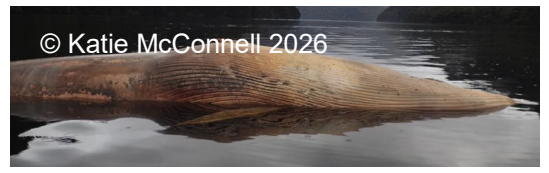 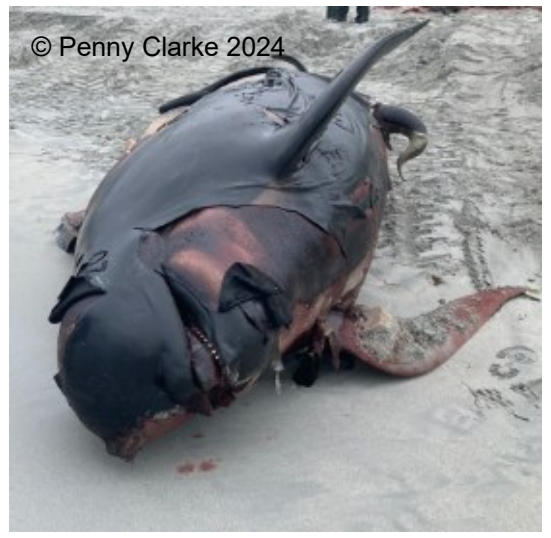
	active_decomposition	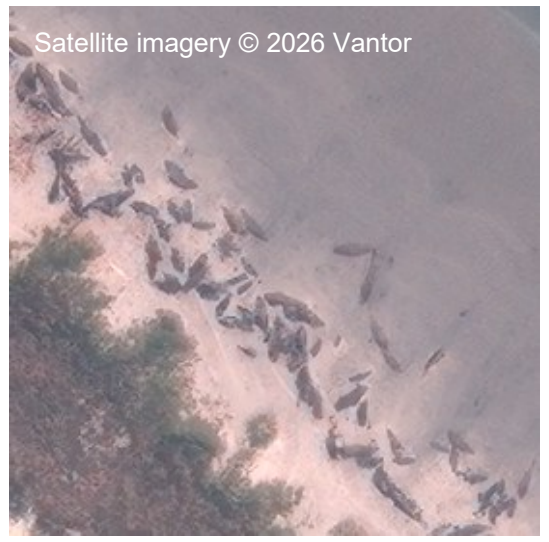	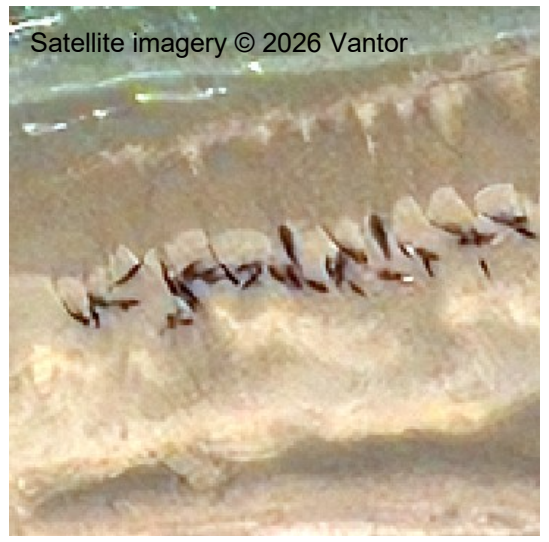	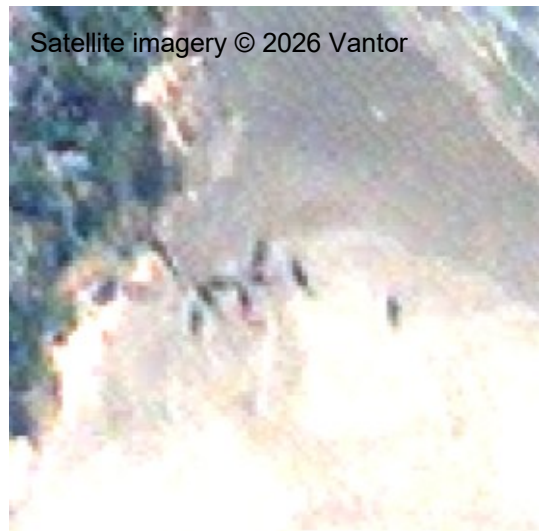		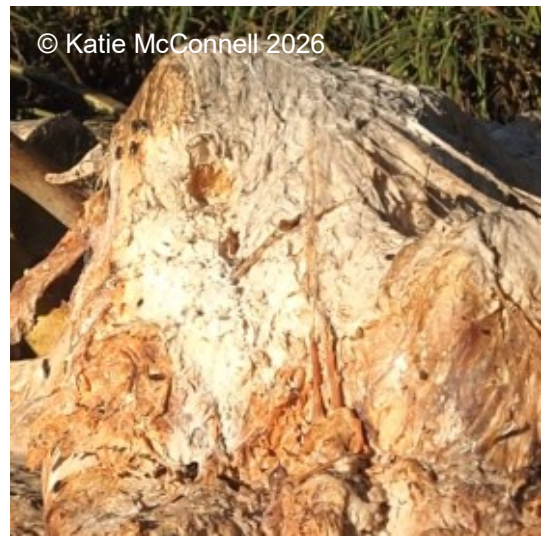

Attribute	Description	Satellite 0.15 m	Satellite 0.3 m	Satellite 0.5 m	Aerial	Ground
			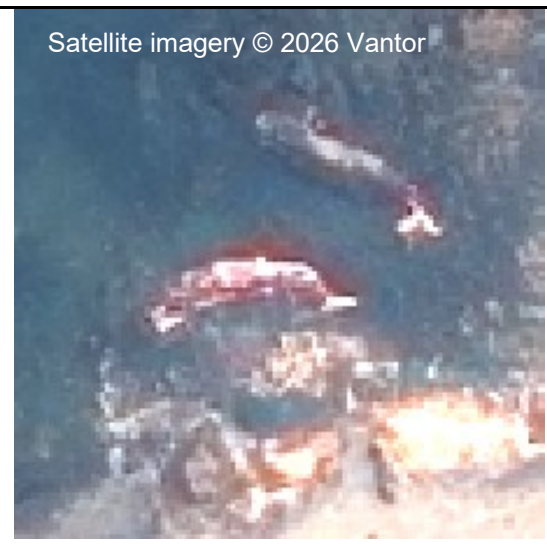	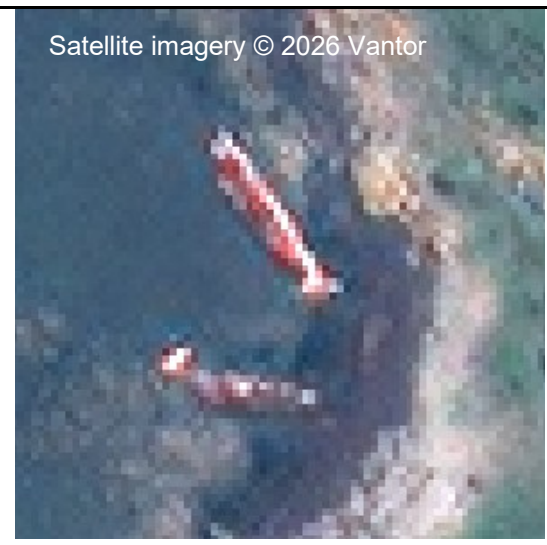 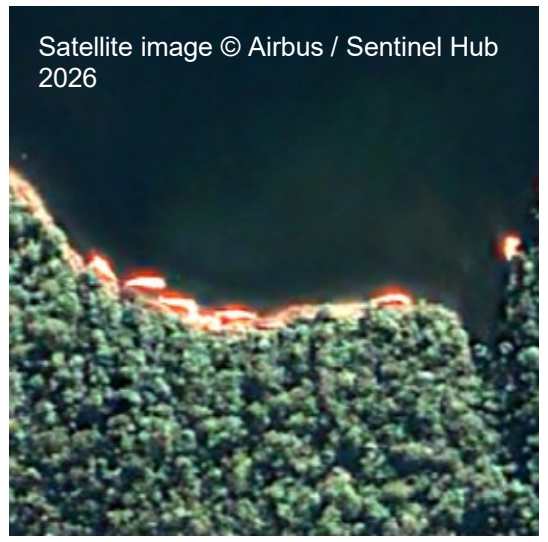	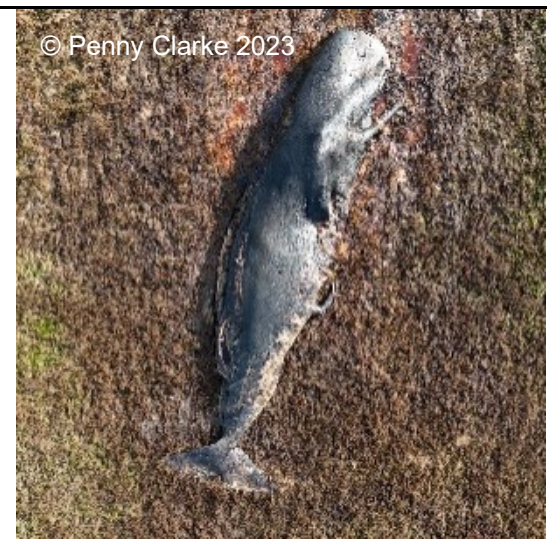	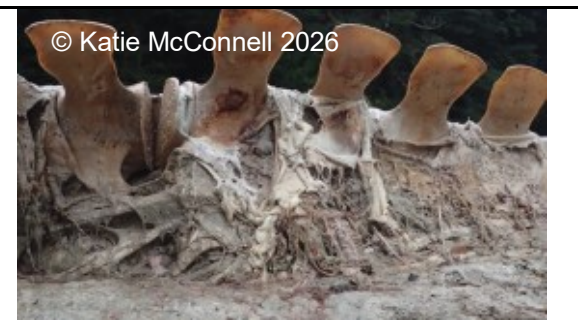
	passive_decomposition	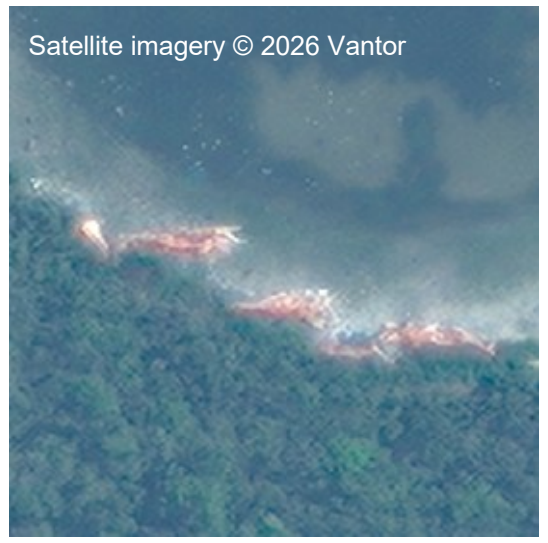	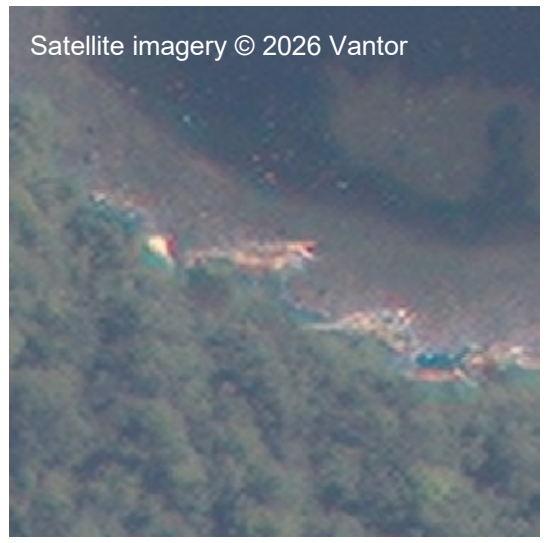	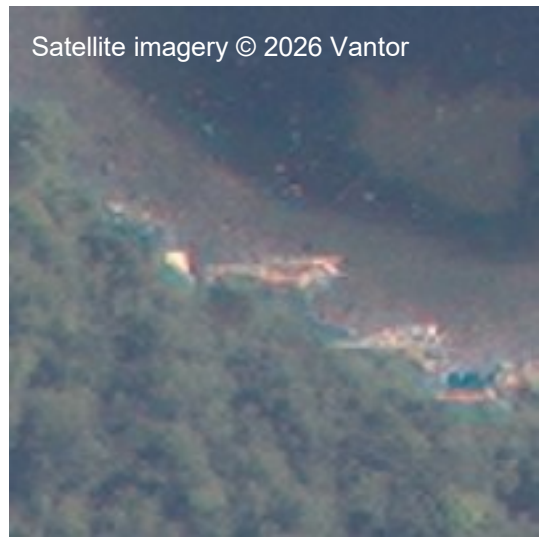		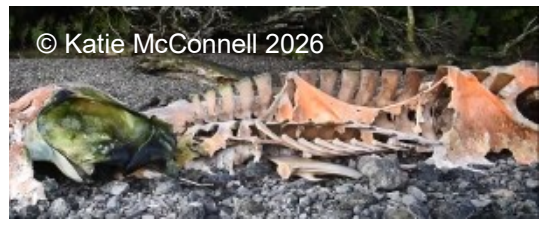 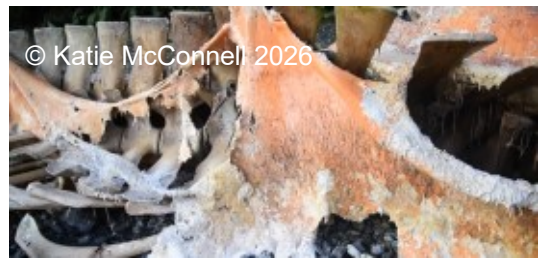
	dry_remains			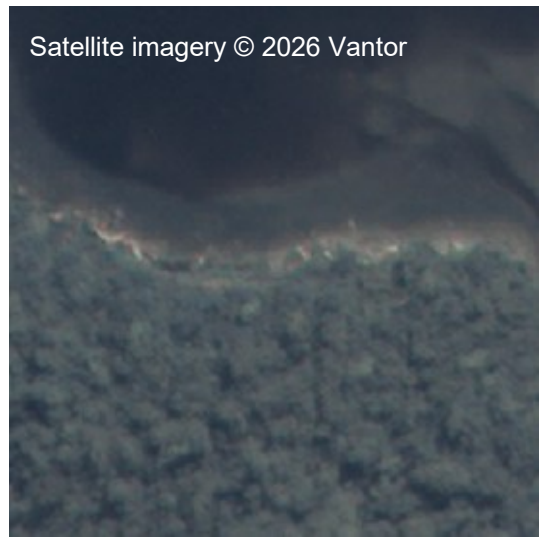		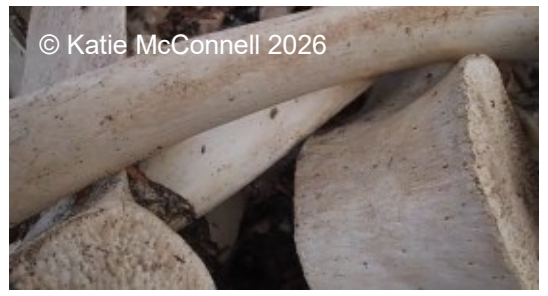

Attribute	Description	Satellite 0.15 m	Satellite 0.3 m	Satellite 0.5 m	Aerial	Ground
				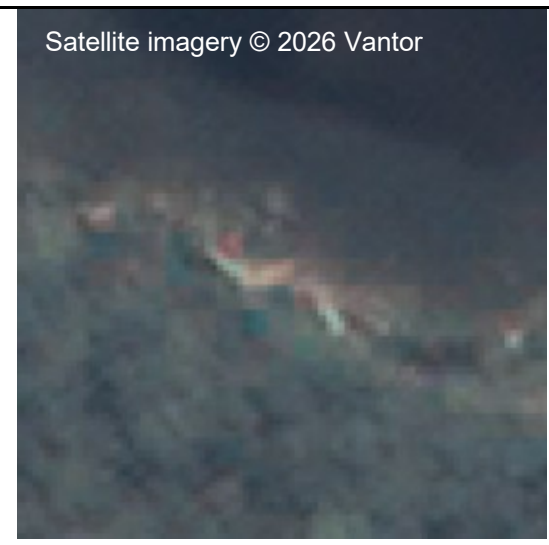<br>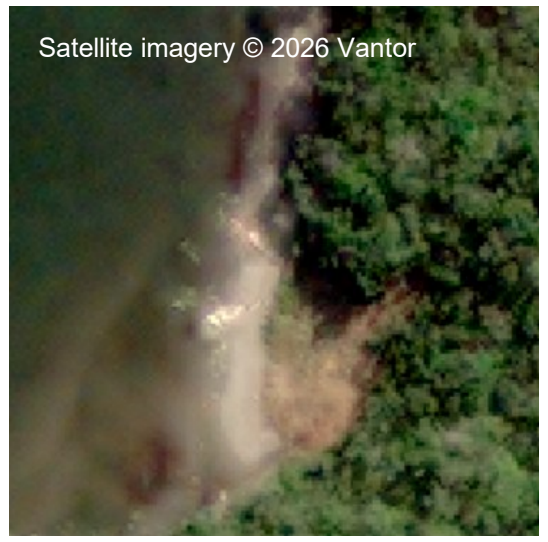		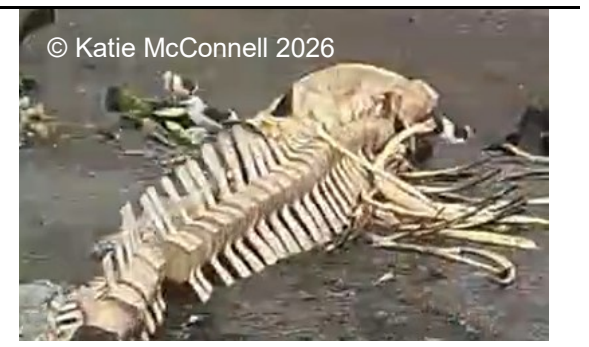<br>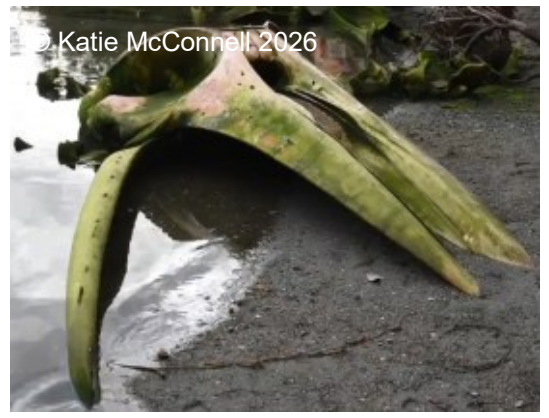
	unknown					
**species**	sei_whale<br><br>*Balaenoptera borealis*	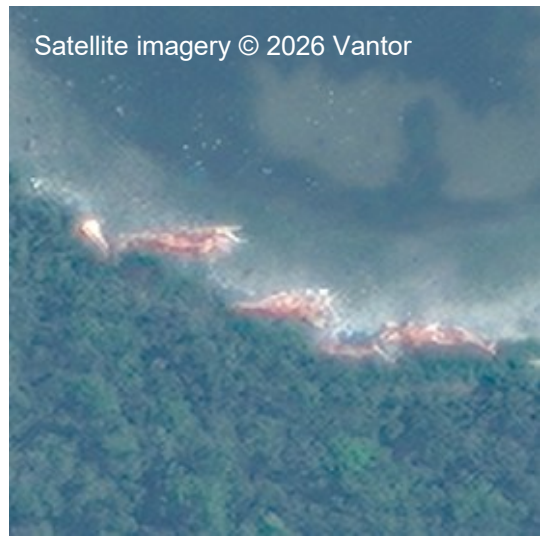	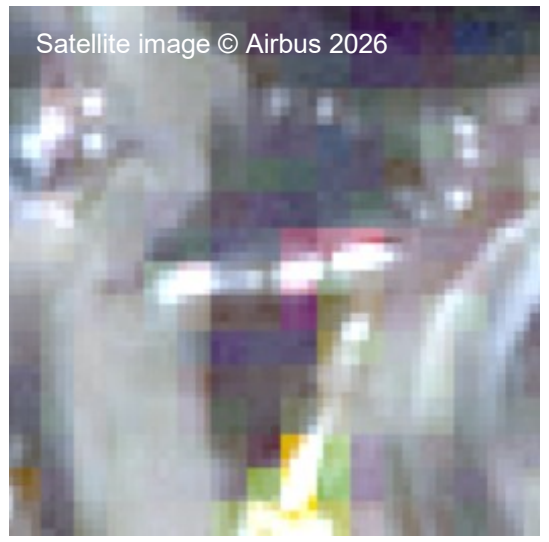	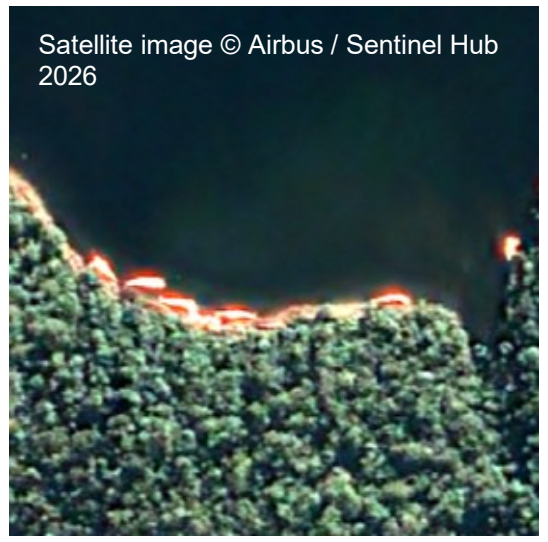	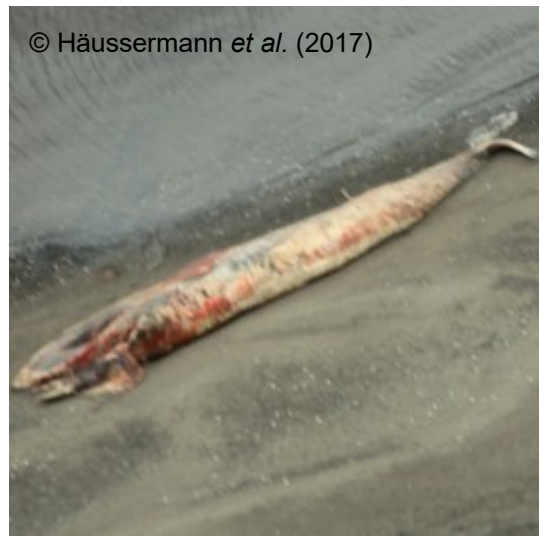	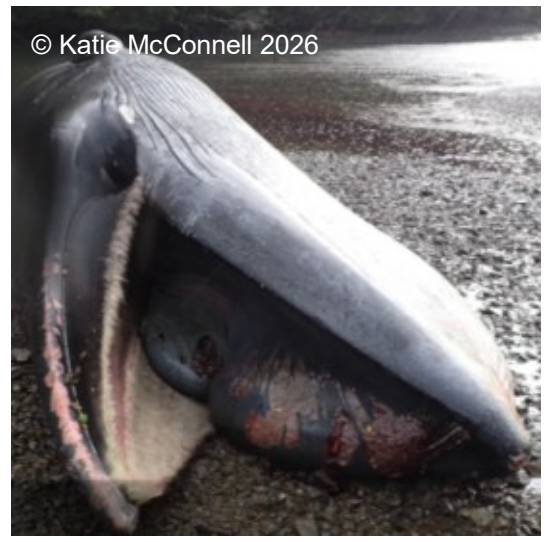

Attribute	Description	Satellite 0.15 m	Satellite 0.3 m	Satellite 0.5 m	Aerial	Ground
	sperm_whale<br>*Physeter macrocephalus*		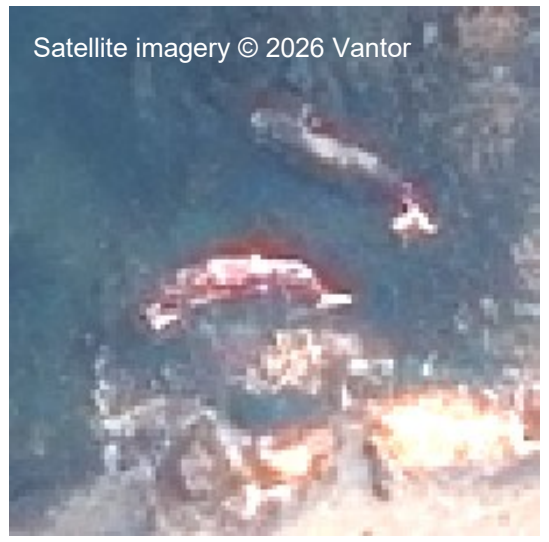	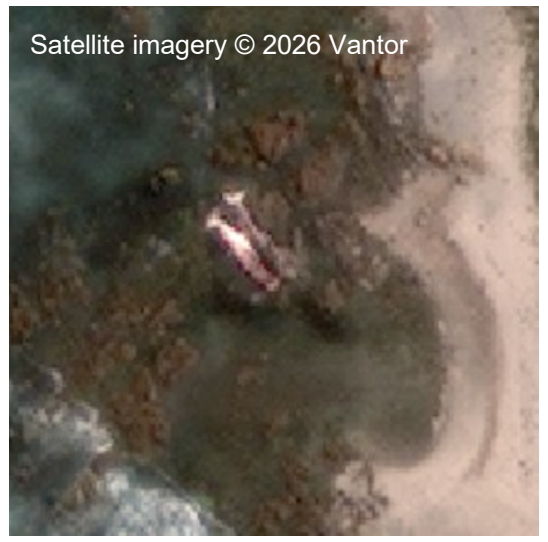	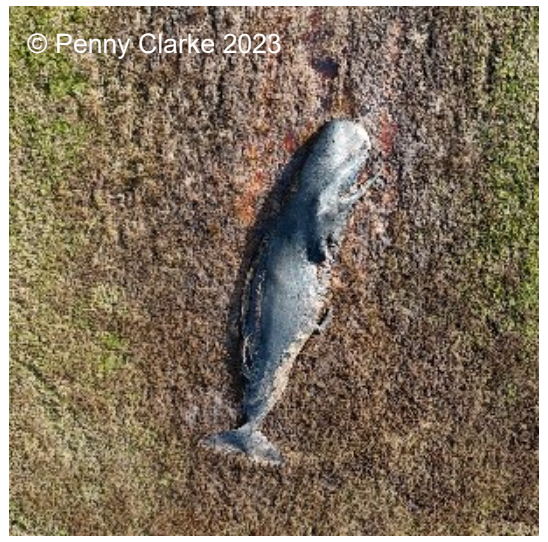	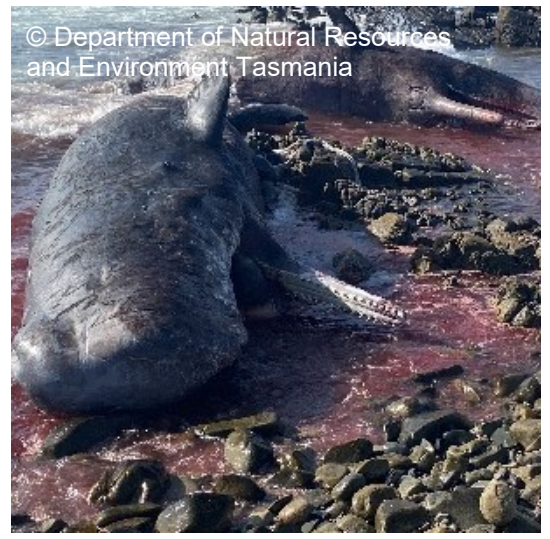
	long-finned_pilot_whale<br>*Globicephala melas edwardii*	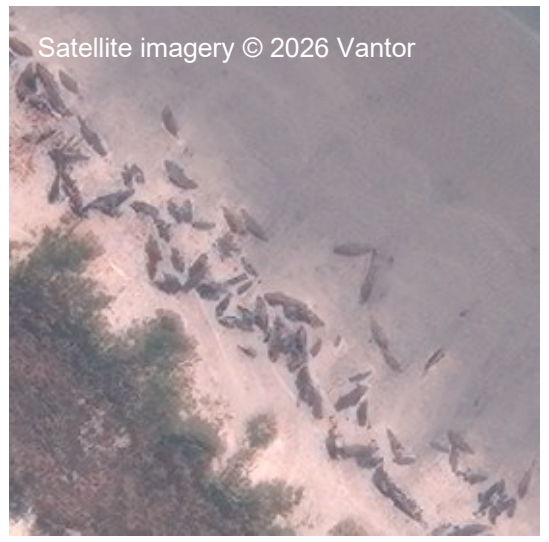	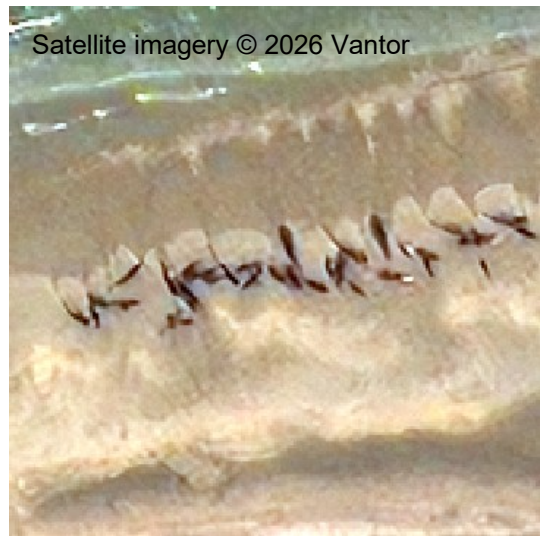	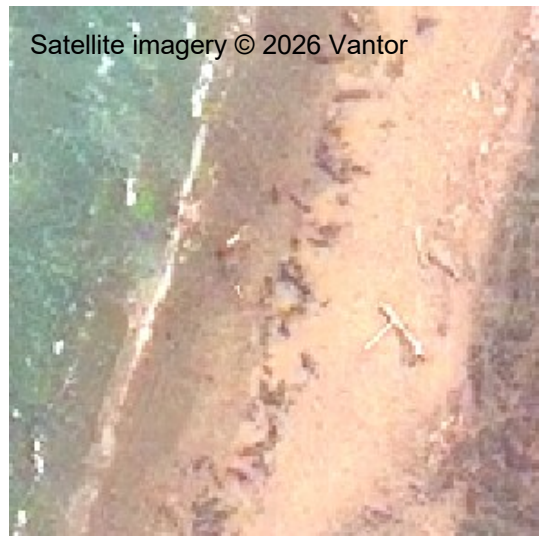	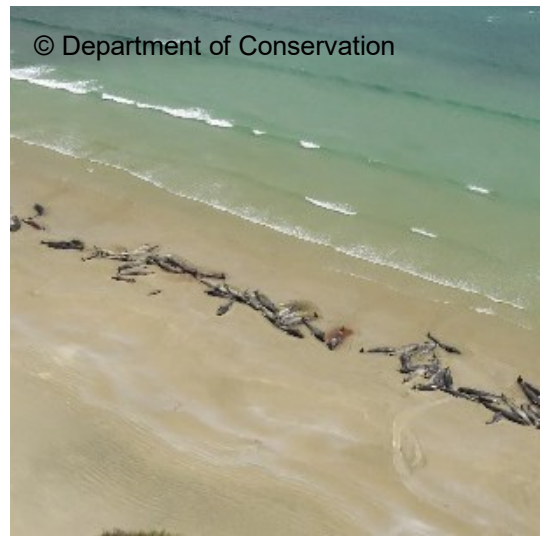	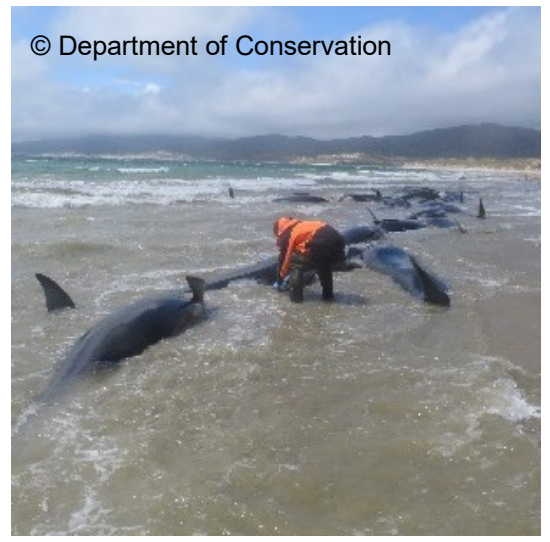
**colour**	black		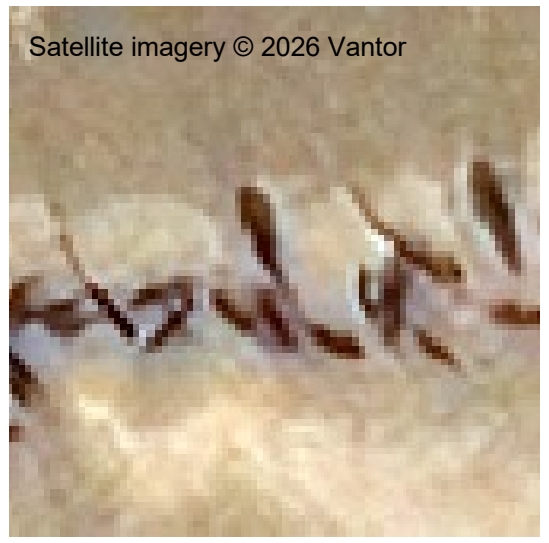			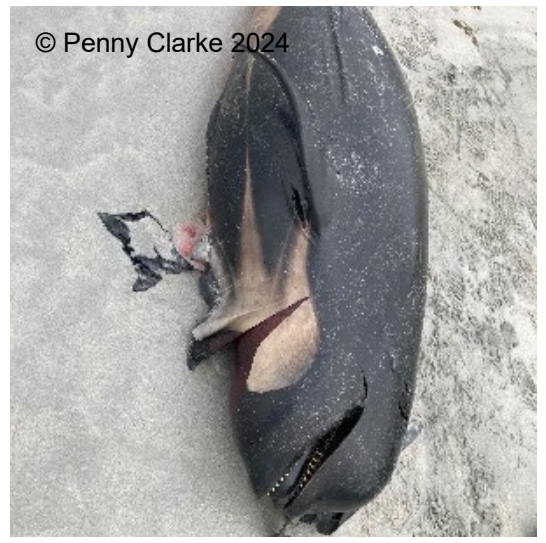

Attribute	Description	Satellite 0.15 m	Satellite 0.3 m	Satellite 0.5 m	Aerial	Ground
	brown			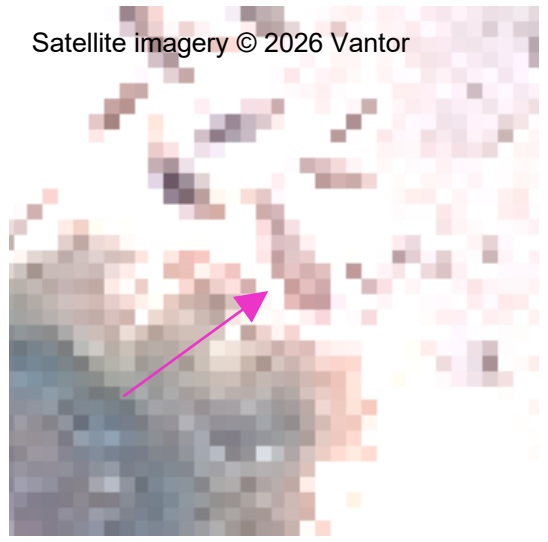		
	cream			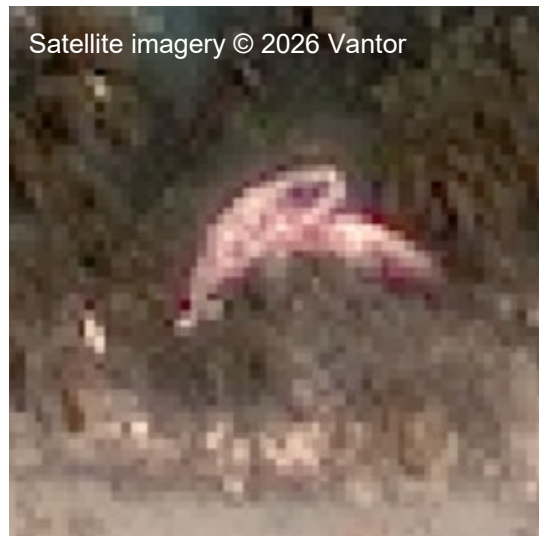 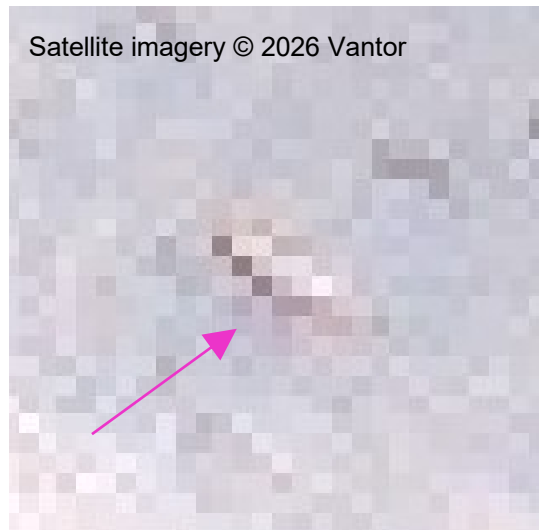		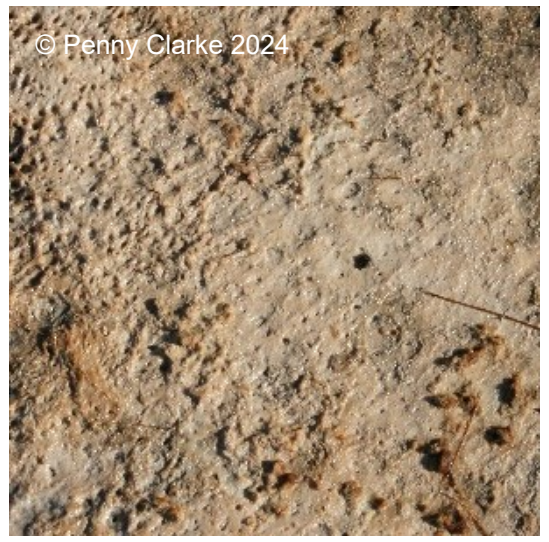
	grey_dark		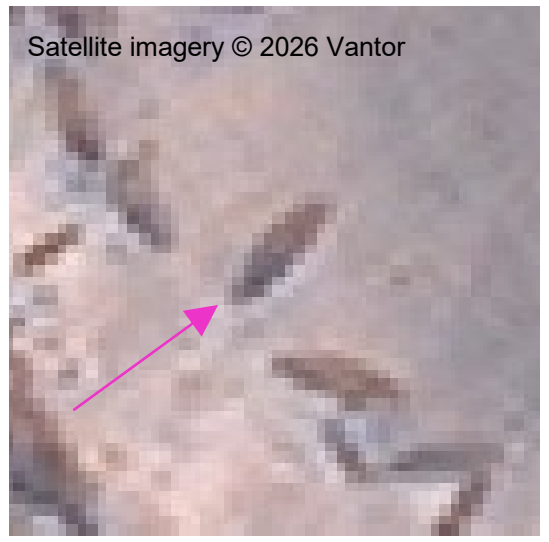	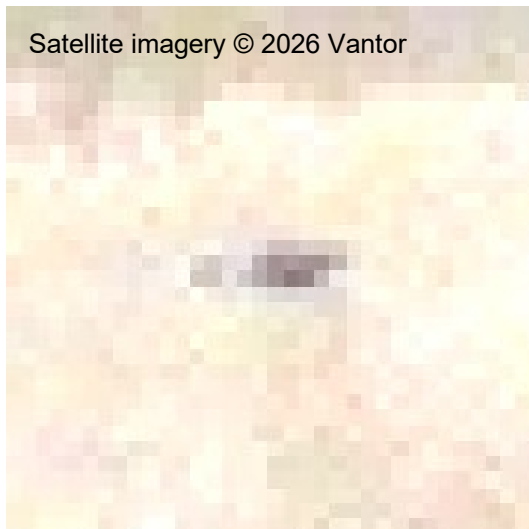	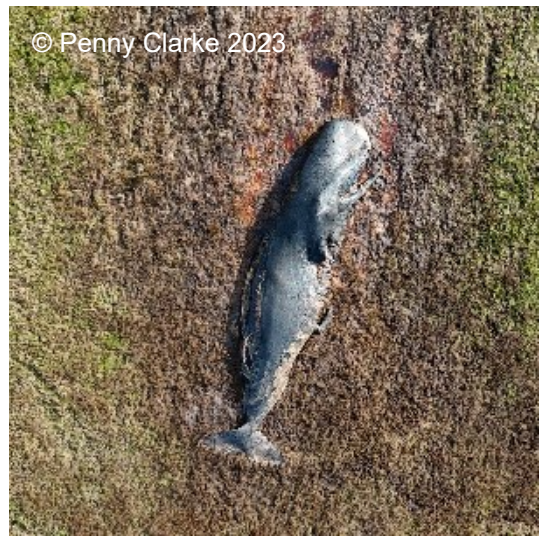	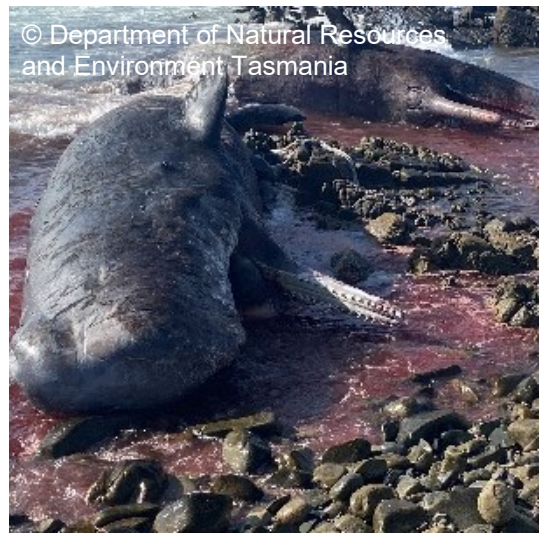

Attribute	Description	Satellite 0.15 m	Satellite 0.3 m	Satellite 0.5 m	Aerial	Ground
	grey_light				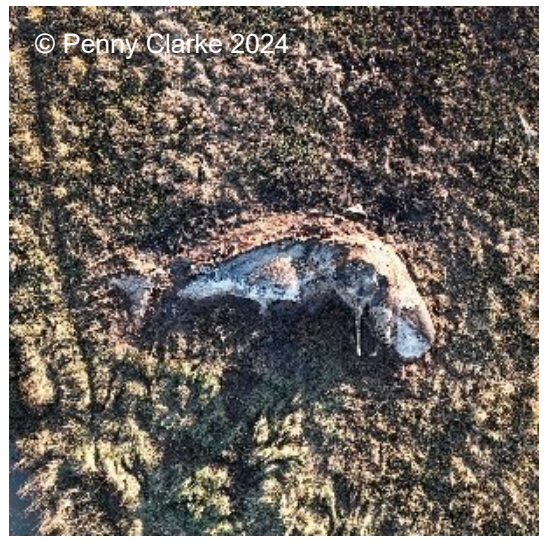	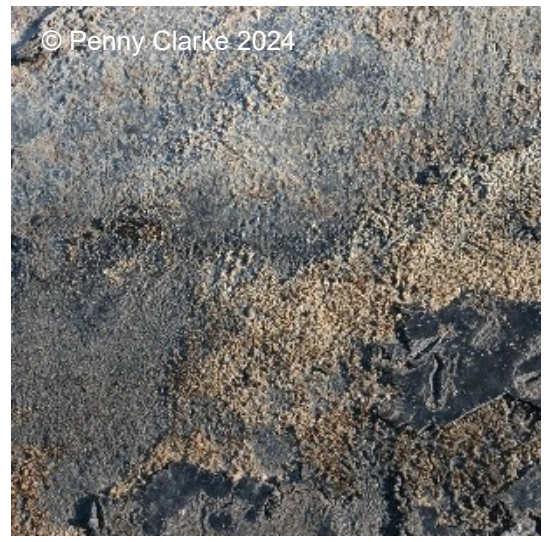
	orange					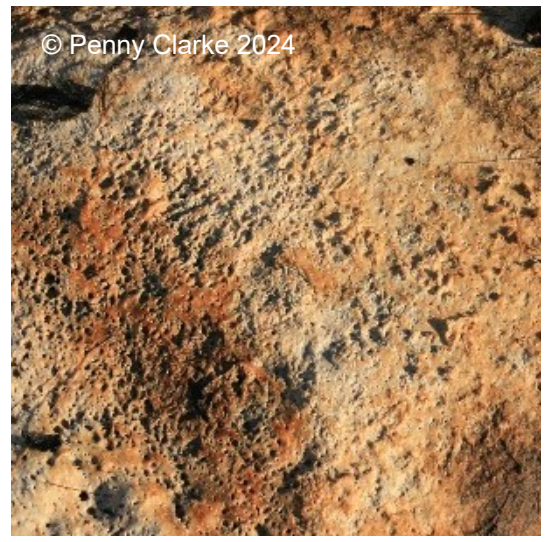
	pink			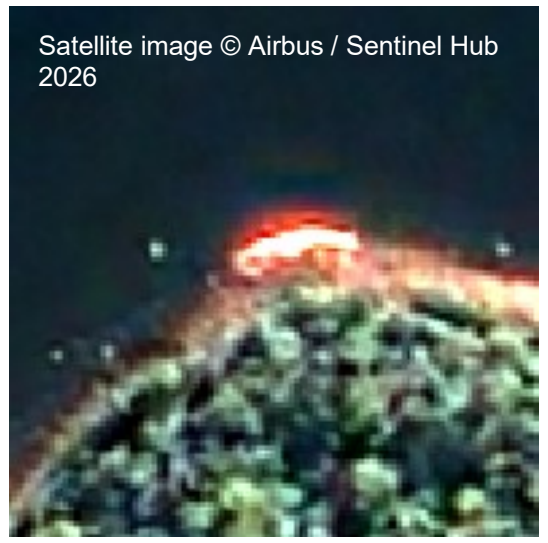		

Attribute	Description	Satellite 0.15 m	Satellite 0.3 m	Satellite 0.5 m	Aerial	Ground
	red		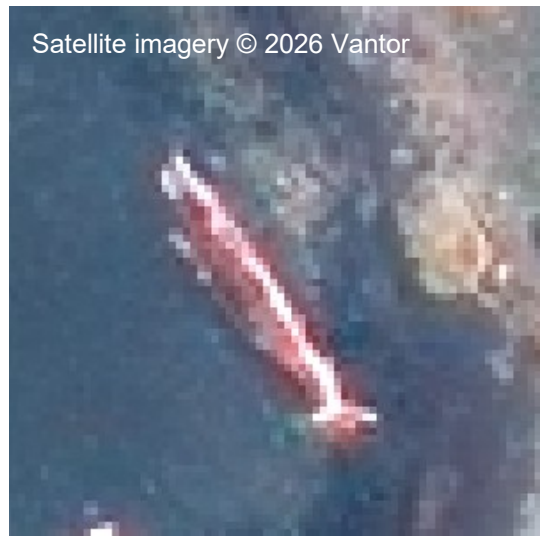			
	white		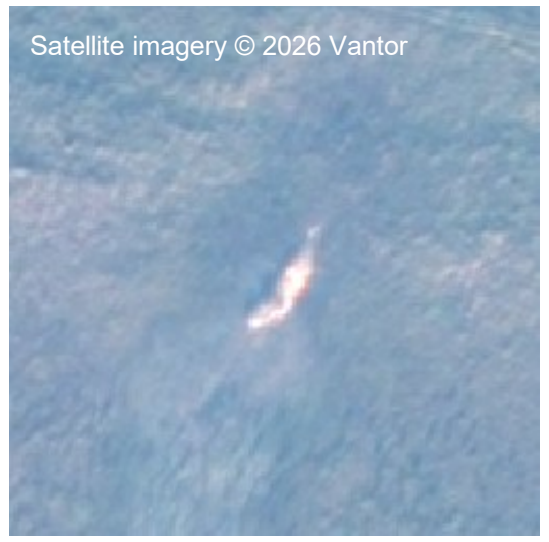 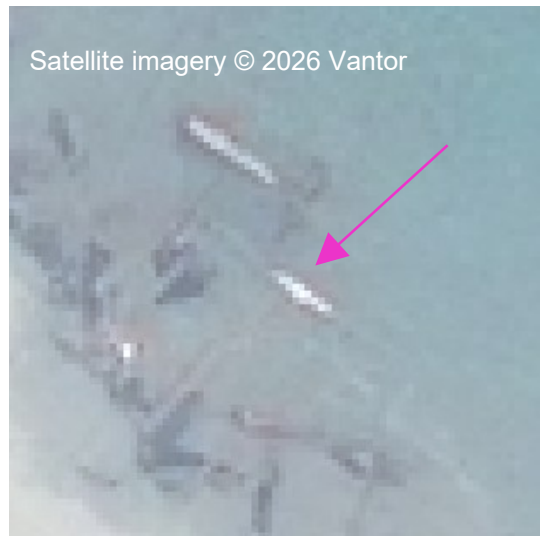			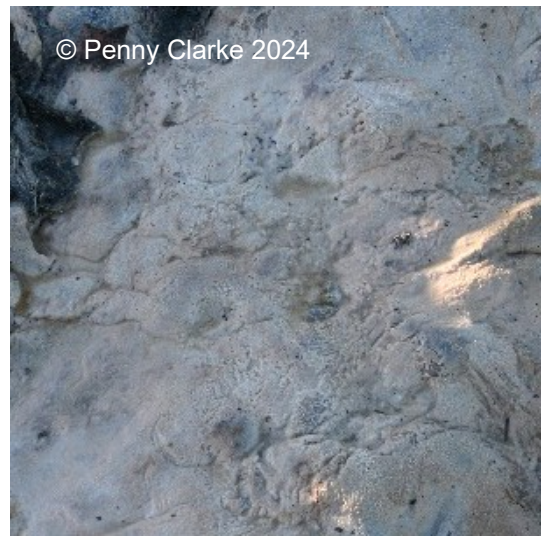 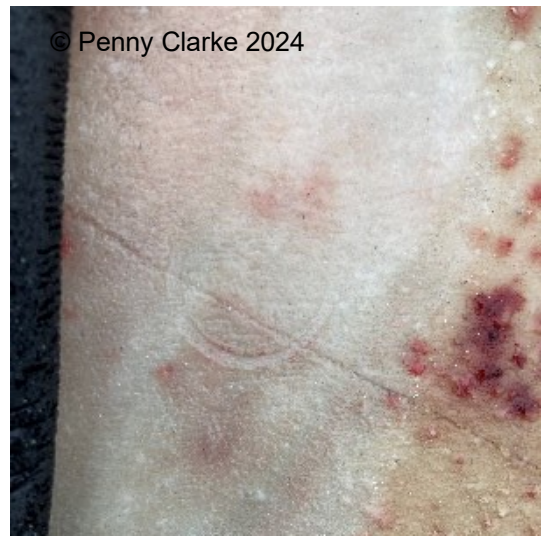
**body_ shape**	ellipsoid		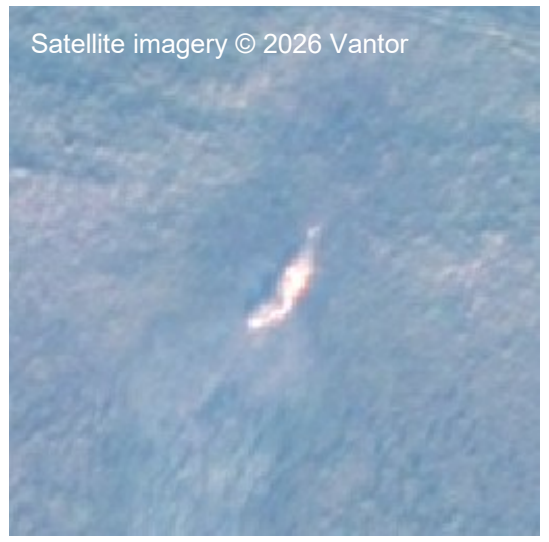	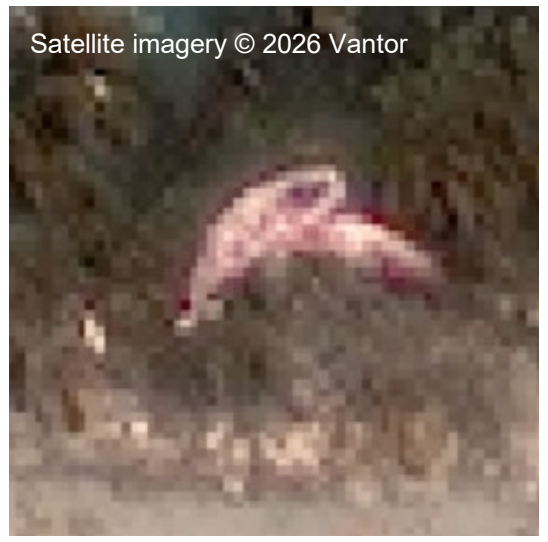	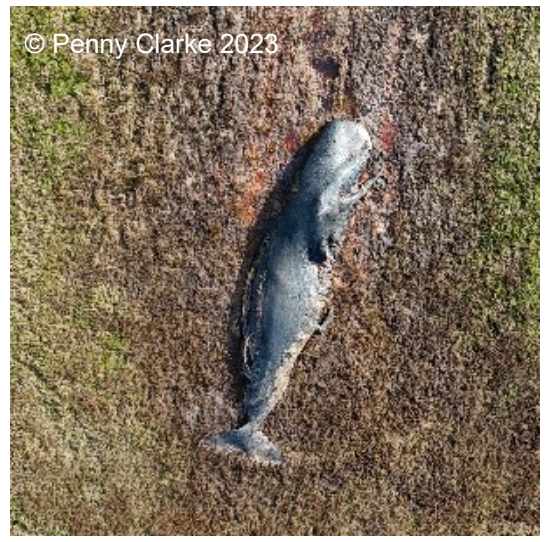	

Attribute	Description	Satellite 0.15 m	Satellite 0.3 m	Satellite 0.5 m	Aerial	Ground
			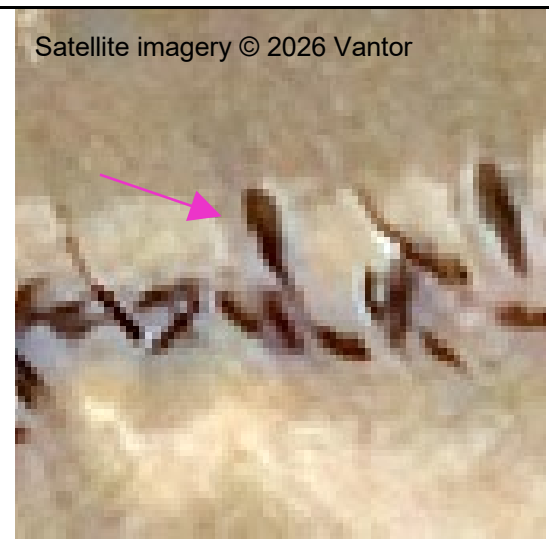	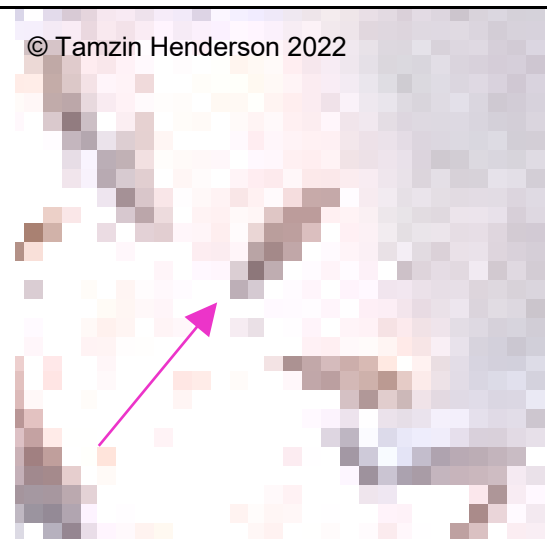	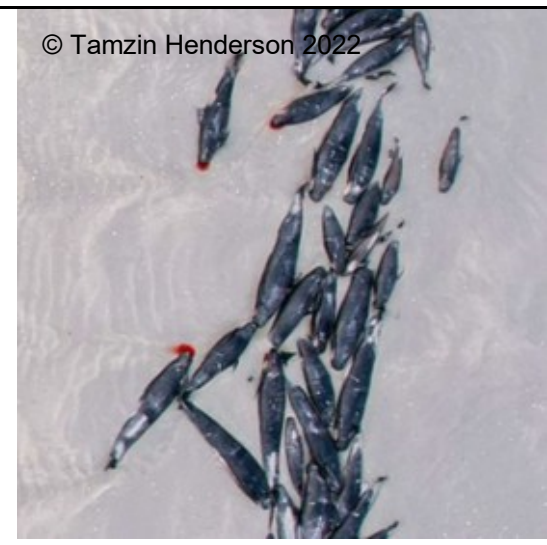	
	streamline		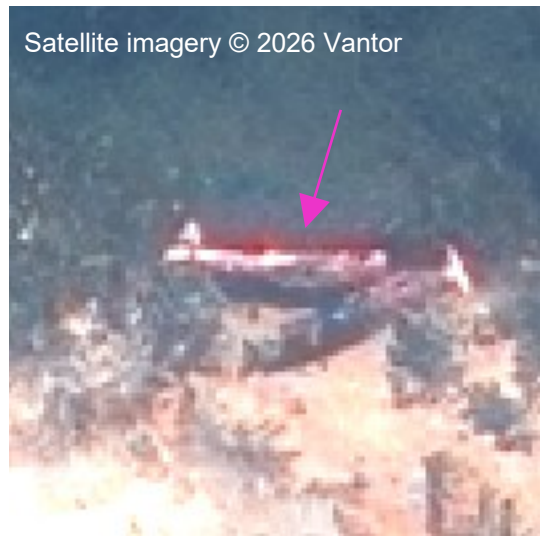			
	irregular_ellipsoid	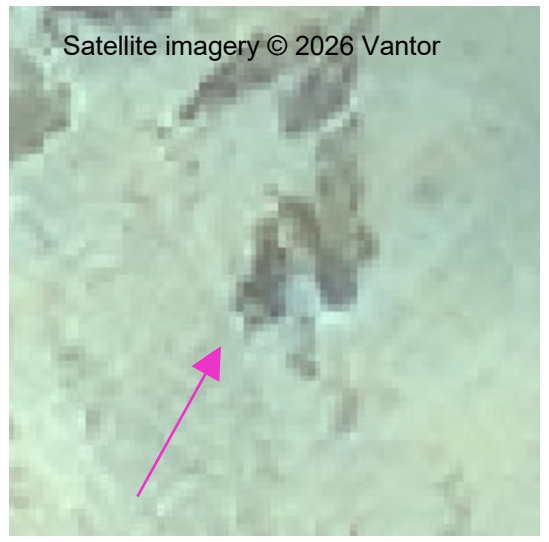	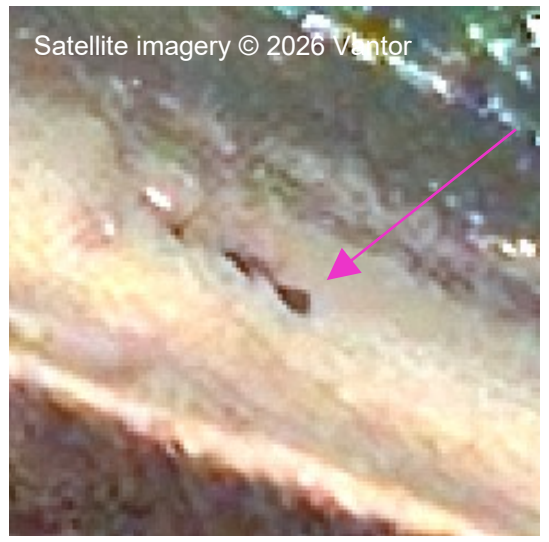	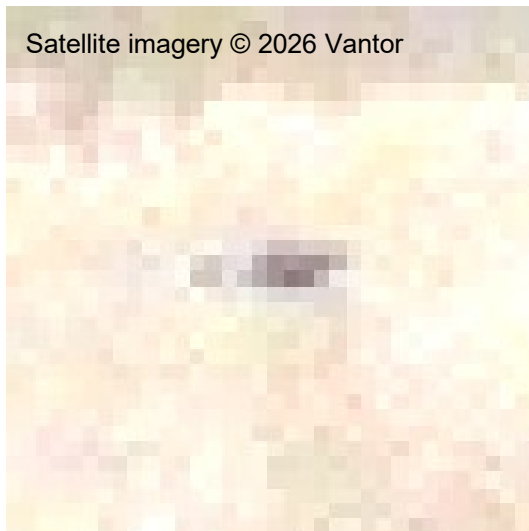		
	irregular_streamline					

Attribute	Description	Satellite 0.15 m	Satellite 0.3 m	Satellite 0.5 m	Aerial	Ground
	undefinable	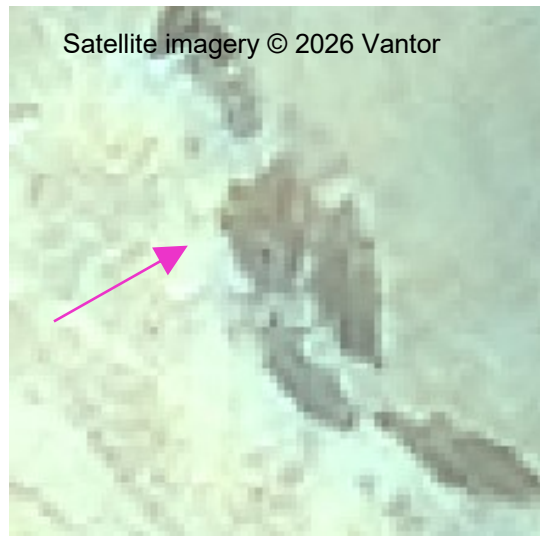	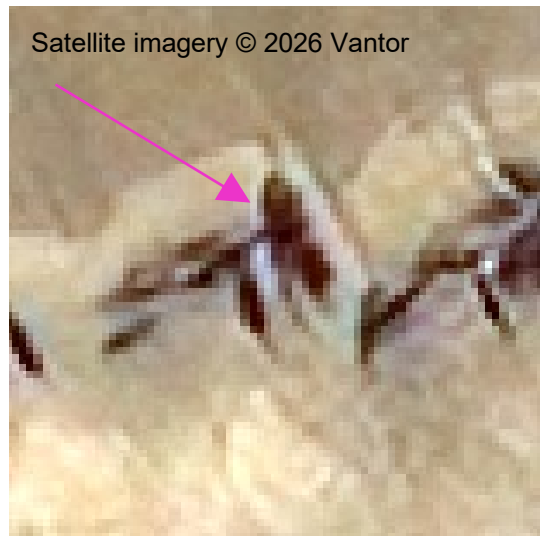	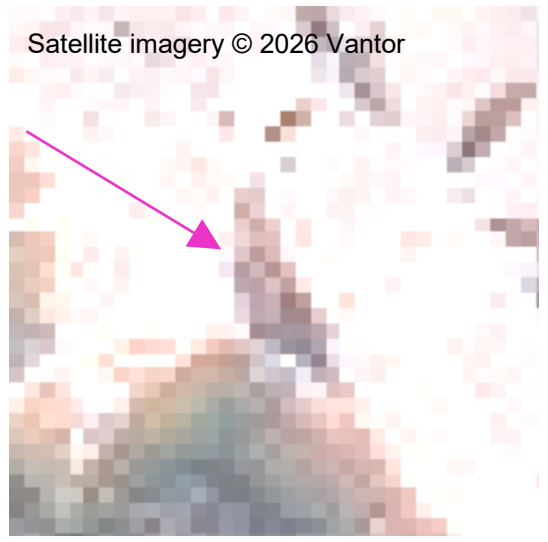		
**flippers**	yes					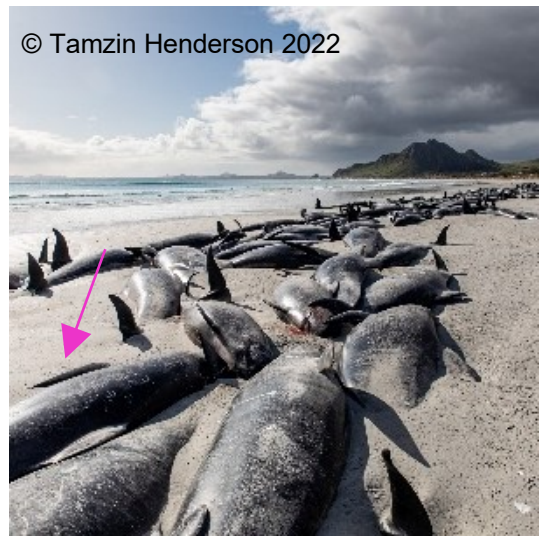 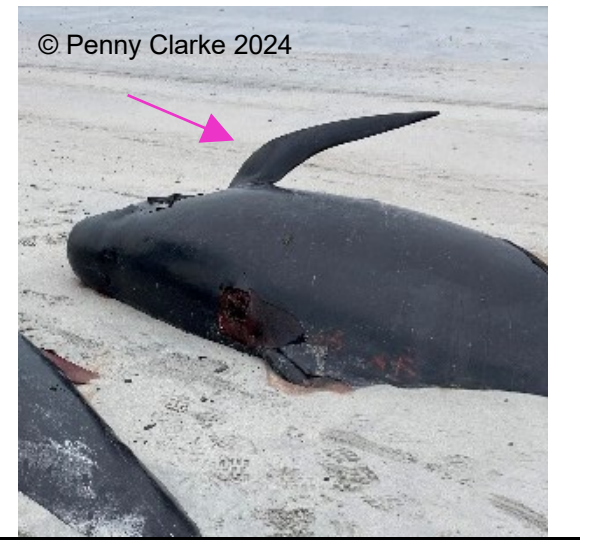
	no		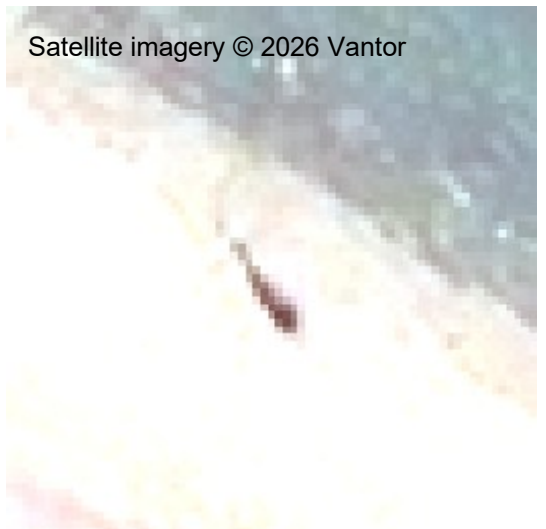	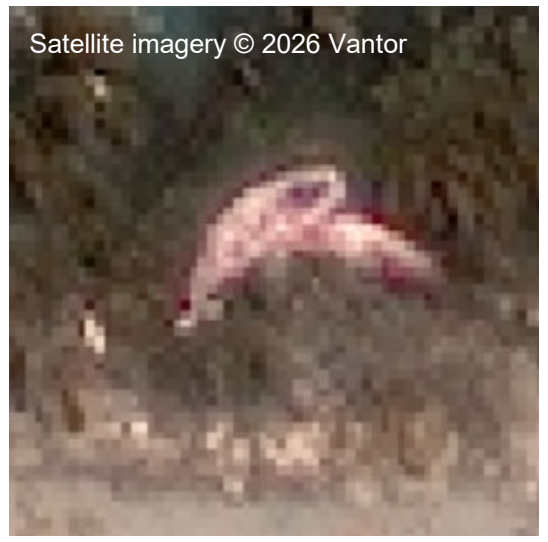		

Attribute	Description	Satellite 0.15 m	Satellite 0.3 m	Satellite 0.5 m	Aerial	Ground
	maybe					
**fluke**	yes			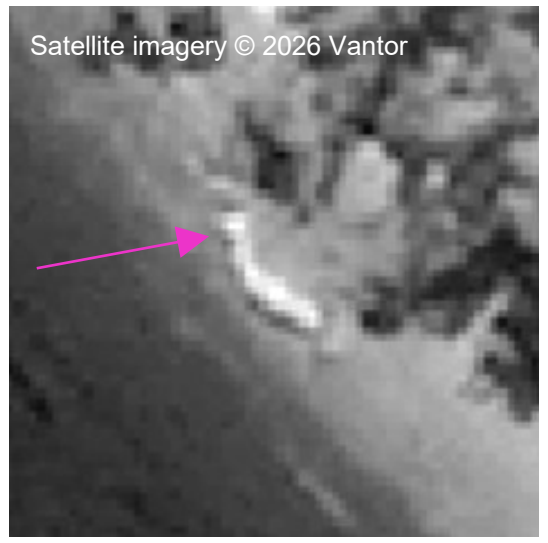	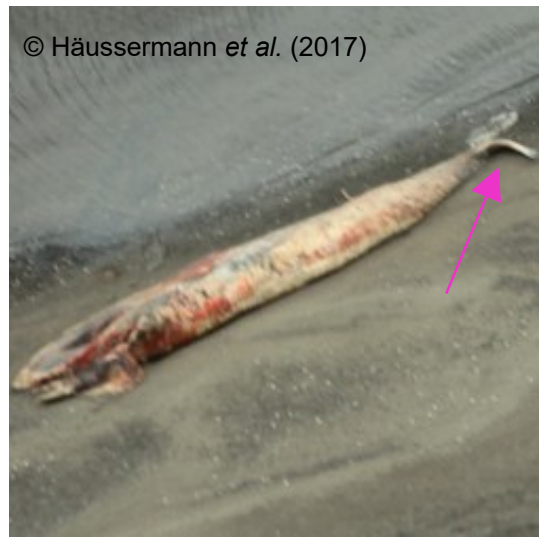	
				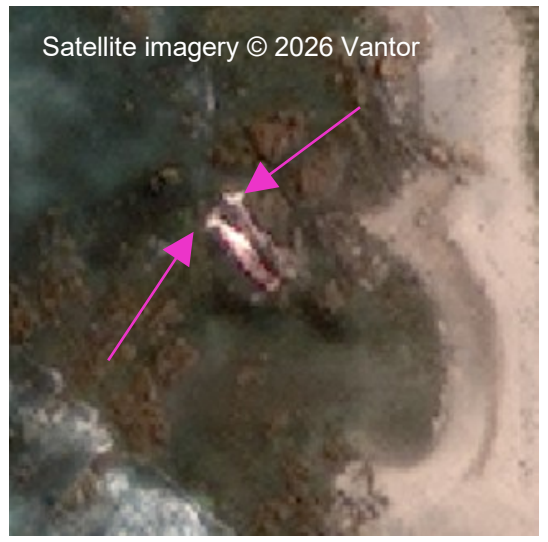	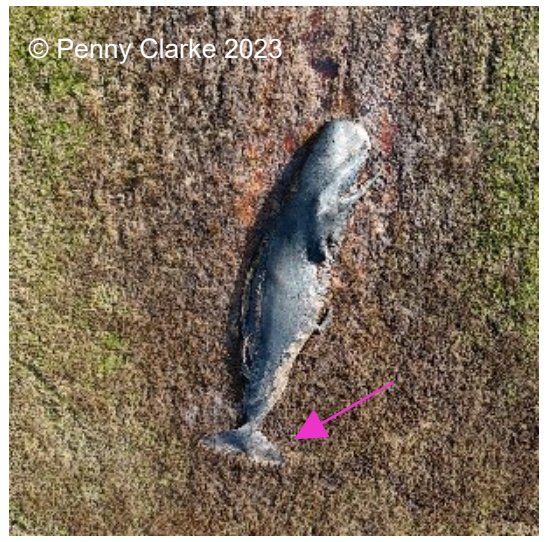	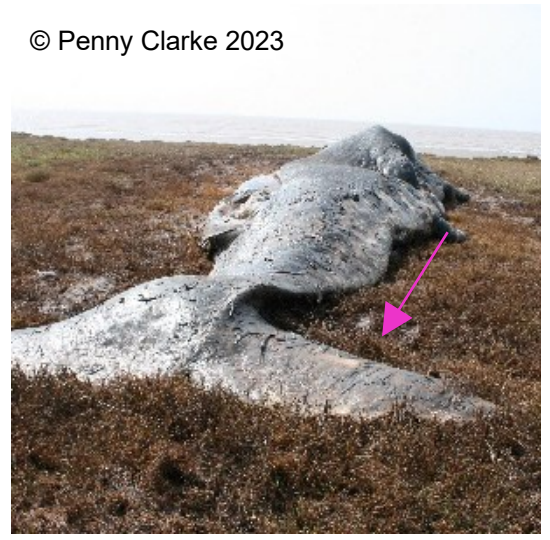
						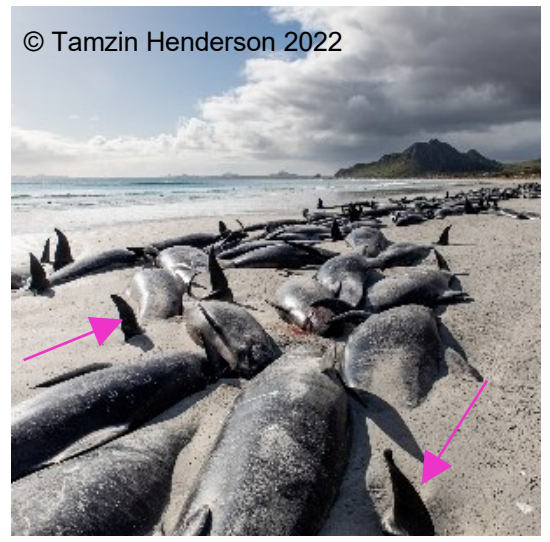

Attribute	Description	Satellite 0.15 m	Satellite 0.3 m	Satellite 0.5 m	Aerial	Ground
	no			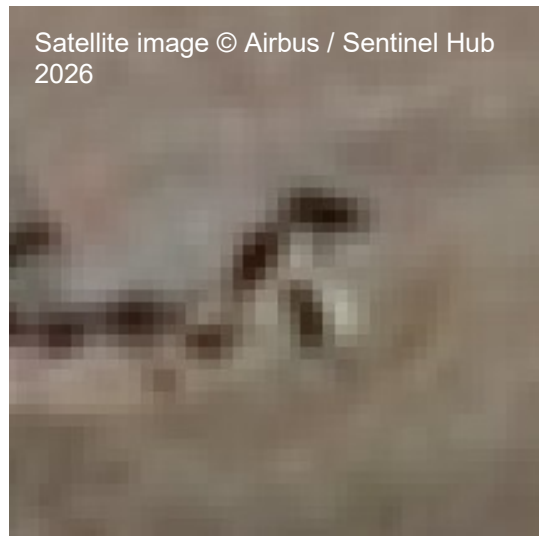		
	maybe		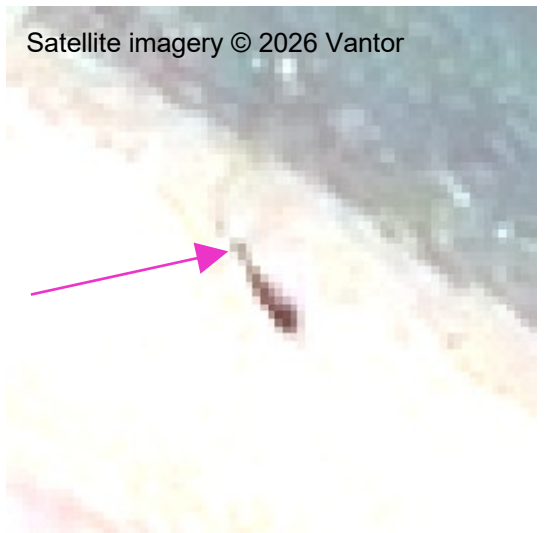			
**slick/bld**	yes		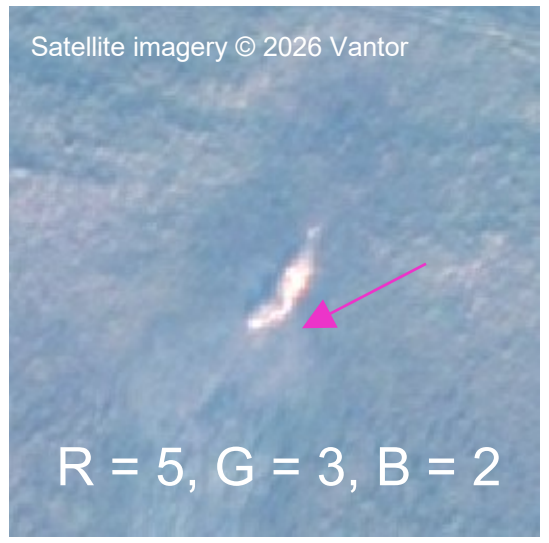<br>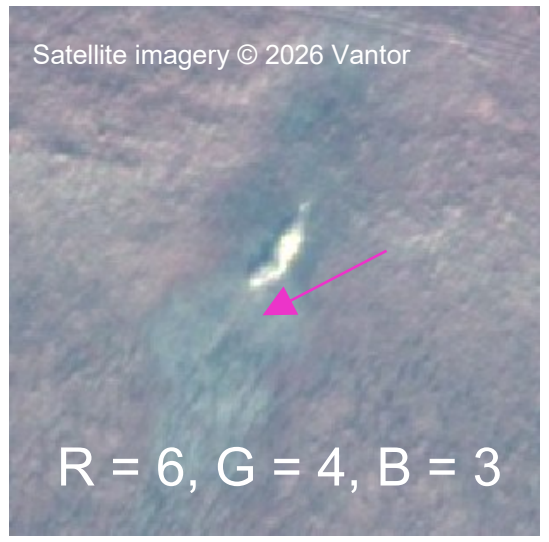	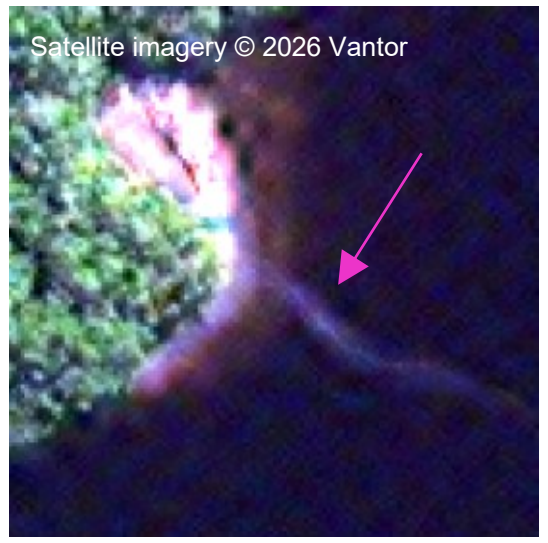		

<table>
<tr><th>Attribute</th><th>Description</th><th>Satellite 0.15 m</th><th>Satellite 0.3 m</th><th>Satellite 0.5 m</th><th>Aerial</th><th>Ground</th></tr>
<tr><td></td><td>no</td><td></td><td></td><td>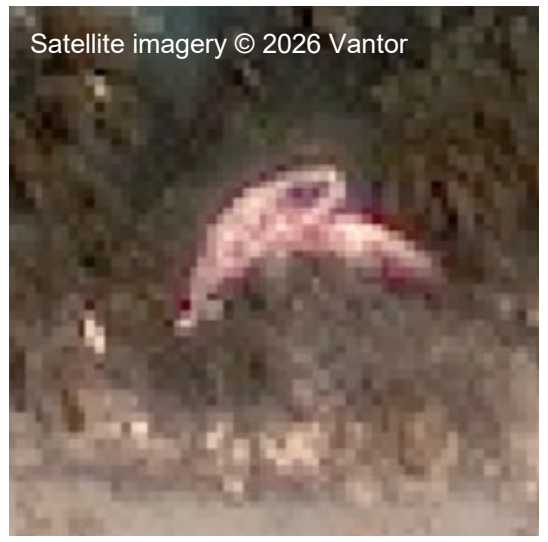
</td><td></td><td></td></tr>
<tr><td></td><td>maybe</td><td></td><td>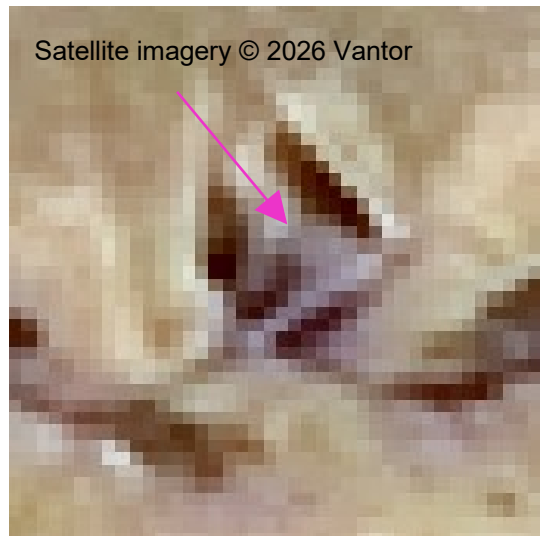
</td><td>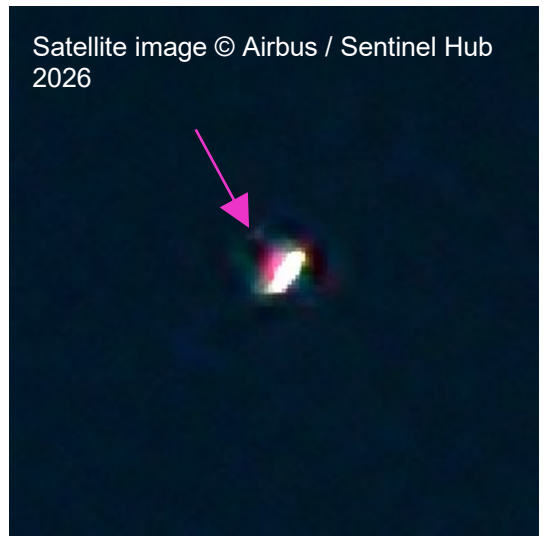
</td><td></td><td></td></tr>
<tr><td>group</td><td>individual</td><td></td><td></td><td>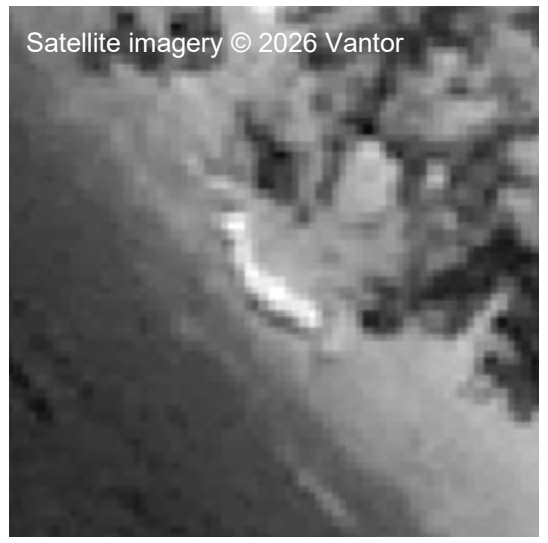
</td><td></td><td></td></tr>
</table>

Attribute	Description	Satellite 0.15 m	Satellite 0.3 m	Satellite 0.5 m	Aerial	Ground
	2_to_5			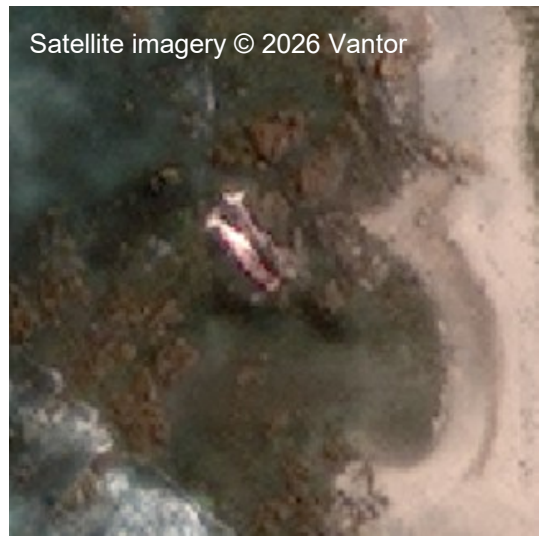		
	6_to_10			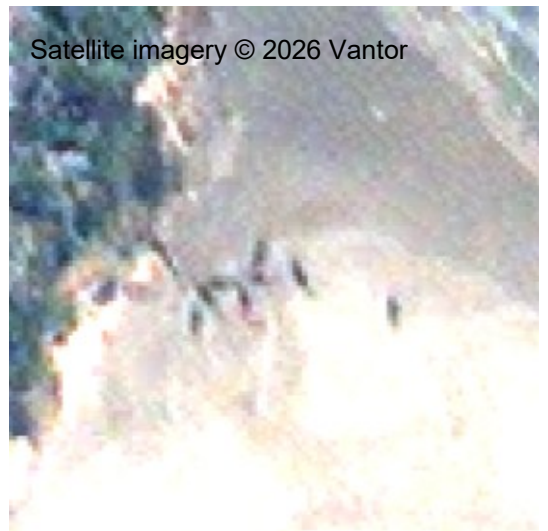		
	11_to_50					
	51_to_100			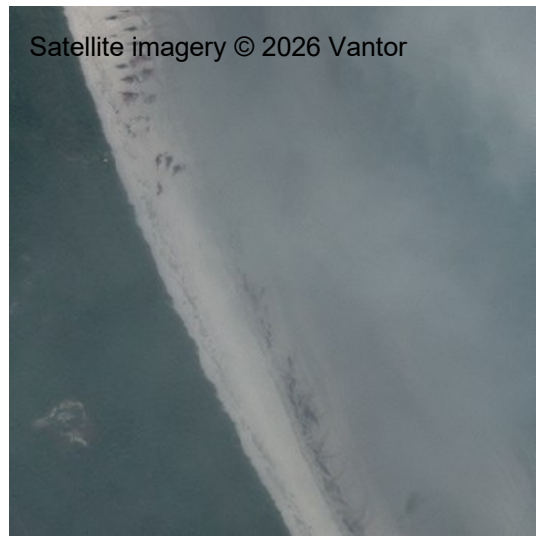	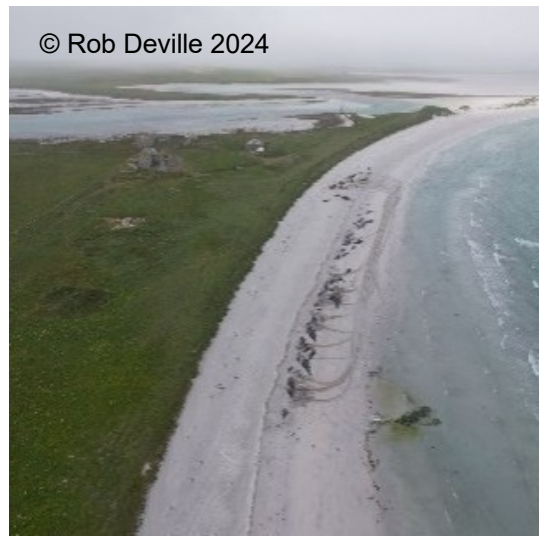	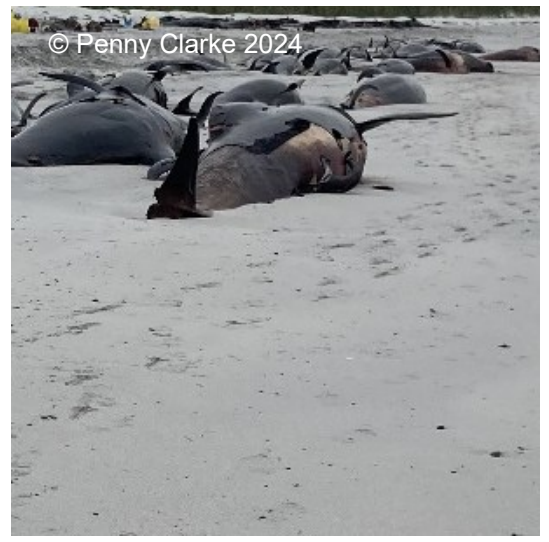

Attribute	Description	Satellite 0.15 m	Satellite 0.3 m	Satellite 0.5 m	Aerial	Ground
	101_to_200		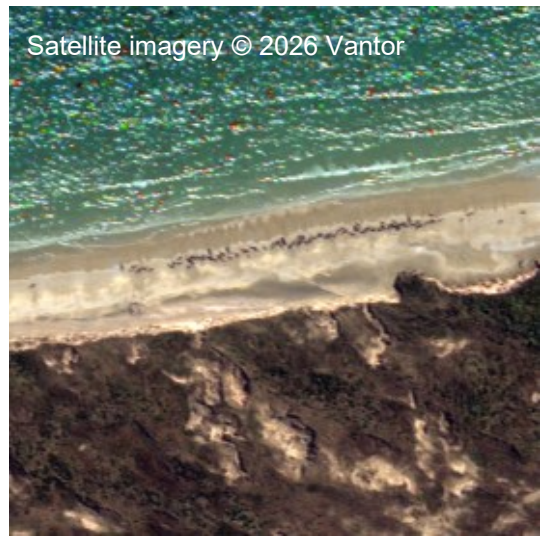	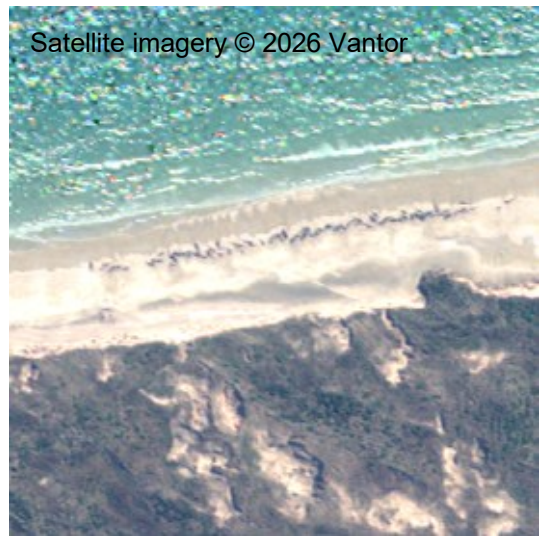	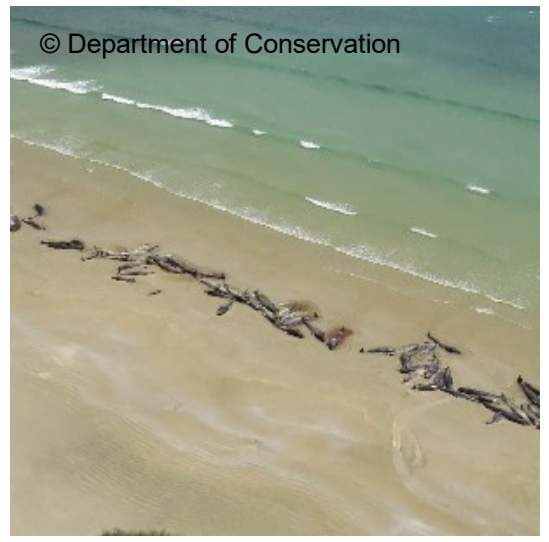	
	201_to_500	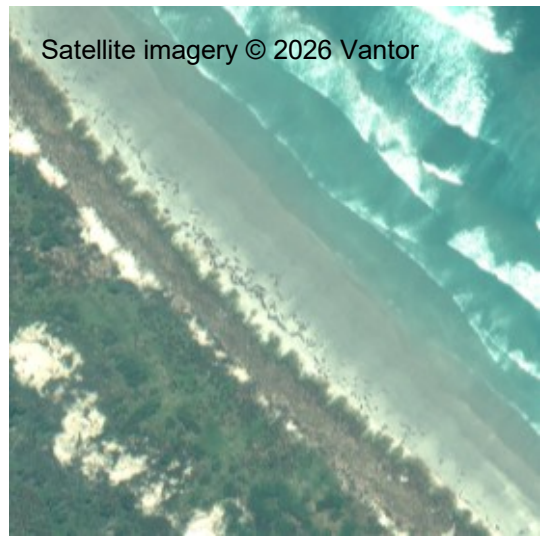	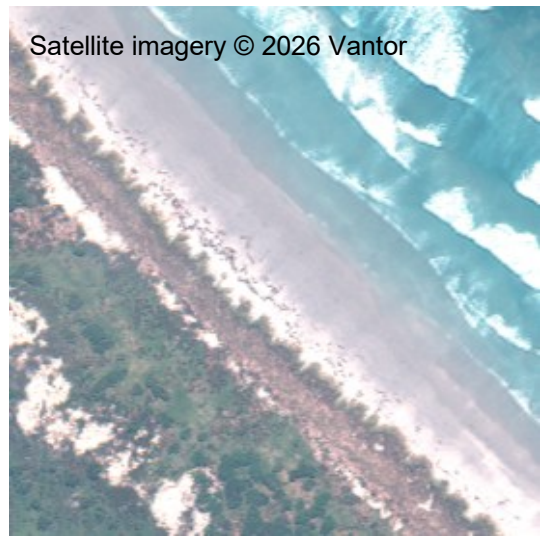	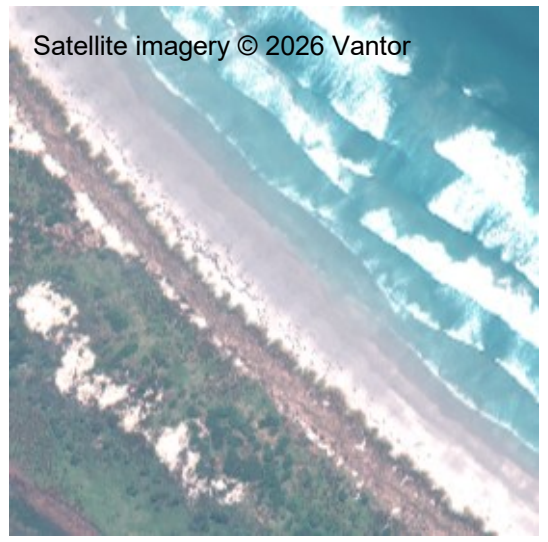	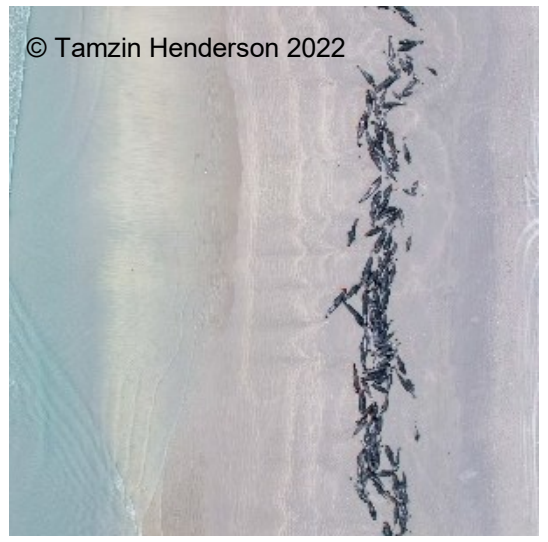	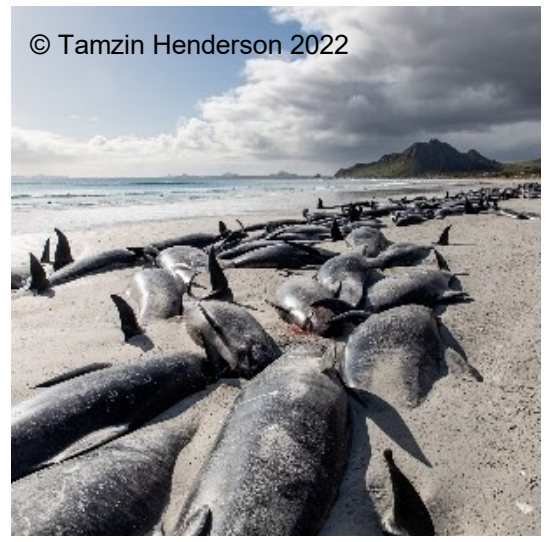
	501+					

****Please note: the following attributes are environmental variables and requires the conditions at the point feature / annotation specifically, and not the whole image****

Cloud Cover (cloud_cov)

Table S3.5: Satellite image examples of the cloud cover values associated with the standardised attribute 'cloud_cov' that could impact feature detection.

skc_sky_clear	few_traces	sct_scattered	bkn_broken	ovc_overcast
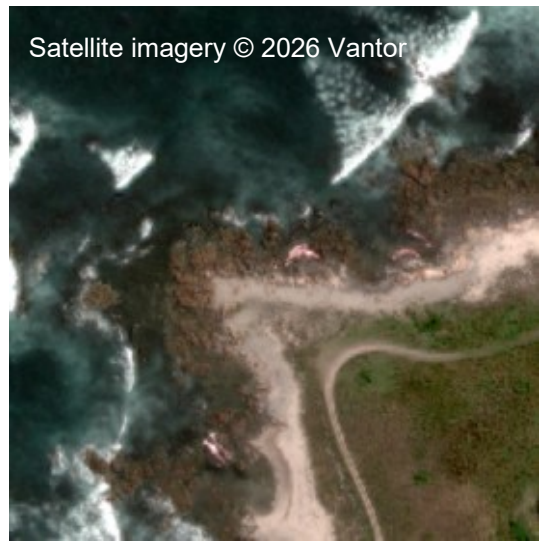	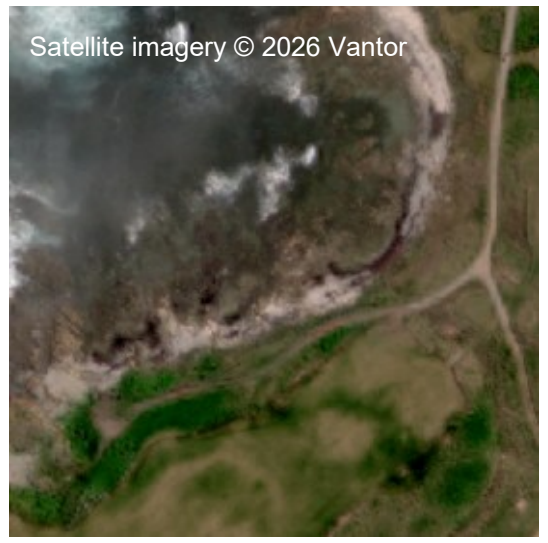	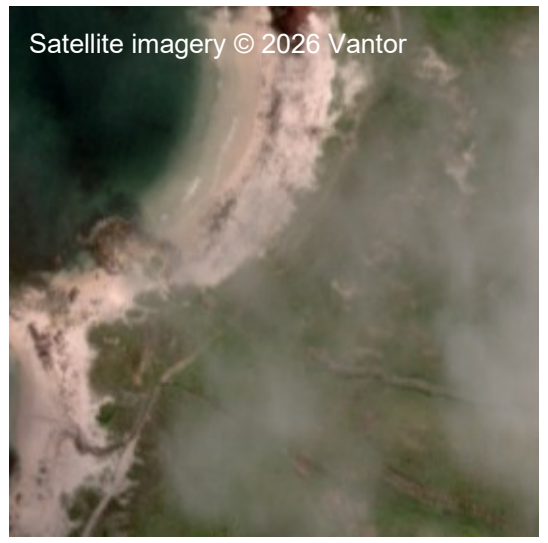	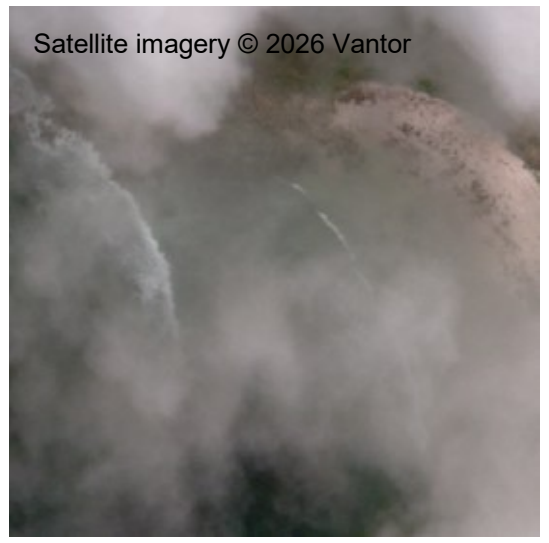	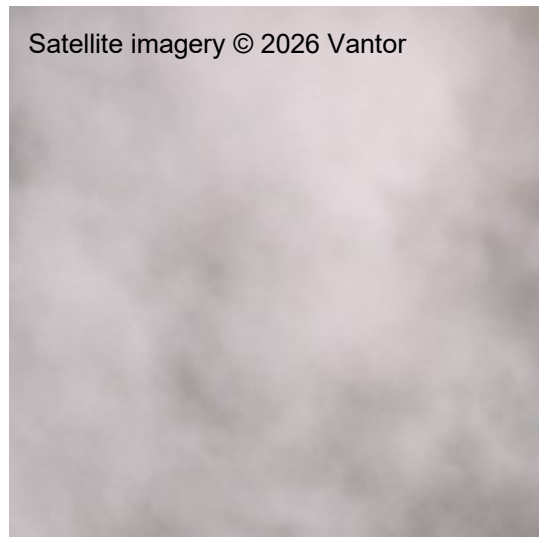

Cloud Thickness (cloud_thic)

Table S3.6: Satellite image examples of the different cloud thickness values associated with the standardised attribute 'cloud_thic' that could impact feature detection.

none	thin	medium_thin	thick
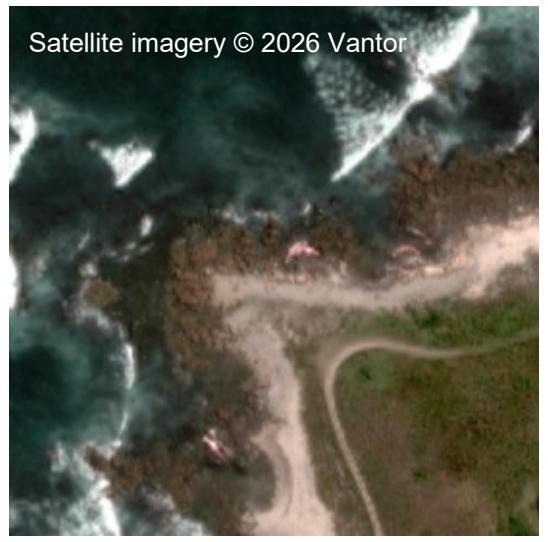	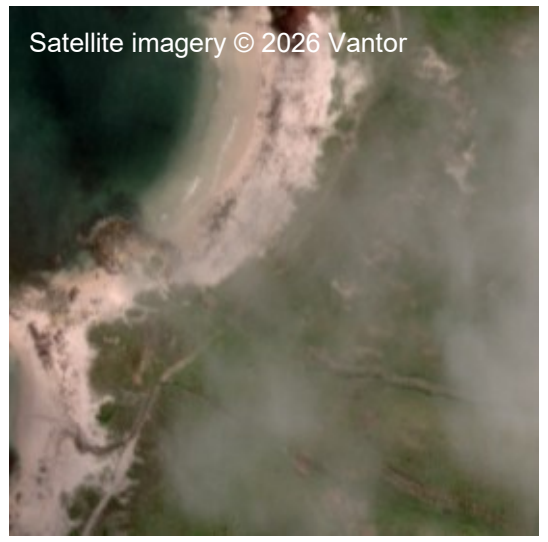	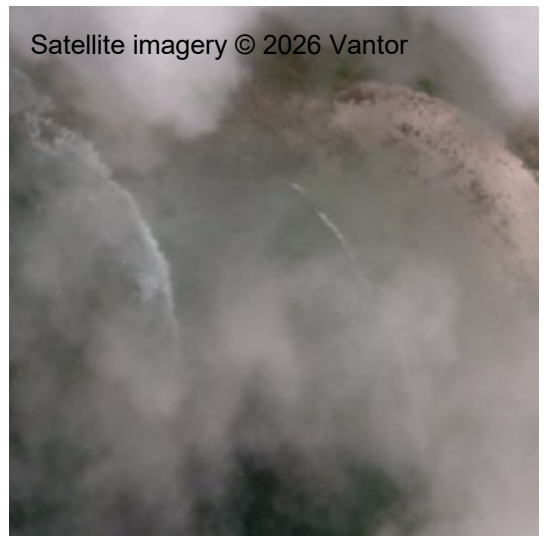	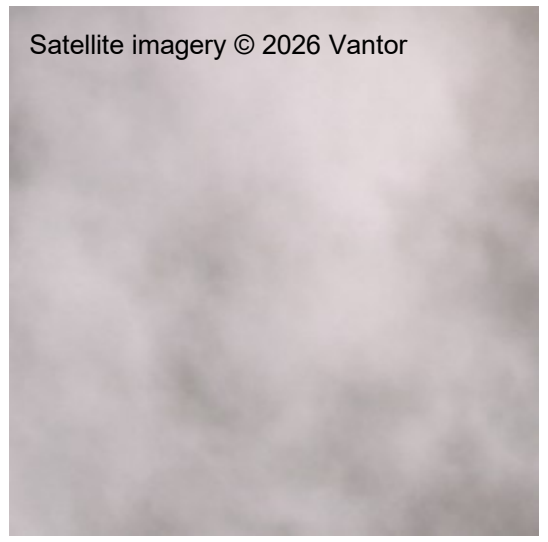

Environment (env)

Table S3.7: Satellite, aerial and ground image examples of the different abiotic environment values associated with the standardised attribute 'env' that a 'stranded_cetacean' or 'floating_carcass' may be found in.

	Satellite	Aerial	Ground
**bedrock**	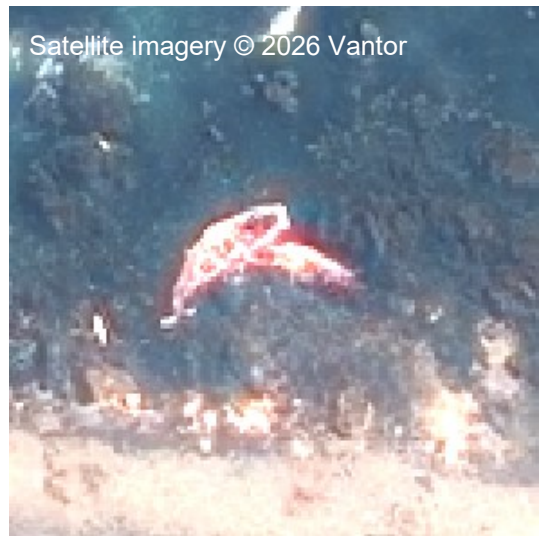		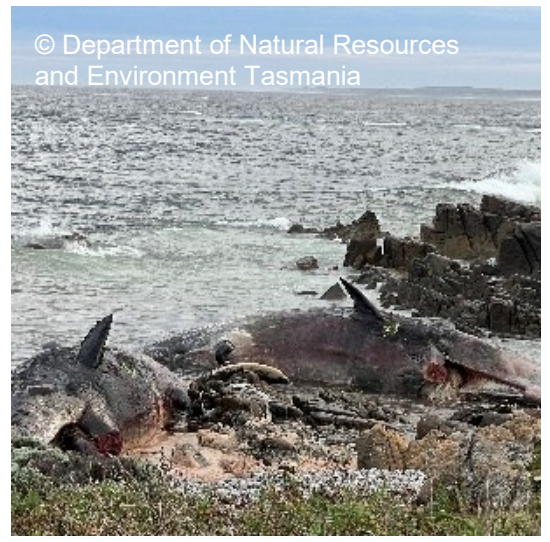
**cobble_beach**			
**estuary_mud_flat**			
**gravel_beach**			
**man_made_structure**	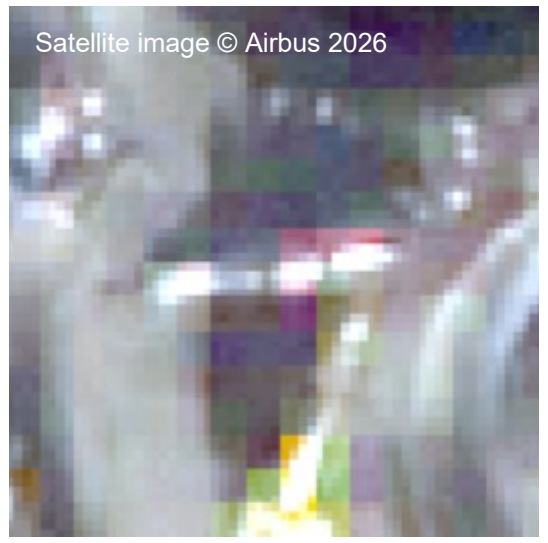		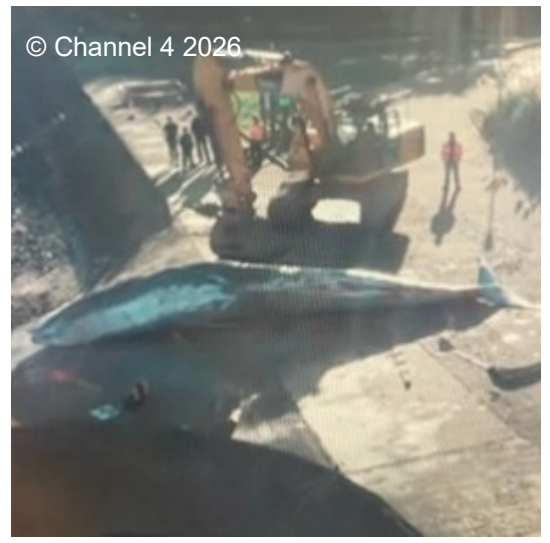
**mangrove**			

**marsh_brackish**	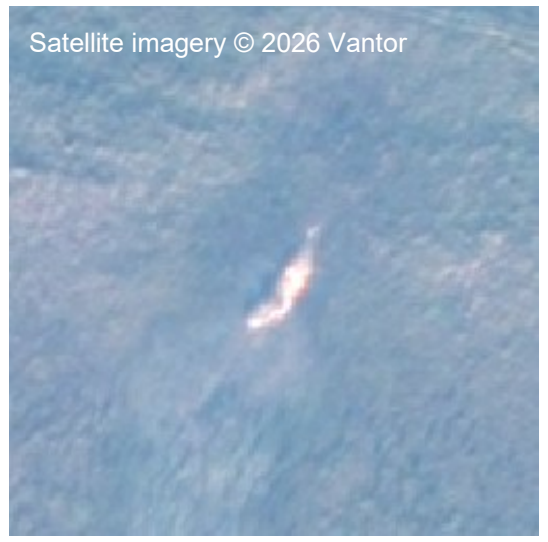	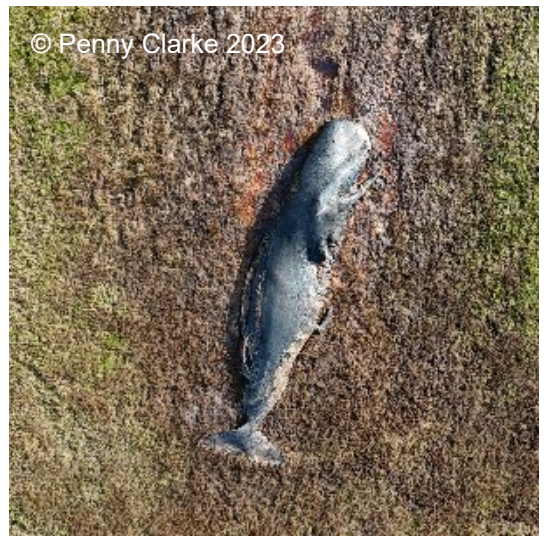	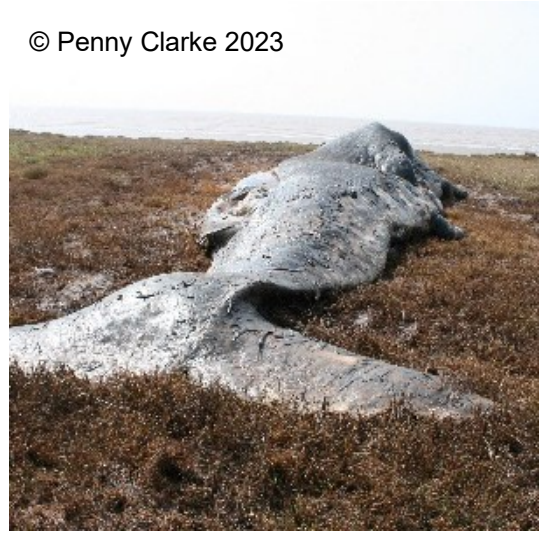
**marsh_freshwater**			
**nearshore_waters**			
**offshore_waters**			
**sand_beach_dark**	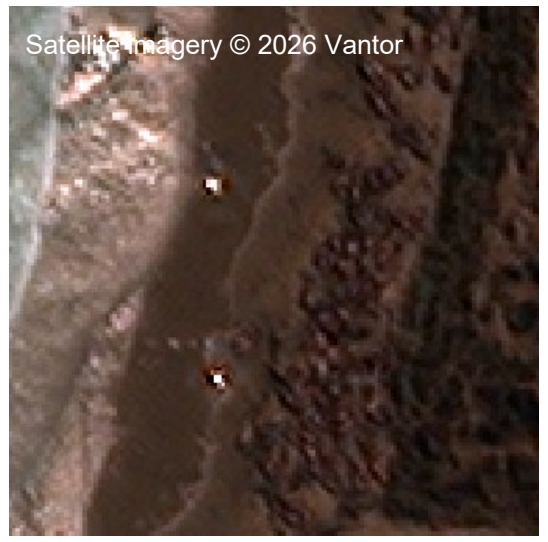		
**sand_beach_light**	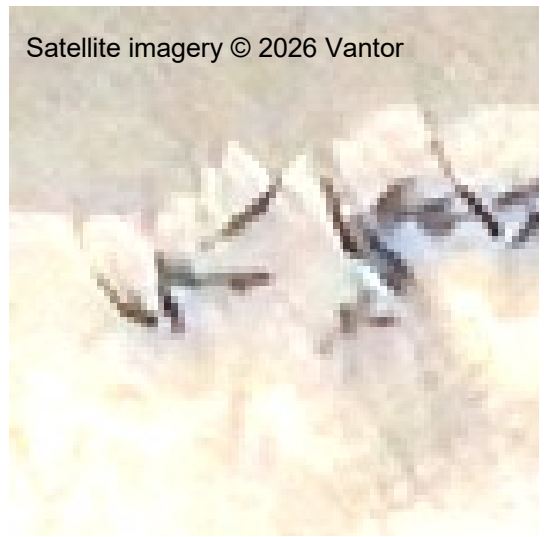		

**shell_beach**			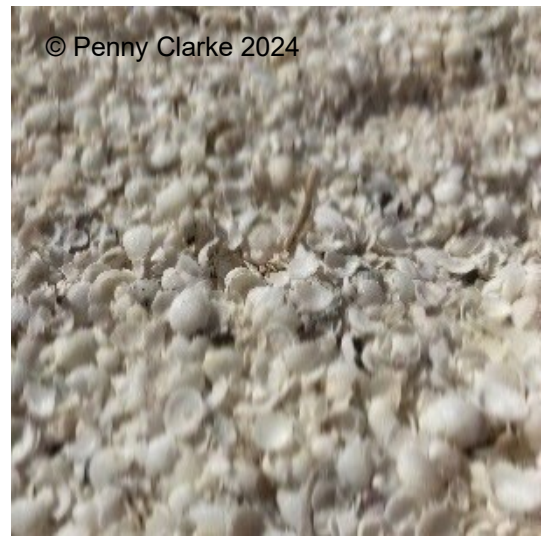
**vegetated_trees**	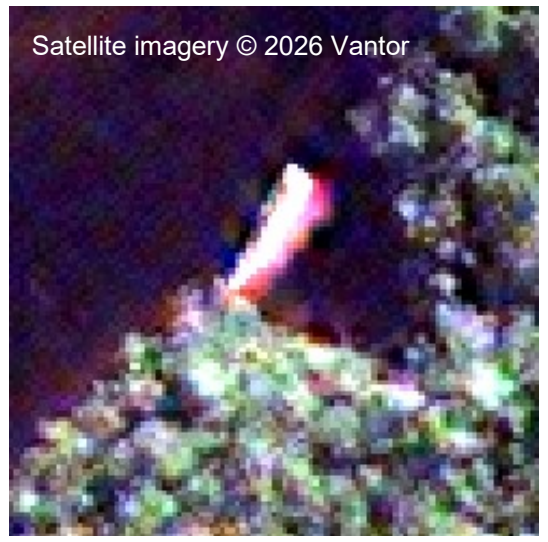	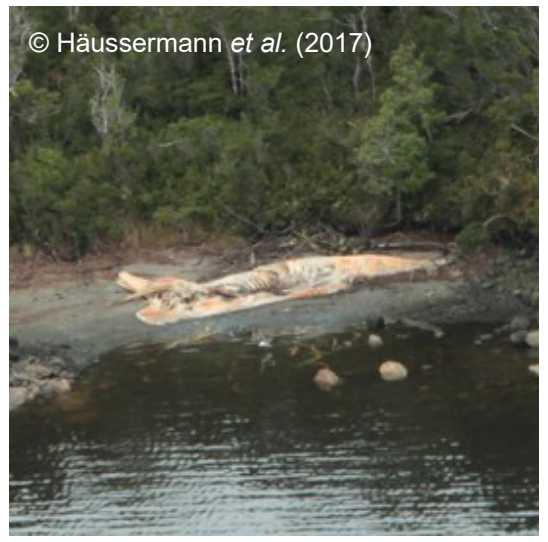	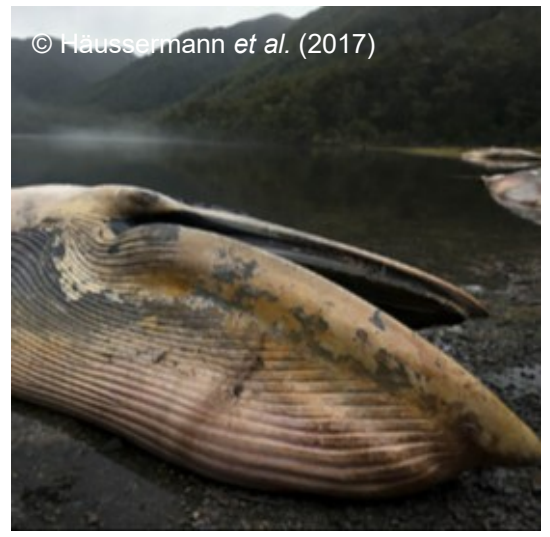
**vegetated_wetland**			

Image brightness (img_bright)

Table S3.8: Satellite image examples of the different image brightness values associated with the standardised attribute 'img_bright' that could impact feature detection.

**bright**	**moderate**	**dark**
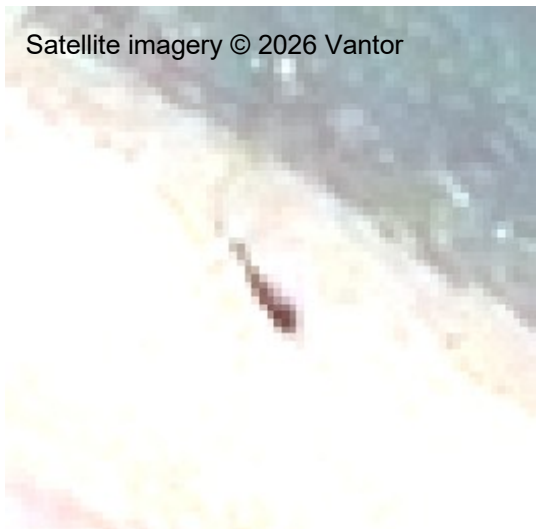	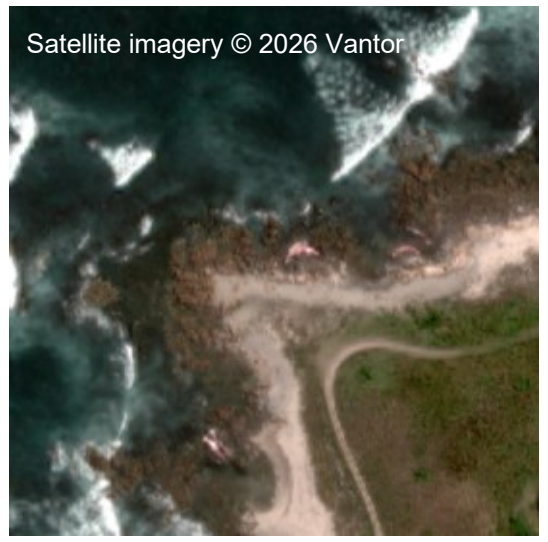	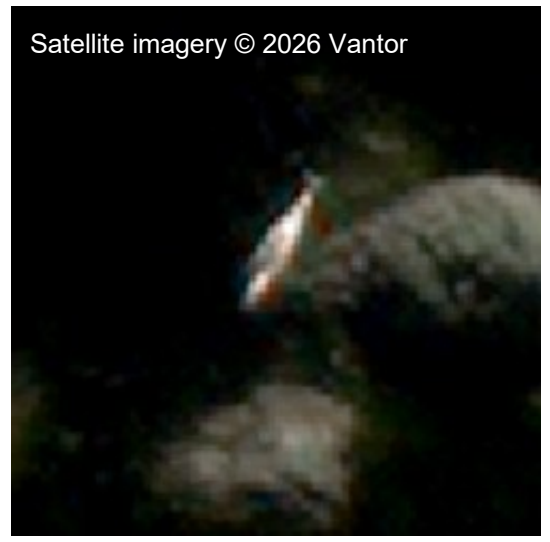

Shade (shade)

Table S3.9: Satellite image examples of the different shade values associated with the standardised attribute 'shade' that could impact feature detection.

full_shade	partial_shade	no_shade
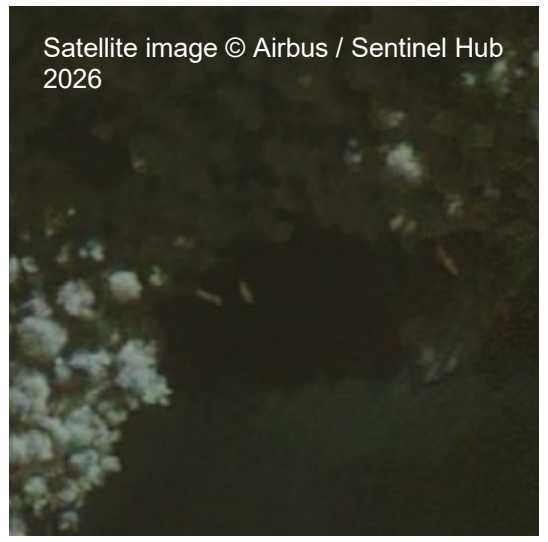 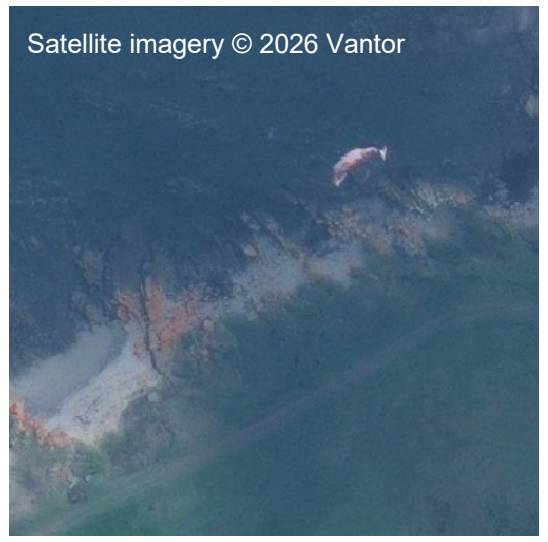	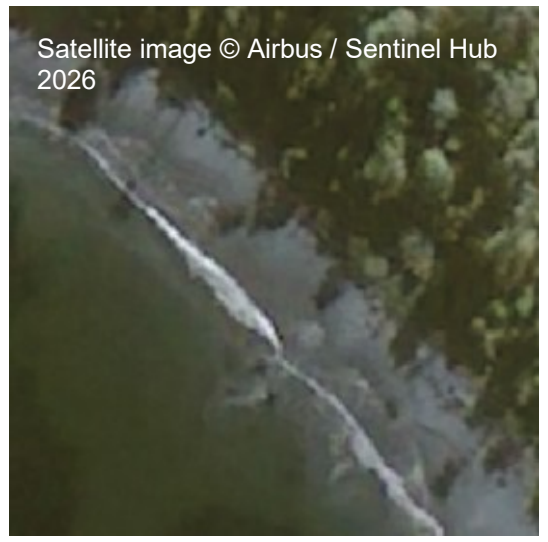	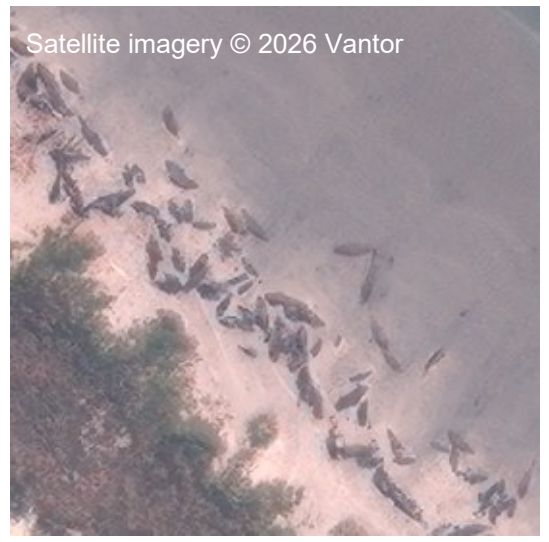 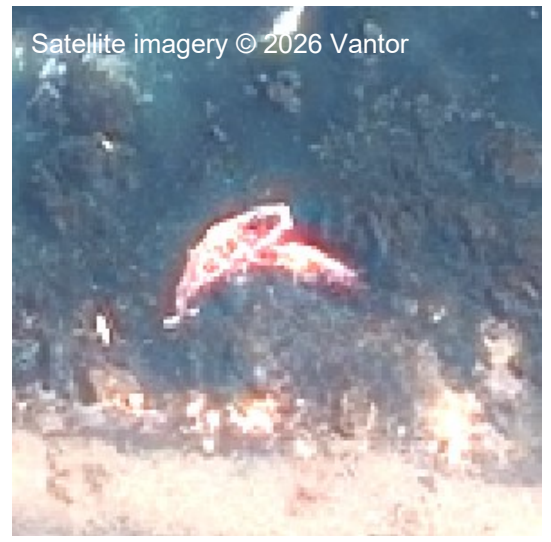

Ice_Cover (ice_cover)

Table S3.10: Satellite image examples of the different ice cover values associated with the standardised attribute 'ice_cover' that could impact feature detection. This attribute is only relevant when reviewing satellite imagery for regions where ice is present.

none	low	medium	dense	very_dense
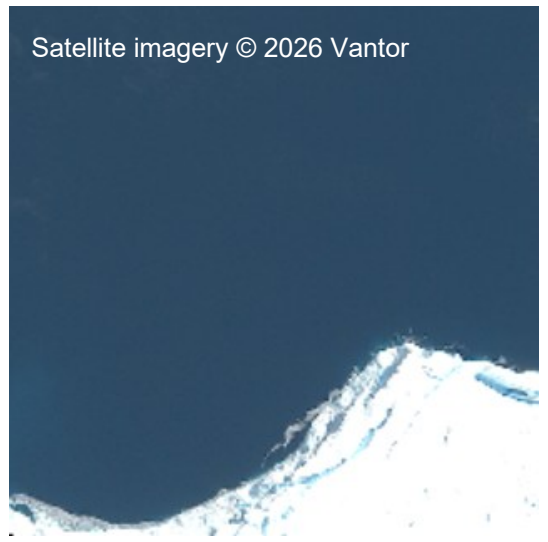	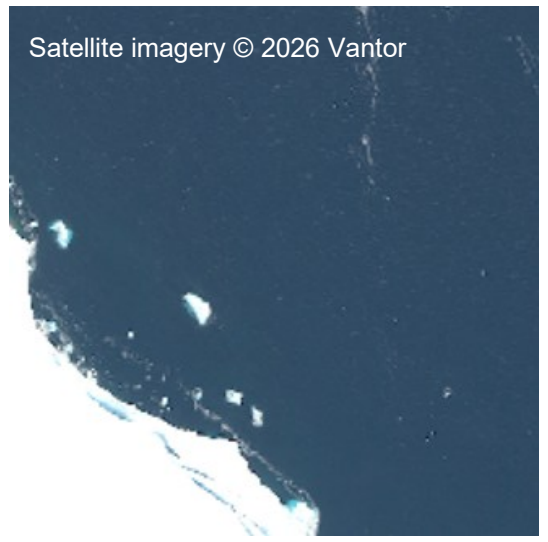	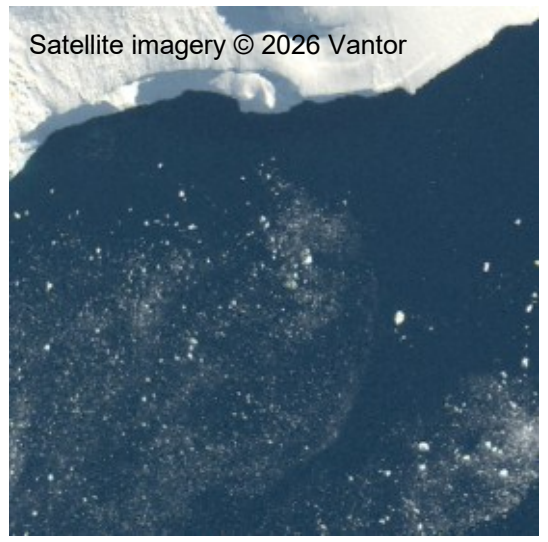	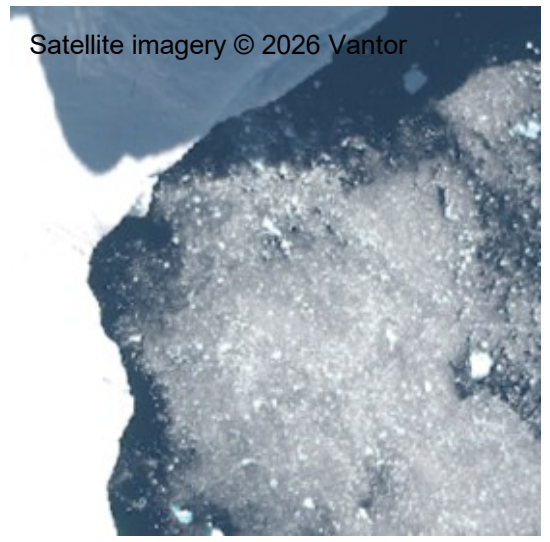	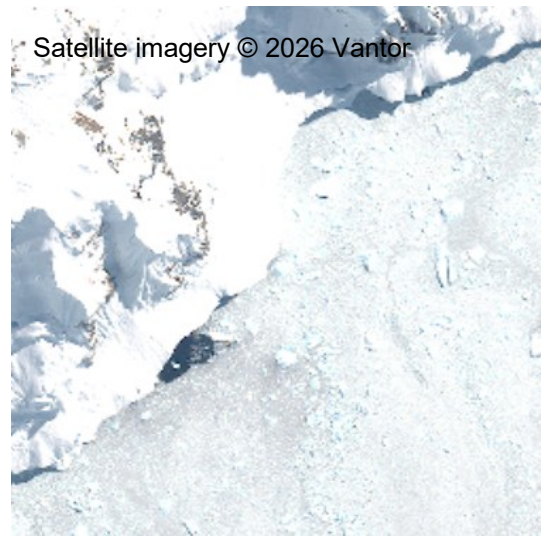

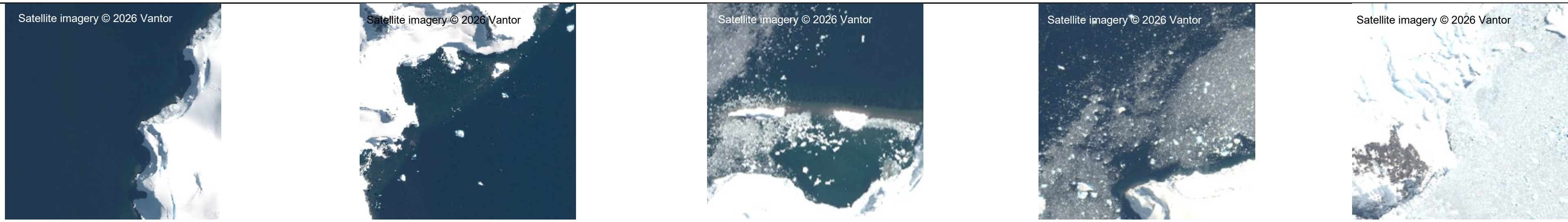

This document is intended to guide users in the detection of stranded cetaceans in optical satellite imagery; however, this data standard / template can be adapted to other remote sensing data types. Therefore, in Figure S3.12, we provide examples of stranded long-finned pilot whale (*Globicephala melas edwardii*) detected in Synthetic Aperture Radar satellite imagery, alongside concurrent (within five hours) optical images. Stranded cetaceans biological, chemical, and physical properties increase the intensity of backscatter to appear bright in SAR.

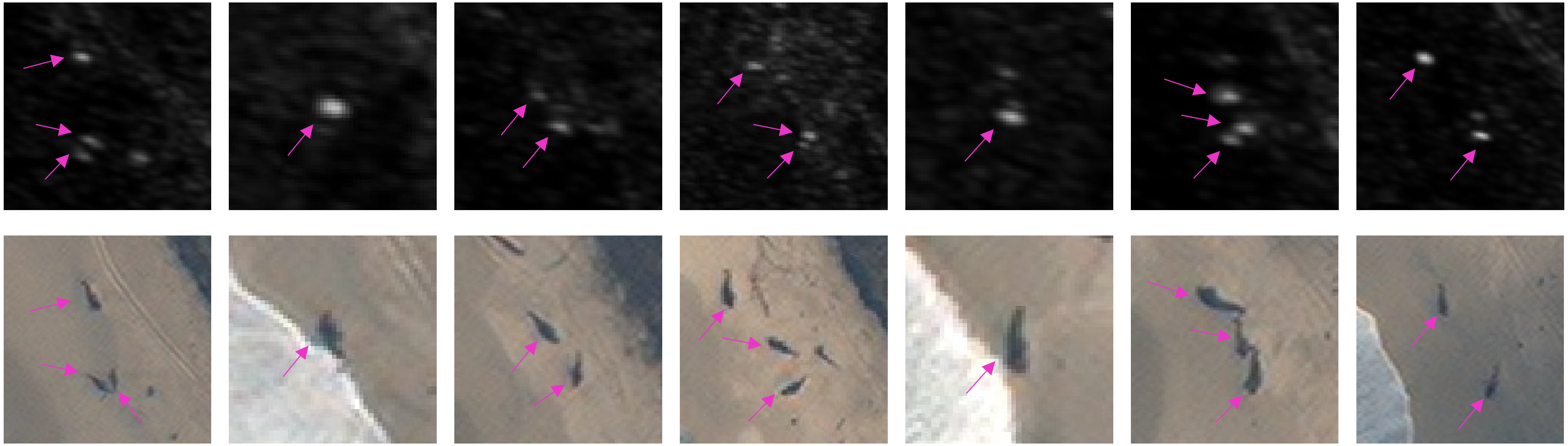

Figure S3.12: Examples of stranded long-finned pilot whale (Globicephala melas edwardii) in Synthetic Aperture Radar (SAR) imagery, alongside concurrent (within 5 hours) optical imagery, *SAR satellite imagery © DLR e.V. 2023, Distribution by Airbus Defence and Space GmbH and optical satellite imagery © 2026 Vantor. Due to the time difference in imagery and animals being moved onshore or washed offshore in the intervening time, stranded cetaceans detected in the SAR imagery may differ in position or are absent in the optical imagery examples.*

References

Clarke, P.J., Cubaynes, H.C., Stockin, K.A., Olavarría, C., de Vos, A., Fretwell, P.T. and Jackson, J.A. (2021) 'Cetacean Strandings From Space: Challenges and Opportunities of Very High Resolution Satellites for the Remote Monitoring of Cetacean Mass Strandings', *Frontiers in Marine Science,* 8, article 650735. Available at: https://doi.org/10.3389/fmars.2021.650735

Committee on Taxonomy (2025) *List of marine mammal species and subspecies*. Society for Marine Mammalogy. Available at: https://marinemammalscience.org/science-and-publications/list-marine-mammal-species-subspecies/ (Accessed: August 17, 2025).

Cubaynes, H.C., Clarke, P.J., Goetz, K.T., Aldrich, T., Fretwell, P.T., Leonard, K.E. and Khan, C.B. (2023) 'Annotating very high-resolution satellite imagery: A whale case study', *MethodsX,* 10, article 102040. Available at: https://doi.org/10.1016/j.mex.2023.102040

Fretwell, P.T., Jackson, J.A., Ulloa Encina, M.J., Häussermann, V., Perez Alvarez, M.J., Olavarría, C. and Gutstein, C.S. (2019) 'Using remote sensing to detect whale strandings in remote areas: The case of sei whales mass mortality in Chilean Patagonia', *PLoS ONE,* 14(11), article e0225209. Available at: https://doi.org/10.1371/journal.pone.0222498

Häussermann, V., Gutstein, C.S., Bedington, M., Cassis, D., Olavarria, C., Dale, A.C., Valenzuela-Toro, A.M., Perez-Alvarez, M.J., Sepúlveda, H.H., McConnell, K.M., Horwitz, F.E. and Försterra, G. (2017) 'Largest baleen whale mass mortality during strong El Niño event is likely related to harmful toxic algal bloom', *PeerJ* 5, article e3123. Available at: https://doi.org/10.7717/peerj.3123/table-7

IJsseldijk, L.L., Brownlow, A.C. and Mazzariol, S. (2019) *Best practice on cetacean post mortem investigation and tissue sampling*. Available at: https://accobams.org/wp-content/uploads/2021/07/Best-practices-on-cetacean-post-mortem-investigation.pdf.

Law, R.J., Jepson, P.D., Deaville, R., Reid, R.J., Patterson, I.A.P., Allchin, C.R. and Jones, B.R. (2006) *Collaborative UK Marine Mammals Strandings Project: summary of contaminant data for the period 1993-2001*. Available at: https://www.cefas.co.uk/publications/techrep/tech131.pdf.

Mądra, A., Frątczak, K., Grzywacz, A. and Matuszewski, S. (2015) 'Long-term study of pig carrion entomofauna', *Forensic Science International,* 252, pp. 1–10. Available at: https://doi.org/10.1016/j.forsciint.2015.04.013

Payne, J.A. (1965) 'A Summer Carrion Study of the Baby Pig Sus Scrofa Linnaeus', *Ecology,* 46(5), pp. 592–602. Available at: https://doi.org/10.2307/1934999
